# Footprint-free human fetal foreskin derived iPSCs: A tool for modeling hepatogenesis associated gene regulatory networks

**DOI:** 10.1038/s41598-017-06546-9

**Published:** 2017-07-24

**Authors:** Peggy Matz, Wasco Wruck, Beatrix Fauler, Diran Herebian, Thorsten Mielke, James Adjaye

**Affiliations:** 1 Max Planck Institute for Molecular Genetics, 14195 Berlin, Germany; 2 Institute for Stem Cell Research and Regenerative Medicine, Heinrich Heine University, 40225 Düsseldorf, Germany; 3 Institute of Biology, Humboldt University of Berlin, 10099 Berlin, Germany; 4 Department of General Pediatrics, Neonatology and Pediatric Cardiology, Heinrich Heine University, 40225 Düsseldorf, Germany

## Abstract

Induced pluripotent stem cells (iPSCs) are similar to embryonic stem cells and can be generated from somatic cells. We have generated episomal plasmid-based and integration-free iPSCs (E-iPSCs) from human fetal foreskin fibroblast cells (HFF1). We used an E-iPSC-line to model hepatogenesis *in vitro*. The HLCs were characterized biochemically, i.e. glycogen storage, ICG uptake and release, UREA and bile acid production, as well as CYP3A4 activity. Ultra-structure analysis by electron microscopy revealed the presence of lipid and glycogen storage, tight junctions and bile canaliculi- all typical features of hepatocytes. Furthermore, the transcriptome of undifferentiated E-iPSC, DE, HE and HLCs were compared to that of fetal liver and primary human hepatocytes (PHH). K-means clustering identified 100 clusters which include developmental stage-specific groups of genes, e.g. OCT4 expression at the undifferentiated stage, SOX17 marking the DE stage, DLK and HNF6 the HE stage, HNF4α and Albumin is specific to HLCs, fetal liver and adult liver (PHH) stage. We use E-iPSCs for modeling gene regulatory networks associated with human hepatogenesis and gastrulation in general.

## Introduction

Human embryonic stem cells (hESCs) derived from inner cell mass cells of the blastocyst undergo symmetric self-renewal and are pluripotent i.e. can give rise to all cells within the three embryonic germ layers-endoderm, ectoderm, mesoderm and also germ cells^1^. Human Induced pluripotent stem cells (iPSCs) were initially derived from dermal fibroblasts by viral transduction mediated over-expression of four embryonic transcription factors OCT4, SOX2, KLF4 and c-MYC or OCT4, SOX2, NANOG and LIN28^2, 3^. Human iPSCs share similar properties with hESCs, however, the integration of pro-viruses into the host genome of viral-derived iPSC is a risk factor for clinical applications in the future^4, 5^. To overcome these drawbacks, non-viral reprogramming methods have been described using non-integrating Sendai viruses, episomal-based plasmid vectors, *in vitro*-derived mRNA and miRNA^5–11^.

The liver is the largest internal organ and hepatocytes are the main functional cells in the liver. Hepatocytes perform a number of complex functions which are essential for life e.g. production of plasma proteins, synthesis of bile acids, the uptake and storage of glucose as well as drug detoxification. The use of primary human hepatocytes (PHH) is problematic, first, they cannot be expanded *in vitro* and second, they are difficult to obtain routinely or in sufficient quantities^12, 13^. Alternatives such as human hepatocarcinoma-derived and transformed, permanent cell lines, including HepG2, THLE and HepaRG, have phenotypes significantly diverged from normal primary hepatocytes^14–16^. A potential alternative could be the differentiation of iPSCs to hepatocyte-like cells. Hepatocyte-like cells (HLCs) generated from human iPSC have shown great promise as an inexhaustible source of cells that mirror the genotype of the donor to satisfy this need. Several groups have already shown how multifunctional applicable HLCs generated from iPSC can serve as cellular models for drug screening and toxicology studies, as a source for disease modeling^17–19^.

To date most studies use viral-derived iPSC to generate HLC, however these have drawbacks (e.g. genome integration). Additionally, most studies focus on one aspect of the multifunctional application of HLCs-derived from iPSCs such as the generation of HLCs from iPSCs, the maturation, modeling liver diseases, drug screening and toxicology^17–24^.

In this study we used an integration-free, episomal-derived induced pluripotent stem cell line (E-iPSC) from human neonatal foreskin fibroblast (HFF1)^25, 26^ to derive and characterize hepatocyte-like cells (HLCs) as well as untangle human hepatogenesis-associated gene regulatory networks.

## Results

### Differentiation of E-iPSCs to hepatocyte-like cells (HLCs)

**Figure 1:**
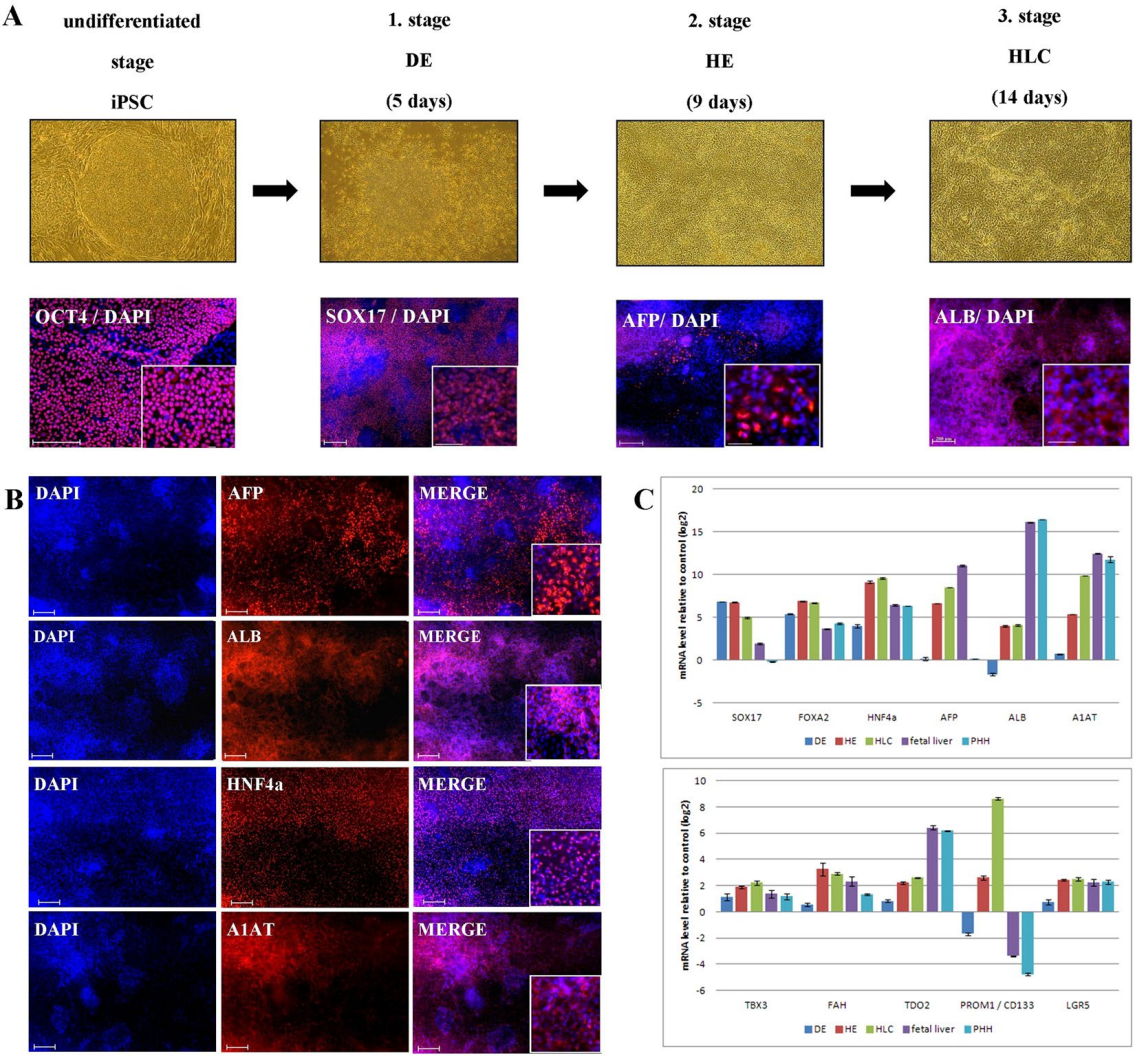
Derivation of hepatocyte-like cells (HLC) from E-iPSCs. (**A**) First row phase contrast images of the differentiation stages, from undifferentiated stage the episomal induced pluripotent stem cells (E-iPSCs) to definitive endoderm (DE), then hepatic endoderm (HE) and finally hepatocyte-like cells (HLCs). Second row immunofluorescence-based staining of stage specific proteins overlapped with DAPI (staining of nucleus). Scale bar: 100 μm Alexa Flour 594 (red). (**B**) Immunofluorescence-based staining of HLC specific proteins AFP, ALB, HNF4α and A1AT. Scale bar: 100 μm Alexa Flour 594 (red). (**C**) Expression patterns of liver specific marker genes during HLC differentiation compared to fetal liver and primary human hepatocytes (PHH) are shown by quantitative real-time PCR (qPCR). Three biological replicates in technical triplicates of each sample were analyzed. The data were normalized to E-iPSCs. The standard deviation is depicted by the error bars.

We derived an integration-free, episomal-based induced pluripotent stem cell line (E-iPSC) from human neonatal foreskin fibroblast (HFF1)^25^ to derive HLCs for this study. The derivation of HLCs was based on a slight modification of the protocol described by Sullivan *et al*.^18^. The differentiation to HLCs consists of three steps (Fig. 1A). First, the cells were differentiated towards definitive endoderm (DE) resulting in down-regulated expression of the pluripotent markers *OCT4*, *SOX2* and *NANOG* and activation of DE specific markers such as *SOX17*. The second step in the protocol resulted in the emergence of hepatic endoderm cells (HE) as defined by the expression of hepatoblasts markers such as *AFP*, *PROM1* and *LGR5* (Fig. 1B,C). Finally, the HE cells were forced into maturation resulting in HLCs expressing mature liver markers such as *ALB*, *A1AT*, *FOXA2*, *HNF4α TBX3*, *FAH* and *TDO2* but still maintaining *AFP* expression (Fig. 1B,C)^20, 27–31^. Quantitative real-time PCR confirmed the results of the immunofluorescence-based detection of protein expression (Fig. 1C).

### Functional analyses of E-iPSC derived HLCs

**Figure 2:**
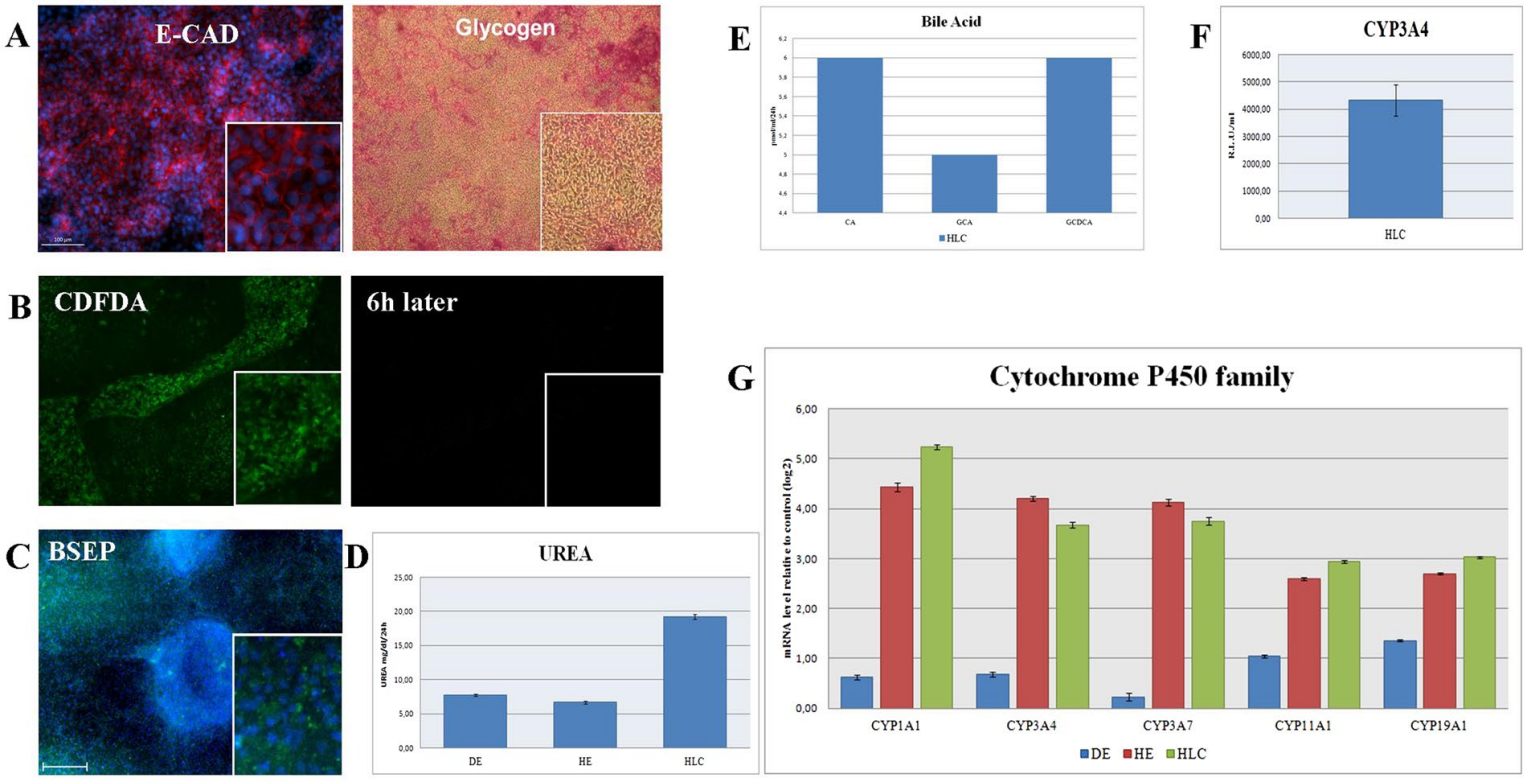
Functional analysis of hepatocyte-like cells (HLCs) derived from E-iPSCs. (**A**) E-Cadherin (E-CAD) antibody staining marking cell shape (left panel), Glycogen storage (right panel), Periodic Acid-Schiff (PAS) assay was used. Glycogen storage is indicated by pink or dark red-purple cytoplasm. (**B**) Visualization of 5 (and 6)-Carboxy-2′,7′-dichlorofluorescein diacetate (CDFDA), immunofluorescence image of HLCs direct after incubation with CDFDA (left panel), and immunofluorescence image of HLCs six hours later (right panel). (**C**) Immunofluorescence-based protein staining of bile salt export pump (BSEP). (**D**) Analysis of UREA production in E-iPSC-DE (DE), E-iPSC-HE (HE) and E-iPSC-HLCs (HLC). Three biological replicates in technical triplicates of each sample were analyzed. The levels of urea are presented as a percentage, considering measured levels of urea in mg/dL/24 h. The error bars indicate the standard errors of the mean. (**E**) Measurement of bile acid secretion of E-iPSC-HLCs (HLC). (**F**) Measurement of CYP3A4 secretion of HLC samples. Three biological replicates in technical triplicates were analyzed. The levels of CYP3A4 are presented as relative light units per milliliter (R.L.U./ml). The error bars indicate the standard errors of the mean. (**G**) Quantitative real-time PCR (qPCR) analysis of cytochrome P450 family member activity of all stages E-iPSC-DE (DE), E-iPSC-HE (HE) and E-iPSC-HLC (HLC) are shown. Three biological replicates in technical triplicates of each sample were analyzed. The data were normalized to E-iPSCs. The standard deviation is depicted by the error bars.

**Figure 3:**
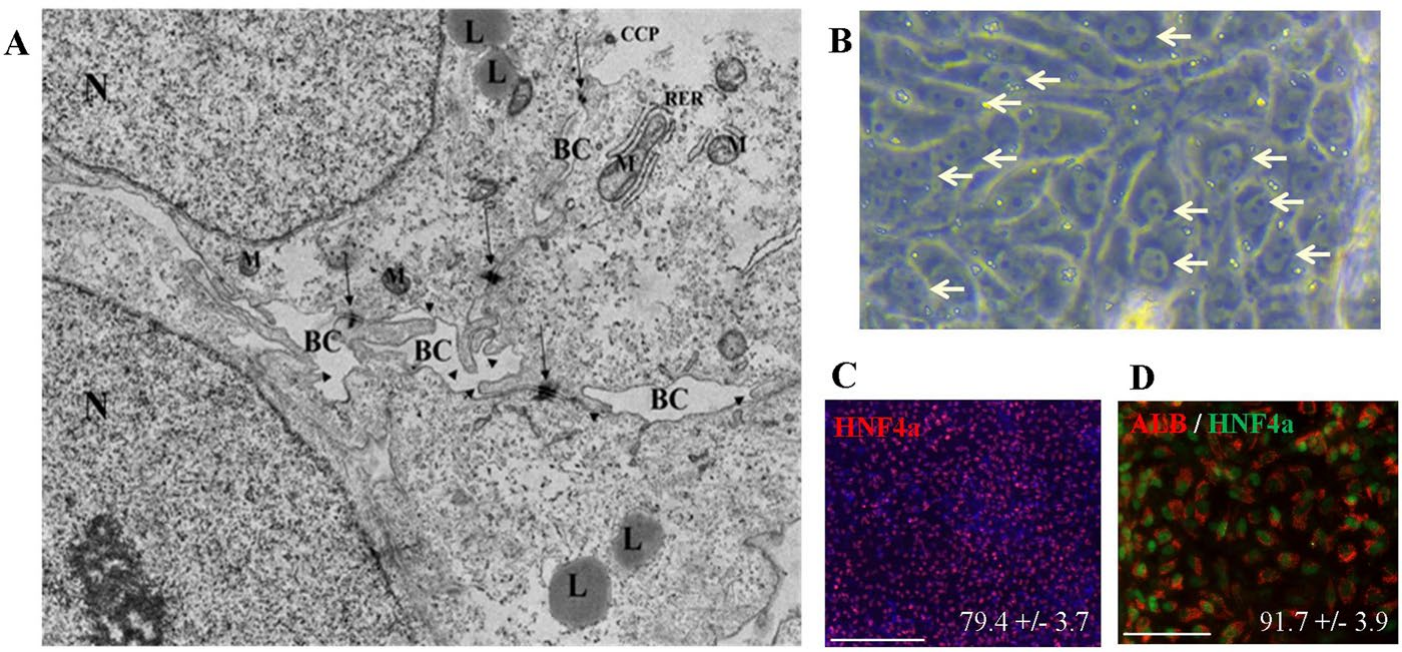
Ultra structure and maturation. (**A**) Electron microscopy image of E-iPSC-HLCs. BC = bile canaliculi; L = lipid; CCP = clathrin coated pits; M = mitochondrion; RER = rough endoplasmic reticulum; N = nucleus; arrow = tight junctions; arrowhead = microvilli. (**B**) Bright field microscopy of HLCs. Bi-nuleated cells are marked by arrow-heads. (**C**) Immunofluorescence-based staining of HNF4α in end-stage HLCs. 79.4% (+/−3.7%) of the cells counted positive for HNF4α. Scale bar: 200 μm Alexa Flour 594 (red). (**D**) Immunofluorescence-based double staining of Albumin (ALB) and HNF4α in end-stage HLCs. 91.7% (+/− 3.9) of HNF4α positive cells were also positive for ALB. Scale bar: 100 μm, Alexa Flour 594 (red) and Alexa Flour 488 (green).

The HLCs bear hallmarks of primary hepatocytes, i.e. (i) cobblestone-shaped epithelial cells expressing E-Cadherin (E-CAD), (ii) the ability to store glycogen as confirmed by Periodic-Acid-Schiff (PAS) staining (Fig. 2A). (iii) Uptake and release of ICG (data not shown) and CDFDA were measured (Fig. 2B). (iv) BSEP was detactable in HLCs by immunofluorescence-based protein staining (Fig. 2C). (v) Urea secretion was measured in all three stages of the differentiation protocol, as anticipated the highest level of production was in HLCs (Fig. 2D). (vi) Bile acids (CA, GCA, GCDCA) were also produced by HLCs (Fig. 2E and Supplementary Figure S1D). (vii) CYP3A4 activity was measured (Fig. 2F). Quantitative real-time PCR and heatmap-based analysis confirmed the expression of CYP3A4 as well as other members of the cytochrome P450 super family of enzymes (Fig. 2G and Supplementary Figure S1A). (viii) Electron microscopy revealed the ultra-structure typical of hepatocytes such as bile canaliculi with microvilli, lipid storage and tight junctions (Fig. 3A). (ix) Bi-nucleated cells could be shown by bright field microscopy (Fig. 3B). Finally, the efficiency of HLC differentiation was scored by HNF4α expression, as well as a double staining of ALBUMIN and HNF4α (Fig. 3C,D).

### Hepatogenesis associated transcriptional road map

**Figure 4:**
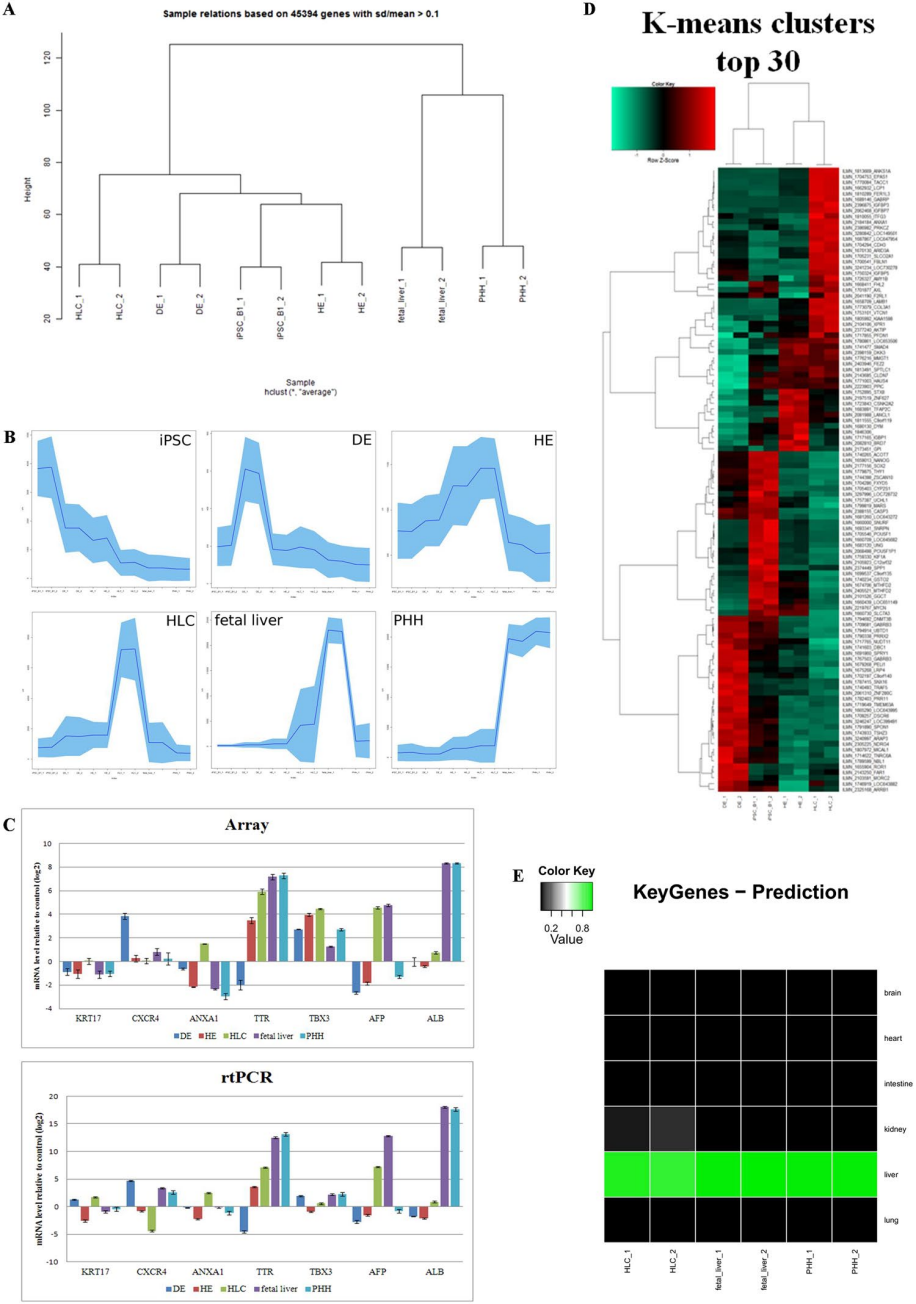
Transcriptional dynamic of hepatocyte-like cells (HLCs) derived from E-iPSCs. (**A**) A cluster dendrogram similarities between replicates and relationship of the samples E-iPSC (iPSC_B1), DE, HE, HLC, fetal liver and PHH. (**B**) K-means cluster 68 contained OCT4 marking undifferentiated stage (iPSC), cluster 81 (sub-cluster 1) contained SOX17 marking DE stage (DE), cluster 37 (sub-cluster 2) represents HE stage (HE), cluster 51 represents HLC stage (HLC), cluster 72 contained AFP marking fetal liver stage (fetal liver) and cluster 91 contained liver marker ALB (PHH). (**C**) Confirmation of microarray data by quantitative real-time (qPCR). On the left hand the array expression data and on the right hand the qPCR expression data of the following genes are shown: *KRT17* and *CXCR4* marking the definitive endoderm stage (DE), *ANXA1*, *TTR* and *TBX3* represents the hepatocyte-like cell stage (HLC), *AFP* marking the fetal liver stage and *ALB* represents the mature liver (PHH) stage. Three biological replicates in technical triplicates of each sample were analyzed. The data were normalized to E-iPSCs. The standard deviation is depicted by the error bars. (**D**) Heatmap of the top 30 genes from each K-means clusters in Fig. 4B. (**E**) KeyGenes prediction for k-means Hepatocyte-like-cell (HLC) cluster9. Data sets for human liver, brain, intestine, kidney, lung and heart were downloaded from NCBI GEO and the KeyGenes tool was employed to generate a training set for these Illumina microarray platform data. As the test set genes from the HLC cluster 9 and HLC, fetal liver and primary human hepatocyte samples were used.

A cluster dendrogram and accompanying correlation co-efficients demonstrates high similarities between replicates. Furthermore, fetal liver and PHH formed a cluster and iPSCs, HE and DE formed a cluster which is then extended by HLCs (Fig. 4A). K-means clustering identified 100 clusters which include developmental stage-specific groups of genes, e.g. OCT4 expression at the undifferentiated stage, SOX17 marking DE stage, HNF6 at the HE stage, PROX1 at the HLC stage, AFP marking the fetal liver stage and ALB marking the mature liver (PHH) stage (Fig. 4B and Supplementary Figure S1B). We further identified upstream regulators of genes within the six selected clusters shown in Fig. 4B by transcription factor over-representation analysis via the oPOSSUM data base^32^. These gene regulatory networks associated with these clusters are presented in Supplementary Figure 3. The network for iPSCs (Supplementary Figure S4A) shows the well known regulatory relations between OCT4 (POU5F1), SOX2, NANOG, KLF4. Most significant factors from the oPOSSUM analysis were STAT1, MZF1 and KLF4 (Z-Score > 10). In the network for DE (Supplementary Figure S4B) SP1, INSM1, MZF1, KLF4, REST are most significant (Z-Score >= 10), in HE (Supplementary Figure S4C) LHX3, MIZF, CTCF, in HLC (Supplementary Figure S4D), PLAG1, EWSR1-FLI1, IRF2, in fetal liver (Supplementary Figure S4E) TAL1::GATA1, HNF1A, ZFN143, GATA1, HNF1B and in primary human hepatocytes (Supplementary Figure S4F) HNF1A, CTCF, ZFX, HNF4A, FOXA2, FOXA1, CEBPA.

**Figure 5:**
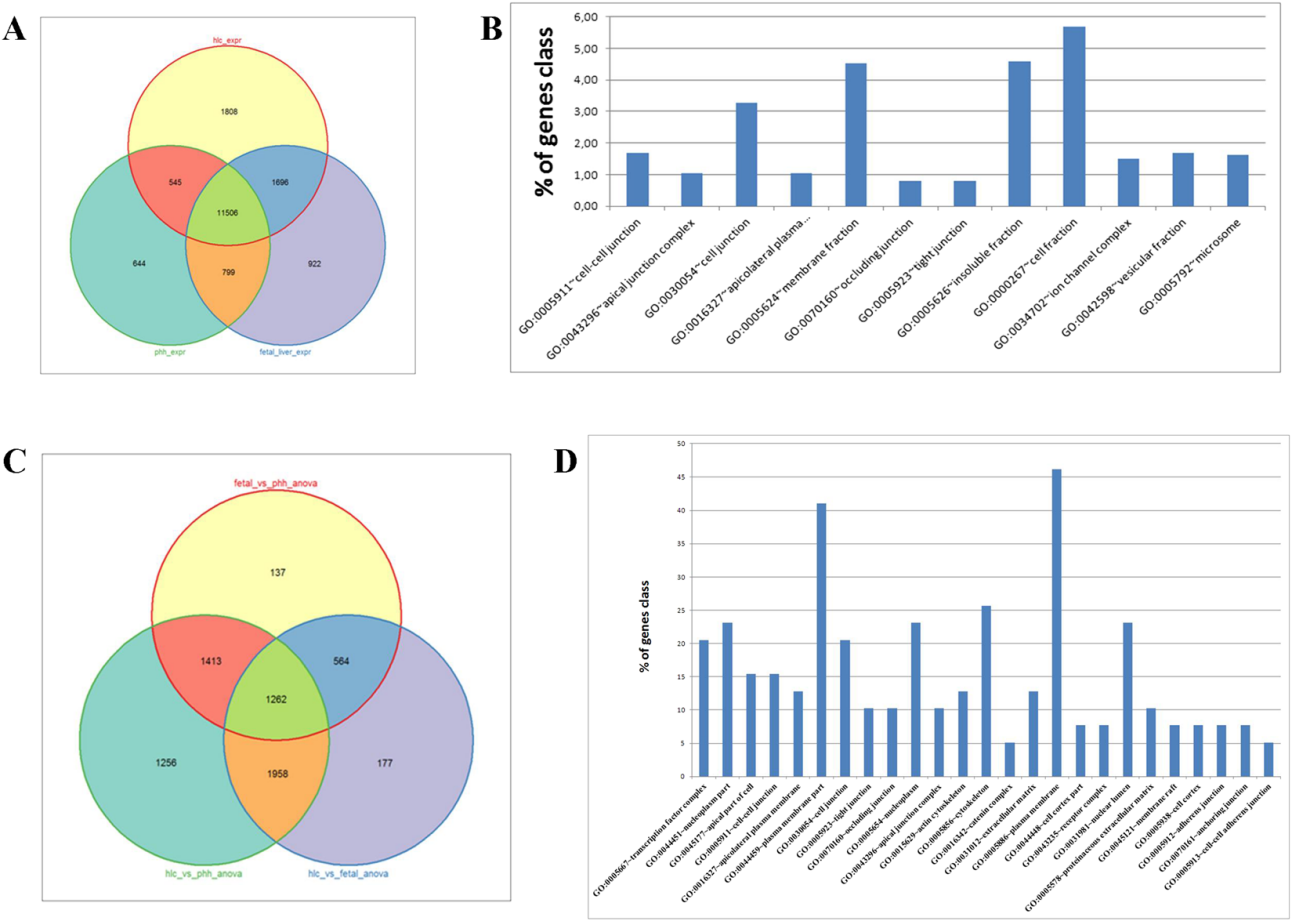
Comparative transcriptome profile analyses. (**A**) Venn diagram of HLCs, fetal liver and PHH. (**B**) GO cellular components of HLC exclusively expressed genes (Fisher extract p < 0.01). (**C**) Venn diagram of fetal liver vs. PHH anova, HLC vs. fetal liver anova and HLC vs. PHH anova. (**D**) GO cellular components of HIPPO pathway genes exclusively expressed genes (Fisher extract p < 0.01) in the intersection of HLC vs. fetal liver anova and HLC vs. PHH anova (intersection with 1958 genes).

The microarray data and quantitative real-time PCR confirmed the expression profile of the listed genes. KRT17 and CXCR4 marking the definitive endoderm stage (DE), ANXA1, TTR and TBX3 represents the hepatocyte-like cell stage (HLC), AFP marking the fetal liver stage and ALB represents the mature liver (PHH) stage (Fig. 4C). Figure 4D shows a heatmap of the top 30 most abundantly expressed genes in each differentiation stage (k-means cluster). In order to assign a tissue-type to the HLCs we applied a tissue prediction tool - KeyGenes^33^ (Fig. 4E and Supplementary Table S1), which confirmed HLC (cluster 9) as liver, comparable to fetal liver and PHH. Furthermore, venn diagram analysis shows the numbers of significantly differentially expressed genes between HLCs, fetal liver and PHHs (Fig. 5A). The HLC-related genes *ANXA1*, *TTR* and *TBX3* as well as the fetal liver-related gene *AFP* and *ALB* representing the matured liver stage PHH are located in the intersection of all three samples and are included in the 11506 genes (Fig. 5A and Supplementary Table S2). A closer look into the exclusive expressed genes in HLCs (1808 genes) uncovered tight junction-specific genes such as *CLDN9*, *CLDN18*, *OCLN*, *PARD6A* and *PARD6B* (Fig. 5B and Supplementary Table S3). One step further, a venn diagram was generated from HLC vs. fetal liver, HLC vs. PHH and fetal liver vs. PHH (Fig. 5C and Supplementary Table S4). DAVID analysis of Hippo signaling related genes from the intersection of HLC vs. PHH and HLC vs. fetal liver dedicated the activity of cell-cell contact related pathways, adherent and tight junction pathway. A chart of these Hippo pathway related genes underlines the predominant expression of cell-cell contact related genes in HLCs (Fig. 5D and Supplementary Table S5). The Hippo pathway, which is responsible for maturation and stabilization of the tight junctions in hepatocytes, and ABC transporters, which are accountable for the uptake and efflux of e.g. bile acids and metabolites, are over represented in HLC, fetal liver and PHH. Bile acid related transporter genes such as *NTCP*, *MRP2*, *ASBT* and *MDR2/3* are highly expressed in HLC compared to the DE and HE stage (Fig. 5D and Supplementary Figure S1C–E).

**Figure 6:**
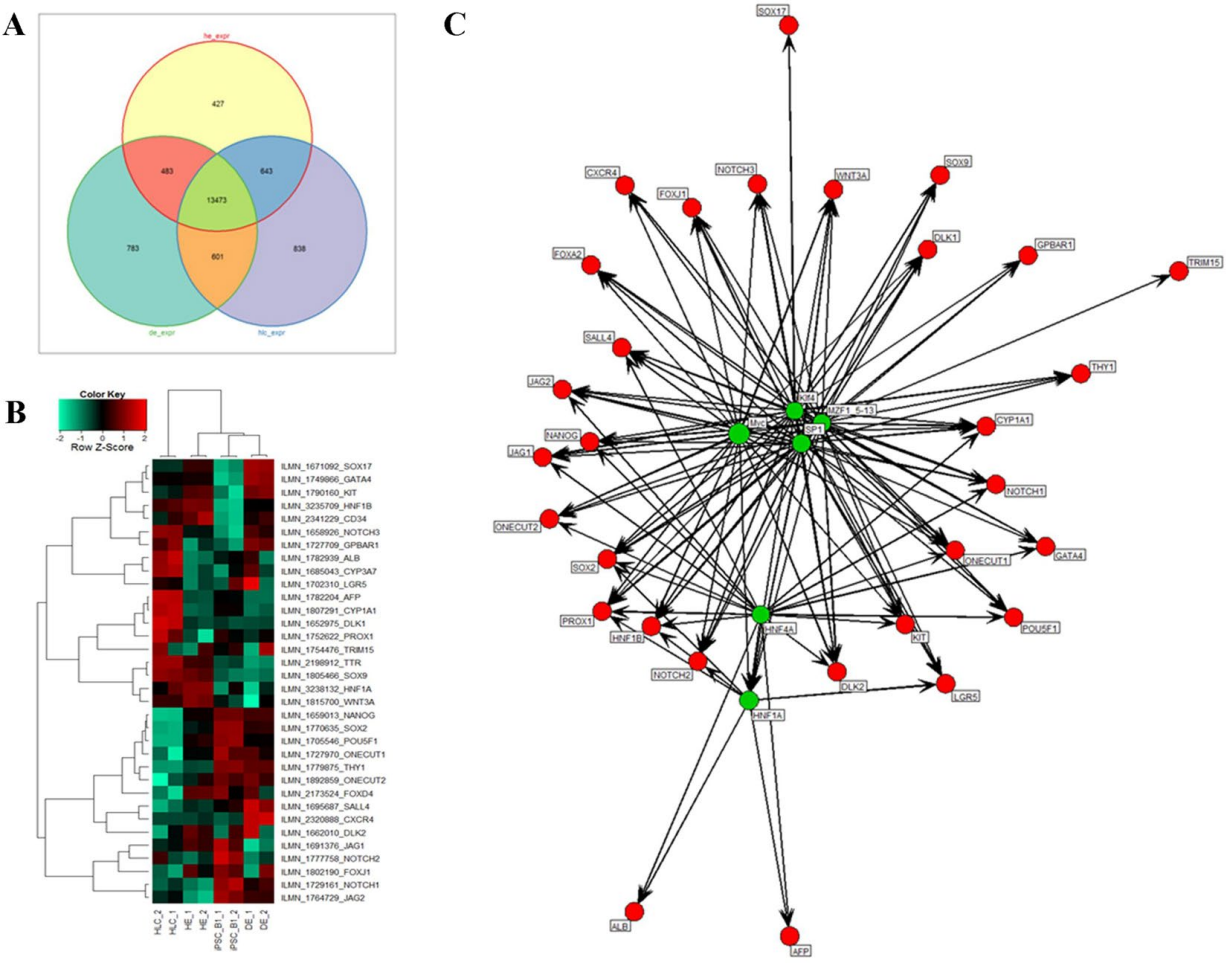
Cell fate decision. (**A**) Venn diagram of DE, HE and HLC. (**B**) Heatmap of bipotential progenitor-associated, hepatocyte-related and cholangiocyte-associated genes. (**C**) An induced network of transcription factors with a Z-score >10 (green, size of circle corresponds to Z-score) and associated genes (red).

A venn diagram of genes which were expressed in DE, HE and HLC shows the relation between the stages of the HLC differentiation (Fig. 6A and Supplementary Table S6). DAVID analysis based on these genes uncover genes which are related to pathways that define the functionality of the liver such as drug metabolism, metabolism of xenobiotics and fatty acid metabolism (Supplementary Figure S2).

### Cell fate determination: hepatocytes or cholangiocytes?

To date most studies on liver cell fate decision making have been conducted in mice^34, 35^. This type of analyses can now be conducted in human using iPSCs. To demonstrate this we generated a heatmap consisting of key genes co- or differentially- expressed in undifferentiated iPSC, DE, HE and HLCs (Fig. 6B and Supplementary Figure S3 and Supplementary Table S7). Progenitor-related genes such as *HNF1A* and *HNF1B* are expressed in HE and HLCs whereas *PROX1* is expressed exclusively in HLCs. Hepatocyte-specific genes such as *ALB*, *AFP*, *ABCB4* and *CYP3A7* are expressed in the HLC samples. Additionally, there exist a group of hepatocyte-related genes which are expressed in HLCs and HE, e.g. *ABCC2*, *RARB* and *TRR*. *WNT3A* as a marker for cholangiocytes as well as *SOX9* and *KRT7* are expressed in both HE and HLCs, whereas *AQP1* and *DLK1* are expressed exclusively in HLCs (Fig. 6A,B). To analyze the relation between these genes for cell fate decision a transcription factor network was created by the use of the data base oPOSSUM^32^ (Supplementary Figure S4). To make it more manageable transcription factors with a Z-score >10 (green circles) and downstream regulating transcription of the progenitor genes (red circles) are shown (Fig. 6C and Supplementary Figure S5). Myc, HNF1A, SP1, MZF1 5–13, HNF4A, Klf4 were the most significant upstream transcription factors (Z-score > 10, p < 1e-15). This demonstrates the dedicated functionalities of this core regulatory network: HNF1A and HNF4A determine the liver fate by regulating ALB and AFP while KLF4 regulates SOX17. Bipotential markers DLK1 and NOTCH3 are both regulated by MYC, Klf4, SP1 and MZF1 5–13. All cholangiocyte marker genes are regulated by KLF4, SP1 and MZF1. *SOX9* is only regulated by these three, while *ONECUT1*, *ONECUT2* and *SALL4* are regulated by these three and additionally by MYC and HNF4A. Furthermore, MYC was the most prominent transcription factor in this network (Z-score of 12.54) and regulates *PROX1* and the transcription factor HNF1A, which is also regulated by HNF4A, as well as the cholangiocyte-related gene *WNT3A* (Fig. 6C, Supplementary Table S8)^28, 36, 37^.

## Discussion

In this study we used an episomal-derived and integration-free iPSC line to model hepatogenesis *in vitro*. Yu *et al*.^6, 26^ showed that iPSCs generated using episomal-based plasmids are free of vector and transgene sequences as we have also shown^25^. A whole-genome sequencing of iPSCs, which were generated by episomal vectors based on the EBNA1/OriP episomal replicon, showed (i) lack of integration of the episomal vector DNA in the host genome, (ii) loss of the episomal vectors in the iPSCs and (iii) no visible changes in the genomes of the iPSCs^26, 38, 39^. The episomal approach is a reliable method for iPSC derivation^40^.

Hepatocytes are the main cell type supporting the detoxification function of the liver and as such they are already extensively used for toxicology screens. Our episomal-derived, viral- and integration-free iPSC line is able to differentiate into hepatocyte-like cells (HLCs) which have similar functional properties as liver-biopsy derived primary human hepatocyes (Figs 1, 2, 3 and 4). These cells can be used for (i) toxicology and drug screening, (ii) future application in tissue replacement therapies, (iii) modeling human diseases *in vitro*.

An important pathway for hepatogenesis is the Hippo-signaling pathway which influences liver cell fate and size^41, 42^. This signal transduction pathway is crucial for early embryonic development, embryonic and adult stem cells, cell proliferation, differentiation, apoptosis, organ size, specific functions in adult organs and tumorgenesis^41, 43–46^. The Hippo pathway is responsible for maturation and stabilization of the tight junctions in hepatocytes^47^ and is over represented in HLCs, fetal liver and PHH. The existence of tight junctions in E-iPSC-derived HLCs is shown by electron microscopy (Figs 3A and 5D). ABC transporters, which are accountable for the uptake and efflux of e.g. bile acids, are also over represented in HLCs, fetal liver and PHH (Fig. 4D). Bile acid measurement shows the excretion of primary bile acids in HLCs. Primary bile acids are involved in drug metabolism and synthesis of cholesterol, steroids and other lipids^48^. Hepatocytes synthesize primary bile acids which carried out by the gut microbiota convert to secondary bile acids by several reactions including dehydroxylation, dehydrogenation and epimerization^49^. A heatmap of bile acid related transporter genes underlines the functionality of the HLCs and shows the highest expression of *NTCP*, *MRP2*, *ASBT* and *MDR2*/*3* in the HLC samples (Fig. 4D).

A cluster dendrogram shows the highest similarities between replicates. Furthermore, fetal liver and PHH formed a cluster and iPSCs, HE and DE formed a cluster which is then extended by HLCs (Fig. 4A). Differences and commonalities between HLCs, fetal liver and PHH are assessed by statistical tests vs. iPSCs and pairwise statistical tests of the three experiments vs. each other. Most genes (4953) are in the intersection set common to all three experiments. Pairwise intersections fetal liver/PHH have 2823 genes, HLCs/PHH 1699 genes and HLCs/fetal liver 988 genes (Supplementary Table S9).

To assess the differences between HLCs, fetal liver and PHH an ANOVA followed by pairwise t-tests was performed. Venn diagram shows the numbers of significantly differentially expressed genes in these pairwise t-tests after filtering for the most variable genes found in the ANOVA (Fig. 5A).

Transcription factor binding site analysis revealed transcription factors are over-represented in these clusters. Top transcription factors by z-score were STAT1, MZF1, KLF4, SP1 and IRF1 for iPSCs, ELF5, FEV, INSM1, FOXI1 and STAT1 for DE, LHX3, MIZF, CTCF, NR3C1 and PAX6 for HE, PLAG1, EWSR1-FLI1, IRF2, MEF2A and ELF5 for HLCs, TAL1:GATA1, HNF1A, ZNF143, GATA1 and HNF1B for fetal liver, HNF1A, CTCF, HNF4A and FOXA2 for PHH (data not shown).

The cluster of iPSCs, definite endoderm (DE) and hepatic endoderm (HE) is successively extended by hepatocyte-like cells (HLCs), fetal liver and primary human hepatocytes (PHH). In most cases fetal liver and PHH are located in one cluster as well as iPSCs, DE and HE are formed another cluster. HLCs mostly are located in between the fetal liver/PHH and the early differentiation stage cluster (Fig. 4A).

Liver specific analysis of genes which are exclusively expressed in HLCs by venn diagram, PaGenBase and DAVID uncover the functionality of the HLC derived from E-iPSCs by showing the activity of cell-cell contact related pathways as well as liver-specific metabolism pathways such as drug metabolism, metabolism of xenobiotics and fatty acid metabolism (Figs 5 and 6 and Supplementary Figure S2).

We demonstrated the feasibility of using iPSCs as *in vitro* models for studying liver cell fate decision making. The heatmap presented in Fig. 6B shows differential expression of key cell fate regulating genes specific to the hepatic endoderm and in some cases also in the HLCs. This implies that the HLCs also harbor cell populations with bipotential properties similar to hepatoblasts. For example, the hepatic endoderm cells express *DLK2* and also a set of transcription factors specific to this stage (Onecut1/HNF-6) which are not expressed in HLCs. These set of transcription factors are putative candidates for directing biliary epithelial cells/cholangiocytes cell fate. However, HLCs express *DLK1* and progenitor-related genes such as *PROX1* and *LGR5* which are not expressed in HEs. Genes such as *WNT3A*, *NOTCH3*, *HNF1A*, *HNF1B* and *SOX9* are expressed in both stages (Fig. 6B). Our overall findings based on gene expression patterns supports the notion that hepatic endoderm (HE) and HLCs in our hepatocyte differentiation protocol are equivalent to the DLK, HNF6 and SOX9-positive bipotential hepatoblasts present in fetal liver and are common progenitors for hepatocytes and biliary epithelial cells/cholangiocytes (Fig. 6B)^34, 35^.

The Notch signaling, hepatocyte nuclear factor-6 (HNF-6) and PROX1 are amongst factors known to regulate lineage commitment in the bipotential hepatoblast progenitor cell population^35, 50, 51^. It should be possible to change the fate of HE cells by manipulating the expression levels of e.g. *PROX1*, *SOX9*, and *HNF6* or even by using small molecules targeting for instance Notch signaling.

Odom *et al*.^52^ described a core transcriptional regulatory circle in human hepatocytes consisting of six transcription factors (ONECUT1/HNF-6, FOXA2, HNF1A, HNF4A, CREB1 and USF1). These transcription factors bound promoters that are central for liver development and function. For this they used a mixture of human hepatocytes from multiple healthy donors to maximize the diversity of gender and age^52^. Our study shows the expression of the transcription factors *ONECUT1/HNF-6*, *FOXA2*, *HNF1A*, *HNF4A* in human HLCs derived from human E-iPSCs. Furthermore, we have demonstrated that amongst other transcription factors, HNF1A and HNF4A are involved in orchestrating cell fate decision of bipotential hepatoblast cells to become either hepatocytes or biliary epithelial cells/cholangiocytes (Fig. 6C).

The generation of a transcription factor network via the oPOSSUM data base^32^ uncovered transcription factors which are involved in cell fate decisions during hepatogenesis. The most prominent transcription factor in our network is MYC -transcription factors which regulates numerous biological processes such as glycolysis, cell proliferation and differentiation^53, 54^. MYC also regulates the expression of bipotential hepatoblast-related genes (e.g. *DLK1*, *PROX1*), cholangicyte-related genes (e.g. *ONECUT1*, *WNT3A* and *SALL4*) as well as hepatocyte-related genes (e.g. *HNF1A*). The second prominent transcription factor is HNF4A- a central regulator of hepatocyte differentiation and function^55^ and regulates hepatocyte-related genes (e.g. *ALB*, *AFP*), cholangiocyte-related genes (e.g. *ONECUT1*, *SALL4*) as well as the hepatoblast-related gene *PROX1* (Fig. 6C, Supplementary Table S8)^28, 36, 37^. his transcription network underlines the bipotential progenitor-related characteristics of the HE and HLC stage in our differentiation procedure. This implies that there are bipotential progenitors within HE and HLC cell populations.

K-means clustering identified developmental stage-specific groups of genes, e.g. OCT4 expression at the undifferentiated stage, SOX17 marking the DE stage, DLK and HNF6 the HE stage, HNF4A and Albumin is specific to HLCs, fetal liver and adult liver (PHH) stage gain an insight into hepatogenesis. Furthermore, gene regulatory networks generated by oPOSSUM data base uncovered the presence of bipotential progenitor populations in both stages in HE and HLC. This analysis should lay the foundation for future efforts to generate long-term cultures of cholangiocytes and HLCs.

The bipotential progenitor population in iPSC-derived HLCs and the presence of AFP expression underline their fetal status. However, we uncovered human hepatogenesis-associated gene regulatory networks which are involved in cell fate decision making during hepatogenesis.

## Conclusion

In summary, we have demonstrated the derivation of integration-free E-iPSCs from somatic cells and differentiated these to hepatocyte-like cells (HLCs) capable of storing glycogen, ICG uptake and release, UREA and bile acid production, as well as CYP3A4 activity. Ultra-structure analysis by electron microscopy revealed the presence of lipid and glycogen storage, tight junctions and bile canaliculi- all typical features of biopsy-derived primary hepatocytes. Model organisms such as zebrafish or mouse are used in order to analyze developmental processes of hepatogenesis^34, 35, 56^. HLCs derived from human E-iPSCs can be used to analyze the human hepatogenesis in details. We uncovered a gene regulatory network which uncovered the presence of bipotential progenitor populations in HE and HLC stage. Additionally, MYC was identified as a prominent regulator of bipotential hepatoblast-related genes expression.

## Methods

### Cell Culture and Differentiation

Human neonatal foreskin fibroblasts HFF1 were purchased from ATCC (HFF1 #SCRC-1041) and were maintained in Dulbecco’s modified Eagle medium (DMEM^TM^, Gibco) containing 10% fetal bovine serum (FBS^TM^, Invitrogen) and 0.5% penicillin and streptomycin (Invitrogen). Human ES and iPS cells were maintained on irradiated mouse embryonic fibroblast (MEF) cells in KnockOut^TM^ DMEM (Invitrogen) supplemented with 20% KnockOut^TM^ Serum Replacement (Invitrogen), 0.1 mM non-essential amino acids (Invitrogen), 0.1 mM L-glutamine (Invitrogen), 0.1 mM ß-Mercaptoethanol (Sigma), 0.5% penicillin and streptomycin and 8ng/ml basic fibroblast growth factor (bFGF, Invitrogen) as described by Wolfrum *et al*.^57^. The human ESC lines H1 and H9 were purchased from WiCell Research Institute (Madison, WI, USA, www.wicell.org, #WA01 and #WA09).

All used cells and cell lines were cultured at 37 °C and 5% CO2 in an incubator (INNOVA CO-170 Incubator, New Brunswick Scientific) under humidified atmosphere. All treatments and maintenance procedures were carried out using a clean bench type HeraSafe (Haereus Instruments).

For differentiation iPSC into HLC the protocol from Sullivan *et al*.^18^ was used. We modified the last step of HLC generation. During the last step of differentiation we used 25ng/ml dexamethasone instead of 10 μM hydrocortisone 21-hemisuccinate.

### Functional Assays for HLC

#### PAS staining

Glycogen storage was identified by Periodic Acid-Schiff (PAS) Staining System (Sigma-Aldrich). Cells were fixed with 4% paraformaldehyde for 15 min and stained according to the manufacturer’s instructions.

#### Uptake and release

To detect the uptake and release of substances ICG (indocyanine green; Cardiogreen, ICG; Sigma) and CDFDA (5 (and 6)-Carboxy-2′,7′-dichlorofluorescein diacetate, CDFDA; Sigma) were used. The cells were incubated in culture medium with freshly diluted ICG (1 mg/ml) or CDFDA (1 μM) for 30 min. at 37 °C. The cells were washed with PBS, fresh culture medium was added and uptake of dye was documented. The release of ICG and CDFDA was examined after 6 h. The results of ICG and PAS assays were examined under an Olympus CK2 phase-contrast microscope and representative morphology was recorded at a magnification of ×50 using a Canon 300D digital camera. The fluorophore of CDFDA was visualized using a Zeiss, LSM 510 Meta confocal microscope with a connected camera for microscopy model AxioCam ICc3 and the software Axiovision 4.6 at a magnification of ×200.

#### Urea measurement

Urea secretion was quantified by a colorimetric assay QuantiChrom^TM^ Urea Assay Kit (DIUR-500 BioAssay Systems) following the manufacturer’s instructions. The assay detects urea directly by using substrates that specifically bind urea. Urea assays were carried out in 96-well plates, and concentrations were measured using a plate reader. Analysis of urea production in E-iPSC-DE (DE), E-iPSC-HE (HE) and E-iPSC-HLC (HLC) were performed. Three biological replicates in technical triplicates of each sample were analyzed. The levels of urea are presented as a percentage, considering measured levels of urea in mg/dL/24 h.

#### Bile acid measurement

Bile acids (cholic acid CA, chenodeoxycholic acid CDCA, deoxycholic acid DCA, ursodeoxycholic acid UDCA, lithocholic acid LCA) including their glycine- and taurine derivatives were analyzed by UPLC-MS/MS. The system consisted of an Acquity UPLC-H Class (Waters, UK) coupled to a Xevo-TQS tandem mass spectrometer (Waters, UK) which is equipped with an ESI source operating in the negative ion mode. Quantitative data were conducted in the multiple reaction monitoring (MRM) mode. The chromatographic separation was performed on Waters UPLC BEH C18 column (100 mm, 2.1 mm ID, 1.7 µm; Waters, UK) using acetonitrile and acidic water (0.1% formic acid) as mobile phases. Analytes were separated by a gradient elution. The injection volume was 5 µL and the column was maintained at 40 °C.

#### CYP3A4 measurement

CYP3A4 activity was measured by using the pGlo kit (Promega) according to manufacturer’s instruction for nonlytic CYP450 activity estimation. The CYP3A4 production was measured in E-iPSC-HLC (HLC). Three biological replicates in technical triplicates of each sample were analyzed. The levels of CYP3A4 are presented as relative light units per milliliter (R.L.U./ml). The error bars indicate the standard errors of the mean.

### Electron microscopy

Cells grown on Thermanox® plastic coverslips (Nunc), were fixed in a modified Karnofsky solution, 2%PFA/2,5%GA in 50 mM Cacodylate buffer, pH7.4 at 4 °C. Cells were washed in 50 mM Cacodylatpuffer/50 mM NaCl and post-fixed for 90 min at room temperature with 0,5% OsO_4_ in the same buffer. After washing steps with water, cells were incubated for 40 min with 0,1% tannic acid in 250 mM Hepes pH7,4, washed with water and stained with 2% uranyl acetate, 90 min at room temperature. Cells were dehydrated in a graded series of ethanol and embedded in Spurr’s resin (Low Viscosity Spurr Kit, Ted Pella, CA, USA). Ultra-thin sections (70 nm) were prepared with an ultramicrotome (Reichert Ultracut E, Leica) and mounted on pioloform-coated slot grids from copper. Sections were counterstained with uranyl acetate and lead citrate.

Ultrathin-sections were first examined using a Philips CM100 transmission electron microscope operated at 100 kV and finally imaged using a FEI Tecnai Spirit transmission electron microscope operated at 120 kV, which was equipped with a 2 k × 2 k Eagle CCD camera (FEI). The MSI-Raster application within the Leginon Software package^58^ was used to automatically image selected regions of interest at a final nominal magnification of 15000× applying a defocus of −4 µm. Raw micrographs were stitched using the Trakem2 plugin implemented in the Fiji software platform^59, 60^.

### Microarray -Based Gene Expression Analysis

Total RNA from iPSC, DE, HE, HLC, PHH and fetal liver in replicates were extracted using the MiniRNeasy Kit (Qiagen) according to the manufacturer’s instructions and quality checked by Nanodrop analysis (Nanodrop Technologies, Wilmington, DE, USA, http://www.nanodrop.com). Approximately 500 ng of DNase treated RNA was sent to ATLAS Biolabs (http://www.atlas-biolabs.de) for whole transcriptome analysis by using microarray. All basic expression data analysis was carried out using the BeadStudio software 3.0. Raw data were background-subtracted and normalized using the “rank invariant” algorithm and then filtered for significant expression on the basis of negative control beads. For correlation coefficient analysis and the generation of Venn diagrams, detected gene expression was defined by a Detection P Value < 0.01 as output by BeadStudio. For differential gene expression analyses, genes had to be at least 1.5 fold up- or down-regulated in a group-wise comparison, to be considered significantly differentially expressed.

For more detailed information see supplementary Materials and Methods.

### Data access

GEO Submission (GSE66282).

## Electronic supplementary material

Supplementary Information
