# Supplementary material for: Footprint-free human fetal foreskin derived iPSCs: A tool for modeling hepatogenesis associated gene regulatory networks
Source: Sci Rep. 2017 Jul 24;7:6294. doi: 10.1038/s41598-017-06546-9 (PMC5524812; doi:10.1038/s41598-017-06546-9)
Supplement: Supplementary file 1 — Supplementary Information [file 41598_2017_6546_MOESM1_ESM.pdf]

Supplementary Data

Footprint-free human fetal foreskin derived iPSCs: a tool for modeling hepatogenesis associated gene regulatory networks.

Peggy Matz[1,2,3] , Wasco Wruck[2], Beatrix Fauler[1], Diran Herebian[4], Thorsten Mielke[1] and James Adjaye[1,2*]

[1]Max Planck Institute for Molecular Genetics, 14195 Berlin, Germany

[2]Institute for Stem Cell Research and Regenerative Medicine, Heinrich Heine University, 40225 Düsseldorf, Germany

[3]Institute of Biology, Humboldt University of Berlin, 10099 Berlin, Germany

[4]Department of General Pediatrics, Neonatology and Pediatric Cardiology, Heinrich Heine University, 40225 Düsseldorf, Germany

***Contact information:**

James Adjaye, Prof. Dr., Institute for stem cell research and regenerative medicine, Medical faculty, Heinrich Heine University, Moorenstraße 5, D 40225 Düsseldorf, Germany, Tel: 0049-211-81-08191, Fax: 0049-211-81-19147, Email: James.Adjaye@med.uni-duesseldorf.de

Supplementary Figure S1: Global gene analysis.

(A) Heatmap of cytochrome P450 family members. The transcriptome profile of the following samples were compared: mature liver (PHH), fetal liver, Hepatocyte-like cells derived from E-iPSCs (HLC), hepatic endoderm derived from E-iPSCs (HE), definitive endoderm derived from E-iPSCs (DE), E-iPSCs (iPSC_B1). (B) 100 k-means cluster are listed. The used k-means cluster for figure 4B are marked with asterisk. One asterisk = iPSC k-means cluster, two asterisk = DE k-means cluster, three asterisk = HE k-means cluster, four asterisk = HLC k-means cluster, five asterisk = fetal liver k-means cluster and six asterisk = PHH k-means cluster. (C) Heatmap of ABC transporter-related genes. The transcriptome profile of the following samples were compared: mature liver (PHH), fetal liver, Hepatocyte-like cells derived from E-iPSCs (HLC), hepatic endoderm derived from E-iPSCs (HE), definitive endoderm derived from E-iPSCs (DE), E-iPSCs (iPSC_B1). (D) Heatmap of bile acid transporter-related genes. The transcriptome profile of the following samples were compared: E-iPSCs (iPSC_B1), definitive endoderm derived from E-iPSCs (DE), hepatic endoderm derived from E-iPSCs (HE), Hepatocyte-like cells derived from E-iPSCs (HLC). (E) Heatmap of HIPPO pathway-related genes. The transcriptome profile of the following samples were compared: mature liver (PHH), fetal liver, Hepatocyte-like cells derived from E-iPSCs (HLC), hepatic endoderm derived from E-iPSCs (HE), definitive endoderm derived from E-iPSCs (DE), E-iPSCs (iPSC_B1). A scheme of the HIPPO signaling pathway is shown.

Supplementary Figure S2: Liver specification.

(A) KEGG pathway analysis of HLC-tissue specific genes resulted in the listed pathways. For that a DAVID analysis was done from the exclusively expressed genes in HLCs from the venn diagram in figure 6A (838 genes from Supp Table 6). (B) Pie chart illustrating tissue distribution of genes exclusively expressed in HLCs. The list of exclusively expressed genes in HLCs (from venn analysis figure 5A) was analysed employing the PaGenBase database. PaGenBase is a database for the collection of tissue- and time-specific pattern genes, including specific genes, selective genes, housekeeping genes and repressed genes (Pan et al. 2013; http://bioinf.xmu.edu.cn/PaGenBase/).

Supplementary Figure S3: Comparison of gene expression of a-priori-known stage-specific markers in multiple pluripotent stem cell lines differentiated into HLCs leading to the central transcription factor network.

(A) heatmap of the stage specific markers listed in Supplementary Figure S5A from this study (Figure 6B) , (B) unpublished data of differentiating ES into HLCs related to the sudy by Jozefczuk et al.[1] and (C) published data from the study by Jozefczuk et al.[1] differentiating iPSCs into HLCs.

Supplementary Figure S4: Gene regulatory networks.

This figure presents clusters associated with the gene regulatory networks from Figure 4B (transcription factor over-representation analysis via the oPOSSUM data base, Kwon et al. 2012). (A) The network for iPSCs shows the regulatory relations between OCT4 (POU5F1), SOX2, NANOG, KLF4. (B) The network for DE demonstrates the regulatory relations between SP1, INSM1, MZF1, KLF4 and REST. (C) The network for HE represents the regulatory relations between LHX3, MIZF and CTCF. (D) The network for HLC illustrates the regulatory relations between PLAG1, EWSR1-FLI1 and IRF2. (E) The network for fetal liver shows the regulatory relations between TAL1::GATA1, HNF1A, ZFN143, GATA1 and HNF1B. (F) The network for primary human hepatocytes (PHH) represents the regulatory relations between HNF1A, CTCF, ZFX, HNF4A, FOXA2, FOXA1 and CEBPA.

Supplementary Figure S5: Scheme for construction of the hepatocyte differentiation transcription factor network.

(A) A-priori-known markers refer to the stages iPSC/ESC (red), DE (yellow), HE (blue) and HLC (green) in the gene expression data and at least one additional cell line. Transcription factor analysis via oPOSSUM3 delivers transcription factors regulating this gene set. (B) Z-score from oPOSSUM3-analysis is plotted vs. GC content. Myc, HNF1A, HNF4A, SP1, MZF1 and KLF4 are most significant with the highest Z-scores greater than 10. (C) The most significant transcription factors with a Z-score greater than 10 (green) are connected with the genes they regulate (red) from the list (A).

Supplementary Tables

Supplementary Table S1: KeyGenes corresponding to Figure 4E.

(A) KeyGenes prediction matrix. KeyGenes Identity score of the queried samples Hepatocyte-like-cells (HLCs), fetal liver and primary human hepatocyte (PHH) to the samples from the training set. (B) KeyGenes classifier genes. Genes determined by the KeyGenes tool as classifiers for the tissue types provided in the training set.

Supplementary Table S2: Gene lists of venn diagram Figure 5A.

Gene names of all expressed genes in hepatocyte-like cells (HLCs), HLCs and fetal liver, fetal liver, fetal liver and primary hepatocytes (PHH), in PHH, PHH and HLCs as well as genes which were expressed in all samples. Gene list relates to venn diagram in Figure 5A.

Supplementary Table S3: Gene list of exclusively expressed genes in HLCs.

List of gene names related to figure 5B GO cellular components of HLCs exclusively expressed genes (Fisher extract $p<0.01$). Data set from venn diagram figure 5A genes which were exclusively expressed in HLCs (1806 genes, Supplementary Table S2) were used for this analysis in DAVID.

Supplementary Table S4: Gene lists of the venn diagram Figure 5C.

Gene names of all expressed genes in hepatocyte-like cells (HLCs) vs. fetal liver, fetal liver vs. primary hepatocytes (PHH) and HLCs vs. PHH as well as genes expressed in the intersection and in all samples. Gene list relates to venn diagram in Figure 5C.

Supplementary Table S5: Gene list related to Hippo pathway.

List of gene names related to figure 5D GO cellular components of Hippo pathway in the intersection of HLCs vs. fetal liver and HLCs vs. PHH (Fisher extract $p<0.01$). Data set from venn diagram Figure 5C genes which were expressed in HLCs vs. fetal liver and HLCs vs. PHH (1958 genes, Supplementary Table S4) were used for this analysis in DAVID.

Supplementary Table S6: Gene lists of the venn diagram Figure 6A.

Gene names of all expressed genes in definitive endoderm (DE), hepatic endoderm (HE) and hepatocyte-like cells (HLCs) as well as genes which were expressed in the intersections and in all samples. Gene list relates to venn diagram in Figure 6A.

Supplementary Table S7: List of genes used for heatmaps in Supplementary Figure S3 and regulatory network analysis via oPOSSUM data base.

List of priori-known markers refer to the stages iPSC/ESC (red), DE (yellow), HE (blue) and HLC (green) in the gene expression data and at least one additional cell line.

Supplementary Table S8: Regulatory network analysis via oPOSSUM data base.

List of transcription factors (TFs) with their classification, JASPAR ID, target gene hits, transcription factor binding site (TFBS) hits and Z-score. The oPOSSUM database (Kwon et al. 2012) was used. TFs list relates to Figure 6C.

Supplementary Table S9: Gene lists of a venn diagram.

Gene names of all expressed genes in hepatocyte-like cells (HLCs) vs. induced pluripotent stem cells (iPSCs), fetal liver vs. iPSCs and PHH vs. iPSCs as well as genes which were expressed in the intersections or all samples generated via venn diagram analysis.

Differences and commonalities between HLCs, fetal liver and PHH are assessed by statistical tests vs. iPSCs and pairwise statistical tests of the three experiments vs. each other. Most genes (4953) are in the intersection set common to all three experiments. Pairwise intersections fetal liver/PHH have 2823 genes, HLCs/PHH 1699 genes and HLCs/fetal liver 988 genes

Supplementary Table S10: Antibody list.

List of antibodies used for immunofluorescence-based staining of cells.

Supplementary Table S11: Quantitative real-time PCR (qPCR) primer sequences.

List of primer sequences used for quantitative real-time PCR.

Supplemental Methods

Immunofluorescence-based detection of proteins

The cells were fixed, permeabilized and stained for immunofluorescent imaging as described by Matz and Adjaye [2]. The list of primary and secondary antibodies used is provided in Supplementary Table S9. Nuclei were counter-stained with DAPI (100 ng/ml, Vector Laboratories, Burlingame, CA, USA, www.vectorlabs.com). The fluorophores on the secondary antibodies were visualized using a Zeiss, LSM 510 Meta confocal microscope with a connected camera for microscopy model AxioCam ICc3 and the software Axiovision 4.6.

Quantitative Real-Time Polymerase Chain Reaction

The Quantitative Real-Time Polymerase Chain Reaction (qPCR) was performed in 384-well Optical Reaction Plates (Applied BioSystems, Foster City, CA, http://www.appliedbiosystems.com). Reactions were carried out on the ABI PRISM 7900HT Sequence Detection System (Applied BioSystems) as previously described [3]. The amplifications were carried out in triplicates per gene with three wells as negative controls without template. *GAPDH* and *ß-ACTIN* were amplified along with the target genes as endogenous control for normalization. The last heating step was performed with a ramp rate of 2% in order to generate a dissociation curve of the qPCR product. The output data generated by the SDS 2 software (Applied BioSystems) were transferred to MS Excel (Microsoft) for analysis. The differential mRNA expression of each gene was normalized against the *GAPDH* mRNA expression in the respective samples and calculated using the comparative Ct (threshold cycle) method (ΔΔCt method). Commercial bought fetal liver (Clontech, #636540) and PHH (Clontech, #636531) RNA was used. All primers which were used are listed in Supplementary Table S11.

Microarray -Based Gene Expression Analysis

Quality of experiments at the bead-summary level is controlled by calculation of the Pearson correlation coefficient between all experiments vs. each other. These coefficients are condensed in a table marking value ranges of the correlation coefficients in different colors to enable detection of outliers. Additionally, a dendrogram is generated via the *plotSampleRelation* method from the R/Bioconductor lumi package to enable comparison of similarities between experimental samples and outlier finding by obvious mis-alignments in the dendrogram[3-5].

Stage specific genes are determined via comparisons of subsequent differentiation stages: DE vs. E-iPSCs, HE vs. DE, HLCs vs. HE, Fetal liver vs. HLCs and PHH vs. fetal liver. Differentially up-

regulated genes in these comparisons are found with the criteria a) Illumina detection p-value < 0.05 in the test case, b) ratio > 1.3333 and c) limma-p-value < 0.05 and limma-q-value < 0.05. Differentially down-regulated genes in these comparisons are found with the criteria a) Illumina detection p-value < 0.05 in the control case, b) ratio < 0.75 and c) limma-p-value < 0.05 and limma-q-value < 0.05. The differentially expressed genes are subjected to pathway analysis via Consensus Path DB [6].

Cytochromes, ABC transporter genes and several other transporter genes were filtered from the microarray experiments of iPSCs, definite endoderm (DE), hepatic endoderm (HE), hepatocyte-like cells (HLCs), fetal liver and primary human. Heat maps of these clusters were generated via the heatmap2 function from R using Euclidean distance as distance measure.

Genes differentially expressed in HLCs vs. E-iPSCs, fetal liver vs. E-iPSCs and PHH vs. E-iPSCs were compared via a Venn diagram. Differential expression was termed significant if a) limma-p-value < 0.05 and limma-q-value <0.05, b) detection-p-value<0.05 at least in one of both conditions and c) ratio < 0.75 or ratio > 1.33.

In order to detect differences between the three experiments in more detail an analysis of variance (ANOVA) was employed to filter genes followed by pairwise t-tests. Genes were termed significant if a) anova-p-value<0.05 and anova-q-value<0.05, b) t-test p-value<0.05 and t-test q-value<0.05 and c) detection-p-value<0.05 at least in one of both conditions.

K-means clustering and analysis of differentiation-stage-specific clusters

Expression profiles of all genes are analyzed via k-means clustering. In order to achieve a sufficient granularity k=100 clusters are conceived. The *kmeans* clustering algorithm from the R/Bioconductor environment is applied for this task. The result of the clustering analysis is an association table of Illumina probes and genes to the 100 clusters. Plots of mean and standard deviation of all genes' expression over all HLC differentiation phases are condensed for all clusters. Heatmaps of these clusters are generated via the *heatmap2* function from R.

Stage-specific clusters containing genes which are prominently expressed in dedicated stages of the differentiation were extracted from the k-means clustering. Five clusters were selected representing stages iPSC (containing POU5F1), definite endoderm (containing SOX17), hepatic endoderm, hepatocyte-like cells, fetal liver (containing AFP) and primary human hepatocyte (containing ALB). The gene sets from these clusters were subjected to transcription factor analysis via oPOSSUM3 [7] single site analysis using default parameters with the exception of restricting the amount of upstream / downstream sequence to 2000/2000 bases. Networks were drawn via the R package *network* scaling transcription factor nodes proportional to the oPOSSUM z-score and using a threshold of z>3.

microRNA analysis is based upon TargetScan 6.2 predictions [8]. Genes from stage-specific k-means clusters are analysed for over-representation in gene sets regulated by the same microRNA. P-values are calculated via the hypergeometric test. False-Discovery rate is assessed using q-values [9]. P-values and q-values <0.05 are marked in red.

Tissue type prediction

In order to check the tissue type of the HLCs the application of a tissue prediction tool was considered. At the time when this manuscript was written the CellNet [10] tool was not able to test data from the Illumina Human HT12 platform. Therefore it was decided to use the tool KeyGenes [11]. Although KeyGenes was developed for Next-Generation-Sequencing data there it provides the possibility to generate own training sets. A training was set up to fit the Illumina Human platform thus avoiding technical bias introduced by differing microarray platforms. Data sets for the Illumina human platform were downloaded from NCBI GEO for tissues brain (GSE29378) [12], kidney (GSE43974) [13], lung (GSE63459), intestine (GSE48634) , heart (GSE64189) and liver (GSE25744) [14]. These data sets were normalized via quantile normalization from the R/Bioconductor [4] package preprocessCore and transformed to a logarithmic scale (base 2). The training data set was generated from them via the R script provided at the KeyGenes web site (www.keygenes.nl) [12]. The test set was produced by filtering genes from k-means cluster 9 which had a peak at HLC from the quantile normalized log2-transformed Illumina data. HLCs, fetal liver and primary human hepatocytes samples were used for the test set.

Analysis of tissues associated with expressed genes

Association of genes' expression with tissues was retrieved from the PaGenBase database version 1.0 [15]. The collection of datasets for specific genes for Homo sapiens/tissue was used for this analysis thus basing it on a combination of microarray and next-generation sequencing data. Genes exclusively expressed (detection p-value<0.05) in HLC cells but not in DE cells and not in HE cells were mapped to PaGenBase tissues.

Transcription factor analysis

The oPOSSUM database [7] was downloaded in June 2014 and Single Site Analysis was performed on this local instance to detect over-represented conserved transcription factor binding sites in a set of progenitor associated genes. This analysis was parameterized to use species "human", 2000 base pairs upstream and downstream each, use only JASPAR Transcription Factor Binding Site (TFBS) profile matrices which belong to the tax group "vertebrates", a minimum relative TFBS position weight matrix (PWM) score of 0.85 and a minimum information content (specificity) of JASPAR TFBS profile matrices of 8.

The network was drawn based on the results of the oPOSSUM analysis using only transcription factors surpassing a predefined for the Z-Score. The Z-Score shows if in the investigated up- and

downstream regions of the set of genes there are more TFBS than in the background. For Supp. Fig. 3 we used a threshold of 3 while for fig. 6 we used a relatively restrictive threshold of 10 to focus on the core regulatory network. For all transcription factors above this threshold we extracted the conserved binding sites from the oPOSSUM analysis output and connected them with the transcription factor itself as edges in the graph. For the drawing and layout of the graph we employed the R package *network* ((http://www.jstatsoft.org/v24/i02/paper (Accessed February 26, 2015) [16] which was instructed to draw red circles for genes and green circles with sizes corresponding to the Z-scores for the transcription factors.

References

1 Jozefczuk, J., Prigione, A., Chavez, L. & Adjaye, J. (2011). Comparative analysis of human embryonic stem cell and induced pluripotent stem cell-derived hepatocyte-like cells reveals current drawbacks and possible strategies for improved differentiation. *Stem Cells Dev* **20**, 1259-75.

2 Matz, P., and Adjaye, J. Generation of iPSC line epiHUVEC from human umbilical vein endothelial cells. *Stem Cell Research*. **15**, 581-583 (2015).

3 Ihaka, R. G. R. R: A Language for Data Analysis and Graphics. *Journal of Computational and Graphical Statistics*. **3**, 299–314 (1996).

4 Gentleman, R. C., *et al.* Bioconductor: open software development for computational biology and bioinformatics. *Genome Biol*. **5**, R80 (2004).

5 Du, P., Kibbe, W. A. & Lin, S. M. lumi: a pipeline for processing Illumina microarray. *Bioinformatics*. **24**, 1547-1548 (2008).

6 Kamburov, A., *et al.* ConsensusPathDB: toward a more complete picture of cell biology. *Nucleic Acids Res*. **39**, D712--D717 (2011).

7 Kwon, A. T., Arenillas, D. J., Worsley, Hunt R. & Wasserman, W. W. oPOSSUM-3: advanced analysis of regulatory motif over-representation across genes or ChIP-Seq datasets. *G3 (Bethesda)*. **2**, 987-1002 (2012).

8 Lewis ,B. P., Burge, C. B. & Bartel, D. P. Conserved seed pairing, often flanked by adenosines, indicates that thousands of human genes are microRNA targets. *Cell*. **120**, 15-20 (2005).

9 Storey, J. D. A Direct Approach to False Discovery Rates. *Journal of the Royal Statistical Society: Series B (Statistical Methodology)*. **64**, 479–98 (2002).

10 Cahan, P. *et al.* CellNet: network biology applied to stem cell engineering. *Cell* **158**, 903–915 (2014).

11 Roost, M. S. *et al.* KeyGenes, a Tool to Probe Tissue Differentiation Using a Human Fetal Transcriptional Atlas. *Stem Cell Rep.* **4**, 1112–1124 (2015).

12 Miller, J. A., Woltjer, R. L., Goodenbour, J. M., Horvath, S. & Geschwind, D. H. Genes and pathways underlying regional and cell type changes in Alzheimer's disease. *Genome Med.* **5**, 48 (2013).

13 Damman, J. *et al.* Hypoxia and Complement-and-Coagulation Pathways in the Deceased Organ Donor as the Major Target for Intervention to Improve Renal Allograft Outcome. *Transplantation* **99**, 1293–1300 (2015).

14 Jozefczuk, J., Prigione, A., Chavez, L. & Adjaye, J. Comparative Analysis of Human Embryonic Stem Cell and Induced Pluripotent Stem Cell-Derived Hepatocyte-Like Cells Reveals Current Drawbacks and Possible Strategies for Improved Differentiation. *Stem Cells Dev.* **20**, 1259–1275 (2010).

15 Pan, J. B., *et al.* PaGenBase: a pattern gene database for the global and dynamic understanding of gene function. *PLoS One*. **8**, e80747 (2013).

16 Butts, C. network: APackage for Managing Relational Data in R. *J Stat Softw*. 2008.

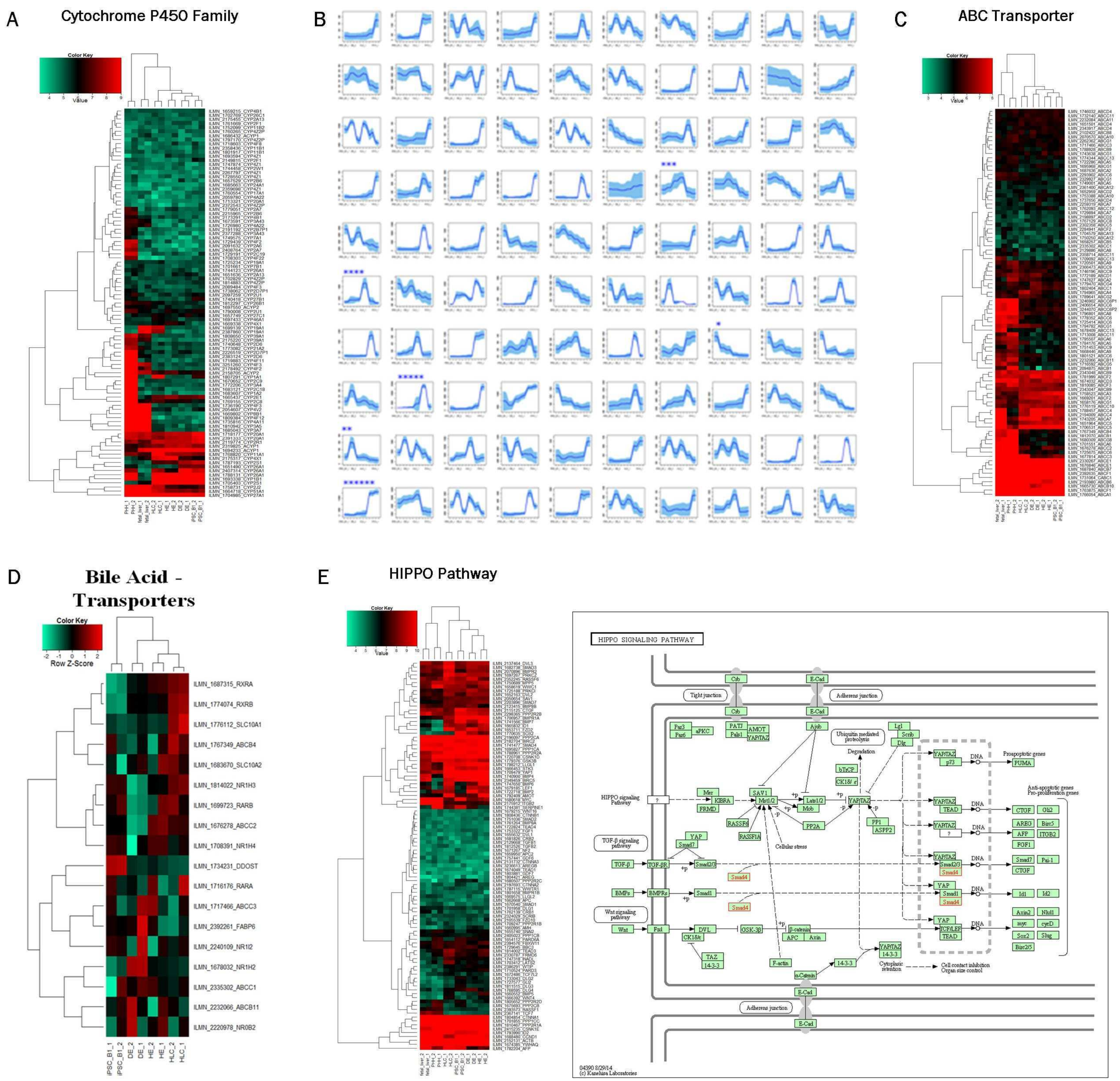

A
Cytochrome P450 Family
Color Key
Value
B
C
ABC Transporter
Color Key
Value
D
Bile Acid -
Transporters
Color Key
Row Z-Score
ILMN_1687315_RXRA
ILMN_1774074_RXRB
ILMN_1776112_SLC10A1
ILMN_1767349_ABCB4
ILMN_1683670_SLC10A2
ILMN_1814022_NR1H3
ILMN_1699723_RARB
ILMN_1676278_ABCC2
ILMN_1708391_NR1H4
ILMN_1734231_DDOST
ILMN_1716176_RARA
ILMN_1717466_ABCC3
ILMN_2392261_FABP6
ILMN_2240109_NR1I2
ILMN_1678032_NR1H2
ILMN_2335302_ABCC1
ILMN_2232066_ABCB11
ILMN_2220978_NR0B2
iPSC_B1_1
iPSC_B1_2
DE_2
DE_1
HE_2
HE_1
HLC_2
HLC_1
E
HIPPO Pathway
Color Key
Value
HIPPO SIGNALING PATHWAY
04390 8/29/14
(c) Kanehisa Laboratories

A

Category	Term	Count	%	PValue
KEGG_PATHWAY	hsa00830:Retinol metabolism	3	8,11	0,006825
KEGG_PATHWAY	hsa00980:Metabolism of xenobiotics by cytochrome P450	3	8,11	0,008375
KEGG_PATHWAY	hsa00982:Drug metabolism	3	8,11	0,008925
KEGG_PATHWAY	hsa00071:Fatty acid metabolism	2	5,41	0,09051
KEGG_PATHWAY	hsa00983:Drug metabolism	2	5,41	0,096986
KEGG_PATHWAY	hsa00350:Tyrosine metabolism	2	5,41	0,099135

B

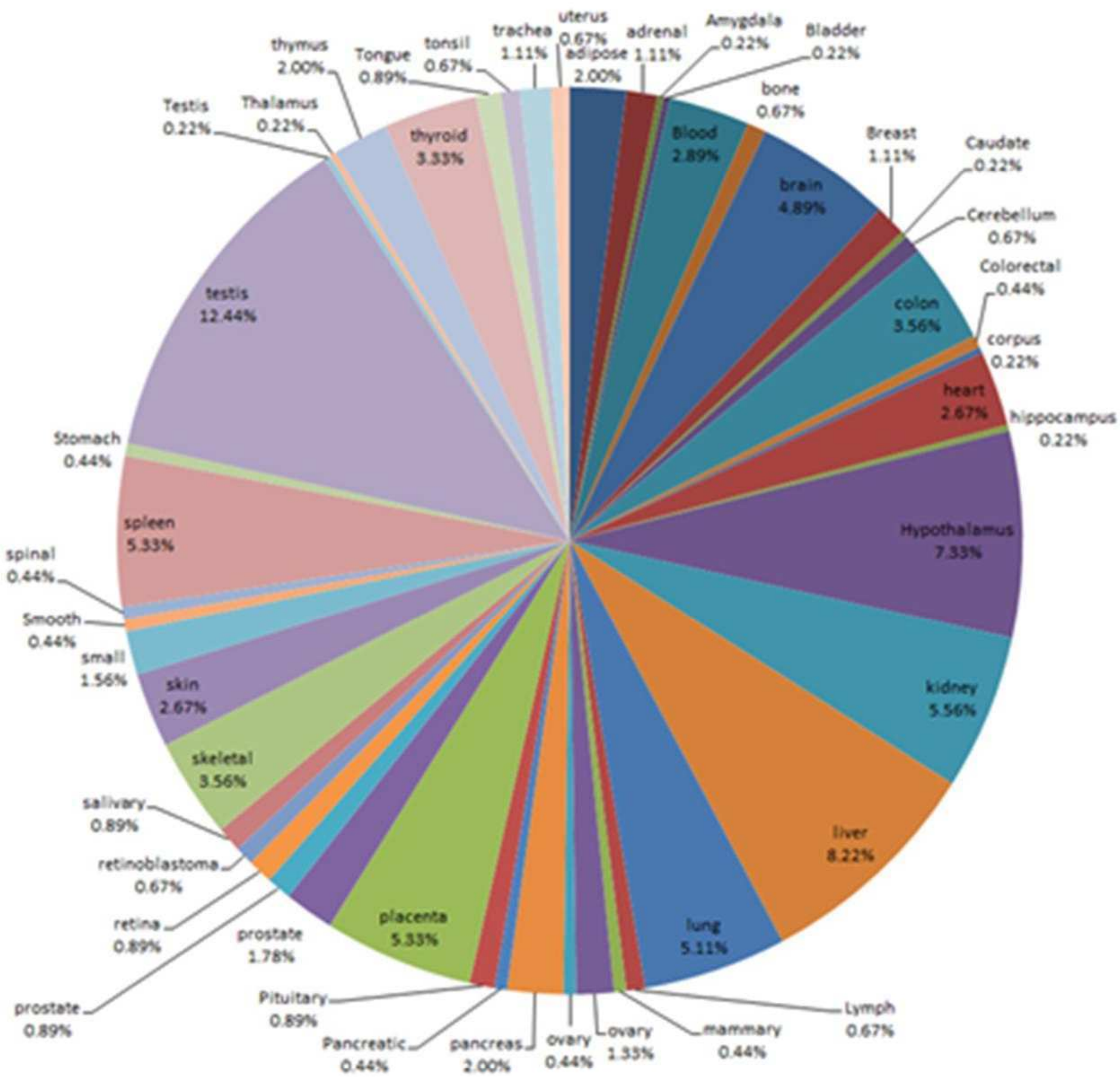

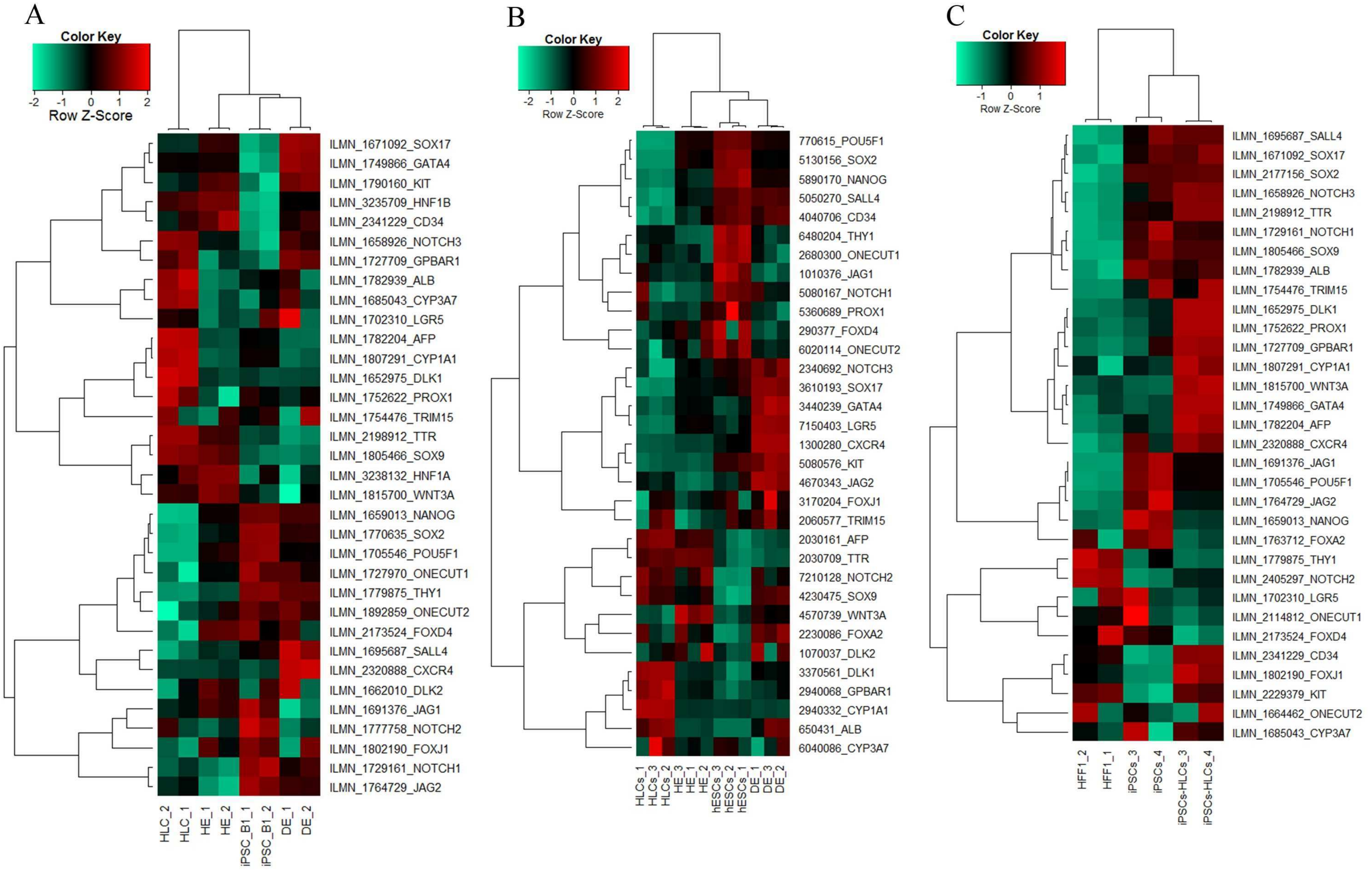
A
Color Key
-2 -1 0 1 2
Row Z-Score
ILMN_1671092_SOX17
ILMN_1749866_GATA4
ILMN_1790160_KIT
ILMN_3235709_HNF1B
ILMN_2341229_CD34
ILMN_1658926_NOTCH3
ILMN_1727709_GPBAR1
ILMN_1782939_ALB
ILMN_1685043_CYP3A7
ILMN_1702310_LGR5
ILMN_1782204_AFP
ILMN_1807291_CYP1A1
ILMN_1652975_DLK1
ILMN_1752622_PROX1
ILMN_1754476_TRIM15
ILMN_2198912_TTR
ILMN_1805466_SOX9
ILMN_3238132_HNF1A
ILMN_1815700_WNT3A
ILMN_1659013_NANOG
ILMN_1770635_SOX2
ILMN_1705546_POU5F1
ILMN_1727970_ONECUT1
ILMN_1779875_THY1
ILMN_1892859_ONECUT2
ILMN_2173524_FOXD4
ILMN_1695687_SALL4
ILMN_2320888_CXCR4
ILMN_1662010_DLK2
ILMN_1691376_JAG1
ILMN_1777758_NOTCH2
ILMN_1802190_FOXJ1
ILMN_1729161_NOTCH1
ILMN_1764729_JAG2
HLC_2
HLC_1
HE_1
HE_2
iPSC_B1_1
iPSC_B1_2
DE_1
DE_2
B
Color Key
-2 -1 0 1 2
Row Z-Score
770615_POU5F1
5130156_SOX2
5890170_NANOG
5050270_SALL4
4040706_CD34
6480204_THY1
2680300_ONECUT1
1010376_JAG1
5080167_NOTCH1
5360689_PROX1
290377_FOXD4
6020114_ONECUT2
2340692_NOTCH3
3610193_SOX17
3440239_GATA4
7150403_LGR5
1300280_CXCR4
5080576_KIT
4670343_JAG2
3170204_FOXJ1
2060577_TRIM15
2030161_AFP
2030709_TTR
7210128_NOTCH2
4230475_SOX9
4570739_WNT3A
2230086_FOXA2
1070037_DLK2
3370561_DLK1
2940068_GPBAR1
2940332_CYP1A1
650431_ALB
6040086_CYP3A7
HLCs_1
HLCs_3
HLCs_2
HE_3
HE_1
HE_2
hESCs_3
hESCs_2
hESCs_1
DE_1
DE_3
DE_2
C
Color Key
-1 0 1
Row Z-Score
ILMN_1695687_SALL4
ILMN_1671092_SOX17
ILMN_2177156_SOX2
ILMN_1658926_NOTCH3
ILMN_2198912_TTR
ILMN_1729161_NOTCH1
ILMN_1805466_SOX9
ILMN_1782939_ALB
ILMN_1754476_TRIM15
ILMN_1652975_DLK1
ILMN_1752622_PROX1
ILMN_1727709_GPBAR1
ILMN_1807291_CYP1A1
ILMN_1815700_WNT3A
ILMN_1749866_GATA4
ILMN_1782204_AFP
ILMN_2320888_CXCR4
ILMN_1691376_JAG1
ILMN_1705546_POU5F1
ILMN_1764729_JAG2
ILMN_1659013_NANOG
ILMN_1763712_FOXA2
ILMN_1779875_THY1
ILMN_2405297_NOTCH2
ILMN_1702310_LGR5
ILMN_2114812_ONECUT1
ILMN_2173524_FOXD4
ILMN_2341229_CD34
ILMN_1802190_FOXJ1
ILMN_2229379_KIT
ILMN_1664462_ONECUT2
ILMN_1685043_CYP3A7
HFF1_2
HFF1_1
iPSCs_3
iPSCs_4
iPSCs-HLCs_3
iPSCs-HLCs_4

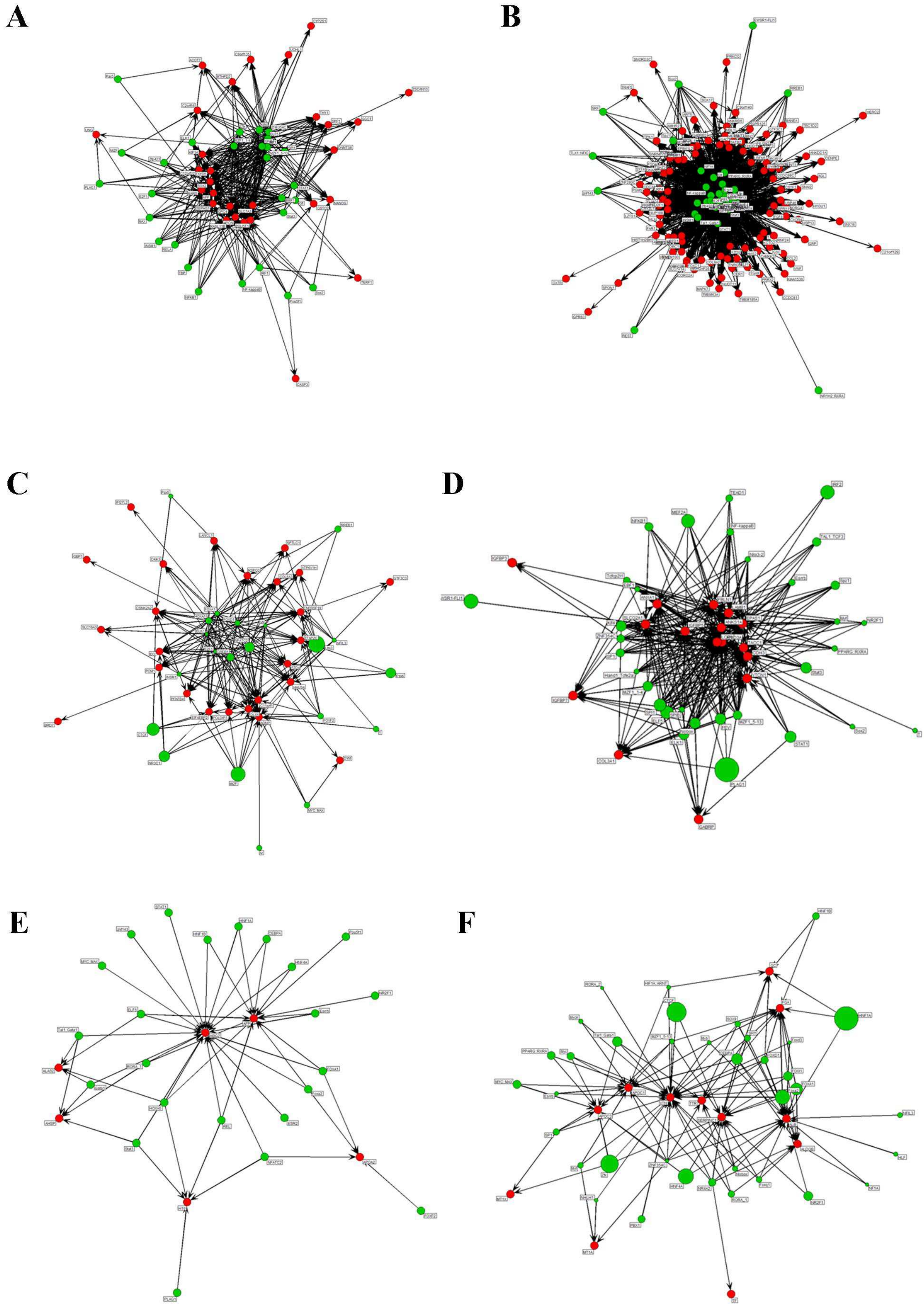
A
B
C
D
E
F

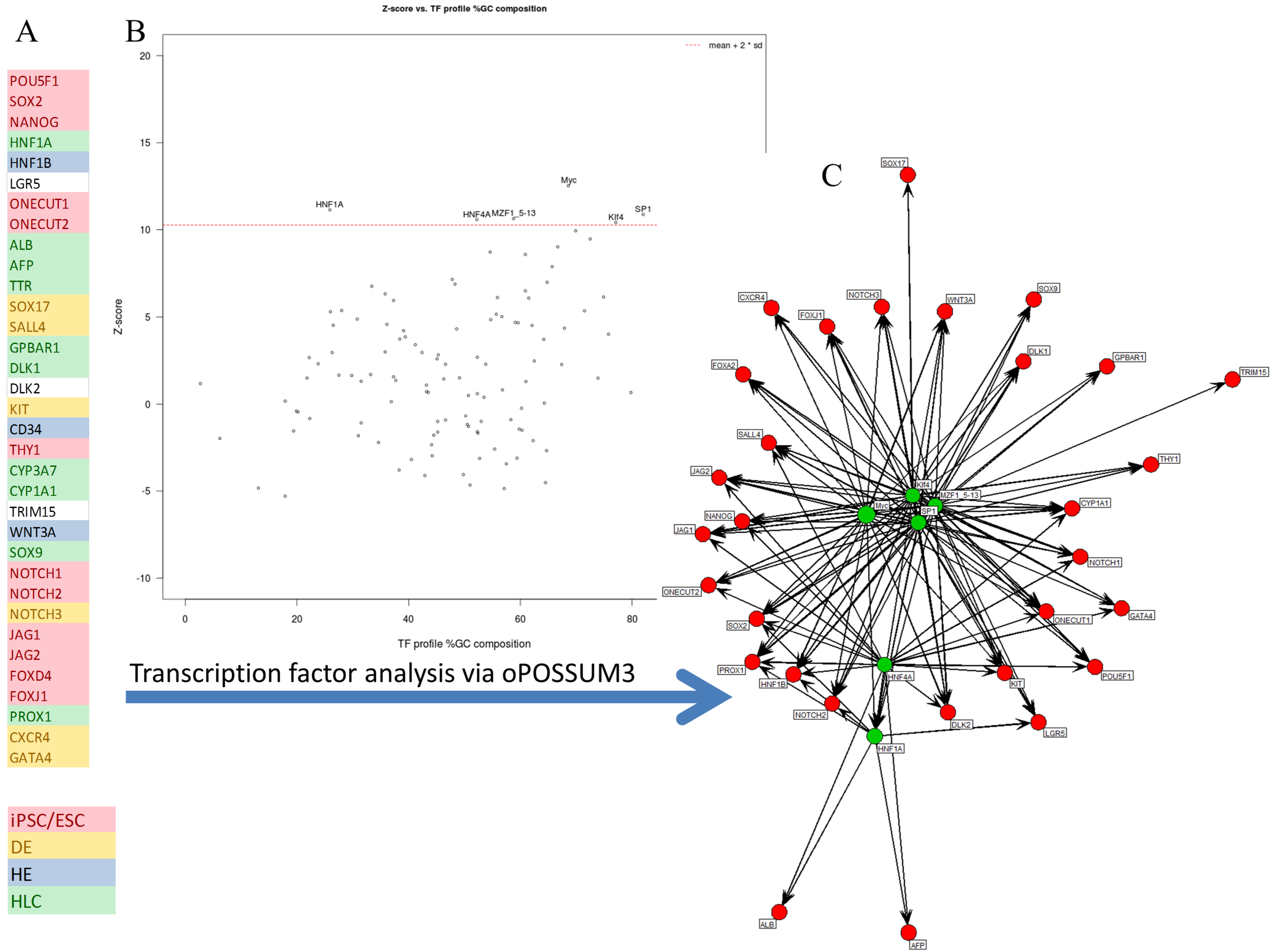
A
B
C
POU5F1
SOX2
NANOG
HNF1A
HNF1B
LGR5
ONECUT1
ONECUT2
ALB
AFP
TTR
SOX17
SALL4
GPBAR1
DLK1
DLK2
KIT
CD34
THY1
CYP3A7
CYP1A1
TRIM15
WNT3A
SOX9
NOTCH1
NOTCH2
NOTCH3
JAG1
JAG2
FOXD4
FOXJ1
PROX1
CXCR4
GATA4
iPSC/ESC
DE
HE
HLC
Z-score vs. TF profile %GC composition
mean + 2 * sd
Z-score
TF profile %GC composition
HNF1A
HNF4A
MZF1_5-13
Myc
Klf4
SP1
Transcription factor analysis via oPOSSUM3

Supplementary Table S1: KeyGenes corresponding to Figure 4E.

A	HLC_1	HLC_2	fetal_liver_1	fetal_liver_2	PHH_1	PHH_2
brain	0,00872249	0,00356035	0,00095596	0,00077447	0,00128284	0,00129498
heart	0,00202789	0,00126442	0,00045451	0,00091234	0,00203436	0,00299791
intestine	0,00058457	0,00194039	0,00044729	0,00040809	0,00748768	0,00180078
kidney	0,05411781	0,09250339	0,00736333	0,00554801	0,003114	0,00645026
liver	0,93235915	0,89657184	0,98934908	0,99221622	0,98571919	0,98730311
lung	0,00218808	0,00415962	0,00142983	0,00014086	0,00036192	0,00015297

B				
brain	ENSG0000010	ENSG0000014	ENSG0000016	ENSG00000166963
heart	ENSG00000118194			
intestine	ENSG0000014	ENSG0000018	ENSG00000260027	
kidney	ENSG0000006	ENSG0000013	ENSG0000018	ENSG00000260027
liver	ENSG0000013	ENSG0000014	ENSG00000155066	
lung	ENSG0000006	ENSG00000173391		

Supplementary Table 2: Gene lists of venn diagram Figure 5A.				
hlc_expr	fetal_liver _expr	hlc_expr AND fetal_liver_expr	phh_expr	hlc_expr AND phh_expr
A2ML1	ABCC13	ABCA4	A1BG	AASS
A4GALT	ABI3	ABCC10	ABCA10	ABCA2
A4GNT	ABI3BP	ABCC4	ABCG4	ABHD11
AADACL4	ABLIM2	ABCG2	ABHD1	ABHD7
ABCA11	ACAP1	ACRC	ACBD5	ABL2
ABHD12B	ACPT	ACSL4	ACSM1	ACBD4
ABHD9	ACRBP	ACSS1	ADAMTS15	ACER3
ACBD7	ACSBG1	ACTA1	ADCYAP1R1	ADCY1
ACCN3	ACSL6	ACTG2	ADH1B	AGR2
ACPP	ADAM12	ADAMTS7	AFAR3	AIM1
ACRV1	ADAMTS5	ADAMTS9	AKR7L	AIM1L
ACTC1	ADD2	ADAMTSL1	ALOXE3	AKR1B10
ADAM2	ADRA2C	ADAT2	ALS2CL	AKR1B15
ADAM8	ALOX12	ADCY3	ALS2CR12	ALK
ADAMTS18	AMN	ADCY4	ALX3	ALPI
ADAMTS19	AMPH	ADORA2A	ANKRD29	ALPK2
ADAMTS2	ANAPC7	ADORA2B	AOX1	AMAC1L2
ADAMTS20	ANK1	ADPRH	APLN	AMY2B
ADAMTS3	ANKAR	AEBP1	APOL1	ANKRD1
ADAMTS6	ANKLE1	AFAP1	AQP7	ANKRD20B
AFF2	ANKRD53	AFAP1L2	AQP9	ANXA8
AHDC1	AOAH	AFF3	ARID5A	ANXA8L2
AHNAK2	APCDD1	AGPAT4	ARL14	ARAP2
AKAP2	APOBEC2	AHCYL2	ARP11	ARFGEF2
AKT3	ARFRP1	AHI1	ASIP	ARHGEF16
ALDH1A3	ARHGAP5	AHSP	ASPHD1	ARL4C
ALDH1L2	ARHGDIG	AIM2	ATOH8	ARMC9
ALDH3B2	ARHGEF9	AKAP10	ATP6V0D2	ARRDC1
ALG10	ARL9	ALG10B	BAI1	ASPH
ALKBH8	ARMC2	ALMS1	BDH2	ATHL1
ALMS1P	ARMC4	ALOX15	BOLA2B	ATP13A3
ALPPL2	ARTN	AMHR2	C10orf108	ATP6V0A4
ALS2CR16	ASCL2	ANAPC2	C10orf21	ATP6V1E2
AMBRA1	ATOH7	ANGPT1	C11orf91	B3GNT3
AMH	ATP1B2	ANGPTL2	C12orf34	B4GALNT4
AMN1	AZU1	ANK3	C12orf39	BACH1
ANGPT2	B3GALT2	ANKRD32	C12orf56	BAK1
ANKDD1A	BAI3	ANKRD41	C12orf8	BEX5
ANKRD34A	BCL2L14	ANKRD52	C14orf11	BIK
ANKRD36	BEST1	ANLN	C14orf121	BLOC1S2
ANKS1B	BPI	ANO2	C14orf126	BTN2A2
ANKS3	BSPH1	ANTXR1	C14orf21	C11orf36
ANO9	BST1	AP1GBP1	C17orf67	C12orf49
ANXA8L1	BZRPL1	AP1M2	C19orf36	C12orf5
AP3B2	C10orf105	AP3M2	C1orf200	C13orf31
APCDD1L	C11orf21	AP4M1	C21orf63	C14orf118
APLF	C14orf162	APEG1	C21orf71	C14orf181
APLP1	C15orf34	APLNR	C3orf41	C14orf78
AQP10	C16orf30	APOBEC3D	C4B	C15orf48
ARHGAP27	C16orf38	APOLD1	C5orf46	C16orf67

ARHGAP29	C17orf76	AQP1	C6orf122	C17orf108
ARHGAP6	C17orf87	ARHGAP11B	C6orf142	C19orf71
ARL13B	C18orf1	ARHGAP15	C6orf222	C22orf33
ARNT2	C18orf51	ARHGAP18	C6orf27	C2orf15
ARSK	C19orf59	ARHGAP20	C7orf65	C2orf21
ASAM	C19orf67	ARHGAP22	C8orf30B	C3orf33
ASPHD2	C1orf101	ARHGAP25	C8orf46	C3orf52
ASRGL1	C1orf175	ARHGAP28	C8ORFK29	C4A
ATP10B	C1orf186	ARHGAP8	C9	C6orf1
ATP12A	C1orf38	ARHGEF17	CADM3	C6orf141
ATP2C2	C1orf92	ARHGEF3	CALCA	C6orf163
ATP6V1B1	C1QTNF4	ARL17P1	CAMK2B	C6orf204
ATP6V1C2	C20orf132	ARMCX1	CCDC103	C7orf53
ATP6V1G2	C20orf175	ARMCX2	CCDC88B	CAMK2D
ATP8B1	C21orf67	ARMCX4	CCK	CAMTA2
ATRX	C21orf69	ART5	CCL13	CBLC
AURKAPS1	C2CD4C	ASAP1IT1	CCR2	CBX8
B3GALNT2	C2orf3	ASCL5	CD200	CCDC126
B3GALTL	C2orf32	ATG2B	CD300E	CCDC17
B3GNT5	C2orf40	ATP2A3	CD40	CCNO
BAALC	C2orf88	ATP6AP1L	CDC27	CDCP1
BAHCC1	C2orf89	ATP7A	CDC37L1	CDK8
BAI2	C3AR1	ATXN2L	CDH15	CDKN2A
BAPX1	C3orf22	AURKA	CES4	CDKN2C
BATF3	C3orf32	AURKB	CH25H	CEP170
BCAR4	C3orf71	B3GALNT1	CHAD	CFHR3
BCL11B	C4orf37	B3GALT4	CHADL	CHST9
BCMO1	C5AR1	B3GNT8	CHRDL2	CIDEA
BEND4	C5orf20	B3GNTL1	CHST4	CLCF1
BEND6	C6orf184	B4GALT6	CLIP4	CLTCL1
BICD1	C7	BACH2	CLU	CNNM4
BMP5	C8orf77	BAGE5	CLUL1	CNTNAP2
BNC1	C8orf79	BARD1	CNDP1	COL11A2
BNC2	C9orf84	BBS12	COPZ2	CRCT1
BOC	CA1	BCAM	COX16	CSDC2
BRWD3	CACNA1I	BCAT1	CPLX1	CST6
BVES	CALCRL	BCL11A	CSAG3A	CXorf21
C10orf39	CAMP	BCL2	CSAG3B	CYP1A1
C10orf41	CAPN11	BDP1	CSF1	CYP1B1
C10orf73	CARD9	BEND5	CTRB1	CYP26A1
C10orf82	CAV3	BEX4	CXCL13	CYP2D7P1
C11orf47	CBFA2T3	BIRC5	CXCL2	CYP2E1
C11orf70	CBLN4	BLM	CXCL6	DAB2IP
C12orf64	CCDC111	BMP6	CYP1A2	DALRD3
C14orf125	CCDC26	BMP7	CYP27B1	DCAF4L1
C14orf176	CCDC27	BMPER	CYP2A6	DDIT4L
C14orf37	CCDC52	BNIPL	CYP2A7	DDX3Y
C15orf33	CCDC81	BPTF	CYP2B6	DHH
C15orf59	CCDC88A	BRAF	CYP2B7P1	DHRS2
C16orf54	CCL21	BRSK1	CYP2C18	DIRAS3
C16orf55	CCL23	BRUNOL6	CYP2C19	DKFZP564O0523
C16orf74	CCL8	BSN	CYP2C9	DKFZp667M2411
C16orf88	CCR1	BSPRY	CYP3A4	DMRTA1
C17orf47	CCR10	BTK	CYP4F11	DNAJC2
C17orf69	CCR9	BTRC	CYP4F22	DNAJC25-GNG10
C17orf78	CCRL2	BUB1	CYP7A1	DNTTIP2

C17orf82	CD163L1	BUB1B	CYSLTR2	DOCK9
C18orf45	CD180	C10orf128	DAO	DSEL
C19orf21	CD19	C10orf33	DAPK2	DUSP27
C19orf39	CD1A	C10orf51	DDTL	DZIP1L
C19orf69	CD209	C10orf78	DDX20	EDN1
C1orf102	CD300A	C10orf88	DDX25	EEA1
C1orf105	CD300C	C11orf61	DERPC	EEF1A2
C1orf133	CD33	C11orf82	DGCR5	EFCAB7
C1orf161	CD36	C11orf84	DIS3	EFHC1
C1orf170	CD37	C12orf48	DKFZp564N2472	EGLN3
C1orf187	CD38	C12orf60	DKFZp686K1684	EGR2
C1orf189	CD4	C12orf68	DNAJC16	EIF2C4
C1orf190	CD48	C13orf18	DOCK5	EIF5A2
C1orf213	CD53	C13orf3	DOK7	ELK4
C1orf215	CD5L	C13orf34	DSG1	ENAH
C1orf229	CDADC1	C14orf145	DUXA	EPHB6
C1orf61	CEACAM8	C14orf19	DYNC1LI1	ESM1
C1orf64	CETP	C15orf20	DYSFIP1	EXOC8
C1orf81	CFHR4	C15orf21	ECEL1	EYA2
C1orf88	CFP	C15orf27	ECT2L	F2RL1
C1orf96	CHAC2	C15orf28	EDAR	F8A3
C20orf12	CHRNA10	C15orf42	EIF3CL	FAM114A1
C20orf151	CHRNB4	C16orf62	ENTPD8	FAM45B
C20orf75	CISH	C16orf79	EPHA3	FAM46B
C21orf129	CLC	C17orf41	EPHA6	FAM59A
C21orf49	CLDN2	C17orf44	EREG	FAM76A
C21orf81	CLEC10A	C17orf65	ERO1LB	FBLIM1
C2orf54	CLEC12A	C17orf75	ESR1	FBXO16
C2orf67	CLEC1B	C17orf80	F2RL3	FEM1B
C3orf15	CLEC3B	C17orf98	FAM139A	FER1L5
C3orf18	CLEC4D	C18orf2	FAM150B	FGD6
C3orf67	CLEC4G	C18orf26	FAM57B	FGF2
C3orf72	CLEC4M	C18orf54	FAM71E2	FLJ10781
C4orf10	CLIC2	C19orf33	FAM80A	FLJ10996
C4orf16	CMAH	C19orf38	FANCA	FLJ26850
C4orf31	CMPK2	C1orf106	FAS	FLJ35024
C4orf39	CMTM1	C1orf116	FBXO25	FLJ41200
C4orf43	CMTM2	C1orf135	FCAMR	FLJ41484
C4orf49	CMTM5	C1orf152	FGF19	FOSL1
C5orf27	COL14A1	C1orf156	FGF21	FOXP4
C6orf100	COL9A1	C1orf165	FGF6	GABRB3
C6orf132	COLEC10	C1orf21	FGF9	GALR2
C6orf134	CORO1A	C1orf24	FIS	GAN
C6orf148	CPA3	C1orf26	FLJ10661	GARNL3
C6orf168	CR1L	C1orf59	FLJ20397	GATA6
C6orf199	CRB1	C1orf89	FLJ21687	GBAP
C6orf221	CSH1	C1orf95	FLJ35785	GGTL3
C6orf25	CSH2	C1QTNF1	FLJ38379	GHRHR
C6orf52	CSPG4	C1QTNF5	FLJ40330	GMIP
C6orf54	CST7	C20orf117	FLJ42289	GPC1
C6orf81	CTSG	C20orf160	FLJ42709	GPR153
C8orf16	CUGBP2	C20orf194	FLJ44790	GPR64
C9orf126	CX3CR1	C20orf196	FLJ45422	GPRC5A
C9orf135	CXADRP3	C20orf94	FLJ45966	GPRC5B
C9orf164	CXCL17	C21orf122	FOXD2	GRAMD1C
C9orf58	CXCL3	C21orf128	FRY	GREM1

C9orf61	CXCR4	C21orf56	FSIP1	GREM2
C9orf93	CXorf18	C2orf37	FSTL4	GRHL1
CA11	CXorf59	C2orf63	FUNDC2	GRIP2
CA3	CYBB	C3orf62	FUT5	HAPLN4
CABYR	CYLC1	C3orf70	G0S2	HDAC9
CACNA1C	CYSLTR1	C4orf18	GAGE5	HES2
CACNA2D2	CYTH4	C4orf29	GBP3	HHAT
CACNA2D3	CYTL1	C4orf38	GBP7	HIST1H3D
CACNG4	DARC	C5orf23	GCK	HIST1H3E
CADM2	DAZ2	C5orf34	GDA	HIT-40
CALCB	DC36	C5orf39	GEM	HIVEP1
CALCR	DCDC5	C5orf42	GEMIN7	HSPB8
CAPN14	DCX	C5orf53	GFRA1	ICOSLG
CART1	DEFA1	C5orf54	GJB3	IER5L
CASQ2	DEFA1B	C5orf62	GLOD5	IFIT3
CCDC105	DEFA3	C6orf117	GLT1D1	IGF1
CCDC113	DEFA4	C6orf124	GNG12	IL1RL1
CCDC147	DERL3	C6orf167	GPD1	IL20RB
CCDC149	DLEC1	C6orf182	GPR141	INPP4A
CCDC153	DNAJC11	C6orf208	GPR88	IPPK
CCDC33	DNAJC28	C6orf26	GRIA3	IQSEC2
CCDC57	DNASE1L3	C6orf59	GSDM1	IRF6
CCDC64B	DNM3	C7orf13	GSTT2B	ISM1
CCDC9	DNTT	C8orf44	GUCA2B	KATNAL2
CCDC96	DOC2A	C8orf51	GZF1	KCNF1
CCKBR	DOK2	C8orf58	HCG18	KCNN2
CCNA1	DPEP2	C9orf100	HEPACAM	KCTD11
CDGAP	DPEP3	C9orf140	HIST1H3A	KCTD17
CDH10	DPF1	C9orf167	HNF4A	KDM6B
CDH13	DPF3	C9orf62	HRCT1	KIAA0284
CDH17	DPT	C9orf66	HSD11B1	KIAA0672
CDH26	DSCR5	CA14	HSD17B13	KIAA0895L
CDH3	DUSP21	CA8	HSF4	KIAA1383
CDH8	DUSP26	CACNB2	HTR2B	KISS1R
CDKAL1	E2F8	CACNB3	IBSP	KLC4
CDS1	ECE1	CACNG6	IGSF9B	KLF10
CDX1	EEFSEC	CAMK1G	IL12A	KLF5
CDX2	EFCBP1	CAMTA1	IL15	KRT18P19
CDY1B	ELANE	CAPN13	IL15RA	KRT18P26
CECR6	ELN	CASC5	IL1RL2	KRT18P30
CEMP1	ELTD1	CC2D2A	IL23A	KRT18P34
CEND1	EMCN	CCDC121	IL6ST	KRT18P42
CERCAM	EMILIN1	CCDC136	JMJD7	KRT7
CFTR	EPB42	CCDC137	KAAG1	KRT80
CGB	ESCO2	CCDC138	KCND3	LAMB3
CGB7	ESRP2	CCDC146	KCNIP3	LAMC2
CHAC1	F13A1	CCDC15	KIAA0467	LARP6
CHGB	FABP3	CCDC150	KIAA0738	LATS2
CHRM3	FAIM3	CCDC151	KIR2DS3	LDHC
CHRNA3	FAM101B	CCDC152	KLHL15	LMTK2
CIZ1	FAM109B	CCDC3	KLHL25	LOC100128202
CKMT1A	FAM120C	CCDC35	KPNA7	LOC100128269
CKMT1B	FAM124B	CCDC41	LGALS4	LOC100128300
CLCNKA	FAM129C	CCDC66	LGALS9B	LOC100128695
CLDN18	FAM178B	CCDC74B	LGI1	LOC100128822
CLDN6	FAM22A	CCDC8	LOC100127988	LOC100129122

CLEC18C	FAM26F	CCDC99	LOC100128295	LOC100129138
CLEC1A	FAM65C	CCNA2	LOC100128340	LOC100129147
CLEC2L	FAM99A	CCNB1	LOC100128781	LOC100129720
CLIC6	FAM9A	CCNB2	LOC100130079	LOC100130133
CLSTN2	FAT4	CCNJ	LOC100130311	LOC100130383
CNIH2	FBXL17	CCNJL	LOC100130420	LOC100130542
CNR1	FBXO43	CCR7	LOC100130426	LOC100130808
CNTN1	FCER1A	CD247	LOC100130575	LOC100130906
CNTN4	FCN1	CD72	LOC100130812	LOC100131017
COL11A1	FCN2	CDC2	LOC100131071	LOC100131076
COL13A1	FCRL2	CDC20	LOC100131091	LOC100131176
COL15A1	FCRLA	CDC42EP5	LOC100131277	LOC100131447
COL22A1	FDPSL2A	CDC45L	LOC100131390	LOC100131460
COL23A1	FLJ11827	CDC7	LOC100131683	LOC100131727
COL4A6	FLJ23834	CDCA1	LOC100131774	LOC100131895
COL9A2	FLJ25404	CDCA2	LOC100132234	LOC100131972
COL9A3	FLJ30430	CDCA3	LOC100132288	LOC100132205
CORO6	FLJ32255	CDCA5	LOC100132506	LOC100132354
COX11	FLJ36032	CDCA7	LOC100132552	LOC100132494
COX18	FLJ36848	CDCA8	LOC100132655	LOC100132910
CPA4	FLJ39639	CDH11	LOC100132738	LOC100133075
CPAMD8	FLJ41170	CDH5	LOC100132888	LOC100133211
CPLX2	FLJ41603	CDH6	LOC100132913	LOC100133435
CPNE2	FLJ41733	CDK3	LOC100132967	LOC100133438
CPSF6	FLJ44290	CDKL2	LOC100133033	LOC100133572
CRABP1	FLJ44606	CDKN3	LOC100133050	LOC100133866
CRH	FMO2	CDT1	LOC100133056	LOC100134407
CRIM1	FNDC1	CEACAM21	LOC100133077	LOC100134424
CRISPLD1	FOLR2	CEACAM6	LOC100133182	LOC100134794
CRLF1	FOLR3	CECR4	LOC100133242	LOC100134815
CROCC	FOXD4	CEL	LOC100133275	LOC152586
CROCCL1	FOXD4L1	CENPA	LOC100133463	LOC154860
CROCCL2	FOXJ1	CENPE	LOC100133479	LOC168474
CRSP6	FOXS1	CENPF	LOC100133568	LOC283922
CRTC1	FPR1	CENPH	LOC100133591	LOC284371
CRYBA1	FPR3	CENPI	LOC100133719	LOC387683
CRYBA2	FXN	CENPK	LOC100133724	LOC388327
CSGALNACT1	FZD9	CENPM	LOC100133982	LOC388514
CSN1S1	GAB3	CENPO	LOC100134081	LOC388630
CSNK1A1L	GALNT14	CENPP	LOC100134210	LOC388681
CST1	GALNT5	CENPQ	LOC100134226	LOC388955
CST4	GALNT9	CEP110	LOC100134363	LOC389641
CTAGE5	GAPT	CEP135	LOC100134712	LOC400558
CTTNBP2NL	GATA1	CEP152	LOC121792	LOC400578
CUBN	Gcom1	CEP250	LOC123876	LOC400836
CUX1	GDF1	CEP290	LOC124220	LOC401074
CXCR6	GFI1B	CEP55	LOC139735	LOC401433
CXorf23	GFRA3	CEP68	LOC143941	LOC401533
CXXC4	GGT5	CFC1B	LOC144383	LOC401588
CXXC6	GH1	CGA	LOC145837	LOC440160
CYP46A1	GH2	CGB1	LOC150223	LOC440905
DAD1L	GIMAP1	CGB5	LOC165186	LOC441151
DBC1	GIMAP4	CGB8	LOC197350	LOC441442
DCLK2	GIMAP5	CHAF1A	LOC201175	LOC442249
DDR2	GIMAP6	CHAF1B	LOC283155	LOC554223
DEFA6	GIMAP7	CHD3	LOC283711	LOC641983

DEFB116	GIMAP8	CHEK2	LOC285016	LOC642559
DENND3	GJA4	CHN1	LOC285141	LOC642648
DFNB31	GKN2	CHRNE	LOC286297	LOC642732
DHX57	GNA15	CHST10	LOC338756	LOC642769
DIRAS2	GNAL	CHST2	LOC342979	LOC643159
DKFZP434L187	GP9	CHSY3	LOC344382	LOC644094
DKFZp686J0529	GPR101	CHTF18	LOC347487	LOC644144
DKKL1	GPR182	CIB2	LOC348021	LOC644150
DLG2	GPR34	CILP	LOC387723	LOC644629
DLL3	GPR4	CKAP2L	LOC388503	LOC644632
DLX1	GPR44	CKMT2	LOC389053	LOC644733
DLX5	GPR65	CLASP2	LOC389072	LOC644814
DMPK	GPRASP1	CLCN2	LOC389791	LOC644884
DMRT1	GRAP2	CLDN11	LOC391359	LOC645304
DMRT2	GRASP	CLDN19	LOC391817	LOC645411
DMXL2	GSTM5	CLDN20	LOC392145	LOC645434
DNAH14	GTSF1	CLEC11A	LOC392197	LOC645520
DNAH2	GUCY2E	CLEC2B	LOC399940	LOC645558
DNAJC18	GYPA	CLEC4A	LOC400707	LOC645659
DNHL1	GYPB	CLIC3	LOC400759	LOC645676
DOK5	GYPE	CLIP3	LOC401233	LOC645863
DOK6	GZMA	CLSTN3	LOC401317	LOC646038
DPEP1	GZMK	CNFN	LOC401398	LOC646348
DPP10	HBA1	CNGB1	LOC401934	LOC646585
DPPA2	HBB	CNRIP1	LOC402571	LOC646609
DPPA3	HBBP1	CNTFR	LOC440014	LOC646804
DPRXP4	HBD	CNTNAP1	LOC440268	LOC646817
DPYSL4	HBE1	CNTROB	LOC440551	LOC646990
DQX1	HBG1	COCH	LOC440925	LOC647060
DRD1IP	HBM	COL12A1	LOC441282	LOC647195
DSC1	HBS1L	COL17A1	LOC442519	LOC647971
DSCR1L1	HBZ	COL20A1	LOC541472	LOC648600
DSCR4	HDC	COL2A1	LOC641741	LOC648732
DSCR6	HELB	COL4A5	LOC641772	LOC648931
DTNB	HEMGN	COLEC12	LOC641788	LOC649346
DTX3	HEPACAM2	CPXM1	LOC641806	LOC650628
DUOX1	HHIP	CPXM2	LOC641975	LOC650683
DUOX2	HIST1H1D	CPZ	LOC642003	LOC650840
DUOXA2	HIST1H2AM	CRHBP	LOC642628	LOC651556
DUSP5P	HIST1H2BO	CRISP2	LOC642656	LOC652094
DUX4	HIST1H3G	CRISPLD2	LOC642749	LOC652838
DVL1	HIVEP3	CRNKL1	LOC642889	LOC653105
DYNC1I1	HK3	CRSP2	LOC642916	LOC653110
DYNLRB2	HLA-DQA1	CRYGS	LOC642935	LOC653199
EDA2R	HMGB3L1	CSF3R	LOC642968	LOC653652
EFCAB3	HOXA5	CTF1	LOC642969	LOC653696
EFHA2	HOXA9	CTGLF1	LOC643897	LOC654002
EFHB	HOXD3	CTNND2	LOC643959	LOC654069
EFS	HRC	CTSE	LOC644012	LOC727735
EGF	HSFYP1	CTSK	LOC644126	LOC727848
EIF4E1B	HSPA12B	CTSL2	LOC644186	LOC727914
ELMOD1	HSPB2	CUL7	LOC644343	LOC728034
ELOVL3	HSPB6	CXCL14	LOC644500	LOC728291
ELOVL7	HTR7	CXorf45	LOC644589	LOC728802
EMID2	IFI44L	CYB5RL	LOC644641	LOC728830
EMX1	IFIT1L	CYorf15B	LOC644686	LOC728832

EMX2	IFNA21	CYP19A1	LOC644689	LOC728889
ENOX1	IGLL1	CYP27C1	LOC645098	LOC729057
EPC2	IGLL3	CYP2S1	LOC645218	LOC729669
EPHB1	IGSF6	CYP4X1	LOC645312	LOC729837
EPHB2	IKZF1	CYTSB	LOC645522	LOC729954
EPHB3	IL10RA	CYYR1	LOC645848	LOC730392
EPN3	IL13RA2	D4S234E	LOC646109	LOC730809
EPS8L1	IL1RAPL1	DACH1	LOC646272	LOC732419
ERC2	IL20	DACT1	LOC646276	LOC93349
ERCC6	IL2RB	DACT3	LOC646372	LONRF2
ERP44	IL33	DAGLA	LOC646458	LOX
ETAA1	IL3RA	DCDC2	LOC646482	LRRC16
EXPH5	IL7R	DCHS1	LOC646561	LRRC16A
EYA1	IL8RB	DCLK1	LOC646743	LRRC23
FALZ	IL8RBP	DCLK3	LOC646813	MAB21L2
FAM123A	IMMP1L	DCLRE1B	LOC646908	MAP3K9
FAM126A	IQCA1	DCUN1D2	LOC646982	MAP7D3
FAM135A	IRAK3	DDX11	LOC647102	MAPK4
FAM181B	ITGA2B	DDX26B	LOC647121	MAPK8IP1
FAM183A	ITGAD	DENND2A	LOC647229	MDS1
FAM21A	ITGB3	DEPDC1	LOC647328	MED13
FAM23B	ITLN1	DEPDC1B	LOC647509	MESDC1
FAM55C	KCNH2	DGKA	LOC647704	MGC102966
FAM63B	KCNIP1	DHDH	LOC647854	MGC57359
FAM70A	KCNK17	DHFR	LOC647910	MICALL1
FAM80B	KCNMB1	DIAPH3	LOC647920	MIR604
FAM82B	KCNU1	DIO3	LOC647965	MIR98
FAM83B	KDM2B	DIO3OS	LOC648025	MIRLET7D
FAM90A6P	KIAA0748	DKFZp451M2119	LOC648174	MLLT4
FBXL14	KIAA1644	DKFZp686E2433	LOC648517	MNX1
FBXL16	KIF6	DKFZp686I15217	LOC648691	MSX1
FBXL21	KIR2DS5	DLC1	LOC648749	MUC4
FBXO10	KIR3DL1	DLG3	LOC648874	NBPF11
FBXO36	KIR3DL2	DLG4	LOC648993	NCRNA00173
FDXR	KLF1	DLGAP5	LOC649071	NEB
FERMT1	KLRB1	DLK1	LOC649160	NEIL1
FGD4	KRT1	DLL1	LOC649238	NFKBID
FGF11	KRT13	DLX3	LOC649305	NNMT
FGF13	KRT6C	DMD	LOC649431	NOL3
FKBP10	LAG3	DNAJA4	LOC649495	NPAS1
FLJ10088	LAMA4	DNASE1	LOC649503	NPPB
FLJ11292	LAT	DNHD1	LOC649540	NPR1
FLJ12684	LCE1E	DNMT3B	LOC649853	NR4A2
FLJ13305	LCP2	DOCK11	LOC649859	NR6A1
FLJ14166	LDHAL6A	DOCK8	LOC650280	NRF1
FLJ14712	LEFTY1	DOPEY1	LOC650889	NRG1
FLJ16779	LGALS12	DPPA4	LOC651075	NTN1
FLJ22536	LGALS14	DPY19L2P2	LOC651112	OLFM2
FLJ22675	LGALS9	DPYSL3	LOC651123	OR52N5
FLJ23152	LHX2	DSC3	LOC651285	OTP
FLJ25006	LILRA2	DSCR10	LOC651986	OXCT2
FLJ27354	LILRA3	DTL	LOC651987	P2RY2
FLJ30428	LILRA5	DUSP9	LOC652164	PARD6A
FLJ31568	LILRA6	DZIP3	LOC652175	PARD6B
FLJ32810	LILRB2	E2F2	LOC652185	PBXIP1
FLJ35258	LILRB4	E2F7	LOC652456	PDE4DIP

FLJ35429	LILRB5	EBI3	LOC652704	PELI2
FLJ35767	LOC100127887	EDNRA	LOC652712	PER1
FLJ35776	LOC100127937	EDNRB	LOC652768	PGGT1B
FLJ37078	LOC100127952	EFEMP1	LOC653082	PHLDA2
FLJ37644	LOC100127974	EFNB2	LOC653113	PHLDA3
FLJ38773	LOC100128090	EFNB3	LOC653321	PHLDB3
FLJ41327	LOC100128186	EGFL6	LOC653342	PJCG6
FLJ41941	LOC100128292	EGFLAM	LOC653423	PKIB
FLJ42957	LOC100128370	EHD3	LOC653513	PLAUR
FLJ44313	LOC100128374	EHMT1	LOC653515	PLCH2
FLJ45983	LOC100128476	ELOVL4	LOC653520	PLEKHA3
FLJ46838	LOC100128640	ELP2P	LOC653567	PLSCR1
FN1	LOC100128691	EME1	LOC653650	PMS2L1
FOXD1	LOC100128881	EMID1	LOC653857	POLH
FOXF1	LOC100129119	EMILIN2	LOC654433	POT1
FOXI3	LOC100129411	EML1	LOC727751	POU5F1P1
FOXL2	LOC100129532	EMR2	LOC728160	PPM1E
FOXR1	LOC100129602	ENDOD1	LOC728285	PPP1R3B
FREM2	LOC100129751	ENG	LOC728505	PPP4R1L
FRMD3	LOC100129878	ENO2	LOC728558	PQLC2
FRMD7	LOC100130441	ENPP2	LOC728671	PRB3
FSBP	LOC100130492	ENPP5	LOC729179	PRODH
FUT1	LOC100130679	ENTPD1	LOC729231	PRR4
FUT10	LOC100130828	EOMES	LOC729252	PSCA
FZD3	LOC100130855	EPB41L3	LOC729350	PSD
GABBR1	LOC100130938	EPHA4	LOC729562	PSG5
GABBR2	LOC100131164	EPR1	LOC729706	PTGS2
GABRB1	LOC100131283	ERAP1	LOC730041	PTK9
GABRG2	LOC100131335	ERBB2IP	LOC730045	PTPN21
GABRP	LOC100131391	ERCC6L	LOC730058	PWWP2
GAGE12E	LOC100131426	ERP27	LOC730077	PYROXD1
GAGE4	LOC100131726	ERVWE1	LOC730100	PZP
GALIG	LOC100131856	ESRRG	LOC730357	RAB11FIP4
GALNTL1	LOC100131871	ETV2	LOC730360	RAB27A
GAS7	LOC100131892	ETV3	LOC730908	RAB30
GATA2	LOC100131982	ETV4	LOC731002	RAPSN
GATA3	LOC100132146	EVC	LOC732300	RBM24
GCC2	LOC100132167	EVI1	LOC732432	RBMS2P
GDF3	LOC100132707	EVI2A	LOC93432	REL
GDF5OS	LOC100132810	EXO1	LOC96610	RELB
GDF6	LOC100132973	EXOD1	LPIN3	REPS1
GEFT	LOC100133263	EZH1	LRRC2	REV3L
GFPT2	LOC100133315	FABP7	LRRC50	RGAG4
GGT7	LOC100133401	FAM101A	LUZP2	RGMB
GINS1	LOC100133545	FAM123B	MAGEA9B	RHOF
GJA3	LOC100133678	FAM129A	MAK	RIMS4
GJB6	LOC100133771	FAM161A	MANEAL	RIN1
GJB7	LOC100134067	FAM162B	MAP6	RND1
GJC3	LOC100134102	FAM164A	MAPT	RNF111
GLB1L2	LOC100134172	FAM19A4	MARK4	RNF152
GLCCI1	LOC100134229	FAM22D	MCC	RNF217
GLDN	LOC100134348	FAM3B	MDM4	RNU4ATAC
GNB3	LOC100134766	FAM49A	MEF2B	RNY5
GNG8	LOC100192378	FAM54A	MESDC2	RP2
GNGT1	LOC127099	FAM64A	MESP1	RPL32P3
GOLGA6B	LOC138652	FAM65B	MFI2	RPPH1

GOLGA8E	LOC142937	FAM70B	MFSD2	RPTOR
GPATCH8	LOC153684	FAM72A	MGC12982	RRAD
GPD2	LOC200493	FAM72D	MGC26733	RRP1
GPR114	LOC201651	FAM90A2P	MGC40170	RTN2
GPR160	LOC283683	FANCB	MGC48637	RTN4R
GPR161	LOC284352	FANCD2	MICA	SCAF1
GPR3	LOC285033	FANCI	MIR1277	SCRN3
GPR61	LOC285205	FAR1	MIR1282	SDR39U1
GPR84	LOC285453	FAR2	MIR193A	SENP3
GPR87	LOC340113	FAT3	MIR219-2	SERF1A
GRAMD1B	LOC388938	FBLN1	MIR302C	SERPINB9
GRAMD2	LOC389857	FBLN2	MIR561	SFN
GRHL2	LOC390594	FBN2	MIR564	SFRS13B
GRHL3	LOC391727	FBN3	MKX	SFTPD
GRIN3B	LOC400236	FBXL2	MMP10	SFXN3
GRPR	LOC400958	FBXL7	MMP25	SH2D3A
GSK3A	LOC401056	FBXO15	MMP3	SHD
GTPBP5	LOC401286	FBXO45	MMP7	SHROOM1
GUCA1A	LOC440421	FBXO9	MOCS3	SLC16A3
GUCA1B	LOC440570	FCGBP	MOGAT1	SLC1A1
GUCY2C	LOC440900	FER	MOGAT2	SLC27A6
GULP1	LOC441007	FER1L3	MPP3	SLC35F2
HAND1	LOC441018	FGD1	MPPED1	SLC38A11
HAPLN1	LOC441081	FGD3	MST1R	SLC39A13
HAPLN2	LOC441212	FGD5	MT1JP	SLC41A2
HAPLN3	LOC441511	FHDC1	MT3	SLC7A4
HAS2	LOC441554	FHOD1	MUC2	SMPD4
HAS2AS	LOC442366	FHOD3	MUC20	SMPDL3B
HAUS6	LOC541471	FIBCD1	MUCL1	SNORA64
HDX	LOC606724	FIGN	MYH1	SNORA71A
HEPH	LOC641705	FIGNL1	NAT2	SNORD12C
HERV-FRD	LOC642073	FILIP1	NBPF15	SNORD38A
HESX1	LOC642113	FKBPL	NDUFA4L2	SNORD69
HEXA	LOC642154	FLJ10246	NECAB2	SNX12
HMGA2	LOC642342	FLJ11235	NEFL	SOAT1
HOMER1	LOC642553	FLJ11783	NEK10	SPAG1
HOXB3	LOC642580	FLJ12078	NETO1	SPAG4
HOXB4	LOC642621	FLJ13197	NKX2-2	SPRYD5
HOXB7	LOC642707	FLJ14107	NOD2	SRCRB4D
HOXB8	LOC642773	FLJ14213	NOM1	SRRM5
HOXC4	LOC642843	FLJ20209	NOS2	SSTR1
HOXC8	LOC642933	FLJ22639	NOS2A	ST14
HRASLS	LOC642943	FLJ32011	NPAS2	ST7OT1
HRK	LOC642960	FLJ33996	NPTX2	STC2
HS3ST1	LOC643256	FLJ34047	NRG4	STRC
HS6ST1	LOC643403	FLJ35934	NUDT4	STX17
HSD3B1	LOC643432	FLJ37453	NXPH4	SUSD2
HSF1	LOC643461	FLJ39653	OASL	SUSD5
HSF2BP	LOC643505	FLJ39660	ODF4	SYN2
HSN2	LOC643570	FLJ44342	OLA1	TAC1
HSPB7	LOC643713	FLJ45337	OR10J1	TAP2
HSPG2	LOC643731	FLJ45513	OR1B1	TBC1D3C
HTR2C	LOC643733	FLJ90757	OR1S2	TCF7L2
HTRA4	LOC643792	FLRT2	OR2T11	TES
IAPP	LOC643815	FLVCR1	OR4M1	THRSP
ID4	LOC643998	FLYWCH1	OR51S1	THSD4

IFNE	LOC644113	FMNL1	OVCA2	TIGIT
IFT140	LOC644399	FMNL2	PALM2	TMEM100
IGF1R	LOC644573	FMOD	PAX8	TMEM104
IGSF5	LOC644580	FOXE3	PAX9	TMEM132A
IGSF9	LOC644596	FOXH1	PCDHA11	TMEM154
IKZF4	LOC644620	FOXK2	PCK1	TMEM164
IL20RA	LOC644624	FOXM1	PDF	TMEM171
IL6	LOC644830	FRAS1	PDZK1IP1	TMEM191C
INA	LOC645010	FREM1	PIGR	TMEM22
INHA	LOC645038	FRMD4A	PIP5KL1	TMEM38A
INMT	LOC645183	FRMPD3	PLK3	TMPRSS2
INSC	LOC645203	FRZB	PNPLA3	TNFRSF10A
INSL4	LOC645508	FSD1	PNRC1	TNFRSF10C
INSR	LOC645563	FUT8	PPAN-P2RY11	TNNT2
INTU	LOC645661	FXYD6	PPFIA3	TRAF1
IRX2	LOC645722	FYB	PPP1R3A	TRAF3
IRX4	LOC645732	FZD2	PRR8	TRAF3IP1
ISL1	LOC646023	FZD6	PRSS2	TRIM22
ISM2	LOC646049	GAB1	PSMA7	TRIM36
ITGA10	LOC646223	GAGE2B	PTGER1	TRIM43
ITGA8	LOC646300	GAL3ST4	PTGFR	TRK1
ITGB1BP3	LOC646345	GALNT10	PTH2R	TSLP
ITGB4	LOC646509	GALNT12	PTK6	TSN
ITGB6	LOC646521	GALNT6	PTPRT	TTTY14
ITLN2	LOC646562	GAS1	PUS10	UBQLN3
JPH1	LOC646734	GAS2L1	PXDNL	UEVLD
JPH3	LOC646795	GATA5	RANBP3L	UGT3A1
JRK	LOC646853	GBGT1	RAPGEF4	ULK4
KAL1	LOC647135	GCNT1	RDH12	USMG5
KBTBD10	LOC647234	GDPD3	RELL2	USP35
KC6	LOC647251	GFER	RELT	USP43
KCNA5	LOC647506	GFRA2	RIG	VLDLR
KCNAB2	LOC647543	GGTLC1	RNF125	WDR47
KCND1	LOC647570	GIN1	RNF148	WNT3
KCNG1	LOC647579	GINS2	RNF168	WNT5A
KCNH8	LOC647592	GINS4	RNPC2	YKT6
KCNJ13	LOC647742	GJA1	RSHL3	ZBTB8A
KCNJ14	LOC647747	GJA5	RTP4	ZNF222
KCNK10	LOC647855	GJC2	RUNDC2A	ZNF24
KCNK12	LOC647911	GK5	SAA1	ZNF251
KCNK7	LOC647947	GLI3	SAA2	ZNF470
KCNK9	LOC648364	GLIPR2	SALL1	ZNF48
KCNQ4	LOC648470	GLRB	SDS	ZNF555
KCNS1	LOC648615	GLT8D2	SEC14L2	ZNF628
KCTD1	LOC648657	GLUD2	SGK2	ZNF679
KDM4D	LOC649023	gm127	SGMS2	ZNF774
KHDC1	LOC649120	GMEB1	SH3BP2	ZXDB
KIAA0226	LOC649397	GNB4	SIGLEC15	
KIAA0514	LOC649443	GNG2	SLC18A1	
KIAA1024	LOC649580	GNG4	SLC22A10	
KIAA1045	LOC649723	GNGT2	SLC22A6	
KIAA1199	LOC649978	GOLGA7B	SLC24A5	
KIAA1202	LOC650132	GOLGA8A	SLC25A24	
KIAA1244	LOC650494	GOLGA9P	SLC25A30	
KIAA1324L	LOC650566	GP6	SLC25A33	
KIAA1407	LOC650677	GPA33	SLC26A8	

KIAA1549	LOC650831	GPATCH1	SLC28A1	
KIAA1571	LOC650950	GPBAR1	SLC35D1	
KIAA1666	LOC651212	GPC3	SLC45A2	
KIAA1772	LOC651635	GPC4	SLC6A13	
KIF16B	LOC651695	GPC5	SLC6A7	
KIF26B	LOC651738	GPM6B	SLC9A3R2	
KIF27	LOC651751	GPR124	SLCO5A1	
KIF3C	LOC651886	GPR137C	SLED1	
KLC2	LOC651959	GPR156	SLPI	
KLC3	LOC652140	GPSM2	SMCR7	
KLF8	LOC652195	GPSM3	SMCR8	
KLHL17	LOC652255	GRK4	SNORA11C	
KLHL23	LOC652437	GSG2	SNORA13	
KLK3	LOC652479	GSTCD	SNORA21	
KLK6	LOC652491	GSTT1	SNORA3	
KLK7	LOC652493	GUCY1A3	SNORA52	
KLK8	LOC652566	GVIN1	SNORA63	
KLRA1	LOC652657	GYLTL1B	SNORA75	
KLRG2	LOC652679	GYS1	SNORD10	
KRBA2	LOC652850	HAND2	SNORD12	
KREMEN2	LOC653303	HAUS5	SNORD14B	
KRT17	LOC653319	HBA2	SNORD15B	
KRT17P3	LOC653337	HBG2	SNORD33	
KRT23	LOC653600	HBQ1	SNORD34	
KRTAP19-6	LOC653680	HCG27	SNORD48	
KRTAP21-1	LOC653765	HCN3	SNORD55	
KRTAP21-2	LOC653895	HDAC7	SORCS2	
KRTAP6-3	LOC653907	HDGFRP3	SPATA3	
KRTDAP	LOC654203	HECW2	SPRR1B	
KSR1	LOC654264	HELQ	SPTBN5	
L1CAM	LOC727796	HEYL	SRD5A2	
L1TD1	LOC728081	HIF3A	SULT1B1	
LAMA1	LOC728351	HIST1H2AG	SULT2B1	
LAMB2L	LOC728608	HIST1H2BH	SULT6B1	
LASS1	LOC728806	HIST1H4E	SUSD4	
LAYN	LOC728946	HJURP	SYPL2	
LCN15	LOC729088	HKR1	SYT9	
LCORL	LOC729533	HLA-DOA	TAF13	
LDLRAD3	LOC729623	HLA-DPA1	TAS2R43	
LECT1	LOC729792	HMHA1	TBX15	
LEMD1	LOC729799	HMMR	TEC	
LGALS13	LOC729870	HOMER3	TGFA	
LGI2	LOC729957	HOM-TES-103	TGS1	
LGR6	LOC729995	HOPX	THEM5	
LIMK1	LOC730063	HOXA2	TLR1	
LIMK2	LOC730240	HOXB2	TLR3	
LIN28	LOC730378	HPDL	TMEM184A	
LINGO2	LOC730385	HPSE	TMEM45B	
LMBR1L	LOC730474	HS6ST2	TMEM52	
LMF1	LOC730668	HSD17B1	TNFAIP2	
LMO1	LOC730919	HSPA12A	TNFRSF9	
LMOD1	LOC730996	HTR2A	TP53AIP1	
LNX1	LOC731444	HVCN1	TPSG1	
LOC100127915	LOC731486	IDO1	TREH	
LOC100127925	LOC732111	IFT172	TRIM16L	
LOC100128260	LOC732138	IFT80	TRIM31	

LOC100128265	LOC732316	IGDCC3	TRIM6-TRIM34	
LOC100128477	LOC92017	IGDCC4	TRPM8	
LOC100128516	LPAR4	IGF2AS	TRQ1	
LOC100128591	LRAT	IGF2BP1	TRR1	
LOC100128653	LRIG3	IGF2BP3	TSSK2	
LOC100128688	LRRC17	IGFBP5	TTC21A	
LOC100128765	LRRC19	IGFL3	TTC28	
LOC100128857	LRRC26	IGSF1	TTC36	
LOC100128908	LRRC33	IHH	TTC9	
LOC100129148	LRRK1	IKBKE	TTTY6	
LOC100129293	LRRN3	IL11	TUBA4	
LOC100129296	LST1	IL17C	UFSP1	
LOC100129343	LY6G5C	IL17D	UGT1A1	
LOC100129361	LY6G6D	ILDR1	UGT1A10	
LOC100129365	LY6G6F	IMPACT	UGT1A4	
LOC100129387	LY86	INCENP	UGT1A6	
LOC100129466	LY9	INPP5D	UGT1A7	
LOC100129503	LYVE1	INSL5	UGT1A9	
LOC100129534	LYZL1	IP6K3	USP2	
LOC100129541	M160	IPW	VSIG2	
LOC100129588	MAG	IQGAP3	VTRNA1-1	
LOC100129652	MAGEA12	IRAK1BP1	WIPF2	
LOC100129674	MAGEA2	IRAK4	WNT8A	
LOC100129677	MAP2K6	IRX6	XPOT	
LOC100129707	MARCO	ITGA3	ZCCHC16	
LOC100129744	MAZ	ITGA9	ZNF414	
LOC100129808	MCCD1	ITGAM	ZNF460	
LOC100129979	MEF2C	ITM2A	ZNF496	
LOC100130009	MFNG	ITPKB	ZNF57	
LOC100130010	MFSD7	ITPR1	ZNF597	
LOC100130224	MGAT5B	JAK2	ZNF664	
LOC100130255	MGC13168	JAM2	ZNF778	
LOC100130298	MGC5139	JAM3	ZNF98	
LOC100130387	MIA2	JHDM1D	ZXDA	
LOC100130518	MICAL2	JMJD2C		
LOC100130663	MICALCL	JMJD5		
LOC100130798	MIMT1	KAZALD1		
LOC100130840	MIR1281	KBTBD6		
LOC100130921	MIR144	KCNAB1		
LOC100130967	MIR182	KCNE1L		
LOC100131031	MIR1976	KCNJ16		
LOC100131128	MIR365-1	KCNJ2		
LOC100131138	MIR593	KCNK13		
LOC100131165	MMRN1	KCNMB4		
LOC100131210	MNDA	KCTD15		
LOC100131289	MPO	KCTD7		
LOC100131311	MRM1	KDR		
LOC100131326	MRPL42P5	KEL		
LOC100131330	MS4A3	KIAA0040		
LOC100131473	MS4A4A	KIAA0082		
LOC100131578	MS4A7	KIAA0363		
LOC100131621	MSLNL	KIAA0367		
LOC100131644	MSR1	KIAA0460		
LOC100131707	MT1B	KIAA0773		
LOC100131716	MX2	KIAA1107		
LOC100131722	MYH11	KIAA1211		

LOC100131930	MYO16	KIAA1333		
LOC100131967	MYO1F	KIAA1467		
LOC100132060	MYO1G	KIAA1524		
LOC100132153	MYT1	KIAA1641		
LOC100132292	NAALADL1	KIAA1797		
LOC100132395	NAPSA	KIAA1908		
LOC100132413	NAPSB	KIF11		
LOC100132428	NCF1C	KIF14		
LOC100132456	NCF2	KIF15		
LOC100132549	NCKAP1L	KIF18A		
LOC100132640	NDUFB4	KIF20A		
LOC100132678	NGFR	KIF23		
LOC100132724	NHLRC4	KIF24		
LOC100132794	NID1	KIF26A		
LOC100132920	NKG7	KIF2C		
LOC100132942	NNAT	KIF4A		
LOC100132960	NOTCH4	KIF5C		
LOC100133070	NR2F1	KIF7		
LOC100133099	NRG3	KIFC1		
LOC100133118	NRXN2	KIFC3		
LOC100133144	NTM	KIT		
LOC100133172	NTSR1	KITLG		
LOC100133234	OGN	KLHDC10		
LOC100133422	OIT3	KLK11		
LOC100133430	OLFM1	KNTC1		
LOC100133459	OLFM4	KRI1		
LOC100133503	OR13C4	L3MBTL		
LOC100133554	OR4S1	LAMA2		
LOC100133555	OR52B6	LAMC3		
LOC100133558	OR5K4	LAT1-3TM		
LOC100133627	P2RX1	LDOC1		
LOC100133747	P2RY13	LEF1		
LOC100133760	P2RY8	LEFTY2		
LOC100133981	PACAP	LEPREL1		
LOC100134002	PADI4	LEPREL2		
LOC100134035	PAQR5	LGALS2		
LOC100134073	PARVG	LGR4		
LOC100134101	PCDH12	LHB		
LOC100134160	PCDH9	LIFR		
LOC100134259	PCDHB14	LIN28B		
LOC100134368	PDE1A	LIN7B		
LOC100134396	PDE2A	LIN9		
LOC100134412	PDE4B	LINGO1		
LOC100134466	PDE6G	LMNB1		
LOC100134474	PF4V1	LMO2		
LOC100134498	PGA3	LOC100127971		
LOC100134528	PGA5	LOC100127983		
LOC100134539	PGLYRP1	LOC100127999		
LOC100134550	PHOSPHO1	LOC100128139		
LOC100134587	PI16	LOC100128191		
LOC100134660	PKD2L1	LOC100128230		
LOC100134700	PLCB2	LOC100128252		
LOC100144604	PLD4	LOC100128398		
LOC100188949	PLEKHH2	LOC100128675		
LOC100192379	PLGLA	LOC100128729		
LOC119358	PLIN	LOC100128737		

LOC124216	PLVAP	LOC100128888		
LOC133993	PNMT	LOC100128974		
LOC134505	POU2AF1	LOC100129064		
LOC144481	POU2F3	LOC100129095		
LOC146439	PPAPDC1A	LOC100129129		
LOC147645	PPP1R14D	LOC100129144		
LOC148137	PPP1R16B	LOC100129149		
LOC151300	PRAM1	LOC100129195		
LOC151457	PRKCB	LOC100129410		
LOC154761	PRKCB1	LOC100129540		
LOC155100	PRND	LOC100129571		
LOC158572	PRO0132	LOC100129580		
LOC162073	PRPS1L1	LOC100129636		
LOC169834	PRR12	LOC100129637		
LOC199800	PRSS21	LOC100129708		
LOC220729	PRSSL1	LOC100129905		
LOC283332	PSCD4	LOC100129977		
LOC283588	PTAFR	LOC100129988		
LOC283767	PTCRA	LOC100130123		
LOC283874	PTGS1	LOC100130217		
LOC284296	PTPRB	LOC100130229		
LOC284297	PTPRCAP	LOC100130367		
LOC284620	PTPRN2	LOC100130413		
LOC285047	PTPRO	LOC100130458		
LOC285500	PVALB	LOC100130506		
LOC338963	PYHIN1	LOC100130555		
LOC339047	RAB3A	LOC100130557		
LOC339535	RAB3C	LOC100130592		
LOC339799	RAMP3	LOC100130776		
LOC340156	RAP2B	LOC100130904		
LOC340357	RASA3	LOC100130935		
LOC341689	RASL10A	LOC100130952		
LOC344595	RASSF10	LOC100131139		
LOC344741	RETN	LOC100131243		
LOC374443	RFPL2	LOC100131253		
LOC387770	RFPL3	LOC100131265		
LOC387856	RFTN2	LOC100131271		
LOC388436	RFX2	LOC100131608		
LOC388458	RGS18	LOC100131894		
LOC388494	RGS6	LOC100132011		
LOC388559	RHAG	LOC100132112		
LOC388565	RHBG	LOC100132119		
LOC388692	RHCE	LOC100132228		
LOC388820	RHOH	LOC100132287		
LOC389118	RHOJ	LOC100132439		
LOC389332	RIPK3	LOC100132475		
LOC390372	RNASE2	LOC100132491		
LOC390414	RNASE3	LOC100132565		
LOC391045	RNF175	LOC100132901		
LOC392382	RNY4	LOC100133076		
LOC392635	ROBO4	LOC100133080		
LOC392787	RSAD2	LOC100133200		
LOC400214	RUNDC3A	LOC100133220		
LOC400743	S100A12	LOC100133482		
LOC400831	S100A8	LOC100133583		
LOC400955	SAMD9L	LOC100133666		

LOC401002	SAMSN1	LOC100133667		
LOC401007	SASH3	LOC100133673		
LOC401237	SCARNA14	LOC100133686		
LOC401252	SCARNA18	LOC100133737		
LOC401431	SCGB3A1	LOC100133744		
LOC401650	SCN7A	LOC100133797		
LOC402116	SDPR	LOC100134009		
LOC402693	SEC14L5	LOC100134052		
LOC404266	SELL	LOC100134098		
LOC415056	SELP	LOC100134241		
LOC439936	SEMA4A	LOC100134261		
LOC440132	SERPINA13	LOC100134301		
LOC440459	SGIP1	LOC100134444		
LOC440585	SH2D2A	LOC100134563		
LOC440864	SH2D3C	LOC100134624		
LOC440910	SH3RF3	LOC100134688		
LOC440978	SIGLEC10	LOC100134703		
LOC441061	SIGLEC11	LOC100134711		
LOC441114	SIGLEC16	LOC100134734		
LOC441208	SIGLEC7	LOC100170939		
LOC441237	SIT1	LOC126520		
LOC441294	SLA2	LOC148709		
LOC441528	SLAMF6	LOC149224		
LOC441737	SLC10A4	LOC153561		
LOC442041	SLC11A1	LOC202134		
LOC442057	SLC13A3	LOC221442		
LOC554207	SLC17A8	LOC282997		
LOC641365	SLC1A7	LOC283755		
LOC641765	SLC22A16	LOC283932		
LOC641801	SLC25A21	LOC284023		
LOC641999	SLC26A1	LOC284648		
LOC642062	SLC26A10	LOC285359		
LOC642097	SLC26A3	LOC285908		
LOC642109	SLC35D3	LOC285943		
LOC642156	SLC4A1	LOC286002		
LOC642267	SLC5A11	LOC286135		
LOC642325	SLC8A3	LOC286367		
LOC642369	SLFN13	LOC338667		
LOC642486	SLITRK1	LOC338799		
LOC642570	SMCP	LOC339483		
LOC642635	SNORA65	LOC346887		
LOC642661	SNORA78	LOC387934		
LOC642759	SNORD114-1	LOC388312		
LOC642780	SNORD114-3	LOC388397		
LOC642787	SNORD25	LOC389102		
LOC642809	SNORD4B	LOC389465		
LOC642852	SNX18	LOC389634		
LOC642980	SNX31	LOC389816		
LOC643012	SNX32	LOC390705		
LOC643015	SOS2	LOC390940		
LOC643018	SOX12	LOC391429		
LOC643187	SOX6	LOC392843		
LOC643222	SPAG6	LOC399829		
LOC643233	SPATA4	LOC400464		
LOC643240	SPESP1	LOC400879		
LOC643272	SPI1	LOC400986		

LOC643293	SPIB	LOC401052		
LOC643313	SPINK2	LOC401620		
LOC643373	SPTA1	LOC401622		
LOC643382	SPTB	LOC439949		
LOC643389	SRD5A3	LOC440145		
LOC643396	SRL	LOC440258		
LOC643401	SRY	LOC440836		
LOC643423	STAB1	LOC440928		
LOC643451	STAB2	LOC441046		
LOC643605	SUCNR1	LOC441066		
LOC643647	SULT1C2	LOC441193		
LOC643664	SV2B	LOC441268		
LOC643841	SYCP1	LOC441864		
LOC643981	TACR3	LOC441907		
LOC643985	TAL1	LOC442075		
LOC644019	TBC1D10C	LOC442181		
LOC644083	TBCEL	LOC442597		
LOC644092	TCL1A	LOC493869		
LOC644276	TCTEX1D1	LOC550112		
LOC644284	TDH	LOC554208		
LOC644297	TEX13A	LOC641518		
LOC644313	TGM6	LOC641710		
LOC644353	THBS2	LOC641823		
LOC644391	TIE1	LOC641978		
LOC644415	TIGD1	LOC642412		
LOC644424	TIMD4	LOC642458		
LOC644436	TLR4	LOC642636		
LOC644544	TLR5	LOC642678		
LOC644612	TMC8	LOC642859		
LOC644644	TMCC2	LOC642909		
LOC644672	TMCO5A	LOC642953		
LOC644701	TMEM119	LOC642995		
LOC644760	TMEM155	LOC643008		
LOC644763	TMEM163	LOC643109		
LOC644844	TMEM169	LOC643339		
LOC644992	TMEM173	LOC643342		
LOC645135	TMEM202	LOC643388		
LOC645172	TMEM204	LOC643466		
LOC645195	TMEM26	LOC643624		
LOC645212	TMEM71	LOC643872		
LOC645241	TMPRSS9	LOC643888		
LOC645321	TNF	LOC643918		
LOC645330	TNFAIP8L3	LOC644001		
LOC645365	TNFRSF11B	LOC644043		
LOC645431	TNFRSF6B	LOC644254		
LOC645464	TNNI2	LOC644404		
LOC645478	TNS4	LOC644496		
LOC645627	TOP1P1	LOC644590		
LOC645723	TRIM58	LOC644617		
LOC645743	TRIM61	LOC644919		
LOC645835	TRPA1	LOC645001		
LOC645963	TRPM3	LOC645079		
LOC646008	TSPAN32	LOC645159		
LOC646012	TTTY11	LOC645367		
LOC646043	TTTY5	LOC645550		
LOC646067	TTYH1	LOC645566		

LOC646111	TUBB1	LOC645638		
LOC646128	UBE2B	LOC645726		
LOC646154	UBE2MP1	LOC645978		
LOC646194	UBXD7	LOC646100		
LOC646208	UCP3	LOC646123		
LOC646312	UPK1A	LOC646144		
LOC646332	UPK3A	LOC646513		
LOC646434	UPP2	LOC646533		
LOC646446	VAV1	LOC646548		
LOC646491	VNN2	LOC646572		
LOC646496	VPREB1	LOC646576		
LOC646568	WDR40B	LOC646632		
LOC646750	WDR49	LOC646674		
LOC646769	WDR76	LOC646762		
LOC646774	WDR78	LOC646808		
LOC646779	WDR88	LOC646916		
LOC646906	WFDC1	LOC647474		
LOC647012	WNT2B	LOC647784		
LOC647054	XAGE1E	LOC647805		
LOC647250	XK	LOC648237		
LOC647262	XLKD1	LOC648434		
LOC647264	YPEL4	LOC648608		
LOC647288	ZBTB38	LOC648758		
LOC647488	ZC3H13	LOC648984		
LOC647515	ZCWPW2	LOC649422		
LOC647568	ZEB2	LOC649445		
LOC647589	ZNF121	LOC649639		
LOC647718	ZNF132	LOC649841		
LOC647827	ZNF37A	LOC649897		
LOC647928	ZNF510	LOC649970		
LOC647955	ZNF662	LOC650034		
LOC647979	ZSCAN22	LOC650128		
LOC647987		LOC650227		
LOC648057		LOC650346		
LOC648130		LOC650406		
LOC648153		LOC650526		
LOC648169		LOC650546		
LOC648196		LOC650815		
LOC648226		LOC651302		
LOC648293		LOC651309		
LOC648342		LOC651380		
LOC648366		LOC651558		
LOC648570		LOC651997		
LOC648585		LOC652324		
LOC648814		LOC652377		
LOC648963		LOC652589		
LOC649025		LOC652615		
LOC649186		LOC652694		
LOC649210		LOC652699		
LOC649270		LOC652755		
LOC649299		LOC652837		
LOC649379		LOC652839		
LOC649500		LOC653158		
LOC649613		LOC653188		
LOC649620		LOC653210		
LOC649680		LOC653234		

LOC649711		LOC653354		
LOC649754		LOC653468		
LOC649801		LOC653487		
LOC649977		LOC653545		
LOC650155		LOC653604		
LOC650263		LOC653663		
LOC650339		LOC653701		
LOC650562		LOC653717		
LOC650580		LOC653877		
LOC650706		LOC653878		
LOC651102		LOC654109		
LOC651169		LOC654123		
LOC651213		LOC727759		
LOC651520		LOC727882		
LOC651745		LOC727884		
LOC651772		LOC727913		
LOC651777		LOC727948		
LOC652002		LOC727997		
LOC652078		LOC728115		
LOC652097		LOC728170		
LOC652234		LOC728178		
LOC652291		LOC728275		
LOC652326		LOC728411		
LOC652330		LOC728417		
LOC652458		LOC728452		
LOC652470		LOC728465		
LOC652534		LOC728470		
LOC652570		LOC728509		
LOC652577		LOC728653		
LOC652630		LOC728686		
LOC652674		LOC728715		
LOC652675		LOC728942		
LOC652688		LOC728971		
LOC652697		LOC729065		
LOC652713		LOC729137		
LOC652771		LOC729143		
LOC652846		LOC729157		
LOC652900		LOC729212		
LOC652904		LOC729260		
LOC653100		LOC729272		
LOC653111		LOC729372		
LOC653157		LOC729389		
LOC653197		LOC729558		
LOC653204		LOC729602		
LOC653269		LOC729684		
LOC653316		LOC729764		
LOC653349		LOC729793		
LOC653471		LOC729810		
LOC653539		LOC729828		
LOC653596		LOC729858		
LOC653796		LOC729941		
LOC653853		LOC730051		
LOC653876		LOC730092		
LOC653962		LOC730153		
LOC654078		LOC730173		
LOC654128		LOC730183		

LOC654253		LOC730286		
LOC727721		LOC730644		
LOC727832		LOC730877		
LOC727868		LOC730994		
LOC727901		LOC730995		
LOC727924		LOC731742		
LOC727935		LOC731751		
LOC727980		LOC731915		
LOC728116		LOC732146		
LOC728190		LOC732450		
LOC728205		LOC81691		
LOC728247		LOC90120		
LOC728308		LOC90499		
LOC728362		LOC91431		
LOC728400		LOC91461		
LOC728448		LOC91661		
LOC728493		LOC92659		
LOC728518		LOXHD1		
LOC728519		LPAR1		
LOC728530		LPAR2		
LOC728543		LPAR5		
LOC728591		LPHN1		
LOC728664		LPL		
LOC728678		LRMP		
LOC728683		LRP6		
LOC728711		LRRC37A4		
LOC728743		LRRC38		
LOC728790		LRRC6		
LOC728876		LRRN2		
LOC728924		LSAMP		
LOC728929		LTB4R		
LOC729008		LTBP2		
LOC729020		LUM		
LOC729046		LXN		
LOC729051		LYPD6B		
LOC729173		LYSMD1		
LOC729176		LYST		
LOC729198		MAD2L1		
LOC729209		MAGOHB		
LOC729234		MAP2		
LOC729351		MAP3K13		
LOC729374		MAP4K1		
LOC729408		MAP7D2		
LOC729409		MAPK10		
LOC729433		MARCH9		
LOC729438		MBLAC2		
LOC729530		MBOAT2		
LOC729570		MCF2L		
LOC729609		MCM10		
LOC729642		MCM2		
LOC729645		MCM3APAS		
LOC729659		MECP2		
LOC729667		MED18		
LOC729683		MEG8		
LOC729739		MELK		
LOC729806		MEP1A		

LOC729885		METTL4		
LOC729950		MEX3B		
LOC729952		MFAP4		
LOC730007		MFAP5		
LOC730012		MGAT3		
LOC730024		MGC16121		
LOC730036		MGC27345		
LOC730087		MGC27348		
LOC730130		MGC39900		
LOC730234		MGC5457		
LOC730387		MICB		
LOC730393		MIPOL1		
LOC730396		MIR1909		
LOC730413		MIR25		
LOC730427		MIR886		
LOC730517		MITF		
LOC730952		MKI67		
LOC731139		MKS1		
LOC731227		MLF1		
LOC731642		MLF1IP		
LOC731718		MMP11		
LOC731835		MMP9		
LOC731895		MND1		
LOC731932		MORN4		
LOC732160		MPZ		
LOC732229		MRE11A		
LOC732443		MSH2		
LOH12CR2		MSH5		
LOH3CR2A		MSLN		
LOXL1		MSRB3		
LOXL3		MTBP		
LPCAT2		MTHFD2		
LPHN3		MTHFD2L		
LPPR3		MTL5		
LRCH1		MUM1L1		
LRCH2		MXD3		
LRFN4		MXRA5		
LRFN5		MXRA8		
LRP1B		MYB		
LRP2		MYBL2		
LRP4		MYCBP		
LRP8		MYCL1		
LRRC16B		MYCN		
LRRC37A		MYCT1		
LRRC49		MYH3		
LRRC69		MYL1		
LRRN1		MYL2		
LRRN4		MYL4		
LSM11		MYL9		
LY6G6C		MYLK4		
LYG1		MYO10		
LYPD1		MYO19		
LYPD5		MYO9A		
LYPD6		MYOF		
MAGEC2		MYOZ3		
MAGI1		NAALAD2		

MAL		NACC1		
MAMDC2		NANOS3		
MAML3		NBEA		
MAP1A		NCAPG		
MAP1B		NCAPG2		
MAP1LC3B2		NCF4		
MAP9		NCRNA00085		
MAPK8IP2		NCRNA00153		
MAPKBP1		NDUFB1		
MARCH11		NDUFS1		
MARK1		NEDD1		
MARVELD1		NEFH		
MATN1		NEIL3		
MATN3		NES		
MBD5		NETO2		
MBL1P1		NEURL4		
MCF2L2		NEXN		
MCOLN2		NFE2		
MCOLN3		NID2		
MECR		NLGN4X		
MED12L		NLGN4Y		
MEGF6		NLK		
MEI1		NLRC3		
MEIS3		NLRP2		
METT10D		NMU		
METTL2B		NOS3		
MFSD6L		NOSTRIN		
MFSD9		NOTCH3		
MGC11082		NOX4		
MGC12965		NPAT		
MGC13005		NPDC1		
MGC15634		NPHP1		
MGC20983		NPHP4		
MGC23270		NPY		
MGC24103		NR1D1		
MGC26718		NR2C2		
MGC35440		NRCAM		
MGC42630		NRGN		
MGC45800		NSUN7		
MGC52498		NT5DC2		
MIA		NT5M		
MINK1		NTS		
MIP		NUDT11		
MIR1247		NUF2		
MIR128-2		NUP210		
MIR181C		NUP62CL		
MIR1915		NXNL1		
MIR205		NYNRIN		
MIR21		OBSCN		
MIR212		ODF2L		
MIR30C2		OIP5		
MIR326		OLFML1		
MIR448		OLFML2A		
MIR450A1		OLFML3		
MIR532		OLR1		
MIR577		OR1J1		

MIR642		OR1L8		
MIR708		OR2T12		
MLC1		OR2W3		
MLH3		ORC4L		
MLLT1		OSBP2		
MMP1		OSBPL10		
MMP14		OSBPL3		
MMP2		OSBPL5		
MMP21		OSM		
MMP24		OTUD3		
MMP28		OTUD7B		
MOBKL2B		OXCT1		
MORN3		P2RY5		
MOXD1		PABPC4L		
MPP7		PACRGL		
MPPED2		PACSIN1		
MRAP2		PAMR1		
MRC2		PAPPA		
MREG		PAQR8		
MRGPRX1		PBK		
MRGPRX3		PBX1		
MRGPRX4		PCBD2		
MSX2		PCDH17		
MT4		PCDH18		
MTHFSD		PCDH7		
MTMR1		PCYOX1L		
MTMR7		PDE5A		
MTUS2		PDGFB		
MUC15		PDGFD		
MUC16		PDGFRA		
MURC		PEAR1		
MYBL1		PEG10		
MYBPC3		PEG3		
MYBPHL		PFN4		
MYL3		PGAP1		
MYL7		PGBD1		
MYO3A		PGBD2		
MYO5A		PGM2L1		
MYOZ1		PGM5		
NACAD		PHF21B		
NAP1L3		PHYHIPL		
NAP1L6		PI4KA		
NAT11		PIAS3		
NAV1		PIP4K2B		
NBR2		PIP5K1B		
NCAM1		PIP5K2A		
NCCRP1		PKIA		
NEBL		PKMYT1		
NELL2		PKN3		
NF1		PLA2G12A		
NFKBIL2		PLA2G7		
NGF		PLAC8		
NHS		PLAC9		
NIPAL4		PLAGL1		
NKAIN4		PLAT		
NKPD1		PLEKHG4		

NKX2-3		PLEKHH1		
NLGN1		PLK1		
NLRP12		PLK4		
NLRP7		PLXDC2		
NME5		PLXNA2		
NMNAT2		PMAIP1		
NOD1		PML		
NOG		PMS1		
NOL4		PMS2L2		
NOVA1		PNMAL1		
NPAS3		POGZ		
NPNT		POLL		
NR0B1		POLQ		
NRXN3		POLR3D		
NTF4		POMC		
NTF5		POSTN		
NTN5		POU2F1		
NTNG1		PPP1R14A		
NTRK2		PPP1R1B		
NUDCD1		PPP1R3D		
NUDT13		PPP1R3F		
NUMBL		PPP2R3B		
NXF4		PRDM1		
NXPH2		PREX1		
OBSL1		PRICKLE1		
OCA2		PRKACB		
OCLN		PRKCQ		
ODAM		PRKX		
ODZ3		PRKY		
ODZ4		PROK1		
OPHN1		PROM1		
OR2H2		PRR11		
OR2L13		PRR15		
OR7E91P		PRRT1		
OSAP		PRRT2		
OSR2		PRSS35		
OTUB2		PRTFDC1		
OTX2		PSCDBP		
OVOL1		PSG2		
OVOL2		PSG6		
OXGR1		PSG7		
P2RX2		PSORS1C1		
P2RX6		PSRC1		
P2RY6		PTGIS		
PA2G4P4		PTH1R		
PADI2		PTK7		
PAIP2B		PTN		
PAK6		PTP4A3		
PAPOLG		PTTG3P		
PAQR6		PUS7L		
PAR5		PWWP2A		
PARD3		PYGO2		
PARG		QPCTL		
PARM1		QRSL1		
PART1		RAB25		
PATE1		RAB34		

PATZ1		RAB38		
PBX4		RAB3B		
PCBP3		RACGAP1		
PCDH10		RAD51AP1		
PCDH11X		RAD54B		
PCDH11Y		RAD54L		
PCDH19		RALGPS2		
PCDHA1		RAMP2		
PCDHA2		RAP1A		
PCDHA3		RARB		
PCDHA4		RASL11B		
PCDHB10		RASL12		
PCDHB13		RAVER2		
PCDHB16		RAX2		
PCDHB17		RB1		
PCDHB18		RBL1		
PCDHB2		RBM19		
PCDHB3		RBMS2		
PCDHB4		RBP2		
PCDHB5		RCOR2		
PCDHGA12		RCSD1		
PCLO		RDM1		
PCNXL2		RECK		
PCP4		RECQL4		
PDAP1		RECQL5		
PDE4D		REEP1		
PDE6B		RELN		
PDE8B		RENBP		
PDGFA		REP15		
PDK1		RFPL1		
PDPN		RFXAP		
PDXDC2		RFXDC2		
PDZD2		RGPD4		
PDZD3		RGS16		
PDZRN3		RGS17		
PELI3		RGS5		
PFKFB2		RHBDL3		
PGBD4		RHOBTB2		
PGF		RIMKLB		
PHACTR1		RIMS3		
PHC3		RIN3		
PHF6		RNASE6		
PHF8		RNASEH2C		
PI15		RNF144A		
PIB5PA		RNF150		
PIP5K1A		RNF157		
PITX1		RNF182		
PITX2		RNF43		
PIWIL4		RNFT2		
PKD1L2		RNU105C		
PKNOX2		RNU2-1		
PKP3		RNU4-2		
PLA2G10		ROBO1		
PLAC1		ROPN1B		
PLAC2		ROR1		
PLCD3		RPP25		

PLCE1		RRM2		
PLCXD3		RSBN1L		
PLD5		RSF1		
PLEKHG2		RSPO3		
PLEKHG4B		S100A3		
PLEKHG5		S1PR3		
PLSCR5		S1PR4		
PMFBP1		SAC		
PNCK		SACS		
POLI		SALL2		
POPDC2		SALL4		
PORCN		SAMD1		
POTEG		SAMD14		
POU6F1		SASS6		
PPEF1		SCAMP5		
PPIL2		SCAND2		
PPIL6		SCAND3		
PPM1J		SCARA3		
PPP1R9A		SCARF2		
PRDM5		SCARNA16		
PRDM7		SCLT1		
PRH1		SCRN1		
PRICKLE3		SCTR		
PRINS		SDCCAG8		
PRKAA2		SDK2		
PRKCE		SDR16C5		
PROCA1		SEC31B		
PROM2		SEMA3F		
PRPF39		SEMA4D		
PRPH		SEMA5A		
PRRX1		SEMA6A		
PRSS12		SEPT10		
PRSS16		SERHL		
PRSS22		SERINC4		
PRTG		SERINC5		
PSD2		SERPINI1		
PTCD3		SESN3		
PTCH1		SEZ6L2		
PTCHD1		SFI1		
PTER		SFMBT2		
PTGES		SFRP1		
PTPDC1		SGCB		
PTPN13		SGOL1		
PTPN14		SGOL2		
PTPRR		SGPP2		
PVRL4		SGSM1		
PWP2		SH3BP1		
RABL5		SH3GL3		
RAD18		SH3PXD2B		
RAD52		SHISA2		
RADIL		SHPRH		
RALYL		SIX4		
RANBP2L1		SKA1		
RASA2		SLA		
RASAL2		SLAMF7		
RASEF		SLC12A6		

RASGRP1		SLC14A1		
RASL10B		SLC16A9		
RASSF9		SLC1A3		
RBAK		SLC1A5		
RBM11		SLC22A15		
RBM20		SLC22A4		
RBM35A		SLC24A3		
RBMS3		SLC25A36		
RBMY3AP		SLC2A5		
RCAN2		SLC35B4		
RCAN3		SLC39A10		
RCBTB1		SLC43A2		
REEP2		SLC45A4		
REG1A		SLC6A8		
RERG		SLC7A8		
RETNLB		SLC9A4		
RFPL3S		SLC9A9		
RFT1		SLCO2A1		
RFX3		SLCO3A1		
RFX6		SLIT2		
RGAG1		SLIT3		
RGL3		SLMO1		
RGP1		SLN		
RGPD5		SMAD6		
RGPD8		SMARCD3		
RGS11		SMC1A		
RGS20		SMC6		
RGS4		SMG6		
RGS7BP		SNAP25		
RIC3		SNCA		
RICTOR		SNCAIP		
RIMBP3B		SNORA26		
RIMS2		SNORA28		
RLN1		SNORA29		
RLN2		SNORA42		
RNF180		SNORA5B		
RNF39		SNORA70B		
RNU105A		SNORA72		
RNU11		SNORA77		
RNU4-1		SNORD11		
ROR2		SNORD13		
RPA4		SNORD17		
RPESP		SNORD52		
RPRM		SNORD80		
RSPO1		SNRPE		
RSPO2		SOSTDC1		
RTKN2		SOX11		
RUFY2		SP6		
RYR2		SPAG5		
RYR3		SPC25		
SALL3		SPG20		
SAMD10		SPOCK2		
SAMD8		SPON1		
SATB1		SPTBN4		
SBSN		SPTLC2		
SCARNA3		SRGAP1		

SCD5		SRPX		
SCN2B		SRR		
SCRG1		SSH1		
SCUBE3		SSX2IP		
SCXA		ST6GALNAC3		
SDK1		ST8SIA4		
SEC15L2		STAG3		
SEC61A2		STAR		
SELE		STEAP2		
SELV		STMN2		
SEMA3A		STOX1		
SEMA3B		STX18		
SEMA3C		STXBP1		
SEMA5B		STXBP5		
SEMA6D		SUDS3		
SEMA7A		SULF1		
SENP1		SULT4A1		
SEPT1		SUPT3H		
SEPT13		SUPT7L		
SEPT3		SUZ12P		
SERPINB7		SVEP1		
SERPINE3		SYDE1		
SERTAD4		SYK		
SFRP2		SYNJ2		
SGCG		TAAR8		
SH2B2		TACC3		
SH3BGR		TADA2A		
SH3BP5		TAF1A		
SH3GL2		TAF1L		
SHOX2		TAF5		
SIKE		TANC2		
SIM2		TARBP1		
SIRPD		TARSL2		
SKIL		TAS2R10		
SLC12A5		TBC1D22B		
SLC13A4		TBC1D5		
SLC15A2		TBX1		
SLC16A1		TBX19		
SLC16A14		TBX2		
SLC16A6		TCAM1		
SLC1A6		TCEAL7		
SLC22A11		TCF19		
SLC2A14		TCF21		
SLC30A2		TCF4		
SLC30A8		TCL1B		
SLC34A1		TEAD4		
SLC35E2		TEK		
SLC35F1		TEKT1		
SLC35F3		TESSP5		
SLC38A8		TET1		
SLC39A2		TEX11		
SLC4A11		TFAP4		
SLC4A3		TFCP2L1		
SLC4A8		TFPI2		
SLC5A3		TGM1		
SLC6A15		THBS4		

SLC6A4		THY1			
SLC6A6		TIGD7			
SLC7A11		TM4SF18			
SLC9A2		TMC6			
SLC9A5		TMEM107			
SLCO1A2		TMEM117			
SLCO6A1		TMEM118			
SLFN5		TMEM128			
SMA5		TMEM132B			
SMAD9		TMEM161B			
SMN2		TMEM168			
SMOC2		TMEM194			
SMPX		TMEM194B			
SMTNL2		TMEM8B			
SMYD5		TMOD2			
SNAP91		TMPRSS13			
SNORA11E		TMSB15A			
SNORA31		TMSB4Y			
SNORA49		TNFRSF25			
SNORA55		TNFSF4			
SNORA73A		TNNC1			
SNORA73B		TOP2A			
SNORA84		TOX2			
SNORD123		TP53I11			
SNORD21		TPX2			
SNORD35A		TRAF2			
SNORD73A		TRAF5			
SOCS7		TRAIP			
SOD3		TREML2			
SOHLH2		TRIB2			
SORCS1		TRIL			
SOS1		TRIM6			
SOX15		TRIM65			
SOX17		TRIM67			
SOX2		TRIM71			
SOX21		TRIP13			
SOX3		TRO			
SP140L		TROAP			
SP5		TRPC1			
SP8		TRPV1			
SPANXA2		TRPV2			
SPANXB1		TSPAN18			
SPATA17		TSPAN5			
SPATA18		TSPYL3			
SPATA22		TSPYL5			
SPATA6		TTC18			
SPDYA		TTC25			
SPEF2		TTC26			
SPINK5		TTC39B			
SPINT1		TTK			
SPNS3		TUBAL3			
SPOCK1		TUBB4			
SPSB4		TUBD1			
SRD5A2L2		TUSC3			
SSPN		TWIST1			
SST		TXNDC16			

SSX3		TXNL4B		
STAC		UAP1L1		
STC1		UBASH3B		
STELLAR		UBE2C		
STK38L		UBE2CBP		
STON1		UBE2T		
STRA6		UBN2		
STS		UBQLNL		
STX19		UBR1		
STYK1		UCA1		
SUFU		UCP2		
SULT1C4		UHMK1		
SUMO4		UHRF1		
SV2A		UNC13D		
SVOPL		UNC5C		
SYCE2		USP32		
SYN1		USP6		
SYNC1		USP9Y		
SYNPO2		UST		
SYNPR		VANGL2		
SYT1		VAPB		
SYT13		VASH1		
SYT3		VASH2		
SYT6		VAT1L		
SYTL1		VAV3		
SYTL3		VCAM1		
TAC3		VCAN		
TAC4		VEGFC		
TAF1		VPRBP		
TAGLN3		VPS13C		
TBC1D1		WAPAL		
TBC1D26		WASPIP		
TBC1D3H		WBSCR19		
TBKBP1		WDHD1		
TBN		WDR27		
TBRG1		WDR35		
TC2N		WDR5B		
TCEAL2		WDR62		
TCERG1L		WDR67		
TCL6		WDR86		
TEX9		WFIKKN1		
TFAP2A		WHSC1		
TFAP2B		WHSC1L1		
TFAP2C		WIPF1		
TGFBR1		WNK4		
THAP3		WSCD1		
THAP9		WTIP		
THEX1		XAGE1D		
THSD1P		XRCC3		
THSD3		YTHDC2		
TIAM1		ZBTB46		
TIAM2		ZDHHC8P		
TIMP4		ZFP112		
TINAGL1		ZFP14		
TLE3		ZFP30		
TMC7		ZFP37		

TMCC3		ZFP82		
TMEFF1		ZKSCAN4		
TMEM108		ZMYND17		
TMEM125		ZNF135		
TMEM16B		ZNF137		
TMEM200A		ZNF167		
TMEM231		ZNF175		
TMEM31		ZNF182		
TMEM40		ZNF184		
TMEM55A		ZNF185		
TMEM59L		ZNF2		
TMEM67		ZNF202		
TMEM68		ZNF211		
TMEM86A		ZNF213		
TMEM87B		ZNF224		
TMEM90B		ZNF225		
TMEM92		ZNF230		
TMPRSS7		ZNF234		
TMTC1		ZNF248		
TMTC2		ZNF273		
TncRNA		ZNF28		
TNFRSF8		ZNF280B		
TNFSF12-TNFSF13		ZNF283		
TNFSF9		ZNF285A		
TNKS		ZNF286C		
TNKS2		ZNF311		
TNNI3		ZNF334		
TNNT1		ZNF335		
TNRC18		ZNF346		
TOX		ZNF383		
TP53TG3		ZNF415		
TP63		ZNF418		
TP73L		ZNF431		
TPBG		ZNF439		
TPCN1		ZNF462		
TPPP3		ZNF471		
TRAF3IP3		ZNF484		
TREM1		ZNF485		
TRERF1		ZNF491		
TREX1		ZNF493		
TRH		ZNF502		
TRIM17		ZNF514		
TRIM62		ZNF521		
TRIM63		ZNF525		
TRIML2		ZNF532		
TRPV6		ZNF543		
TSGA10		ZNF571		
TSPAN2		ZNF573		
TSPY1		ZNF594		
TSPY3		ZNF605		
TTC30B		ZNF606		
TTC7A		ZNF608		
TTLL7		ZNF609		
TTN		ZNF611		
TTTY15		ZNF616		
TTTY17A		ZNF641		

TTYH2		ZNF681		
TUB		ZNF695		
TUBB8		ZNF701		
TUBGCP3		ZNF711		
TWIST2		ZNF740		
TXNDC3		ZNF761		
UBE2NL		ZNF77		
UBE2QP2		ZNF772		
UGT3A2		ZNF775		
UGT8		ZNF788		
ULBP1		ZNF79		
ULBP2		ZNF792		
UNC13A		ZNF813		
UNC5B		ZNF816A		
UNG2		ZNF827		
UPK2		ZNF83		
UPK3B		ZNRF3		
USP19		ZRSR2		
USP28		ZSCAN29		
USP45		ZSWIM3		
USP54		ZSWIM5		
VCX		ZWINT		
VCX3A				
VCX-C				
VDAC2				
VGF				
VGLL1				
VIPR2				
VIT				
VPS13B				
VTCN1				
VTI1A				
WASF1				
WDR17				
WFDC10B				
WFDC2				
WISP1				
WIZ				
WNT10A				
WNT11				
WNT4				
WNT6				
WWTR1				
XAGE2B				
XKR4				
XKR6				
XKRX				
YBX2				
YJEFN3				
YLPM1				
ZBBX				
ZBED2				
ZBTB7C				
ZC3H6				
ZC3H7B				
ZC3HAV1L				

ZCCHC10				
ZCCHC4				
ZDHHC15				
ZDHHC20				
ZFHX2				
ZFP2				
ZFP28				
ZFP42				
ZFP57				
ZFP62				
ZFP64				
ZFP92				
ZIC2				
ZIC3				
ZNF101				
ZNF124				
ZNF138				
ZNF154				
ZNF204				
ZNF215				
ZNF235				
ZNF236				
ZNF286A				
ZNF292				
ZNF300				
ZNF321				
ZNF333				
ZNF345				
ZNF347				
ZNF354A				
ZNF397				
ZNF404				
ZNF420				
ZNF423				
ZNF425				
ZNF429				
ZNF454				
ZNF469				
ZNF488				
ZNF519				
ZNF542				
ZNF551				
ZNF552				
ZNF567				
ZNF570				
ZNF585B				
ZNF587				
ZNF599				
ZNF607				
ZNF610				
ZNF623				
ZNF625				
ZNF630				
ZNF646				
ZNF658				
ZNF660				

ZNF667				
ZNF675				
ZNF687				
ZNF691				
ZNF699				
ZNF702P				
ZNF703				
ZNF708				
ZNF71				
ZNF714				
ZNF720				
ZNF750				
ZNF764				
ZNF765				
ZNF790				
ZNF8				
ZNF831				
ZNF85				
ZNRF2				
ZRANB3				
ZSCAN12				
ZSCAN4				

fetal_liver_expr AND phh_expr	hlc_expr AND fetal_liver_expr AND phh_expr
A2M	A1CF
AAA1	A2LD1
ABCA6	AAAS
ABCB1	AACS
ABCB11	AACSL
ABCC11	AADAC
ABCC6P2	AADACL1
ABCG5	AADAT
ABCG8	AAGAB
ABHD14B	AAK1
ABLIM3	AAMP
ACADSB	AARS
ACMSD	AARS2
ACOT12	AARSD1
ACP5	AASDH
ACSM2A	AASDHPPT
ACSM2B	AATF
ACSM5	AATK
ACVRL1	ABAT
ACY3	ABCA1
ADAMTSL3	ABCA3
ADH1C	ABCA5
ADH4	ABCA7
ADH6	ABCA8
ADORA3	ABCA9
ADRB2	ABCB10
AGTR1	ABCB4
AGXT	ABCB6
AGXT2	ABCB7
AGXT2L1	ABCB9
ALAS2	ABCC2
ALDH3B1	ABCC3
ALDOB	ABCC5
ALKBH3	ABCC6
ALPK1	ABCC6P1
AMBP	ABCC9
AMICA1	ABCD1
ANGPTL3	ABCD3
ANGPTL6	ABCE1
ANXA10	ABCF1
ANXA13	ABCF2
AOC3	ABCF3
APCS	ABCG1
APOA5	ABHD10
APOC2	ABHD12
APOC4	ABHD14A
APOH	ABHD15
AQP12A	ABHD2
ARG1	ABHD3
ARVCF	ABHD4

ASB9	ABHD5
ASH1L	ABHD6
ASPA	ABHD8
ASPG	ABI1
ATF7IP	ABI2
ATP2B2	ABL1
AZGP1	ABLIM1
B3GAT1	ABR
BAAT	ABT1
BBOX1	ABTB1
BCL10	ABTB2
BCL2L10	ACAA1
BCL6B	ACAA2
BDH1	ACACA
BHMT	ACACB
BHMT2	ACAD10
BMX	ACAD11
BTBD11	ACAD8
BTBD16	ACAD9
BTN3A3	ACADL
BZW1	ACADM
C10orf11	ACADS
C10orf116	ACADVL
C10orf68	ACAP2
C10orf72	ACAT1
C14orf105	ACAT2
C14orf68	ACBD3
C14orf73	ACBD6
C15orf52	ACCS
C16orf45	ACD
C16orf50	ACE2
C16orf73	ACER2
C19orf51	ACHE
C1orf168	ACIN1
C1orf51	ACLY
C1QB	ACN9
C1QC	ACO1
C1QL2	ACO2
C1R	ACOT1
C2	ACOT11
C20orf107	ACOT2
C20orf62	ACOT4
C21orf34	ACOT7
C22orf27	ACOT8
C2CD4B	ACOT9
C3	ACOX1
C3orf25	ACOX2
C3orf46	ACOX3
C3orf54	ACP1
C4BPA	ACP2
C4BPB	ACP6
C5orf43	ACPL2
C6	ACSF2
C6orf165	ACSL1
C7orf31	ACSL3
C7orf58	ACSL5

C8A	ACSM3
C8B	ACSS2
C8G	ACSS3
C8ORFK36	ACTA2
C9orf150	ACTB
C9orf24	ACTG1
CA12	ACTL6A
CA5A	ACTN1
CA9	ACTN4
CABP1	ACTR10
CACNA1S	ACTR1A
CACNA2D4	ACTR1B
CAMK1	ACTR2
CAMK1D	ACTR3
CASP1	ACTR3B
CCDC13	ACTR5
CCL14	ACTR6
CCL15	ACTR8
CCL16	ACTRT1
CCL20	ACVR1
CCL3	ACVR1B
CCL3L1	ACVR2A
CCL3L3	ACVR2B
CCL4L1	ACY1
CCL4L2	ACYP1
CD3EAP	ACYP2
CD7	ADA
CD93	ADAL
CDA	ADAM10
CDH20	ADAM15
CDH23	ADAM17
CDK5R2	ADAM19
CDYL2	ADAM9
CER1	ADAMTS1
CES8	ADAMTSL2
CFHR1	ADAMTSL4
CFHR2	ADAMTSL5
CFHR5	ADAP2
CLDN5	ADAR
CLEC14A	ADARB1
CLGN	ADAT1
CLRN3	ADAT3
CNGA1	ADCK1
COL27A1	ADCK2
COX6A2	ADCK4
COX7A1	ADCK5
CP	ADCY6
CPB2	ADCY7
CRP	ADCY9
CRYAA	ADD1
CRYBB2	ADD3
CRYGB	ADH1A
CTSF	ADH5
CTSS	ADHFE1
CUX2	ADI1
CXCL10	ADIPOR1

CYP21A2	ADIPOR2
CYP2D6	ADK
CYP39A1	ADM
CYP3A43	ADM2
CYP3A5	ADNP
CYP4A11	ADNP2
CYP4A22	ADO
CYP4B1	ADORA1
CYP4F12	ADPGK
CYP4F2	ADPRHL2
CYP4F3	ADRA1A
CYP8B1	ADRA1B
DBH	ADRA2A
DDO	ADRA2B
DENND2D	ADRBK1
DEPDC7	ADRM1
DHODH	ADSL
DHRS9	ADSS
DHX58	ADSSL1
DKFZP564J102	AEBP2
DKFZp779M0652	AEN
DMGDH	AES
DNAJC12	AFAP1L1
DNMT3L	AFF1
DOCK10	AFF4
DOK1	AFG3L1
DPYD	AFG3L2
DPYSL5	AFM
DTX1	AFMID
DYRK3	AFP
EBI2	AFTPH
ECM2	AGA
ECSCR	AGAP3
EEPD1	AGAP6
EID3	AGAP8
ELAVL3	AGBL5
ENPP3	AGFG1
ENPP7	AGFG2
EPB41	AGGF1
EPC1	AGK
ESPNL	AGL
EVC2	AGMAT
EVI2B	AGPAT1
F11	AGPAT2
F13B	AGPAT3
F5	AGPAT5
F8	AGPAT6
F9	AGPAT9
FABP1	AGPS
FAM21B	AGRN
FAM21C	AGT
FAM47E	AGTPBP1
FAM50B	AGTRAP
FAM53A	AGXT2L2
FAM78A	AHCTF1
FBXL19	AHCY

FCER1G	AHCYL1
FCN3	AHNAK
FERMT3	AHR
FETUB	AHSA1
FIT1	AHSA2
FLJ20581	AHSG
FLJ35801	AIDA
FMO3	AIF1
FOLH1B	AIF1L
FPGS	AIFM1
FRAT1	AIFM2
FRK	AIG1
G6PC	AIMP2
GAS2	AIP
GBA3	AIRE
GC	AJAP1
GDPD4	AK1
GGT1	AK2
GGT2	AK2P2
GGT3P	AK3
GGTLC2	AK3L1
GIMAP2	AKAP1
GK	AKAP11
GLIPR1	AKAP12
GLTPD2	AKAP13
GLYAT	AKAP7
GLYATL1	AKAP8
GNA14	AKAP8L
GNAT1	AKIRIN1
GNMT	AKIRIN2
GPIHBP1	AKNA
GPLD1	AKR1A1
GPNMB	AKR1B1
GPR109B	AKR1C2
GPR112	AKR1C3
GPR116	AKR1C4
GPR146	AKR1D1
GPT	AKR7A2
GRAP	AKR7A3
GRRP1	AKT1
GSTT2	AKT1S1
GUCA2A	AKTIP
GYS2	ALAD
HAL	ALAS1
HAMP	ALB
HAO1	ALCAM
HAO2	ALDH16A1
HAS1	ALDH18A1
HCK	ALDH1A1
HCLS1	ALDH1A2
HES5	ALDH1B1
HFE2	ALDH1L1
HGD	ALDH2
HGFAC	ALDH3A2
HGSNAT	ALDH4A1
HHEX	ALDH5A1

HIST1H2AE	ALDH6A1
HIST1H2BJ	ALDH7A1
HIST1H3H	ALDH8A1
HIST1H4J	ALDH9A1
HIST2H2AB	ALDOA
HLA-DMB	ALDOC
HLF	ALG1
HMP19	ALG11
HNF4G	ALG12
HOXB1	ALG13
HP	ALG14
HPR	ALG1L
HRG	ALG2
HSD17B6	ALG3
HSFX1	ALG5
HULC	ALG6
ICAM2	ALG8
ICAM4	ALG9
IFI30	ALKBH1
IFI44	ALKBH2
IGFALS	ALKBH4
IKBKAP	ALKBH5
IL1B	ALKBH6
IL1RN	ALKBH7
IL22RA1	ALOX15B
IL6R	ALOX5
ING5	ALOX5AP
INHBB	ALPL
IRF2	ALPP
IRF5	ALS2
ITGAL	ALS2CR14
ITGB7	ALS2CR4
ITIH2	ALX1
ITIH3	AMAC1L3
ITIH4	AMACR
IYD	AMD1
JMJD2A	AMDHD1
JUNB	AMDHD2
KCNA6	AMFR
KENAE	AMMECR1
KGFLP1	AMMECR1L
KIAA0776	AMOT
KIAA1324	AMOTL2
KIAA1881	AMT
KIAA1920	AMY1A
KIF12	AMY1B
KILLIN	AMY1C
KISS1	AMY2A
KLHL33	AMZ2
KLHL35	ANAPC1
KLKB1	ANAPC10
KMO	ANAPC11
KNG1	ANAPC13
KRT126P	ANAPC4
KRT222	ANAPC5
KRT24	ANG

KRT6B	ANGEL1
KRT75	ANGEL2
L3MBTL4	ANGPTL4
LAMA3	ANKFY1
LAPTM5	ANKHD1
LAT2	ANKHD1-EIF4EBP3
LBP	ANKIB1
LCN12	ANKLE2
LCN2	ANKMY1
LDB1	ANKMY2
LDHD	ANKRA2
LEAP-2	ANKRD10
LECT2	ANKRD11
LENG8	ANKRD12
LINCR	ANKRD13A
LMO7	ANKRD13C
LOC100128533	ANKRD13D
LOC100128657	ANKRD16
LOC100129076	ANKRD17
LOC100129191	ANKRD20A1
LOC100129445	ANKRD22
LOC100129463	ANKRD23
LOC100129887	ANKRD24
LOC100130111	ANKRD26
LOC100130179	ANKRD27
LOC100131061	ANKRD28
LOC100131223	ANKRD30B
LOC100131471	ANKRD33
LOC100131512	ANKRD35
LOC100131693	ANKRD36B
LOC100131733	ANKRD37
LOC100132289	ANKRD38
LOC100132346	ANKRD39
LOC100132364	ANKRD40
LOC100132553	ANKRD43
LOC100132560	ANKRD44
LOC100132771	ANKRD46
LOC100132938	ANKRD47
LOC100133511	ANKRD49
LOC100133551	ANKRD50
LOC100133913	ANKRD54
LOC100133972	ANKRD57
LOC100134152	ANKRD6
LOC100134266	ANKRD9
LOC147710	ANKS1A
LOC149351	ANKS4B
LOC283314	ANKZF1
LOC285095	ANO1
LOC285412	ANO10
LOC286310	ANO6
LOC339782	ANO8
LOC387647	ANP32A
LOC387686	ANP32B
LOC388237	ANP32C
LOC389523	ANP32E
LOC391352	ANPEP

LOC392221	ANTXR2
LOC392288	ANXA1
LOC400163	ANXA11
LOC440059	ANXA2
LOC440080	ANXA2P1
LOC440313	ANXA2P2
LOC440503	ANXA2P3
LOC441179	ANXA3
LOC441488	ANXA4
LOC441714	ANXA5
LOC442609	ANXA6
LOC493754	ANXA7
LOC54103	ANXA9
LOC553137	AOF2
LOC554235	AP1B1
LOC55908	AP1G1
LOC641825	AP1G2
LOC641941	AP1M1
LOC641942	AP1S1
LOC642252	AP1S2
LOC642282	AP2A1
LOC642399	AP2A2
LOC642812	AP2B1
LOC643047	AP2M1
LOC643145	AP2S1
LOC643669	AP3B1
LOC643912	AP3D1
LOC643932	AP3M1
LOC643933	AP3S1
LOC644334	AP3S2
LOC644584	AP4B1
LOC645084	AP4E1
LOC645118	APAF1
LOC645128	APBA2BP
LOC645133	APBA3
LOC645217	APBB1IP
LOC645276	APBB3
LOC645284	APEH
LOC645600	APEX1
LOC645897	APEX2
LOC646089	APH1A
LOC646282	APH1B
LOC646330	API5
LOC646452	APIP
LOC646675	APITD1
LOC646845	APLP2
LOC647089	APOA1
LOC647108	APOA1BP
LOC647588	APOA2
LOC647786	APOA4
LOC648213	APOB
LOC648982	APOBEC3B
LOC649396	APOBEC3C
LOC649497	APOBEC3F
LOC649604	APOBEC3G
LOC649917	APOC1

LOC650251	APOC3
LOC650491	APOE
LOC650538	APOF
LOC650681	APOL2
LOC651333	APOL3
LOC651511	APOM
LOC651659	APOO
LOC651979	APOOL
LOC652045	APP
LOC652673	APPBP2
LOC652773	APPL1
LOC652819	APPL2
LOC653066	APRT
LOC653073	APTX
LOC653270	AQP11
LOC653458	AQP12B
LOC653463	AQP3
LOC653498	AQP7P1
LOC653609	AQP7P2
LOC653610	AQR
LOC653879	AR
LOC653968	ARAF
LOC654042	ARAP1
LOC654096	ARAP3
LOC654116	ARCN1
LOC654164	ARD1A
LOC727753	ARF1
LOC727768	ARF3
LOC727869	ARF4
LOC727908	ARF5
LOC728288	ARF6
LOC728290	ARFGAP1
LOC728441	ARFGAP2
LOC728835	ARFGAP3
LOC729905	ARFGEF1
LOC730102	ARFIP1
LOC730243	ARG2
LOC730805	ARGLU1
LOC731074	ARHGAP1
LOC731656	ARHGAP10
LOC731969	ARHGAP11A
LOC85389	ARHGAP12
LOC85390	ARHGAP17
LOC90925	ARHGAP19
LONRF3	ARHGAP21
LPA	ARHGAP23
LPPR1	ARHGAP24
LRRC31	ARHGAP30
LRRK2	ARHGAP4
LY96	ARHGAP9
LYPD2	ARHGDIA
LYPLA2P1	ARHGDIB
LYZ	ARHGEF1
MALL	ARHGEF10
MAMLD1	ARHGEF10L
MAOB	ARHGEF11

MAP1D	ARHGEF12
MAP3K5	ARHGEF18
MAP3K7IP3	ARHGEF19
MAPK11	ARHGEF2
MARCH8	ARHGEF5
MASP2	ARHGEF5L
MAST4	ARHGEF6
MAT1A	ARHGEF7
MBL2	ARID1A
MCCC2	ARID2
MEG3	ARID3A
MIR1224	ARID3B
MIR1267	ARID4A
MIR330	ARID4B
MLKL	ARID5B
MLLT3	ARIH1
MLN	ARIH2
MOGAT3	ARL1
MPL	ARL15
MPV17L	ARL16
MR1	ARL17B
MRGPRF	ARL2
MS4A6A	ARL2BP
MSTP9	ARL3
MT1M	ARL4A
MTM	ARL4D
MUPCDH	ARL5A
MYH14	ARL5B
MYRIP	ARL6IP1
NAGS	ARL6IP4
NALCN	ARL6IP5
NAT8	ARL6IP6
NCF1	ARL8B
NCKAP5	ARMC1
NDUFC2	ARMC10
NFASC	ARMC5
NFIA	ARMC6
NFIC	ARMC7
NFIX	ARMC8
NFS1	ARMCX3
NINJ2	ARMCX5
NLF2	ARMCX6
NMNAT3	ARMET
NPC1L1	ARNT
NPL	ARNTL
NPR3	ARPC1A
NR0B2	ARPC1B
NR1H4	ARPC2
NR1I2	ARPC3
NR1I3	ARPC4
NRTN	ARPC5
NT5E	ARPC5L
NTN4	ARPM1
NUDT16P	ARPP19
NXF3	ARRB1
OBP2B	ARRDC2

ONECUT1	ARRDC3
ONECUT2	ARRDC4
OR2W5	ARS2
OR52N2	ARSA
ORM1	ARSB
ORM2	ARSD
OSGIN1	ARSE
OSTbeta	ARSG
OTC	ART1
OXER1	ART4
OXT	ARV1
P2RX7	ARVP6125
PACRG	AS3MT
PAQR9	ASAH1
PCAF	ASAH2C
PCDH20	ASAP1
PCDH24	ASAP2
PCDHAC2	ASAP3
PCDHGB3	ASB1
PCGF3	ASB13
PCGF5	ASB3
PCSK9	ASB6
PDE12	ASB7
PDE3B	ASB8
PECAM1	ASCC1
PFKFB1	ASCC2
PGBD5	ASCC3
PGLYRP2	ASF1A
PHACS	ASF1B
PIGB	ASGR1
PIK3AP1	ASGR2
PIK3C2G	ASH2L
PION	ASL
PIPOX	ASMTL
PKLR	ASNA1
PLA1A	ASNS
PLA2G1B	ASNSD1
PLA2G4C	ASPM
PLEK	ASPSCR1
PLEKHG1	ASS1
PLG	ASTE1
PLGLB1	ASTN2
PLGLB2	ASXL1
PM20D1	ASXL2
PMCHL1	ATAD1
PNMA3	ATAD2
PNMA6A	ATAD2B
POLR1A	ATAD3A
PON1	ATE1
PON3	ATF1
PPID	ATF2
PPIF	ATF3
PRAMEF17	ATF4
PRAP1	ATF5
PRG2	ATF6
PRG4	ATF6B

PRIM2	ATF7IP2
PRKAR1B	ATG10
PROC	ATG12
PRODH2	ATG16L1
PROP1	ATG16L2
PROZ	ATG2A
PRR22	ATG3
PRR7	ATG4A
PRSS3	ATG4B
PRSS42	ATG4C
PRTN3	ATG4D
PSG9	ATG5
PSMB9	ATG7
PTGER4	ATG9A
PTHLH	ATIC
PTPRH	ATL1
PYCRL	ATL2
RAB26	ATL3
RAB7B	ATM
RAB7L1	ATMIN
RAI2	ATN1
RAMP1	ATOX1
RASGRP3	ATP10A
RBM43	ATP11B
RCP9	ATP11C
RET	ATP13A1
RGR	ATP13A2
RGS1	ATP1A1
RGS14	ATP1A3
RGS3	ATP1B1
RILP	ATP1B3
RINL	ATP2A2
RNF126P1	ATP2B1
RNF207	ATP2B4
RNU86	ATP2C1
RORC	ATP5A1
RPL22L1	ATP5B
RSPH10B	ATP5C1
RTN1	ATP5D
RTN4RL1	ATP5E
RTP2	ATP5EP2
RTP3	ATP5F1
RUNDC3B	ATP5G1
S1PR1	ATP5G2
SAA4	ATP5G3
SAMD5	ATP5H
SAMD9	ATP5I
SARDH	ATP5J
SARM1	ATP5J2
SCARNA17	ATP5L
SCARNA7	ATP5O
SCARNA9L	ATP5S
SCGN	ATP5SL
SCN9A	ATP6AP1
SDCBP2	ATP6AP2
SDR42E1	ATP6V0A1

SEC14L4	ATP6V0A2
SERPINA11	ATP6V0B
SERPINA4	ATP6V0C
SERPINA6	ATP6V0D1
SERPINA7	ATP6V0E1
SERPIND1	ATP6V0E2
SHE	ATP6V1A
SHISA4	ATP6V1B2
SIGLEC14	ATP6V1C1
SKAP1	ATP6V1D
SLAMF9	ATP6V1E1
SLC16A13	ATP6V1F
SLC17A1	ATP6V1G1
SLC17A2	ATP6V1H
SLC17A3	ATP7B
SLC17A4	ATP8B2
SLC17A9	ATP8B4
SLC1A2	ATP9A
SLC22A20	ATP9B
SLC22A25	ATPAF1
SLC22A3	ATPAF2
SLC22A9	ATPBD1B
SLC2A2	ATPBD3
SLC2A6	ATPBD4
SLC2A9	ATPIF1
SLC30A10	ATRIP
SLC38A3	ATRN
SLC38A4	ATXN1
SLC3A1	ATXN10
SLC46A1	ATXN1L
SLC6A1	ATXN2
SLCO1B3	ATXN3
SMOC1	ATXN7L2
SNHG10	ATXN7L3
SNORD100	AUH
SNORD16	AUP1
SNORD32A	AURKAIP1
SNORD56	AUTS2
SNORD96A	AVEN
SNX15	AVL9
SOBP	AVP
SP100	AVPI1
SPAG4L	AVPR2
SPIC	AXIN1
SPON2	AXIN2
SPP2	AXL
SQRDL	AXUD1
SRGN	AYP1p1
SSX4	AZI1
STX11	AZI2
SULT1E1	AZIN1
SYTL4	B2M
TAAR1	B3GALT6
TAF8	B3GAT3
TAF9L	B3GNT1
TAPBPL	B3GNT2

TAT	B3Gn-T6
TBX10	B3GNT6
TBXAS1	B4GALT1
TCF1	B4GALT2
TCF7	B4GALT3
TCP11L2	B4GALT4
TDRD6	B4GALT5
TENC1	B4GALT7
TEPP	B9D1
TESC	B9D2
TFF3	BACE1
TFR2	BACE2
THEM4	BAD
THG1L	BAG2
THPO	BAG3
TIMM17A	BAG4
TLE6	BAG5
TLR8	BAHD1
TMED6	BAIAP2
TMEM110	BAIAP2L1
TMEM139	BAIAP2L2
TMEM151	BAMBI
TMEM176B	BANF1
TMEM195	BANP
TMEM220	BAP1
TMEM233	BASP1
TMEM82	BAT1
TNFRSF14	BAT2
TNFSF11	BAT2D1
TNRC9	BAT2L
TNXA	BAT3
TOR2A	BAT4
TOX3	BAT5
TREML1	BAX
TRIM10	BAZ1A
TRIM15	BAZ1B
TRIM47	BAZ2B
TRIM73	BBC3
TRPS1	BBS1
T-SP1	BBS10
TSPAN8	BBS2
TUBA3E	BBS4
TUBA4A	BBS7
TXNL4A	BBS9
TYMP	BBX
TYROBP	BCAN
UFD1L	BCAP29
UGT2A3	BCAP31
UGT2B10	BCAR1
UGT2B15	BCAR3
UGT2B17	BCAS2
UGT2B4	BCAS4
UPB1	BCAT2
UROC1	BCCIP
USH2A	BCDIN3D
VCX2	BCHE

VMD2L3	BCKDHA
VNN1	BCKDHB
VNN3	BCKDK
VOPP1	BCL2L1
VPREB3	BCL2L11
VSIG4	BCL2L12
VSNL1	BCL2L13
WARS2	BCL2L2
ZDHHC19	BCL3
ZFAT	BCL6
ZFP3	BCL7A
ZIM2	BCL7B
ZMYND12	BCL7C
ZMYND15	BCL9
ZNF385B	BCL9L
ZNF516	BCLAF1
ZNF533	BCOR
ZNF541	BCORL1
ZNF584	BCR
ZNF713	BCS1L
ZNF844	BCYRN1
	BDNF
	BECN1
	BEGAIN
	BEND3
	BEND7
	BET1
	BET1L
	BEX1
	BEX2
	BEXL1
	BFAR
	BGLAP
	BGN
	BHLHB2
	BHLHB9
	BICD2
	BID
	BIN1
	BIN3
	BIRC2
	BIRC3
	BIRC6
	BIVM
	BLCAP
	BLMH
	BLOC1S1
	BLVRA
	BLVRB
	BLZF1
	BMF
	BMI1
	BMP1
	BMP2
	BMP2K
	BMP4

	BMP8B
	BMPR1A
	BMPR2
	BMS1
	BMS1P5
	BNIP1
	BNIP2
	BNIP3
	BNIP3L
	BOAT
	BOK
	BOLA1
	BOLA2
	BOLA3
	BOP1
	BP75
	BPGM
	BPHL
	BPNT1
	BRCA1
	BRCC3
	BRD1
	BRD2
	BRD3
	BRD4
	BRD7
	BRD7P2
	BRD8
	BRD9
	BRE
	BRF1
	BRF2
	BRI3
	BRI3BP
	BRI3P1
	BRIX1
	BRMS1
	BRMS1L
	BRP44
	BRP44L
	BRPF1
	BRPF3
	BRSK2
	BRWD1
	BRWD2
	BSCL2
	BSDC1
	BSG
	BST2
	BTAF1
	BTBD1
	BTBD10
	BTBD12
	BTBD15
	BTBD2
	BTBD3

	BTBD6
	BTBD7
	BTD
	BTF3
	BTF3L4
	BTG1
	BTG2
	BTG3
	BTN2A1
	BTN3A1
	BTN3A2
	BUB3
	BUD13
	BUD31
	BYSL
	BZW2
	C10orf10
	C10orf104
	C10orf114
	C10orf118
	C10orf119
	C10orf12
	C10orf125
	C10orf137
	C10orf140
	C10orf2
	C10orf26
	C10orf28
	C10orf32
	C10orf35
	C10orf4
	C10orf47
	C10orf54
	C10orf57
	C10orf58
	C10orf59
	C10orf6
	C10orf61
	C10orf65
	C10orf75
	C10orf76
	C10orf84
	C11orf1
	C11orf10
	C11orf17
	C11orf2
	C11orf24
	C11orf35
	C11orf46
	C11orf48
	C11orf49
	C11orf51
	C11orf52
	C11orf54
	C11orf57
	C11orf58

	C11orf59
	C11orf60
	C11orf63
	C11orf67
	C11orf68
	C11orf71
	C11orf73
	C11orf74
	C11orf75
	C11orf80
	C11orf83
	C11orf9
	C12orf10
	C12orf11
	C12orf23
	C12orf24
	C12orf26
	C12orf27
	C12orf29
	C12orf30
	C12orf31
	C12orf32
	C12orf35
	C12orf4
	C12orf41
	C12orf43
	C12orf44
	C12orf45
	C12orf47
	C12orf51
	C12orf52
	C12orf57
	C12orf62
	C12orf65
	C12orf66
	C12orf76
	C12orf77
	C13orf1
	C13orf15
	C13orf23
	C13orf25
	C13orf27
	C13orf37
	C13orf7
	C14orf100
	C14orf102
	C14orf104
	C14orf106
	C14orf109
	C14orf112
	C14orf124
	C14orf129
	C14orf131
	C14orf132
	C14orf133
	C14orf135

	C14orf138
	C14orf139
	C14orf142
	C14orf143
	C14orf147
	C14orf149
	C14orf153
	C14orf156
	C14orf159
	C14orf166
	C14orf167
	C14orf169
	C14orf173
	C14orf174
	C14orf179
	C14orf2
	C14orf28
	C14orf32
	C14orf4
	C14orf43
	C14orf45
	C14orf79
	C14orf80
	C14orf82
	C14orf85
	C14orf93
	C15orf17
	C15orf23
	C15orf24
	C15orf29
	C15orf38
	C15orf39
	C15orf40
	C15orf41
	C15orf44
	C15orf57
	C15orf63
	C16orf13
	C16orf33
	C16orf35
	C16orf42
	C16orf48
	C16orf5
	C16orf52
	C16orf53
	C16orf56
	C16orf57
	C16orf58
	C16orf59
	C16orf61
	C16orf63
	C16orf68
	C16orf7
	C16orf70
	C16orf72
	C16orf75

	C16orf80
	C16orf86
	C16orf87
	C16orf91
	C16orf93
	C17orf100
	C17orf101
	C17orf106
	C17orf28
	C17orf37
	C17orf39
	C17orf42
	C17orf45
	C17orf48
	C17orf49
	C17orf51
	C17orf53
	C17orf56
	C17orf58
	C17orf59
	C17orf61
	C17orf62
	C17orf63
	C17orf68
	C17orf70
	C17orf71
	C17orf74
	C17orf79
	C17orf81
	C17orf85
	C17orf88
	C17orf89
	C17orf90
	C17orf91
	C17orf95
	C17orf96
	C17orf97
	C18orf10
	C18orf18
	C18orf19
	C18orf21
	C18orf22
	C18orf25
	C18orf32
	C18orf55
	C18orf56
	C18orf8
	C19orf10
	C19orf12
	C19orf2
	C19orf20
	C19orf22
	C19orf24
	C19orf25
	C19orf28
	C19orf29

	C19orf30
	C19orf31
	C19orf40
	C19orf42
	C19orf43
	C19orf44
	C19orf46
	C19orf47
	C19orf48
	C19orf50
	C19orf52
	C19orf53
	C19orf54
	C19orf56
	C19orf6
	C19orf60
	C19orf61
	C19orf62
	C19orf63
	C19orf64
	C19orf66
	C19orf70
	C1D
	C1GALT1
	C1GALT1C1
	C1orf104
	C1orf107
	C1orf109
	C1orf112
	C1orf115
	C1orf122
	C1orf123
	C1orf124
	C1orf128
	C1orf130
	C1orf131
	C1orf144
	C1orf149
	C1orf151
	C1orf159
	C1orf162
	C1orf163
	C1orf166
	C1orf172
	C1orf174
	C1orf177
	C1orf188
	C1orf19
	C1orf198
	C1orf201
	C1orf203
	C1orf210
	C1orf212
	C1orf216
	C1orf218
	C1orf25

	C1orf31
	C1orf35
	C1orf41
	C1orf43
	C1orf50
	C1orf52
	C1orf53
	C1orf54
	C1orf55
	C1orf56
	C1orf57
	C1orf63
	C1orf66
	C1orf69
	C1orf71
	C1orf74
	C1orf77
	C1orf83
	C1orf85
	C1orf86
	C1orf9
	C1orf91
	C1orf93
	C1orf97
	C1QA
	C1QBP
	C1QTNF6
	C1RL
	C1S
	C20orf100
	C20orf108
	C20orf11
	C20orf111
	C20orf127
	C20orf177
	C20orf191
	C20orf199
	C20orf20
	C20orf24
	C20orf27
	C20orf29
	C20orf3
	C20orf30
	C20orf4
	C20orf43
	C20orf45
	C20orf46
	C20orf52
	C20orf55
	C20orf56
	C20orf7
	C20orf72
	C21orf119
	C21orf124
	C21orf126
	C21orf2

	C21orf24
	C21orf30
	C21orf33
	C21orf37
	C21orf45
	C21orf51
	C21orf55
	C21orf57
	C21orf58
	C21orf59
	C21orf66
	C21orf7
	C21orf70
	C21orf91
	C22orf13
	C22orf25
	C22orf28
	C22orf29
	C22orf30
	C22orf32
	C22orf36
	C22orf39
	C22orf40
	C22orf9
	C2CD2
	C2orf18
	C2orf24
	C2orf25
	C2orf28
	C2orf29
	C2orf30
	C2orf34
	C2orf42
	C2orf43
	C2orf44
	C2orf47
	C2orf49
	C2orf56
	C2orf64
	C2orf68
	C2orf69
	C2orf7
	C2orf76
	C2orf79
	C2orf82
	C3orf1
	C3orf10
	C3orf14
	C3orf17
	C3orf19
	C3orf21
	C3orf23
	C3orf26
	C3orf31
	C3orf34
	C3orf36

	C3orf37
	C3orf38
	C3orf39
	C3orf50
	C3orf58
	C3orf59
	C3orf60
	C3orf63
	C3orf64
	C3orf75
	C4orf14
	C4orf19
	C4orf23
	C4orf27
	C4orf32
	C4orf33
	C4orf34
	C4orf41
	C4orf46
	C4orf48
	C5
	C5orf13
	C5orf15
	C5orf21
	C5orf22
	C5orf24
	C5orf25
	C5orf28
	C5orf30
	C5orf32
	C5orf33
	C5orf35
	C5orf37
	C5orf4
	C5orf41
	C5orf44
	C5orf5
	C5orf51
	C6orf106
	C6orf108
	C6orf111
	C6orf115
	C6orf120
	C6orf125
	C6orf129
	C6orf130
	C6orf136
	C6orf138
	C6orf145
	C6orf153
	C6orf160
	C6orf162
	C6orf170
	C6orf173
	C6orf192
	C6orf203

	C6orf211
	C6orf225
	C6orf47
	C6orf48
	C6orf57
	C6orf61
	C6orf62
	C6orf64
	C6orf66
	C6orf70
	C6orf72
	C6orf85
	C6orf89
	C7orf10
	C7orf11
	C7orf20
	C7orf23
	C7orf25
	C7orf26
	C7orf27
	C7orf28A
	C7orf28B
	C7orf29
	C7orf30
	C7orf36
	C7orf38
	C7orf40
	C7orf41
	C7orf42
	C7orf43
	C7orf44
	C7orf47
	C7orf49
	C7orf50
	C7orf54
	C7orf55
	C7orf59
	C7orf63
	C7orf68
	C7orf70
	C8orf33
	C8orf37
	C8orf38
	C8orf4
	C8orf40
	C8orf41
	C8orf42
	C8orf45
	C8orf47
	C8orf48
	C8orf55
	C8orf59
	C8orf76
	C8orf83
	C9orf102
	C9orf103

	C9orf10OS
	C9orf114
	C9orf116
	C9orf119
	C9orf123
	C9orf127
	C9orf130
	C9orf142
	C9orf156
	C9orf16
	C9orf169
	C9orf21
	C9orf23
	C9orf25
	C9orf3
	C9orf30
	C9orf37
	C9orf40
	C9orf45
	C9orf46
	C9orf5
	C9orf6
	C9orf64
	C9orf69
	C9orf7
	C9orf72
	C9orf75
	C9orf78
	C9orf80
	C9orf82
	C9orf85
	C9orf86
	C9orf89
	C9orf9
	C9orf90
	C9orf91
	C9orf95
	CA13
	CA2
	CA4
	CA5B
	CAB39
	CAB39L
	CABC1
	CABIN1
	CABLES1
	CABLES2
	CACHD1
	CACNA1H
	CACNG1
	CACYBP
	CAD
	CADM1
	CADM4
	CADPS2
	CALB2

	CALCOCO1
	CALCOCO2
	CALD1
	CALHM2
	CALM1
	CALM2
	CALM3
	CALML4
	CALN1
	CALR
	CALU
	CAMK2G
	CAMK2N1
	CAMKK2
	CAMLG
	CAMSAP1
	CAMSAP1L1
	CAND1
	CAND2
	CANT1
	CANX
	CAP1
	CAP2
	CAPG
	CAPN1
	CAPN12
	CAPN2
	CAPN3
	CAPN5
	CAPN6
	CAPN7
	CAPNS1
	CAPRIN1
	CAPRIN2
	CAPS
	CAPS2
	CAPZA1
	CAPZA2
	CAPZB
	CARD10
	CARD11
	CARD14
	CARD8
	CARHSP1
	CARKD
	CARM1
	CARS
	CARS2
	CASC3
	CASC4
	CASD1
	CASK
	CASKIN2
	CASP2
	CASP3
	CASP4

	CASP6
	CASP7
	CASP9
	CAST
	CASZ1
	CAT
	CATSPER2
	CATSPER2P1
	CAV1
	CAV2
	CBARA1
	CBFA2T2
	CBFB
	CBL
	CBLB
	CBLL1
	CBLN3
	CBR1
	CBR3
	CBR4
	CBS
	CBWD1
	CBWD3
	CBWD5
	CBX1
	CBX2
	CBX3
	CBX4
	CBX5
	CBX6
	CBX7
	CBY1
	CC2D1A
	CC2D1B
	CCAR1
	CCBE1
	CCBL1
	CCBL2
	CCBP2
	CCDC101
	CCDC102A
	CCDC104
	CCDC106
	CCDC107
	CCDC109A
	CCDC109B
	CCDC112
	CCDC115
	CCDC117
	CCDC12
	CCDC120
	CCDC123
	CCDC124
	CCDC125
	CCDC127
	CCDC128

	CCDC130
	CCDC132
	CCDC135
	CCDC14
	CCDC16
	CCDC18
	CCDC21
	CCDC22
	CCDC23
	CCDC24
	CCDC25
	CCDC28A
	CCDC28B
	CCDC34
	CCDC43
	CCDC45
	CCDC46
	CCDC47
	CCDC49
	CCDC5
	CCDC50
	CCDC51
	CCDC53
	CCDC55
	CCDC56
	CCDC58
	CCDC59
	CCDC6
	CCDC68
	CCDC69
	CCDC71
	CCDC72
	CCDC76
	CCDC77
	CCDC84
	CCDC85B
	CCDC86
	CCDC88C
	CCDC90A
	CCDC90B
	CCDC91
	CCDC92
	CCDC93
	CCDC94
	CCDC97
	CCHCR1
	CCL2
	CCL5
	CCM2
	CCNB1IP1
	CCNC
	CCND1
	CCND2
	CCND3
	CCNDBP1
	CCNE1

	CCNE2
	CCNF
	CCNG1
	CCNG2
	CCNH
	CCNI
	CCNI2
	CCNK
	CCNL1
	CCNL2
	CCNT1
	CCNT2
	CCNY
	CCNYL1
	CCPG1
	CCR6
	CCRK
	CCRN4L
	CCS
	CCT2
	CCT3
	CCT4
	CCT5
	CCT6A
	CCT6B
	CCT6P1
	CCT7
	CCT8
	CD14
	CD151
	CD163
	CD164
	CD1D
	CD24
	CD248
	CD276
	CD2AP
	CD2BP2
	CD302
	CD320
	CD34
	CD44
	CD46
	CD47
	CD52
	CD55
	CD58
	CD59
	CD63
	CD68
	CD74
	CD79A
	CD79B
	CD81
	CD82
	CD83

	CD84
	CD86
	CD9
	CD97
	CD99
	CD99L2
	CDAN1
	CDC123
	CDC14A
	CDC14B
	CDC16
	CDC23
	CDC25A
	CDC25B
	CDC25C
	CDC26
	CDC2L1
	CDC2L2
	CDC2L5
	CDC2L6
	CDC34
	CDC37
	CDC40
	CDC42
	CDC42BPA
	CDC42BPB
	CDC42EP1
	CDC42EP2
	CDC42EP4
	CDC42SE1
	CDC42SE2
	CDC5L
	CDCA4
	CDCA7L
	CDH1
	CDH2
	CDH24
	CDIPT
	CDK10
	CDK2
	CDK2AP1
	CDK2AP2
	CDK4
	CDK5
	CDK5R1
	CDK5RAP1
	CDK5RAP2
	CDK5RAP3
	CDK6
	CDK7
	CDK9
	CDKL3
	CDKN1A
	CDKN1B
	CDKN1C
	CDKN2AIP

	CDKN2AIPNL
	CDKN2B
	CDKN2D
	CDNF
	CDO1
	CDR2
	CDR2L
	CDRT4
	CDS2
	CDV3
	CDYL
	CEACAM1
	CEBPA
	CEBPB
	CEBPD
	CEBPG
	CEBPZ
	CECR1
	CECR5
	CECR7
	CELSR2
	CELSR3
	CENPB
	CENPBD1
	CENPC1
	CENPJ
	CENPL
	CENPN
	CENPT
	CENPV
	CENTA1
	CENTB2
	CENTD2
	CENTG2
	CENTG3
	CEP164
	CEP192
	CEP27
	CEP350
	CEP57
	CEP63
	CEP70
	CEP72
	CEP78
	CEPT1
	CERK
	CES1
	CES2
	CES3
	CETN2
	CETN3
	CFB
	CFD
	CFDP1
	CFH
	CFI

	CFL1
	CFL2
	CFLAR
	CGGBP1
	CGI-96
	CGN
	CGNL1
	CGRRF1
	CHCHD1
	CHCHD10
	CHCHD2
	CHCHD3
	CHCHD4
	CHCHD5
	CHCHD6
	CHCHD7
	CHCHD8
	CHCHD9
	CHD1
	CHD1L
	CHD2
	CHD4
	CHD6
	CHD7
	CHD8
	CHD9
	CHDH
	CHEK1
	CHERP
	CHES1
	CHFR
	CHI3L1
	CHIC2
	CHKA
	CHKB
	CHM
	CHML
	CHMP1A
	CHMP1B
	CHMP2A
	CHMP2B
	CHMP4A
	CHMP4B
	CHMP4C
	CHMP5
	CHMP6
	CHMP7
	CHN2
	CHORDC1
	CHP
	CHPF
	CHPF2
	CHPT1
	CHRAC1
	CHRD
	CHRNA2

	CHRNA5
	CHRNB1
	CHST1
	CHST12
	CHST13
	CHST14
	CHST15
	CHST3
	CHST7
	CHSY1
	CHTF8
	CHUK
	CHURC1
	CIAO1
	CIAPIN1
	CIB1
	CIC
	CICE
	CICK0721Q.1
	CIDEB
	CIDEC
	CIDECP
	CINP
	CIP29
	CIR1
	CIRBP
	CIRH1A
	CISD1
	CISD2
	CISD3
	CITED2
	CITED4
	CKAP2
	CKAP4
	CKAP5
	CKB
	CKLF
	CKS1B
	CKS2
	CLASP1
	CLCC1
	CLCN3
	CLCN5
	CLCN6
	CLCN7
	CLDN1
	CLDN10
	CLDN12
	CLDN14
	CLDN15
	CLDN23
	CLDN3
	CLDN4
	CLDN7
	CLDN9
	CLDND1

	CLDND2
	CLEC16A
	CLEC2D
	CLIC1
	CLIC4
	CLINT1
	CLIP1
	CLIP2
	CLK1
	CLK2
	CLK3
	CLK4
	CLMN
	CLN3
	CLN5
	CLN6
	CLN8
	CLNS1A
	CLOCK
	CLP1
	CLPP
	CLPTM1
	CLPTM1L
	CLPX
	CLRN1
	CLSTN1
	CLTA
	CLTB
	CLTC
	CLUAP1
	CLYBL
	CMAS
	CMBL
	CMC1
	CMIP
	CMPK1
	CMTM3
	CMTM4
	CMTM6
	CMTM7
	CMTM8
	CMYA5
	CNBP
	CNDP2
	CNIH
	CNIH4
	CNKSR1
	CNKSR3
	CNN2
	CNN3
	CNNM2
	CNNM3
	CNO
	CNOT1
	CNOT10
	CNOT2

	CNOT3
	CNOT4
	CNOT6
	CNOT6L
	CNOT7
	CNOT8
	CNPY2
	CNPY3
	CNPY4
	CNTLN
	CNTNAP5
	COASY
	COBL
	COBLL1
	COBRA1
	COG1
	COG2
	COG3
	COG4
	COG5
	COG6
	COG7
	COG8
	COIL
	COL16A1
	COL18A1
	COL1A1
	COL1A2
	COL3A1
	COL4A1
	COL4A2
	COL4A3BP
	COL5A1
	COL5A2
	COL6A1
	COL6A2
	COL6A3
	COL7A1
	COL8A2
	COLEC11
	COLQ
	COMMD1
	COMMD10
	COMMD2
	COMMD3
	COMMD4
	COMMD5
	COMMD6
	COMMD7
	COMMD8
	COMMD9
	COMT
	COMTD1
	COPA
	COPB1
	COPB2

	COPE
	COPG
	COPG2
	COPG2IT1
	COPS2
	COPS3
	COPS4
	COPS5
	COPS6
	COPS7A
	COPS7B
	COPS8
	COPZ1
	COQ10A
	COQ10B
	COQ2
	COQ3
	COQ4
	COQ5
	COQ6
	COQ7
	COQ9
	CORO1B
	CORO1C
	CORO2A
	CORO7
	COTL1
	COX10
	COX15
	COX17
	COX19
	COX4I1
	COX4NB
	COX5A
	COX5B
	COX6A1
	COX6B1
	COX6C
	COX7A2
	COX7A2L
	COX7B
	COX7C
	COX8A
	CP110
	CPD
	CPE
	CPEB2
	CPEB3
	CPEB4
	CPM
	CPN1
	CPN2
	CPNE1
	CPNE3
	CPNE8
	CPOX

	CPPED1
	CPS1
	CPSF1
	CPSF2
	CPSF3
	CPSF3L
	CPSF4
	CPT1A
	CPT1B
	CPT2
	CPVL
	CRABP2
	CRADD
	CRAMP1L
	CRAT
	CRB3
	CRBN
	CRCP
	CREB1
	CREB3
	CREB3L2
	CREB3L3
	CREB3L4
	CREB5
	CREBBP
	CREBL1
	CREBL2
	CREBZF
	CREG1
	CRELD1
	CRELD2
	CRHR1
	CRIP1
	CRIP2
	CRIPAK
	CRIPT
	CRK
	CRKL
	CRKRS
	CRLF3
	CRLS1
	CRMP1
	CROP
	CROT
	CRSP9
	CRTAP
	CRTC2
	CRTC3
	CRY1
	CRY2
	CRYAB
	CRYL1
	CRYM
	CRYZ
	CRYZL1
	CS

	CSAD
	CSDA
	CSDE1
	CSE1L
	CSF1R
	CSF2RA
	CSHL1
	CSK
	CSNK1A1
	CSNK1D
	CSNK1E
	CSNK1G1
	CSNK1G2
	CSNK1G3
	CSNK2A1
	CSNK2A1P
	CSNK2A2
	CSNK2B
	CSPP1
	CSRNP2
	CSRP1
	CSRP2
	CSRP2BP
	CST3
	CSTB
	CSTF2
	CSTF2T
	CSTF3
	CTAGE6
	CTBP1
	CTBP2
	CTBS
	CTCF
	CTDP1
	CTDSP1
	CTDSP2
	CTDSPL
	CTDSPL2
	CTGF
	CTGLF3
	CTGLF7
	CTH
	CTHRC1
	CTNNA1
	CTNNAL1
	CTNNB1
	CTNNBIP1
	CTNNBL1
	CTNND1
	CTNS
	CTPS
	CTPS2
	CTR9
	CTSA
	CTSB
	CTSC

	CTSD
	CTSH
	CTSL1
	CTSO
	CTSZ
	CTTN
	CTU2
	CTXN1
	CUEDC1
	CUEDC2
	CUGBP1
	CUL1
	CUL2
	CUL4A
	CUL4B
	CUL5
	CUL9
	CUTA
	CUTC
	CUTL1
	CWC15
	CWC22
	CWF19L1
	CWF19L2
	CX3CL1
	CXADR
	CXCL1
	CXCL12
	CXCL16
	CXCR7
	CXorf12
	CXorf26
	CXorf36
	CXorf38
	CXorf39
	CXorf40A
	CXorf40B
	CXorf57
	CXorf64
	CXXC1
	CXXC5
	CYB561
	CYB561D1
	CYB561D2
	CYB5A
	CYB5B
	CYB5D1
	CYB5D2
	CYB5R1
	CYB5R2
	CYB5R3
	CYB5R4
	CYBA
	CYBASC3
	CYBRD1
	CYC1

	CYCS
	CYCSL1
	CYFIP1
	CYFIP2
	CYGB
	CYHR1
	CYLD
	CYLN2
	CYorf14
	CYorf15A
	CYP11A1
	CYP20A1
	CYP26B1
	CYP27A1
	CYP2C8
	CYP2J2
	CYP2R1
	CYP2U1
	CYP3A7
	CYP4V2
	CYP51A1
	CYR61
	CYTH1
	CYTH2
	CYTH3
	CYTSA
	D2HGDH
	DAAM1
	DAAM2
	DAB2
	DACT2
	DAD1
	DAG1
	DAGLB
	DAK
	DAP
	DAP3
	DAPK1
	DAPK3
	DAPP1
	DARS
	DARS2
	DAXX
	DAZAP1
	DAZAP2
	DBI
	DBN1
	DBNDD1
	DBNDD2
	DBNL
	DBP
	DBR1
	DBT
	DCAF10
	DCAF15
	DCAF16

	DCAF6
	DCAF7
	DCAKD
	DCBLD1
	DCBLD2
	DCI
	DCK
	DCLRE1A
	DCLRE1C
	DCN
	DCP1A
	DCP1B
	DCP2
	DCPS
	DCTD
	DCTN1
	DCTN2
	DCTN3
	DCTN4
	DCTN5
	DCTN6
	DCTPP1
	DCUN1D1
	DCUN1D3
	DCUN1D4
	DCUN1D5
	DCXR
	DDA1
	DDAH1
	DDAH2
	DDB1
	DDB2
	DDC
	DDEF2
	DDHD2
	DDIT3
	DDIT4
	DDOST
	DDR1
	DDRGK1
	DDT
	DDX1
	DDX10
	DDX12
	DDX17
	DDX18
	DDX19A
	DDX19B
	DDX19-DDX19L
	DDX21
	DDX23
	DDX24
	DDX27
	DDX28
	DDX31
	DDX39

	DDX3X
	DDX41
	DDX42
	DDX46
	DDX47
	DDX49
	DDX5
	DDX50
	DDX51
	DDX52
	DDX54
	DDX55
	DDX56
	DDX58
	DDX59
	DDX60
	DEAF1
	DECR1
	DECR2
	DEDD
	DEDD2
	DEF6
	DEF8
	DEFB1
	DEFB123
	DEGS1
	DEK
	DEM1
	DENND1A
	DENND2C
	DENND4A
	DENND4B
	DENND4C
	DENND5A
	DENND5B
	DENR
	DEPDC5
	DEPDC6
	DERA
	DERL1
	DERL2
	DET1
	DEXI
	DFFA
	DFFB
	DFNA5
	DFNB59
	DGAT1
	DGAT2
	DGCR11
	DGCR14
	DGCR2
	DGCR6
	DGCR6L
	DGCR8
	DGKD

	DGKQ
	DGUOK
	DHCR24
	DHCR7
	DHDDS
	DHFRL1
	DHPS
	DHRS1
	DHRS11
	DHRS12
	DHRS13
	DHRS3
	DHRS4
	DHRS4L1
	DHRS4L2
	DHRS7
	DHRS7B
	DHRSX
	DHTKD1
	DHX15
	DHX16
	DHX29
	DHX30
	DHX32
	DHX33
	DHX34
	DHX35
	DHX36
	DHX37
	DHX38
	DHX40
	DHX8
	DHX9
	DIABLO
	DIAPH1
	DIAPH2
	DICER1
	DIDO1
	DIMT1L
	DIO1
	DIO2
	DIP2A
	DIP2B
	DIP2C
	DIRC2
	DIS3L
	DIS3L2
	DISP1
	DIXDC1
	dJ341D10.1
	DKC1
	DKFZp434K191
	DKFZp434M131
	DKFZp434N035
	DKFZP586I1420
	DKFZp686O24166

	DKFZp761P0423
	DKFZP779L1853
	DKK1
	DKK3
	DLAT
	DLD
	DLEU1
	DLEU2
	DLG1
	DLG5
	DLGAP4
	DLK2
	DLST
	DMAP1
	DMC1
	DMKN
	DMTF1
	DMWD
	DMXL1
	DNA2
	DNAH1
	DNAJA1
	DNAJA2
	DNAJA3
	DNAJB1
	DNAJB11
	DNAJB12
	DNAJB14
	DNAJB2
	DNAJB4
	DNAJB5
	DNAJB6
	DNAJB9
	DNAJC1
	DNAJC10
	DNAJC13
	DNAJC14
	DNAJC15
	DNAJC17
	DNAJC19
	DNAJC21
	DNAJC22
	DNAJC24
	DNAJC25
	DNAJC27
	DNAJC3
	DNAJC30
	DNAJC4
	DNAJC5
	DNAJC7
	DNAJC8
	DNAJC9
	DNAL1
	DNAL4
	DNALI1
	DNASE1L1

	DNASE2
	DNCL1
	DNLZ
	DNM1L
	DNM2
	DNMT1
	DNPEP
	DNTTIP1
	DOCK1
	DOCK2
	DOCK3
	DOCK6
	DOCK7
	DOHH
	DOK4
	DOLK
	DOLPP1
	DOM3Z
	DONSON
	DOPEY2
	DOT1L
	DPAGT1
	DPF2
	DPH2
	DPH3
	DPH5
	DPM1
	DPM2
	DPM3
	DPP3
	DPP4
	DPP7
	DPP8
	DPP9
	DPY19L1
	DPY19L4
	DPY30
	DPYS
	DPYSL2
	DR1
	DRAM1
	DRAP1
	DRD4
	DRG1
	DRG2
	DSC2
	DSCAM
	DSCC1
	DSCR3
	DSE
	DSG2
	DSN1
	DSP
	DST
	DSTN
	DSTYK

	DTD1
	DTNA
	DTNBP1
	DTWD1
	DTWD2
	DTX2
	DTX3L
	DULLARD
	DUS1L
	DUS2L
	DUS3L
	DUS4L
	DUSP1
	DUSP10
	DUSP11
	DUSP12
	DUSP13
	DUSP14
	DUSP15
	DUSP16
	DUSP18
	DUSP19
	DUSP2
	DUSP22
	DUSP23
	DUSP28
	DUSP3
	DUSP5
	DUSP6
	DUSP8
	DUT
	DUXAP3
	DVL2
	DVL3
	DYDC2
	DYM
	DYNC1H1
	DYNC1I2
	DYNC1LI2
	DYNC2H1
	DYNC2LI1
	DYNLL1
	DYNLL2
	DYNLRB1
	DYNLT1
	DYNLT3
	DYRK1A
	DYRK1B
	DYRK2
	DYRK4
	DYSF
	E2F1
	E2F3
	E2F4
	E2F5
	E2F6

	E4F1
	EAF1
	EAF2
	EAPP
	EARS2
	EBAG9
	EBF4
	EBNA1BP2
	EBP
	EBPL
	ECD
	ECE2
	ECGF1
	ECH1
	ECHDC1
	ECHDC2
	ECHDC3
	ECHS1
	ECM1
	ECOP
	ECSIT
	ECT2
	EDARADD
	EDC3
	EDC4
	EDEM1
	EDEM2
	EDEM3
	EDF1
	EDG1
	EDG4
	EED
	EEF1A1
	EEF1AL7
	EEF1B2
	EEF1D
	EEF1E1
	EEF1G
	EEF2
	EEF2K
	EFCAB4A
	EFEMP2
	EFHA1
	EFHD1
	EFHD2
	EFNA1
	EFNA4
	EFNB1
	EFR3A
	EFR3B
	EFTUD1
	EFTUD2
	EGFL7
	EGFR
	EGLN1
	EGLN2

	EGOT
	EGR1
	EHBP1
	EHBP1L1
	EHD1
	EHD2
	EHD4
	EHHADH
	EHMT2
	EI24
	EID1
	EID2
	EID2B
	EIF1
	EIF1AD
	EIF1AX
	EIF1AY
	EIF1B
	EIF2A
	EIF2AK1
	EIF2AK2
	EIF2AK3
	EIF2AK4
	EIF2B1
	EIF2B2
	EIF2B3
	EIF2B4
	EIF2B5
	EIF2C1
	EIF2C2
	EIF2C3
	EIF2S1
	EIF2S2
	EIF2S3
	EIF3A
	EIF3B
	EIF3C
	EIF3D
	EIF3E
	EIF3F
	EIF3G
	EIF3H
	EIF3I
	EIF3J
	EIF3K
	EIF3L
	EIF3M
	EIF4A1
	EIF4A2
	EIF4A3
	EIF4B
	EIF4E
	EIF4E2
	EIF4E3
	EIF4EBP1
	EIF4EBP2

	EIF4EBP3
	EIF4ENIF1
	EIF4G1
	EIF4G2
	EIF4G3
	EIF4H
	EIF5
	EIF5A
	EIF5B
	EIF6
	ELAC1
	ELAC2
	ELAVL1
	ELF1
	ELF2
	ELF3
	ELF4
	ELF5
	ELK1
	ELL
	ELL2
	ELL3
	ELMO1
	ELMO2
	ELMO3
	ELMOD2
	ELMOD3
	ELOF1
	ELOVL1
	ELOVL2
	ELOVL5
	ELOVL6
	ELP2
	ELP3
	ELP4
	EMD
	EMG1
	EML2
	EML3
	EML4
	EMP1
	EMP3
	EMX2OS
	ENC1
	ENDOG
	ENHO
	ENO1
	ENO3
	ENOPH1
	ENOSF1
	ENOX2
	ENPEP
	ENPP1
	ENPP4
	ENSA
	ENTPD3

	ENTPD4
	ENTPD5
	ENTPD6
	ENTPD7
	ENY2
	EP300
	EP400
	EPAS1
	EPB41L1
	EPB41L2
	EPB41L4A
	EPB41L4B
	EPB41L5
	EPB49
	EPCAM
	EPDR1
	EPHA1
	EPHA10
	EPHA2
	EPHA8
	EPHB4
	EPHX1
	EPHX2
	EPM2A
	EPM2AIP1
	EPN1
	EPN2
	EPO
	EPOR
	EPPB9
	EPRS
	EPS15
	EPS15L1
	EPS8
	EPSTI1
	ERAL1
	ERAP2
	ERBB2
	ERBB3
	ERC1
	ERCC1
	ERCC2
	ERCC3
	ERCC5
	ERCC8
	ERF
	ERGIC1
	ERGIC2
	ERGIC3
	ERH
	ERI1
	ERI2
	ERI3
	ERICH1
	ERLIN1
	ERLIN2

	ERMAP
	ERMP1
	ERN1
	ERO1L
	ERP29
	ERRFI1
	ESAM
	ESCO1
	ESD
	ESPL1
	ESPN
	ESRRA
	ESRRAP2
	ESYT1
	ETF1
	ETFA
	ETFB
	ETFDH
	ETHE1
	ETNK1
	ETNK2
	ETS1
	ETS2
	ETV5
	ETV6
	EVI5
	EVI5L
	EVL
	EWSR1
	EXD2
	EXD3
	EXOC1
	EXOC2
	EXOC3
	EXOC4
	EXOC5
	EXOC6
	EXOC7
	EXOSC1
	EXOSC10
	EXOSC2
	EXOSC3
	EXOSC4
	EXOSC5
	EXOSC6
	EXOSC7
	EXOSC8
	EXOSC9
	EXT1
	EXT2
	EXTL2
	EXTL3
	EYA3
	EYS
	EZH2
	EZR

	F10
	F11R
	F12
	F2
	F2R
	F3
	F7
	F8A1
	FAAH
	FAAH2
	FABP5
	FABP5L2
	FADD
	FADS1
	FADS2
	FADS3
	FAF1
	FAF2
	FAH
	FAHD1
	FAHD2A
	FAHD2B
	FAIM
	FAM100A
	FAM100B
	FAM102A
	FAM102B
	FAM103A1
	FAM104A
	FAM104B
	FAM105A
	FAM105B
	FAM107B
	FAM108A2
	FAM108A3
	FAM108B1
	FAM108C1
	FAM109A
	FAM10A4
	FAM10A7
	FAM110A
	FAM110B
	FAM111A
	FAM113A
	FAM113B
	FAM114A2
	FAM115A
	FAM116A
	FAM116B
	FAM117A
	FAM117B
	FAM118A
	FAM118B
	FAM119A
	FAM119B
	FAM120A

	FAM120AOS
	FAM120B
	FAM122A
	FAM122B
	FAM125A
	FAM125B
	FAM126B
	FAM127A
	FAM127B
	FAM127C
	FAM128A
	FAM129B
	FAM131A
	FAM133B
	FAM134A
	FAM134B
	FAM134C
	FAM136A
	FAM136B
	FAM13A
	FAM13B
	FAM149A
	FAM149B1
	FAM14B
	FAM151A
	FAM153B
	FAM156A
	FAM156B
	FAM158A
	FAM160A2
	FAM160B1
	FAM160B2
	FAM162A
	FAM164C
	FAM165B
	FAM168B
	FAM171A1
	FAM172A
	FAM173A
	FAM173B
	FAM174A
	FAM174B
	FAM175A
	FAM175B
	FAM176A
	FAM176B
	FAM177A1
	FAM178A
	FAM179B
	FAM184A
	FAM188A
	FAM188B
	FAM189B
	FAM18B
	FAM18B2
	FAM190B

	FAM193A
	FAM193B
	FAM195A
	FAM195B
	FAM20A
	FAM20B
	FAM20C
	FAM21D
	FAM24B
	FAM26E
	FAM30A
	FAM32A
	FAM35A
	FAM36A
	FAM38A
	FAM38B
	FAM39DP
	FAM39E
	FAM3A
	FAM3C
	FAM40A
	FAM40B
	FAM43A
	FAM44B
	FAM45A
	FAM46A
	FAM46C
	FAM48A
	FAM49B
	FAM50A
	FAM53B
	FAM53C
	FAM54B
	FAM57A
	FAM58A
	FAM60A
	FAM62B
	FAM63A
	FAM65A
	FAM69A
	FAM69B
	FAM71E1
	FAM72B
	FAM73A
	FAM73B
	FAM75B
	FAM75C1
	FAM76B
	FAM82A2
	FAM83D
	FAM83F
	FAM83H
	FAM84B
	FAM86A
	FAM86B1
	FAM86C

	FAM86D
	FAM89A
	FAM89B
	FAM8A1
	FAM90A3
	FAM91A1
	FAM92A1
	FAM96A
	FAM96B
	FAM98A
	FAM98C
	FANCC
	FANCE
	FANCG
	FANCL
	FARP1
	FARP2
	FARS2
	FARSA
	FARSLB
	FASN
	FASTK
	FASTKD1
	FASTKD2
	FASTKD3
	FASTKD5
	FAT1
	FAU
	FBL
	FBLN5
	FBLN7
	FBP1
	FBRS
	FBS1
	FBXL10
	FBXL11
	FBXL12
	FBXL15
	FBXL18
	FBXL20
	FBXL3
	FBXL5
	FBXL6
	FBXO11
	FBXO17
	FBXO18
	FBXO2
	FBXO21
	FBXO22
	FBXO28
	FBXO3
	FBXO30
	FBXO31
	FBXO32
	FBXO33
	FBXO34

	FBXO38
	FBXO4
	FBXO42
	FBXO44
	FBXO46
	FBXO5
	FBXO6
	FBXO7
	FBXO8
	FBXW11
	FBXW2
	FBXW4
	FBXW5
	FBXW7
	FBXW8
	FBXW9
	FCAR
	FCF1
	FCGR2A
	FCGR2B
	FCGR3A
	FCGR3B
	FCGRT
	FCHO2
	FCHSD2
	FCRLB
	FDFT1
	FDPS
	FDX1
	FDX1L
	FECH
	FEM1A
	FEM1C
	FEN1
	FER1L4
	FERMT2
	FES
	FEZ1
	FEZ2
	FGA
	FGB
	FGD2
	FGF18
	FGF23
	FGFR1OP
	FGFR1OP2
	FGFR3
	FGFR4
	FGFRL1
	FGG
	FGGY
	FGL1
	FGL2
	FGR
	FH
	FHIT

	FHL1
	FHL2
	FHL3
	FIBP
	FICD
	FIG4
	FIGNL2
	FILIP1L
	FIP1L1
	FIS1
	FIZ1
	FJX1
	FKBP11
	FKBP14
	FKBP15
	FKBP1A
	FKBP1B
	FKBP1P1
	FKBP2
	FKBP3
	FKBP4
	FKBP5
	FKBP8
	FKBP9L
	FKRP
	FKSG30
	FKTN
	FLAD1
	FLCN
	FLI1
	FLII
	FLJ10081
	FLJ10213
	FLJ10357
	FLJ10374
	FLJ10916
	FLJ10986
	FLJ12355
	FLJ12949
	FLJ20021
	FLJ20125
	FLJ20254
	FLJ20273
	FLJ20444
	FLJ20489
	FLJ20628
	FLJ20674
	FLJ20699
	FLJ20718
	FLJ20850
	FLJ20920
	FLJ21865
	FLJ21986
	FLJ22184
	FLJ22222
	FLJ22531

	FLJ22662
	FLJ22795
	FLJ23584
	FLJ25363
	FLJ30092
	FLJ31306
	FLJ33630
	FLJ35220
	FLJ35390
	FLJ36070
	FLJ36131
	FLJ37396
	FLJ37786
	FLJ38482
	FLJ38717
	FLJ38973
	FLJ39827
	FLJ40113
	FLJ40504
	FLJ40722
	FLJ40852
	FLJ42258
	FLJ42627
	FLJ43681
	FLJ44054
	FLJ44124
	FLJ45032
	FLJ45202
	FLJ45244
	FLJ45256
	FLJ46309
	FLJ46552
	FLJ46906
	FLJ90086
	FLNA
	FLNB
	FLNC
	FLOT1
	FLOT2
	FLRT3
	FLVCR2
	FLYWCH2
	FMO1
	FMO4
	FMO5
	FMO6P
	FN3KRP
	FNBP1
	FNBP1L
	FNBP4
	FNDC3A
	FNDC3B
	FNDC4
	FNDC5
	FNIP1
	FNIP2

	FNTA
	FNTB
	FOLH1
	FOLR1
	FOS
	FOSB
	FOSL2
	FOXA1
	FOXA2
	FOXA3
	FOXC1
	FOXC2
	FOXD4L4
	FOXJ2
	FOXJ3
	FOXK1
	FOXN2
	FOXO1
	FOXO3
	FOXO4
	FOXP1
	FOXQ1
	FOXRED1
	FOXRED2
	FRAG1
	FRAP1
	FRAT2
	FREQ
	FRG1
	FRMD6
	FRMD8
	FRRS1
	FRS3
	FRYL
	FSCN1
	FST
	FSTL1
	FSTL3
	FTCD
	FTH1
	FTHL11
	FTHL12
	FTHL16
	FTHL2
	FTHL3
	FTHL7
	FTHL8
	FTL
	FTO
	FTSJ1
	FTSJ2
	FTSJ3
	FTSJD1
	FTSJD2
	FUBP1
	FUBP3

	FUCA1
	FUCA2
	FUK
	FUNDC1
	FURIN
	FUS
	FUT11
	FUT4
	FUT6
	FUZ
	FVT1
	FXC1
	FXR1
	FXR2
	FXYD1
	FXYD2
	FXYD5
	FYCO1
	FYN
	FYTTD1
	FZD1
	FZD4
	FZD5
	FZD7
	FZD8
	FZR1
	G3BP1
	G3BP2
	G6PC3
	G6PD
	GAA
	GAB2
	GABARAP
	GABARAPL1
	GABARAPL2
	GABPA
	GABPB1
	GABPB2
	GABRE
	GAD1
	GADD45A
	GADD45B
	GADD45G
	GADD45GIP1
	GAK
	GAL
	GAL3ST1
	GALC
	GALE
	GALK1
	GALK2
	GALM
	GALNS
	GALNT1
	GALNT11
	GALNT2

	GALNT3
	GALNT4
	GALNTL4
	GALT
	GAMT
	GANAB
	GAPDH
	GAPDHL6
	GAPVD1
	GAR1
	GARNL4
	GARS
	GART
	GAS2L3
	GAS6
	GAS8
	GATA4
	GATAD1
	GATAD2A
	GATAD2B
	GATC
	GATM
	GATS
	GBA
	GBA2
	GBAS
	GBE1
	GBF1
	GBP1
	GBP2
	GBP4
	GCA
	GCAT
	GCC1
	GCDH
	GCET2
	GCGR
	GCH1
	GCHFR
	GCKR
	GCLC
	GCLM
	GCM1
	GCN1L1
	GCNT2
	GCNT3
	GCSH
	GDAP2
	GDE1
	GDF11
	GDF15
	GDI1
	GDI2
	GDPD1
	GDPD5
	GEMIN4

	GEMIN5
	GEMIN6
	GEMIN8
	GEN1
	GFM1
	GFM2
	GFOD1
	GFOD2
	GFPT1
	GGA1
	GGA2
	GGA3
	GGCT
	GGCX
	GGH
	GGNBP2
	GGPS1
	GHDC
	GHITM
	GHR
	GINS3
	GIPC1
	GIT1
	GIT2
	GIYD1
	GIYD2
	GJB1
	GJB2
	GJC1
	GKAP1
	GLA
	GLB1
	GLB1L
	GLCE
	GLDC
	GLE1
	GLG1
	GLI4
	GLIS3
	GLMN
	GLO1
	GLOD4
	GLRX
	GLRX2
	GLRX3
	GLRX5
	GLS
	GLS2
	GLT25D1
	GLT8D1
	GLTP
	GLTPD1
	GLTSCR1
	GLTSCR2
	GLUD1
	GLUL

	GLYCTK
	GM2A
	GMCL1
	GMDS
	GMEB2
	GMFB
	GMFG
	GMNN
	GMPPA
	GMPPB
	GMPR
	GMPR2
	GMPS
	GNA11
	GNA12
	GNA13
	GNAI1
	GNAI2
	GNAI3
	GNAQ
	GNAS
	GNB1
	GNB1L
	GNB2
	GNB2L1
	GNB5
	GNE
	GNG10
	GNG11
	GNG5
	GNG7
	GNL1
	GNL2
	GNL3
	GNL3L
	GNPAT
	GNPDA1
	GNPDA2
	GNPNAT1
	GNPTAB
	GNPTG
	GNRH1
	GNS
	GOLGA1
	GOLGA2
	GOLGA3
	GOLGA4
	GOLGA5
	GOLGA7
	GOLGA8B
	GOLGB1
	GOLIM4
	GOLM1
	GOLPH3
	GOLPH3L
	GOLPH4

	GOLSYN
	GOLT1A
	GOLT1B
	GON4L
	GOPC
	GORASP1
	GORASP2
	GOSR1
	GOSR2
	GOT1
	GOT2
	GP1BA
	GPAA1
	GPAM
	GPATCH2
	GPATCH3
	GPATCH4
	GPBP1
	GPBP1L1
	GPC2
	GPC6
	GPD1L
	GPER
	GPHN
	GPI
	GPKOW
	GPM6A
	GPN1
	GPN2
	GPN3
	GPR1
	GPR108
	GPR125
	GPR126
	GPR128
	GPR137
	GPR137B
	GPR143
	GPR162
	GPR172A
	GPR175
	GPR177
	GPR180
	GPR19
	GPR37
	GPR56
	GPR89A
	GPR89B
	GPR89C
	GPR98
	GPRASP2
	GPRC5C
	GPS1
	GPS2
	GPSM1
	GPT2

	GPX1
	GPX2
	GPX3
	GPX4
	GPX7
	GPX8
	GRAMD1A
	GRAMD3
	GRAMD4
	GRB10
	GRB14
	GRB2
	GRB7
	GREB1
	GRHPR
	GRINA
	GRIPAP1
	GRK5
	GRK6
	GRM2
	GRN
	GRPEL1
	GRPEL2
	GRSF1
	GRTP1
	GRWD1
	GSDMB
	GSDMD
	GSK3B
	GSN
	GSPT1
	GSPT2
	GSR
	GSS
	GSTA1
	GSTA2
	GSTA3
	GSTA4
	GSTA5
	GSTK1
	GSTM1
	GSTM2
	GSTM3
	GSTM4
	GSTO1
	GSTO2
	GSTP1
	GSTTP2
	GSTZ1
	GTDC1
	GTF2A1
	GTF2A2
	GTF2B
	GTF2E1
	GTF2E2
	GTF2F1

	GTF2F2
	GTF2H1
	GTF2H2B
	GTF2H3
	GTF2H4
	GTF2H5
	GTF2I
	GTF2IP1
	GTF2IRD1
	GTF2IRD2B
	GTF2IRD2P
	GTF3A
	GTF3C1
	GTF3C2
	GTF3C3
	GTF3C5
	GTF3C6
	GTPBP1
	GTPBP10
	GTPBP2
	GTPBP3
	GTPBP4
	GTPBP6
	GTPBP8
	GTSE1
	GUF1
	GUK1
	GUSB
	GUSBL1
	GUSBL2
	GXYLT1
	GYG1
	GYG2
	GYPC
	H19
	H1F0
	H1FX
	H2AFJ
	H2AFV
	H2AFX
	H2AFY
	H2AFY2
	H2AFZ
	H3F3A
	H3F3B
	H6PD
	HAAO
	HABP2
	HABP4
	HACE1
	HACL1
	HADH
	HADH2
	HADHA
	HADHB
	HAGH

	HAGHL
	HARBI1
	HARS
	HARS2
	HAS3
	HAT1
	HAUS4
	HAUS8
	HAVCR2
	HAX1
	HBEGF
	HBP1
	HBXIP
	HCCA2
	HCCS
	HCFC1
	HCFC1R1
	HCFC2
	HCG2P7
	HCG4
	HCN4
	HCP5
	HCST
	HDAC1
	HDAC11
	HDAC2
	HDAC3
	HDAC4
	HDAC6
	HDAC7A
	HDAC8
	HDDC2
	HDDC3
	HDGF
	HDGF2
	HDHD1A
	HDHD2
	HDHD3
	HEATR1
	HEATR2
	HEATR3
	HEATR5A
	HEATR5B
	HEATR6
	HEBP1
	HEBP2
	HECA
	HECTD1
	HECTD2
	HECTD3
	HEG1
	HELLS
	HELZ
	HEMK1
	HERC1
	HERC2

	HERC4
	HERC5
	HERC6
	HERPUD1
	HERPUD2
	HES1
	HES4
	HES6
	HEXB
	HEXDC
	HEXIM1
	HEXIM2
	HEY1
	HEY2
	HGS
	HHATL
	HHLA3
	HIAT1
	HIATL1
	HIATL2
	HIBADH
	HIBCH
	HIC2
	HIF1A
	HIF1AN
	HIGD1A
	HIGD2A
	HINFP
	HINT1
	HINT2
	HINT3
	HIP1
	HIP1R
	HIP2
	HIPK2
	HIRA
	HIRIP3
	HISPPD2A
	HIST1H1A
	HIST1H1C
	HIST1H2AC
	HIST1H2BC
	HIST1H2BD
	HIST1H2BE
	HIST1H2BG
	HIST1H2BK
	HIST1H3F
	HIST1H4C
	HIST1H4H
	HIST1H4K
	HIST2H2AA3
	HIST2H2AA4
	HIST2H2AC
	HIST2H2BE
	HIST2H4A
	HIST2H4B

	HIST3H2A
	HIVEP2
	HK1
	HK2
	HKDC1
	HLA-A
	HLA-A29.1
	HLA-B
	HLA-C
	HLA-DMA
	HLA-DRA
	HLA-DRB3
	HLA-DRB4
	HLA-DRB6
	HLA-E
	HLA-F
	HLA-G
	HLA-H
	HLCS
	HLTF
	HLX
	HM13
	HMBOX1
	HMBS
	HMG20A
	HMG20B
	HMGA1
	HMGB1
	HMGB1L1
	HMGB2
	HMGB3
	HMGCL
	HMGCR
	HMGCS1
	HMGCS2
	HMGN1
	HMGN2
	HMGN3
	HMGN4
	HMGXB4
	HMOX1
	HMOX2
	HN1
	HN1L
	HNF1A
	HNF1B
	HNMT
	HNRNPA0
	HNRNPA1
	HNRNPA1L2
	HNRNPA2B1
	HNRNPA3
	HNRNPA3P1
	HNRNPAB
	HNRNPC
	HNRNPD

	HNRNPF
	HNRNPH1
	HNRNPH2
	HNRNPH3
	HNRNPK
	HNRNPL
	HNRNPM
	HNRNPR
	HNRNPU
	HNRNPUL1
	HNRNPUL2
	HNRPA1L-2
	HNRPA1P4
	HNRPA2B1
	HNRPC
	HNRPDL
	HNRPH1
	HNRPH3
	HNRPK
	HNRPLL
	HNRPM
	HNRPR
	HNRPUL1
	HNRPUL2
	HOMER2
	HOOK1
	HOOK2
	HOOK3
	HOXA6
	HOXB5
	HOXB6
	HOXC13
	HOXC6
	HP1BP3
	HPCAL1
	HPD
	HPGD
	HPN
	HPRT1
	HPS1
	HPS3
	HPS4
	HPS5
	HPS6
	HPX
	HRAS
	HRASLS3
	HRB
	HRH1
	HRSP12
	HS1BP3
	HS2ST1
	HS3ST3A1
	HSBP1
	HSCB
	HSD11B1L

	HSD11B2
	HSD17B10
	HSD17B11
	HSD17B12
	HSD17B14
	HSD17B2
	HSD17B4
	HSD17B7
	HSD17B7P2
	HSD17B8
	HSD3B7
	HSDL1
	HSDL2
	HSF2
	HSGT1
	HSP90AA1
	HSP90AB1
	HSP90B1
	HSPA13
	HSPA14
	HSPA1A
	HSPA1B
	HSPA1L
	HSPA2
	HSPA4
	HSPA4L
	HSPA5
	HSPA6
	HSPA7
	HSPA8
	HSPA9
	HSPB1
	HSPBAP1
	HSPBL2
	HSPBP1
	HSPC111
	HSPC157
	HSPC159
	HSPC171
	HSPC268
	HSPCAL3
	HSPD1
	HSPE1
	HSPH1
	HSZFP36
	HTATIP2
	HTR1E
	HTRA1
	HTRA2
	HTT
	HUS1B
	HUWE1
	HYAL1
	HYAL2
	HYAL3
	HYDIN

	HYI
	HYLS1
	HYOU1
	IAH1
	IARS
	IARS2
	IBTK
	ICA1
	ICAM1
	ICAM3
	ICK
	ICMT
	ICT1
	ID1
	ID2
	ID2B
	ID3
	IDE
	IDH1
	IDH2
	IDH3A
	IDH3B
	IDH3G
	IDI1
	IDO2
	IDS
	IDUA
	IER2
	IER3
	IER3IP1
	IER5
	IFFO1
	IFFO2
	IFI16
	IFI27
	IFI27L1
	IFI27L2
	IFI35
	IFI6
	IFIH1
	IFIT1
	IFIT2
	IFIT5
	IFITM1
	IFITM2
	IFITM3
	IFITM4P
	IFNAR1
	IFNAR2
	IFNGR1
	IFNGR2
	IFP38
	IFRD1
	IFRD2
	IFT122
	IFT20

	IFT52
	IFT57
	IFT74
	IFT88
	IGBP1
	IGF2
	IGF2BP2
	IGF2R
	IGFBP1
	IGFBP2
	IGFBP3
	IGFBP4
	IGFBP6
	IGFBP7
	IGFBPL1
	IGHMBP2
	IGSF11
	IGSF3
	IGSF8
	IHPK3
	IK
	IKBIP
	IKBKB
	IKBKG
	IKZF2
	IKZF3
	IKZF5
	IL10
	IL10RB
	IL11RA
	IL13RA1
	IL16
	IL17RA
	IL17RB
	IL17RC
	IL17RD
	IL18
	IL18BP
	IL18R1
	IL1R1
	IL1R2
	IL1RAP
	IL21R
	IL24
	IL25
	IL27RA
	IL28RA
	IL32
	IL4R
	IL5RA
	IL8
	ILF2
	ILF3
	ILK
	ILKAP
	ILVBL

	IMAA
	IMMP2L
	IMMT
	IMP3
	IMP4
	IMPA1
	IMPA2
	IMPAD1
	IMPDH1
	IMPDH2
	INADL
	INCA1
	INF2
	ING1
	ING2
	ING3
	ING4
	INHBE
	INO80
	INO80B
	INO80C
	INO80D
	INO80E
	INPP1
	INPP5A
	INPP5B
	INPP5E
	INPP5F
	INPP5K
	INPPL1
	INSIG1
	INSIG2
	INS-IGF2
	INSM1
	INTS1
	INTS10
	INTS12
	INTS2
	INTS3
	INTS4
	INTS5
	INTS6
	INTS7
	INTS8
	INTS9
	INVS
	IP6K1
	IP6K2
	IPO11
	IPO13
	IPO4
	IPO5
	IPO7
	IPO8
	IPO9
	IPP

	IQCB1
	IQCC
	IQCG
	IQCK
	IQGAP1
	IQGAP2
	IQSEC1
	IRAK1
	IRAK2
	IREB2
	IRF1
	IRF2BP1
	IRF2BP2
	IRF3
	IRF4
	IRF7
	IRF8
	IRF9
	IRS1
	IRS2
	IRX1
	IRX3
	IRX5
	ISCA1
	ISCA1L
	ISCA2
	ISCU
	ISG15
	ISG20
	ISG20L1
	ISG20L2
	ISOC1
	ISOC2
	ISY1
	ISYNA1
	ITCH
	ITFG1
	ITFG2
	ITFG3
	ITGA1
	ITGA11
	ITGA2
	ITGA4
	ITGA5
	ITGA6
	ITGA7
	ITGAE
	ITGAV
	ITGB1
	ITGB1BP1
	ITGB2
	ITGB3BP
	ITGB4BP
	ITGB5
	ITIH1
	ITIH5

	ITM2B
	ITM2C
	ITPA
	ITPK1
	ITPKA
	ITPKC
	ITPR2
	ITPR3
	ITPRIP
	ITPRIPL2
	ITSN1
	IVD
	IVNS1ABP
	IWS1
	JAG1
	JAG2
	JAGN1
	JAK1
	JARID1A
	JARID1D
	JARID2
	JAZF1
	JCLN
	JDP2
	JMJD1A
	JMJD1C
	JMJD2B
	JMJD4
	JMJD6
	JMJD8
	JMY
	JOSD1
	JOSD2
	JPH2
	JTB
	JUB
	JUN
	JUND
	JUP
	KANK1
	KANK2
	KANK3
	KANK4
	KARS
	KAT2A
	KAT2B
	KAT5
	KATNA1
	KATNAL1
	KATNB1
	KBTBD11
	KBTBD2
	KBTBD3
	KBTBD4
	KBTBD7
	KBTBD8

	KBTBD9
	KCMF1
	KCNH6
	KCNJ10
	KCNJ4
	KCNJ8
	KCNK1
	KCNK3
	KCNK4
	KCNK5
	KCNK6
	KCNMA1
	KCNMB2
	KCNMB3
	KCNN4
	KCNQ1OT1
	KCNQ2
	KCNS3
	KCNT2
	KCTD10
	KCTD12
	KCTD13
	KCTD14
	KCTD18
	KCTD2
	KCTD20
	KCTD21
	KCTD3
	KCTD5
	KCTD6
	KCTD9
	KDELC1
	KDELC2
	KDELR1
	KDELR2
	KDELR3
	KDM3B
	KDM5B
	KDSR
	KEAP1
	KHDRBS1
	KHDRBS3
	KHK
	KHNYN
	KHSRP
	KIAA0020
	KIAA0090
	KIAA0100
	KIAA0101
	KIAA0114
	KIAA0133
	KIAA0141
	KIAA0146
	KIAA0174
	KIAA0182
	KIAA0194

	KIAA0195
	KIAA0196
	KIAA0232
	KIAA0240
	KIAA0247
	KIAA0251
	KIAA0258
	KIAA0261
	KIAA0319L
	KIAA0355
	KIAA0368
	KIAA0391
	KIAA0406
	KIAA0408
	KIAA0415
	KIAA0427
	KIAA0430
	KIAA0492
	KIAA0494
	KIAA0495
	KIAA0513
	KIAA0528
	KIAA0556
	KIAA0562
	KIAA0564
	KIAA0586
	KIAA0649
	KIAA0652
	KIAA0664
	KIAA0753
	KIAA0831
	KIAA0892
	KIAA0895
	KIAA0907
	KIAA0913
	KIAA0922
	KIAA0947
	KIAA1009
	KIAA1012
	KIAA1026
	KIAA1033
	KIAA1128
	KIAA1143
	KIAA1147
	KIAA1160
	KIAA1161
	KIAA1191
	KIAA1217
	KIAA1267
	KIAA1274
	KIAA1279
	KIAA1285
	KIAA1310
	KIAA1328
	KIAA1370
	KIAA1429

	KIAA1430
	KIAA1468
	KIAA1522
	KIAA1530
	KIAA1539
	KIAA1543
	KIAA1545
	KIAA1586
	KIAA1598
	KIAA1600
	KIAA1602
	KIAA1618
	KIAA1632
	KIAA1671
	KIAA1683
	KIAA1688
	KIAA1704
	KIAA1712
	KIAA1715
	KIAA1731
	KIAA1737
	KIAA1751
	KIAA1826
	KIAA1862
	KIAA1875
	KIAA1949
	KIAA1958
	KIAA1967
	KIAA1984
	KIAA2010
	KIAA2013
	KIAA2026
	KIDINS220
	KIF13B
	KIF1A
	KIF1B
	KIF1C
	KIF20B
	KIF21A
	KIF22
	KIF2A
	KIF3B
	KIF5B
	KIFAP3
	KIFC2
	KLB
	KLC1
	KLF11
	KLF12
	KLF13
	KLF15
	KLF2
	KLF3
	KLF4
	KLF6
	KLF9

	KLHDC2
	KLHDC3
	KLHDC4
	KLHDC5
	KLHDC8B
	KLHDC9
	KLHL12
	KLHL13
	KLHL18
	KLHL2
	KLHL20
	KLHL21
	KLHL22
	KLHL24
	KLHL26
	KLHL28
	KLHL29
	KLHL3
	KLHL36
	KLHL5
	KLHL7
	KLHL8
	KLHL9
	KLRAQ1
	KLRG1
	KNCN
	KPNA1
	KPNA2
	KPNA3
	KPNA4
	KPNA6
	KPNB1
	KPTN
	KRAS
	KRBA1
	KRCC1
	KREMEN1
	KRIT1
	KRR1
	KRT10
	KRT18
	KRT18P13
	KRT18P17
	KRT18P28
	KRT19
	KRT8
	KRT8P9
	KRTAP10-11
	KRTAP10-2
	KRTAP4-8
	KRTCAP2
	KRTCAP3
	KSR2
	KTELC1
	KTI12
	KTN1

	KYNU
	L2HGDH
	L3MBTL2
	L3MBTL3
	LACTB
	LACTB2
	LAD1
	LAGE3
	LAIR1
	LAMA5
	LAMB1
	LAMB2
	LAMC1
	LAMP1
	LAMP2
	LAMP3
	LANCL1
	LANCL2
	LAP3
	LAPTM4A
	LAPTM4B
	LARGE
	LARP1
	LARP1B
	LARP4
	LARP4B
	LARP7
	LARS
	LARS2
	LAS1L
	LASP1
	LASS2
	LASS4
	LASS5
	LASS6
	LBA1
	LBH
	LBR
	LBX2
	LCAT
	LCLAT1
	LCMT1
	LCMT2
	LCN1L1
	LCOR
	LCP1
	LDB2
	LDHA
	LDHB
	LDLR
	LDLRAP1
	LDOC1L
	LEAP2
	LEMD2
	LEMD3
	LENG1

	LEO1
	LEP
	LEPR
	LEPRE1
	LEPROT
	LEPROTL1
	LETM1
	LETMD1
	LFNG
	LGALS1
	LGALS3
	LGALS3BP
	LGALS8
	LGMN
	LGTN
	LHFP
	LHFPL2
	LHPP
	LIAS
	LIF
	LIG1
	LIG3
	LIG4
	LILRB1
	LILRB3
	LIMA1
	LIMCH1
	LIME1
	LIMS1
	LIMS2
	LIN37
	LIN52
	LIN54
	LIN7A
	LIN7C
	LINS1
	LIPA
	LIPC
	LIPG
	LIPT1
	LITAF
	LIX1
	LIX1L
	LLGL1
	LLGL2
	LLPH
	LMAN1
	LMAN2
	LMAN2L
	LMBR1
	LMBRD1
	LMCD1
	LMF2
	LMNA
	LMNB2
	LMO4

	LMOD3
	LMTK3
	LNPEP
	LNX2
	LOC100008588
	LOC100008589
	LOC100009676
	LOC100049716
	LOC100125556
	LOC100127893
	LOC100127894
	LOC100127913
	LOC100127918
	LOC100127922
	LOC100127975
	LOC100127982
	LOC100127993
	LOC100128002
	LOC100128007
	LOC100128016
	LOC100128056
	LOC100128060
	LOC100128062
	LOC100128083
	LOC100128084
	LOC100128086
	LOC100128098
	LOC100128115
	LOC100128126
	LOC100128163
	LOC100128168
	LOC100128196
	LOC100128221
	LOC100128266
	LOC100128274
	LOC100128288
	LOC100128291
	LOC100128309
	LOC100128326
	LOC100128337
	LOC100128353
	LOC100128356
	LOC100128392
	LOC100128410
	LOC100128423
	LOC100128425
	LOC100128440
	LOC100128460
	LOC100128469
	LOC100128485
	LOC100128498
	LOC100128505
	LOC100128507
	LOC100128510
	LOC100128525
	LOC100128528

	LOC100128547
	LOC100128548
	LOC100128585
	LOC100128627
	LOC100128672
	LOC100128689
	LOC100128731
	LOC100128760
	LOC100128771
	LOC100128775
	LOC100128805
	LOC100128816
	LOC100128836
	LOC100128883
	LOC100128893
	LOC100128899
	LOC100128918
	LOC100128936
	LOC100128975
	LOC100128994
	LOC100129022
	LOC100129027
	LOC100129028
	LOC100129034
	LOC100129055
	LOC100129067
	LOC100129086
	LOC100129093
	LOC100129094
	LOC100129104
	LOC100129118
	LOC100129139
	LOC100129141
	LOC100129158
	LOC100129201
	LOC100129203
	LOC100129211
	LOC100129237
	LOC100129243
	LOC100129267
	LOC100129269
	LOC100129295
	LOC100129297
	LOC100129303
	LOC100129335
	LOC100129362
	LOC100129379
	LOC100129424
	LOC100129426
	LOC100129441
	LOC100129478
	LOC100129502
	LOC100129518
	LOC100129522
	LOC100129539
	LOC100129543

	LOC100129550
	LOC100129552
	LOC100129553
	LOC100129566
	LOC100129585
	LOC100129599
	LOC100129608
	LOC100129630
	LOC100129645
	LOC100129650
	LOC100129657
	LOC100129668
	LOC100129673
	LOC100129681
	LOC100129685
	LOC100129697
	LOC100129716
	LOC100129742
	LOC100129758
	LOC100129759
	LOC100129781
	LOC100129828
	LOC100129866
	LOC100129882
	LOC100129890
	LOC100129902
	LOC100129906
	LOC100129907
	LOC100129934
	LOC100129952
	LOC100129958
	LOC100129960
	LOC100129975
	LOC100129982
	LOC100130003
	LOC100130053
	LOC100130070
	LOC100130071
	LOC100130092
	LOC100130093
	LOC100130131
	LOC100130138
	LOC100130154
	LOC100130168
	LOC100130171
	LOC100130178
	LOC100130190
	LOC100130233
	LOC100130263
	LOC100130276
	LOC100130289
	LOC100130291
	LOC100130308
	LOC100130332
	LOC100130353
	LOC100130445

	LOC100130446
	LOC100130476
	LOC100130511
	LOC100130516
	LOC100130522
	LOC100130550
	LOC100130552
	LOC100130553
	LOC100130556
	LOC100130561
	LOC100130562
	LOC100130598
	LOC100130604
	LOC100130623
	LOC100130624
	LOC100130633
	LOC100130701
	LOC100130707
	LOC100130715
	LOC100130746
	LOC100130750
	LOC100130764
	LOC100130775
	LOC100130802
	LOC100130818
	LOC100130835
	LOC100130837
	LOC100130856
	LOC100130886
	LOC100130892
	LOC100130905
	LOC100130914
	LOC100130919
	LOC100130932
	LOC100130934
	LOC100130980
	LOC100131009
	LOC100131085
	LOC100131096
	LOC100131160
	LOC100131166
	LOC100131187
	LOC100131196
	LOC100131205
	LOC100131261
	LOC100131294
	LOC100131308
	LOC100131323
	LOC100131336
	LOC100131349
	LOC100131360
	LOC100131368
	LOC100131381
	LOC100131387
	LOC100131403
	LOC100131452

	LOC100131510
	LOC100131526
	LOC100131530
	LOC100131531
	LOC100131541
	LOC100131572
	LOC100131609
	LOC100131643
	LOC100131655
	LOC100131672
	LOC100131675
	LOC100131713
	LOC100131718
	LOC100131735
	LOC100131737
	LOC100131744
	LOC100131767
	LOC100131785
	LOC100131786
	LOC100131787
	LOC100131801
	LOC100131810
	LOC100131835
	LOC100131850
	LOC100131859
	LOC100131866
	LOC100131905
	LOC100131940
	LOC100131970
	LOC100131971
	LOC100131980
	LOC100131989
	LOC100132024
	LOC100132032
	LOC100132037
	LOC100132086
	LOC100132106
	LOC100132139
	LOC100132199
	LOC100132213
	LOC100132247
	LOC100132266
	LOC100132291
	LOC100132299
	LOC100132308
	LOC100132323
	LOC100132324
	LOC100132347
	LOC100132352
	LOC100132369
	LOC100132391
	LOC100132394
	LOC100132418
	LOC100132425
	LOC100132444
	LOC100132457

	LOC100132474
	LOC100132485
	LOC100132488
	LOC100132493
	LOC100132496
	LOC100132499
	LOC100132503
	LOC100132510
	LOC100132513
	LOC100132519
	LOC100132526
	LOC100132528
	LOC100132535
	LOC100132547
	LOC100132564
	LOC100132585
	LOC100132593
	LOC100132652
	LOC100132657
	LOC100132658
	LOC100132673
	LOC100132705
	LOC100132715
	LOC100132717
	LOC100132727
	LOC100132728
	LOC100132740
	LOC100132742
	LOC100132761
	LOC100132767
	LOC100132773
	LOC100132774
	LOC100132787
	LOC100132795
	LOC100132797
	LOC100132804
	LOC100132805
	LOC100132829
	LOC100132863
	LOC100132894
	LOC100132909
	LOC100132918
	LOC100132992
	LOC100133008
	LOC100133012
	LOC100133017
	LOC100133019
	LOC100133036
	LOC100133045
	LOC100133055
	LOC100133129
	LOC100133163
	LOC100133177
	LOC100133185
	LOC100133222
	LOC100133224

	LOC100133232
	LOC100133233
	LOC100133264
	LOC100133273
	LOC100133277
	LOC100133298
	LOC100133328
	LOC100133329
	LOC100133372
	LOC100133390
	LOC100133402
	LOC100133465
	LOC100133477
	LOC100133478
	LOC100133489
	LOC100133516
	LOC100133517
	LOC100133565
	LOC100133578
	LOC100133600
	LOC100133607
	LOC100133609
	LOC100133649
	LOC100133662
	LOC100133692
	LOC100133697
	LOC100133758
	LOC100133772
	LOC100133773
	LOC100133795
	LOC100133800
	LOC100133803
	LOC100133812
	LOC100133823
	LOC100133836
	LOC100133840
	LOC100133851
	LOC100133876
	LOC100133888
	LOC100133916
	LOC100133923
	LOC100133930
	LOC100133931
	LOC100133950
	LOC100133999
	LOC100134011
	LOC100134018
	LOC100134053
	LOC100134083
	LOC100134108
	LOC100134122
	LOC100134134
	LOC100134144
	LOC100134147
	LOC100134159
	LOC100134182

	LOC100134188
	LOC100134189
	LOC100134209
	LOC100134253
	LOC100134273
	LOC100134291
	LOC100134300
	LOC100134304
	LOC100134361
	LOC100134364
	LOC100134393
	LOC100134436
	LOC100134440
	LOC100134468
	LOC100134504
	LOC100134530
	LOC100134537
	LOC100134540
	LOC100134584
	LOC100134634
	LOC100134648
	LOC100134868
	LOC100190938
	LOC100190939
	LOC100190986
	LOC113230
	LOC113386
	LOC123688
	LOC124512
	LOC126235
	LOC127295
	LOC128192
	LOC130773
	LOC131691
	LOC132241
	LOC134997
	LOC136143
	LOC137107
	LOC143543
	LOC143666
	LOC144438
	LOC145853
	LOC146053
	LOC146177
	LOC146517
	LOC146909
	LOC147727
	LOC147804
	LOC148413
	LOC148430
	LOC148915
	LOC149134
	LOC149448
	LOC149501
	LOC150051
	LOC150568

	LOC151162
	LOC151579
	LOC152195
	LOC152217
	LOC158160
	LOC158301
	LOC158345
	LOC161527
	LOC163233
	LOC196752
	LOC200030
	LOC201229
	LOC201725
	LOC202051
	LOC202227
	LOC202781
	LOC203547
	LOC205251
	LOC220433
	LOC220686
	LOC221710
	LOC23117
	LOC253039
	LOC255167
	LOC255275
	LOC255326
	LOC255783
	LOC257396
	LOC25845
	LOC283267
	LOC283412
	LOC283481
	LOC283788
	LOC283849
	LOC283953
	LOC283999
	LOC284167
	LOC284230
	LOC284393
	LOC284422
	LOC284821
	LOC284988
	LOC285053
	LOC285074
	LOC285176
	LOC285550
	LOC285733
	LOC285741
	LOC285900
	LOC286016
	LOC286157
	LOC286208
	LOC286444
	LOC286467
	LOC286512
	LOC338758

	LOC338870
	LOC339290
	LOC339352
	LOC339778
	LOC339804
	LOC339843
	LOC339970
	LOC340260
	LOC340274
	LOC340598
	LOC341230
	LOC341315
	LOC341457
	LOC341784
	LOC341965
	LOC342994
	LOC343184
	LOC344328
	LOC345041
	LOC345645
	LOC346085
	LOC346950
	LOC347292
	LOC347376
	LOC347544
	LOC374395
	LOC387703
	LOC387753
	LOC387763
	LOC387790
	LOC387791
	LOC387820
	LOC387825
	LOC387841
	LOC387867
	LOC387882
	LOC387930
	LOC388076
	LOC388122
	LOC388275
	LOC388282
	LOC388339
	LOC388344
	LOC388401
	LOC388474
	LOC388524
	LOC388532
	LOC388556
	LOC388564
	LOC388588
	LOC388621
	LOC388654
	LOC388707
	LOC388720
	LOC388789
	LOC388796

	LOC388814
	LOC388907
	LOC388969
	LOC389049
	LOC389101
	LOC389137
	LOC389141
	LOC389156
	LOC389168
	LOC389203
	LOC389223
	LOC389286
	LOC389293
	LOC389322
	LOC389342
	LOC389386
	LOC389404
	LOC389435
	LOC389517
	LOC389599
	LOC389662
	LOC389672
	LOC389765
	LOC389787
	LOC389873
	LOC389901
	LOC390183
	LOC390251
	LOC390298
	LOC390345
	LOC390354
	LOC390466
	LOC390530
	LOC390557
	LOC390578
	LOC390660
	LOC390671
	LOC390735
	LOC390876
	LOC390956
	LOC391019
	LOC391044
	LOC391075
	LOC391126
	LOC391132
	LOC391169
	LOC391334
	LOC391370
	LOC391532
	LOC391578
	LOC391655
	LOC391656
	LOC391670
	LOC391692
	LOC391769
	LOC391777

	LOC391811
	LOC391825
	LOC391833
	LOC392008
	LOC392285
	LOC392301
	LOC392437
	LOC392501
	LOC392522
	LOC392713
	LOC392871
	LOC399491
	LOC399744
	LOC399748
	LOC399804
	LOC399881
	LOC399900
	LOC399942
	LOC399959
	LOC399965
	LOC399988
	LOC400013
	LOC400027
	LOC400061
	LOC400145
	LOC400304
	LOC400389
	LOC400446
	LOC400455
	LOC400506
	LOC400652
	LOC400657
	LOC400713
	LOC400721
	LOC400750
	LOC400890
	LOC400948
	LOC400963
	LOC401010
	LOC401019
	LOC401076
	LOC401098
	LOC401115
	LOC401127
	LOC401152
	LOC401206
	LOC401218
	LOC401238
	LOC401321
	LOC401357
	LOC401397
	LOC401537
	LOC401561
	LOC401640
	LOC401648
	LOC401676

	LOC401677
	LOC401717
	LOC401720
	LOC401817
	LOC401847
	LOC402057
	LOC402112
	LOC402175
	LOC402221
	LOC402251
	LOC402342
	LOC402509
	LOC402560
	LOC402562
	LOC402644
	LOC402677
	LOC402694
	LOC407835
	LOC439950
	LOC439953
	LOC439992
	LOC439994
	LOC440027
	LOC440043
	LOC440055
	LOC440063
	LOC440093
	LOC440157
	LOC440280
	LOC440311
	LOC440341
	LOC440345
	LOC440348
	LOC440349
	LOC440353
	LOC440354
	LOC440359
	LOC440366
	LOC440389
	LOC440396
	LOC440487
	LOC440498
	LOC440509
	LOC440525
	LOC440563
	LOC440575
	LOC440589
	LOC440595
	LOC440704
	LOC440731
	LOC440733
	LOC440737
	LOC440748
	LOC440776
	LOC440895
	LOC440926

	LOC440927
	LOC440957
	LOC440991
	LOC440993
	LOC441009
	LOC441013
	LOC441019
	LOC441032
	LOC441034
	LOC441050
	LOC441073
	LOC441087
	LOC441089
	LOC441124
	LOC441131
	LOC441150
	LOC441154
	LOC441155
	LOC441191
	LOC441241
	LOC441246
	LOC441251
	LOC441253
	LOC441377
	LOC441378
	LOC441408
	LOC441426
	LOC441453
	LOC441454
	LOC441455
	LOC441461
	LOC441481
	LOC441484
	LOC441506
	LOC441550
	LOC441632
	LOC441642
	LOC441743
	LOC441763
	LOC441775
	LOC441876
	LOC441896
	LOC442064
	LOC442153
	LOC442162
	LOC442180
	LOC442232
	LOC442270
	LOC442421
	LOC442442
	LOC442454
	LOC442582
	LOC442727
	LOC550643
	LOC552889
	LOC553158

	LOC554203
	LOC554206
	LOC572558
	LOC613037
	LOC641298
	LOC641367
	LOC641727
	LOC641746
	LOC641750
	LOC641768
	LOC641798
	LOC641802
	LOC641814
	LOC641819
	LOC641820
	LOC641844
	LOC641848
	LOC641849
	LOC641972
	LOC641992
	LOC641996
	LOC642017
	LOC642031
	LOC642033
	LOC642076
	LOC642082
	LOC642197
	LOC642210
	LOC642236
	LOC642250
	LOC642255
	LOC642299
	LOC642333
	LOC642357
	LOC642361
	LOC642367
	LOC642377
	LOC642393
	LOC642443
	LOC642446
	LOC642449
	LOC642468
	LOC642469
	LOC642489
	LOC642502
	LOC642513
	LOC642567
	LOC642585
	LOC642590
	LOC642691
	LOC642726
	LOC642738
	LOC642741
	LOC642755
	LOC642771
	LOC642784

	LOC642815
	LOC642817
	LOC642828
	LOC642838
	LOC642892
	LOC642897
	LOC642921
	LOC642934
	LOC642946
	LOC642947
	LOC642956
	LOC642975
	LOC642981
	LOC642989
	LOC643007
	LOC643011
	LOC643031
	LOC643035
	LOC643123
	LOC643167
	LOC643176
	LOC643206
	LOC643220
	LOC643224
	LOC643284
	LOC643287
	LOC643300
	LOC643308
	LOC643310
	LOC643319
	LOC643336
	LOC643357
	LOC643358
	LOC643384
	LOC643387
	LOC643431
	LOC643433
	LOC643438
	LOC643446
	LOC643452
	LOC643507
	LOC643509
	LOC643531
	LOC643534
	LOC643665
	LOC643668
	LOC643778
	LOC643779
	LOC643790
	LOC643802
	LOC643856
	LOC643863
	LOC643870
	LOC643873
	LOC643882
	LOC643894

	LOC643905
	LOC643911
	LOC643949
	LOC643960
	LOC643995
	LOC643997
	LOC644029
	LOC644033
	LOC644037
	LOC644039
	LOC644063
	LOC644075
	LOC644096
	LOC644101
	LOC644124
	LOC644128
	LOC644131
	LOC644132
	LOC644162
	LOC644172
	LOC644191
	LOC644214
	LOC644237
	LOC644250
	LOC644256
	LOC644265
	LOC644310
	LOC644315
	LOC644322
	LOC644330
	LOC644338
	LOC644360
	LOC644363
	LOC644380
	LOC644384
	LOC644422
	LOC644423
	LOC644464
	LOC644482
	LOC644511
	LOC644517
	LOC644563
	LOC644591
	LOC644604
	LOC644615
	LOC644619
	LOC644634
	LOC644642
	LOC644670
	LOC644684
	LOC644694
	LOC644739
	LOC644743
	LOC644745
	LOC644761
	LOC644762

	LOC644774
	LOC644790
	LOC644799
	LOC644809
	LOC644816
	LOC644852
	LOC644860
	LOC644863
	LOC644869
	LOC644877
	LOC644879
	LOC644889
	LOC644907
	LOC644914
	LOC644928
	LOC644931
	LOC644934
	LOC644935
	LOC644936
	LOC644937
	LOC644949
	LOC644950
	LOC644979
	LOC644988
	LOC644990
	LOC645015
	LOC645018
	LOC645058
	LOC645086
	LOC645094
	LOC645100
	LOC645138
	LOC645157
	LOC645166
	LOC645173
	LOC645174
	LOC645175
	LOC645176
	LOC645231
	LOC645233
	LOC645236
	LOC645251
	LOC645289
	LOC645296
	LOC645313
	LOC645317
	LOC645332
	LOC645351
	LOC645362
	LOC645378
	LOC645381
	LOC645385
	LOC645387
	LOC645430
	LOC645436
	LOC645452

	LOC645466
	LOC645489
	LOC645515
	LOC645586
	LOC645605
	LOC645609
	LOC645630
	LOC645671
	LOC645683
	LOC645688
	LOC645691
	LOC645693
	LOC645715
	LOC645737
	LOC645762
	LOC645895
	LOC645899
	LOC645904
	LOC645937
	LOC645968
	LOC645969
	LOC645979
	LOC646034
	LOC646044
	LOC646093
	LOC646103
	LOC646135
	LOC646195
	LOC646197
	LOC646200
	LOC646214
	LOC646278
	LOC646294
	LOC646301
	LOC646316
	LOC646347
	LOC646350
	LOC646403
	LOC646463
	LOC646476
	LOC646483
	LOC646508
	LOC646527
	LOC646531
	LOC646547
	LOC646567
	LOC646630
	LOC646672
	LOC646688
	LOC646723
	LOC646746
	LOC646753
	LOC646766
	LOC646783
	LOC646784
	LOC646785

	LOC646786
	LOC646791
	LOC646819
	LOC646821
	LOC646836
	LOC646841
	LOC646849
	LOC646897
	LOC646900
	LOC646909
	LOC646936
	LOC646942
	LOC646949
	LOC646956
	LOC646966
	LOC646981
	LOC646993
	LOC646996
	LOC647000
	LOC647009
	LOC647030
	LOC647037
	LOC647074
	LOC647081
	LOC647086
	LOC647099
	LOC647104
	LOC647150
	LOC647169
	LOC647243
	LOC647276
	LOC647285
	LOC647302
	LOC647307
	LOC647322
	LOC647340
	LOC647346
	LOC647349
	LOC647361
	LOC647363
	LOC647389
	LOC647436
	LOC647450
	LOC647456
	LOC647597
	LOC647650
	LOC647673
	LOC647691
	LOC647741
	LOC647834
	LOC647856
	LOC647859
	LOC647886
	LOC647949
	LOC647954
	LOC648000

	LOC648024
	LOC648059
	LOC648099
	LOC648103
	LOC648176
	LOC648210
	LOC648249
	LOC648283
	LOC648294
	LOC648343
	LOC648370
	LOC648390
	LOC648399
	LOC648526
	LOC648581
	LOC648605
	LOC648622
	LOC648638
	LOC648659
	LOC648665
	LOC648682
	LOC648695
	LOC648705
	LOC648729
	LOC648740
	LOC648742
	LOC648744
	LOC648771
	LOC648822
	LOC648852
	LOC648907
	LOC648921
	LOC648927
	LOC648980
	LOC649009
	LOC649044
	LOC649049
	LOC649076
	LOC649143
	LOC649150
	LOC649169
	LOC649209
	LOC649214
	LOC649330
	LOC649365
	LOC649447
	LOC649548
	LOC649553
	LOC649555
	LOC649679
	LOC649821
	LOC649839
	LOC649864
	LOC649873
	LOC649946
	LOC649999

	LOC650020
	LOC650029
	LOC650111
	LOC650116
	LOC650152
	LOC650157
	LOC650215
	LOC650254
	LOC650276
	LOC650293
	LOC650298
	LOC650321
	LOC650369
	LOC650515
	LOC650518
	LOC650646
	LOC650698
	LOC650717
	LOC650737
	LOC650739
	LOC650757
	LOC650780
	LOC650803
	LOC650826
	LOC650832
	LOC650898
	LOC650909
	LOC651029
	LOC651064
	LOC651073
	LOC651137
	LOC651143
	LOC651149
	LOC651166
	LOC651198
	LOC651202
	LOC651296
	LOC651436
	LOC651453
	LOC651575
	LOC651576
	LOC651621
	LOC651680
	LOC651697
	LOC651816
	LOC651894
	LOC651919
	LOC652044
	LOC652071
	LOC652226
	LOC652281
	LOC652322
	LOC652388
	LOC652481
	LOC652489
	LOC652541

	LOC652545
	LOC652565
	LOC652595
	LOC652607
	LOC652608
	LOC652624
	LOC652627
	LOC652634
	LOC652669
	LOC652672
	LOC652685
	LOC652698
	LOC652726
	LOC652736
	LOC652741
	LOC652826
	LOC652864
	LOC652903
	LOC652968
	LOC652993
	LOC653034
	LOC653071
	LOC653079
	LOC653080
	LOC653086
	LOC653103
	LOC653108
	LOC653115
	LOC653147
	LOC653156
	LOC653162
	LOC653171
	LOC653226
	LOC653232
	LOC653242
	LOC653257
	LOC653308
	LOC653314
	LOC653324
	LOC653333
	LOC653344
	LOC653352
	LOC653375
	LOC653377
	LOC653381
	LOC653382
	LOC653383
	LOC653419
	LOC653421
	LOC653438
	LOC653450
	LOC653472
	LOC653479
	LOC653489
	LOC653496
	LOC653505

	LOC653506
	LOC653557
	LOC653566
	LOC653583
	LOC653590
	LOC653631
	LOC653635
	LOC653658
	LOC653702
	LOC653720
	LOC653737
	LOC653752
	LOC653773
	LOC653778
	LOC653820
	LOC653829
	LOC653874
	LOC653881
	LOC653884
	LOC653888
	LOC653972
	LOC653994
	LOC654000
	LOC654074
	LOC654085
	LOC654103
	LOC654121
	LOC654126
	LOC654135
	LOC654155
	LOC654161
	LOC654174
	LOC654189
	LOC654191
	LOC654194
	LOC654201
	LOC654244
	LOC654260
	LOC654342
	LOC654350
	LOC678655
	LOC723805
	LOC723972
	LOC727726
	LOC727732
	LOC727758
	LOC727761
	LOC727762
	LOC727773
	LOC727797
	LOC727803
	LOC727808
	LOC727818
	LOC727820
	LOC727821
	LOC727825

	LOC727826
	LOC727865
	LOC727866
	LOC727877
	LOC727899
	LOC727947
	LOC727950
	LOC727962
	LOC727963
	LOC727967
	LOC727970
	LOC727984
	LOC727987
	LOC728002
	LOC728006
	LOC728014
	LOC728026
	LOC728031
	LOC728037
	LOC728059
	LOC728060
	LOC728069
	LOC728086
	LOC728098
	LOC728105
	LOC728126
	LOC728127
	LOC728128
	LOC728138
	LOC728139
	LOC728142
	LOC728147
	LOC728153
	LOC728179
	LOC728181
	LOC728188
	LOC728207
	LOC728208
	LOC728216
	LOC728226
	LOC728229
	LOC728244
	LOC728262
	LOC728263
	LOC728310
	LOC728312
	LOC728324
	LOC728368
	LOC728408
	LOC728416
	LOC728428
	LOC728431
	LOC728440
	LOC728453
	LOC728457
	LOC728467

	LOC728473
	LOC728476
	LOC728481
	LOC728484
	LOC728485
	LOC728492
	LOC728499
	LOC728517
	LOC728532
	LOC728533
	LOC728537
	LOC728553
	LOC728554
	LOC728556
	LOC728564
	LOC728565
	LOC728572
	LOC728576
	LOC728590
	LOC728602
	LOC728620
	LOC728635
	LOC728640
	LOC728643
	LOC728650
	LOC728658
	LOC728661
	LOC728666
	LOC728672
	LOC728689
	LOC728693
	LOC728698
	LOC728728
	LOC728732
	LOC728734
	LOC728739
	LOC728741
	LOC728748
	LOC728755
	LOC728758
	LOC728772
	LOC728774
	LOC728779
	LOC728780
	LOC728782
	LOC728787
	LOC728791
	LOC728809
	LOC728811
	LOC728820
	LOC728823
	LOC728825
	LOC728831
	LOC728843
	LOC728844
	LOC728855

	LOC728873
	LOC728877
	LOC728888
	LOC728901
	LOC728903
	LOC728908
	LOC728919
	LOC728931
	LOC728937
	LOC728944
	LOC728953
	LOC728965
	LOC728969
	LOC728973
	LOC728975
	LOC728979
	LOC728992
	LOC729004
	LOC729009
	LOC729021
	LOC729081
	LOC729082
	LOC729086
	LOC729090
	LOC729101
	LOC729102
	LOC729120
	LOC729123
	LOC729142
	LOC729148
	LOC729200
	LOC729208
	LOC729217
	LOC729222
	LOC729236
	LOC729255
	LOC729259
	LOC729279
	LOC729298
	LOC729301
	LOC729313
	LOC729317
	LOC729324
	LOC729332
	LOC729340
	LOC729342
	LOC729348
	LOC729362
	LOC729366
	LOC729375
	LOC729397
	LOC729402
	LOC729404
	LOC729406
	LOC729417
	LOC729421

	LOC729423
	LOC729439
	LOC729446
	LOC729466
	LOC729484
	LOC729495
	LOC729500
	LOC729505
	LOC729510
	LOC729513
	LOC729519
	LOC729535
	LOC729559
	LOC729580
	LOC729587
	LOC729595
	LOC729603
	LOC729608
	LOC729617
	LOC729646
	LOC729660
	LOC729666
	LOC729677
	LOC729679
	LOC729680
	LOC729686
	LOC729687
	LOC729692
	LOC729708
	LOC729742
	LOC729760
	LOC729768
	LOC729769
	LOC729774
	LOC729776
	LOC729779
	LOC729780
	LOC729789
	LOC729798
	LOC729816
	LOC729841
	LOC729843
	LOC729852
	LOC729859
	LOC729887
	LOC729898
	LOC729903
	LOC729920
	LOC729926
	LOC729960
	LOC729964
	LOC729970
	LOC729978
	LOC729985
	LOC729992
	LOC730004

	LOC730005
	LOC730020
	LOC730029
	LOC730050
	LOC730052
	LOC730060
	LOC730074
	LOC730081
	LOC730098
	LOC730101
	LOC730107
	LOC730134
	LOC730167
	LOC730176
	LOC730187
	LOC730202
	LOC730226
	LOC730235
	LOC730236
	LOC730246
	LOC730254
	LOC730255
	LOC730256
	LOC730273
	LOC730278
	LOC730284
	LOC730288
	LOC730313
	LOC730316
	LOC730323
	LOC730324
	LOC730358
	LOC730382
	LOC730415
	LOC730417
	LOC730432
	LOC730455
	LOC730525
	LOC730534
	LOC730535
	LOC730704
	LOC730740
	LOC730744
	LOC730746
	LOC730754
	LOC730820
	LOC730841
	LOC730990
	LOC730993
	LOC731007
	LOC731049
	LOC731096
	LOC731196
	LOC731231
	LOC731308
	LOC731314

	LOC731365
	LOC731542
	LOC731640
	LOC731724
	LOC731777
	LOC731779
	LOC731789
	LOC731878
	LOC731950
	LOC731954
	LOC731985
	LOC731999
	LOC732007
	LOC732075
	LOC732165
	LOC732172
	LOC732360
	LOC732425
	LOC791120
	LOC88523
	LOC90342
	LOC90586
	LOC90624
	LOC91316
	LOC91561
	LOC91664
	LOC92249
	LOC92497
	LOC92755
	LOC92973
	LOC93622
	LOH12CR1
	LONP1
	LONP2
	LONRF1
	LOXL4
	LPAR3
	LPCAT1
	LPCAT3
	LPCAT4
	LPGAT1
	LPHN2
	LPIN1
	LPIN2
	LPP
	LPPR2
	LPXN
	LQK1
	LRAP
	LRBA
	LRCH3
	LRCH4
	LRDD
	LRFN3
	LRG1
	LRIG1

	LRIG2
	LRP1
	LRP10
	LRP11
	LRP3
	LRP5
	LRP5L
	LRPAP1
	LRPPRC
	LRRC1
	LRRC14
	LRRC20
	LRRC28
	LRRC29
	LRRC3
	LRRC32
	LRRC37B
	LRRC37B2
	LRRC40
	LRRC41
	LRRC42
	LRRC45
	LRRC47
	LRRC56
	LRRC57
	LRRC58
	LRRC59
	LRRC61
	LRRC8A
	LRRC8D
	LRRC8E
	LRRCC1
	LRRFIP1
	LRRFIP2
	LRSAM1
	LRTOMT
	LRWD1
	LSG1
	LSM1
	LSM10
	LSM12
	LSM14A
	LSM2
	LSM3
	LSM4
	LSM5
	LSM6
	LSM7
	LSM8
	LSMD1
	LSP1
	LSR
	LSS
	LTA
	LTA4H
	LTB

	LTBP3
	LTBP4
	LTBR
	LTF
	LTV1
	LUC7L
	LUC7L2
	LUZP1
	LY6E
	LY6H
	LYAR
	LYL1
	LYN
	LYPD3
	LYPLA1
	LYPLA2
	LYPLAL1
	LYRM1
	LYRM2
	LYRM4
	LYRM5
	LYRM7
	LYSMD2
	LYSMD3
	LYSMD4
	LZIC
	LZTFL1
	LZTR1
	LZTS2
	M6PR
	M6PRBP1
	MACF1
	MACROD1
	MAD1L1
	MAD2L1BP
	MAD2L2
	MADD
	MAEA
	MAF
	MAF1
	MAFB
	MAFF
	MAFG
	MAGED1
	MAGED2
	MAGED4B
	MAGEE1
	MAGEF1
	MAGEH1
	MAGEL2
	Magmas
	MAGOH
	MAGT1
	MAK10
	MAK16
	MAL2

	MALT1
	MAMDC4
	MAML1
	MAN1A1
	MAN1A2
	MAN1B1
	MAN1C1
	MAN2A1
	MAN2A2
	MAN2B1
	MAN2B2
	MAN2C1
	MANBA
	MANBAL
	MANEA
	MANSC1
	MAOA
	MAP1LC3A
	MAP1LC3B
	MAP1S
	MAP2K1
	MAP2K1IP1
	MAP2K2
	MAP2K3
	MAP2K4
	MAP2K5
	MAP2K7
	MAP3K1
	MAP3K10
	MAP3K11
	MAP3K12
	MAP3K14
	MAP3K2
	MAP3K3
	MAP3K4
	MAP3K6
	MAP3K7
	MAP3K7IP1
	MAP3K7IP2
	MAP3K8
	MAP4
	MAP4K2
	MAP4K3
	MAP4K4
	MAP4K5
	MAP6D1
	MAP7
	MAP7D1
	MAPBPIP
	MAPK1
	MAPK13
	MAPK14
	MAPK3
	MAPK6
	MAPK7
	MAPK8IP3

	MAPK9
	MAPKAP1
	MAPKAPK2
	MAPKAPK3
	MAPKAPK5
	MAPKSP1
	MAPRE1
	MAPRE2
	MAPRE3
	MARCH2
	MARCH3
	MARCH5
	MARCH6
	MARCH7
	MARCKS
	MARCKSL1
	MARK2
	MARK3
	MARS
	MARS2
	MARVELD2
	MARVELD3
	MASP1
	MAST2
	MAST3
	MASTL
	MAT2A
	MAT2B
	MATK
	MATN2
	MATR3
	MAX
	MBD1
	MBD2
	MBD3
	MBD4
	MBD6
	MBIP
	MBNL1
	MBNL2
	MBNL3
	MBOAT1
	MBOAT7
	MBP
	MBTD1
	MBTPS1
	MC1R
	MCART1
	MCAT
	MCCC1
	MCEE
	MCFD2
	MCHR2
	MCL1
	MCM3
	MCM3AP

	MCM4
	MCM5
	MCM6
	MCM7
	MCM8
	MCOLN1
	MCPH1
	MCRS1
	MCTS1
	MDC1
	MDH1
	MDH2
	MDK
	MDM1
	MDM2
	MDN1
	MDP1
	ME1
	ME2
	ME3
	MEA1
	MEAF6
	MED1
	MED10
	MED11
	MED12
	MED13L
	MED14
	MED15
	MED16
	MED17
	MED19
	MED20
	MED21
	MED22
	MED23
	MED24
	MED25
	MED26
	MED27
	MED28
	MED29
	MED30
	MED31
	MED4
	MED6
	MED7
	MED8
	MED9
	MEF2A
	MEF2D
	MEGF10
	MEGF8
	MEGF9
	MEIS1
	MEIS2

	MEIS3P1
	MEMO1
	MEN1
	MEPCE
	MERTK
	MEST
	MET
	METAP1
	METAP2
	METRN
	METRNL
	METT11D1
	METT5D1
	METTL1
	METTL10
	METTL11A
	METTL13
	METTL14
	METTL2A
	METTL3
	METTL5
	METTL6
	METTL7A
	METTL7B
	METTL9
	MEX3A
	MEX3C
	MEX3D
	MFAP1
	MFAP2
	MFAP3
	MFAP3L
	MFF
	MFGE8
	MFHAS1
	MFN1
	MFN2
	MFSD1
	MFSD10
	MFSD11
	MFSD3
	MFSD5
	MFSD6
	MFSD8
	MGA
	MGAT1
	MGAT2
	MGAT4A
	MGAT4B
	MGC10997
	MGC12760
	MGC13057
	MGC15763
	MGC16169
	MGC16384
	MGC16703

	MGC18216
	MGC23284
	MGC26356
	MGC2752
	MGC29506
	MGC3020
	MGC3032
	MGC3196
	MGC33556
	MGC35361
	MGC3731
	MGC40489
	MGC4677
	MGC52000
	MGC57346
	MGC61598
	MGC70857
	MGC71993
	MGC72080
	MGC72104
	MGC87042
	MGC87895
	MGEA5
	MGLL
	MGMT
	MGP
	MGRN1
	MGST1
	MGST2
	MGST3
	MIA3
	MIB1
	MIB2
	MICAL1
	MICALL2
	MID1
	MID1IP1
	MID2
	MIDN
	MIER1
	MIER2
	MIF
	MIF4GD
	MIIP
	MINA
	MINPP1
	MIOS
	MIPEP
	MIR1185-1
	MIR1228
	MIR1253
	MIR125B2
	MIR129-2
	MIR130A
	MIR1974
	MIR1978

	MIR2116
	MIR29B1
	MIR300
	MIR342
	MIR345
	MIR373
	MIR488
	MIR557
	MIR574
	MIR586
	MIR599
	MIR607
	MIR635
	MIR657
	MIR877
	MIS12
	MITD1
	MKI67IP
	MKKS
	MKL1
	MKL2
	MKLN1
	MKNK1
	MKNK2
	MKRN1
	MKRN2
	MLEC
	MLF2
	MLH1
	MLL
	MLL3
	MLL4
	MLL5
	MLLT10
	MLLT11
	MLLT6
	MLPH
	MLST8
	MLX
	MLXIPL
	MLYCD
	MMAA
	MMAB
	MMACHC
	MMADHC
	MMD
	MME
	MMGT1
	MMP15
	MMP23A
	MMP23B
	MMS19
	MMS19L
	MN1
	MNAT1
	MNS1

	MNT
	MOAP1
	MOBK1B
	MOBKL1A
	MOBKL1B
	MOBKL2A
	MOBKL2C
	MOBKL3
	MOCOS
	MOCS1
	MOCS2
	MOGS
	MON1A
	MON1B
	MON2
	MORC2
	MORC3
	MORC4
	MORF4L1
	MORF4L2
	MORG1
	MORN2
	MOS
	MOSC1
	MOSC2
	MOSPD1
	MOSPD2
	MOSPD3
	MOV10
	MPDU1
	MPDZ
	MPG
	MPHOSPH10
	MPHOSPH6
	MPHOSPH8
	MPHOSPH9
	MPI
	MPND
	MPP1
	MPP5
	MPP6
	MPPE1
	MPRIP
	MPST
	MPV17
	MPV17L2
	MPZL1
	MPZL2
	MRFAP1
	MRFAP1L1
	MRI1
	MRLC2
	MRP63
	MRPL1
	MRPL10
	MRPL11

	MRPL12
	MRPL13
	MRPL14
	MRPL15
	MRPL16
	MRPL17
	MRPL18
	MRPL19
	MRPL2
	MRPL20
	MRPL21
	MRPL22
	MRPL23
	MRPL24
	MRPL27
	MRPL28
	MRPL3
	MRPL30
	MRPL32
	MRPL33
	MRPL34
	MRPL35
	MRPL36
	MRPL37
	MRPL38
	MRPL39
	MRPL4
	MRPL40
	MRPL41
	MRPL42
	MRPL43
	MRPL44
	MRPL45
	MRPL46
	MRPL47
	MRPL48
	MRPL49
	MRPL50
	MRPL51
	MRPL52
	MRPL53
	MRPL54
	MRPL55
	MRPL9
	MRPS10
	MRPS11
	MRPS12
	MRPS14
	MRPS15
	MRPS16
	MRPS17
	MRPS18A
	MRPS18B
	MRPS18C
	MRPS2
	MRPS21

	MRPS22
	MRPS23
	MRPS24
	MRPS25
	MRPS26
	MRPS27
	MRPS28
	MRPS30
	MRPS31
	MRPS33
	MRPS34
	MRPS35
	MRPS36
	MRPS5
	MRPS6
	MRPS7
	MRPS9
	MRRF
	MRS2
	MRTO4
	MSH3
	MSH6
	MSI1
	MSI2
	MSL1
	MSL2
	MSL3
	MSL3L1
	MSN
	MSRA
	MSRB2
	MST1
	MST4
	MSTO1
	MT1A
	MT1E
	MT1F
	MT1G
	MT1H
	MT1X
	MT2A
	MTA1
	MTA2
	MTA3
	MTAP
	MTCH1
	MTCH2
	MTCP1
	MTDH
	MTE
	MTERF
	MTERFD1
	MTF1
	MTF2
	MTFMT
	MTFR1

	MTG1
	MTHFD1
	MTHFD1L
	MTHFR
	MTHFS
	MTIF2
	MTIF3
	MTM1
	MTMR10
	MTMR11
	MTMR12
	MTMR14
	MTMR15
	MTMR2
	MTMR3
	MTMR4
	MTMR6
	MTMR9
	MTO1
	MTP18
	MTPN
	MTR
	MTRF1
	MTRF1L
	MTRR
	MTSS1
	MTSS1L
	MTTP
	MTUS1
	MTX1
	MTX2
	MTX3
	MUC1
	MUC6
	MUDENG
	MUL1
	MUM1
	MUS81
	MUSTN1
	MUT
	MUTED
	MUTYH
	MVD
	MVK
	MVP
	MX1
	MXD1
	MXD4
	MXI1
	MXRA7
	MYADM
	MYBBP1A
	MYC
	MYCBP2
	MYD88
	MYH10

	MYH9
	MYL12A
	MYL5
	MYL6
	MYL6B
	MYLIP
	MYLK
	MYNN
	MYO18A
	MYO1B
	MYO1C
	MYO1D
	MYO3B
	MYO5B
	MYO5C
	MYO6
	MYO7A
	MYO9B
	MYOM1
	MYOM2
	MYPOP
	MYST1
	MYST2
	MYST3
	MZF1
	N4BP1
	N4BP2
	N4BP2L1
	N4BP2L2
	N6AMT1
	N6AMT2
	NAAA
	NAB1
	NAB2
	NACA
	NACA2
	NACAP1
	NACC2
	NADK
	NADSYN1
	NAE1
	NAF1
	NAG18
	NAGA
	NAGK
	NAGLU
	NAGPA
	NAIF1
	NAMPT
	NANOS1
	NANP
	NANS
	NAP1L1
	NAP1L4
	NAP1L5
	NAPA

	NAPB
	NAPEPLD
	NAPG
	NAPRT1
	NARF
	NARFL
	NARG1
	NARG1L
	NARG2
	NARS
	NARS2
	NASP
	NAT1
	NAT10
	NAT12
	NAT13
	NAT14
	NAT15
	NAT5
	NAT6
	NAT8B
	NAT9
	NAV2
	NBAS
	NBEAL2
	NBL1
	NBN
	NBPF1
	NBPF10
	NBPF14
	NBPF20
	NBPF3
	NBPF8
	NCALD
	NCAN
	NCAPD2
	NCAPD3
	NCAPH2
	NCBP1
	NCBP2
	NCDN
	NCK1
	NCK2
	NCKAP1
	NCKIPSD
	NCL
	NCLN
	NCOA1
	NCOA3
	NCOA4
	NCOA5
	NCOA6
	NCOA6IP
	NCOA7
	NCOR1
	NCOR2

	NCRNA00081
	NCRNA00087
	NCRNA00092
	NCRNA00094
	NCRNA00095
	NCRNA00152
	NCRNA00200
	NCRNA00219
	NCSTN
	NDC80
	NDE1
	NDEL1
	NDFIP1
	NDFIP2
	NDN
	NDNL2
	NDRG1
	NDRG2
	NDRG3
	NDRG4
	NDST1
	NDST2
	NDUFA1
	NDUFA10
	NDUFA11
	NDUFA12
	NDUFA13
	NDUFA2
	NDUFA3
	NDUFA4
	NDUFA5
	NDUFA6
	NDUFA7
	NDUFA8
	NDUFA9
	NDUFAB1
	NDUFAF1
	NDUFAF2
	NDUFAF3
	NDUFB10
	NDUFB11
	NDUFB2
	NDUFB3
	NDUFB5
	NDUFB6
	NDUFB7
	NDUFB8
	NDUFB9
	NDUFC1
	NDUFS2
	NDUFS3
	NDUFS4
	NDUFS5
	NDUFS7
	NDUFS8
	NDUFV1

	NDUFV2
	NDUFV3
	NECAB3
	NECAP1
	NECAP2
	NEDD4
	NEDD4L
	NEDD8
	NEDD9
	NEIL2
	NEK1
	NEK2
	NEK3
	NEK6
	NEK8
	NELF
	NENF
	NEO1
	NET1
	NEU1
	NEU4
	NEURL1B
	NEURL2
	NEUROG2
	NFAT5
	NFATC1
	NFATC2IP
	NFATC3
	NFE2L1
	NFE2L2
	NFE2L3
	NFIB
	NFIL3
	NFKB1
	NFKB2
	NFKBIA
	NFKBIB
	NFKBIE
	NFKBIZ
	NFRKB
	NFU1
	NFX1
	NFXL1
	NFYA
	NFYB
	NFYC
	NGDN
	NGEF
	NGFRAP1
	NGLY1
	NGRN
	NHEDC2
	NHLRC2
	NHLRC3
	NHP2
	NHP2L1

	NICN1
	NIF3L1
	NIN
	NINJ1
	NINL
	NIP30
	NIP7
	NIPA1
	NIPA2
	NIPAL1
	NIPBL
	NIPSNAP1
	NIPSNAP3A
	NISCH
	NIT1
	NIT2
	NKAP
	NKD2
	NKIRAS1
	NKIRAS2
	NKRF
	NKTR
	NKX3-1
	NLE1
	NLGN2
	NLN
	NLRP3
	NLRP8
	NLRX1
	NMB
	NMD3
	NME1
	NME1-NME2
	NME2
	NME3
	NME4
	NME6
	NME7
	NMI
	NMNAT1
	NMRAL1
	NMT1
	NMT2
	NNT
	NOB1
	NOC2L
	NOC3L
	NOC4L
	NODAL
	NOL10
	NOL11
	NOL12
	NOL6
	NOL7
	NOL8
	NOLA1

	NOLC1
	NOMO1
	NOMO2
	NONO
	NOP10
	NOP14
	NOP16
	NOP2
	NOP56
	NOP58
	NOSIP
	NOTCH1
	NOTCH2
	NOTCH2NL
	NOXA1
	NOXO1
	NP
	N-PAC
	NPAL3
	NPB
	NPC1
	NPC2
	NPEPL1
	NPEPPS
	NPHP3
	NPIP
	NPLOC4
	NPM3
	NPR2
	NPTN
	NPW
	NPY5R
	NQO1
	NQO2
	NR1D2
	NR1H2
	NR1H3
	NR2C1
	NR2C2AP
	NR2F2
	NR2F6
	NR3C1
	NR3C2
	NR5A2
	NRAS
	NRBF2
	NRBP1
	NRBP2
	NRD1
	NRIP1
	NRIP3
	NRK
	NRM
	NRP1
	NRSN2
	NSA2

	NSBP1
	NSD1
	NSDHL
	NSF
	NSFL1C
	NSL1
	NSMAF
	NSMCE1
	NSMCE2
	NSMCE4A
	NSUN2
	NSUN3
	NSUN4
	NSUN5
	NSUN5B
	NSUN5C
	NSUN6
	NT5C
	NT5C2
	NT5C3
	NT5C3L
	NT5DC1
	NT5DC3
	NTAN1
	NTF3
	NTHL1
	NUAK1
	NUAK2
	NUB1
	NUBP1
	NUBP2
	NUBPL
	NUCB1
	NUCB2
	NUCKS1
	NUDC
	NUDCD2
	NUDCD3
	NUDT1
	NUDT14
	NUDT15
	NUDT16
	NUDT16L1
	NUDT18
	NUDT2
	NUDT21
	NUDT22
	NUDT3
	NUDT5
	NUDT6
	NUDT7
	NUDT8
	NUDT9
	NUFIP1
	NUFIP2
	NUMA1

	NUMB
	NUP107
	NUP133
	NUP153
	NUP155
	NUP160
	NUP188
	NUP205
	NUP214
	NUP35
	NUP37
	NUP43
	NUP50
	NUP54
	NUP62
	NUP85
	NUP88
	NUP93
	NUP98
	NUPL2
	NUPR1
	NUS1
	NUSAP1
	NUTF2
	NVL
	NXF1
	NXN
	NXT1
	NXT2
	NY-REN-7
	OAF
	OAS1
	OAS2
	OAS3
	OAT
	OAZ1
	OAZ2
	OBFC1
	OBFC2A
	OBFC2B
	OCEL1
	OCIAD1
	OCIAD2
	OCRL
	ODC1
	ODF2
	ODF3B
	OFD1
	OGDH
	OGDHL
	OGFOD1
	OGFR
	OGFRL1
	OGG1
	OGT
	OKL38

	OMA1
	OPA1
	OPA3
	OPLAH
	OPN3
	OPRL1
	OPTN
	OR11H1
	OR2A20P
	OR2A42
	OR2A9P
	OR2B2
	OR56B1
	OR7E156P
	OR9A4
	ORAI1
	ORAI3
	ORAOV1
	ORC1L
	ORC2L
	ORC3L
	ORC5L
	ORC6L
	ORMDL1
	ORMDL2
	ORMDL3
	OS9
	OSBP
	OSBPL11
	OSBPL1A
	OSBPL2
	OSBPL6
	OSBPL7
	OSBPL8
	OSBPL9
	OSCP1
	OSGEP
	OSGEPL1
	OSGIN2
	OSR1
	OSTalpha
	OSTC
	OSTCL
	OSTF1
	OSTM1
	OTUB1
	OTUD1
	OTUD4
	OTUD5
	OTUD6B
	OVGP1
	OXA1L
	OXR1
	OXSM
	OXSR1
	OXTR

	P15RS
	P2RX4
	P2RY11
	P4HA1
	P4HA2
	P4HB
	P4HTM
	P704P
	P76
	P8
	PA2G4
	PAAF1
	PABPC1
	PABPC1L
	PABPC3
	PABPC4
	PABPC5
	PABPN1
	PACS1
	PACS2
	PACSIN2
	PACSIN3
	PAF1
	PAFAH1B1
	PAFAH1B2
	PAFAH1B3
	PAFAH2
	PAG1
	PAGE4
	PAH
	PAICS
	PAIP1
	PAIP2
	PAK1
	PAK1IP1
	PAK2
	PAK4
	PALB2
	PALLD
	PALM
	PALMD
	PAM
	PAN2
	PAN3
	PANK1
	PANK2
	PANK3
	PANK4
	PANX1
	PANX2
	PAOX
	PAPD1
	PAPD4
	PAPD5
	PAPLN
	PAPOLA

	PAPSS1
	PAPSS2
	PAQR3
	PAQR4
	PAQR7
	PARD6G
	PARK7
	PARL
	PARN
	PARP1
	PARP10
	PARP11
	PARP12
	PARP14
	PARP16
	PARP2
	PARP3
	PARP4
	PARP6
	PARP8
	PARP9
	PARS2
	PARVA
	PARVB
	PASK
	PATE2
	PATE3
	PATL1
	PAWR
	PAXIP1
	PBLD
	PBRM1
	PBX2
	PBX3
	PC
	PCBD1
	PCBP1
	PCBP2
	PCBP4
	PCCA
	PCCB
	PCDHB19P
	PCDHB9
	PCDHGB6
	PCF11
	PCGF1
	PCGF2
	PCGF6
	PCID2
	PCIF1
	PCK2
	PCM1
	PCMT1
	PCMTD1
	PCMTD2
	PCNA

	PCNP
	PCNT
	PCNX
	PCNXL3
	PCOLCE
	PCOLCE2
	PCSK1N
	PCSK4
	PCSK5
	PCSK6
	PCSK7
	PCTK2
	PCTK3
	PCTP
	PCYOX1
	PCYT2
	PDCD10
	PDCD11
	PDCD2
	PDCD2L
	PDCD4
	PDCD5
	PDCD6
	PDCD6IP
	PDCD7
	PDCL
	PDCL3
	PDDC1
	PDE4A
	PDE4C
	PDE6D
	PDE7A
	PDE8A
	PDE9A
	PDGFC
	PDGFRB
	PDGFRL
	PDHA1
	PDHB
	PDHX
	PDIA3P
	PDIA4
	PDIA5
	PDIA6
	PDIK1L
	PDK2
	PDK3
	PDK4
	PDLIM1
	PDLIM3
	PDLIM5
	PDLIM7
	PDP2
	PDPK1
	PDPR
	PDRG1

	PDS5A
	PDS5B
	PDSS1
	PDSS2
	PDXDC1
	PDXK
	PDXP
	PDZD8
	PDZK1
	PDZK1P1
	PEA15
	PEBP1
	PECI
	PECR
	PEF1
	PELI1
	PELO
	PELP1
	PEMT
	PEPD
	PER2
	PER3
	PERP
	PES1
	PET112L
	PEX1
	PEX10
	PEX11A
	PEX11B
	PEX11G
	PEX13
	PEX14
	PEX16
	PEX19
	PEX5
	PEX6
	PEX7
	PFAAP5
	PFAS
	PFDN1
	PFDN2
	PFDN4
	PFDN5
	PFDN6
	PFKFB3
	PFKFB4
	PFKL
	PFKM
	PFKP
	PFN1
	PFN2
	PFTK1
	PGAM1
	PGAM4
	PGAM5
	PGAP3

	PGBD3
	PGCP
	PGD
	PGK1
	PGLS
	PGM1
	PGM2
	PGM3
	PGP
	PGPEP1
	PGRMC1
	PGRMC2
	PGS1
	PHACTR2
	PHACTR4
	PHAX
	PHB
	PHB2
	PHC1
	PHC2
	PHCA
	PHF1
	PHF10
	PHF11
	PHF12
	PHF13
	PHF14
	PHF15
	PHF16
	PHF17
	PHF19
	PHF2
	PHF20
	PHF20L1
	PHF21A
	PHF23
	PHF3
	PHF5A
	PHF7
	PHGDH
	PHIP
	PHKA1
	PHKA2
	PHKB
	PHKG2
	PHLDA1
	PHLDB1
	PHLDB2
	PHLPP1
	PHLPP2
	PHOSPHO2
	PHPT1
	PHRF1
	PHTF1
	PHYH
	PHYHD1

	PI4K2A
	PI4K2B
	PI4KAP1
	PI4KAP2
	PI4KB
	PIAS1
	PIAS2
	PIAS4
	PIBF1
	PICALM
	PICK1
	PID1
	PIF1
	PIGA
	PIGC
	PIGF
	PIGG
	PIGH
	PIGK
	PIGL
	PIGM
	PIGN
	PIGO
	PIGP
	PIGQ
	PIGS
	PIGT
	PIGU
	PIGV
	PIGW
	PIGX
	PIGY
	PIGZ
	PIH1D1
	PIK3C2A
	PIK3C2B
	PIK3C3
	PIK3CA
	PIK3CB
	PIK3CD
	PIK3IP1
	PIK3R1
	PIK3R2
	PIK3R4
	PIK4CA
	PILRA
	PILRB
	PIM1
	PIM2
	PIM3
	PIN1
	PIN4
	PINK1
	PINX1
	PIP3-E
	PIP4K2A

	PIP4K2C
	PIP5K1C
	PIP5K2B
	PIPSL
	PIR
	PISD
	PITPNA
	PITPNB
	PITPNC1
	PITPNM1
	PITRM1
	PJA1
	PJA2
	PKD1
	PKD2
	PKDCC
	PKIG
	PKM2
	PKN1
	PKN2
	PKNOX1
	PKP2
	PKP4
	PLA2G12B
	PLA2G15
	PLA2G16
	PLA2G2C
	PLA2G2D
	PLA2G4B
	PLA2G6
	PLAA
	PLAG1
	PLAGL2
	PLAU
	PLCB1
	PLCD1
	PLCG1
	PLCG2
	PLCL2
	PLCXD1
	PLD1
	PLD2
	PLD3
	PLD6
	PLDN
	PLEC1
	PLEK2
	PLEKHA1
	PLEKHA2
	PLEKHA4
	PLEKHA5
	PLEKHA6
	PLEKHA7
	PLEKHA9
	PLEKHB2
	PLEKHF1

	PLEKHF2
	PLEKHG3
	PLEKHG6
	PLEKHH3
	PLEKHJ1
	PLEKHM1
	PLEKHM2
	PLEKHN1
	PLEKHO1
	PLEKHO2
	PLIN2
	PLIN5
	PLK2
	PLLP
	PLOD1
	PLOD2
	PLOD3
	PLP1
	PLP2
	PLRG1
	PLS1
	PLS3
	PLSCR3
	PLSCR4
	PLTP
	PLXNA1
	PLXNA3
	PLXNA4
	PLXNB1
	PLXNB2
	PLXND1
	PM20D2
	PMEPA1
	PMF1
	PMM1
	PMM2
	PMP22
	PMPCA
	PMPCB
	PMS2
	PMS2CL
	PMS2L3
	PMS2L4
	PMS2L5
	PMVK
	PNKD
	PNKP
	PNMA1
	PNN
	PNO1
	PNPLA2
	PNPLA4
	PNPLA6
	PNPLA7
	PNPLA8
	PNPO

	PNPT1
	PNRC2
	PODN
	PODXL
	POFUT1
	POFUT2
	POGK
	POL3S
	POLA1
	POLA2
	POLB
	POLD1
	POLD2
	POLD3
	POLD4
	POLDIP2
	POLDIP3
	POLE
	POLE2
	POLE3
	POLE4
	POLG
	POLG2
	POLM
	POLN
	POLR1B
	POLR1C
	POLR1D
	POLR1E
	POLR2A
	POLR2B
	POLR2C
	POLR2D
	POLR2E
	POLR2F
	POLR2G
	POLR2H
	POLR2I
	POLR2J
	POLR2J2
	POLR2J3
	POLR2J4
	POLR2K
	POLR2L
	POLR3A
	POLR3B
	POLR3C
	POLR3E
	POLR3F
	POLR3G
	POLR3GL
	POLR3H
	POLR3K
	POLRMT
	POLS
	POM121C

	POMGNT1
	POMP
	POMT1
	POMT2
	PON2
	POP1
	POP4
	POP5
	POP7
	POR
	POTEF
	PP14571
	PPA1
	PPA2
	PPAN
	PPAP2A
	PPAP2B
	PPAP2C
	PPAPDC1B
	PPAPDC2
	PPARA
	PPARBP
	PPARD
	PPARG
	PPARGC1A
	PPAT
	PPBP
	PPCS
	PPDPF
	PPFIA1
	PPFIBP1
	PPFIBP2
	PPHLN1
	PPIA
	PPIAL4A
	PPIB
	PPIC
	PPIE
	PPIG
	PPIH
	PPIL1
	PPIL3
	PPIL5
	PPL
	PPM1A
	PPM1B
	PPM1D
	PPM1F
	PPM1G
	PPM1H
	PPM1K
	PPM1M
	PPM2C
	PPME1
	PPOX
	PPP1CA

	PPP1CB
	PPP1CC
	PPP1R10
	PPP1R11
	PPP1R12A
	PPP1R12B
	PPP1R12C
	PPP1R13B
	PPP1R13L
	PPP1R14B
	PPP1R15A
	PPP1R15B
	PPP1R16A
	PPP1R1A
	PPP1R1C
	PPP1R2
	PPP1R3C
	PPP1R3E
	PPP1R7
	PPP1R8
	PPP2CA
	PPP2CB
	PPP2R1A
	PPP2R1B
	PPP2R2A
	PPP2R2B
	PPP2R2D
	PPP2R3A
	PPP2R3C
	PPP2R4
	PPP2R5A
	PPP2R5B
	PPP2R5C
	PPP2R5D
	PPP2R5E
	PPP3CA
	PPP3CB
	PPP3CC
	PPP3R1
	PPP4C
	PPP4R1
	PPP4R4
	PPP6C
	PPPDE1
	PPPDE2
	PPRC1
	PPT1
	PPT2
	PPTC7
	PPWD1
	PQBP1
	PQLC1
	PQLC3
	PRAF2
	PRAGMIN
	PRAMEF13

	PRAMEF7
	PRC1
	PRCC
	PRCP
	PRDM10
	PRDM4
	PRDX1
	PRDX2
	PRDX3
	PRDX4
	PRDX5
	PRDX6
	PREB
	PREI3
	PRELID1
	PREP
	PREPL
	PRIC285
	PRICKLE2
	PRICKLE4
	PRIM1
	PRIM2A
	PRKAA1
	PRKAB1
	PRKAB2
	PRKAG1
	PRKAG2
	PRKAR1A
	PRKAR2A
	PRKCA
	PRKCABP
	PRKCD
	PRKCDBP
	PRKCH
	PRKCI
	PRKCSH
	PRKCZ
	PRKD1
	PRKD2
	PRKD3
	PRKDC
	PRKRA
	PRKRIP1
	PRKRIR
	PRLR
	PRMT1
	PRMT10
	PRMT2
	PRMT3
	PRMT5
	PRMT6
	PRMT7
	PRNP
	PRNPIP
	PRO0628
	PRO1853

	PROCR
	PROS1
	ProSAPiP1
	PROSC
	PROX1
	PRPF18
	PRPF19
	PRPF3
	PRPF31
	PRPF38A
	PRPF38B
	PRPF4
	PRPF40A
	PRPF4B
	PRPF6
	PRPF8
	PRPS1
	PRPS2
	PRPSAP1
	PRPSAP2
	PRR13
	PRR14
	PRR15L
	PRR19
	PRR3
	PRR5
	PRRC1
	PRRG1
	PRRG2
	PRRG4
	PRRT3
	PRSS23
	PRSS7
	PRSS8
	PRUNE
	PSAP
	PSAT1
	PSCD1
	PSCD2
	PSD3
	PSD4
	PSEN1
	PSEN2
	PSENEN
	PSG3
	PSG4
	PSIP1
	PSKH1
	PSMA1
	PSMA2
	PSMA3
	PSMA4
	PSMA5
	PSMA6
	PSMB1
	PSMB10

	PSMB2
	PSMB3
	PSMB4
	PSMB5
	PSMB6
	PSMB7
	PSMB8
	PSMC1
	PSMC2
	PSMC3
	PSMC3IP
	PSMC4
	PSMC5
	PSMC6
	PSMD1
	PSMD10
	PSMD11
	PSMD12
	PSMD13
	PSMD14
	PSMD2
	PSMD3
	PSMD4
	PSMD5
	PSMD6
	PSMD7
	PSMD8
	PSMD9
	PSME1
	PSME2
	PSME3
	PSME4
	PSMF1
	PSMG1
	PSMG2
	PSMG3
	PSMG4
	PSPC1
	PSPH
	PSTK
	PSTPIP2
	PTAR1
	PTBP1
	PTBP2
	PTCD1
	PTCD2
	PTDSS1
	PTDSS2
	PTEN
	PTGES2
	PTGES3
	PTGFRN
	PTGR1
	PTGR2
	PTK2
	PTK2B

	PTMA
	PTMS
	PTOV1
	PTP4A1
	PTP4A2
	PTPLA
	PTPLAD1
	PTPLAD2
	PTPLB
	PTPMT1
	PTPN1
	PTPN11
	PTPN12
	PTPN2
	PTPN23
	PTPN3
	PTPN4
	PTPN6
	PTPN7
	PTPN9
	PTPRA
	PTPRD
	PTPRE
	PTPRF
	PTPRG
	PTPRK
	PTPRM
	PTPRU
	PTRF
	PTRH1
	PTRH2
	PTS
	PTTG1
	PTTG1IP
	PUF60
	PUM1
	PUM2
	PURA
	PURB
	PURG
	PUS1
	PUS3
	PUS7
	PUSL1
	PVR
	PVRL1
	PVRL2
	PVRL3
	PWP1
	PWWP2B
	PXDN
	PXK
	PXMP2
	PXMP3
	PXMP4
	PXN

	PYCARD
	PYCR1
	PYCR2
	PYGB
	PYGL
	QARS
	QDPR
	QKI
	QPCT
	QPRT
	QRFPR
	QRICH1
	QSER1
	QSOX1
	QSOX2
	QTRT1
	QTRTD1
	R3HCC1
	R3HDM1
	R3HDM2
	RAB10
	RAB11A
	RAB11FIP1
	RAB11FIP2
	RAB11FIP3
	RAB11FIP5
	RAB12
	RAB13
	RAB14
	RAB15
	RAB17
	RAB18
	RAB1A
	RAB1B
	RAB20
	RAB21
	RAB22A
	RAB23
	RAB24
	RAB28
	RAB2A
	RAB2B
	RAB31
	RAB32
	RAB33B
	RAB35
	RAB37
	RAB3GAP1
	RAB3GAP2
	RAB3IL1
	RAB3IP
	RAB40B
	RAB40C
	RAB43
	RAB4A
	RAB4B

	RAB5A
	RAB5B
	RAB5C
	RAB6A
	RAB6B
	RAB7A
	RAB8A
	RAB8B
	RAB9A
	RABAC1
	RABEP1
	RABEPK
	RABGAP1
	RABGAP1L
	RABGEF1
	RABGGTA
	RABGGTB
	RABIF
	RABL2A
	RABL2B
	RABL3
	RABL4
	RAC1
	RAC2
	RAC3
	RAD1
	RAD17
	RAD21
	RAD23A
	RAD23B
	RAD50
	RAD51
	RAD51C
	RAD51L1
	RAD51L3
	RAD54L2
	RAD9A
	RAE1
	RAF1
	RAG1AP1
	RAGE
	RAI1
	RAI14
	RALA
	RALB
	RALBP1
	RALGAPA1
	RALGAPB
	RALGDS
	RALGPS1
	RALY
	RAN
	RANBP1
	RANBP10
	RANBP2
	RANBP3

	RANBP6
	RANBP9
	RANGAP1
	RANGRF
	RAP1B
	RAP1BL
	RAP1GAP
	RAP1GDS1
	RAP2A
	RAP2C
	RAPGEF1
	RAPGEF2
	RAPGEF5
	RAPGEF6
	RAPGEFL1
	RAPH1
	RARA
	RARRES1
	RARRES2
	RARRES3
	RARS
	RARS2
	RASA1
	RASA4P
	RASAL3
	RASD1
	RASGRP2
	RASIP1
	RASL11A
	RASSF1
	RASSF2
	RASSF4
	RASSF5
	RASSF6
	RASSF7
	RAVER1
	RAXL1
	RB1CC1
	RBBP4
	RBBP5
	RBBP6
	RBBP7
	RBBP8
	RBBP9
	RBCK1
	RBED1
	RBKS
	RBL2
	RBM10
	RBM12
	RBM12B
	RBM14
	RBM15
	RBM15B
	RBM16
	RBM17

	RBM18
	RBM22
	RBM23
	RBM25
	RBM26
	RBM27
	RBM28
	RBM3
	RBM33
	RBM34
	RBM38
	RBM39
	RBM4
	RBM41
	RBM42
	RBM45
	RBM47
	RBM4B
	RBM5
	RBM6
	RBM7
	RBM9
	RBMS1
	RBMX
	RBMX2
	RBMY2FP
	RBP1
	RBP4
	RBP5
	RBP7
	RBPJ
	RBPMS
	RBPMS2
	RBX1
	RC3H2
	RCADH5
	RCAN1
	RCBTB2
	RCC2
	RCCD1
	RCE1
	RCHY1
	RCL1
	RCN1
	RCN2
	RCN3
	RCOR3
	RDBP
	RDH10
	RDH11
	RDH13
	RDH14
	RDH16
	RDH5
	RDX
	REC8

	RECQL
	REEP4
	REEP5
	REEP6
	RELA
	RELL1
	REPIN1
	REPS2
	RER1
	RERE
	RETSAT
	REV1
	REXO1
	REXO2
	REXO4
	RFC1
	RFC2
	RFC3
	RFC4
	RFC5
	RFESD
	RFFL
	RFK
	RFNG
	RFP
	RFPL4A
	RFTN1
	RFWD2
	RFWD3
	RFX1
	RFX4
	RFX5
	RFX7
	RFXANK
	RG9MTD1
	RG9MTD2
	RG9MTD3
	RGL1
	RGL2
	RGL4
	RGMA
	RGN
	RGS10
	RGS12
	RGS19
	RGS2
	RHBDD1
	RHBDD2
	RHBDD3
	RHBDF1
	RHBDF2
	RHBDL1
	RHBDL2
	RHEB
	RHOA
	RHOB

	RHOBTB1
	RHOBTB3
	RHOC
	RHOD
	RHOG
	RHOQ
	RHOT1
	RHOT2
	RHOU
	RHPN2
	RIC8A
	RIC8B
	RICH2
	RICS
	RIF1
	RILPL1
	RILPL2
	RIMBP3
	RIN2
	RING1
	RINT1
	RIOK1
	RIOK2
	RIOK3
	RIPK1
	RIPK2
	RIPK4
	RIPK5
	RIT1
	RLF
	RMI1
	RMND1
	RMND5A
	RMND5B
	RN5S9
	RN7SK
	RN7SL1
	RNASE1
	RNASE10
	RNASE4
	RNASEH1
	RNASEH2A
	RNASEH2B
	RNASEK
	RNASEL
	RNASEN
	RNASET2
	RND2
	RND3
	RNF10
	RNF103
	RNF11
	RNF112
	RNF113A
	RNF114
	RNF115

	RNF121
	RNF122
	RNF123
	RNF126
	RNF128
	RNF13
	RNF130
	RNF135
	RNF138
	RNF14
	RNF141
	RNF144
	RNF144B
	RNF145
	RNF146
	RNF149
	RNF160
	RNF165
	RNF166
	RNF167
	RNF169
	RNF170
	RNF181
	RNF185
	RNF187
	RNF19A
	RNF19B
	RNF20
	RNF213
	RNF214
	RNF215
	RNF216
	RNF216L
	RNF219
	RNF220
	RNF24
	RNF25
	RNF26
	RNF31
	RNF32
	RNF34
	RNF38
	RNF4
	RNF40
	RNF41
	RNF44
	RNF5
	RNF5P1
	RNF7
	RNF8
	RNFT1
	RNGTT
	RNH1
	RNMT
	RNMTL1
	RNPC3

	RNPEP
	RNPEPL1
	RNPS1
	RNU1-3
	RNU1-5
	RNU1A3
	RNU1F1
	RNU1G2
	RNU6-1
	RNU6-15
	RNY1
	RNY3
	ROBLD3
	ROBO3
	ROCK1
	ROCK2
	ROD1
	ROGDI
	ROM1
	ROMO1
	RORA
	RP11-49G10.8
	RP11-529I10.4
	RP5-1022P6.2
	RP9
	RP9P
	RPA1
	RPA2
	RPA3
	RPAIN
	RPAP1
	RPAP2
	RPAP3
	RPE
	RPF1
	RPF2
	RPGR
	RPH3AL
	RPIA
	RPL10A
	RPL11
	RPL12
	RPL12P6
	RPL13
	RPL13A
	RPL13L
	RPL14
	RPL14L
	RPL15
	RPL17
	RPL18
	RPL18A
	RPL19
	RPL21
	RPL22
	RPL23

	RPL23A
	RPL23AP13
	RPL23AP53
	RPL23AP7
	RPL24
	RPL26
	RPL26L1
	RPL27
	RPL27A
	RPL28
	RPL29
	RPL29P2
	RPL3
	RPL30
	RPL31
	RPL31P10
	RPL32
	RPL34
	RPL35
	RPL35A
	RPL36
	RPL36A
	RPL36AL
	RPL37
	RPL37A
	RPL38
	RPL39
	RPL39L
	RPL4
	RPL41
	RPL5
	RPL6
	RPL7
	RPL7A
	RPL7L1
	RPL8
	RPL9
	RPLP0
	RPLP1
	RPLP2
	RPN1
	RPN2
	RPP14
	RPP21
	RPP38
	RPP40
	RPRC1
	RPRD1A
	RPRD1B
	RPRD2
	RPS10
	RPS10P3
	RPS11
	RPS12
	RPS13
	RPS14

	RPS15
	RPS15A
	RPS16
	RPS17
	RPS18
	RPS19
	RPS19BP1
	RPS2
	RPS20
	RPS21
	RPS23
	RPS24
	RPS25
	RPS26
	RPS26L
	RPS26P10
	RPS26P11
	RPS27
	RPS27A
	RPS27L
	RPS28
	RPS29
	RPS3
	RPS3A
	RPS4X
	RPS4Y1
	RPS4Y2
	RPS5
	RPS6
	RPS6KA1
	RPS6KA2
	RPS6KA3
	RPS6KA4
	RPS6KA5
	RPS6KB1
	RPS6KB2
	RPS6KC1
	RPS6KL1
	RPS6P1
	RPS7
	RPS8
	RPS9
	RPSA
	RPUSD1
	RPUSD2
	RPUSD3
	RPUSD4
	RQCD1
	RRAGA
	RRAGB
	RRAGC
	RRAGD
	RRAS
	RRAS2
	RRBP1
	RREB1

	RRM1
	RRM2B
	RRN3
	RRP12
	RRP15
	RRP1B
	RRP7A
	RRP7B
	RRP8
	RRP9
	RRS1
	RSAD1
	RSBN1
	RSC1A1
	RSL1D1
	RSL24D1
	RSPH3
	RSPH9
	RSPRY1
	RSRC1
	RSRC2
	RSU1
	RTCD1
	RTEL1
	RTF1
	RTKN
	RTN3
	RTN4
	RTN4IP1
	RTTN
	RUFY1
	RUFY3
	RUNDC1
	RUNDC2C
	RUNX1
	RUNX1T1
	RUNX2
	RUNX3
	RUSC1
	RUSC2
	RUVBL1
	RUVBL2
	RWDD1
	RWDD2A
	RWDD2B
	RWDD3
	RWDD4A
	RXRA
	RXRB
	RXRG
	RYBP
	RYK
	S100A10
	S100A11
	S100A13
	S100A14

	S100A16
	S100A4
	S100A6
	S100A9
	S100P
	S100PBP
	SAAL1
	SAC3D1
	SACM1L
	SAE1
	SAFB
	SAFB2
	SAMD11
	SAMD4A
	SAMD4B
	SAMD6
	SAMM50
	SAP130
	SAP18
	SAP30
	SAP30BP
	SAP30L
	SAPS1
	SAPS2
	SAPS3
	SAR1A
	SAR1B
	SARS
	SARS2
	SART3
	SASH1
	SAT1
	SAT2
	SATB2
	SAV1
	SBDS
	SBDSP
	SBF1
	SBF2
	SBK1
	SBNO2
	SC4MOL
	SC5DL
	SC65
	SCAMP1
	SCAMP2
	SCAMP3
	SCAMP4
	SCAND1
	SCAP
	SCAPER
	SCARB1
	SCARB2
	SCARNA10
	SCARNA13
	SCARNA9

	SCCPDH
	SCD
	SCFD1
	SCFD2
	SCG5
	SCGB3A2
	SCHIP1
	SCLY
	SCMH1
	SCML1
	SCML2
	SCNM1
	SCNN1A
	SCNN1D
	SCO1
	SCO2
	SCOC
	SCP2
	SCPEP1
	SCRIB
	SCRN2
	SCYL1
	SCYL1BP1
	SCYL2
	SCYL3
	SDAD1
	SDC1
	SDC2
	SDC3
	SDC4
	SDCBP
	SDCCAG1
	SDCCAG10
	SDCCAG3
	SDF2
	SDF2L1
	SDF4
	SDHA
	SDHAF1
	SDHAF2
	SDHALP1
	SDHAP2
	SDHAP3
	SDHB
	SDHC
	SDHD
	SDSL
	SEC11A
	SEC11C
	SEC13
	SEC14L1
	SEC16A
	SEC22A
	SEC22B
	SEC22C
	SEC23A

	SEC23B
	SEC23IP
	SEC24A
	SEC24B
	SEC24C
	SEC24D
	SEC31A
	SEC61A1
	SEC61B
	SEC61G
	SEC62
	SEC63
	SECISBP2
	SECISBP2L
	SEH1L
	SEL1L
	SEL1L3
	SELENBP1
	SELI
	SELK
	SELM
	SELO
	SELS
	SELT
	SEMA3E
	SEMA4B
	SEMA4C
	SEMA4F
	SEMA4G
	SEMA6B
	SEMA6C
	SENP2
	SENP5
	SENP6
	SENP7
	SEP15
	SEPHS1
	SEPHS2
	SEPN1
	SEPP1
	SEPSECS
	SEPT11
	SEPT2
	SEPT4
	SEPT5
	SEPT6
	SEPT7
	SEPT9
	SEPW1
	SEPX1
	SERAC1
	SERBP1
	SERF1B
	SERF2
	SERGEF
	SERINC1

	SERINC2
	SERINC3
	SERP1
	SERP2
	SERPINA1
	SERPINA10
	SERPINA3
	SERPINA5
	SERPINB1
	SERPINB6
	SERPINB8
	SERPINC1
	SERPINE1
	SERPINE2
	SERPINF1
	SERPINF2
	SERPING1
	SERPINH1
	SERTAD1
	SERTAD2
	SERTAD3
	SESN1
	SESN2
	SESTD1
	SET
	SETBP1
	SETD1A
	SETD1B
	SETD2
	SETD3
	SETD4
	SETD5
	SETD6
	SETD8
	SETDB1
	SETDB2
	SETMAR
	SETX
	SF1
	SF3A1
	SF3A2
	SF3A3
	SF3B1
	SF3B14
	SF3B2
	SF3B3
	SF3B4
	SF3B5
	SF4
	SFMBT1
	SFPQ
	SFRS1
	SFRS10
	SFRS11
	SFRS12
	SFRS13A

	SFRS14
	SFRS15
	SFRS16
	SFRS17A
	SFRS18
	SFRS2
	SFRS2B
	SFRS2IP
	SFRS3
	SFRS4
	SFRS5
	SFRS6
	SFRS7
	SFRS8
	SFRS9
	SFT2D1
	SFT2D2
	SFT2D3
	SFXN1
	SFXN2
	SFXN4
	SFXN5
	SGCE
	SGK
	SGK1
	SGK3
	SGMS1
	SGPL1
	SGSH
	SGSM2
	SGSM3
	SGTA
	SH2B1
	SH2B3
	SH2D1A
	SH2D4A
	SH2D5
	SH3BGRL
	SH3BGRL2
	SH3BGRL3
	SH3BP4
	SH3BP5L
	SH3D19
	SH3GL1
	SH3GLB1
	SH3GLB2
	SH3KBP1
	SH3PXD2A
	SH3RF1
	SH3RF2
	SH3TC1
	SH3YL1
	SHANK2
	SHANK3
	SHARPIN
	SHB

	SHBG
	SHC1
	SHC2
	SHCBP1
	SHF
	SHFM1
	SHISA5
	SHKBP1
	SHMT1
	SHMT2
	SHOC2
	SHPK
	SHQ1
	SHRM
	SHROOM2
	SHROOM3
	SHROOM4
	SIAE
	SIAH1
	SIAH2
	SIDT2
	SIGIRR
	SIGLEC6
	SIGMAR1
	SIK1
	SIK2
	SIK3
	SIL1
	SILV
	SIN3A
	SIN3B
	SIP1
	SIPA1
	SIPA1L1
	SIPA1L2
	SIPA1L3
	SIRPA
	SIRT1
	SIRT2
	SIRT4
	SIRT5
	SIRT7
	SIVA
	SIVA1
	SIX5
	SKA2
	SKAP2
	SKI
	SKIV2L
	SKIV2L2
	SKP1
	SKP1A
	SKP2
	SLAIN1
	SLAIN2
	SLBP

	SLC10A1
	SLC10A3
	SLC10A7
	SLC11A2
	SLC12A2
	SLC12A4
	SLC12A8
	SLC12A9
	SLC13A5
	SLC15A1
	SLC15A3
	SLC15A4
	SLC16A10
	SLC16A12
	SLC16A2
	SLC16A4
	SLC16A5
	SLC19A1
	SLC19A2
	SLC19A3
	SLC1A4
	SLC20A1
	SLC20A2
	SLC22A1
	SLC22A17
	SLC22A18
	SLC22A18AS
	SLC22A23
	SLC22A5
	SLC22A7
	SLC23A1
	SLC23A2
	SLC23A3
	SLC24A1
	SLC24A2
	SLC24A6
	SLC25A1
	SLC25A10
	SLC25A11
	SLC25A12
	SLC25A13
	SLC25A14
	SLC25A15
	SLC25A16
	SLC25A17
	SLC25A18
	SLC25A19
	SLC25A20
	SLC25A22
	SLC25A23
	SLC25A25
	SLC25A26
	SLC25A28
	SLC25A29
	SLC25A3
	SLC25A34

	SLC25A37
	SLC25A38
	SLC25A39
	SLC25A4
	SLC25A40
	SLC25A42
	SLC25A43
	SLC25A44
	SLC25A45
	SLC25A46
	SLC25A5
	SLC25A6
	SLC26A11
	SLC26A2
	SLC26A6
	SLC27A1
	SLC27A2
	SLC27A3
	SLC27A5
	SLC29A1
	SLC29A2
	SLC29A3
	SLC29A4
	SLC2A1
	SLC2A10
	SLC2A11
	SLC2A12
	SLC2A3
	SLC2A4RG
	SLC2A8
	SLC30A1
	SLC30A3
	SLC30A5
	SLC30A7
	SLC30A9
	SLC31A1
	SLC31A2
	SLC33A1
	SLC35A1
	SLC35A2
	SLC35A3
	SLC35A4
	SLC35A5
	SLC35B1
	SLC35B2
	SLC35B3
	SLC35C1
	SLC35C2
	SLC35D2
	SLC35E1
	SLC35E3
	SLC35F5
	SLC36A1
	SLC36A4
	SLC37A1
	SLC37A3

	SLC37A4
	SLC38A1
	SLC38A10
	SLC38A2
	SLC38A5
	SLC38A6
	SLC38A7
	SLC38A9
	SLC39A1
	SLC39A11
	SLC39A14
	SLC39A3
	SLC39A4
	SLC39A5
	SLC39A6
	SLC39A7
	SLC39A8
	SLC39A9
	SLC3A2
	SLC40A1
	SLC41A1
	SLC41A3
	SLC43A1
	SLC43A3
	SLC44A1
	SLC44A2
	SLC44A3
	SLC44A4
	SLC45A3
	SLC46A3
	SLC47A1
	SLC48A1
	SLC4A1AP
	SLC4A2
	SLC4A4
	SLC4A5
	SLC4A7
	SLC5A10
	SLC5A12
	SLC5A6
	SLC5A8
	SLC5A9
	SLC6A10P
	SLC6A12
	SLC6A16
	SLC6A9
	SLC7A1
	SLC7A2
	SLC7A3
	SLC7A5
	SLC7A6
	SLC7A6OS
	SLC7A7
	SLC7A9
	SLC9A1
	SLC9A3R1

	SLC9A6
	SLC9A7
	SLC9A8
	SLCO1B1
	SLCO2B1
	SLCO4A1
	SLCO4C1
	SLFN11
	SLK
	SLMAP
	SLTM
	SLU7
	SMA4
	SMAD2
	SMAD3
	SMAD4
	SMAD5
	SMAD7
	SMAGP
	SMAP1
	SMAP2
	SMARCA1
	SMARCA2
	SMARCA4
	SMARCA5
	SMARCAD1
	SMARCAL1
	SMARCB1
	SMARCC1
	SMARCC2
	SMARCD1
	SMARCD2
	SMARCE1
	SMC2
	SMC3
	SMC4
	SMCR5
	SMCR7L
	SMEK2
	SMG1
	SMG5
	SMG7
	SMN1
	SMNDC1
	SMO
	SMOX
	SMPD1
	SMPD2
	SMPDL3A
	SMS
	SMTN
	SMU1
	SMUG1
	SMURF1
	SMYD2
	SMYD3

	SMYD4
	SNAI2
	SNAP23
	SNAP29
	SNAP47
	SNAPC1
	SNAPC2
	SNAPC3
	SNAPC4
	SNAPC5
	SNAPIN
	SND1
	SNF8
	SNHG1
	SNHG11
	SNHG12
	SNHG3-RCC1
	SNHG5
	SNHG6
	SNHG7
	SNHG8
	SNHG9
	SNIP1
	SNN
	SNORA10
	SNORA11D
	SNORA12
	SNORA18
	SNORA24
	SNORA25
	SNORA32
	SNORA33
	SNORA41
	SNORA45
	SNORA57
	SNORA58
	SNORA59A
	SNORA59B
	SNORA5C
	SNORA6
	SNORA61
	SNORA62
	SNORA67
	SNORA68
	SNORA70
	SNORA70C
	SNORA76
	SNORA7B
	SNORA8
	SNORA80
	SNORD104
	SNORD114-2
	SNORD14A
	SNORD22
	SNORD31
	SNORD35B

	SNORD36A
	SNORD36C
	SNORD3A
	SNORD3C
	SNORD3D
	SNORD49A
	SNORD4A
	SNORD57
	SNORD6
	SNORD65
	SNORD68
	SNORD78
	SNORD83B
	SNORD87
	SNORD89
	SNORD99
	SNRK
	SNRNP200
	SNRNP25
	SNRNP27
	SNRNP35
	SNRNP40
	SNRNP48
	SNRNP70
	SNRPA
	SNRPA1
	SNRPB
	SNRPB2
	SNRPC
	SNRPD1
	SNRPD2
	SNRPD3
	SNRPF
	SNRPG
	SNRPN
	SNTA1
	SNTB1
	SNTB2
	SNUPN
	SNURF
	SNW1
	SNX1
	SNX10
	SNX11
	SNX13
	SNX14
	SNX16
	SNX17
	SNX19
	SNX2
	SNX21
	SNX22
	SNX24
	SNX25
	SNX26
	SNX27

	SNX29
	SNX3
	SNX30
	SNX33
	SNX4
	SNX5
	SNX6
	SNX7
	SNX8
	SOAT2
	SOCS1
	SOCS2
	SOCS3
	SOCS4
	SOCS5
	SOCS6
	SOD1
	SOD2
	SOLH
	SON
	SORBS1
	SORBS2
	SORBS3
	SORD
	SORL1
	SORT1
	SOX13
	SOX18
	SOX4
	SOX7
	SOX8
	SOX9
	SP1
	SP110
	SP140
	SP2
	SP3
	SP4
	SPA17
	SPAG16
	SPAG7
	SPAG9
	SPARC
	SPARCL1
	SPAST
	SPATA13
	SPATA2
	SPATA20
	SPATA2L
	SPATA5L1
	SPATA7
	SPATC1
	SPATS2
	SPATS2L
	SPC24
	SPCS1

	SPCS2
	SPCS3
	SPEN
	SPG11
	SPG21
	SPG3A
	SPG7
	SPHAR
	SPHK1
	SPHK2
	SPIN1
	SPIN3
	SPIN4
	SPINK1
	SPINT2
	SPINT3
	SPIRE1
	SPIRE2
	SPN
	SPNS1
	SPNS2
	SPOP
	SPOPL
	SPP1
	SPPL2A
	SPPL2B
	SPPL3
	SPR
	SPRED1
	SPRED2
	SPRN
	SPRR1A
	SPRY1
	SPRY2
	SPRY4
	SPRYD3
	SPRYD4
	SPSB1
	SPSB2
	SPSB3
	SPTAN1
	SPTBN1
	SPTBN2
	SPTLC1
	SPTLC3
	SPTY2D1
	SQLE
	SQSTM1
	SR140
	SRA1
	SRBD1
	SRC
	SRCAP
	SRD5A1
	SREBF1
	SREBF2

	SRF
	SRFBP1
	SRGAP2
	SRGAP3
	SRI
	SRM
	SRP14
	SRP14P1
	SRP19
	SRP54
	SRP68
	SRP72
	SRP9
	SRPK1
	SRPK2
	SRPR
	SRPRB
	SRPX2
	SRRD
	SRRM1
	SRRM1L
	SRRM2
	SRXN1
	SS18
	SS18L1
	SS18L2
	SSB
	SSBP1
	SSBP2
	SSBP3
	SSBP4
	SSFA2
	SSH2
	SSH3
	SSNA1
	SSPO
	SSR1
	SSR2
	SSR3
	SSR4
	SSRP1
	SSSCA1
	SSTR2
	SSU72
	ST13
	ST3GAL1
	ST3GAL2
	ST3GAL3
	ST3GAL4
	ST3GAL5
	ST3GAL6
	ST5
	ST6GAL1
	ST6GALNAC2
	ST6GALNAC4
	ST6GALNAC6

	ST7
	ST7L
	STAG1
	STAG2
	STAG3L1
	STAG3L2
	STAG3L3
	STAG3L4
	STAM
	STAM2
	STAMBP
	STAMBPL1
	STAP2
	STARD10
	STARD13
	STARD3
	STARD3NL
	STARD5
	STARD7
	STARD8
	STAT1
	STAT2
	STAT3
	STAT4
	STAT5A
	STAT5B
	STAT6
	STAU1
	STAU2
	STBD1
	STEAP1
	STEAP3
	STIL
	STIM1
	STIM2
	STIP1
	STK10
	STK11
	STK11IP
	STK16
	STK17B
	STK19
	STK24
	STK25
	STK3
	STK32C
	STK35
	STK36
	STK38
	STK39
	STK4
	STK40
	STMN1
	STMN3
	STOM
	STOML1

	STOML2
	STOX2
	STRA13
	STRADA
	STRADB
	STRAP
	STRBP
	STRN
	STRN3
	STRN4
	STS-1
	STT3A
	STT3B
	STUB1
	STX10
	STX12
	STX16
	STX1A
	STX2
	STX3
	STX4
	STX5
	STX6
	STX7
	STX8
	STXBP2
	STXBP3
	STXBP6
	STYXL1
	SUB1
	SUCLA2
	SUCLG1
	SUCLG2
	SUGT1
	SULF2
	SULT1A1
	SULT1A2
	SULT1A3
	SULT1A4
	SULT2A1
	SUMF1
	SUMF2
	SUMO1
	SUMO1P3
	SUMO2
	SUMO3
	SUOX
	SUPT16H
	SUPT4H1
	SUPT5H
	SUPT6H
	SUPV3L1
	SURF1
	SURF2
	SURF4
	SURF6

	SUSD1
	SUSD3
	SUV39H1
	SUV39H2
	SUV420H1
	SUV420H2
	SUZ12
	SVIL
	SWAP70
	SYAP1
	SYCE1L
	SYDE2
	SYF2
	SYMPK
	SYNCRIP
	SYNE2
	SYNGR1
	SYNJ1
	SYNJ2BP
	SYNM
	SYPL1
	SYS1
	SYT11
	SYT15
	SYT17
	SYT7
	SYTL2
	SYVN1
	TACC1
	TACC2
	TACO1
	TACSTD1
	TACSTD2
	TADA1L
	TADA2B
	TADA3
	TAF10
	TAF12
	TAF15
	TAF1B
	TAF1C
	TAF1D
	TAF2
	TAF4
	TAF5L
	TAF6
	TAF6L
	TAF7
	TAF9
	TAGLN
	TAGLN2
	TALDO1
	TANC1
	TANK
	TAOK1
	TAOK2

	TAOK3
	TAP1
	TAPBP
	TAPT1
	TARBP2
	TARDBP
	TARS
	TARS2
	TASP1
	TATDN1
	TATDN2
	TATDN3
	TAX1BP1
	TAX1BP3
	TAZ
	TBC1D10A
	TBC1D10B
	TBC1D13
	TBC1D14
	TBC1D15
	TBC1D16
	TBC1D17
	TBC1D19
	TBC1D2
	TBC1D20
	TBC1D21
	TBC1D22A
	TBC1D23
	TBC1D24
	TBC1D25
	TBC1D2B
	TBC1D3B
	TBC1D3F
	TBC1D3G
	TBC1D3I
	TBC1D4
	TBC1D7
	TBC1D8
	TBC1D8B
	TBC1D9
	TBC1D9B
	TBCA
	TBCB
	TBCC
	TBCCD1
	TBCD
	TBCE
	TBK1
	TBL1X
	TBL1XR1
	TBL2
	TBL3
	TBP
	TBPL1
	TBRG4
	TBX21

	TBX3
	TCEA1
	TCEA2
	TCEA3
	TCEAL1
	TCEAL3
	TCEAL4
	TCEAL8
	TCEB1
	TCEB2
	TCEB3
	TCERG1
	TCF12
	TCF2
	TCF20
	TCF25
	TCF3
	TCF7L1
	TCFL5
	TCHP
	TCIRG1
	TCN2
	TCP1
	TCP10L
	TCP11L1
	TCTA
	TCTEX1D2
	TCTN1
	TCTN3
	TDG
	TDO2
	TDP1
	TDRD1
	TDRD3
	TDRD7
	TEAD2
	TEAD3
	TECPR1
	TECR
	TEF
	TELO2
	TERF1
	TERF2
	TERF2IP
	TESK1
	TESK2
	TEX10
	TEX2
	TEX261
	TEX264
	TF
	TFAM
	TFAMP1
	TFB1M
	TFB2M
	TFCP2

	TFDP1
	TFDP2
	TFE3
	TFEC
	TFG
	TFIP11
	TFPI
	TFPT
	TFRC
	TGDS
	TGFB1I1
	TGFB3
	TGFBI
	TGFBR2
	TGFBR3
	TGFBRAP1
	TGIF1
	TGIF2
	TGM2
	TGM3
	TGOLN2
	TH1L
	THADA
	THAP1
	THAP10
	THAP11
	THAP6
	THAP7
	THAP8
	THBS1
	THBS3
	THEM2
	THNSL1
	THNSL2
	THOC1
	THOC2
	THOC3
	THOC4
	THOC5
	THOC6
	THOC7
	THOP1
	THRA
	THRAP3
	THRAP5
	THUMPD1
	THUMPD2
	THUMPD3
	THYN1
	TIA1
	TIAF1
	TIAL1
	TICAM1
	TICAM2
	TIFA
	TIGA1

	TIGD2
	TIGD5
	TIGD6
	TIMELESS
	TIMM10
	TIMM17B
	TIMM22
	TIMM23
	TIMM23B
	TIMM44
	TIMM8A
	TIMM8B
	TIMM9
	TIMP1
	TIMP2
	TIMP3
	TINF2
	TINP1
	TIPARP
	TIPIN
	TIPRL
	TIRAP
	TJAP1
	TJP1
	TJP2
	TJP3
	TK1
	TK2
	TKT
	TLCD1
	TLE1
	TLE2
	TLE4
	TLK1
	TLK2
	TLN1
	TLN2
	TLR6
	TM2D1
	TM2D2
	TM2D3
	TM4SF1
	TM4SF4
	TM4SF5
	TM6SF2
	TM7SF2
	TM7SF3
	TM9SF1
	TM9SF2
	TM9SF3
	TM9SF4
	TMBIM1
	TMBIM4
	TMBIM6
	TMC4
	TMCC1

	TMCO1
	TMCO3
	TMCO6
	TMCO7
	TMED1
	TMED10
	TMED10P
	TMED2
	TMED3
	TMED4
	TMED5
	TMED7
	TMED9
	TMEM1
	TMEM101
	TMEM105
	TMEM106A
	TMEM106B
	TMEM106C
	TMEM109
	TMEM11
	TMEM111
	TMEM115
	TMEM116
	TMEM120A
	TMEM123
	TMEM126A
	TMEM126B
	TMEM127
	TMEM129
	TMEM131
	TMEM133
	TMEM134
	TMEM135
	TMEM136
	TMEM137
	TMEM138
	TMEM140
	TMEM141
	TMEM143
	TMEM144
	TMEM147
	TMEM149
	TMEM14A
	TMEM14B
	TMEM14C
	TMEM14D
	TMEM150A
	TMEM156
	TMEM158
	TMEM159
	TMEM160
	TMEM161A
	TMEM165
	TMEM166
	TMEM167A

	TMEM167B
	TMEM16A
	TMEM17
	TMEM170A
	TMEM170B
	TMEM175
	TMEM176A
	TMEM177
	TMEM178
	TMEM179B
	TMEM18
	TMEM180
	TMEM181
	TMEM183A
	TMEM183B
	TMEM184B
	TMEM184C
	TMEM185A
	TMEM185B
	TMEM186
	TMEM187
	TMEM188
	TMEM189
	TMEM189-UBE2V1
	TMEM19
	TMEM191A
	TMEM191B
	TMEM192
	TMEM194A
	TMEM198
	TMEM199
	TMEM2
	TMEM20
	TMEM203
	TMEM205
	TMEM206
	TMEM207
	TMEM208
	TMEM209
	TMEM214
	TMEM216
	TMEM217
	TMEM218
	TMEM219
	TMEM222
	TMEM25
	TMEM27
	TMEM30A
	TMEM30B
	TMEM33
	TMEM37
	TMEM38B
	TMEM39A
	TMEM39B
	TMEM4
	TMEM41A

	TMEM41B
	TMEM42
	TMEM43
	TMEM44
	TMEM45A
	TMEM47
	TMEM48
	TMEM49
	TMEM5
	TMEM50A
	TMEM50B
	TMEM51
	TMEM53
	TMEM54
	TMEM55B
	TMEM56
	TMEM57
	TMEM59
	TMEM60
	TMEM62
	TMEM63A
	TMEM63B
	TMEM64
	TMEM66
	TMEM69
	TMEM70
	TMEM77
	TMEM79
	TMEM8
	TMEM80
	TMEM85
	TMEM86B
	TMEM87A
	TMEM88
	TMEM9
	TMEM91
	TMEM93
	TMEM97
	TMEM98
	TMEM99
	TMEM9B
	TMF1
	TMLHE
	TMOD1
	TMOD3
	TMPO
	TMPRSS12
	TMPRSS6
	TMSB10
	TMSB4X
	TMSL3
	TMTC3
	TMTC4
	TMUB1
	TMUB2
	TMX1

	TMX3
	TMX4
	TNC
	TNFAIP1
	TNFAIP3
	TNFAIP8
	TNFAIP8L1
	TNFRSF10B
	TNFRSF10D
	TNFRSF12A
	TNFRSF19
	TNFRSF1A
	TNFRSF1B
	TNFRSF21
	TNFSF10
	TNFSF12
	TNFSF13B
	TNFSF14
	TNFSF15
	TNIP1
	TNIP2
	TNK2
	TNKS1BP1
	TNPO1
	TNPO2
	TNPO3
	TNRC15
	TNRC6A
	TNRC6B
	TNS1
	TNS3
	TOB1
	TOB2
	TOE1
	TOLLIP
	TOM1
	TOM1L1
	TOM1L2
	TOMM20
	TOMM22
	TOMM34
	TOMM40
	TOMM40L
	TOMM5
	TOMM6
	TOMM7
	TOMM70A
	TOP1
	TOP1MT
	TOP1P2
	TOP2B
	TOP3A
	TOP3B
	TOPBP1
	TOPORS
	TOR1A

	TOR1AIP1
	TOR1AIP2
	TOR1B
	TOR3A
	TOX4
	TP53
	TP53AP1
	TP53BP1
	TP53BP2
	TP53I13
	TP53I3
	TP53INP1
	TP53INP2
	TP53RK
	TP53TG1
	TPCN2
	TPD52
	TPD52L1
	TPD52L2
	TPI1
	TPK1
	TPM1
	TPM2
	TPM3
	TPM4
	TPMT
	TPP1
	TPP2
	TPR
	TPRG1L
	TPRKB
	TPST1
	TPST2
	TPT1
	TRA1P2
	TRA2A
	TRABD
	TRADD
	TRAF3IP2
	TRAF4
	TRAF6
	TRAF7
	TRAFD1
	TRAK1
	TRAK2
	TRAM1
	TRAM2
	TRAP1
	TRAPPC1
	TRAPPC2
	TRAPPC2L
	TRAPPC2P1
	TRAPPC3
	TRAPPC4
	TRAPPC5
	TRAPPC6A

	TRAPPC6B
	TRAPPC9
	TRIAP1
	TRIB1
	TRIB3
	TRIM11
	TRIM13
	TRIM2
	TRIM21
	TRIM23
	TRIM24
	TRIM25
	TRIM26
	TRIM27
	TRIM28
	TRIM3
	TRIM32
	TRIM33
	TRIM35
	TRIM37
	TRIM38
	TRIM39
	TRIM4
	TRIM41
	TRIM44
	TRIM45
	TRIM5
	TRIM52
	TRIM55
	TRIM56
	TRIM66
	TRIM68
	TRIM78P
	TRIM8
	TRIM9
	TRIML1
	TRIO
	TRIOBP
	TRIP10
	TRIP11
	TRIP12
	TRIP4
	TRIP6
	TRIT1
	TRMT1
	TRMT11
	TRMT112
	TRMT12
	TRMT2A
	TRMT2B
	TRMT5
	TRMT6
	TRMT61A
	TRMU
	TRNAU1AP
	TRNP1

	TRNT1
	TROVE2
	TRPC4AP
	TRPM4
	TRPT1
	TRRAP
	TRUB2
	TSC1
	TSC2
	TSC22D1
	TSC22D2
	TSC22D3
	TSC22D4
	TSEN15
	TSEN2
	TSEN34
	TSEN54
	TSFM
	TSG101
	TSGA14
	TSHZ1
	TSHZ2
	TSHZ3
	TSKU
	TSNAX
	TSPAN1
	TSPAN10
	TSPAN12
	TSPAN13
	TSPAN14
	TSPAN15
	TSPAN17
	TSPAN3
	TSPAN31
	TSPAN33
	TSPAN4
	TSPAN6
	TSPAN7
	TSPAN9
	TSPO
	TSPYL1
	TSPYL2
	TSPYL4
	TSPYL6
	TSR1
	TSR2
	TSSC1
	TSSC4
	TST
	TSTA3
	TSTD1
	TSTD2
	TTC1
	TTC12
	TTC13
	TTC14

	TTC15
	TTC17
	TTC19
	TTC23
	TTC27
	TTC3
	TTC30A
	TTC31
	TTC32
	TTC33
	TTC35
	TTC37
	TTC38
	TTC39C
	TTC4
	TTC5
	TTC7B
	TTC8
	TTC9C
	TTF1
	TTF2
	TTL
	TTLL1
	TTLL12
	TTLL3
	TTLL4
	TTLL5
	TTPAL
	TTR
	TTRAP
	TTYH3
	TUBA1A
	TUBA1B
	TUBA1C
	TUBA3D
	TUBB
	TUBB2A
	TUBB2B
	TUBB2C
	TUBB3
	TUBB4Q
	TUBB6
	TUBE1
	TUBG1
	TUBG2
	TUBGCP2
	TUBGCP4
	TUBGCP5
	TUBGCP6
	TUFM
	TUFT1
	TUG1
	TULP3
	TULP4
	TUSC1
	TUSC2

	TUSC4
	TUT1
	TWF1
	TWF2
	TWISTNB
	TWSG1
	TXLNA
	TXN
	TXN2
	TXNDC11
	TXNDC12
	TXNDC14
	TXNDC15
	TXNDC17
	TXNDC5
	TXNDC9
	TXNIP
	TXNL1
	TXNL2
	TXNRD1
	TXNRD2
	TYK2
	TYMS
	TYRO3
	TYSND1
	TYW1
	TYW1B
	TYW3
	U1SNRNPBP
	U2AF1
	U2AF1L2
	U2AF1L4
	U2AF2
	UAP1
	UBA1
	UBA2
	UBA3
	UBA5
	UBA52
	UBA6
	UBA7
	UBAC1
	UBAC2
	UBAP1
	UBAP2
	UBAP2L
	UBB
	UBC
	UBD
	UBE1
	UBE1C
	UBE1DC1
	UBE2A
	UBE2D2
	UBE2D3
	UBE2D4

	UBE2E1
	UBE2E2
	UBE2E3
	UBE2F
	UBE2G1
	UBE2G2
	UBE2H
	UBE2I
	UBE2J1
	UBE2J2
	UBE2K
	UBE2L3
	UBE2L6
	UBE2M
	UBE2N
	UBE2O
	UBE2Q1
	UBE2Q2
	UBE2R2
	UBE2V1
	UBE2V2
	UBE2W
	UBE2Z
	UBE3A
	UBE3B
	UBE3C
	UBE4A
	UBE4B
	UBFD1
	UBIAD1
	UBL3
	UBL4A
	UBL5
	UBL7
	UBLCP1
	UBN1
	UBOX5
	UBP1
	UBQLN1
	UBQLN2
	UBQLN4
	UBR2
	UBR3
	UBR4
	UBR5
	UBR7
	UBTD1
	UBTD2
	UBTF
	UBXN1
	UBXN11
	UBXN2A
	UBXN2B
	UBXN4
	UBXN6
	UBXN8

	UCHL1
	UCHL3
	UCHL5
	UCHL5IP
	UCK1
	UCK2
	UCKL1
	UCN
	UCRC
	UFC1
	UFM1
	UFSP2
	UGCG
	UGCGL1
	UGCGL2
	UGDH
	UGP2
	UGT1A3
	UGT2B11
	UGT2B28
	UGT2B7
	UHRF1BP1
	UHRF1BP1L
	UHRF2
	UIMC1
	ULK1
	ULK2
	ULK3
	UMPS
	UNC119
	UNC119B
	UNC13B
	UNC45A
	UNC50
	UNC5CL
	UNC84A
	UNC84B
	UNC93A
	UNC93B1
	UNG
	UNKL
	UPF1
	UPF2
	UPF3A
	UPF3B
	UPLP
	UPP1
	UPRT
	UQCC
	UQCR
	UQCRB
	UQCRC1
	UQCRC2
	UQCRFS1
	UQCRH
	UQCRHL

	UQCRQ
	URB1
	URB2
	URG4
	URM1
	UROD
	UROS
	USE1
	USF1
	USF2
	USH1G
	USO1
	USP1
	USP10
	USP11
	USP12
	USP13
	USP14
	USP15
	USP16
	USP18
	USP21
	USP22
	USP24
	USP25
	USP3
	USP30
	USP33
	USP34
	USP36
	USP37
	USP38
	USP39
	USP4
	USP41
	USP42
	USP46
	USP47
	USP48
	USP49
	USP5
	USP6NL
	USP7
	USP8
	USP9X
	USPL1
	UTP11L
	UTP14A
	UTP14C
	UTP15
	UTP18
	UTP23
	UTP3
	UTP6
	UTRN
	UTS2

	UTX
	UTY
	UVRAG
	UXS1
	UXT
	VAC14
	VAMP1
	VAMP2
	VAMP3
	VAMP4
	VAMP5
	VAMP7
	VAMP8
	VAPA
	VARS
	VARS2
	VASN
	VASP
	VAT1
	VAV2
	VBP1
	VCL
	VCP
	VCPIP1
	VDAC1
	VDAC3
	VEGFA
	VEGFB
	VENTXP1
	VEZF1
	VEZT
	VGLL4
	VHL
	VIL1
	VIL2
	VIM
	VIPR1
	VISA
	VKORC1
	VKORC1L1
	VMO1
	VN1R2
	VPS11
	VPS13A
	VPS13D
	VPS16
	VPS18
	VPS24
	VPS25
	VPS26
	VPS26A
	VPS26B
	VPS28
	VPS29
	VPS33A
	VPS33B

	VPS35
	VPS36
	VPS37A
	VPS37B
	VPS37C
	VPS37D
	VPS39
	VPS41
	VPS45
	VPS4A
	VPS4B
	VPS52
	VPS54
	VPS72
	VPS8
	VRK1
	VRK2
	VRK3
	VTA1
	VTI1B
	VTN
	VWA1
	VWA3A
	VWA5A
	VWCE
	VWF
	WAC
	WARS
	WAS
	WASF2
	WASF3
	WASH1
	WASH2P
	WASH5P
	WASL
	WBP1
	WBP11
	WBP2
	WBP4
	WBP5
	WBSCR16
	WBSCR22
	WBSCR27
	WDFY1
	WDFY2
	WDFY3
	WDR1
	WDR12
	WDR13
	WDR18
	WDR19
	WDR20
	WDR21A
	WDR22
	WDR23
	WDR24

	WDR25
	WDR26
	WDR33
	WDR34
	WDR36
	WDR37
	WDR4
	WDR40A
	WDR41
	WDR42A
	WDR43
	WDR44
	WDR45
	WDR45L
	WDR46
	WDR48
	WDR5
	WDR51A
	WDR51B
	WDR53
	WDR54
	WDR55
	WDR57
	WDR59
	WDR6
	WDR60
	WDR61
	WDR68
	WDR7
	WDR70
	WDR72
	WDR73
	WDR74
	WDR75
	WDR77
	WDR79
	WDR8
	WDR81
	WDR82
	WDR85
	WDR89
	WDR90
	WDR91
	WDR92
	WDSOF1
	WDSUB1
	WDYHV1
	WEE1
	WFS1
	WHAMM
	WHSC2
	WIBG
	WIPI1
	WIPI2
	WNK1
	WNK3

	WNT5B
	WRB
	WRN
	WRNIP1
	WSB1
	WSB2
	WTAP
	WWC1
	WWC2
	WWC3
	WWOX
	WWP1
	WWP2
	XAB2
	XAF1
	XBP1
	XCR1
	XDH
	XIAP
	XIST
	XKR8
	XPA
	XPC
	XPNPEP1
	XPNPEP2
	XPNPEP3
	XPO1
	XPO4
	XPO5
	XPO6
	XPO7
	XPR1
	XRCC1
	XRCC2
	XRCC5
	XRCC6
	XRCC6BP1
	XRN1
	XRN2
	XYLB
	XYLT2
	YAF2
	YAP1
	YARS
	YARS2
	YBX1
	YDJC
	YEATS2
	YEATS4
	YES1
	YIF1A
	YIF1B
	YIPF1
	YIPF2
	YIPF3
	YIPF4

	YIPF5
	YIPF6
	YME1L1
	YOD1
	YPEL1
	YPEL2
	YPEL3
	YPEL5
	YRDC
	YTHDC1
	YTHDF1
	YTHDF2
	YTHDF3
	YWHAB
	YWHAE
	YWHAG
	YWHAH
	YWHAQ
	YWHAZ
	YY1
	YY1AP1
	ZADH2
	ZAK
	ZBED1
	ZBED3
	ZBED4
	ZBED5
	ZBTB11
	ZBTB16
	ZBTB17
	ZBTB2
	ZBTB20
	ZBTB22
	ZBTB24
	ZBTB25
	ZBTB3
	ZBTB32
	ZBTB33
	ZBTB34
	ZBTB39
	ZBTB4
	ZBTB40
	ZBTB42
	ZBTB43
	ZBTB44
	ZBTB45
	ZBTB47
	ZBTB48
	ZBTB5
	ZBTB6
	ZBTB7A
	ZBTB7B
	ZBTB8OS
	ZBTB9
	ZC3H10
	ZC3H11B

	ZC3H12A
	ZC3H12B
	ZC3H12C
	ZC3H14
	ZC3H15
	ZC3H18
	ZC3H3
	ZC3H4
	ZC3H5
	ZC3H7A
	ZC3H8
	ZC3HAV1
	ZC3HC1
	ZC4H2
	ZCCHC11
	ZCCHC14
	ZCCHC17
	ZCCHC24
	ZCCHC3
	ZCCHC6
	ZCCHC7
	ZCCHC8
	ZCCHC9
	ZCRB1
	ZCWPW1
	ZDHHC1
	ZDHHC11
	ZDHHC12
	ZDHHC13
	ZDHHC14
	ZDHHC16
	ZDHHC17
	ZDHHC18
	ZDHHC2
	ZDHHC23
	ZDHHC24
	ZDHHC3
	ZDHHC4
	ZDHHC5
	ZDHHC6
	ZDHHC7
	ZDHHC8
	ZDHHC9
	ZER1
	ZFAND1
	ZFAND2A
	ZFAND2B
	ZFAND3
	ZFAND5
	ZFAND6
	ZFC3H1
	ZFHX3
	ZFP1
	ZFP106
	ZFP161
	ZFP36

	ZFP36L1
	ZFP36L2
	ZFP90
	ZFP91
	ZFPL1
	ZFPM1
	ZFR
	ZFX
	ZFY
	ZFYVE1
	ZFYVE16
	ZFYVE19
	ZFYVE20
	ZFYVE21
	ZFYVE26
	ZFYVE27
	ZGPAT
	ZHX1
	ZHX2
	ZHX3
	ZIK1
	ZKSCAN1
	ZKSCAN2
	ZKSCAN3
	ZKSCAN5
	ZMAT2
	ZMAT3
	ZMAT5
	ZMIZ1
	ZMIZ2
	ZMPSTE24
	ZMYM1
	ZMYM2
	ZMYM3
	ZMYM4
	ZMYM5
	ZMYM6
	ZMYND11
	ZMYND19
	ZMYND8
	ZNF10
	ZNF114
	ZNF12
	ZNF131
	ZNF133
	ZNF134
	ZNF136
	ZNF14
	ZNF140
	ZNF142
	ZNF143
	ZNF146
	ZNF148
	ZNF155
	ZNF16
	ZNF160

	ZNF165
	ZNF17
	ZNF174
	ZNF177
	ZNF18
	ZNF181
	ZNF187
	ZNF189
	ZNF193
	ZNF195
	ZNF197
	ZNF20
	ZNF200
	ZNF205
	ZNF207
	ZNF212
	ZNF217
	ZNF219
	ZNF22
	ZNF223
	ZNF226
	ZNF227
	ZNF23
	ZNF232
	ZNF239
	ZNF25
	ZNF250
	ZNF252
	ZNF254
	ZNF256
	ZNF259
	ZNF26
	ZNF260
	ZNF263
	ZNF264
	ZNF266
	ZNF268
	ZNF271
	ZNF274
	ZNF275
	ZNF276
	ZNF277
	ZNF280C
	ZNF280D
	ZNF281
	ZNF282
	ZNF295
	ZNF296
	ZNF3
	ZNF30
	ZNF302
	ZNF304
	ZNF317
	ZNF318
	ZNF319
	ZNF32

	ZNF320
	ZNF322A
	ZNF322B
	ZNF323
	ZNF324
	ZNF324B
	ZNF326
	ZNF329
	ZNF330
	ZNF331
	ZNF337
	ZNF33A
	ZNF33B
	ZNF34
	ZNF341
	ZNF343
	ZNF35
	ZNF350
	ZNF358
	ZNF362
	ZNF364
	ZNF384
	ZNF385A
	ZNF394
	ZNF395
	ZNF398
	ZNF407
	ZNF408
	ZNF410
	ZNF416
	ZNF417
	ZNF419
	ZNF426
	ZNF428
	ZNF430
	ZNF432
	ZNF433
	ZNF434
	ZNF436
	ZNF438
	ZNF442
	ZNF443
	ZNF444
	ZNF446
	ZNF45
	ZNF451
	ZNF467
	ZNF468
	ZNF473
	ZNF480
	ZNF483
	ZNF486
	ZNF490
	ZNF498
	ZNF500
	ZNF503

	ZNF507
	ZNF509
	ZNF511
	ZNF512
	ZNF512B
	ZNF513
	ZNF517
	ZNF518A
	ZNF518B
	ZNF524
	ZNF526
	ZNF529
	ZNF544
	ZNF548
	ZNF549
	ZNF550
	ZNF557
	ZNF558
	ZNF559
	ZNF561
	ZNF562
	ZNF563
	ZNF564
	ZNF565
	ZNF574
	ZNF576
	ZNF577
	ZNF579
	ZNF580
	ZNF581
	ZNF583
	ZNF585A
	ZNF586
	ZNF589
	ZNF592
	ZNF593
	ZNF598
	ZNF600
	ZNF613
	ZNF614
	ZNF615
	ZNF618
	ZNF621
	ZNF622
	ZNF624
	ZNF626
	ZNF627
	ZNF629
	ZNF638
	ZNF644
	ZNF649
	ZNF650
	ZNF652
	ZNF653
	ZNF654
	ZNF655

	ZNF658B
	ZNF668
	ZNF669
	ZNF670
	ZNF671
	ZNF672
	ZNF673
	ZNF674
	ZNF680
	ZNF682
	ZNF684
	ZNF688
	ZNF689
	ZNF69
	ZNF692
	ZNF696
	ZNF697
	ZNF7
	ZNF700
	ZNF706
	ZNF707
	ZNF716
	ZNF717
	ZNF721
	ZNF738
	ZNF74
	ZNF746
	ZNF75A
	ZNF75D
	ZNF76
	ZNF763
	ZNF766
	ZNF767
	ZNF768
	ZNF770
	ZNF771
	ZNF773
	ZNF776
	ZNF777
	ZNF783
	ZNF784
	ZNF785
	ZNF786
	ZNF787
	ZNF789
	ZNF791
	ZNF800
	ZNF805
	ZNF815
	ZNF821
	ZNF823
	ZNF828
	ZNF830
	ZNF837
	ZNF839
	ZNF84

	ZNF841
	ZNF845
	ZNF860
	ZNF91
	ZNF93
	ZNFX1
	ZNHIT1
	ZNHIT2
	ZNHIT3
	ZNHIT6
	ZNRD1
	ZP3
	ZRANB1
	ZRANB2
	ZSCAN12L1
	ZSCAN16
	ZSCAN18
	ZSCAN2
	ZSCAN21
	ZSCAN5A
	ZSWIM1
	ZSWIM4
	ZSWIM6
	ZSWIM7
	ZUFSP
	ZW10
	ZWILCH
	ZXDC
	ZYG11B
	ZYX
	ZZEF1
	ZZZ3

Supplementary Table S3: Gene list of exclusively expressed genes in HLCs.

Term	%	PValue	Genes
GO:0005911~cell-cell junction	1,694	9,18E-05	CLDN18, OCLN, CLDN9, CLDN10, MIP, COL17A1, PVRL1, PPL, WNK4, TGM1, OBSL1, MLLT4, PARD6A, MICALL2, PARD6B, GJB7, MAGI1, LIN7C, MPP7, GJB6, ABCB4, LIN7A, IGSF5, ITGA6, PKP3, DSC1, ABCC2, PDZD3
GO:0043296~apical junction complex	1,028	5,81E-04	MICALL2, PARD6A, PARD6B, CLDN18, CLDN9, OCLN, MAGI1, LIN7C, MPP7, CLDN10, LIN7A, IGSF5, WNK4, PKP3, PPL, DSC1, PDZD3
GO:0030054~cell junction	3,267	5,88E-04	SEPT3, CLDN9, OCLN, GABRB1, FERMT1, SYT6, GABBR2, ZNRF2, WNK4, SV2A, CHRNA3, ANKS1B, MAGI1, MPP7, LIG4, PCLO, IGSF5, RND1, CHRM3, OPHN1, ERC2, IGSF9, CLDN18, CTNND2, CLDN10, MIP, PVRL4, SYNPR, COL17A1, PVRL1, PPL, SYN2, TGM1, OBSL1, MLLT4, GABRP, NPHP1, PARD6A, MICALL2, PARD6B, GJB7, NLGN1, LIN7C, GJB6, HOMER1, ABCB4, LIN7A, PPP1R9A, ITGA6, RAPSN, PKP3, DSC1, ABCC2, PDZD3
GO:0016327~apicolateral plasma membrane	1,028	8,13E-04	MICALL2, PARD6A, PARD6B, CLDN18, CLDN9, OCLN, MAGI1, LIN7C, MPP7, CLDN10, LIN7A, IGSF5, WNK4, PKP3, PPL, DSC1, PDZD3
GO:0005624~membrane fraction	4,537	0,00141	SEPT3, SLC15A1, VAPB, SLC15A2, HIP1R, CYP2S1, PDLIM5, DPP10, HPS1, CNGB1, SLC16A1, SLC23A1, ART5, KCNK9, SLC24A2, SLC1A6, ATP8B1, LMOD1, JPH1, PCDHGA12, STS, CYP1A1, SLC22A7, NR0B1, FOLH1, DGAT2, CNTN1, RYR2, ABAT, ERC2, LOC388514, STEAP2, TM4SF4, GCNT3, HSD3B1, OAS3, ARF6, OAS2, ABCA3, MIP, FMO4, IGF1R, SYNPR, PVRL1, FMO1, RASGRP1, BCL2, CYP26B1, TNKS, FUT1, A4GNT, MTMR7, UGT2B28, ACSL5, KLK6, CYP46A1, CYP2C8, LIN7C, MAL, HOMER1, LIN7A, SLC10A1, ABCB4, PPP1R9A, KCNJ8, LASS1, MAP2, ADRA1B, MEP1A, DSC1, DIO1, ABCC2, RDH16, PDZD3, RSC1A1
GO:0070160~occluding junction	0,786	0,00236	MICALL2, PARD6A, PARD6B, CLDN18, CLDN9, OCLN, MAGI1, LIN7C, CLDN10, MPP7, LIN7A, IGSF5, WNK4

GO:0005923~tight junction	0,786	0,00236	MICALL2, PARD6A, PARD6B, CLDN18, CLDN9, OCLN, MAGI1, LIN7C, CLDN10, MPP7, LIN7A, IGSF5, WNK4
GO:0005626~insoluble fraction	4,598	0,00244	SEPT3, SLC15A1, VAPB, SLC15A2, HIP1R, CYP2S1, PDLIM5, DPP10, HPS1, CNGB1, SLC16A1, SLC23A1, ART5, KCNK9, SLC24A2, SLC1A6, ATP8B1, SYNJ2, LMOD1, JPH1, PCDHGA12, STS, CYP1A1, SLC22A7, NR0B1, FOLH1, DGAT2, CNTN1, RYR2, ABAT, ERC2, LOC388514, STEAP2, TM4SF4, GCNT3, HSD3B1, OAS3, ARF6, OAS2, ABCA3, MIP, FMO4, IGF1R, SYNPR, PVRL1, FMO1, RASGRP1, BCL2, CYP26B1, TNKS, FUT1, A4GNT, MTMR7, UGT2B28, ACSL5, KLK6, CYP46A1, CYP2C8, LIN7C, MAL, HOMER1, LIN7A, SLC10A1, ABCB4, PPP1R9A, KCNJ8, LASS1, MAP2, ADRA1B, MEP1A, DSC1, DIO1, ABCC2, RDH16, PDZD3, RSC1A1
GO:0000267~cell fraction	5,687	0,00252	CGA, SEPT3, CGB, INSL4, SLC15A1, VAPB, SLC15A2, HIP1R, CYP2S1, PDLIM5, DPP10, HPS1, CGB7, CNGB1, SLC16A1, SLC23A1, ART5, KCNK9, STAC, IAPP, SLC24A2, SLC1A6, ATP8B1, SYNJ2, LMOD1, JPH1, PCDHGA12, STS, CYP1A1, SLC22A7, FBP1, NR0B1, FOLH1, DGAT2, CNTN1, RYR2, ABAT, ERC2, LOC388514, STEAP2, CRYBA1, TM4SF4, GCNT3, HSD3B1, OAS3, ARF6, OAS2, TRH, ABCA3, TAC3, LOC653269, IL17C, MIP, FMO4, CALCB, IGF1R, SYNPR, PVRL1, FMO1, RASGRP1, BCL2, CYP26B1, HAAO, SRR, TNKS, FUT1, A4GNT, MTMR7, UGT2B28, ACSL5, KLK6, CYP46A1, CYP2C8, LIN7C, MTL5, MAL, IDO1, HOMER1, LIN7A, ABCB4, SLC10A1, PPP1R9A, KCNJ8, LASS1, MAP2, MEP1A, ADRA1B, DSC1, DIO1, ABCC2, RDH16, PDZD3, LHB, RSC1A1
GO:0034702~ion channel complex	1,512	0,00345	FXYD1, KCNE1L, CLCN2, GABRB1, CACNB2, KCNA5, CNGB1, KCNJ14, KCNJ13, KCNS3, KCNQ4, KCNS1, TTYH2, KCNQ2, CHRNA3, ANO9, GABRP, SCN2B, KCTD1, CACNG1, CLIC3, KCNJ8, CLIC6, RYR2, KCTD14
GO:0042598~vesicular fraction	1,694	0,00441	HSD3B1, CYP2S1, OAS3, OAS2, FMO4, MIP, IGF1R, PVRL1, FMO1, BCL2, CYP26B1, TNKS, JPH1, UGT2B28, ACSL5, KLK6, STS, CYP1A1, CYP46A1, CYP2C8, DGAT2, KCNJ8, LASS1, MAP2, LOC388514, DIO1, STEAP2, RDH16

GO:0005792~microsome	1,633	0,00575	HSD3B1, CYP2S1, OAS3, OAS2, FMO4, MIP, IGF1R, PVRL1, FMO1, BCL2, CYP26B1,<br>TNKS, JPH1, UGT2B28, ACSL5, KLK6, STS, CYP1A1, CYP46A1, CYP2C8,<br>DGAT2, KCNJ8, LASS1, MAP2, LOC388514, DIO1, RDH16

Supplementary Table S4: Gene lists of the venn diagram Figure 5C.					
fetal_vs_ phh_anova	hlc_vs_ fetal_anova	fetal_vs_ phh_anova AND hlc_vs_fetal_ anova	hlc_vs_ phh_anova	fetal_vs_phh_ anova AND hlc_vs_phh_ anova	hlc_vs_fetal_ anova AND hlc_vs_phh anova
ADIPOR1	A2M	ABCB10	AASDH	ABAT	AADAC
AEN	AARS	ABHD11	ABCB11	ABCA2	AAK1
ANXA11	ABCB6	ACAP1	ABCC10	ABCB1	ABCA8
ANXA8L2	ABHD8	ACSL4	ABCG8	ABCC11	ABCC6P2
ASAP1IT1	ABI3	ACSS1	ABT1	ABCC2	ABHD12
ATF2	ACAD8	ACTN4	ACAD9	ABCC3	ABHD14A
ATP7A	ACBD3	ADAP2	ACLY	ABHD1	ABHD14B
BCL7C	ADAMTSL4	ADORA1	ACO1	ABLIM1	ACAA1
C16orf59	AEBP2	AFF3	ACSS2	ABLIM3	ACADM
C16orf87	AFF4	AHSP	ACTB	ABTB2	ACAT2
C17orf49	AMDHD1	AKTIP	ACYP1	ACAA2	ACBD7
C1orf144	APOBEC3F	ALAS2	ACYP2	ACACA	ACER3
C1orf25	ARFGAP2	ANK1	ADAMTSL2	ACACB	ACP1
CAND2	ARID5B	ANKRD41	ADARB1	ACAD11	ACPL2
CBR4	ARIH1	ANXA2P3	ADH5	ACADVL	ACPP
CCNF	ARS2	AP2S1	ADHFE1	ACOT4	ACSM3
CKS2	ASCC1	APOA2	ADRA2A	ACOT9	ACTC1
CLDN3	ATE1	APOBEC2	AEBP1	ACSL5	ACTN1
CNO	ATP5H	APOM	AES	ACSM5	ACVR1
COQ6	ATP5J2	APP	AGMAT	ACTR5	ADAM19
COTL1	ATP6V1E1	ARHGAP15	AGPAT6	ACY3	ADAM9
CREBL2	AVP	ARHGAP22	AIDA	ADAM15	ADAMTS1
CSRP2BP	AZIN1	ARHGAP30	AKAP1	ADCY3	ADAMTS19
DC36	B9D1	ARHGAP9	AKAP10	ADCYAP1R1	ADAR
DENND2C	BCOR	ARIH2	AKR1D1	ADH1B	ADH1A
DGKQ	C12orf10	ARL4A	ALOXE3	ADH1C	ADH4
DLEU1	C12orf49	ARL9	ALS2CR4	ADI1	ADIPOR2
DSTYK	C14orf166	ARMCX3	AMD1	ADK	ADORA2B
DYNLT1	C17orf76	ASCL2	ANAPC1	ADPRHL2	AFAP1
E2F3	C17orf96	ASTE1	ANGEL2	ADRA1A	AFAP1L2
E2F8	C5orf15	ATAD1	ANGPTL2	ADRM1	AGBL5
EFCAB4A	C7orf42	ATL3	ANKFY1	ADSL	AGK
ENO1	CCDC120	ATP1B1	ANKRD13A	ADSSL1	AGTPBP1
FAM104A	CD38	ATP1B2	ANKRD16	AFAR3	AHCYL1
FAM164C	CD74	ATP5G1	ANKRD17	AFF1	AHCYL2
FANCI	CEBPD	ATP6V0A1	ANKRD33	AFMID	AHSG
GATA5	CIRH1A	AYP1p1	ANKRD39	AFP	AIF1L
GMPS	CLDN15	BACE2	ANKS4B	AFTPH	AIFM1
HERC1	CNGA1	BANP	AOF2	AGPAT4	AIG1
HLA-G	CNN3	BCAR1	AP1G2	AGPAT9	AIM1L
HMGB1L1	CTDSP1	BEND7	AP3M1	AGXT2L1	AIP
HPS4	CXADR	BEX1	APOE	AGXT2L2	AK2P2
IL18BP	CYGB	BGN	APTX	AHR	AKR1B1
INPP5D	DAZAP1	BIK	ARHGAP1	AKIRIN2	AKT1
IRS1	DENND5A	BLVRA	ARID2	AKR1A1	ALB
JOSD2	DGCR2	BLVRB	ARL16	AKR1B10	ALDH1A3
KCNT2	DLGAP4	BMP2	ARL6IP4	AKR1C2	ALDH3B2

KIAA0831	DRD4	BMP2K	ARMC1	AKR1C3	ALG10
KIF21A	EIF2B2	BNIP3L	ARMC10	AKR1C4	ALG10B
LDOC1L	ELMOD2	BOLA3	ARPC1B	AKR7A3	ALKBH3
LOC100129685	F13B	BPGM	ARPC3	ALAS1	ALX1
LOC100130367	F2RL1	BSN	ARPC5L	ALDH1B1	AMBP
LOC100131261	FAM119B	BTG2	ARSA	ALG14	AMFR
LOC100131381	FAM171A1	BTK	ARSK	ALG1L	AMOT
LOC100132863	FAM50A	BZRPL1	ART5	ALG2	AMY1B
LOC100134291	FIS1	C10orf33	ASCC3	ALG3	AMZ2
LOC100190986	FLJ22795	C10orf35	ATG16L1	ALPL	ANAPC11
LOC143666	FLJ40113	C12orf27	ATG2A	ALS2CL	ANGPTL3
LOC282997	FLJ40504	C12orf45	ATG4B	AMACR	ANK3
LOC387867	FOLR3	C14orf173	ATN1	AMHR2	ANKIB1
LOC642361	FUK	C15orf57	ATP12A	AMY2A	ANKLE2
LOC644879	GADD45GIP1	C19orf22	ATP5C1	AMY2B	ANKRD10
LOC644931	GMFB	C19orf42	ATP5G3	ANAPC5	ANKRD11
LOC650546	GOLGA5	C19orf48	ATP5S	ANKRD35	ANKRD38
LOC730740	GPC3	C19orf62	ATP5SL	ANLN	ANKRD50
LPAR5	GSTA2	C1orf162	ATP6V1B2	ANO1	ANKS1A
LRRC32	HADHB	C1orf59	ATP8B2	ANXA10	ANO10
MAD2L1	HEMGN	C1QC	ATRIP	ANXA2P2	ANO6
MARK3	HK3	C20orf108	AVEN	ANXA4	ANTXR1
MBLAC2	JUNB	C20orf175	B9D2	ANXA6	ANXA1
METAP2	KIAA1545	C20orf27	BAG5	AOX1	ANXA3
MPHOSPH9	KLF2	C22orf25	BANF1	AP2B1	AP1M2
MXD3	LIPA	C2orf32	BAT2L	AP2M1	AP3B2
NAP1L4	LOC100129122	C3orf10	BBS2	AP4E1	APBB1IP
NEFH	LOC100133477	C3orf54	BCL2L12	APCS	APH1A
NOSTRIN	LOC145853	C5orf23	BDNF	APOC4	APOA1
ODC1	LOC283767	C5orf4	BEND6	APOF	APOC1
OIP5	LOC284821	C7orf27	BLZF1	APOL2	APRT
PA2G4	LOC285733	C9orf123	BMPR2	APOL3	AQP12A
PASK	LOC399748	C9orf30	BMX	AQP7P1	AQR
PCBD2	LOC440313	C9orf40	BPHL	AQP7P2	ARCN1
PDXK	LOC644860	CALD1	BRD3	AQP9	ARFGEF1
PGAM5	LOC644934	CBX3	BRD7P2	AR	ARG2
PGBD3	LOC645236	CCDC102A	BRE	ARHGAP12	ARHGAP10
PHLDA2	LOC645979	CCDC26	BSPRY	ARHGEF3	ARHGAP18
PLAC9	LOC648294	CCDC52	BTBD10	ARID1A	ARHGAP21
PLTP	LOC650298	CCL15	BTG1	ARL14	ARHGAP28
PLXNB2	LOC651697	CCL23	BTN3A3	ARL2	ARHGAP29
PNO1	LOC728290	CCNE2	C10orf11	ARL5B	ARHGAP8
PRMT6	LOC728823	CD34	C10orf32	ARL8B	ARHGEF10
ProSAPiP1	LOC729776	CD5L	C10orf88	ARMC5	ARHGEF17
PRTFDC1	LOC730316	CD97	C11orf54	ARMC6	ARHGEF7
RAD21	LOC732425	CDC14B	C12orf30	ARMCX6	ARID3A
RASSF7	LRRN3	CDH5	C12orf62	ARNTL	ARID3B
RBBP4	LRSAM1	CDKN2D	C13orf34	ARP11	ARL5A
RBPJ	MAPKAPK5	CENPM	C14orf105	ARRDC4	ARMC7
RCE1	MARCH2	CENTG2	C14orf133	ARSD	ARPC1A
RFNG	MARCH8	CFD	C14orf143	ASB13	ARPC2
RGL1	MAT2B	CKAP2	C14orf2	ASB7	ARPC5
RHBDF2	MCOLN1	CLEC14A	C15orf63	ASIP	ARPP19
RHOQ	MED8	CLEC1B	C16orf68	ATF3	ARRDC2
RPA3	MFSD1	CLEC4G	C16orf7	ATF4	ASAM
RRP1	MKRN2	CLIC2	C17orf101	ATF6	ASAP2

RUSC2	MRPL1	CNDP2	C17orf53	ATOH8	ASB3
S100A16	MRPL24	CNPY2	C17orf62	ATP1B3	ASNS
SASS6	MRPL37	CNPY4	C17orf79	ATP5A1	ATF7IP2
SLBP	MRPL55	CNRIP1	C17orf80	AURKA	ATIC
SLC1A1	MYO18A	COL9A1	C17orf85	AZGP1	ATP1A1
SLC39A11	NAT12	COLEC11	C17orf89	BAAT	ATP2B1
SLMO1	NBN	COMMD4	C19orf10	BAD	ATP2B4
SNHG5	NME3	COPS3	C19orf2	BAG3	ATP2C1
SP4	NR1H2	CPOX	C19orf50	BAIAP2	ATP5E
SPINK1	NUP88	CRB1	C19orf70	BAIAP2L2	ATP5EP2
SPTY2D1	OSGEP	CRHBP	C1orf106	BBOX1	ATP6AP1
TACC3	PARP4	CSNK1D	C1orf124	BCKDHB	ATP6V0A4
TGFB1I1	PDE8B	CTGLF1	C1orf165	BCL10	ATP6V0B
TMEM127	PDHB	CTNS	C1orf24	BCL11A	ATP6V1B1
TMEM171	PDS5A	CUL4A	C1orf43	BCL3	ATP6V1H
TNFSF4	PLEKHB2	CX3CR1	C1orf55	BCL7B	ATP9A
TOR1AIP2	PMS2L4	CXCL12	C1orf85	BDH2	ATXN3
TP53I3	POLB	CYBB	C20orf117	BECN1	AXIN2
TPI1	POLR1E	CYBRD1	C20orf177	BEXL1	AZI1
TRIM39	POLR3F	CYP1A1	C20orf29	BHMT	B3GALT4
TRMT5	PPM1B	CYP26A1	C20orf45	BIRC3	B4GALT1
TSPAN18	PPP2CA	CYP3A7	C20orf72	BLOC1S2	B4GALT4
TSPAN5	PRKCDBP	CYTL1	C21orf119	BNIP2	B4GALT6
TTC13	PRR13	DCDC2	C21orf55	BOAT	BAI2
TUBA1A	PRRG1	DCK	C21orf70	BOK	BAIAP2L1
USP14	PSG3	DCN	C22orf27	BST2	BAMBI
VASH1	PTPN1	DKC1	C2orf44	BTBD12	BAPX1
WRNIP1	RBPMS2	DLK1	C2orf69	BTF3	BARD1
ZHX2	RHBDF1	DNAJA3	C2orf82	BTRC	BASP1
ZNF121	RHOG	DNAJA4	C3orf25	BUB1	BAT2
ZNF407	RPS26	DNAJC3	C3orf50	BUB1B	BAT2D1
ZNF451	RPS9	DNASE1L3	C4A	BYSL	BAZ2B
ZNF512	SAC3D1	DOCK10	C4BPB	C10orf116	BBS1
ZSWIM5	SCRIB	DOCK8	C4orf18	C10orf125	BBS4
	SEC14L4	DOLPP1	C5orf21	C10orf140	BBS9
	SERPINA5	DPYSL5	C6orf108	C10orf4	BCAP29
	SERPINF2	DST	C6orf153	C10orf59	BCAR3
	SF4	DUSP9	C6orf192	C10orf68	BCAR4
	SH3RF1	DUT	C6orf221	C11orf17	BCAT1
	SHE	E2F2	C6orf62	C11orf48	BCL9
	SLC25A29	E2F4	C7orf40	C12orf31	BCL9L
	SLC25A42	ECM1	C7orf70	C12orf34	BDH1
	SLC2A9	EFNA1	C8orf38	C12orf44	BFAR
	SLC39A3	EHBP1L1	C8orf40	C12orf56	BICD1
	SLC45A3	EIF1AY	C8orf47	C13orf3	BIN1
	SLC48A1	EIF4E3	C9orf119	C14orf106	BLOC1S1
	SLCO1B3	ELTD1	C9orf135	C14orf147	BMF
	SNX22	EMILIN1	C9orf58	C14orf149	BMP6
	SP2	ENPP2	C9orf93	C14orf167	BMP7
	ST3GAL3	EPB41	CA12	C14orf169	BMPR1A
	STK11	EPB42	CA2	C15orf52	BNC1
	SYS1	EPOR	CABC1	C16orf48	BNC2
	THAP11	ERMAP	CACNG4	C16orf91	BNIPL
	TIAF1	ETV4	CAD	C17orf68	BOC
	TJP3	ETV5	CALCOCO2	C17orf90	BRD7
	TNS1	EZH2	CAND1	C17orf91	BRD8

	TOMM7	FAM117A	CAPN6	C18orf10	BRP44
	TOR2A	FAM124B	CAPRIN2	C18orf18	BRP44L
	TPD52L2	FAM188A	CARS2	C18orf8	BRSK1
	TRAM1	FAM46C	CASP6	C19orf12	BRWD1
	TSPAN12	FAM57A	CATSPER2	C19orf24	BTAF1
	TTTY14	FAM65C	CCDC107	C19orf36	BTBD2
	UBE2E2	FAM73B	CCDC13	C19orf63	BTBD3
	UCP2	FCER1G	CCDC132	C1GALT1C1	BTG3
	VEZT	FCGBP	CCDC14	C1orf107	BZW2
	WASL	FCRLB	CCDC24	C1orf122	C10orf118
	YIPF1	FECH	CCDC47	C1orf149	C10orf65
	YTHDF1	FERMT3	CCDC49	C1orf168	C11orf51
	ZBED4	FGL2	CCDC8	C1orf212	C11orf59
	ZDHHC6	FGR	CCDC97	C1orf51	C11orf70
	ZNF277	FLII	CCL2	C1orf63	C11orf80
	ZNF524	FLJ20273	CCL3L1	C1R	C12orf11
	ZNRD1	FLJ20489	CCNA1	C1RL	C12orf35
	ZSCAN16	FMO1	CCNG2	C1S	C12orf47
		FOXD4L1	CCNI	C20orf111	C12orf52
		FOXO1	CCPG1	C20orf24	C13orf23
		FOXQ1	CCR7	C20orf46	C14orf102
		FPR3	CCRK	C20orf55	C14orf135
		FYB	CD58	C20orf7	C14orf145
		GAB3	CD83	C21orf91	C14orf4
		GAPVD1	CD99L2	C22orf29	C14orf73
		GART	CDAN1	C2orf18	C14orf78
		GATA1	CDC25C	C2orf47	C15orf41
		GFPT1	CDC40	C4BPA	C17orf58
		GFRA2	CDC42	C4orf32	C17orf63
		GIMAP4	CDC42EP5	C4orf34	C17orf69
		GIMAP6	CDCA2	C5orf13	C17orf71
		GIMAP7	CDH23	C5orf35	C18orf26
		GJA4	CDH6	C5orf46	C18orf54
		GLIPR2	CDX2	C5orf53	C19orf43
		GLRX5	CEACAM1	C6	C1orf105
		GMFG	CENPQ	C6orf141	C1orf152
		GMNN	CEP110	C6orf142	C1orf198
		GMPR	CEP68	C6orf145	C1orf53
		GNG11	CHD8	C6orf160	C1orf57
		GPBAR1	CHN1	C7orf20	C1orf74
		GPC1	CHST14	C7orf44	C1orf88
		GPC5	CLCN3	C7orf50	C1orf9
		GPR116	CLEC16A	C8G	C20orf20
		GPR126	CLIP4	C9orf150	C20orf43
		GPR137B	CLK2	C9orf3	C20orf75
		GPR34	CLUAP1	C9orf82	C21orf2
		GPR44	CLYBL	C9orf89	C21orf66
		GPT2	CMC1	CABLES1	C2orf15
		GRAP	CMTM6	CACNA1H	C2orf28
		GSN	CNKSR3	CALCA	C3orf19
		GSTA4	CNOT1	CAMK2B	C3orf59
		GSTM2	CNTN4	CAPN5	C4orf14
		GYPA	CNTNAP1	CASC4	C4orf31
		GYPB	COL22A1	CASP7	C4orf41
		GYPC	COL6A2	CASZ1	C5
		GYPE	COMMD5	CAV1	C5orf33

		HAL	COPB1	CAV2	C5orf42
		HBA1	COX15	CBLC	C5orf51
		HBA2	COX7A2	CBLL1	C5orf54
		HBB	CRABP1	CBR3	C6orf117
		HBBP1	CRADD	CBS	C6orf204
		HBG1	CRCP	CBX4	C6orf225
		HBG2	CREB1	CBX5	C7orf23
		HBM	CREBBP	CCDC106	C7orf68
		HBQ1	CRKL	CCDC21	C8A
		HBZ	CROP	CCDC3	C8orf4
		HCLS1	CRTAP	CCDC50	C8orf42
		HDDC3	CRY2	CCDC88B	C8orf55
		HDHD2	CRYZ	CCDC94	C8orf58
		HEBP2	CS	CCL16	C9orf140
		HEYL	CSNK1G1	CCL20	C9orf167
		HIST1H2BH	CSNK2A1	CCNB1	CA3
		HLA-DMB	CSNK2A1P	CCNB2	CA4
		HLA-DPA1	CSTF3	CCNE1	CA5A
		HLA-DRA	CTDSP2	CCNL1	CA5B
		HLA-DRB6	CTDSPL	CCNYL1	CAB39L
		HMBS	CTNNAL1	CD40	CABYR
		HOXA5	CTNND2	CD47	CACHD1
		HOXA6	CTSF	CD59	CACNA2D3
		HPRT1	CTSH	CD68	CACNB3
		HSD11B2	CTSS	CDA	CADM1
		HSPC159	CTSZ	CDC2L6	CALB2
		HTRA2	CWF19L2	CDC42EP2	CALCR
		ICAM4	CX3CL1	CDC5L	CALU
		IDS	CXCL10	CDC7	CAMK1D
		IFNGR1	CXorf57	CDCA4	CAMK2D
		IFT122	CYP21A2	CDCA5	CAMK2G
		IKZF1	CYP2A6	CDK9	CAMK2N1
		IL11RA	CYP3A43	CDKN1B	CAMLG
		IL1RAPL1	CYR61	CDKN2B	CAMSAP1L1
		INF2	CYTH1	CDR2	CANT1
		INS-IGF2	CYTH2	CDV3	CAPNS1
		ITLN1	D2HGDH	CECR5	CARD10
		JAZF1	DAAM1	CEP192	CARM1
		KCNH2	DAZAP2	CES1	CART1
		KCNK6	DCAKD	CES2	CASC3
		KCTD3	DCTD	CES4	CASP1
		KEL	DDHD2	CES8	CASP9
		KIAA1598	DDRGK1	CETN3	CASQ2
		KIAA1737	DDX1	CFHR3	CAT
		KIF13B	DDX12	CFHR5	CBFA2T2
		KIF6	DDX17	CFL2	CBL
		KLF5	DDX21	CHAD	CBLB
		KLHDC8B	DEF8	CHCHD10	CBX1
		KLHL3	DENND4C	CHDH	CBX2
		KLRB1	DENR	CHMP1B	CCBE1
		KRT1	DEPDC6	CHMP2A	CCDC101
		KRT13	DGCR6	CHMP4B	CCDC123
		LAGE3	DGKA	CHMP4C	CCDC136
		LAMA4	DGUOK	CHST9	CCDC151
		LAT	DHODH	CHURC1	CCDC153
		LDB2	DHRS1	CIDEC	CCDC45

		LECT2	DHRS3	CITED4	CCDC56
		LEPR	DHRS4L2	CLCF1	CCDC6
		LILRB2	DHX37	CLDN23	CCDC84
		LILRB5	DIO3	CLMN	CCDC86
		LINGO1	DIRAS3	CLP1	CCKBR
		LIPC	DLG3	CMBL	CCNB1IP1
		LMNB1	DNAH14	CNBP	CCND2
		LMO2	DNAJB1	CNDP1	CCNG1
		LOC100129902	DNAJC14	CNPY3	CCNJL
		LOC100130556	DNAJC18	COL27A1	CCNK
		LOC100130624	DNAJC27	COL3A1	CCS
		LOC100130746	DNAL1	COPZ2	CD163
		LOC100131164	DNMT3L	COQ5	CD24
		LOC100132499	DOHH	COX4I1	CD320
		LOC100133545	DOPEY1	COX5A	CD9
		LOC100133609	DPAGT1	CP	CD99
		LOC100133678	DPH2	CPN2	CDC14A
		LOC100134424	DPH3	CPSF3	CDC42SE1
		LOC147727	DPY30	CREB5	CDH1
		LOC200030	DPYSL2	CRELD2	CDH10
		LOC387686	DSCR6	CRIP1	CDH24
		LOC388654	DSE	CRIP2	CDH3
		LOC391132	DSN1	CRP	CDK5RAP3
		LOC391769	DUS4L	CRY1	CDK7
		LOC392288	DUXA	CRYAA	CDKN1C
		LOC439949	DUXAP3	CRYM	CDO1
		LOC440570	DVL2	CSDA	CDR2L
		LOC440731	ECHDC3	CST3	CDS1
		LOC441018	ECM2	CSTF2	CDYL
		LOC441081	EEF1B2	CTBS	CELSR2
		LOC641768	EFNB3	CTH	CENPJ
		LOC642073	EGLN2	CTSO	CENTG3
		LOC642113	EGR1	CUTC	CEP55
		LOC642469	EI24	CUX2	CEPT1
		LOC643008	EIF2AK2	CWF19L1	CERCAM
		LOC643431	EIF2S2	CXCL6	CGA
		LOC644852	EIF3E	CYB561D2	CGB1
		LOC644928	EIF3H	CYLD	CHCHD2
		LOC645284	EIF4B	CYP1A2	CHCHD7
		LOC646791	EIF4E2	CYP2A7	CHCHD9
		LOC647579	EIF6	CYP2B6	CHD4
		LOC648399	ELAVL3	CYP2C18	CHES1
		LOC649396	ELOVL4	CYP2C19	CHFR
		LOC651309	ENPP1	CYP2C8	CHM
		LOC651816	EPHA8	CYP2C9	CHMP5
		LOC653907	EPHB3	CYP2D6	CHST12
		LOC729687	EPHX2	CYP2D7P1	CHST13
		LOC729870	EPRS	CYP2E1	CHST15
		LOC730525	EPS15	CYP2U1	CHST3
		LOC731049	ERBB3	CYP3A4	CHSY1
		LOC732165	ERCC2	CYP4F11	CIB2
		LOC732316	ERO1LB	CYP4F2	CISD1
		LOC732419	ESD	CYP4F22	CKAP4
		LRMP	ETFA	CYP8B1	CKB
		LRPAP1	ETNK2	CYTSA	CKMT1A
		LRRC17	EVC	CYYR1	CLCNKA

		LST1	EXOC1	DAB2	CLDN1
		LXN	EXOC5	DAK	CLDN10
		LY96	EXOSC10	DAO	CLDN18
		LYL1	EXOSC5	DAXX	CLDN6
		LYVE1	EXOSC7	DBNDD1	CLEC2D
		MAGEA12	EXTL2	DBR1	CLIP3
		MAP3K8	F10	DCPS	CLN8
		MAP7	FABP5L2	DCTN6	CMTM4
		MARCH3	FADD	DCUN1D3	CMTM8
		MARCO	FAF1	DCXR	CNIH4
		MAST3	FAHD1	DDIT3	CNN2
		MED16	FAHD2B	DDT	COBLL1
		MEP1A	FAM108B1	DDTL	COG5
		MFNG	FAM110A	DDX19B	COG6
		MGC13057	FAM127C	DDX28	COL11A1
		MGST3	FAM162A	DEFB1	COL12A1
		MINPP1	FAM173A	DEGS1	COL4A2
		MIR1976	FAM173B	DEPDC1B	COL4A5
		MPP1	FAM176B	DEPDC7	COL4A6
		MPP5	FAM183A	DERL1	COL5A1
		MRPS7	FAM195A	DERL2	COL6A1
		MS4A6A	FAM20A	DET1	COL6A3
		MS4A7	FAM20B	DEXI	COL7A1
		MTFR1	FAM36A	DGCR5	COLEC12
		MUC6	FAM64A	DHRS2	COMMD1
		MUSTN1	FAM69A	DHRS9	COPA
		MXI1	FAM86A	DHTKD1	COPG
		MYB	FAM8A1	DHX58	CORO2A
		MYL4	FAM96B	DIAPH3	COX10
		MYT1	FANCB	DIDO1	COX7B
		MZF1	FAR1	DKFZp779M0652	CPD
		NAPEPLD	FAR2	DMRTA1	CPE
		NBPF14	FBXL15	DNAH1	CPNE3
		NCKAP1L	FBXO3	DNAJB4	CPS1
		NCOA7	FBXO45	DNAJC25	CPSF2
		NDUFA2	FDX1	DNPEP	CPXM2
		NFE2	FERMT1	DNTTIP2	CPZ
		NKD2	FGL1	DOCK7	CRABP2
		NMNAT3	FHOD3	DONSON	CREB3L3
		NOS3	FIP1L1	DPM1	CRH
		NP	FKBP4	DPP7	CRIM1
		NPL	FLJ10081	DSCC1	CRISPLD1
		NR2F1	FLJ10357	DUS2L	CRISPLD2
		NUDT14	FLJ10374	DUSP1	CRMP1
		OAT	FLJ10661	DUSP10	CRSP2
		OLFM1	FLJ10916	DUSP16	CRYZL1
		OSBP	FLJ11783	DUSP22	CSDE1
		OSBP2	FLJ13305	DYM	CSF2RA
		P2RY13	FLJ20699	DYNLL2	CSNK1G3
		P4HA2	FLJ22531	DYNLT3	CSNK2A2
		PAFAH1B3	FLJ38717	E2F7	CSRNP2
		PAK2	FLJ41484	EAPP	CSRP1
		PANK2	FLJ44124	EBNA1BP2	CST1
		PANX2	FLJ45244	ECGF1	CST4
		PCNT	FLJ46309	ECHDC1	CST6
		PECAM1	FLRT3	ECHS1	CTHRC1

		PERP	FMO4	ECT2	CTNNA1
		PIGQ	FNBP1	ECT2L	CTPS2
		PIN1	FOLH1	EEPD1	CTSB
		PKP4	FOLH1B	EFEMP2	CTSL1
		PLAC8	FOXH1	EFHD1	CTSL2
		PLCB2	FRAG1	EGFR	CUL4B
		PLEK	FRAP1	EGLN1	CXCL16
		PLOD3	FRAT1	EIF2B5	CXCR7
		PNMA3	FREM1	EIF2S1	CXorf45
		POLE3	FSIP1	EIF5	CXXC5
		POLS	FST	ELAC1	CYB5D1
		PPAPDC2	FTHL11	ELK4	CYB5R2
		PPM1F	FTHL2	ELL	CYBASC3
		PPOX	FYN	ELL3	CYC1
		PPP2R2B	GALC	ELOVL5	CYCS
		PPP3CC	GALNT10	ELOVL6	CYFIP1
		PPP4R1	GALNT3	ENDOG	CYP11A1
		PRDX2	GATA4	ENPP7	CYP1B1
		PRO0628	GATM	ENTPD5	CYP27A1
		PRR14	GATS	ENTPD8	CYP2S1
		PRR15L	GBE1	EPB41L2	CYP4X1
		PRR5	GCK	EPB41L4B	D4S234E
		PRRG4	GCKR	EPB41L5	DACT1
		PRSS8	GCN1L1	EPHA2	DAD1L
		PSD	GDE1	EPS8	DAG1
		PTMA	GDI1	ERAP2	DBNDD2
		PTPN11	GDI2	ERCC6L	DCAF10
		PTS	GDPD1	ERGIC1	DCBLD2
		PYGB	GGT2	ERLIN2	DCP1B
		RAB3IL1	GGTLC1	ERN1	DCTN2
		RABGEF1	GHITM	ERO1L	DDAH1
		RAC2	GINS4	ESM1	DDAH2
		RAD23A	GIT1	ESPNL	DDEF2
		RAD51C	GJC1	ESRRA	DDIT4
		RAMP2	GK	ESRRAP2	DDIT4L
		RAPGEF5	GK5	ETNK1	DDR1
		RAPH1	GLE1	ETS2	DDX39
		RASGRP2	GLG1	EXOSC6	DDX42
		RASIP1	GLRX2	F8A1	DDX47
		RBM47	GLS2	FAAH	DDX50
		RBP1	GLT8D2	FADS2	DEAF1
		RBP2	GMEB1	FAHD2A	DENND2D
		RBX1	GMPR2	FAIM	DENND4A
		RCCD1	GNAS	FAM100A	DERA
		RELN	GNB5	FAM102A	DFFA
		RETN	GOLT1A	FAM105B	DFNA5
		RFESD	GON4L	FAM107B	DFNB59
		RFX1	GPATCH4	FAM134B	DHX30
		RGS10	GPR141	FAM13A	DHX40
		RGS18	GPR146	FAM160B1	DHX57
		RHAG	GPX3	FAM174A	DIABLO
		RHCE	GRHPR	FAM178A	DIO2
		RNF123	GRINA	FAM35A	DIP2B
		RNF145	GRIP2	FAM86B1	DIRAS2
		RNF213	GRN	FAM91A1	DIRC2
		RNPEP	GSG2	FAM96A	DKFZP586I1420

		RP5-1022P6.2	GTF3C5	FANCA	DKFZp761P0423
		RPIA	GTSE1	FANCD2	DKK1
		RPL14	H2AFY2	FANCG	DKK3
		RPL36A	HAUS4	FAS	DLD
		RPS26L	HCCS	FASTKD5	DLG2
		RPUSD3	HCFC1R1	FBL	DLG5
		RRAGC	HCG2P7	FBLN2	DLK2
		RSPH3	HDAC7A	FBLN7	DLX3
		RUNDC3A	HDHD1A	FBXL3	DLX5
		S100A10	HEATR5B	FBXL6	DMGDH
		S100A12	HEBP1	FBXO25	DMKN
		S100A8	HERC4	FBXO31	DMTF1
		SAMSN1	HIAT1	FBXO46	DNAH2
		SAPS2	HIF1AN	FBXO6	DNAJB12
		SCGB3A1	HINFP	FBXW4	DNAJB6
		SDPR	HINT1	FCAMR	DNAJC10
		SELL	HIST1H2BD	FEM1B	DNAJC13
		SELM	HIST2H2AA4	FEM1C	DNAL4
		SERPINA13	HIST2H2AC	FGF2	DNMT3B
		SERPINB9	HIST2H2BE	FGGY	DOCK1
		SERPINI1	HKR1	FHDC1	DOCK11
		SETD8	HLA-C	FICD	DPEP1
		SGIP1	HMGA1	FIS	DPM3
		SH2D3C	HMGB3	FKBP5	DPP8
		SH2D4A	HNRNPD	FLJ10986	DPPA3
		SHANK3	HNRNPF	FLJ12078	DPPA4
		SHD	HNRNPR	FLJ20581	DPY19L1
		SLA2	HNRPH3	FLJ20674	DPY19L4
		SLC13A5	HOXB8	FLJ38482	DPYS
		SLC17A2	HRG	FLJ42289	DPYSL3
		SLC1A4	HS6ST2	FLVCR2	DRG2
		SLC22A25	HSPA1A	FMO3	DSC2
		SLC22A4	HSPBL2	FNDC4	DSG2
		SLC25A37	HTR2B	FOSB	DTX3
		SLC25A39	HUWE1	FOSL1	DULLARD
		SLC26A3	IFI30	FOSL2	DUOX1
		SLC2A10	IHH	FOXA2	DYNC1I1
		SLC40A1	IL13RA1	FOXD2	DYNC1I2
		SLC5A11	IL17RB	FOXP1	DYNC1LI2
		SLC6A9	IL17RC	FRYL	DYRK3
		SLC9A9	IL18R1	FSTL3	ECD
		SMC2	IL8	FTH1	EDARADD
		SMC4	IMP4	FTSJD2	EDC3
		SMOX	INHA	FVT1	EDG4
		SNAP25	INO80C	G0S2	EDN1
		SNCA	IPW	GABPB2	EDNRB
		SNHG3-RCC1	IQCC	GADD45A	EEF1A1
		SNORD36C	IRAK1	GADD45B	EFEMP1
		SNRPF	IRF2BP2	GAK	EFNB2
		SNRPN	IRF5	GALK2	EFS
		SOBP	ISM1	GAS8	EIF2B4
		SOX18	ITCH	GBP1	EIF4A2
		SOX6	ITFG1	GBP3	EIF4A3
		SPI1	ITFG3	GBP7	EIF4G3
		SPP1	ITGB1BP1	GCC1	EIF4H
		SPTA1	ITSN1	GCLM	ELAVL1

		SRRD	JDP2	GDF15	ELMOD1
		ST6GALNAC4	JTB	GEM	EME1
		STAB2	KCNJ16	GFOD2	EMID2
		STAT5B	KCNJ8	GFRA1	ENAH
		STRADB	KCNS1	GGNBP2	ENC1
		SUMF2	KIAA0363	GHR	ENO2
		SYNGR1	KIAA0556	GJB2	ENPEP
		TAL1	KIAA0907	GJB3	ENSA
		TBC1D10A	KIAA1191	GLIPR1	ENTPD4
		TCF20	KIAA1267	GLOD5	EPAS1
		TCF21	KIAA1285	GLT25D1	EPCAM
		TCTEX1D1	KIAA1328	GLYATL1	EPHA1
		TESC	KIAA1632	GMPPB	EPHA4
		TFDP1	KIDINS220	GNE	EPM2AIP1
		TFDP2	KIF22	GNMT	EPN3
		TFRC	KIF2A	GOLSYN	EPSTI1
		TGM2	KLF15	GPD1	ERC1
		TMCO6	KLF8	GPT	ERCC1
		TMED4	KLHL2	GPX2	ERCC5
		TMED9	KRCC1	GPX4	ESPN
		TMEM14B	KRT17	GRB14	ESRRG
		TMEM14D	KRTCAP2	GRIA3	ETFB
		TMEM167B	LACTB	GRPEL1	EVL
		TMEM170B	LAMA3	GSTM4	EWSR1
		TMEM173	LANCL2	GSTP1	EXD2
		TMEM2	LASS2	GSTT2	EXOC2
		TMEM43	LASS5	GSTT2B	EXOC7
		TMPRSS9	LBR	GTPBP4	EXPH5
		TNFAIP8L3	LCLAT1	H2AFX	EXTL3
		TRAF4	LCMT2	H3F3B	EYA1
		TRIM10	LCOR	HACE1	EZR
		TRIM8	LDHD	HAO2	F12
		TSPAN32	LGALS3BP	HAS3	F2
		TSPAN7	LGI2	HBEGF	F3
		TTC25	LIME1	HBP1	FAM100B
		TTTY11	LIN9	HCG4	FAM108C1
		TUBB1	LMNA	HCP5	FAM114A2
		TUBB4Q	LOC100127988	HDAC1	FAM116B
		TXNDC16	LOC100128326	HECTD1	FAM118A
		UBAC1	LOC100128477	HECTD3	FAM119A
		UBXN6	LOC100128510	HEPACAM	FAM120A
		UGCG	LOC100128688	HERC2	FAM120B
		UNC84A	LOC100128731	HEXIM1	FAM123A
		UROS	LOC100129580	HGD	FAM127B
		VCAM1	LOC100129742	HIBADH	FAM129B
		VNN2	LOC100130229	HIC2	FAM149A
		VWA1	LOC100130557	HIGD1A	FAM156A
		VWF	LOC100130633	HIP1R	FAM156B
		WAS	LOC100130905	HIST1H2AC	FAM158A
		WDR40A	LOC100131096	HIST1H2BC	FAM164A
		WNK4	LOC100131541	HIST1H3D	FAM172A
		XK	LOC100131810	HK1	FAM181B
		XLKD1	LOC100131835	HLA-A	FAM184A
		XPO7	LOC100131866	HLA-B	FAM189B
		YBX1	LOC100131940	HLA-E	FAM195B
		YOD1	LOC100132323	HLA-F	FAM44B

		YPEL2	LOC100132510	HLF	FAM46B
		YPEL4	LOC100132717	HLTF	FAM48A
		ZADH2	LOC100133077	HLX	FAM49A
		ZBTB17	LOC100133578	HM13	FAM60A
		ZDHHC23	LOC100133972	HMOX1	FAM62B
		ZEB2	LOC100134266	HNF4A	FAM65A
		ZFAND3	LOC134997	HNRNPH2	FAM65B
		ZMAT2	LOC148430	HNRPUL1	FAM80B
		ZNF35	LOC149448	HNRPUL2	FAM83B
		ZNF443	LOC152195	HOMER2	FAM84B
		ZNF696	LOC201175	HPCAL1	FAM89A
		ZNF697	LOC202227	HPGD	FANCE
		ZWINT	LOC203547	HPS6	FARP1
			LOC255326	HPSE	FAT1
			LOC283953	HSD11B1	FAT3
			LOC285016	HSD17B12	FBLIM1
			LOC285359	HSD17B13	FBLN1
			LOC285900	HSD17B6	FBLN5
			LOC286002	HSD3B7	FBN2
			LOC341315	HSPB1	FBN3
			LOC341457	HSPC111	FBXL10
			LOC387723	HTATIP2	FBXL14
			LOC387841	HYI	FBXL2
			LOC388076	ID2	FBXL21
			LOC388524	IFFO2	FBXO2
			LOC388681	IFI27	FBXO21
			LOC389072	IFI44	FBXO4
			LOC390956	IFIH1	FBXW8
			LOC391833	IFIT2	FER1L3
			LOC392221	IFIT3	FGA
			LOC399900	IFNAR2	FGD1
			LOC400389	IFT52	FGD6
			LOC400464	IGDCC4	FGF11
			LOC401357	IGF1	FGG
			LOC401588	IGFBP1	FH
			LOC439953	IGFBP2	FHL2
			LOC440396	IGSF8	FHL3
			LOC440585	IK	FIG4
			LOC440589	IKZF5	FILIP1
			LOC440748	IL15	FIT1
			LOC440991	IL15RA	FKBP14
			LOC441114	IL1R2	FKBP2
			LOC442609	IL1RL1	FLJ10996
			LOC641765	IL1RN	FLJ12684
			LOC641825	IL28RA	FLJ14712
			LOC641844	IL32	FLJ16779
			LOC642282	IL5RA	FLJ22184
			LOC642299	IL6R	FLJ30428
			LOC643031	IMMP2L	FLJ31568
			LOC643300	INHBE	FLJ42957
			LOC643433	INSIG1	FLJ44342
			LOC643438	INTS12	FLNA
			LOC644033	INTS9	FLNB
			LOC644101	IPO11	FLNC
			LOC644919	IRF1	FLRT2
			LOC644990	IRF7	FMNL2

			LOC645001	IRS2	FMO5
			LOC645430	ITGA9	FMOD
			LOC645452	ITGB4BP	FN1
			LOC645693	ITGB7	FNBP1L
			LOC645897	ITPKC	FNDC3B
			LOC645963	JAK1	FNTA
			LOC646272	JAM2	FOLR1
			LOC646282	JMJD6	FRAS1
			LOC646845	JMJD7	FRAT2
			LOC647009	JMY	FREM2
			LOC647081	JOSD1	FRMD6
			LOC647150	JUB	FRS3
			LOC647276	JUN	FRZB
			LOC647718	JUND	FSTL1
			LOC648024	KCNIP3	FTHL16
			LOC649169	KCNK5	FTHL7
			LOC649447	KIAA0232	FTL
			LOC649503	KIAA0406	FUCA1
			LOC649639	KIAA0427	FUCA2
			LOC649859	KIAA0564	FUT4
			LOC649873	KIAA0586	FXR1
			LOC650128	KIAA0892	FZD2
			LOC650157	KIAA1543	FZD3
			LOC650737	KIAA1671	FZD4
			LOC651198	KIAA1875	FZD6
			LOC651202	KIF11	FZD7
			LOC651285	KIF14	G6PC3
			LOC651987	KIF3B	GAB2
			LOC652470	KLF10	GABRP
			LOC652826	KLF11	GAD1
			LOC652968	KLF4	GALNT11
			LOC653066	KLF6	GALNT6
			LOC653073	KLF9	GALNTL1
			LOC653080	KLHDC3	GALT
			LOC653383	KLHL15	GAPDH
			LOC653505	KLHL21	GAPDHL6
			LOC653752	KLHL29	GAR1
			LOC654350	KLHL5	GARNL4
			LOC727820	KRR1	GARS
			LOC727825	KTELC1	GAS6
			LOC727901	KTI12	GAS7
			LOC727984	KTN1	GATA2
			LOC727987	LAD1	GATA3
			LOC728069	LAMB3	GATAD1
			LOC728188	LAMC3	GATAD2A
			LOC728441	LAMP1	GC
			LOC728452	LAMP2	GCDH
			LOC728499	LAP3	GCGR
			LOC728554	LARP1	GCM1
			LOC728620	LARP1B	GCNT1
			LOC728635	LBP	GDF3
			LOC728802	LDHA	GFPT2
			LOC729009	LDLR	GGT1
			LOC729301	LEMD3	GGTLC2
			LOC729340	LGALS1	GJA1
			LOC729375	LGALS4	GLA

			LOC729408	LGALS8	GLB1
			LOC729446	LGTN	GLDN
			LOC729466	LIMS1	GLI3
			LOC729535	LIMS2	GLMN
			LOC729603	LINCR	GLRX
			LOC729660	LIPT1	GLS
			LOC730004	LMO7	GLTSCR1
			LOC730313	LOC100128191	GNA12
			LOC730908	LOC100128392	GNB1
			LOC731542	LOC100128525	GNL2
			LOC88523	LOC100128585	GNPAT
			LOC91431	LOC100128888	GNPDA1
			LOC91664	LOC100129086	GOLGA3
			LPAR1	LOC100129424	GPAM
			LPCAT2	LOC100129522	GPC4
			LPIN2	LOC100129681	GPN1
			LRRC2	LOC100129781	GPR161
			LUC7L	LOC100130092	GPR162
			LYPLAL1	LOC100130522	GPR98
			LYSMD1	LOC100130542	GPRC5A
			M6PR	LOC100130886	GPS1
			MAF	LOC100130919	GPS2
			MAGOH	LOC100132552	GPSM1
			MANEA	LOC100132553	GPX8
			MAP1LC3B2	LOC100132655	GRAMD2
			MAP4K2	LOC100132938	GRAMD3
			MAPK3	LOC100133511	GRB7
			MARCKS	LOC100133591	GRHL2
			MAST4	LOC100133697	GRHL3
			MASTL	LOC100134108	GSTA3
			MAT1A	LOC100134144	GSTO1
			MBNL3	LOC100134407	GTF2IP1
			MDH2	LOC100134584	GTF2IRD1
			ME1	LOC143941	GTF3A
			MED12	LOC145837	GTF3C1
			MED28	LOC147710	GTF3C3
			MED31	LOC152217	GTPBP3
			MED7	LOC158345	GUCA1A
			MEGF9	LOC197350	GUCY1A3
			MESP1	LOC221710	GUCY2C
			METRN	LOC255167	GUK1
			MFAP4	LOC284988	GXYLT1
			MFF	LOC286297	HAND1
			MFSD3	LOC286444	HAPLN1
			MGC26718	LOC387934	HARS2
			MGC39900	LOC388503	HAS1
			MGC40489	LOC388564	HDAC7
			MGC5457	LOC389141	HDGFRP3
			MGC61598	LOC389386	HEATR6
			MGC70857	LOC389672	HELZ
			MLEC	LOC390251	HEPH
			MLKL	LOC399491	HES2
			MLLT10	LOC400578	HES6
			MLLT6	LOC400721	HEY1
			MLN	LOC400750	HHEX
			MMP25	LOC401127	HINT2

			MOCS1	LOC401317	HINT3
			MOGAT3	LOC401537	HIST1H2BK
			MPDZ	LOC402560	HIST1H4K
			MPPE1	LOC440055	HK2
			MRGPRX1	LOC440345	HKDC1
			MRGPRX4	LOC440359	HMGCL
			MRPL14	LOC440498	HMGCS2
			MRPL15	LOC441013	HMGN4
			MRPL43	LOC441453	HNMT
			MRPL45	LOC441896	HNRNPA1
			MRPS11	LOC641983	HNRNPA1L2
			MRPS12	LOC642076	HNRNPU
			MRPS18A	LOC642628	HNRPA1L-2
			MRS2	LOC642755	HNRPA1P4
			MSH6	LOC643319	HNRPDL
			MSRB2	LOC643856	HOMER1
			MSRB3	LOC644132	HOMER3
			MTF2	LOC644322	HOPX
			MTIF3	LOC644422	HOXB4
			MTPN	LOC644914	HOXB5
			MTRR	LOC645018	HOXB7
			MTX3	LOC645094	HOXC6
			MUC2	LOC645381	HOXC8
			MXD1	LOC645489	HSBP1
			MYL3	LOC645522	HSD17B2
			MYL6B	LOC645969	HSD3B1
			MYO9B	LOC646109	HSN2
			N6AMT1	LOC646675	HSP90AA1
			NAALAD2	LOC646743	HSP90AB1
			NAGS	LOC646981	HSPA12A
			NAT11	LOC647856	HSPA4
			NBR2	LOC647859	HSPB8
			NDUFA1	LOC648249	HTR1E
			NDUFA10	LOC648517	HTRA4
			NDUFB2	LOC648682	HYAL1
			NEBL	LOC649076	ID1
			NEK3	LOC649495	IDH3B
			NENF	LOC649497	IDI1
			NEXN	LOC649553	IFI27L1
			NF1	LOC650280	IFI27L2
			NFIA	LOC650803	IFNAR1
			NGDN	LOC651143	IFT140
			NIF3L1	LOC652545	IFT74
			NIN	LOC652773	IGDCC3
			NISCH	LOC653158	IGFBP3
			NIT2	LOC653171	IGFBP5
			NKIRAS1	LOC653199	IGFBP6
			NLGN2	LOC653342	IGSF3
			NLGN4Y	LOC653458	IGSF5
			NNMT	LOC653610	IL17D
			NOL6	LOC653720	IL22RA1
			NPC1L1	LOC653879	IMP3
			NR1D2	LOC654244	INA
			NR2C1	LOC728014	INADL
			NR2C2	LOC728034	INPP1
			NR3C2	LOC728098	INTS2

			NRCAM	LOC728126	INTS6
			NRD1	LOC728138	INTU
			NRM	LOC728139	INVS
			NSDHL	LOC728160	IPO8
			NSF	LOC728208	IQCB1
			NSUN4	LOC728431	IQGAP1
			NT5C2	LOC728666	IRX2
			NT5DC2	LOC728811	IRX4
			NTF4	LOC728855	ISCU
			NUB1	LOC728908	ISL1
			NUBP1	LOC729666	ISM2
			NUDT2	LOC729816	ISOC1
			NUDT7	LOC729905	ISYNA1
			NUP107	LOC729970	ITFG2
			NUP133	LOC730020	ITGA2
			NUP62CL	LOC730029	ITGA3
			NXF1	LOC730051	ITGA5
			OCIAD1	LOC730256	ITGAL
			ODAM	LOC730324	ITGB1
			OGFOD1	LOC730417	ITGB2
			OLR1	LOC730534	ITGB4
			OPRL1	LOC730820	ITGB5
			ORMDL1	LOC730993	ITLN2
			OSBPL7	LOC730994	ITPR3
			OSBPL8	LOC730995	IVNS1ABP
			OSR1	LOC731314	IWS1
			OSTalpha	LOC731656	JARID1A
			OTUB2	LOC731878	JARID1D
			OXR1	LOC732007	JPH3
			P2RX4	LONP2	JUP
			PABPC3	LONRF3	KCNA6
			PAFAH1B2	LPIN1	KCNJ2
			PAICS	LPIN3	KCNK12
			PAK1	LRCH4	KCNMA1
			PAK4	LRG1	KCNMB4
			PAK6	LRP5L	KCTD12
			PBK	LRRC3	KCTD2
			PBX3	LRRC50	KDELC2
			PCDH10	LRRC8A	KDELR1
			PCDH17	LRRC8E	KDELR2
			PCDH18	LRRCC1	KDM3B
			PCDHA1	LTBP4	KDM4D
			PCDHGB3	LYSMD2	KDM5B
			PCGF2	MAFF	KHDC1
			PCMTD1	MAK16	KHDRBS1
			PCNP	MAN2B2	KIAA0040
			PCSK9	MAOA	KIAA0174
			PCYOX1	MAP1LC3A	KIAA0368
			PDCD6IP	MAP1LC3B	KIAA0672
			PDCL3	MAP2K7	KIAA0947
			PDE5A	MAP3K4	KIAA1024
			PDGFRL	MAPRE3	KIAA1217
			PDS5B	MARCH5	KIAA1324L
			PDZD2	MBIP	KIAA1429
			PDZK1IP1	MBOAT2	KIAA1549
			PELI1	MCCC1	KIAA1688

			PELO	MCL1	KIAA1967
			PES1	MCM3	KIF1A
			PEX11A	MEAF6	KIF26B
			PFKFB1	MED10	KIF27
			PGAP1	MELK	KIF3C
			PGM1	MFSD2	KIF5C
			PGM5	MFSD5	KIF7
			PGPEP1	MGAT2	KIFAP3
			PGRMC2	MGAT3	KITLG
			PHB	MGAT4B	KLB
			PHC2	MGC102966	KLC1
			PHF10	MGLL	KLHDC5
			PHF5A	MGRN1	KLHL24
			PHGDH	MGST1	KLHL28
			PHLDB1	MIA3	KLK6
			PI4K2B	MIF	KLK8
			PIGN	MIR1974	KLRG2
			PIGP	MIR1978	KMO
			PIK3AP1	MKLN1	KRT17P3
			PIK3C3	MLPH	KRT18P17
			PINX1	MLXIPL	KRT18P26
			PKIA	MLYCD	KRT18P42
			PLA2G16	MMAA	KRT19
			PLAT	MMP7	KRT23
			PLEKHG6	MND1	KRT7
			PLOD2	MNT	KRT75
			PLP2	MOCOS	KRT8
			PLSCR3	MORG1	KRT80
			PLXNA2	MOSC2	KRT8P9
			PMAIP1	MR1	KRTAP10-2
			PMPCB	MRLC2	KRTDAP
			PNMAL1	MRPL34	KSR1
			PNPLA4	MRPL52	L1TD1
			PNPLA6	MRPS15	L3MBTL
			POLD1	MRPS30	LAMA1
			POLD3	MSH2	LAMA5
			POLDIP2	MSH5	LAMB1
			POLR2A	MST1R	LAMC1
			POLR2G	MTCP1	LASS6
			POLR2J4	MTMR11	LATS2
			POMC	MUC20	LCAT
			POMP	MUPCDH	LCN15
			PON3	MUTYH	LCP1
			POP4	MVP	LDHB
			POU2F1	MX1	LEAP2
			PPA2	MYD88	LEF1
			PPAN	MYL12A	LEMD2
			PPAT	MYL6	LEPREL1
			PPHLN1	MYLK	LEPREL2
			PPIAL4A	MYO1B	LEPROTL1
			PPIE	MYOM1	LHB
			PPM1H	MYRIP	LHFP
			PPP1R3F	N4BP2L1	LHFPL2
			PPP1R7	NACC2	LHPP
			PPP1R9A	NADK	LIAS
			PPP2R1B	NAGK	LIFR

			PPP2R5A	NAMPT	LIG3
			PPT1	NAT2	LIMA1
			PRDX4	NBAS	LIMCH1
			PRICKLE3	NBPF20	LIMK2
			PRMT3	NCAPD3	LIN28
			PRMT5	NCAPG	LIN28B
			PROSC	NCRNA00092	LIX1L
			PROZ	NDN	LLGL1
			PRPF19	NDUFS2	LMAN2L
			PRPF38A	NECAP2	LMCD1
			PRTG	NEDD4	LMO1
			PSD4	NEFL	LMTK3
			PSMB3	NEK1	LOC100008589
			PSMD2	NEK2	LOC100127993
			PSMD6	NFATC1	LOC100128168
			PSMD7	NFE2L2	LOC100128771
			PSME2	NFIC	LOC100128975
			PSMG4	NFKB1	LOC100129034
			PTGR1	NFKB2	LOC100129144
			PTMS	NFKBIB	LOC100129550
			PTP4A1	NFKBIE	LOC100129637
			PTPDC1	NFYC	LOC100129958
			PTPLB	NGFRAP1	LOC100130921
			PTPRA	NHLRC2	LOC100131289
			PTPRE	NKX3-1	LOC100131471
			PUF60	NMT2	LOC100132526
			PUS7L	NNT	LOC100132535
			PVR	NOD2	LOC100132901
			RAB10	NOP14	LOC100133760
			RAB11FIP3	NOSIP	LOC100134587
			RAB35	NOTCH2NL	LOC146517
			RAB38	NOXA1	LOC151162
			RAB5C	NPAS2	LOC154761
			RAB7L1	NPIP	LOC158160
			RABGAP1	NPTX2	LOC255783
			RABL3	NR1I2	LOC283267
			RAD17	NR3C1	LOC283932
			RAD51AP1	NR4A2	LOC284422
			RAD54L	NRBP2	LOC284620
			RAI14	NRG1	LOC286467
			RALA	NRTN	LOC339535
			RALY	NSFL1C	LOC344595
			RALYL	NTN4	LOC346085
			RANBP3	NUDT1	LOC346887
			RAPGEF1	NUDT16	LOC388275
			RARB	NUDT18	LOC389168
			RASL11A	NUDT22	LOC389332
			RASSF5	NUDT5	LOC389342
			RBBP9	NUDT9	LOC391045
			RBCK1	NUF2	LOC392437
			RBM17	NUFIP2	LOC392787
			RBM5	NUP98	LOC392871
			REL	NXT1	LOC399804
			RERG	OAF	LOC399965
			RFX5	OASL	LOC400455
			RGS11	OCEL1	LOC400713

			RGS17	OCIAD2	LOC401074
			RILPL2	ODF3B	LOC401115
			RNF103	OGDHL	LOC401431
			RNF13	OGFR	LOC401720
			RNF170	OKL38	LOC402112
			RNF4	OPLAH	LOC440132
			RNF43	OPN3	LOC440349
			RNFT2	OPTN	LOC440905
			RNGTT	OR1S2	LOC440928
			RNH1	ORM1	LOC441061
			RNPS1	ORM2	LOC441131
			RNY3	OSGIN1	LOC442249
			ROM1	OSGIN2	LOC554235
			RP2	OTUD1	LOC55908
			RPH3AL	PALM2	LOC642412
			RPL12	PAN3	LOC642489
			RPL12P6	PAOX	LOC642590
			RPL13	PAPSS2	LOC643293
			RPL13A	PARK7	LOC643389
			RPL14L	PARP1	LOC644128
			RPL3	PARP10	LOC644150
			RPL34	PARP3	LOC644237
			RPL4	PARP8	LOC644612
			RPL8	PCCA	LOC644761
			RPP40	PCDH20	LOC645241
			RPS6KB1	PCF11	LOC645251
			RPS6KC1	PCGF1	LOC645317
			RQCD1	PCK1	LOC645431
			RSBN1	PCM1	LOC646043
			RSPO1	PCSK6	LOC646123
			RTN4IP1	PDLIM1	LOC646144
			RUFY1	PDLIM7	LOC646347
			RUNX2	PDRG1	LOC646630
			RUSC1	PEAR1	LOC646769
			RUVBL2	PER2	LOC646783
			RWDD2B	PEX13	LOC646786
			RYR3	PGAM1	LOC646942
			S100P	PGAM4	LOC647262
			S1PR1	PGK1	LOC647322
			SACS	PGM3	LOC647691
			SALL1	PGRMC1	LOC647949
			SALL4	PHAX	LOC647954
			SAP130	PHF19	LOC648390
			SBDS	PHYHD1	LOC648740
			SBSN	PICK1	LOC648814
			SCARF2	PID1	LOC648927
			SCGN	PIGR	LOC649009
			SCMH1	PIM3	LOC650369
			SDC1	PIP4K2A	LOC650832
			SDCCAG10	PIPOX	LOC651659
			SDHALP1	PITPNB	LOC652097
			SDHC	PKD1	LOC652577
			SDHD	PKIG	LOC652900
			SEC16A	PLA2G4C	LOC653188
			SEC61G	PLAGL1	LOC653269
			SELO	PLEKHF2	LOC653344

			SEMA3C	PLIN2	LOC653506
			SEMA5B	PLK4	LOC654042
			SERINC3	PLLP	LOC728060
			SERPINA10	PLS1	LOC728115
			SERPINB6	PMM1	LOC728226
			SERPINE3	PMM2	LOC728324
			SERPING1	PMPCA	LOC728532
			SERPINH1	PNKD	LOC728533
			SESN3	PNPLA2	LOC728537
			SETDB1	PNPLA7	LOC728661
			SF3B4	PNRC1	LOC728715
			SFRS2IP	POLA1	LOC728734
			SGK2	POLA2	LOC728755
			SGSM2	POLD4	LOC729102
			SH2B3	POLQ	LOC729222
			SH3BP1	POLR1C	LOC729279
			SH3YL1	POR	LOC729317
			SHC2	POU5F1P1	LOC729366
			SHCBP1	PPA1	LOC729423
			SHISA2	PPAP2B	LOC729679
			SHPRH	PPIF	LOC729686
			SIDT2	PPP1R11	LOC729760
			SIL1	PPP1R15B	LOC729841
			SIRPA	PPP2CB	LOC729843
			SIRT5	PPP3CB	LOC730024
			SIVA1	PPP4R4	LOC730278
			SIX4	PPPDE2	LOC731954
			SKIV2L2	PPRC1	LOC731985
			SLC16A10	PRAMEF17	LOC91461
			SLC16A12	PRKDC	LOC91661
			SLC17A3	PRLR	LOC92249
			SLC22A3	PRMT1	LOXL1
			SLC23A3	PRNP	LPAR2
			SLC25A10	PRR8	LPHN3
			SLC25A26	PRSS3	LRCH2
			SLC25A28	PSEN2	LRFN5
			SLC27A5	PSMA2	LRIG2
			SLC29A2	PSMA7	LRP2
			SLC30A10	PSMB8	LRP4
			SLC30A9	PSMB9	LRRC1
			SLC35A2	PSMC3IP	LRRC20
			SLC35B4	PSMC6	LRRC23
			SLC41A3	PSMD5	LRRC37B
			SLC4A8	PSME1	LRRC8D
			SLC6A4	PTAR1	LRRN1
			SMA4	PTH2R	LRRN2
			SMAD6	PUM2	LRRN4
			SMARCA4	PUS3	LSM12
			SMYD3	PZP	LSM14A
			SNCAIP	QKI	LSP1
			SNORA70B	R3HDM1	LTA4H
			SNTB1	RAB17	LUM
			SNX14	RAB20	LY6E
			SOCS2	RAB21	LYPD6
			SOCS3	RAB24	LYPD6B
			SOD3	RAB40B	M6PRBP1

			SP8	RAB40C	MAD2L2
			SPATA20	RAB43	MAFB
			SPATA5L1	RAB8A	MAGEE1
			SPG11	RAD23B	MAK10
			SPG20	RAMP1	MAMDC2
			SPG21	RAN	MAML1
			SPIC	RANBP10	MAN1A2
			SPRED1	RANBP3L	MAN2A2
			SPSB3	RANBP6	MANBA
			SRBD1	RAPGEF4	MANBAL
			SREBF1	RARRES2	MAOB
			SRPR	RARRES3	MAP1A
			SRPRB	RASD1	MAP1B
			SRRM1	RASSF4	MAP2
			SSBP3	RBBP8	MAP2K1IP1
			SSH1	RBP7	MAP2K3
			SSH2	RCC2	MAP3K1
			SSTR2	RCL1	MAP3K6
			SSX4	RDH14	MAP3K7
			STAT1	RDH16	MAP4K4
			STAT4	RELA	MAPK4
			STAT6	RELB	MAPK7
			STK36	RET	MAPKAPK3
			STK4	REXO1	MAPKBP1
			STUB1	RFC3	MAPRE1
			STXBP6	RFTN1	MARCH11
			STYK1	RFXAP	MARK1
			SUCLG2	RHEB	MARS
			SURF6	RHOB	MATN3
			SYN2	RHOD	MBD2
			SYPL2	RIOK1	MBNL1
			SYT13	RNASE1	MBOAT7
			SYT17	RNASEH1	MBTPS1
			SYTL1	RNASEH2A	MCM3AP
			TADA3	RND1	MCOLN2
			TAF1	RNF115	MED15
			TAF10	RNF125	MED20
			TAF5	RNF130	MED29
			TAF5L	RNF144B	MED6
			TARBP1	RNF148	MEGF6
			TAS2R10	RNF152	MEIS1
			TBC1D15	ROBLD3	MEIS2
			TBC1D3B	RORA	MEIS3P1
			TBCC	RORC	MERTK
			TBCD	RPA1	MEST
			TCEA1	RPA2	METAP1
			TCEB3	RPF1	METRNL
			TCF12	RPS27L	METTL2A
			TDP1	RPS3	MEX3A
			TERF1	RPS3A	MFAP2
			TEX10	RPS6KA2	MFAP5
			TGFBI	RRAD	MFGE8
			TGM3	RRAGA	MFSD10
			THAP10	RRAGD	MFSD11
			THBS4	RRAS	MFSD6L
			THEM4	RRM1	MGC18216

			THOC2	RRN3	MGC26356
			THY1	RTN4R	MGC45800
			TIGD6	RTP3	MID1
			TIMM23	RUNDC1	MIOS
			TIRAP	RUNDC3B	MIR1228
			TJAP1	RWDD2A	MIR205
			TLR1	S100PBP	MIR21
			TM2D1	SAAL1	MKKS
			TM9SF4	SAMD4A	MKL1
			TMBIM1	SAMD5	MLLT11
			TMCO7	SBDSP	MMACHC
			TMED5	SC4MOL	MMD
			TMED6	SCAMP4	MMGT1
			TMEM115	SCHIP1	MMP23B
			TMEM118	SCO2	MMP28
			TMEM147	SCP2	MMP9
			TMEM14A	SDCBP	MOBKL2B
			TMEM194	SDCBP2	MOGS
			TMEM200A	SDF4	MOSPD2
			TMEM205	SDS	MOSPD3
			TMEM52	SEC14L2	MPDU1
			TMEM55A	SEC61B	MPRIP
			TMEM93	SECISBP2	MPZL1
			TMTC2	SELS	MRFAP1L1
			TNFRSF10A	SEMA4D	MRGPRX3
			TNFRSF9	SENP7	MRPL13
			TNFSF14	SEPHS2	MRPL21
			TNK2	SEPT4	MRPL35
			TNNC1	SERINC2	MRPL46
			TOM1	SERPINB8	MRPL51
			TOPBP1	SERPINE1	MRPL54
			TP53	SERTAD1	MRPS17
			TPBG	SERTAD2	MRPS28
			TPX2	SESN2	MSI2
			TRAF3IP3	SETD2	MST4
			TRAPPC1	SETD6	MSX2
			TRAPPC4	SF3A2	MT1A
			TRIB2	SF3A3	MT1E
			TRIB3	SFRS5	MT1F
			TRIM45	SFT2D2	MT1G
			TRIM47	SFTPD	MT1M
			TRIP11	SGMS2	MT1X
			TRK1	SGOL1	MT2A
			TSC1	SGOL2	MTA1
			TSGA14	SH2D5	MTA3
			TSHZ2	SH3BP2	MTAP
			TSHZ3	SH3D19	MTHFD1L
			TSNAX	SH3GLB2	MTHFSD
			TSPAN33	SHB	MTM
			TSPAN4	SHFM1	MTMR10
			TSTA3	SHMT1	MTMR7
			TTC14	SHMT2	MTR
			TTC32	SIGIRR	MTSS1
			TTC38	SKAP1	MTUS2
			TTF2	SLC10A1	MUC15
			TTL	SLC12A4	MUC16

			TTPAL	SLC16A2	MUT
			TUBGCP3	SLC17A4	MXRA5
			TUG1	SLC20A2	MXRA7
			TXNIP	SLC22A1	MYCN
			UBA7	SLC22A18	MYL7
			UBAP2	SLC22A18AS	MYL9
			UBASH3B	SLC24A3	MYLIP
			UBE2Q2	SLC25A1	MYO10
			UBE4B	SLC25A22	MYO5A
			UBN2	SLC25A33	MYOF
			UBXN8	SLC25A4	MYOZ1
			UCKL1	SLC25A45	NAE1
			UGT1A9	SLC25A6	NALCN
			UGT3A1	SLC28A1	NAP1L1
			UNC45A	SLC2A6	NAPB
			UQCRFS1	SLC30A1	NAT13
			USP16	SLC31A1	NAT14
			USP30	SLC31A2	NAV1
			USP33	SLC35C1	NAV2
			USP39	SLC35D1	NCK2
			VIM	SLC41A2	NCOA3
			VKORC1	SLC44A3	NCOA6
			VPS39	SLC46A1	NCOR2
			VPS41	SLC46A3	NCRNA00085
			WDR13	SLC4A4	NCRNA00219
			WDR45	SLC9A8	NCSTN
			WDR55	SLPI	NDRG4
			WDR60	SMARCC2	NDUFA6
			WDR8	SMPD1	NDUFA9
			WTIP	SMPDL3A	NDUFB9
			XAF1	SNAPC4	NDUFC1
			XPA	SNIP1	NDUFV2
			XPNPEP2	SNORA64	NES
			XPO5	SNORA72	NETO2
			ZAK	SNORA7B	NEU1
			ZBTB9	SNORD16	NFAT5
			ZC3H12B	SNORD89	NFE2L3
			ZC3H4	SNRK	NFIX
			ZC3H5	SNRNP40	NFX1
			ZDHHC19	SNX10	NGF
			ZDHHC9	SNX5	NHS
			ZFP90	SOD2	NINL
			ZHX3	SORCS2	NIPA2
			ZKSCAN2	SPAG1	NKAIN4
			ZKSCAN4	SPAG9	NKX2-3
			ZMYM3	SPATA2L	NLGN1
			ZNF133	SPON2	NLGN4X
			ZNF135	SPR	NLRP12
			ZNF175	SPRED2	NLRP7
			ZNF193	SPSB1	NMNAT2
			ZNF197	SPTBN1	NOD1
			ZNF200	SPTBN5	NOTCH3
			ZNF232	SQSTM1	NPC2
			ZNF251	SRD5A2	NPEPPS
			ZNF252	SRXN1	NPPB
			ZNF256	SSH3	NPTN

			ZNF260	SSR2	NPY
			ZNF266	ST3GAL4	NQO1
			ZNF285A	STAP2	NR1H4
			ZNF3	STARD10	NR5A2
			ZNF300	STARD5	NRAS
			ZNF33A	STAT3	NRXN3
			ZNF362	STIL	NSMAF
			ZNF420	STK19	NSMCE1
			ZNF436	STK24	NSUN6
			ZNF490	STMN1	NSUN7
			ZNF493	STX11	NT5C3
			ZNF500	STX5	NTF3
			ZNF519	SULT2A1	NTF5
			ZNF521	SUPV3L1	NTNG1
			ZNF541	SURF4	NTRK2
			ZNF543	SUSD3	NUAK1
			ZNF544	SUSD4	NUDT11
			ZNF594	SUZ12	NUMB
			ZNF598	SYNCRIP	NUP35
			ZNF607	SYPL1	NUP62
			ZNF613	SYVN1	NUP85
			ZNF615	TACO1	NXN
			ZNF616	TAF13	ODF2L
			ZNF630	TAOK3	ODZ3
			ZNF653	TAP1	ODZ4
			ZNF658	TAPBPL	OFD1
			ZNF700	TASP1	OGG1
			ZNF711	TAT	OLFML3
			ZNF800	TBC1D16	ONECUT2
			ZNF84	TBC1D8	OR7E156P
			ZNFX1	TBL1X	ORMDL3
			ZRSR2	TBL2	OSBPL10
			ZSWIM6	TBX15	OSBPL3
				TCF25	OSBPL5
				TESK1	OTC
				TFG	OTUB1
				TGFA	OTX2
				THOC5	OVOL1
				THRSP	OVOL2
				TIA1	P2RX2
				TICAM1	P2RY6
				TIFA	P4HA1
				TIMELESS	PABPC4L
				TIMM22	PACS2
				TIMP3	PAH
				TIPARP	PALB2
				TK1	PALLD
				TK2	PALMD
				TM6SF2	PAM
				TMEM105	PANK1
				TMEM107	PAPOLA
				TMEM134	PAQR3
				TMEM139	PARD6G
				TMEM16A	PARM1
				TMEM185A	PARP11
				TMEM185B	PARP6

				TMEM22	PBX1
				TMEM27	PCCB
				TMEM37	PCDH7
				TMEM45A	PCDHA3
				TNFAIP8	PCDHB17
				TNIP1	PCDHB2
				TNKS1BP1	PCDHB3
				TNRC6B	PCDHB5
				TOB1	PCNX
				TOP2A	PCNXL2
				TOR1AIP1	PCOLCE
				TP53INP1	PCP4
				TP53INP2	PCSK5
				TPD52L1	PCSK7
				TPRG1L	PDE4D
				TPST1	PDGFRA
				TRIB1	PDPN
				TRIM22	PDPR
				TRIM25	PDXP
				TRIM31	PDZK1
				TRIM35	PDZRN3
				TRIO	PEA15
				TRIP13	PECI
				TROAP	PEF1
				TRPM8	PELI3
				TSG101	PEMT
				TSKU	PFDN1
				TSPYL2	PFKFB4
				TTC36	PFN2
				TTC39C	PGCP
				TTC5	PGF
				TTK	PGLS
				TTLL3	PGLYRP2
				TUBA1B	PGS1
				TUBA4A	PHACTR2
				TUBB	PHB2
				TUBB6	PHC1
				TUSC1	PHF16
				TWF2	PHF17
				TXNDC11	PHF20
				TXNDC17	PHLDA3
				UAP1	PHPT1
				UBAP1	PHYH
				UBC	PHYHIPL
				UBD	PIAS3
				UBE2J1	PIBF1
				UBE2L3	PIGG
				UBIAD1	PIK3C2G
				UBP1	PITX1
				UBQLN1	PITX2
				UBQLN3	PKD2
				UBR2	PKDCC
				UBTD1	PKIB
				UBXN11	PKM2
				UFM1	PKN1
				UFSP1	PKNOX1

				UGT1A1	PLA1A
				UGT1A10	PLA2G10
				UGT1A4	PLAC2
				UGT1A6	PLAU
				UGT1A7	PLCB1
				UGT2B11	PLCD1
				UGT2B15	PLCE1
				UGT2B17	PLCXD3
				UHRF1BP1L	PLD2
				UMPS	PLD3
				UNC84B	PLD5
				URB2	PLEKHA5
				URG4	PLEKHA9
				USP2	PLEKHG3
				USP24	PLSCR4
				USP34	PLXDC2
				USP38	PMP22
				USP6NL	PNCK
				UST	PNMA6A
				UTP3	PNPO
				VAPA	PODXL
				VASN	POFUT2
				VEGFA	POLE
				VISA	POLR2C
				VNN1	POLR2J3
				VRK3	POMT2
				WDR21A	PPARD
				WDR43	PPFIA1
				WDR67	PPFIBP1
				WDR7	PPIL3
				WDR74	PPP1CC
				WTAP	PPP1R13L
				WWC1	PPP1R15A
				WWP2	PPP1R2
				XDH	PPP1R3C
				XRCC3	PPP2R1A
				YES1	PPP2R3B
				YIPF6	PPP2R5C
				YRDC	PPP2R5E
				YWHAB	PQLC1
				ZBTB16	PRAGMIN
				ZBTB2	PRDM1
				ZBTB43	PRDM7
				ZBTB7B	PRICKLE1
				ZC3H12A	PRKAA1
				ZC3HAV1	PRKCH
				ZCCHC24	PRKCI
				ZCCHC6	PRKCZ
				ZDHHC1	PRKD1
				ZDHHC11	PRKD2
				ZFAND2A	PRMT2
				ZFAND6	PRMT7
				ZFP36	PROCA1
				ZGPAT	PRODH2
				ZMYND12	PROM1
				ZMYND15	PROM2

				ZNF213	PRPH
				ZNF22	PRPS1
				ZNF226	PRPSAP1
				ZNF24	PRR11
				ZNF274	PRSS12
				ZNF295	PRSS16
				ZNF364	PRSS22
				ZNF394	PRSS23
				ZNF593	PSMB5
				ZNF622	PSME3
				ZNF654	PSPC1
				ZNF664	PSTPIP2
				ZNF668	PTBP1
				ZNF746	PTCD1
				ZNF787	PTDSS2
				ZNF788	PTGES
				ZNF828	PTGIS
				ZNHIT2	PTK7
				ZP3	PTN
				ZSWIM1	PTPLA
				ZSWIM3	PTPN12
				ZXDB	PTPN13
					PTPN21
					PTTG1IP
					PUM1
					PVRL4
					PXN
					PYCARD
					PYCR1
					PYGL
					QSOX1
					RAB11A
					RAB11FIP4
					RAB15
					RAB25
					RAB34
					RAB3GAP1
					RAB3GAP2
					RAB3IP
					RAD54B
					RADIL
					RALGAPB
					RALGPS1
					RALGPS2
					RAP2C
					RASA1
					RASGRP1
					RASGRP3
					RASL11B
					RBM12
					RBM15B
					RBM25
					RBM28
					RBM35A
					RBM9
					RBMS1

					RBMS2
					RBMS2P
					RBMX
					RBP5
					RCAN2
					RCN1
					RCOR2
					RDH10
					REEP1
					RERE
					RFWD2
					RFX7
					RGS4
					RHOBTB1
					RHOBTB2
					RHOBTB3
					RICH2
					RICS
					RILPL1
					RIMKLB
					RIMS2
					RIN2
					RIOK3
					RIT1
					RNASEN
					RNF128
					RNF38
					RNF44
					RNF5P1
					RNU105A
					ROD1
					ROR1
					ROR2
					RPAP1
					RPESP
					RPL32
					RPL37
					RPL39L
					RPLP0
					RPS6KA5
					RPUSD2
					RRAGB
					RTN2
					RTTN
					RUNX1
					RUNX1T1
					S100A11
					S100A13
					S100A3
					S100A6
					S1PR3
					SAE1
					SAPS3
					SARDH
					SBK1
					SCAMP1

					SCAMP3
					SCAND3
					SCD
					SCD5
					SCGB3A2
					SCN2B
					SCNN1A
					SCRN1
					SCUBE3
					SDHA
					SDK2
					SEC11A
					SEC14L1
					SEC22A
					SEC31A
					SECISBP2L
					SELV
					SEMA3A
					SEMA5A
					SEMA6A
					SEPT6
					SEPT9
					SEPW1
					SERPINC1
					SERPINE2
					SERPINF1
					SERTAD4
					SETBP1
					SETD3
					SEZ6L2
					SF3B1
					SFRP1
					SFRP2
					SFRS14
					SFRS18
					SFRS9
					SFXN4
					SGCB
					SGK
					SGK1
					SGPL1
					SH3GL3
					SH3KBP1
					SH3PXD2B
					SH3TC1
					SHISA5
					SHROOM2
					SIGLEC6
					SILV
					SIPA1L2
					SLC12A2
					SLC16A1
					SLC16A3
					SLC16A5
					SLC1A3
					SLC1A6

					SLC22A5
					SLC23A1
					SLC25A14
					SLC25A18
					SLC25A20
					SLC25A25
					SLC25A36
					SLC25A38
					SLC27A3
					SLC2A12
					SLC2A14
					SLC2A3
					SLC2A5
					SLC35D2
					SLC35E1
					SLC35F1
					SLC35F2
					SLC38A1
					SLC38A2
					SLC38A9
					SLC39A10
					SLC39A14
					SLC43A2
					SLC4A7
					SLC5A6
					SLC6A6
					SLC7A11
					SLC7A2
					SLC7A3
					SLC7A6
					SLC7A9
					SLCO1A2
					SLCO2A1
					SLCO2B1
					SLIT2
					SLIT3
					SLN
					SMAD3
					SMAD4
					SMAD5
					SMAD7
					SMAP1
					SMAP2
					SMARCD1
					SMOC2
					SMS
					SMTNL2
					SMYD4
					SNHG8
					SNORA10
					SNORA8
					SNORD123
					SNTB2
					SNX16
					SNX2
					SNX24

					SOCS1
					SORBS1
					SORCS1
					SORL1
					SORT1
					SOX11
					SOX15
					SOX17
					SOX3
					SP110
					SP3
					SP5
					SP6
					SPARCL1
					SPATA17
					SPATA18
					SPATA7
					SPATS2
					SPINT1
					SPNS1
					SPNS2
					SPRYD4
					SPTAN1
					SPTLC1
					SRC
					SRP68
					SSSCA1
					SSU72
					ST6GAL1
					ST6GALNAC2
					ST6GALNAC3
					STARD13
					STARD8
					STC1
					STEAP3
					STIP1
					STON1
					STOX2
					STRA6
					STRAP
					STRC
					STRN4
					STS-1
					STX6
					STX8
					STYXL1
					SULF2
					SULT1A1
					SULT1A2
					SUPT3H
					SUSD2
					SUV420H1
					SV2A
					SVEP1
					SVIL
					SYDE1

					SYNE2
					SYNJ2
					TAC3
					TACSTD2
					TAF4
					TAF6L
					TAF9L
					TAGLN
					TANC1
					TANC2
					TAOK1
					TARDBP
					TAX1BP1
					TBC1D14
					TBCB
					TBCE
					TBKBP1
					TBX3
					TC2N
					TCEA3
					TCEAL2
					TCF2
					TCF4
					TDG
					TEAD2
					TEAD3
					TEAD4
					TET1
					TF
					TFAP2A
					TFAP2B
					TFAP2C
					TFIP11
					TGFBR2
					TGM1
					THPO
					THSD3
					TIAM2
					TICAM2
					TIGA1
					TIGD7
					TINAGL1
					TKT
					TLE3
					TLE4
					TLK2
					TLN2
					TM7SF3
					TMC7
					TMED3
					TMEFF1
					TMEM125
					TMEM128
					TMEM132A
					TMEM144
					TMEM150A

					TMEM154
					TMEM178
					TMEM184B
					TMEM184C
					TMEM195
					TMEM231
					TMEM233
					TMEM30B
					TMEM41B
					TMEM54
					TMEM68
					TMEM77
					TMEM87A
					TMEM88
					TMTC1
					TMX4
					TNC
					TNFRSF19
					TNFRSF1A
					TNFSF10
					TNFSF12
					TNNT2
					TNPO1
					TOMM34
					TP53BP1
					TP53TG1
					TP63
					TP73L
					TPM1
					TPM4
					TRAK1
					TRH
					TRIL
					TRIM17
					TRIM37
					TRIM6
					TRIM71
					TRIM9
					TRIML2
					TRIP10
					TRPC1
					TRPV6
					TRRAP
					TSPAN13
					TSPAN3
					TST
					TTC17
					TTC23
					TTLL1
					TTLL5
					TTR
					TTYH3
					TUB
					TUBB2B
					TUBB3
					TUBB4

					TUBB8
					TUFT1
					TULP3
					TULP4
					TUSC3
					TWIST1
					TXLNA
					TXNDC3
					TXNDC9
					TXNRD2
					TYRO3
					UBB
					UBE2E1
					UBE2L6
					UBE2V1
					UBE2Z
					UBE3A
					UBQLN4
					UBR5
					UBXN4
					UCA1
					UCHL1
					UCHL5
					UCRC
					UGT2B10
					UGT3A2
					ULK1
					UNC5B
					UNKL
					UPF2
					UPF3B
					UPK2
					USP13
					USP36
					USP46
					USP5
					UVRAG
					VAMP1
					VANGL2
					VARS2
					VAT1
					VAV3
					VCAN
					VCX
					VEZF1
					VGF
					VGLL1
					VGLL4
					VIL2
					VPS24
					VPS28
					VPS33B
					VPS35
					VPS37C
					VTCN1
					VTN

					VWA5A
					WARS
					WASF3
					WBP5
					WDFY2
					WDR27
					WDR4
					WDR42A
					WDR54
					WDR6
					WDR68
					WDR72
					WDR82
					WDR86
					WDSUB1
					WDYHV1
					WEE1
					WNK1
					WNT11
					WNT4
					WNT5B
					WNT6
					WSB1
					WWC3
					XAGE2B
					XKR6
					XKRX
					XPC
					XPNPEP1
					XPR1
					XYLT2
					YAP1
					YEATS2
					YPEL1
					YTHDC1
					YWHAZ
					ZBTB20
					ZBTB34
					ZBTB4
					ZC3H12C
					ZC3H8
					ZCCHC11
					ZDHHC13
					ZDHHC18
					ZDHHC4
					ZDHHC7
					ZDHHC8
					ZFC3H1
					ZFHX3
					ZFP36L1
					ZFP37
					ZFP82
					ZIC2
					ZMIZ1
					ZMYM2
					ZNF10

					ZNF136
					ZNF138
					ZNF185
					ZNF204
					ZNF207
					ZNF215
					ZNF217
					ZNF219
					ZNF248
					ZNF26
					ZNF271
					ZNF275
					ZNF280D
					ZNF286A
					ZNF286C
					ZNF322A
					ZNF331
					ZNF334
					ZNF337
					ZNF384
					ZNF415
					ZNF423
					ZNF426
					ZNF430
					ZNF431
					ZNF433
					ZNF454
					ZNF462
					ZNF488
					ZNF511
					ZNF518B
					ZNF525
					ZNF552
					ZNF558
					ZNF564
					ZNF567
					ZNF573
					ZNF584
					ZNF589
					ZNF606
					ZNF608
					ZNF610
					ZNF627
					ZNF641
					ZNF680
					ZNF701
					ZNF702P
					ZNF721
					ZNF74
					ZNF750
					ZNF761
					ZNF763
					ZNF783
					ZNF813
					ZNF816A
					ZNF827

					ZNF83
					ZNF845
					ZNF91
					ZNF93
					ZNHIT1
					ZNRF2
					ZSCAN18

fetal_vs_phh_ anova AND hlc_vs_fetal_ anova AND hlc_vs_phh_ anova
A1CF
AARS2
AASS
ABCA6
ABCB4
ABCB7
ABCC4
ABCC5
ABCC6
ABHD2
ABL1
ABR
ABTB1
ACAT1
ACHE
ACMSD
ACOT12
ACOT7
ACOX2
ACSL1
ACSM2A
ACSM2B
ACVR2A
ACY1
ADAMTS9
ADD3
ADM
ADNP
ADRB2
AGPAT2
AGT
AGTR1
AGXT
AHNAK
AIF1
AK1
AK2
AKAP12
AKAP13
AKIRIN1
AKR1B15
ALAD
ALCAM
ALDH18A1
ALDH1A1
ALDH1L1
ALDH2

ALDH3A2
ALDH4A1
ALDH7A1
ALDH8A1
ALDH9A1
ALDOA
ALDOB
AMMECR1
ANG
ANGPT1
ANGPTL4
ANKRA2
ANKRD1
ANKRD57
ANP32B
ANXA2
AP1S1
AP2A1
AP3B1
AP3D1
APOB
APOC2
APOC3
APOH
APPBP2
AQP11
ARAP3
ARG1
ARHGAP17
ARHGAP23
ARHGAP24
ARHGDIA
ARHGEF16
ARHGEF5
ARID4A
ARL4C
ARMCX2
ART4
ASB1
ASB8
ASB9
ASF1B
ASGR1
ASGR2
ASH2L
ASL
ASPM
ATF5
ATF6B
ATG4A
ATG7
ATM
ATP2B2
ATP5J
ATP6V0D1
ATP6V0E1

ATP6V0E2
ATP6V1A
ATP6V1E2
ATPAF1
ATRN
ATXN2
AURKB
AVPI1
AXL
AXUD1
B2M
B3GALNT1
BAT3
BBX
BCHE
BCKDHA
BCL2L2
BEND5
BEX2
BHLHB2
BHMT2
BID
BIRC6
BMP1
BMP4
BOLA2
BPNT1
BPTF
BRMS1
BTBD7
C10orf137
C10orf47
C10orf6
C12orf32
C12orf4
C12orf41
C12orf48
C12orf51
C12orf57
C12orf76
C13orf15
C13orf7
C14orf131
C14orf174
C14orf68
C14orf93
C15orf21
C15orf42
C16orf35
C17orf41
C19orf64
C19orf66
C1orf128
C1orf135
C1orf54
C1QTNF6

C20orf160
C20orf199
C20orf56
C21orf33
C21orf57
C21orf58
C22orf13
C22orf28
C22orf30
C2orf56
C3
C3orf21
C3orf52
C3orf70
C5orf30
C5orf32
C6orf115
C6orf173
C6orf48
C6orf66
C7orf55
C8orf33
C9orf100
C9orf103
C9orf21
C9orf45
C9orf78
CA14
CALML4
CAP1
CAPRIN1
CARD8
CARS
CASP2
CASP4
CAST
CBR1
CCBL1
CCDC28B
CCDC34
CCDC53
CCDC77
CCL14
CCNA2
CCND1
CCND3
CCNDBP1
CCRN4L
CCT8
CD14
CD151
CD1D
CD248
CD81
CD93
CDC2

CDC20
CDC23
CDC25A
CDC42EP1
CDC45L
CDCA3
CDK10
CDK2
CDK6
CDKN1A
CDKN3
CDRT4
CDT1
CEBPB
CECR1
CENPA
CENPE
CENPF
CENPV
CEP135
CEP152
CERK
CFB
CFH
CFHR1
CFHR2
CFI
CGN
CHAF1B
CHD7
CHD9
CHIC2
CHN2
CHPT1
CHST7
CIB1
CIDEB
CKAP2L
CKAP5
CLCC1
CLDN7
CLDND1
CLIP1
CLNS1A
CLSTN1
CMAS
CMIP
CMPK1
CMTM3
CMTM7
CNIH
CNOT6
CNOT8
CNTNAP2
COBRA1
COL1A2

COL4A1
COL4A3BP
COL5A2
COMT
COPG2
COPS7B
COQ9
COX5B
COX7C
CP110
CPB2
CPN1
CPSF4
CPVL
CRB3
CREB3L2
CRK
CRLF3
CSE1L
CSF1R
CSF3R
CSNK1E
CSRP2
CTAGE6
CTSE
CXCL14
CYB561
CYB5A
CYBA
CYCSL1
CYP2J2
CYP39A1
CYP3A5
CYP4A11
CYP4F12
CYP4F3
CYP4V2
CYTSB
DACT3
DBI
DCAF7
DCP2
DDA1
DDB1
DDC
DDX11
DDX23
DDX54
DECR1
DECR2
DEDD2
DENND2A
DHDDS
DHRS4
DHX32
DICER1

DIO1
DLGAP5
DMAP1
DMC1
DNAJC12
DNASE1
DNASE2
DNMT1
DOK4
DPF2
DPM2
DPYD
DSP
DSTN
DUS3L
DUSP14
DUSP23
DUSP28
DUSP6
DVL3
DYNC1H1
DYNLL1
EARS2
EBP
EBPL
ECH1
ECHDC2
EEF1A2
EEF1D
EGFLAM
EHD4
EHHADH
EIF2AK1
EIF3D
EIF3L
EIF4A1
EIF5A
ELF3
ELL2
ELOVL1
ELOVL2
ELP2
EMILIN2
EML1
ENPP5
EP400
EPB41L3
EPHB6
ERI1
ERICH1
ERP29
ERRFI1
ESAM
ETFDH
ETS1
EVC2

EXO1
EYA2
F11
F11R
F5
F7
F9
FAAH2
FABP1
FABP7
FADS1
FAM111A
FAM117B
FAM125B
FAM129A
FAM160B2
FAM176A
FAM177A1
FAM39DP
FAM46A
FAM82A2
FASTKD1
FBXL20
FBXO11
FBXO18
FBXO32
FBXO38
FBXO5
FBXW5
FEN1
FES
FEZ1
FEZ2
FGD5
FGFR3
FKBP1A
FLJ14213
FLJ21986
FOXA1
FOXA3
FOXJ2
FOXM1
FSCN1
FTCD
FTHL12
FTHL8
FXYD1
FXYD2
G6PC
GAL3ST1
GALK1
GAMT
GAS1
GBA3
GBAS
GBGT1

GBP2
GCA
GCH1
GCHFR
GCLC
GCNT2
GGA3
GGCX
GHDC
GINS3
GLYAT
GLYCTK
GNA13
GOT1
GPER
GPM6B
GPR128
GPR175
GPR177
GPR37
GPR56
GPRASP2
GPRC5C
GPX1
GSDMD
GSR
GSTA1
GSTA5
GSTK1
GSTT1
GTF2E2
GTF2IRD2B
GTF3C2
GUSB
H19
H2AFY
H3F3A
HABP2
HACL1
HADH
HADH2
HAGH
HAO1
HCFC1
HCN4
HDAC2
HDDC2
HEG1
HERC5
HES4
HEXB
HFE2
HGFAC
HGS
HIST1H4C
HIST2H2AA3

HIST2H2AB
HJURP
HLA-H
HMGB2
HMGXB4
HMMR
HNRNPAB
HOXB2
HP
HPD
HPN
HPR
HPS1
HPX
HRASLS3
HRSP12
HSD17B10
HSD17B11
HSZFP36
HTRA1
HTT
HULC
HYOU1
IAH1
IARS2
ICAM2
ID3
IER3
IFI35
IFI6
IFITM1
IFITM2
IFITM3
IFRD1
IFRD2
IFT88
IGF2BP2
IGF2BP3
IGFBP7
IGSF1
IKBKG
IL20RB
IMPDH2
INTS4
IP6K1
IQCK
IRAK2
IRF8
IRX3
ISCA1
ISG20
ISOC2
ITIH1
ITIH2
ITIH4
ITM2A

ITM2C
ITPR1
ITPRIPL2
IVD
IYD
JAG1
JAM3
JARID2
JMJD8
KANK3
KANK4
KAT2B
KBTBD11
KCNS3
KCTD14
KCTD5
KEAP1
KHK
KIAA0101
KIAA0319L
KIAA0355
KIAA0528
KIAA0922
KIAA1026
KIAA1712
KIAA1797
KIAA1881
KIAA2013
KIF12
KIF15
KIF1B
KIF20A
KIF20B
KIF2C
KIFC1
KIT
KLHL36
KLKB1
KNG1
KNTC1
KPNB1
KRT18
KRT18P13
KRT18P28
KRT222
KYNU
LACTB2
LAMC2
LANCL1
LARP6
LARS
LBH
LCMT1
LEAP-2
LGALS2
LIN37

LIPG
LITAF
LMNB2
LMO4
LOC100128266
LOC100128410
LOC100129028
LOC100129552
LOC100129905
LOC100130506
LOC100130707
LOC100131205
LOC100131735
LOC100132287
LOC100132394
LOC100132728
LOC100132795
LOC100133923
LOC100134134
LOC146177
LOC148915
LOC149501
LOC205251
LOC338799
LOC339970
LOC340274
LOC340598
LOC387763
LOC388514
LOC389599
LOC390530
LOC391075
LOC400027
LOC440145
LOC441377
LOC441506
LOC550643
LOC642252
LOC642502
LOC642567
LOC644037
LOC644191
LOC644743
LOC645015
LOC645166
LOC645173
LOC645515
LOC645715
LOC646294
LOC646688
LOC646723
LOC647346
LOC647786
LOC648771
LOC650646
LOC653110

LOC653381
LOC653498
LOC653635
LOC653778
LOC653888
LOC654096
LOC654103
LOC727866
LOC728037
LOC728453
LOC728553
LOC728672
LOC729208
LOC729217
LOC729887
LOC730202
LOC730246
LOC730323
LOC731950
LOC81691
LOXL4
LPHN2
LPP
LRFN3
LRIG1
LRP3
LRRC16
LRRC28
LRRFIP2
LTBR
LUZP1
LYSMD4
MACF1
MAD1L1
MAD2L1BP
MAGED1
MAGED2
MALL
MALT1
MAP2K1
MAP2K5
MAPBPIP
MAPKAP1
MARCKSL1
MARVELD3
MASP1
MASP2
MBNL2
MCM10
MCM2
MCM4
MCM6
MCM7
MDC1
MDK
MED23

MED24
MED25
METTL1
METTL7A
METTL7B
MFSD6
MGC4677
MGC57346
MGEA5
MICALL2
MID1IP1
MID2
MIDN
MLF2
MMS19
MMS19L
MOBKL2C
MPST
MPV17
MPV17L2
MRPL11
MRPL17
MRPL20
MRPL23
MRPL36
MRPL40
MRPS27
MSRA
MSX1
MT1H
MTHFD2
MTHFS
MTL5
MTO1
MUC1
MYADM
MYC
MYH10
MYH9
MYO6
MYO9A
MYPOP
NAAA
NARF
NAT15
NCALD
NCAPD2
NDC80
NDRG2
NDUFAF1
NDUFAF3
NEDD9
NELF
NEO1
NFE2L1
NFIB

NFIL3
NGEF
NINJ1
NINJ2
NIP7
NIPBL
NIPSNAP1
NMRAL1
NODAL
NONO
NOP16
NOP2
NR0B2
NR1H3
NR1I3
NR2F2
NRP1
NSL1
NT5DC3
NT5E
NT5M
NTHL1
NTN1
NUAK2
NUCB1
NUCKS1
NUDT15
NUP160
NUP205
NUP43
NUP93
NUPR1
NUSAP1
NUTF2
OAS1
OAS2
OBFC2A
OCRL
ODF2
OR9A4
ORAOV1
ORC2L
ORC3L
OSBPL1A
OSBPL9
OSCP1
OXCT2
OXER1
OXSR1
P2RY2
P4HB
P4HTM
P76
P8
PABPC1
PABPC4

PACSIN2
PALM
PAMR1
PAPSS1
PAQR8
PAQR9
PARL
PARP9
PARVA
PAWR
PBLD
PCBD1
PCBP2
PCDH24
PCGF5
PCK2
PCNA
PCYT2
PDE12
PDE9A
PDGFRB
PDIA3P
PDXDC1
PDZD8
PEBP1
PEG10
PEPD
PEX11G
PEX16
PFAS
PFKP
PHF21A
PHIP
PHKB
PHLDA1
PIK3R2
PIM2
PINK1
PIP5K2A
PJA1
PKMYT1
PKP2
PLA2G12B
PLAA
PLAG1
PLCXD1
PLD1
PLD6
PLEKHF1
PLEKHG4
PLGLB1
PMVK
POGK
POLR2L
POLR3A
POLR3C

POLR3GL
POLR3H
PON1
PON2
PPDPF
PPIC
PPIH
PPP1R16A
PPP1R1A
PPP2R2A
PPP2R3C
PQBP1
PRAP1
PRC1
PRDX5
PRIC285
PRICKLE2
PRKAB1
PRKAG2
PRKX
PROC
PROS1
PRR4
PSCD2
PSMB10
PSMB7
PSMD12
PSRC1
PTBP2
PTGES2
PTGFRN
PTH1R
PTK2
PTOV1
PTPRF
PTPRH
PTPRM
PTRF
PTTG1
PTTG3P
PVRL2
PWWP2B
PXMP2
QARS
QDPR
QPRT
RAB11FIP1
RAB11FIP5
RAB13
RAB23
RAB27A
RAB2B
RAB31
RAB32
RAB9A
RABGGTB

RABL2B
RACGAP1
RAP1GAP
RAPGEF2
RARA
RASSF1
RAVER2
RBKS
RBM12B
RBM4
RBP4
RBPMS
RCAN1
RCOR3
RDH5
REEP6
REPS2
RETSAT
RFWD3
RGN
RGS12
RHBDD1
RHOC
RHOT1
RHOU
RIC8A
RIPK2
RIPK4
RIPK5
RMI1
RNASE4
RND3
RNF126
RNF135
RNF144
RNF144A
RNF150
RNF185
RNMTL1
RNPC3
ROBO3
ROMO1
RPL15
RPL23
RPL36AL
RPL41
RPP21
RPP38
RPRC1
RPS2
RPS26P11
RPS4X
RPS6KB2
RRAS2
RREB1
RRP15

RSPO3
RTKN
RTN4
RTP2
RYK
SAA4
SACM1L
SALL2
SAMD9
SAMM50
SAT1
SCAND1
SCARA3
SCCPDH
SCG5
SCYL1
SDC4
SDHB
SDSL
SEC24C
SELENBP1
SEMA4F
SEMA6B
SEPN1
SEPT11
SEPT2
SEPX1
SERF2
SERPINA1
SERPINA11
SERPINA3
SERPINA4
SERPINA6
SERPINB1
SESN1
SFRS4
SGCE
SH3BP4
SHRM
SIGLEC14
SIK1
SIK3
SIPA1
SKA1
SKA2
SKP2
SLC11A2
SLC15A3
SLC15A4
SLC16A4
SLC16A9
SLC20A1
SLC25A15
SLC25A43
SLC27A2
SLC2A1

SLC37A3
SLC39A5
SLC39A8
SLC3A2
SLC43A3
SLC44A1
SLC44A2
SLC7A5
SLC7A7
SMAGP
SMARCA1
SMARCAD1
SMARCB1
SMARCC1
SMC3
SMURF1
SNAP29
SNHG6
SNRNP35
SNRPD2
SNUPN
SNX27
SOD1
SON
SORBS2
SORD
SOX4
SOX9
SPARC
SPC24
SPIN4
SPINT2
SPRY1
SPRY2
SPRYD3
SQRDL
SS18
SSR4
ST7
STBD1
STC2
STK3
STK40
STMN3
STOM
STRADA
STT3A
STX3
SUCLG1
SUMO3
SUPT16H
SUSD1
SWAP70
SYNJ1
SYTL4
TACC1

TACC2
TACSTD1
TBC1D9B
TBPL1
TDO2
TEK
TERF2IP
TFB2M
TFPI
TGIF1
TH1L
THSD4
TIMP2
TJP1
TJP2
TM4SF1
TM4SF18
TM4SF4
TM4SF5
TMEM140
TMEM166
TMEM176A
TMEM176B
TMEM181
TMEM189-UBE2V1
TMEM194A
TMEM216
TMEM217
TMEM49
TMEM51
TMEM59
TMEM86B
TMOD1
TMPRSS2
TMPRSS6
TNFAIP3
TNFRSF10B
TNFRSF12A
TNFRSF14
TNFRSF21
TNS3
TOM1L2
TOP2B
TP53AP1
TPM2
TPMT
TPR
TPST2
TRAF3IP2
TRAF6
TRIM15
TRIM26
TRIM33
TRIOBP
TRIP6
TRO

TSC22D2
TSPAN17
TSPO
TSPYL1
TSPYL3
TSPYL5
TSSC4
TTC3
TUBA1C
TUBD1
TUBG1
TXN
TXNDC12
TXNRD1
TYSND1
U2AF1
UAP1L1
UBAP2L
UBE2C
UBE2D4
UBE2T
UBL4A
UCHL5IP
UGP2
UGT2B4
UGT2B7
UHRF1
ULK4
UNG
UPB1
UPP1
UQCC
UQCRQ
UROD
USE1
USMG5
USP15
USP21
VAMP5
VBP1
VCL
VDAC3
VIPR1
VLDLR
VNN3
VOPP1
VPS37A
VPS8
VRK1
VSNL1
WBP11
WDR1
WDR20
WDR23
WDR51A
WNT5A

XBP1
XPO1
XPO4
XRN1
YARS
YWHAG
ZCCHC14
ZER1
ZFAND2B
ZFAND5
ZFP106
ZFYVE26
ZMAT3
ZMYND8
ZNF137
ZNF148
ZNF165
ZNF211
ZNF318
ZNF320
ZNF33B
ZNF341
ZNF428
ZNF439
ZNF503
ZNF514
ZNF518A
ZNF532
ZNF585A
ZNF609
ZNF621
ZNF626
ZNF671
ZNF69
ZNF7
ZNF784
ZNF841
ZSCAN2
ZSWIM4

Supplementary Table S5: Gene list related to Hippo pathway.

Term	%	PValue	Genes
GO:0005667~transcription factor complex	20,51	1,63E-06	SMAD7, TEAD4, SMAD4, SMAD3, LEF1, TEAD2, TEAD3, YAP1
GO:0044451~nucleoplasm part	23,08	1,24E-04	SMAD7, TEAD4, SMAD4, SMAD3, LEF1, TEAD2, TEAD3, YAP1, PPP1CC
GO:0045177~apical part of cell	15,38	1,35E-04	PRKCZ, INADL, PRKCI, CDH1, FZD3, FZD6
GO:0005911~cell-cell junction	15,38	1,79E-04	PRKCZ, INADL, SMAD7, AMOT, CDH1, PARD6G
GO:0016327~apicolateral plasma membrane	12,82	1,85E-04	PRKCZ, INADL, AMOT, PARD6G, FZD6
GO:0044459~plasma membrane part	41,03	4,91E-04	PRKCZ, INADL, SMAD7, TGFBR2, PRKCI, CDH1, FZD3, ITGB2, FZD2, CTNNA1, FZD6, FRMD6, AMOT, PARD6G, DLG2, BMPR1A
GO:0030054~cell junction	20,51	5,17E-04	PRKCZ, INADL, SMAD7, AMOT, CDH1, PARD6G, CTNNA1, DLG2
GO:0005923~tight junction	10,26	0,0011	PRKCZ, INADL, AMOT, PARD6G
GO:0070160~occluding junction	10,26	0,0011	PRKCZ, INADL, AMOT, PARD6G
GO:0005654~nucleoplasm	23,08	0,0027	SMAD7, TEAD4, SMAD4, SMAD3, LEF1, TEAD2, TEAD3, YAP1, PPP1CC
GO:0043296~apical junction complex	10,26	0,0027	PRKCZ, INADL, AMOT, PARD6G
GO:0015629~actin cytoskeleton	12,82	0,0067	PRKCZ, AMOT, CDH1, CTNNA1, LLGL1
GO:0005856~cytoskeleton	25,64	0,0124	PRKCZ, PPP2R1A, YWHAZ, FRMD6, AMOT, CDH1, CTNNA1, DLG2, LATS2, LLGL1
GO:0016342~catenin complex	5,13	0,0140	SMAD7, CDH1
GO:0031012~extracellular matrix	12,82	0,0156	WNT4, WNT5B, WNT11, BMP7, WNT6
GO:0005886~plasma membrane	46,15	0,0188	PRKCZ, INADL, SMAD7, TGFBR2, PRKCI, SMAD3, CDH1, ITGB2, FZD3, FZD2, CTNNA1, FZD7, FZD6, FRMD6, AMOT, PARD6G, DLG2, BMPR1A
GO:0044448~cell cortex part	7,69	0,0228	PRKCZ, PRKCI, LLGL1
GO:0043235~receptor complex	7,69	0,0421	TGFBR2, SMAD3, ITGB2
GO:0031981~nuclear lumen	23,08	0,0451	SMAD7, TEAD4, SMAD4, SMAD3, LEF1, TEAD2, TEAD3, YAP1, PPP1CC
GO:0005578~proteinaceous extracellular matrix	10,26	0,0605	WNT4, WNT5B, WNT11, WNT6
GO:0045121~membrane raft	7,69	0,0611	PRKCZ, TGFBR2, BMPR1A
GO:0005938~cell cortex	7,69	0,0634	PRKCZ, PRKCI, LLGL1
GO:0005912~adherens junction	7,69	0,0704	SMAD7, CDH1, CTNNA1
GO:0070161~anchoring junction	7,69	0,0842	SMAD7, CDH1, CTNNA1
GO:0005913~cell-cell adherens junction	5,13	0,0941	SMAD7, CDH1

Supplementary Table S6: Gene lists of the venn diagram Figure 6A.

he_expr	hlc_expr	he_expr AND hlc_expr	de_expr	he_expr AND de_expr	hlc_expr AND de_expr	he_expr AND hlc_expr AND de_expr
ABCC6P2	A4GNT	A1CF	ABCG4	ABCC1	AADACL4	A2LD1
ADAM12	AADAC	ABCC2	ABI3	ACCN2	ABAT	A2ML1
ADC	ABCA5	ACADL	ACADSB	ADAM23	ABCA11	A4GALT
ADPRHL1	ABCA8	ACCN3	ACAP1	ADAMTSL3	ABCA2	AAAS
AMBN	ABCA9	ADAT2	ACPT	ADD2	ABCA4	AACS
APBB2	ABCB4	ADRA1B	ACSBG1	ADRB2	ABCC6	AACSL
ARFIP2	ABCC6P1	AFAP1L1	ACSM4	AGR3	ABCC9	AADACL1
ARL6	ACRV1	AFF2	ACTN3	AMPH	ABCD1	AADAT
BCL2A1	ACSL5	AGT	ACVRL1	ANAPC7	ABCG2	AAGAB
BOLL	ACSS1	AKR1B10	ADAMTS8	ANK2	ACE2	AAK1
C10orf112	ADAMTS2	AKR1B15	ADRA2C	AOAH	ACTG2	AAMP
C11orf65	ADAMTS20	ALDH1L1	AGER	APBB1	ADAM2	AARS
C13orf33	ADAMTSL1	ALDH3B2	AGXT2L1	APCDD1	ADAM8	AARS2
C14orf165	ADCY4	ALG10	ALDOB	ARRB2	ADAMTS7	AARSD1
C19orf57	ADH1A	ALG2	ANK1	ASB9	ADAMTSL5	AASDH
C1orf103	ADRA1A	AMAC1L2	ANKRD19	ASCL2	AFF3	AASDHPPT
C1orf182	AFM	AMDHD1	ANKRD55	ASPHD1	AGPAT9	AASS
C2	AGMAT	AMY2A	ANKRD58	ATOH7	AKAP2	AATF
C21orf34	AGPAT3	ANKRD43	ANO4	ATP1B2	ALDH1A2	AATK
C3orf57	AHSG	ANXA4	ANO7	ATP6V0D2	ALPPL2	ABCA1
C4orf36	AIFM2	ANXA8	AOX1	B3GAT1	ALS2CR16	ABCA3
C4orf47	ALMS1P	ANXA8L1	ARHGEF4	B3GNT4	AMH	ABCA7
C5orf38	ALOX15	ANXA9	ARNTL2	BARX1	ANAPC2	ABCB10
C5orf43	ALOX5AP	APOB	ARTN	BCAS3	ANGPT2	ABCB6
C7orf46	ALPI	APOC3	ARVCF	BCL10	ANKRD23	ABCB7
C7orf51	AMY2B	APOL2	ATOH8	BCL6B	ANKRD36	ABCB9
C8orf79	ANKS4B	AQP3	ATP1A2	BEST4	ANKRD41	ABCC10
CACNA1S	ANP32E	ARHGAP18	ATP2B3	BFSP1	ANO1	ABCC3
CALCA	ANXA8L2	ARHGAP8	ATP8B3	BOLA2B	APCDD1L	ABCC4
CASC1	APLF	ARNTL	B3GALT5	BSPH1	APOBEC3B	ABCC5
CBLN1	AQP1	ARRDC4	BAI1	BTBD11	APOF	ABCD3
CD69	AQP10	ASCL5	BAZ2A	BZW1	AQP7P1	ABCE1
CDYL2	ARAP2	ATP10B	BDKRB2	C12orf28	AQP7P2	ABCF1
CHRDL2	ARHGAP20	ATP2A3	BHLHE22	C12orf34	ARHGAP11B	ABCF2
CLGN	ARHGAP29	ATP2C2	BRDT	C12orf56	ARVP6125	ABCF3
COL21A1	ARHGAP30	ATP6V1C2	BREA2	C14orf101	ASAP1IT1	ABCG1
CPSF4L	ARHGAP6	ATP7A	BTBD9	C14orf11	ATF6B	ABHD10
CR2	ARPM1	ATP8B1	C10orf85	C14orf115	ATG2B	ABHD11
CRYBB2	ARRDC1	AURKAPS1	C10orf96	C14orf65	ATP13A3	ABHD12
CUZD1	ART4	BACH1	C12orf36	C15orf34	ATP8B4	ABHD12B
CXorf18	ASGR2	BCAM	C12orf73	C16orf45	ATRX	ABHD14A
CXorf48	BAI2	BEND6	C14orf121	C17orf93	B3GNT3	ABHD15
CXorf56	BCAR4	C10orf10	C14orf21	C19orf4	B3GNT5	ABHD2
DAZ2	BCMO1	C10orf65	C14orf72	C1orf51	BAALC	ABHD3

DAZ4	BNC1	C10orf73	C16orf11	C20orf54	BATF3	ABHD4
DHODH	BRUNOL6	C11orf36	C16orf69	C21orf63	BCAN	ABHD5
DKFZp547K05	C10orf128	C11orf52	C17orf67	C2CD4C	BDP1	ABHD6
DKFZP564J10	C10orf41	C11orf71	C17orf76	C3orf54	BOC	ABHD7
DLX6	C10orf54	C17orf75	C19orf51	C3orf71	BRD4	ABHD8
DLX6AS	C12orf27	C18orf19	C1orf222	C5orf45	BTK	ABHD9
DNAI1	C13orf31	C18orf2	C1orf94	C6orf126	BTN3A2	ABI1
DNTT	C15orf48	C19orf69	C1QL1	C8orf13	C10orf39	ABI2
DYNC1LI1	C16orf54	C1orf105	C1QL4	C9orf125	C14orf176	ABL1
EBF3	C16orf55	C1orf161	C1QTNF4	C9orf129	C15orf59	ABL2
EDIL3	C17orf74	C1orf177	C20orf112	C9orf24	C17orf108	ABLIM1
EEFSEC	C17orf82	C1orf189	C20orf62	CA12	C19orf66	ABR
EMR4	C19orf21	C1orf213	C2CD4B	CADPS	C19orf71	ABT1
EN2	C1orf170	C1orf64	C2orf48	CAMK1	C1orf95	ABTB1
EPS8L2	C1orf188	C20orf151	C2orf78	CAMK1D	C1QA	ABTB2
EPS8L3	C1orf210	C2orf67	C2orf80	CAMKV	C1QTNF5	ACAA1
EREG	C1orf215	C3orf60	C3orf32	CAPN9	C21orf128	ACAA2
ERVK6	C1orf229	C4orf16	C3orf35	CCDC4	C4orf38	ACACA
EXOG	C1orf81	C4orf19	C3orf51	CCDC88A	C4orf39	ACACB
FA2H	C1QTNF1	C4orf32	C4orf11	CCL26	C6orf204	ACAD10
FAM153A	C21orf119	C6orf100	C5orf20	CD2	C6orf25	ACAD11
FAM155B	C21orf49	C8orf47	C7orf57	CD200	C6orf81	ACAD8
FAM178B	C2orf54	C8orf51	C8orf15	CD3EAP	C8orf16	ACAD9
FAM187B	C3orf15	C9orf164	C8orf30B	CD7	C8orf44	ACADM
FAM46D	C3orf36	C9orf66	C8orf34	CDA	C9orf3	ACADS
FAM47E	C3orf62	CACNG1	C8orf77	CDH20	CA8	ACADVL
FANK1	C3orf70	CASQ2	C8orf80	CDK5R2	CACNA2D3	ACAP2
FBXO25	C5orf23	CBX8	C8ORFK29	CER1	CALCR	ACAT1
FGFBP1	C5orf27	CCBP2	CA10	CHGA	CAMK1G	ACAT2
FGFR1	C6orf199	CCDC126	CA5BP	CHRNB4	CAPN14	ACBD3
FLJ22447	C6orf208	CCDC57	CACNA1D	CHST4	CCDC147	ACBD4
FLJ23865	C6orf221	CCDC68	CACNA1I	CHST6	CCDC17	ACBD6
FLJ36032	C7orf53	CD163	CADM3	CHST8	CCDC35	ACBD7
FLJ36848	C8orf4	CDK8	CAMK2B	CLEC4D	CCNJ	ACCS
FLJ37512	C9orf62	CEACAM21	CAMKK1	CNIH3	CDH26	ACD
FLJ40330	CA3	CECR4	CAPN10	COL9A1	CDKN2B	ACER2
FLJ44450	CACNG4	CFB	CAPN11	COX11P	CDKN2C	ACER3
FLJ90036	CADM2	CFC1B	CBY3	CPNE4	CEACAM1	ACHE
FOXJ1	CAMTA2	CFH	CCDC141	CPT1C	CGA	ACIN1
FXYD4	CAPN3	CFHR3	CCDC19	CSAG1	CGB1	ACLY
G0S2	CASP4	CHGB	CCDC36	CTSF	CHST1	ACN9
GAGE12H	CCDC146	CHTF8	CCDC74A	CUX2	CKMT2	ACO1
GAS2	CCDC149	CIDEB	CCDC81	DBF4	CLSTN2	ACO2
GCNT6	CCDC153	CIDECP	CCL8	DCX	CNR1	ACOT1
GDAP1L1	CCDC33	CLDN9	CD163L1	DDX25	COL20A1	ACOT11
GDEP	CCDC96	CPEB4	CD164L2	DDX6	CORO6	ACOT2
GNAT1	CCR7	CPN1	CD3G	DEPDC7	CPPED1	ACOT4
GPR112	CD247	CRH	CD48	DGCR5	CRIM1	ACOT7
GPR150	CD302	CROCCL1	CD70	DNAJC11	CRLF1	ACOT8
GSDM1	CD84	CRYBA1	CDC14C	DNAJC12	CROCCL2	ACOT9

GTF2H2	CDGAP	CSGALNACT1	CDH12	DOCK5	CRYBA2	ACOX1
GTSF1	CDH17	CSNK1A1L	CDH4	DOK1	CSDC2	ACOX2
HCG18	CDH5	CTSE	CECR2	DPY19L3	CSF3R	ACOX3
HCK	CDH8	CTSO	CELP	DUSP26	CTAGE5	ACP1
HGD	CDKN2A	CXorf21	CHADL	DZIP1	CTGLF1	ACP2
HGSNAT	CDNF	CYP46A1	CHRNA10	ECAT8	CXCL1	ACP6
HIST1H2AM	CDY1B	DALRD3	CHST11	EDA	CYP2D7P1	ACPL2
HIST1H3G	CEACAM6	DDC	CIT	EFCBP1	DDX26B	ACPP
HIST1H4I	CEND1	DHFR	CLUL1	FABP5L3	DEFB1	ACRC
HIST2H2BF	CEP170	DIRAS2	CMTM1	FAM124A	DEFB116	ACSF2
HYALP1	CGB	DIRAS3	CNN1	FAM133A	DEFB123	ACSL1
ICA1L	CGB5	DMRT2	CNNM1	FAM167A	DENND3	ACSL3
ICF45	CGB7	DMXL2	CNTNAP3	FAM179A	DKFZP434L18	ACSL4
IFI44	CGB8	DNAJB4	CNTNAP3B	FAM59B	DKFZp686E24	ACSM3
IL15RA	CHRD	DNAJC22	COL27A1	FAM80A	DNHL1	ACSS2
INSRR	CHRNA2	DNAJC25-GNG	COL29A1	FANCF	DPY19L2P2	ACSS3
IRF2	CHRNE	DOK5	COL6A6	FAT4	DQX1	ACTA1
ISL2	CIDEA	DOK6	COL8A1	FCN3	DST	ACTA2
JARID1C	CIDEC	DPEP1	CORO1A	FGF12	DZIP3	ACTB
JMJD7	CILP	DPYS	CORO2B	FGF19	EBI3	ACTC1
KCNH2	CLDN18	DUSP27	COX7A1	FLJ10324	ECM1	ACTG1
KERA	CLEC18C	DUSP9	CRYGD	FLJ25404	EGF	ACTL6A
KIAA0692	CLEC1A	DUX4	CSF2	FLJ40125	ELOVL2	ACTN1
KIAA1324	CLEC2B	EDA2R	CSMD2	FLJ44379	EMX1	ACTN4
KIAA1345	CLEC2L	EGFL6	CSPG4	FOXD4	ERP44	ACTR10
KILLIN	CLIC3	EHHADH	CST2	FOXI2	ETV2	ACTR1A
KLHL14	CNNM2	EIF4E1B	CUGBP2	FPR1	EVI1	ACTR1B
KLRC1	CNTN4	ELF5	CXCL11	FRAT1	EZH1	ACTR2
LCE1B	COL15A1	ELMOD1	CXCL6	FRMD5	FAM101A	ACTR3
LNP1	COLEC11	ELP2P	CXCR1	FZD9	FAM183A	ACTR3B
LOC10012788	COX18	EMR2	CXCR4	GABRA5	FAM21A	ACTR5
LOC10012801	CPA4	ENTPD1	CXorf27	GAL3ST3	FAM90A6P	ACTR6
LOC10012815	CPAMD8	EPN3	CYP2A13	GAP43	FAM91A1	ACTR8
LOC10012820	CPLX2	EPO	CYSLTR2	GDPD4	FBLN7	ACTRT1
LOC10012829	CPNE2	ERBB2IP	DACH2	GEMIN7	FCGBP	ACVR1
LOC10012836	CRCT1	ERCC6	DDN	GFRA3	FGD4	ACVR1B
LOC10012852	CREB3L3	ETHE1	DDX20	GGT2	FLCN	ACVR2A
LOC10012859	CRISP2	EXOC8	DEFB125	GK	FLI1	ACVR2B
LOC10012888	CRTC1	F2	DGKB	GLI1	FLJ10088	ACY1
LOC10012905	CRYAB	F7	DHRS9	GLI2	FLJ10246	ACYP1
LOC10012911	CSF1R	FALZ	DIRAS1	GLIPR1L1	FLJ11292	ACYP2
LOC10012926	CSN1S1	FAM151A	DIS3	GLT25D2	FLJ21986	ADA
LOC10012943	CST6	FAM176A	DISC1	GNA14	FLJ23152	ADAL
LOC10012944	CUBN	FAM188B	DISP2	GPR101	FLJ26850	ADAM10
LOC10013008	CXCR6	FAM76A	DKFZp761E19	GPR146	FLJ35258	ADAM15
LOC10013018	CYP19A1	FARP2	DKK4	GPR182	FLJ37078	ADAM17
LOC10013032	CYP1A1	FBXL21	DMRTA2	GPR55	FLJ41484	ADAM19
LOC10013050	CYP26B1	FBXL3	DNAJC16	GPR83	FLJ45513	ADAM9
LOC10013065	CYP3A7	FBXO4	DNM1	GRIA3	FOSB	ADAMTS1
LOC10013099	CYP4V2	FCRLB	DNM3	GRM4	FOXF1	ADAMTS18

LOC10013125	DCAF4L1	FGB	DOC2A	GRM8	FOXL2	ADAMTS19
LOC10013128	DCDC2	FGG	DOK7	HBS1L	GABBR2	ADAMTS3
LOC10013148	DCLK2	FIGN	DPF1	HDAC5	GAS2L1	ADAMTS6
LOC10013169	DDX60	FLJ27354	DRD3	HHLA2	GBP2	ADAMTS9
LOC10013170	DEFA6	FLJ33630	DYRK3	HIST1H1D	GGT7	ADAMTSL2
LOC10013174	DHH	FLJ35429	EBF2	HIST1H1E	GHRHR	ADAMTSL4
LOC10013196	DHRS4L1	FLJ37644	EBI2	HIST1H4J	GLCCI1	ADAP2
LOC10013196	DIO1	FLJ41200	ECE1	HLA-DMB	GMFG	ADAR
LOC10013196	DKFZp434M1:	FLJ46838	ECEL1	HLA-DPB2	GNB3	ADARB1
LOC10013198	DKFZp686J05:	FMO1	ECSCR	HMGB3L1	GOLGA8E	ADAT1
LOC10013228	DKFZP779L18	FN1	ELAVL3	HMP19	GP6	ADAT3
LOC10013236	DLC1	FOLH1	ELAVL4	HOXD3	GPBAR1	ADCK1
LOC10013255	DLG1	FOXE3	ESRRB	HR	GPD2	ADCK2
LOC10013264	DLX1	FOXI3	F8	HRC	GPR124	ADCK4
LOC10013273	DMPK	FTCD	FAM101B	HSPC047	GRAMD2	ADCK5
LOC10013288	DMRT1	GAGE2B	FAM124B	HTR3A	GREM1	ADCY1
LOC10013289	DNHD1	GATA5	FAM13AOS	ICAM4	GRK4	ADCY3
LOC10013293	DOCK9	GCM1	FAM150B	IFT81	GTPBP5	ADCY6
LOC10013295	DPRXP4	GHDC	FAM159A	IGSF9B	GUCY2C	ADCY7
LOC10013297	DSC1	GIN1	FAM159B	IL1RAPL1	GVIN1	ADCY9
LOC10013346	DSCR4	GJA3	FAM169A	IL2RB	HDAC8	ADD1
LOC10013347	DTX3L	GJB1	FAM43B	IL3RA	HEMK1	ADD3
LOC10013369	DUOX1	GJB2	FAM53A	INHBB	HES2	ADH5
LOC10013371	DUOX2	GLRB	FAM57B	JAKMIP2	HIF3A	ADHFE1
LOC10013402	DUOXA2	GLUD2	FAM62C	KCND2	HIP1R	ADI1
LOC10013403	DUSP5P	GLUL	FAM65C	KCNG3	HIRA	ADIPOR1
LOC10013406	DYNLRB2	GNAQ	FAM71F1	KCTD8	HIST1H2BC	ADIPOR2
LOC10013417	EFCAB3	GOLGA7B	FAM78B	KIAA1086	HIST1H2BE	ADK
LOC10013422	EFHA2	GOLSYN	FAM90A1	KIAA1409	HMGA2	ADM
LOC10013426	EGR2	GPC5	FANCA	KIF13A	HP1BP3	ADM2
LOC10013433	ELAC1	GPR156	FBN1	KLHL25	HPDL	ADNP
LOC10013459	ELOVL3	GPRC5A	FBXL17	KLHL33	HSD11B2	ADNP2
LOC10013471	ELOVL7	GRAMD1C	FBXL19	KLKB1	HSN2	ADO
LOC10013471	EMX2	GRHL1	FCHO1	KRT126P	HTR2A	ADORA1
LOC10013482	ENOX1	GRHL3	FCN1	LCK	HTRA4	ADORA2A
LOC121838	ERAP1	GSK3A	FCRL5	LEFTY1	IFIT2	ADORA2B
LOC133874	ERAP2	GSTA1	FERMT3	LGALS12	IFT57	ADPGK
LOC138864	ESM1	GSTA2	FEZF2	LHFPL4	IGF1R	ADPRH
LOC149351	ETAA1	GSTA5	FGF17	LHX6	IL1RAP	ADPRHL2
LOC150223	EXD3	GTF2IRD2P	FGF8	LOC10012837	IL6	ADRA2A
LOC150759	EXOD1	GULP1	FGFBP3	LOC10012847	IRF8	ADRA2B
LOC196993	F8A3	HAAO	FLJ20581	LOC10012907	ITGA1	ADRBK1
LOC220930	FAM123B	HCN4	FLJ21839	LOC10012919	ITGAM	ADRM1
LOC284276	FAM22D	HEXIM1	FLJ23754	LOC10012960	KCNK7	ADSL
LOC284293	FAM23B	HEYL	FLJ27365	LOC10013017	KIAA0226	ADSS
LOC285216	FAM55C	HHAT	FLJ27465	LOC10013028	KIAA0284	ADSSL1
LOC285548	FBXO9	HIST1H2AC	FLJ30430	LOC10013045	KIAA1024	AEBP1
LOC340529	FCGR2A	HIST1H2BG	FLJ36144	LOC10013082	KIAA1202	AEBP2
LOC360030	FER1L5	HIST1H4H	FLJ39632	LOC10013093	KIAA1875	AEN
LOC389124	FGD2	HLA-DPA1	FLJ40194	LOC10013094	KIF27	AES

LOC389458	FLJ13197	HMGCS2	FLJ40473	LOC10013122	KLC3	AFAP1
LOC390282	FLJ16779	HNF1A	FLJ45422	LOC10013126	KLK7	AFAP1L2
LOC390712	FLJ22639	HOXA2	FLJ45445	LOC10013147	KRT18P34	AFF1
LOC392781	FLJ22675	HPX	FLJ90231	LOC10013172	LAMB3	AFF4
LOC393076	FLJ35776	HS6ST1	FMN2	LOC10013173	LCAT	AFG3L1
LOC401233	FLJ37786	HSD17B2	FOXF2	LOC10013189	LIF	AFG3L2
LOC402571	FLJ38773	HSD17B7P2	FOXO6	LOC10013234	LMBR1L	AFMID
LOC440131	FLJ41327	HSD3B7	FOXP3	LOC10013266	LOC10004971	AFP
LOC440181	FLJ41941	HSPA1L	FPGS	LOC10013283	LOC10012791	AFTPH
LOC440330	FLJ44313	ID4	FRMPD4	LOC10013293	LOC10012792	AGA
LOC440577	FLJ45983	IGF1	FSHR	LOC10013303	LOC10012835	AGAP3
LOC441007	FOXR1	IGF2	FSTL4	LOC10013324	LOC10012851	AGAP6
LOC441054	FRMD3	IL21R	FSTL5	LOC10013331	LOC10012852	AGAP8
LOC441487	FRMD7	INSR	FUSSEL18	LOC10013340	LOC10012854	AGBL5
LOC441714	FXYD2	ISM1	FZD10	LOC10013343	LOC10012859	AGFG1
LOC441957	FYB	ISM2	GAFA2	LOC10013391	LOC10012872	AGFG2
LOC442726	GABRB1	ITGA7	GALNT13	LOC10013415	LOC10012881	AGGF1
LOC541471	GAGE12E	ITPKA	GGT1	LOC10013417	LOC10012890	AGK
LOC541472	GAGE4	ITPR1	GH1	LOC10013420	LOC10012906	AGL
LOC553137	GALIG	JRK	GH2	LOC10013478	LOC10012909	AGPAT1
LOC641788	GARNL3	KBTBD3	GHRLOS	LOC10013481	LOC10012930	AGPAT2
LOC641806	GBAP	KCNAB2	GIGYF1	LOC127099	LOC10012936	AGPAT4
LOC642113	GBP1	KCTD18	GJA4	LOC220115	LOC10012938	AGPAT5
LOC642420	GCKR	KIAA0251	GKN2	LOC283314	LOC10012954	AGPAT6
LOC642615	GCNT3	KIAA1045	GLB1L3	LOC284998	LOC10012957	AGPS
LOC642660	GDF6	KIAA1199	GLT1D1	LOC285141	LOC10012958	AGR2
LOC642675	GEFT	KIAA1772	GLYATL1	LOC285407	LOC10012967	AGRN
LOC643236	GINS1	KISS1R	GNAL	LOC285412	LOC10012972	AGTPBP1
LOC643304	GJA5	KLF10	GNASAS	LOC339782	LOC10013009	AGTRAP
LOC643731	GJB6	KLF3	GNAZ	LOC374491	LOC10013041	AGXT2L2
LOC643853	GJB7	KLK8	GPR172B	LOC388755	LOC10013052	AHCTF1
LOC643896	GNGT2	KNCN	GPR176	LOC389895	LOC10013090	AHCY
LOC643959	GPR61	KRR1	GRAP	LOC391764	LOC10013092	AHCYL1
LOC644006	GPR87	KRT7	GRIK5	LOC400174	LOC10013095	AHCYL2
LOC644558	GSTA3	KRT80	GRIN2C	LOC400406	LOC10013101	AHDC1
LOC644573	GSTM3	KRTAP21-2	GRM3	LOC440386	LOC10013116	AHI1
LOC644584	HAPLN3	KYNU	GRP	LOC440925	LOC10013117	AHNAK
LOC644589	HCG27	LDHC	GRRP1	LOC441081	LOC10013125	AHNAK2
LOC644596	HDX	LGR4	GSC	LOC441511	LOC10013127	AHR
LOC644715	HERV-FRD	LMO1	GSTT2	LOC442308	LOC10013128	AHSA1
LOC644766	HIST1H2AG	LOC10012800	GUCA2A	LOC474170	LOC10013132	AHSA2
LOC644999	HIST1H3D	LOC10012808	GYPB	LOC493754	LOC10013136	AHSP
LOC645084	HKDC1	LOC10012820	GYPE	LOC641941	LOC10013144	AIDA
LOC645249	HOXB3	LOC10012865	HCLS1	LOC642280	LOC10013164	AIF1
LOC645284	HOXC4	LOC10012882	HCRTR2	LOC642341	LOC10013172	AIF1L
LOC645314	HPD	LOC10012910	HDC	LOC642342	LOC10013215	AIFM1
LOC645323	HSD17B1	LOC10012912	HELB	LOC643669	LOC10013226	AIG1
LOC645620	HSD3B1	LOC10012929	HHEX	LOC643719	LOC10013228	AIM1
LOC645944	HSF2BP	LOC10012941	HHLA1	LOC643912	LOC10013247	AIM1L
LOC646191	HSPA6	LOC10012950	HIST1H3J	LOC643932	LOC10013252	AIM2

LOC646330	HSPB7	LOC10012963	HP	LOC644451	LOC10013294	AIMP2
LOC646346	HSPG2	LOC10012965	HRASLS2	LOC644738	LOC10013296	AIP
LOC646372	IAPP	LOC10012967	HRES1	LOC645682	LOC10013342	AIRE
LOC646697	ICAM1	LOC10012970	HRIHFB2122	LOC645733	LOC10013362	AJAP1
LOC646938	IFIH1	LOC10012996	HSFYP1	LOC646438	LOC10013374	AK1
LOC647083	IFIT3	LOC10012998	IFI30	LOC646869	LOC10013376	AK2
LOC647274	IFIT5	LOC10013021	IGSF21	LOC647042	LOC10013379	AK2P2
LOC647328	IFNE	LOC10013025	IL15	LOC647439	LOC10013384	AK3
LOC647588	IGFBP1	LOC10013054	IL20	LOC647570	LOC10013439	AK3L1
LOC647592	IGFBPL1	LOC10013055	IL34	LOC648377	LOC10013443	AKAP1
LOC647630	IL17C	LOC10013055	IL8RB	LOC649023	LOC10013458	AKAP10
LOC647841	IL18R1	LOC10013093	IMPG2	LOC649305	LOC10013466	AKAP11
LOC648863	IL1R1	LOC10013096	INDO	LOC650132	LOC10013470	AKAP12
LOC649571	IL1R2	LOC10013108	IRAK3	LOC652051	LOC10017093	AKAP13
LOC650251	IL20RA	LOC10013112	ITGA2B	LOC652790	LOC152586	AKAP7
LOC650418	IL32	LOC10013136	ITIH2	LOC653075	LOC282997	AKAP8
LOC650509	INHA	LOC10013157	ITK	LOC653337	LOC283332	AKAP8L
LOC650867	INSL4	LOC10013170	ITPRIPL1	LOC653895	LOC284648	AKIRIN1
LOC651212	ITGA10	LOC10013171	JMJD2A	LOC653907	LOC286002	AKIRIN2
LOC652140	ITGA8	LOC10013189	KCNB1	LOC654053	LOC338963	AKNA
LOC652300	ITGB6	LOC10013193	KCNH3	LOC727751	LOC339047	AKR1A1
LOC652346	ITIH1	LOC10013206	KCNIP3	LOC727815	LOC341689	AKR1B1
LOC652657	JCLN	LOC10013229	KCNRG	LOC728288	LOC388282	AKR1C2
LOC652670	KBTBD10	LOC10013235	KIAA0853	LOC728522	LOC388458	AKR1C3
LOC652773	KC6	LOC10013239	KIAA0953	LOC728729	LOC388630	AKR1C4
LOC653189	KCNJ14	LOC10013256	KIAA1644	LOC728887	LOC389634	AKR1D1
LOC653284	KCNK9	LOC10013277	KIF17	LOC728927	LOC389641	AKR7A2
LOC653303	KCNQ4	LOC10013290	KIF21B	LOC729264	LOC390940	AKR7A3
LOC653423	KHK	LOC10013291	KIF6	LOC729350	LOC391429	AKT1
LOC653515	KIAA0258	LOC10013303	KIRREL2	LOC729378	LOC392713	AKT1S1
LOC654042	KIAA1407	LOC10013307	KLF1	LOC729581	LOC399959	AKT3
LOC654264	KIAA1683	LOC10013308	KLF7	LOC729732	LOC400955	AKTIP
LOC727831	KIAA1908	LOC10013322	KLHL35	LOC730063	LOC400986	ALAD
LOC727849	KIFC3	LOC10013405	KLHL4	LOC730100	LOC401007	ALAS1
LOC727869	KLHDC10	LOC10013425	KRT3	LOC730385	LOC401052	ALB
LOC728599	KLK11	LOC10013436	L3MBTL4	LOC732445	LOC401252	ALCAM
LOC728612	KLK3	LOC10013444	LAG3	LOC96610	LOC401622	ALDH16A1
LOC728934	KRBA2	LOC10013449	LAMA3	LPAR4	LOC441124	ALDH18A1
LOC728945	KRT17	LOC10013455	LAT	LRAT	LOC441193	ALDH1A1
LOC729113	KRT23	LOC10013462	LDHD	LRIG3	LOC441251	ALDH1A3
LOC729562	KRTAP19-6	LOC10013470	LGI1	LRP12	LOC441737	ALDH1B1
LOC729623	KRTAP4-8	LOC124216	LGI4	LRRC26	LOC442041	ALDH1L2
LOC729652	KSR1	LOC146909	LHFPL5	LYPD2	LOC442249	ALDH2
LOC729905	LCORL	LOC148137	LHX1	LYPLA2P1	LOC554208	ALDH3A2
LOC730058	LGALS13	LOC154761	LHX2	LZTS1	LOC641819	ALDH4A1
LOC730268	LHB	LOC155100	LILRB5	MALL	LOC641983	ALDH5A1
LOC730474	LIG4	LOC158572	LIMD2	MAP2K6	LOC642325	ALDH6A1
LOC730964	LIPC	LOC283849	LMO3	MAP3K15	LOC642377	ALDH7A1
LOC731074	LMF1	LOC283922	LMO7	MAPK12	LOC642809	ALDH8A1
LOC731075	LMOD1	LOC284422	LOC10009363	MAPK1IP1L	LOC642838	ALDH9A1

LOC731432	LNX1	LOC285733	LOC10012788	MAST1	LOC642995	ALDOA
LOC732432	LOC10012826	LOC286135	LOC10012791	MAZ	LOC643109	ALDOC
LOC85391	LOC10012830	LOC339799	LOC10012794	MESP1	LOC643187	ALG1
LPPR1	LOC10012847	LOC340274	LOC10012795	MGAT4C	LOC643373	ALG10B
LRRC3B	LOC10012867	LOC344328	LOC10012809	MIR1224	LOC643396	ALG11
LRRC4C	LOC10012876	LOC374443	LOC10012829	MIR1323	LOC643981	ALG12
LRRC67	LOC10012885	LOC387770	LOC10012840	MIR302C	LOC644043	ALG13
MAGEA9B	LOC10012912	LOC387790	LOC10012872	MIR365-1	LOC644092	ALG14
MAOB	LOC10012913	LOC388327	LOC10012874	MIXL1	LOC644284	ALG1L
MARK4	LOC10012914	LOC389118	LOC10012890	MKX	LOC644297	ALG3
MBLAC1	LOC10012929	LOC389332	LOC10012928	MLLT3	LOC644424	ALG5
MESDC2	LOC10012946	LOC390251	LOC10012932	MMP25	LOC644884	ALG6
MGC5139	LOC10012969	LOC392382	LOC10012939	MPP2	LOC645079	ALG8
MIR106A	LOC10013001	LOC400558	LOC10012946	MUC12	LOC645159	ALG9
MIR1275	LOC10013038	LOC400743	LOC10012949	MYH6	LOC645241	ALK
MIR199B	LOC10013059	LOC400831	LOC10012977	NANOG	LOC645313	ALKBH1
MIR363	LOC10013066	LOC401431	LOC10013008	NAP1L2	LOC645330	ALKBH2
MIR376C	LOC10013079	LOC402116	LOC10013044	NDUFB4	LOC645411	ALKBH4
MIR383	LOC10013080	LOC440525	LOC10013051	NEFL	LOC645464	ALKBH5
MIR507	LOC10013084	LOC441032	LOC10013052	NELL1	LOC645638	ALKBH6
MIR564	LOC10013103	LOC441378	LOC10013067	NKD1	LOC645743	ALKBH7
MIR761	LOC10013121	LOC441632	LOC10013073	NKX6-2	LOC645963	ALKBH8
MIR92A2	LOC10013131	LOC441864	LOC10013076	NMNAT3	LOC646038	ALMS1
MIR939	LOC10013133	LOC442064	LOC10013080	NOTUM	LOC646194	ALOX15B
MR1	LOC10013147	LOC553158	LOC10013081	NPFFR2	LOC646434	ALOX5
MS4A13	LOC10013165	LOC554207	LOC10013104	NPL	LOC646496	ALPK2
MSX2P1	LOC10013189	LOC641518	LOC10013104	NPTX2	LOC646990	ALPL
MYCNOS	LOC10013202	LOC642267	LOC10013139	NRTN	LOC647250	ALPP
MYF6	LOC10013220	LOC642648	LOC10013168	NTM	LOC647589	ALS2
MYH11	LOC10013249	LOC642732	LOC10013176	NTN3	LOC647650	ALS2CR14
MYLPF	LOC10013264	LOC642769	LOC10013183	NUDT10	LOC647827	ALS2CR4
NCRNA00160	LOC10013311	LOC642787	LOC10013204	NUDT4	LOC647928	ALX1
NDUFC2	LOC10013317	LOC643389	LOC10013209	OLFM1	LOC648169	AMAC1L3
NEFM	LOC10013340	LOC643647	LOC10013216	ONECUT2	LOC648226	AMACR
NFKBIL1	LOC10013343	LOC643888	LOC10013250	OPRK1	LOC648366	AMBRA1
NFS1	LOC10013355	LOC643911	LOC10013256	OR10AG1	LOC648570	AMD1
NRG3	LOC10013355	LOC643985	LOC10013270	OR4S1	LOC648814	AMDHD2
NRXN1	LOC10013355	LOC644001	LOC10013307	OXER1	LOC649270	AMFR
NUDT16P	LOC10013357	LOC644096	LOC10013318	PCDH20	LOC649711	AMHR2
NUPL1	LOC10013366	LOC644384	LOC10013319	PCDH21	LOC649841	AMMECR1
OPCML	LOC10013386	LOC644544	LOC10013331	PCSK9	LOC649999	AMMECR1L
OR2A14	LOC10013400	LOC644612	LOC10013347	PDE12	LOC650111	AMN1
OR5D18	LOC10013403	LOC644632	LOC10013358	PDE3B	LOC650155	AMOT
OR6Y1	LOC10013414	LOC644694	LOC10013360	PDZD4	LOC650227	AMOTL2
OR7G2	LOC10013416	LOC644701	LOC10013365	PGBD5	LOC650346	AMT
OR7G3	LOC10013425	LOC644733	LOC10013374	PIK3R3	LOC650546	AMY1A
PCDH9	LOC10013441	LOC644763	LOC10013377	PLCH1	LOC650580	AMY1B
PCDHA7	LOC10013453	LOC644992	LOC10013388	PLD4	LOC650683	AMY1C
PCGF3	LOC10014460	LOC645195	LOC10013393	PLEKHB1	LOC650706	AMZ2
PDYN	LOC10018894	LOC645434	LOC10013400	PMCHL1	LOC650757	ANAPC1

PLA2G2A	LOC10019237	LOC645659	LOC10013428	PNMA2	LOC651073	ANAPC10
PPBPL2	LOC113230	LOC645969	LOC10013435	PNMA3	LOC651309	ANAPC11
PPIL4	LOC126520	LOC646111	LOC10013476	PODXL2	LOC652044	ANAPC13
PRCD	LOC162073	LOC646208	LOC10013482	POLR1A	LOC652078	ANAPC4
PRM3	LOC220729	LOC646491	LOC126767	POM121L4P	LOC652226	ANAPC5
PRODH2	LOC255275	LOC646513	LOC128322	POMZP3	LOC652234	ANG
PROP1	LOC255326	LOC646533	LOC131873	POU5F1	LOC652570	ANGEL1
PRR20E	LOC283588	LOC646568	LOC133185	PPAPDC1A	LOC652589	ANGEL2
PRSS1	LOC283767	LOC646572	LOC144383	PPAPDC3	LOC652697	ANGPT1
PTCHD2	LOC283874	LOC646981	LOC149620	PPID	LOC653034	ANGPTL2
PTH2R	LOC284296	LOC647169	LOC152024	PPP1R16B	LOC653100	ANGPTL4
PTPRT	LOC284297	LOC647195	LOC157627	PRAC	LOC653115	ANK3
RAX	LOC285047	LOC647262	LOC165186	PRDM14	LOC653188	ANKDD1A
RCP9	LOC338667	LOC647264	LOC200420	PRIM2	LOC653197	ANKFY1
RDH12	LOC340156	LOC647488	LOC201175	PRKAR1B	LOC653257	ANKHD1
REG3G	LOC387856	LOC648608	LOC202181	PRKCB	LOC653269	ANKHD1-EIF4E
RGS21	LOC388436	LOC648963	LOC221136	PRKCB1	LOC653349	ANKIB1
RMRP	LOC388514	LOC649210	LOC255620	PROK2	LOC653352	ANKLE2
RNU86	LOC388559	LOC649801	LOC283116	PRPH2	LOC653545	ANKMY1
RSHL3	LOC388565	LOC649897	LOC283155	PRR16	LOC653717	ANKMY2
RTN1	LOC388681	LOC650020	LOC283174	PRR7	LOC653720	ANKRA2
SCG3	LOC388814	LOC650254	LOC283683	PRRX2	LOC654201	ANKRD1
SCN9A	LOC389102	LOC650339	LOC284428	PRSS42	LOC654253	ANKRD10
SCRT1	LOC390372	LOC650780	LOC285016	psiTPTE22	LOC654342	ANKRD11
SECTM1	LOC392843	LOC650815	LOC285296	PTPRZ1	LOC723805	ANKRD12
SEMA3D	LOC400145	LOC651029	LOC285620	RAB3C	LOC727759	ANKRD13A
SENP8	LOC401588	LOC651621	LOC286076	RAI2	LOC727832	ANKRD13C
SFRP5	LOC402560	LOC651772	LOC339742	RASA3	LOC727913	ANKRD13D
SFRS12IP1	LOC402693	LOC651777	LOC339760	RASGRP4	LOC727950	ANKRD16
SLC13A2	LOC415056	LOC652291	LOC339809	RASL10A	LOC728147	ANKRD17
SLC22A6	LOC439936	LOC652634	LOC340094	RBM43	LOC728262	ANKRD20A1
SLC25A31	LOC440836	LOC652669	LOC340970	REST	LOC728308	ANKRD20B
SLC26A8	LOC440978	LOC652698	LOC342934	RET	LOC728683	ANKRD22
SLC2A9	LOC440993	LOC652699	LOC349114	RFTN2	LOC728711	ANKRD24
SLC30A6	LOC441151	LOC652736	LOC388002	RGR	LOC728790	ANKRD26
SLC35D1	LOC441208	LOC652837	LOC388237	RGS1	LOC728942	ANKRD27
SMCR7	LOC442421	LOC652904	LOC388255	RINL	LOC729008	ANKRD28
SNHG10	LOC554223	LOC653105	LOC388925	RLTPR	LOC729173	ANKRD30B
SNORD58B	LOC641365	LOC653110	LOC391703	RNF175	LOC729176	ANKRD32
SNX15	LOC641801	LOC653333	LOC391727	RNF2	LOC729198	ANKRD33
SOS2	LOC641823	LOC653383	LOC392264	RPS6KA6	LOC729260	ANKRD34A
SPAG6	LOC641999	LOC653696	LOC399940	RTP1	LOC729602	ANKRD35
SV2B	LOC642062	LOC654135	LOC400707	RYR1	LOC729609	ANKRD36B
SYN3	LOC642097	LOC727721	LOC400958	SBNO1	LOC729659	ANKRD37
SYPL2	LOC642109	LOC727797	LOC401442	SCARNA12	LOC729739	ANKRD38
SYT9	LOC642369	LOC727866	LOC401911	SCNN1G	LOC729793	ANKRD39
TCEAL5	LOC642486	LOC727997	LOC402176	SEMA4A	LOC730007	ANKRD40
TCTN2	LOC642635	LOC728493	LOC440350	SHE	LOC730130	ANKRD44
TDH	LOC642759	LOC728748	LOC440508	SHISA3	LOC730153	ANKRD46
TDRG1	LOC642953	LOC728831	LOC441179	SHISA4	LOC730236	ANKRD47

TGM6	LOC643008	LOC729020	LOC441416	SIRT6	LOC730387	ANKRD49
TMC5	LOC643012	LOC729051	LOC441505	SLC1A2	LOC730517	ANKRD50
TMCO4	LOC643018	LOC729065	LOC441666	SLC25A24	LOC730809	ANKRD52
TMEM110	LOC643222	LOC729212	LOC441806	SLC7A14	LOC730995	ANKRD54
TMEM173	LOC643240	LOC729667	LOC641693	SLC8A2	LOC732419	ANKRD57
TMEM202	LOC643339	LOC729852	LOC641922	SLITRK4	LOC732450	ANKRD6
TMPRSS4	LOC643382	LOC730087	LOC642073	SMCHD1	LOC91664	ANKRD9
TNXA	LOC643388	LOC730092	LOC642154	SMCP	LOC93349	ANKS1A
TOP	LOC643466	LOC730226	LOC642160	SNORA65	LOH3CR2A	ANKS1B
TRIM15	LOC643605	LOC730358	LOC642362	SNORA79	LOX	ANKS3
TRPV4	LOC643841	LOC730393	LOC642381	SNORA7A	LOXHD1	ANKZF1
TTC29	LOC644019	LOC731196	LOC642456	SNORD100	LOXL4	ANLN
UBE2B	LOC644083	LOC90499	LOC642788	SNORD15B	LPPR2	ANO10
UCP1	LOC644313	LOH12CR2	LOC642833	SNORD25	LRRC37A	ANO2
UFD1L	LOC644322	LPAR5	LOC642935	SNORD32A	LRRC37A4	ANO6
USP53	LOC644391	LRRC16B	LOC643296	SNORD33	LRRC56	ANO8
WARS2	LOC644415	LRRC29	LOC643550	SNORD55	LSM11	ANO9
WDR3	LOC644436	LY6H	LOC643556	SNORD96A	MAMDC4	ANP32A
WIPF2	LOC644496	MAP1A	LOC643700	SOBP	MAML3	ANP32B
WNT3A	LOC644644	MAP2	LOC643712	SOX10	MAN2A2	ANP32C
XK	LOC644672	MAP7D2	LOC643815	SOX12	MAP1LC3B2	ANPEP
XPOT	LOC644814	MAPK14	LOC643817	SPATA4	MAP9	ANTXR1
ZCCHC16	LOC644844	09. Mrz	LOC643836	SPINK2	MAPK8IP2	ANTXR2
ZCWPW2	LOC644990	MBD5	LOC643879	SRD5A3	MEF2A	ANXA1
ZMYND12	LOC645135	MDM1	LOC644012	SRY	MEGF9	ANXA11
ZNF214	LOC645172	MECP2	LOC644113	ST6GALNAC5	MGC13005	ANXA2
ZNF233	LOC645212	MEI1	LOC644234	STK32A	MGC26718	ANXA2P1
ZNF385B	LOC645365	MEP1A	LOC644366	STK33	MGC5457	ANXA2P2
ZNF566	LOC645367	MGC45800	LOC644580	SYT4	MIR1253	ANXA2P3
ZNF747	LOC645478	MGP	LOC644641	T	MIR29B1	ANXA3
ZNF836	LOC645520	MIR181C	LOC644695	TAF3	MIR604	ANXA5
ZNF92	LOC645627	MIR21	LOC644714	TAF4B	MLL3	ANXA6
ZSCAN20	LOC645671	MIR635	LOC644836	TBX22	MLLT1	ANXA7
	LOC645676	MOCS2	LOC645010	TDGF1	MLLT4	AOF2
	LOC645723	MORC3	LOC645188	TDRKH	MMP14	AP1B1
	LOC645835	MORN3	LOC645217	THBS2	MMP9	AP1G1
	LOC646008	MOXD1	LOC645335	THG1L	MUC4	AP1G2
	LOC646012	MREG	LOC645722	TIGD1	MUC6	AP1GBP1
	LOC646128	MSRA	LOC645848	TLE6	MYCL1	AP1M1
	LOC646154	MTTP	LOC645967	TMEM132D	MYCT1	AP1M2
	LOC646312	MUC15	LOC645974	TMEM145	MYH3	AP1S1
	LOC646403	MYL3	LOC646300	TMEM155	MYL2	AP1S2
	LOC646576	MYLK	LOC646304	TMEM169	NACAD	AP2A1
	LOC646585	NANP	LOC646422	TMPRSS11E2	NBPF11	AP2A2
	LOC646632	NAT1	LOC646867	TNFRSF14	NCF4	AP2B1
	LOC646746	NLRP3	LOC646934	TNMD	NCRNA00173	AP2M1
	LOC646762	NOL4	LOC647082	TOP1P1	NEB	AP2S1
	LOC646774	NOSTRIN	LOC647295	TOR2A	NEIL1	AP3B1
	LOC646779	NPAS3	LOC647359	TOX3	NLRP2	AP3B2
	LOC646804	NPNT	LOC647499	TPTE2	NNMT	AP3D1

LOC646906	NRF1	LOC647521	TRDMT1	NOG	AP3M1
LOC647054	NRXN3	LOC647606	TRIM46	NOS3	AP3M2
LOC647060	NTNG1	LOC647854	TRIM61	NR0B1	AP3S1
LOC647515	NUDT13	LOC647911	TRIM73	NR4A2	AP3S2
LOC647568	NUDT8	LOC647993	TRPS1	NRG1	AP4B1
LOC647955	NUP98	LOC648039	TTC28	NTF5	AP4E1
LOC647979	OCLN	LOC648069	TTC9	NTN1	AP4M1
LOC647987	OR2H2	LOC648138	TTLL6	NXF4	APAF1
LOC648130	OSR1	LOC648205	TTYH1	ODF3B	APBA2BP
LOC648196	OTUD7B	LOC648716	TUBA3E	OLFML2A	APBA3
LOC648293	OVOL1	LOC648815	TUBA4A	OR2W3	APBB1IP
LOC648585	P2RY2	LOC648855	UPK1A	OSBP2	APBB3
LOC648600	PAFAH1B2	LOC649199	USH2A	PACRGL	APEG1
LOC648682	PCBP3	LOC649282	USP44	PAIP2B	APEH
LOC648744	PCDHB10	LOC649292	UTP20	PATE1	APEX1
LOC648984	PCDHB13	LOC649604	VAX2	PBX4	APEX2
LOC649025	PCLO	LOC649635	VCX2	PCDHGA12	APH1A
LOC649186	PCSK6	LOC649702	VENTX	PDE4DIP	APH1B
LOC649379	PDE8B	LOC650003	VSNL1	PDE8A	API5
LOC649500	PELI3	LOC650036	WBSCR17	PDGFB	APIP
LOC649613	PFN4	LOC650037	WNT8A	PDK1	APITD1
LOC649620	PID1	LOC650061	ZC3H13	PDLIM5	APLNR
LOC649680	PIWIL4	LOC650239	ZDHHC22	PDXDC2	APLP1
LOC649977	PKP3	LOC650823	ZFPM2	PEAR1	APLP2
LOC650293	PLA2G12B	LOC650845	ZNF132	PHF6	APOA1
LOC650562	PLA2G2C	LOC650922	ZNF280A	PIP5K1A	APOA1BP
LOC650628	PLA2G7	LOC650950	ZNF385D	PIP5K1B	APOA2
LOC651102	PLAC8	LOC651075	ZNF43	PLAUR	APOA4
LOC651169	PLD6	LOC651511	ZNF460	PLCH2	APOBEC3C
LOC651213	PLP2	LOC651951	ZNF510	PLD5	APOBEC3D
LOC651520	PM20D2	LOC652003	ZNF533	POLH	APOBEC3F
LOC651558	POSTN	LOC652045	ZNF57	PPM2C	APOBEC3G
LOC651680	PPM1J	LOC652175	ZNF620	PPP1R1B	APOC1
LOC651997	PPP1R1C	LOC652183	ZNF642	PPP1R3E	APOE
LOC652094	PRKCE	LOC652260	ZNF643	PRDM5	APOL3
LOC652326	PRR15	LOC652543	ZNF713	PRDM7	APOLD1
LOC652627	PSD4	LOC652606	ZNF81	PRH1	APOM
LOC652630	RAP1A	LOC652635	ZNF844	PRO0628	APOO
LOC652674	RARB	LOC652684	ZSCAN10	PRPF39	APOOL
LOC652688	RARRES1	LOC652721		PSG4	APP
LOC652713	RASGRP1	LOC652722		PTCHD1	APPBP2
LOC652771	RB1	LOC652762		PTPN7	APPL1
LOC652839	RBAK	LOC652805		PVRL1	APPL2
LOC652846	RBP2	LOC653066		PXMP4	APRT
LOC653157	RBP4	LOC653113		QPCTL	APTX
LOC653199	REEP1	LOC653194		RALGPS2	AQP11
LOC653204	REL	LOC653319		RAMP2	AQP12B
LOC653234	RETNLB	LOC653355		RASGRP2	AQR
LOC653316	RFPL1	LOC653641		RBM24	AR
LOC653539	RFX6	LOC653689		RBMS3	ARAF

LOC653652	RGAG1	LOC727737
LOC653701	RGAG4	LOC727937
LOC653796	RGN	LOC728441
LOC653853	RP11-49G10.8	LOC728566
LOC653876	RP2	LOC728671
LOC653962	RPH3AL	LOC728673
LOC654002	RRM2	LOC729010
LOC654078	RUNX1	LOC729225
LOC654128	S100P	LOC729231
LOC727882	SCP2	LOC729486
LOC727901	SCUBE3	LOC729487
LOC727924	SDCCAG8	LOC729832
LOC728178	SDK1	LOC729854
LOC728205	SDR39U1	LOC730032
LOC728216	SENP1	LOC730086
LOC728291	SERP2	LOC730281
LOC728362	SERPINC1	LOC730291
LOC728400	SERPINF2	LOC730686
LOC728519	SGCG	LOC730919
LOC728543	SGMS1	LOC730953
LOC728664	SH2B2	LOC731496
LOC728811	SH3BGR	LOC92017
LOC728876	SH3BP5	LOC93432
LOC728924	SHANK2	LPPR5
LOC728929	SHD	LRRC2
LOC729209	SIK1	LRRC33
LOC729374	SLC12A5	LRRK1
LOC729408	SLC13A5	LTC4S
LOC729433	SLC16A14	LUZP6
LOC729530	SLC16A6	M160
LOC729558	SLC1A4	MAGEB3
LOC729642	SLC22A7	MAGI2
LOC729645	SLC23A1	MAGI3
LOC729828	SLC30A2	MAMLD1
LOC729837	SLC34A1	MAP1D
LOC730036	SLC38A11	MAP6
LOC730392	SLC39A5	MAPK11
LOC730396	SLC39A9	04. Mrz
LOC730644	SLC41A2	MAST4
LOC730877	SLC6A12	MBD3L2
LOC730952	SLC6A4	MCC
LOC731227	SLC7A9	MDFI
LOC731718	SLFN11	MEG3
LOC731742	SLN	METTL11B
LOC732160	SMAD9	METTL8
LOC732229	SMOC2	MGAT5B
LOC732443	SMTNL2	MGC13168
LONRF2	SNORA5B	MGC40168
LPL	SNORD114-2	MIAT
LRCH1	SNORD78	MIR1208

RCSD1	ARAP1
RDH16	ARAP3
RGL4	ARCN1
RGPD5	ARD1A
RGS11	ARF1
RGS20	ARF3
RIMBP3B	ARF4
RND1	ARF5
RNU105C	ARF6
RNU11	ARFGAP1
RNU4-1	ARFGAP2
RP9P	ARFGAP3
SAMD10	ARFGEF1
SAMD14	ARFGEF2
SAMD4A	ARFIP1
SCAF1	ARG2
SCARNA3	ARGLU1
SCFD2	ARHGAP1
SDC3	ARHGAP10
SELE	ARHGAP11A
SEMA3B	ARHGAP12
SEMA6D	ARHGAP15
13. Sep	ARHGAP17
SFI1	ARHGAP19
SFN	ARHGAP21
SFXN3	ARHGAP22
SHOX2	ARHGAP23
SHROOM1	ARHGAP24
SLC14A1	ARHGAP25
SLC22A1	ARHGAP27
SLC4A3	ARHGAP28
SLC6A16	ARHGAP4
SLC9A9	ARHGAP9
SMC1A	ARHGDIA
SNAP25	ARHGDIB
SNORA11E	ARHGEF1
SNORA28	ARHGEF10
SNORA71A	ARHGEF10L
SNORA72	ARHGEF11
SNORA77	ARHGEF12
SNORA80	ARHGEF16
SNORD11	ARHGEF17
SNORD123	ARHGEF18
SNORD69	ARHGEF19
SNORD87	ARHGEF2
SNORD99	ARHGEF3
SOCS7	ARHGEF5
SPANXA2	ARHGEF5L
SPANXB1	ARHGEF6
SPARCL1	ARHGEF7

LRMP	SNORD89	MIR1234
LRRC38	SOAT2	MIR1267
LTB	SOCS6	MIR331
LTF	SOS1	MIR720
LY6G6C	SOSTDC1	MIRLET7I
LYG1	SPAG4	MLKL
LYPD5	SPRN	MMP16
LYST	SPTLC3	MOBP
MAL	SRD5A2L2	MPP3
MAP3K13	SRR	MPV17L
MAPKBP1	SRRM5	MRAS
MATN1	SSFA2	MRGPRF
MCF2L2	SUMO4	MRM1
MDM2	SYK	MT3
MED12L	SYNPO2	MYLC2PL
METTL2B	TAAR8	MYO1G
MFSD9	TAC1	MYRIP
MGC23270	TAC4	MYSM1
MGC35440	TBC1D8B	MYST4
MGC52498	TCF21	MYT1
MGLL	TCP10L	NCRNA00113
MIA	TEX11	NCRNA00162
MICALL2	TFAP2B	NECAB1
MIP	TFCP2L1	NFASC
MIR1247	THAP3	NFIA
MIR128-2	THAP8	NFIC
MIR1915	THBS1	NGFR
MIR212	THEX1	NHLH2
MIR30C2	THSD3	NKAIN1
MIR326	TIGD6	NKX2-5
MIR448	TIGIT	NLF2
MIR450A1	TLR6	NLRP5
MIR532	TM4SF18	NMUR2
MIR574	TM6SF2	NOS1AP
MIR577	TMCC3	NOS2
MIR642	TMEM105	NPM2
MIR708	TMEM191C	NPPA
MIRLET7D	TMEM20	NPTX1
MMP1	TMEM37	NR1I2
MMP21	TMEM56	NRN1L
MPP7	TMEM68	NRP2
MRGPRX1	TMEM8B	NTN4
MRGPRX3	TMOD2	NTRK1
MRGPRX4	TMPO	ONECUT1
MSLN	TMPRSS13	OR52N2
MT4	TMTC2	OR5AP2
MTSS1L	TNFSF12-TNFS	OR5K4
MTUS2	TRIM17	OVOS2
MXRA8	TRIM62	PACRG
MYBPC3	TRPV6	PADI3

SPATA13	ARID1A
SPTBN4	ARID2
SRCAP	ARID3A
SRPX2	ARID3B
SSX3	ARID4A
ST3GAL4	ARID4B
STAC	ARID5B
STK32C	ARIH1
SULT4A1	ARIH2
SYCE2	ARL1
TBC1D21	ARL13B
TBN	ARL15
TFEC	ARL16
TFPI2	ARL17B
TIMP3	ARL17P1
TM4SF1	ARL2
TMEM171	ARL2BP
TMEM31	ARL3
TNFRSF10A	ARL4A
TNFRSF1B	ARL4C
TNFSF12	ARL4D
TNFSF9	ARL5A
TNRC18	ARL5B
TP53I11	ARL6IP1
TPPP3	ARL6IP4
TRAF3	ARL6IP5
TREX1	ARL6IP6
TRH	ARL8B
TRPV2	ARMC1
TSPY3	ARMC10
UBE2NL	ARMC5
UBR1	ARMC6
UNC5C	ARMC7
UNC5CL	ARMC8
USP19	ARMC9
VAPA	ARMCX1
VAPB	ARMCX2
VIPR2	ARMCX3
VPS13B	ARMCX4
VWA3A	ARMCX5
WDR17	ARMCX6
WFDC10B	ARMET
WNK4	ARNT
ZBTB7B	ARNT2
ZC3HAV1L	ARPC1A
ZMYND17	ARPC1B
ZNF236	ARPC2
ZNF276	ARPC3
ZNF345	ARPC4
ZNF346	ARPC5

MYBPHL	TSGA10	PAQR5
MYL1	TSPYL4	PAX6
MYLK4	TTC30A	PAX7
MYO7A	TTLL7	PCDH24
MYOM1	TTR	PCDHAC1
NAT8B	TWF1	PCDHAC2
NEU4	UBA7	PCDHGA10
NHEDC2	UGT2B7	PCDHGC3
NIPAL1	ULBP2	PDE2A
NIPAL4	ULK4	PDE4B
NKAIN4	UMPS	PENK
NKPD1	UNC93A	PGA3
NKX2-3	UPK2	PIPOX
NLRC3	UPK3B	PKLR
NR6A1	USP43	PLA2G3
NTF4	VGLL1	PLCB2
NTN5	VIL1	PLCB4
NTRK2	VN1R2	PLEKHH2
NXPH2	VTN	PNMA6A
OAS2	WAPAL	PPAN-P2RY11
OAS3	WDR47	PPARGC1B
OCA2	WFIKKN1	PPFIA4
ODAM	WNT11	PPIF
OLFM2	WNT6	PPP2R2C
OLFML1	XAGE2B	PRAMEF9
OLR1	XKR4	PRDM2
OPHN1	XKRX	PRDM6
OR1L8	YLPM1	PRDM9
OR2L13	ZBTB16	PRG4
OR2T12	ZBTB32	PRPF40B
OR52N5	ZCCHC4	PRR12
OR7E91P	ZDHHC15	PTAFR
OSM	ZDHHC20	PTGDS
OSR2	ZFP62	PTGS1
OXGR1	ZNF184	RAB11B
P2RX6	ZNF230	RAB26
P8	ZNF311	RAB33A
PA2G4P4	ZNF404	RALGAPA2
PADI2	ZNF429	RAPGEF3
PALMD	ZNF552	RASAL1
PARD6B	ZNF587	RASD2
PATZ1	ZNF611	RASGRF2
PBLD	ZNF623	RCC1
PCP4	ZNF703	RD3
PDAP1	ZNF740	RELT
PDZD3	ZNF750	REM2
PGBD4	ZNF774	RERGL
PGPEP1	ZNRF2	RFPL2
PHF8	ZXDB	RFPL3
PHLDA2		RGS14

ZNF542	ARPC5L
ZNF563	ARPP19
ZNF628	ARRB1
ZNF699	ARRDC2
ZNF771	ARRDC3
ZNF790	ARS2
ZNF8	ARSA
	ARSB
	ARSD
	ARSE
	ARSG
	ARSK
	ART1
	ART5
	ARV1
	AS3MT
	ASAH1
	ASAH2C
	ASAM
	ASAP1
	ASAP2
	ASAP3
	ASB1
	ASB13
	ASB3
	ASB6
	ASB7
	ASB8
	ASCC1
	ASCC2
	ASCC3
	ASF1A
	ASF1B
	ASGR1
	ASH2L
	ASL
	ASMTL
	ASNA1
	ASNS
	ASNSD1
	ASPH
	ASPHD2
	ASPM
	ASPSCR1
	ASRGL1
	ASS1
	ASTE1
	ASTN2
	ASXL1
	ASXL2

PHYHIPL	RGS7	ATAD1
PI4KA	RHEBL1	ATAD2
PIGZ	RHPN1	ATAD2B
PJCG6	RIMBP2	ATAD3A
PKD1L2	RIT2	ATE1
PLA2G16	RNF125	ATF1
PLAC2	RNU6ATAC	ATF2
PLEK2	RNY4	ATF3
PLEKHG4B	RORB	ATF4
PLEKHG5	RPRML	ATF5
PLSCR5	RTN4RL1	ATF6
PMFBP1	S100Z	ATF7IP2
PNPLA4	S1PR5	ATG10
PODN	SALL1	ATG12
POPDC2	SAMD3	ATG16L1
PPARA	SAMSN1	ATG16L2
PPEF1	SCARNA14	ATG2A
PPIL6	SCARNA18	ATG3
PRAMEF13	SCARNA22	ATG4A
PRKAA2	SCARNA8	ATG4B
PROX1	SCARNA9L	ATG4C
PRSS22	SCG2	ATG4D
PRTG	SCN4A	ATG5
PSCA	SCN4B	ATG7
PSD2	SCNN1B	ATG9A
PSG2	SDCBP2	ATHL1
PSG3	SEMG1	ATIC
PSG5	SERHL2	ATL1
PSG6	SERPINB5	ATL2
PSG7	SEZ6	ATL3
PTK9	SFTA1P	ATM
PVRL4	SH2D2A	ATMIN
PZP	SH2D3C	ATN1
QRSL1	SH3RF3	ATOX1
RABL5	SHC3	ATP10A
RAD18	SLAMF6	ATP11B
RAPSN	SLC10A4	ATP11C
RASA2	SLC17A9	ATP12A
RASEF	SLC24A5	ATP13A1
RBL1	SLC25A35	ATP13A2
RBM19	SLC26A10	ATP1A1
RBMY3AP	SLC2A6	ATP1A3
REV3L	SLC30A4	ATP1B1
RFT1	SLC34A2	ATP1B3
RFXDC2	SLC45A1	ATP2A2
RGP1	SLC6A1	ATP2B1
RICTOR	SLC7A10	ATP2B4
RIN3	SLCO5A1	ATP2C1
RLN1	SLITRK5	ATP5A1
RNASE6	SNAI1	ATP5B

RNF180	SNAI3	ATP5C1
RNF32	SNORA21	ATP5D
ROPN1B	SNORA3	ATP5E
RPL32P3	SNORA34	ATP5EP2
RRAD	SNORD10	ATP5F1
RSPO1	SNORD109B	ATP5G1
S100A3	SNORD11B	ATP5G2
SAC	SNORD12	ATP5G3
SBSN	SNORD12B	ATP5H
SCARNA9	SNORD14B	ATP5I
SCN2B	SNORD48	ATP5J
SCRG1	SNORD53	ATP5J2
SCXA	SNORD56	ATP5L
SDR16C5	SNORD76	ATP5O
SEC15L2	SNORD91A	ATP5S
SEMA7A	SPACA1	ATP5SL
01. Sep	SPANXA1	ATP6AP1
SERPINA3	SPATA3	ATP6AP1L
SERPINB7	SPOCD1	ATP6AP2
SERPINB8	SSX4	ATP6V0A1
SERPINE3	ST8SIA1	ATP6V0A2
SH3TC1	ST8SIA2	ATP6V0A4
SIM2	ST8SIA5	ATP6V0B
SIRPD	STK32B	ATP6V0C
SLA	STXBP4	ATP6V0D1
SLC10A1	SULT2B1	ATP6V0E1
SLC15A2	SYNGR3	ATP6V0E2
SLC1A6	TAS2R4	ATP6V1A
SLC22A11	TCEB3CL	ATP6V1B1
SLC22A18AS	TDGF3	ATP6V1B2
SLC22A4	TDRD12	ATP6V1C1
SLC23A3	TEC	ATP6V1D
SLC24A2	TEKT2	ATP6V1E1
SLC25A34	TEKT3	ATP6V1E2
SLC30A8	TESC	ATP6V1F
SLC35E2	TEX14	ATP6V1G1
SLC38A8	TFF3	ATP6V1G2
SLC4A4	TGFA	ATP6V1H
SLC7A4	THEM4	ATP7B
SLC9A2	THSD7B	ATP8B2
SLCO6A1	TINAG	ATP9A
SMPD4	TKTL1	ATP9B
SMPX	TMCC2	ATPAF1
SNORA31	TMEFF2	ATPAF2
SNORA49	TMEM103	ATPBD1B
SNORA55	TMEM150C	ATPBD3
SOD3	TMEM151	ATPBD4
SP140	TMEM16D	ATPIF1
SPATA22	TMEM201	ATRIP
SPDYA	TMEM52	ATRN

SPEF2
SPNS3
SSH1
SST
SSTR1
STELLAR
STS
STX18
STX19
SUFU
SULT1C4
SULT2A1
SYNC1
SYTL3
TAS2R10
TBC1D26
TBRG1
TF
TGFBR1
TGM3
THRSP
TIFA
TINAGL1
TM4SF4
TM4SF5
TMEM117
TMEM140
TMEM161B
TMEM176A
TMEM194B
TMEM217
TMEM40
TMEM86A
TMPRSS6
TMPRSS7
TncRNA
TNFSF10
TNKS2
TP53TG3
TP73L
TPCN1
TRAF1
TRAF3IP3
TREM1
TREML2
TRIM43
TRIM63
TRIM67
TSHZ2
TSPAN1

TMEM61
TMEM74
TNFAIP2
TNFRSF11A
TNFRSF11B
TNFSF11
TNNC2
TNP1
TREML1
TRIM47
TRIM59
TRPC6
TRPM3
TRPM6
TRPM7
TSPAN8
TTC21A
UNC5D
USHBP1
USP2
UTF1
UTS2D
VANGL1
VOPP1
VSTM1
WDR88
WIPF3
WNK2
WNT10B
ZAN
ZAP70
ZCCHC12
ZFR2
ZMAT4
ZNF414
ZNF516
ZNF530
ZSCAN22
ZXDA

ATXN1
ATXN10
ATXN1L
ATXN2
ATXN2L
ATXN3
ATXN7L2
ATXN7L3
AUH
AUP1
AURKA
AURKAIP1
AURKB
AUTS2
AVEN
AVL9
AVP
AVPI1
AVPR2
AXIN1
AXIN2
AXL
AXUD1
AYP1p1
AZI1
AZI2
AZIN1
B2M
B3GALNT1
B3GALNT2
B3GALT4
B3GALT6
B3GALTL
B3GAT3
B3GNT1
B3GNT2
B3Gn-T6
B3GNT6
B3GNT8
B3GNTL1
B4GALNT4
B4GALT1
B4GALT2
B4GALT3
B4GALT4
B4GALT5
B4GALT6
B4GALT7
B9D1
B9D2

TSPAN2
TSPY1
TTC18
TTC30B
TTN
TTTY17A
TUBAL3
UBASH3B
UBD
UBE2QP2
UBQLN3
UBQLNL
UGT2B28
UNC13A
UNC13D
UNG2
USP45
VIT
VMO1
VTI1A
WASF1
WHSC1
WISP1
WNT10A
WWTR1
XAGE1D
XCR1
ZBED2
ZBTB47
ZBTB7C
ZC3H7B
ZCCHC10
ZDHHC14
ZFP28
ZFP57
ZFP92
ZNF292
ZNF321
ZNF350
ZNF469
ZNF71
ZNF717
ZNF831
ZSCAN4

BACE1
BACE2
BACH2
BAD
BAG2
BAG3
BAG4
BAG5
BAGE5
BAHCC1
BAHD1
BAIAP2
BAIAP2L1
BAIAP2L2
BAK1
BAMBI
BANF1
BANP
BAP1
BAPX1
BARD1
BASP1
BAT1
BAT2
BAT2D1
BAT2L
BAT3
BAT4
BAT5
BAX
BAZ1A
BAZ1B
BAZ2B
BBC3
BBS1
BBS10
BBS12
BBS2
BBS4
BBS7
BBS9
BBX
BCAP29
BCAP31
BCAR1
BCAR3
BCAS2
BCAS4
BCAT1
BCAT2

BCCIP
BCDIN3D
BCHE
BCKDHA
BCKDHB
BCKDK
BCL11A
BCL11B
BCL2
BCL2L1
BCL2L11
BCL2L12
BCL2L13
BCL2L2
BCL3
BCL6
BCL7A
BCL7B
BCL7C
BCL9
BCL9L
BCLAF1
BCOR
BCORL1
BCR
BCS1L
BCYRN1
BDNF
BECN1
BEGAIN
BEND3
BEND4
BEND5
BEND7
BET1
BET1L
BEX1
BEX2
BEX4
BEX5
BEXL1
BFAR
BGLAP
BGN
BHLHB2
BHLHB9
BICD1
BICD2
BID
BIK

BIN1
BIN3
BIRC2
BIRC3
BIRC5
BIRC6
BIVM
BLCAP
BLM
BLMH
BLOC1S1
BLOC1S2
BLVRA
BLVRB
BLZF1
BMF
BMI1
BMP1
BMP2
BMP2K
BMP4
BMP5
BMP6
BMP7
BMP8B
BMPER
BMPR1A
BMPR2
BMS1
BMS1P5
BNC2
BNIP1
BNIP2
BNIP3
BNIP3L
BNIPL
BOAT
BOK
BOLA1
BOLA2
BOLA3
BOP1
BP75
BPGM
BPHL
BPNT1
BPTF
BRAF
BRCA1
BRCC3

BRD1
BRD2
BRD3
BRD7
BRD7P2
BRD8
BRD9
BRE
BRF1
BRF2
BRI3
BRI3BP
BRI3P1
BRIX1
BRMS1
BRMS1L
BRP44
BRP44L
BRPF1
BRPF3
BRSK1
BRSK2
BRWD1
BRWD2
BRWD3
BSCL2
BSDC1
BSG
BSN
BSPRY
BST2
BTAF1
BTBD1
BTBD10
BTBD12
BTBD15
BTBD2
BTBD3
BTBD6
BTBD7
BTD
BTF3
BTF3L4
BTG1
BTG2
BTG3
BTN2A1
BTN2A2
BTN3A1
BTRC

BUB1
BUB1B
BUB3
BUD13
BUD31
BVES
BYSL
BZW2
C10orf104
C10orf114
C10orf118
C10orf119
C10orf12
C10orf125
C10orf137
C10orf140
C10orf2
C10orf26
C10orf28
C10orf32
C10orf33
C10orf35
C10orf4
C10orf47
C10orf51
C10orf57
C10orf58
C10orf59
C10orf6
C10orf61
C10orf75
C10orf76
C10orf78
C10orf82
C10orf84
C10orf88
C11orf1
C11orf10
C11orf17
C11orf2
C11orf24
C11orf35
C11orf46
C11orf47
C11orf48
C11orf49
C11orf51
C11orf54
C11orf57
C11orf58

C11orf59
C11orf60
C11orf61
C11orf63
C11orf67
C11orf68
C11orf70
C11orf73
C11orf74
C11orf75
C11orf80
C11orf82
C11orf83
C11orf84
C11orf9
C12orf10
C12orf11
C12orf23
C12orf24
C12orf26
C12orf29
C12orf30
C12orf31
C12orf32
C12orf35
C12orf4
C12orf41
C12orf43
C12orf44
C12orf45
C12orf47
C12orf48
C12orf49
C12orf5
C12orf51
C12orf52
C12orf57
C12orf60
C12orf62
C12orf64
C12orf65
C12orf66
C12orf68
C12orf76
C12orf77
C13orf1
C13orf15
C13orf18
C13orf23
C13orf25

C13orf27
C13orf3
C13orf34
C13orf37
C13orf7
C14orf100
C14orf102
C14orf104
C14orf106
C14orf109
C14orf112
C14orf118
C14orf124
C14orf125
C14orf129
C14orf131
C14orf132
C14orf133
C14orf135
C14orf138
C14orf139
C14orf142
C14orf143
C14orf145
C14orf147
C14orf149
C14orf153
C14orf156
C14orf159
C14orf166
C14orf167
C14orf169
C14orf173
C14orf174
C14orf179
C14orf181
C14orf19
C14orf2
C14orf28
C14orf32
C14orf37
C14orf4
C14orf43
C14orf45
C14orf78
C14orf79
C14orf80
C14orf82
C14orf85
C14orf93

C15orf17
C15orf20
C15orf21
C15orf23
C15orf24
C15orf27
C15orf28
C15orf29
C15orf33
C15orf38
C15orf39
C15orf40
C15orf41
C15orf42
C15orf44
C15orf57
C15orf63
C16orf13
C16orf33
C16orf35
C16orf42
C16orf48
C16orf5
C16orf52
C16orf53
C16orf56
C16orf57
C16orf58
C16orf59
C16orf61
C16orf62
C16orf63
C16orf67
C16orf68
C16orf7
C16orf70
C16orf72
C16orf74
C16orf75
C16orf79
C16orf80
C16orf86
C16orf87
C16orf88
C16orf91
C16orf93
C17orf100
C17orf101
C17orf106
C17orf28

C17orf37
C17orf39
C17orf41
C17orf42
C17orf44
C17orf45
C17orf47
C17orf48
C17orf49
C17orf51
C17orf53
C17orf56
C17orf58
C17orf59
C17orf61
C17orf62
C17orf63
C17orf65
C17orf68
C17orf69
C17orf70
C17orf71
C17orf78
C17orf79
C17orf80
C17orf81
C17orf85
C17orf88
C17orf89
C17orf90
C17orf91
C17orf95
C17orf96
C17orf97
C17orf98
C18orf10
C18orf18
C18orf21
C18orf22
C18orf25
C18orf26
C18orf32
C18orf45
C18orf54
C18orf55
C18orf56
C18orf8
C19orf10
C19orf12
C19orf2

C19orf20
C19orf22
C19orf24
C19orf25
C19orf28
C19orf29
C19orf30
C19orf31
C19orf33
C19orf38
C19orf39
C19orf40
C19orf42
C19orf43
C19orf44
C19orf46
C19orf47
C19orf48
C19orf50
C19orf52
C19orf53
C19orf54
C19orf56
C19orf6
C19orf60
C19orf61
C19orf62
C19orf63
C19orf64
C19orf70
C1D
C1GALT1
C1GALT1C1
C1orf102
C1orf104
C1orf106
C1orf107
C1orf109
C1orf112
C1orf115
C1orf116
C1orf122
C1orf123
C1orf124
C1orf128
C1orf130
C1orf131
C1orf133
C1orf135
C1orf144

C1orf149
C1orf151
C1orf152
C1orf156
C1orf159
C1orf162
C1orf163
C1orf165
C1orf166
C1orf172
C1orf174
C1orf187
C1orf19
C1orf190
C1orf198
C1orf201
C1orf203
C1orf21
C1orf212
C1orf216
C1orf218
C1orf24
C1orf25
C1orf26
C1orf31
C1orf35
C1orf41
C1orf43
C1orf50
C1orf52
C1orf53
C1orf54
C1orf55
C1orf56
C1orf57
C1orf59
C1orf61
C1orf63
C1orf66
C1orf69
C1orf71
C1orf74
C1orf77
C1orf83
C1orf85
C1orf86
C1orf88
C1orf89
C1orf9
C1orf91

C1orf93
C1orf96
C1orf97
C1QBP
C1QTNF6
C1RL
C1S
C20orf100
C20orf108
C20orf11
C20orf111
C20orf117
C20orf12
C20orf127
C20orf160
C20orf177
C20orf191
C20orf194
C20orf196
C20orf199
C20orf20
C20orf24
C20orf27
C20orf29
C20orf3
C20orf30
C20orf4
C20orf43
C20orf45
C20orf46
C20orf52
C20orf55
C20orf56
C20orf7
C20orf72
C20orf75
C20orf94
C21orf122
C21orf124
C21orf126
C21orf129
C21orf2
C21orf24
C21orf30
C21orf33
C21orf37
C21orf45
C21orf51
C21orf55
C21orf56

C21orf57
C21orf58
C21orf59
C21orf66
C21orf7
C21orf70
C21orf81
C21orf91
C22orf13
C22orf25
C22orf28
C22orf29
C22orf30
C22orf32
C22orf33
C22orf36
C22orf39
C22orf40
C22orf9
C2CD2
C2orf15
C2orf18
C2orf21
C2orf24
C2orf25
C2orf28
C2orf29
C2orf30
C2orf34
C2orf37
C2orf42
C2orf43
C2orf44
C2orf47
C2orf49
C2orf56
C2orf63
C2orf64
C2orf68
C2orf69
C2orf7
C2orf76
C2orf79
C2orf82
C3orf1
C3orf10
C3orf14
C3orf17
C3orf18
C3orf19

C3orf21
C3orf23
C3orf26
C3orf31
C3orf33
C3orf34
C3orf37
C3orf38
C3orf39
C3orf50
C3orf52
C3orf58
C3orf59
C3orf63
C3orf64
C3orf67
C3orf72
C3orf75
C4A
C4orf10
C4orf14
C4orf18
C4orf23
C4orf27
C4orf29
C4orf31
C4orf33
C4orf34
C4orf41
C4orf43
C4orf46
C4orf48
C4orf49
C5
C5orf13
C5orf15
C5orf21
C5orf22
C5orf24
C5orf25
C5orf28
C5orf30
C5orf32
C5orf33
C5orf34
C5orf35
C5orf37
C5orf39
C5orf4
C5orf41

C5orf42
C5orf44
C5orf5
C5orf51
C5orf53
C5orf54
C5orf62
C6orf1
C6orf106
C6orf108
C6orf111
C6orf115
C6orf117
C6orf120
C6orf124
C6orf125
C6orf129
C6orf130
C6orf132
C6orf134
C6orf136
C6orf138
C6orf141
C6orf145
C6orf148
C6orf153
C6orf160
C6orf162
C6orf163
C6orf167
C6orf168
C6orf170
C6orf173
C6orf182
C6orf192
C6orf203
C6orf211
C6orf225
C6orf26
C6orf47
C6orf48
C6orf52
C6orf54
C6orf57
C6orf59
C6orf61
C6orf62
C6orf64
C6orf66
C6orf70

C6orf72
C6orf85
C6orf89
C7orf10
C7orf11
C7orf13
C7orf20
C7orf23
C7orf25
C7orf26
C7orf27
C7orf28A
C7orf28B
C7orf29
C7orf30
C7orf36
C7orf38
C7orf40
C7orf41
C7orf42
C7orf43
C7orf44
C7orf47
C7orf49
C7orf50
C7orf54
C7orf55
C7orf59
C7orf63
C7orf68
C7orf70
C8orf33
C8orf37
C8orf38
C8orf40
C8orf41
C8orf42
C8orf45
C8orf48
C8orf55
C8orf58
C8orf59
C8orf76
C8orf83
C9orf100
C9orf102
C9orf103
C9orf10OS
C9orf114
C9orf116

C9orf119
C9orf123
C9orf126
C9orf127
C9orf130
C9orf135
C9orf140
C9orf142
C9orf156
C9orf16
C9orf167
C9orf169
C9orf21
C9orf23
C9orf25
C9orf30
C9orf37
C9orf40
C9orf45
C9orf46
C9orf5
C9orf58
C9orf6
C9orf61
C9orf64
C9orf69
C9orf7
C9orf72
C9orf75
C9orf78
C9orf80
C9orf82
C9orf85
C9orf86
C9orf89
C9orf9
C9orf90
C9orf91
C9orf93
C9orf95
CA11
CA13
CA14
CA2
CA4
CA5B
CAB39
CAB39L
CABC1
CABIN1

CABLES1
CABLES2
CABYR
CACHD1
CACNA1C
CACNA1H
CACNA2D2
CACNB2
CACNB3
CACNG6
CACYBP
CAD
CADM1
CADM4
CADPS2
CALB2
CALCB
CALCOCO1
CALCOCO2
CALD1
CALHM2
CALM1
CALM2
CALM3
CALML4
CALN1
CALR
CALU
CAMK2D
CAMK2G
CAMK2N1
CAMKK2
CAMLG
CAMSAP1
CAMSAP1L1
CAMTA1
CAND1
CAND2
CANT1
CANX
CAP1
CAP2
CAPG
CAPN1
CAPN12
CAPN13
CAPN2
CAPN5
CAPN6
CAPN7

CAPNS1
CAPRIN1
CAPRIN2
CAPS
CAPS2
CAPZA1
CAPZA2
CAPZB
CARD10
CARD11
CARD14
CARD8
CARHSP1
CARKD
CARM1
CARS
CARS2
CART1
CASC3
CASC4
CASC5
CASD1
CASK
CASKIN2
CASP2
CASP3
CASP6
CASP7
CASP9
CAST
CASZ1
CAT
CATSPER2
CATSPER2P1
CAV1
CAV2
CBARA1
CBFA2T2
CBFB
CBL
CBLB
CBLC
CBLL1
CBLN3
CBR1
CBR3
CBR4
CBS
CBWD1
CBWD3

CBWD5
CBX1
CBX2
CBX3
CBX4
CBX5
CBX6
CBX7
CBY1
CC2D1A
CC2D1B
CC2D2A
CCAR1
CCBE1
CCBL1
CCBL2
CCDC101
CCDC102A
CCDC104
CCDC105
CCDC106
CCDC107
CCDC109A
CCDC109B
CCDC112
CCDC113
CCDC115
CCDC117
CCDC12
CCDC120
CCDC121
CCDC123
CCDC124
CCDC125
CCDC127
CCDC128
CCDC130
CCDC132
CCDC135
CCDC136
CCDC137
CCDC138
CCDC14
CCDC15
CCDC150
CCDC151
CCDC152
CCDC16
CCDC18
CCDC21

CCDC22
CCDC23
CCDC24
CCDC25
CCDC28A
CCDC28B
CCDC3
CCDC34
CCDC41
CCDC43
CCDC45
CCDC46
CCDC47
CCDC49
CCDC5
CCDC50
CCDC51
CCDC53
CCDC55
CCDC56
CCDC58
CCDC59
CCDC6
CCDC64B
CCDC66
CCDC69
CCDC71
CCDC72
CCDC74B
CCDC76
CCDC77
CCDC8
CCDC84
CCDC85B
CCDC86
CCDC88C
CCDC9
CCDC90A
CCDC90B
CCDC91
CCDC92
CCDC93
CCDC94
CCDC97
CCDC99
CCHCR1
CCKBR
CCL2
CCL5
CCM2

CCNA1
CCNA2
CCNB1
CCNB1IP1
CCNB2
CCNC
CCND1
CCND2
CCND3
CCNDBP1
CCNE1
CCNE2
CCNF
CCNG1
CCNG2
CCNH
CCNI
CCNI2
CCNJL
CCNK
CCNL1
CCNL2
CCNO
CCNT1
CCNT2
CCNY
CCNYL1
CCPG1
CCR6
CCRK
CCRN4L
CCS
CCT2
CCT3
CCT4
CCT5
CCT6A
CCT6B
CCT6P1
CCT7
CCT8
CD14
CD151
CD164
CD1D
CD24
CD248
CD276
CD2AP
CD2BP2

CD320
CD34
CD44
CD46
CD47
CD52
CD55
CD58
CD59
CD63
CD68
CD72
CD74
CD79A
CD79B
CD81
CD82
CD83
CD86
CD9
CD97
CD99
CD99L2
CDAN1
CDC123
CDC14A
CDC14B
CDC16
CDC2
CDC20
CDC23
CDC25A
CDC25B
CDC25C
CDC26
CDC2L1
CDC2L2
CDC2L5
CDC2L6
CDC34
CDC37
CDC40
CDC42
CDC42BPA
CDC42BPB
CDC42EP1
CDC42EP2
CDC42EP4
CDC42EP5
CDC42SE1

CDC42SE2
CDC45L
CDC5L
CDC7
CDCA1
CDCA2
CDCA3
CDCA4
CDCA5
CDCA7
CDCA7L
CDCA8
CDCP1
CDH1
CDH10
CDH11
CDH13
CDH2
CDH24
CDH3
CDH6
CDIPT
CDK10
CDK2
CDK2AP1
CDK2AP2
CDK3
CDK4
CDK5
CDK5R1
CDK5RAP1
CDK5RAP2
CDK5RAP3
CDK6
CDK7
CDK9
CDKAL1
CDKL2
CDKL3
CDKN1A
CDKN1B
CDKN1C
CDKN2AIP
CDKN2AIPNL
CDKN2D
CDKN3
CDO1
CDR2
CDR2L
CDRT4

CDS1
CDS2
CDT1
CDV3
CDX1
CDX2
CDYL
CEBPA
CEBPB
CEBPD
CEBPG
CEBPZ
CECR1
CECR5
CECR6
CECR7
CEL
CELSR2
CELSR3
CEMP1
CENPA
CENPB
CENPBD1
CENPC1
CENPE
CENPF
CENPH
CENPI
CENPJ
CENPK
CENPL
CENPM
CENPN
CENPO
CENPP
CENPQ
CENPT
CENPV
CENTA1
CENTB2
CENTD2
CENTG2
CENTG3
CEP110
CEP135
CEP152
CEP164
CEP192
CEP250
CEP27

CEP290
CEP350
CEP55
CEP57
CEP63
CEP68
CEP70
CEP72
CEP78
CEPT1
CERCAM
CERK
CES1
CES2
CES3
CETN2
CETN3
CFD
CFDP1
CFI
CFL1
CFL2
CFLAR
CFTR
CGGBP1
CGI-96
CGN
CGNL1
CGRRF1
CHAC1
CHAF1A
CHAF1B
CHCHD1
CHCHD10
CHCHD2
CHCHD3
CHCHD4
CHCHD5
CHCHD6
CHCHD7
CHCHD8
CHCHD9
CHD1
CHD1L
CHD2
CHD3
CHD4
CHD6
CHD7
CHD8

CHD9
CHDH
CHEK1
CHEK2
CHERP
CHES1
CHFR
CHI3L1
CHIC2
CHKA
CHKB
CHM
CHML
CHMP1A
CHMP1B
CHMP2A
CHMP2B
CHMP4A
CHMP4B
CHMP4C
CHMP5
CHMP6
CHMP7
CHN1
CHN2
CHORDC1
CHP
CHPF
CHPF2
CHPT1
CHRAC1
CHRM3
CHRNA3
CHRNA5
CHRNB1
CHST10
CHST12
CHST13
CHST14
CHST15
CHST2
CHST3
CHST7
CHST9
CHSY1
CHSY3
CHTF18
CHUK
CHURC1
CIAO1

CIAPIN1
CIB1
CIB2
CIC
CICE
CICK0721Q.1
CINP
CIP29
CIR1
CIRBP
CIRH1A
CISD1
CISD2
CISD3
CITED2
CITED4
CIZ1
CKAP2
CKAP2L
CKAP4
CKAP5
CKB
CKLF
CKMT1A
CKMT1B
CKS1B
CKS2
CLASP1
CLASP2
CLCC1
CLCF1
CLCN2
CLCN3
CLCN5
CLCN6
CLCN7
CLCNKA
CLDN1
CLDN10
CLDN11
CLDN12
CLDN14
CLDN15
CLDN19
CLDN20
CLDN23
CLDN3
CLDN4
CLDN6
CLDN7

CLDND1
CLDND2
CLEC11A
CLEC16A
CLEC2D
CLEC4A
CLIC1
CLIC4
CLIC6
CLINT1
CLIP1
CLIP2
CLIP3
CLK1
CLK2
CLK3
CLK4
CLMN
CLN3
CLN5
CLN6
CLN8
CLNS1A
CLOCK
CLP1
CLPP
CLPTM1
CLPTM1L
CLPX
CLRN1
CLSTN1
CLSTN3
CLTA
CLTB
CLTC
CLTCL1
CLUAP1
CLYBL
CMAS
CMBL
CMC1
CMIP
CMPK1
CMTM3
CMTM4
CMTM6
CMTM7
CMTM8
CMYA5
CNBP

CNDP2
CNFN
CNGB1
CNIH
CNIH2
CNIH4
CNKSR1
CNKSR3
CNN2
CNN3
CNNM3
CNNM4
CNO
CNOT1
CNOT10
CNOT2
CNOT3
CNOT4
CNOT6
CNOT6L
CNOT7
CNOT8
CNPY2
CNPY3
CNPY4
CNRIP1
CNTFR
CNTLN
CNTN1
CNTNAP1
CNTNAP2
CNTNAP5
CNTROB
COASY
COBL
COBLL1
COBRA1
COCH
COG1
COG2
COG3
COG4
COG5
COG6
COG7
COG8
COIL
COL11A1
COL11A2
COL12A1

COL13A1
COL16A1
COL17A1
COL18A1
COL1A1
COL1A2
COL22A1
COL23A1
COL2A1
COL3A1
COL4A1
COL4A2
COL4A3BP
COL4A5
COL4A6
COL5A1
COL5A2
COL6A1
COL6A2
COL6A3
COL7A1
COL8A2
COL9A2
COL9A3
COLEC12
COLQ
COMMD1
COMMD10
COMMD2
COMMD3
COMMD4
COMMD5
COMMD6
COMMD7
COMMD8
COMMD9
COMT
COMTD1
COPA
COPB1
COPB2
COPE
COPG
COPG2
COPG2IT1
COPS2
COPS3
COPS4
COPS5
COPS6

COPS7A
COPS7B
COPS8
COPZ1
COQ10A
COQ10B
COQ2
COQ3
COQ4
COQ5
COQ6
COQ7
COQ9
CORO1B
CORO1C
CORO2A
CORO7
COTL1
COX10
COX11
COX15
COX17
COX19
COX4I1
COX4NB
COX5A
COX5B
COX6A1
COX6B1
COX6C
COX7A2
COX7A2L
COX7B
COX7C
COX8A
CP110
CPD
CPE
CPEB2
CPEB3
CPM
CPN2
CPNE1
CPNE3
CPNE8
CPOX
CPS1
CPSF1
CPSF2
CPSF3

CPSF3L
CPSF4
CPSF6
CPT1A
CPT1B
CPT2
CPVL
CPXM1
CPXM2
CPZ
CRABP1
CRABP2
CRADD
CRAMP1L
CRAT
CRB3
CRBN
CRCP
CREB1
CREB3
CREB3L2
CREB3L4
CREB5
CREBBP
CREBL1
CREBL2
CREBZF
CREG1
CRELD1
CRELD2
CRHBP
CRHR1
CRIP1
CRIP2
CRIPAK
CRIPT
CRISPLD1
CRISPLD2
CRK
CRKL
CRKRS
CRLF3
CRLS1
CRMP1
CRNKL1
CROCC
CROP
CROT
CRSP2
CRSP6

CRSP9
CRTAP
CRTC2
CRTC3
CRY1
CRY2
CRYGS
CRYL1
CRYM
CRYZ
CRYZL1
CS
CSAD
CSDA
CSDE1
CSE1L
CSF2RA
CSHL1
CSK
CSNK1A1
CSNK1D
CSNK1E
CSNK1G1
CSNK1G2
CSNK1G3
CSNK2A1
CSNK2A1P
CSNK2A2
CSNK2B
CSPP1
CSRNP2
CSRP1
CSRP2
CSRP2BP
CST1
CST3
CST4
CSTB
CSTF2
CSTF2T
CSTF3
CTAGE6
CTBP1
CTBP2
CTBS
CTCF
CTDP1
CTDSP1
CTDSP2
CTDSPL

CTDSPL2
CTF1
CTGF
CTGLF3
CTGLF7
CTH
CTHRC1
CTNNA1
CTNNAL1
CTNNB1
CTNNBIP1
CTNNBL1
CTNND1
CTNND2
CTNS
CTPS
CTPS2
CTR9
CTSA
CTSB
CTSC
CTSD
CTSH
CTSK
CTSL1
CTSL2
CTSZ
CTTN
CTTNBP2NL
CTU2
CTXN1
CUEDC1
CUEDC2
CUGBP1
CUL1
CUL2
CUL4A
CUL4B
CUL5
CUL7
CUL9
CUTA
CUTC
CUTL1
CUX1
CWC15
CWC22
CWF19L1
CWF19L2
CX3CL1

CXADR
CXCL12
CXCL14
CXCL16
CXCR7
CXorf12
CXorf23
CXorf26
CXorf36
CXorf38
CXorf39
CXorf40A
CXorf40B
CXorf45
CXorf57
CXorf64
CXXC1
CXXC4
CXXC5
CXXC6
CYB561
CYB561D1
CYB561D2
CYB5A
CYB5B
CYB5D1
CYB5D2
CYB5R1
CYB5R2
CYB5R3
CYB5R4
CYB5RL
CYBA
CYBASC3
CYBRD1
CYC1
CYCS
CYCSL1
CYFIP1
CYFIP2
CYGB
CYHR1
CYLD
CYLN2
CYorf14
CYorf15A
CYorf15B
CYP11A1
CYP1B1
CYP20A1

CYP26A1
CYP27A1
CYP27C1
CYP2C8
CYP2E1
CYP2J2
CYP2R1
CYP2S1
CYP2U1
CYP4X1
CYP51A1
CYR61
CYTH1
CYTH2
CYTH3
CYTSA
CYTSB
CYYR1
D2HGDH
D4S234E
DAAM1
DAAM2
DAB2
DAB2IP
DACH1
DACT1
DACT2
DACT3
DAD1
DAD1L
DAG1
DAGLA
DAGLB
DAK
DAP
DAP3
DAPK1
DAPK3
DAPP1
DARS
DARS2
DAXX
DAZAP1
DAZAP2
DBC1
DBI
DBN1
DBNDD1
DBNDD2
DBNL

DBP
DBR1
DBT
DCAF10
DCAF15
DCAF16
DCAF6
DCAF7
DCAKD
DCBLD1
DCBLD2
DCHS1
DCI
DCK
DCLK1
DCLK3
DCLRE1A
DCLRE1B
DCLRE1C
DCN
DCP1A
DCP1B
DCP2
DCPS
DCTD
DCTN1
DCTN2
DCTN3
DCTN4
DCTN5
DCTN6
DCTPP1
DCUN1D1
DCUN1D2
DCUN1D3
DCUN1D4
DCUN1D5
DCXR
DDA1
DDAH1
DDAH2
DDB1
DDB2
DDEF2
DDHD2
DDIT3
DDIT4
DDIT4L
DDOST
DDR1

DDR2
DDRGK1
DDT
DDX1
DDX10
DDX11
DDX12
DDX17
DDX18
DDX19A
DDX19B
DDX19-DDX19
DDX21
DDX23
DDX24
DDX27
DDX28
DDX31
DDX39
DDX3X
DDX3Y
DDX41
DDX42
DDX46
DDX47
DDX49
DDX5
DDX50
DDX51
DDX52
DDX54
DDX55
DDX56
DDX58
DDX59
DEAF1
DECR1
DECR2
DEDD
DEDD2
DEF6
DEF8
DEGS1
DEK
DEM1
DENND1A
DENND2A
DENND2C
DENND4A
DENND4B

DENND4C
DENND5A
DENND5B
DENR
DEPDC1
DEPDC1B
DEPDC5
DEPDC6
DERA
DERL1
DERL2
DET1
DEXI
DFFA
DFFB
DFNA5
DFNB31
DFNB59
DGAT1
DGAT2
DGCR11
DGCR14
DGCR2
DGCR6
DGCR6L
DGCR8
DGKA
DGKD
DGKQ
DGUOK
DHCR24
DHCR7
DHDDS
DHDH
DHFRL1
DHPS
DHRS1
DHRS11
DHRS12
DHRS13
DHRS2
DHRS3
DHRS4
DHRS4L2
DHRS7
DHRS7B
DHRSX
DHTKD1
DHX15
DHX16

DHX29
DHX30
DHX32
DHX33
DHX34
DHX35
DHX36
DHX37
DHX38
DHX40
DHX57
DHX8
DHX9
DIABLO
DIAPH1
DIAPH2
DIAPH3
DICER1
DIDO1
DIMT1L
DIO2
DIO3
DIO3OS
DIP2A
DIP2B
DIP2C
DIRC2
DIS3L
DIS3L2
DISP1
DIXDC1
dJ341D10.1
DKC1
DKFZp434K19
DKFZp434N03
DKFZp451M21
DKFZP564O05
DKFZP586I142
DKFZp667M24
DKFZp686I152
DKFZp686O24
DKFZp761P04
DKK1
DKK3
DKKL1
DLAT
DLD
DLEU1
DLEU2
DLG2

DLG3
DLG4
DLG5
DLGAP4
DLGAP5
DLK1
DLK2
DLL1
DLL3
DLST
DLX3
DLX5
DMAP1
DMC1
DMD
DMKN
DMRTA1
DMTF1
DMWD
DMXL1
DNA2
DNAH1
DNAH14
DNAH2
DNAJA1
DNAJA2
DNAJA3
DNAJA4
DNAJB1
DNAJB11
DNAJB12
DNAJB14
DNAJB2
DNAJB5
DNAJB6
DNAJB9
DNAJC1
DNAJC10
DNAJC13
DNAJC14
DNAJC15
DNAJC17
DNAJC18
DNAJC19
DNAJC2
DNAJC21
DNAJC24
DNAJC25
DNAJC27
DNAJC3

DNAJC30
DNAJC4
DNAJC5
DNAJC7
DNAJC8
DNAJC9
DNAL1
DNAL4
DNALI1
DNASE1
DNASE1L1
DNASE2
DNCL1
DNLZ
DNM1L
DNM2
DNMT1
DNMT3B
DNPEP
DNTTIP1
DNTTIP2
DOCK1
DOCK11
DOCK2
DOCK3
DOCK6
DOCK7
DOCK8
DOHH
DOK4
DOLK
DOLPP1
DOM3Z
DONSON
DOPEY1
DOPEY2
DOT1L
DPAGT1
DPF2
DPH2
DPH3
DPH5
DPM1
DPM2
DPM3
DPP10
DPP3
DPP4
DPP7
DPP8

DPP9
DPPA2
DPPA3
DPPA4
DPY19L1
DPY19L4
DPY30
DPYSL2
DPYSL3
DPYSL4
DR1
DRAM1
DRAP1
DRD1IP
DRD4
DRG1
DRG2
DSC2
DSC3
DSCAM
DSCC1
DSCR10
DSCR1L1
DSCR3
DSCR6
DSE
DSEL
DSG2
DSN1
DSP
DSTN
DSTYK
DTD1
DTL
DTNA
DTNB
DTNBP1
DTWD1
DTWD2
DTX2
DTX3
DULLARD
DUS1L
DUS2L
DUS3L
DUS4L
DUSP1
DUSP10
DUSP11
DUSP12

DUSP13
DUSP14
DUSP15
DUSP16
DUSP18
DUSP19
DUSP2
DUSP22
DUSP23
DUSP28
DUSP3
DUSP5
DUSP6
DUSP8
DUT
DUXAP3
DVL1
DVL2
DVL3
DYDC2
DYM
DYNC1H1
DYNC1I1
DYNC1I2
DYNC1LI2
DYNC2H1
DYNC2LI1
DYNLL1
DYNLL2
DYNLRB1
DYNLT1
DYNLT3
DYRK1A
DYRK1B
DYRK2
DYRK4
DYSF
DZIP1L
E2F1
E2F2
E2F3
E2F4
E2F5
E2F6
E2F7
E4F1
EAF1
EAF2
EAPP
EARS2

EBAG9
EBF4
EBNA1BP2
EBP
EBPL
ECD
ECE2
ECGF1
ECH1
ECHDC1
ECHDC2
ECHDC3
ECHS1
ECOP
ECSIT
ECT2
EDARADD
EDC3
EDC4
EDEM1
EDEM2
EDEM3
EDF1
EDG1
EDG4
EDN1
EDNRA
EDNRB
EEA1
EED
EEF1A1
EEF1A2
EEF1AL7
EEF1B2
EEF1D
EEF1E1
EEF1G
EEF2
EEF2K
EFCAB4A
EFCAB7
EFEMP1
EFEMP2
EFHA1
EFHB
EFHC1
EFHD1
EFHD2
EFNA1
EFNA4

EFNB1
EFNB2
EFNB3
EFR3A
EFR3B
EFS
EFTUD1
EFTUD2
EGFL7
EGFLAM
EGFR
EGLN1
EGLN2
EGLN3
EGOT
EGR1
EHBP1
EHBP1L1
EHD1
EHD2
EHD3
EHD4
EHMT1
EHMT2
EI24
EID1
EID2
EID2B
EIF1
EIF1AD
EIF1AX
EIF1AY
EIF1B
EIF2A
EIF2AK1
EIF2AK2
EIF2AK3
EIF2AK4
EIF2B1
EIF2B2
EIF2B3
EIF2B4
EIF2B5
EIF2C1
EIF2C2
EIF2C3
EIF2C4
EIF2S1
EIF2S2
EIF2S3

EIF3A
EIF3B
EIF3C
EIF3D
EIF3E
EIF3F
EIF3G
EIF3H
EIF3I
EIF3J
EIF3K
EIF3L
EIF3M
EIF4A1
EIF4A2
EIF4A3
EIF4B
EIF4E
EIF4E2
EIF4E3
EIF4EBP1
EIF4EBP2
EIF4EBP3
EIF4ENIF1
EIF4G1
EIF4G2
EIF4G3
EIF4H
EIF5
EIF5A
EIF5A2
EIF5B
EIF6
ELAC2
ELAVL1
ELF1
ELF2
ELF3
ELF4
ELK1
ELK4
ELL
ELL2
ELL3
ELMO1
ELMO2
ELMO3
ELMOD2
ELMOD3
ELOF1

ELOVL1
ELOVL4
ELOVL5
ELOVL6
ELP2
ELP3
ELP4
EMD
EME1
EMG1
EMID1
EMID2
EMILIN2
EML1
EML2
EML3
EML4
EMP1
EMP3
EMX2OS
ENAH
ENC1
ENDOD1
ENDOG
ENG
ENHO
ENO1
ENO2
ENO3
ENOPH1
ENOSF1
ENOX2
ENPEP
ENPP1
ENPP2
ENPP4
ENPP5
ENSA
ENTPD3
ENTPD4
ENTPD5
ENTPD6
ENTPD7
ENY2
EOMES
EP300
EP400
EPAS1
EPB41L1
EPB41L2

EPB41L3
EPB41L4A
EPB41L4B
EPB41L5
EPB49
EPC2
EPCAM
EPDR1
EPHA1
EPHA10
EPHA2
EPHA4
EPHA8
EPHB1
EPHB2
EPHB3
EPHB4
EPHB6
EPHX1
EPHX2
EPM2A
EPM2AIP1
EPN1
EPN2
EPOR
EPPB9
EPR1
EPRS
EPS15
EPS15L1
EPS8
EPS8L1
EPSTI1
ERAL1
ERBB2
ERBB3
ERC1
ERC2
ERCC1
ERCC2
ERCC3
ERCC5
ERCC6L
ERCC8
ERF
ERGIC1
ERGIC2
ERGIC3
ERH
ERI1

ERI2
ERI3
ERICH1
ERLIN1
ERLIN2
ERMAP
ERMP1
ERN1
ERO1L
ERP27
ERP29
ERRFI1
ERVWE1
ESAM
ESCO1
ESD
ESPL1
ESPN
ESRRA
ESRRAP2
ESRRG
ESYT1
ETF1
ETFA
ETFB
ETFDH
ETNK1
ETNK2
ETS1
ETS2
ETV3
ETV4
ETV5
ETV6
EVC
EVI2A
EVI5
EVI5L
EVL
EWSR1
EXD2
EXO1
EXOC1
EXOC2
EXOC3
EXOC4
EXOC5
EXOC6
EXOC7
EXOSC1

EXOSC10
EXOSC2
EXOSC3
EXOSC4
EXOSC5
EXOSC6
EXOSC7
EXOSC8
EXOSC9
EXPH5
EXT1
EXT2
EXTL2
EXTL3
EYA1
EYA2
EYA3
EYS
EZH2
EZR
F10
F11R
F12
F2R
F2RL1
F3
F8A1
FAAH
FAAH2
FABP5
FABP5L2
FABP7
FADD
FADS1
FADS2
FADS3
FAF1
FAF2
FAH
FAHD1
FAHD2A
FAHD2B
FAIM
FAM100A
FAM100B
FAM102A
FAM102B
FAM103A1
FAM104A
FAM104B

FAM105A
FAM105B
FAM107B
FAM108A2
FAM108A3
FAM108B1
FAM108C1
FAM109A
FAM10A4
FAM10A7
FAM110A
FAM110B
FAM111A
FAM113A
FAM113B
FAM114A1
FAM114A2
FAM115A
FAM116A
FAM116B
FAM117A
FAM117B
FAM118A
FAM118B
FAM119A
FAM119B
FAM120A
FAM120AOS
FAM120B
FAM122A
FAM122B
FAM123A
FAM125A
FAM125B
FAM126A
FAM126B
FAM127A
FAM127B
FAM127C
FAM128A
FAM129A
FAM129B
FAM131A
FAM133B
FAM134A
FAM134B
FAM134C
FAM135A
FAM136A
FAM136B

FAM13A
FAM13B
FAM149A
FAM149B1
FAM14B
FAM153B
FAM156A
FAM156B
FAM158A
FAM160A2
FAM160B1
FAM160B2
FAM161A
FAM162A
FAM162B
FAM164A
FAM164C
FAM165B
FAM168B
FAM171A1
FAM172A
FAM173A
FAM173B
FAM174A
FAM174B
FAM175A
FAM175B
FAM176B
FAM177A1
FAM178A
FAM179B
FAM181B
FAM184A
FAM188A
FAM189B
FAM18B
FAM18B2
FAM190B
FAM193A
FAM193B
FAM195A
FAM195B
FAM19A4
FAM20A
FAM20B
FAM20C
FAM21D
FAM24B
FAM26E
FAM30A

FAM32A
FAM35A
FAM36A
FAM38A
FAM38B
FAM39DP
FAM39E
FAM3A
FAM3B
FAM3C
FAM40A
FAM40B
FAM43A
FAM44B
FAM45A
FAM45B
FAM46A
FAM46B
FAM46C
FAM48A
FAM49A
FAM49B
FAM50A
FAM53B
FAM53C
FAM54A
FAM54B
FAM57A
FAM58A
FAM59A
FAM60A
FAM62B
FAM63A
FAM63B
FAM64A
FAM65A
FAM65B
FAM69A
FAM69B
FAM70A
FAM70B
FAM71E1
FAM72A
FAM72B
FAM72D
FAM73A
FAM73B
FAM75B
FAM75C1
FAM76B

FAM80B
FAM82A2
FAM82B
FAM83B
FAM83D
FAM83F
FAM83H
FAM84B
FAM86A
FAM86B1
FAM86C
FAM86D
FAM89A
FAM89B
FAM8A1
FAM90A2P
FAM90A3
FAM92A1
FAM96A
FAM96B
FAM98A
FAM98C
FANCB
FANCC
FANCD2
FANCE
FANCG
FANCI
FANCL
FAR1
FAR2
FARP1
FARS2
FARSA
FARSLB
FASN
FASTK
FASTKD1
FASTKD2
FASTKD3
FASTKD5
FAT1
FAT3
FAU
FBL
FBLIM1
FBLN1
FBLN2
FBLN5
FBN2

FBN3
FBP1
FBRS
FBS1
FBXL10
FBXL11
FBXL12
FBXL14
FBXL15
FBXL16
FBXL18
FBXL2
FBXL20
FBXL5
FBXL6
FBXL7
FBXO10
FBXO11
FBXO15
FBXO16
FBXO17
FBXO18
FBXO2
FBXO21
FBXO22
FBXO28
FBXO3
FBXO30
FBXO31
FBXO32
FBXO33
FBXO34
FBXO36
FBXO38
FBXO42
FBXO44
FBXO45
FBXO46
FBXO5
FBXO6
FBXO7
FBXO8
FBXW11
FBXW2
FBXW4
FBXW5
FBXW7
FBXW8
FBXW9
FCAR

FCF1
FCGR2B
FCGR3A
FCGR3B
FCGRT
FCHO2
FCHSD2
FDFT1
FDPS
FDX1
FDX1L
FDXR
FECH
FEM1A
FEM1B
FEM1C
FEN1
FER
FER1L3
FER1L4
FERMT1
FERMT2
FES
FEZ1
FEZ2
FGA
FGD1
FGD3
FGD5
FGD6
FGF11
FGF13
FGF18
FGF2
FGF23
FGFR1OP
FGFR1OP2
FGFR3
FGFR4
FGFRL1
FGGY
FGL1
FGL2
FGR
FH
FHDC1
FHIT
FHL1
FHL2
FHL3

FHOD1
FHOD3
FIBCD1
FIBP
FICD
FIG4
FIGNL1
FIGNL2
FILIP1
FILIP1L
FIP1L1
FIS1
FIZ1
FJX1
FKBP10
FKBP11
FKBP14
FKBP15
FKBP1A
FKBP1B
FKBP1P1
FKBP2
FKBP3
FKBP4
FKBP5
FKBP8
FKBP9L
FKBPL
FKRP
FKSG30
FKTN
FLAD1
FLII
FLJ10081
FLJ10213
FLJ10357
FLJ10374
FLJ10781
FLJ10916
FLJ10986
FLJ10996
FLJ11235
FLJ11783
FLJ12078
FLJ12355
FLJ12684
FLJ12949
FLJ13305
FLJ14107
FLJ14166

FLJ14213
FLJ14712
FLJ20021
FLJ20125
FLJ20209
FLJ20254
FLJ20273
FLJ20444
FLJ20489
FLJ20628
FLJ20674
FLJ20699
FLJ20718
FLJ20850
FLJ20920
FLJ21865
FLJ22184
FLJ22222
FLJ22531
FLJ22536
FLJ22662
FLJ22795
FLJ23584
FLJ25006
FLJ25363
FLJ30092
FLJ30428
FLJ31306
FLJ31568
FLJ32011
FLJ32810
FLJ33996
FLJ34047
FLJ35024
FLJ35220
FLJ35390
FLJ35767
FLJ35934
FLJ36070
FLJ36131
FLJ37396
FLJ37453
FLJ38482
FLJ38717
FLJ38973
FLJ39653
FLJ39660
FLJ39827
FLJ40113
FLJ40504

FLJ40722
FLJ40852
FLJ42258
FLJ42627
FLJ42957
FLJ43681
FLJ44054
FLJ44124
FLJ44342
FLJ45032
FLJ45202
FLJ45244
FLJ45256
FLJ45337
FLJ46309
FLJ46552
FLJ46906
FLJ90086
FLJ90757
FLNA
FLNB
FLNC
FLOT1
FLOT2
FLRT2
FLRT3
FLVCR1
FLVCR2
FLYWCH1
FLYWCH2
FMNL1
FMNL2
FMO4
FMO5
FMO6P
FMOD
FN3KRP
FNBP1
FNBP1L
FNBP4
FNDC3A
FNDC3B
FNDC4
FNDC5
FNIP1
FNIP2
FNTA
FNTB
FOLR1
FOS

FOSL1
FOSL2
FOXA1
FOXA2
FOXA3
FOXC1
FOXC2
FOXD1
FOXD4L4
FOXH1
FOXJ2
FOXJ3
FOXK1
FOXK2
FOXM1
FOXN2
FOXO1
FOXO3
FOXO4
FOXP1
FOXP4
FOXQ1
FOXRED1
FOXRED2
FRAG1
FRAP1
FRAS1
FRAT2
FREM1
FREM2
FREQ
FRG1
FRMD4A
FRMD6
FRMD8
FRMPD3
FRRS1
FRS3
FRYL
FRZB
FSBP
FSCN1
FSD1
FST
FSTL1
FSTL3
FTH1
FTHL11
FTHL12
FTHL16

FTHL2
FTHL3
FTHL7
FTHL8
FTL
FTO
FTSJ1
FTSJ2
FTSJ3
FTSJD1
FTSJD2
FUBP1
FUBP3
FUCA1
FUCA2
FUK
FUNDC1
FURIN
FUS
FUT1
FUT10
FUT11
FUT4
FUT6
FUT8
FUZ
FVT1
FXC1
FXR1
FXR2
FXYD1
FXYD5
FXYD6
FYCO1
FYN
FYTTD1
FZD1
FZD2
FZD3
FZD4
FZD5
FZD6
FZD7
FZD8
FZR1
G3BP1
G3BP2
G6PC3
G6PD
GAA

GAB1
GAB2
GABARAP
GABARAPL1
GABARAPL2
GABBR1
GABPA
GABPB1
GABPB2
GABRB3
GABRE
GABRG2
GABRP
GAD1
GADD45A
GADD45B
GADD45G
GADD45GIP1
GAK
GAL
GAL3ST1
GAL3ST4
GALC
GALE
GALK1
GALK2
GALM
GALNS
GALNT1
GALNT10
GALNT11
GALNT12
GALNT2
GALNT3
GALNT4
GALNT6
GALNTL1
GALNTL4
GALR2
GALT
GAMT
GAN
GANAB
GAPDH
GAPDHL6
GAPVD1
GAR1
GARNL4
GARS
GART

GAS1
GAS2L3
GAS6
GAS7
GAS8
GATA2
GATA3
GATA4
GATA6
GATAD1
GATAD2A
GATAD2B
GATC
GATM
GATS
GBA
GBA2
GBAS
GBE1
GBF1
GBGT1
GBP4
GCA
GCAT
GCC1
GCC2
GCDH
GCET2
GCGR
GCH1
GCHFR
GCLC
GCLM
GCN1L1
GCNT1
GCNT2
GCSH
GDAP2
GDE1
GDF11
GDF15
GDF3
GDF5OS
GDI1
GDI2
GDPD1
GDPD3
GDPD5
GEMIN4
GEMIN5

GEMIN6
GEMIN8
GEN1
GFER
GFM1
GFM2
GFOD1
GFOD2
GFPT1
GFPT2
GFRA2
GGA1
GGA2
GGA3
GGCT
GGCX
GGH
GGNBP2
GGPS1
GGTL3
GGTLC1
GHITM
GHR
GINS2
GINS3
GINS4
GIPC1
GIT1
GIT2
GIYD1
GIYD2
GJA1
GJC1
GJC2
GJC3
GK5
GKAP1
GLA
GLB1
GLB1L
GLB1L2
GLCE
GLDC
GLDN
GLE1
GLG1
GLI3
GLI4
GLIPR2
GLIS3

GLMN
GLO1
GLOD4
GLRX
GLRX2
GLRX3
GLRX5
GLS
GLS2
GLT25D1
GLT8D1
GLT8D2
GLTP
GLTPD1
GLTSCR1
GLTSCR2
GLUD1
GLYCTK
gm127
GM2A
GMCL1
GMDS
GMEB1
GMEB2
GMFB
GMIP
GMNN
GMPPA
GMPPB
GMPR
GMPR2
GMPS
GNA11
GNA12
GNA13
GNAI1
GNAI2
GNAI3
GNAS
GNB1
GNB1L
GNB2
GNB2L1
GNB4
GNB5
GNE
GNG10
GNG11
GNG2
GNG4

GNG5
GNG7
GNG8
GNGT1
GNL1
GNL2
GNL3
GNL3L
GNPAT
GNPDA1
GNPDA2
GNPNAT1
GNPTAB
GNPTG
GNRH1
GNS
GOLGA1
GOLGA2
GOLGA3
GOLGA4
GOLGA5
GOLGA6B
GOLGA7
GOLGA8A
GOLGA8B
GOLGA9P
GOLGB1
GOLIM4
GOLM1
GOLPH3
GOLPH3L
GOLPH4
GOLT1A
GOLT1B
GON4L
GOPC
GORASP1
GORASP2
GOSR1
GOSR2
GOT1
GOT2
GP1BA
GPA33
GPAA1
GPAM
GPATCH1
GPATCH2
GPATCH3
GPATCH4

GPATCH8
GPBP1
GPBP1L1
GPC1
GPC2
GPC3
GPC4
GPC6
GPD1L
GPER
GPHN
GPI
GPKOW
GPM6A
GPM6B
GPN1
GPN2
GPN3
GPR1
GPR108
GPR114
GPR125
GPR126
GPR128
GPR137
GPR137B
GPR137C
GPR143
GPR153
GPR160
GPR161
GPR162
GPR172A
GPR175
GPR177
GPR180
GPR19
GPR3
GPR37
GPR56
GPR64
GPR84
GPR89A
GPR89B
GPR89C
GPR98
GPRASP2
GPRC5B
GPRC5C
GPS1

GPS2
GPSM1
GPSM2
GPSM3
GPT2
GPX1
GPX2
GPX3
GPX4
GPX7
GPX8
GRAMD1A
GRAMD1B
GRAMD3
GRAMD4
GRB10
GRB14
GRB2
GRB7
GREB1
GREM2
GRHL2
GRHPR
GRIN3B
GRINA
GRIP2
GRIPAP1
GRK5
GRK6
GRM2
GRN
GRPEL1
GRPEL2
GRPR
GRSF1
GRTP1
GRWD1
GSDMB
GSDMD
GSG2
GSK3B
GSN
GSPT1
GSPT2
GSR
GSS
GSTA4
GSTCD
GSTK1
GSTM1

GSTM2
GSTM4
GSTO1
GSTO2
GSTP1
GSTT1
GSTTP2
GSTZ1
GTDC1
GTF2A1
GTF2A2
GTF2B
GTF2E1
GTF2E2
GTF2F1
GTF2F2
GTF2H1
GTF2H2B
GTF2H3
GTF2H4
GTF2H5
GTF2I
GTF2IP1
GTF2IRD1
GTF2IRD2B
GTF3A
GTF3C1
GTF3C2
GTF3C3
GTF3C5
GTF3C6
GTPBP1
GTPBP10
GTPBP2
GTPBP3
GTPBP4
GTPBP6
GTPBP8
GTSE1
GUCA1A
GUCA1B
GUCY1A3
GUF1
GUK1
GUSB
GUSBL1
GUSBL2
GXYLT1
GYG1
GYG2

GYLTL1B
GYPC
GYS1
H19
H1F0
H1FX
H2AFJ
H2AFV
H2AFX
H2AFY
H2AFY2
H2AFZ
H3F3A
H3F3B
H6PD
HABP2
HABP4
HACE1
HACL1
HADH
HADH2
HADHA
HADHB
HAGH
HAGHL
HAND1
HAND2
HAPLN1
HAPLN2
HAPLN4
HARBI1
HARS
HARS2
HAS2
HAS2AS
HAS3
HAT1
HAUS4
HAUS5
HAUS6
HAUS8
HAVCR2
HAX1
HBA2
HBEGF
HBG2
HBP1
HBQ1
HBXIP
HCCA2

HCCS
HCFC1
HCFC1R1
HCFC2
HCG2P7
HCG4
HCN3
HCP5
HCST
HDAC1
HDAC11
HDAC2
HDAC3
HDAC4
HDAC6
HDAC7
HDAC7A
HDAC9
HDDC2
HDDC3
HDGF
HDGF2
HDGFRP3
HDHD1A
HDHD2
HDHD3
HEATR1
HEATR2
HEATR3
HEATR5A
HEATR5B
HEATR6
HEBP1
HEBP2
HECA
HECTD1
HECTD2
HECTD3
HECW2
HEG1
HELLS
HELQ
HELZ
HEPH
HERC1
HERC2
HERC4
HERC5
HERC6
HERPUD1

HERPUD2
HES1
HES4
HES6
HESX1
HEXA
HEXB
HEXDC
HEXIM2
HEY1
HEY2
HGS
HHATL
HHLA3
HIAT1
HIATL1
HIATL2
HIBADH
HIBCH
HIC2
HIF1A
HIF1AN
HIGD1A
HIGD2A
HINFP
HINT1
HINT2
HINT3
HIP1
HIP2
HIPK2
HIRIP3
HISPPD2A
HIST1H1A
HIST1H1C
HIST1H2BD
HIST1H2BH
HIST1H2BK
HIST1H3E
HIST1H3F
HIST1H4C
HIST1H4E
HIST1H4K
HIST2H2AA3
HIST2H2AA4
HIST2H2AC
HIST2H2BE
HIST2H4A
HIST2H4B
HIST3H2A

HIT-40
HIVEP1
HIVEP2
HJURP
HK1
HK2
HKR1
HLA-A
HLA-A29.1
HLA-B
HLA-C
HLA-DMA
HLA-DOA
HLA-DRA
HLA-DRB3
HLA-DRB4
HLA-DRB6
HLA-E
HLA-F
HLA-G
HLA-H
HLCS
HLTF
HLX
HM13
HMBOX1
HMBS
HMG20A
HMG20B
HMGA1
HMGB1
HMGB1L1
HMGB2
HMGB3
HMGCL
HMGCR
HMGCS1
HMGN1
HMGN2
HMGN3
HMGN4
HMGXB4
HMHA1
HMMR
HMOX1
HMOX2
HN1
HN1L
HNF1B
HNMT

HNRNPA0
HNRNPA1
HNRNPA1L2
HNRNPA2B1
HNRNPA3
HNRNPA3P1
HNRNPAB
HNRNPC
HNRNPD
HNRNPF
HNRNPH1
HNRNPH2
HNRNPH3
HNRNPK
HNRNPL
HNRNPM
HNRNPR
HNRNPU
HNRNPUL1
HNRNPUL2
HNRPA1L-2
HNRPA1P4
HNRPA2B1
HNRPC
HNRPDL
HNRPH1
HNRPH3
HNRPK
HNRPLL
HNRPM
HNRPR
HNRPUL1
HNRPUL2
HOMER1
HOMER2
HOMER3
HOM-TES-103
HOOK1
HOOK2
HOOK3
HOPX
HOXA6
HOXB2
HOXB4
HOXB5
HOXB6
HOXB7
HOXB8
HOXC13
HOXC6

HOXC8
HPCAL1
HPGD
HPN
HPRT1
HPS1
HPS3
HPS4
HPS5
HPS6
HPSE
HRAS
HRASLS
HRASLS3
HRB
HRH1
HRK
HRSP12
HS1BP3
HS2ST1
HS3ST1
HS3ST3A1
HS6ST2
HSBP1
HSCB
HSD11B1L
HSD17B10
HSD17B11
HSD17B12
HSD17B14
HSD17B4
HSD17B7
HSD17B8
HSDL1
HSDL2
HSF1
HSF2
HSGT1
HSP90AA1
HSP90AB1
HSP90B1
HSPA12A
HSPA13
HSPA14
HSPA1A
HSPA1B
HSPA2
HSPA4
HSPA4L
HSPA5

HSPA7
HSPA8
HSPA9
HSPB1
HSPB8
HSPBAP1
HSPBL2
HSPBP1
HSPC111
HSPC157
HSPC159
HSPC171
HSPC268
HSPCAL3
HSPD1
HSPE1
HSPH1
HSZFP36
HTATIP2
HTR1E
HTR2C
HTRA1
HTRA2
HTT
HUS1B
HUWE1
HVCN1
HYAL1
HYAL2
HYAL3
HYDIN
HYI
HYLS1
HYOU1
IAH1
IARS
IARS2
IBTK
ICA1
ICAM3
ICK
ICMT
ICOSLG
ICT1
ID1
ID2
ID2B
ID3
IDE
IDH1

IDH2
IDH3A
IDH3B
IDH3G
IDI1
IDO1
IDO2
IDS
IDUA
IER2
IER3
IER3IP1
IER5
IER5L
IFFO1
IFFO2
IFI16
IFI27
IFI27L1
IFI27L2
IFI35
IFI6
IFIT1
IFITM1
IFITM2
IFITM3
IFITM4P
IFNAR1
IFNAR2
IFNGR1
IFNGR2
IFP38
IFRD1
IFRD2
IFT122
IFT140
IFT172
IFT20
IFT52
IFT74
IFT80
IFT88
IGBP1
IGDCC3
IGDCC4
IGF2AS
IGF2BP1
IGF2BP2
IGF2BP3
IGF2R

IGFBP2
IGFBP3
IGFBP4
IGFBP5
IGFBP6
IGFBP7
IGFL3
IGHMBP2
IGSF1
IGSF11
IGSF3
IGSF5
IGSF8
IGSF9
IHH
IHPK3
IK
IKBIP
IKBKB
IKBKE
IKBKG
IKZF2
IKZF3
IKZF4
IKZF5
IL10
IL10RB
IL11
IL11RA
IL13RA1
IL16
IL17D
IL17RA
IL17RB
IL17RC
IL17RD
IL18
IL18BP
IL1RL1
IL20RB
IL24
IL25
IL27RA
IL28RA
IL4R
IL5RA
IL8
ILDR1
ILF2
ILF3

ILK
ILKAP
ILVBL
IMAA
IMMP2L
IMMT
IMP3
IMP4
IMPA1
IMPA2
IMPACT
IMPAD1
IMPDH1
IMPDH2
INA
INADL
INCA1
INCENP
INF2
ING1
ING2
ING3
ING4
INHBE
INMT
INO80
INO80B
INO80C
INO80D
INO80E
INPP1
INPP4A
INPP5A
INPP5B
INPP5D
INPP5E
INPP5F
INPP5K
INPPL1
INSC
INSIG1
INSIG2
INS-IGF2
INSL5
INSM1
INTS1
INTS10
INTS12
INTS2
INTS3

INTS4
INTS5
INTS6
INTS7
INTS8
INTS9
INTU
INVS
IP6K1
IP6K2
IP6K3
IPO11
IPO13
IPO4
IPO5
IPO7
IPO8
IPO9
IPP
IPPK
IPW
IQCB1
IQCC
IQCG
IQCK
IQGAP1
IQGAP2
IQGAP3
IQSEC1
IQSEC2
IRAK1
IRAK1BP1
IRAK2
IRAK4
IREB2
IRF1
IRF2BP1
IRF2BP2
IRF3
IRF4
IRF6
IRF7
IRF9
IRS1
IRS2
IRX1
IRX2
IRX3
IRX4
IRX5

IRX6
ISCA1
ISCA1L
ISCA2
ISCU
ISG15
ISG20
ISG20L1
ISG20L2
ISL1
ISOC1
ISOC2
ISY1
ISYNA1
ITCH
ITFG1
ITFG2
ITFG3
ITGA11
ITGA2
ITGA3
ITGA4
ITGA5
ITGA6
ITGA9
ITGAE
ITGAV
ITGB1
ITGB1BP1
ITGB1BP3
ITGB2
ITGB3BP
ITGB4
ITGB4BP
ITGB5
ITIH5
ITLN2
ITM2A
ITM2B
ITM2C
ITPA
ITPK1
ITPKB
ITPKC
ITPR2
ITPR3
ITPRIP
ITPRIPL2
ITSN1
IVD

IVNS1ABP
IWS1
JAG1
JAG2
JAGN1
JAK1
JAK2
JAM2
JAM3
JARID1A
JARID1D
JARID2
JAZF1
JDP2
JHDM1D
JMJD1A
JMJD1C
JMJD2B
JMJD2C
JMJD4
JMJD5
JMJD6
JMJD8
JMY
JOSD1
JOSD2
JPH1
JPH2
JPH3
JTB
JUB
JUN
JUND
JUP
KAL1
KANK1
KANK2
KANK3
KANK4
KARS
KAT2A
KAT2B
KAT5
KATNA1
KATNAL1
KATNAL2
KATNB1
KAZALD1
KBTBD11
KBTBD2

KBTBD4
KBTBD6
KBTBD7
KBTBD8
KBTBD9
KCMF1
KCNA5
KCNAB1
KCND1
KCNE1L
KCNF1
KCNG1
KCNH6
KCNH8
KCNJ10
KCNJ13
KCNJ16
KCNJ2
KCNJ4
KCNJ8
KCNK1
KCNK10
KCNK12
KCNK13
KCNK3
KCNK4
KCNK5
KCNK6
KCNMA1
KCNMB2
KCNMB3
KCNMB4
KCNN2
KCNN4
KCNQ1OT1
KCNQ2
KCNS1
KCNS3
KCNT2
KCTD1
KCTD10
KCTD11
KCTD12
KCTD13
KCTD14
KCTD15
KCTD17
KCTD2
KCTD20
KCTD21

KCTD3
KCTD5
KCTD6
KCTD7
KCTD9
KDELC1
KDELC2
KDELR1
KDELR2
KDELR3
KDM3B
KDM4D
KDM5B
KDM6B
KDR
KDSR
KEAP1
KEL
KHDC1
KHDRBS1
KHDRBS3
KHNYN
KHSRP
KIAA0020
KIAA0040
KIAA0082
KIAA0090
KIAA0100
KIAA0101
KIAA0114
KIAA0133
KIAA0141
KIAA0146
KIAA0174
KIAA0182
KIAA0194
KIAA0195
KIAA0196
KIAA0232
KIAA0240
KIAA0247
KIAA0261
KIAA0319L
KIAA0355
KIAA0363
KIAA0367
KIAA0368
KIAA0391
KIAA0406
KIAA0408

KIAA0415
KIAA0427
KIAA0430
KIAA0460
KIAA0492
KIAA0494
KIAA0495
KIAA0513
KIAA0514
KIAA0528
KIAA0556
KIAA0562
KIAA0564
KIAA0586
KIAA0649
KIAA0652
KIAA0664
KIAA0672
KIAA0753
KIAA0773
KIAA0831
KIAA0892
KIAA0895
KIAA0895L
KIAA0907
KIAA0913
KIAA0922
KIAA0947
KIAA1009
KIAA1012
KIAA1026
KIAA1033
KIAA1107
KIAA1128
KIAA1143
KIAA1147
KIAA1160
KIAA1161
KIAA1191
KIAA1211
KIAA1217
KIAA1244
KIAA1267
KIAA1274
KIAA1279
KIAA1285
KIAA1310
KIAA1324L
KIAA1328
KIAA1333

KIAA1370
KIAA1383
KIAA1429
KIAA1430
KIAA1467
KIAA1468
KIAA1522
KIAA1524
KIAA1530
KIAA1539
KIAA1543
KIAA1545
KIAA1549
KIAA1571
KIAA1586
KIAA1598
KIAA1600
KIAA1602
KIAA1618
KIAA1632
KIAA1641
KIAA1666
KIAA1671
KIAA1688
KIAA1704
KIAA1712
KIAA1715
KIAA1731
KIAA1737
KIAA1751
KIAA1797
KIAA1826
KIAA1862
KIAA1949
KIAA1958
KIAA1967
KIAA1984
KIAA2010
KIAA2013
KIAA2026
KIDINS220
KIF11
KIF13B
KIF14
KIF15
KIF16B
KIF18A
KIF1A
KIF1B
KIF1C

KIF20A
KIF20B
KIF21A
KIF22
KIF23
KIF24
KIF26A
KIF26B
KIF2A
KIF2C
KIF3B
KIF3C
KIF4A
KIF5B
KIF5C
KIF7
KIFAP3
KIFC1
KIFC2
KIT
KITLG
KLB
KLC1
KLC2
KLC4
KLF11
KLF12
KLF13
KLF15
KLF2
KLF4
KLF5
KLF6
KLF8
KLF9
KLHDC2
KLHDC3
KLHDC4
KLHDC5
KLHDC8B
KLHDC9
KLHL12
KLHL13
KLHL17
KLHL18
KLHL2
KLHL20
KLHL21
KLHL22
KLHL23

KLHL24
KLHL26
KLHL28
KLHL29
KLHL3
KLHL36
KLHL5
KLHL7
KLHL8
KLHL9
KLK6
KLRA1
KLRAQ1
KLRG1
KLRG2
KNTC1
KPNA1
KPNA2
KPNA3
KPNA4
KPNA6
KPNB1
KPTN
KRAS
KRBA1
KRCC1
KREMEN1
KREMEN2
KRI1
KRIT1
KRT10
KRT17P3
KRT18
KRT18P13
KRT18P17
KRT18P19
KRT18P26
KRT18P28
KRT18P30
KRT18P42
KRT19
KRT8
KRT8P9
KRTAP10-11
KRTAP10-2
KRTAP21-1
KRTAP6-3
KRTCAP2
KRTCAP3
KRTDAP

KSR2
KTELC1
KTI12
KTN1
L1CAM
L1TD1
L2HGDH
L3MBTL
L3MBTL2
L3MBTL3
LACTB
LACTB2
LAD1
LAGE3
LAIR1
LAMA1
LAMA2
LAMA5
LAMB1
LAMB2
LAMB2L
LAMC1
LAMC2
LAMC3
LAMP1
LAMP2
LAMP3
LANCL1
LANCL2
LAP3
LAPTM4A
LAPTM4B
LARGE
LARP1
LARP1B
LARP4
LARP4B
LARP6
LARP7
LARS
LARS2
LAS1L
LASP1
LASS1
LASS2
LASS4
LASS5
LASS6
LAT1-3TM
LATS2

LAYN
LBA1
LBH
LBR
LBX2
LCLAT1
LCMT1
LCMT2
LCN15
LCN1L1
LCOR
LCP1
LDB2
LDHA
LDHB
LDLR
LDLRAD3
LDLRAP1
LDOC1
LDOC1L
LEAP2
LECT1
LEF1
LEFTY2
LEMD1
LEMD2
LEMD3
LENG1
LEO1
LEP
LEPR
LEPRE1
LEPREL1
LEPREL2
LEPROT
LEPROTL1
LETM1
LETMD1
LFNG
LGALS1
LGALS2
LGALS3
LGALS3BP
LGALS8
LGI2
LGMN
LGR6
LGTN
LHFP
LHFPL2

LHPP
LIAS
LIFR
LIG1
LIG3
LILRB1
LILRB3
LIMA1
LIMCH1
LIME1
LIMK1
LIMK2
LIMS1
LIMS2
LIN28
LIN28B
LIN37
LIN52
LIN54
LIN7A
LIN7B
LIN7C
LIN9
LINGO1
LINGO2
LINS1
LIPA
LIPG
LIPT1
LITAF
LIX1
LIX1L
LLGL1
LLGL2
LLPH
LMAN1
LMAN2
LMAN2L
LMBR1
LMBRD1
LMCD1
LMF2
LMNA
LMNB1
LMNB2
LMO2
LMO4
LMOD3
LMTK2
LMTK3

LNPEP
LNX2
LOC10000858
LOC10000858
LOC10000967
LOC10012555
LOC10012789
LOC10012789
LOC10012791
LOC10012791
LOC10012792
LOC10012797
LOC10012797
LOC10012798
LOC10012798
LOC10012799
LOC10012799
LOC10012800
LOC10012801
LOC10012805
LOC10012806
LOC10012806
LOC10012808
LOC10012808
LOC10012809
LOC10012811
LOC10012812
LOC10012813
LOC10012816
LOC10012816
LOC10012819
LOC10012819
LOC10012822
LOC10012823
LOC10012825
LOC10012826
LOC10012826
LOC10012826
LOC10012827
LOC10012828
LOC10012829
LOC10012830
LOC10012832
LOC10012833
LOC10012835
LOC10012839
LOC10012839
LOC10012841
LOC10012842
LOC10012842

LOC10012844
LOC10012846
LOC10012846
LOC10012848
LOC10012849
LOC10012850
LOC10012850
LOC10012851
LOC10012852
LOC10012854
LOC10012858
LOC10012862
LOC10012867
LOC10012868
LOC10012868
LOC10012869
LOC10012873
LOC10012873
LOC10012876
LOC10012877
LOC10012877
LOC10012880
LOC10012883
LOC10012888
LOC10012888
LOC10012889
LOC10012889
LOC10012891
LOC10012893
LOC10012897
LOC10012897
LOC10012899
LOC10012902
LOC10012902
LOC10012902
LOC10012903
LOC10012905
LOC10012906
LOC10012908
LOC10012909
LOC10012909
LOC10012911
LOC10012913
LOC10012914
LOC10012914
LOC10012914
LOC10012914
LOC10012915
LOC10012919
LOC10012920

LOC10012920
LOC10012921
LOC10012923
LOC10012924
LOC10012926
LOC10012926
LOC10012929
LOC10012929
LOC10012933
LOC10012934
LOC10012936
LOC10012936
LOC10012937
LOC10012942
LOC10012942
LOC10012944
LOC10012947
LOC10012950
LOC10012951
LOC10012952
LOC10012953
LOC10012953
LOC10012954
LOC10012954
LOC10012955
LOC10012955
LOC10012955
LOC10012956
LOC10012958
LOC10012958
LOC10012959
LOC10012960
LOC10012963
LOC10012963
LOC10012964
LOC10012965
LOC10012965
LOC10012966
LOC10012967
LOC10012968
LOC10012968
LOC10012970
LOC10012971
LOC10012974
LOC10012974
LOC10012975
LOC10012975
LOC10012978
LOC10012980
LOC10012982

LOC10012986
LOC10012988
LOC10012989
LOC10012990
LOC10012990
LOC10012990
LOC10012990
LOC10012993
LOC10012995
LOC10012995
LOC10012997
LOC10012997
LOC10012997
LOC10012998
LOC10013000
LOC10013000
LOC10013005
LOC10013007
LOC10013007
LOC10013009
LOC10013012
LOC10013013
LOC10013013
LOC10013013
LOC10013015
LOC10013016
LOC10013017
LOC10013017
LOC10013019
LOC10013022
LOC10013022
LOC10013023
LOC10013026
LOC10013027
LOC10013028
LOC10013029
LOC10013029
LOC10013030
LOC10013033
LOC10013035
LOC10013036
LOC10013038
LOC10013044
LOC10013044
LOC10013045
LOC10013047
LOC10013050
LOC10013051
LOC10013051
LOC10013051

LOC10013055
LOC10013055
LOC10013055
LOC10013055
LOC10013056
LOC10013056
LOC10013059
LOC10013060
LOC10013062
LOC10013062
LOC10013063
LOC10013070
LOC10013070
LOC10013071
LOC10013074
LOC10013075
LOC10013076
LOC10013077
LOC10013077
LOC10013080
LOC10013081
LOC10013083
LOC10013083
LOC10013085
LOC10013088
LOC10013089
LOC10013090
LOC10013090
LOC10013091
LOC10013091
LOC10013093
LOC10013093
LOC10013098
LOC10013100
LOC10013107
LOC10013109
LOC10013113
LOC10013113
LOC10013116
LOC10013116
LOC10013118
LOC10013119
LOC10013120
LOC10013124
LOC10013126
LOC10013126
LOC10013129
LOC10013130
LOC10013132
LOC10013133

LOC10013134
LOC10013138
LOC10013138
LOC10013140
LOC10013145
LOC10013146
LOC10013151
LOC10013152
LOC10013153
LOC10013153
LOC10013154
LOC10013157
LOC10013160
LOC10013160
LOC10013162
LOC10013164
LOC10013167
LOC10013167
LOC10013171
LOC10013171
LOC10013172
LOC10013173
LOC10013173
LOC10013174
LOC10013176
LOC10013178
LOC10013178
LOC10013178
LOC10013180
LOC10013181
LOC10013183
LOC10013185
LOC10013185
LOC10013186
LOC10013190
LOC10013194
LOC10013196
LOC10013197
LOC10013197
LOC10013197
LOC10013198
LOC10013198
LOC10013201
LOC10013203
LOC10013203
LOC10013208
LOC10013210
LOC10013211
LOC10013211
LOC10013213

LOC10013219
LOC10013221
LOC10013222
LOC10013224
LOC10013229
LOC10013229
LOC10013230
LOC10013232
LOC10013232
LOC10013234
LOC10013235
LOC10013236
LOC10013239
LOC10013239
LOC10013241
LOC10013241
LOC10013242
LOC10013242
LOC10013243
LOC10013244
LOC10013245
LOC10013245
LOC10013247
LOC10013248
LOC10013248
LOC10013249
LOC10013249
LOC10013249
LOC10013249
LOC10013250
LOC10013251
LOC10013251
LOC10013251
LOC10013252
LOC10013253
LOC10013254
LOC10013254
LOC10013256
LOC10013258
LOC10013259
LOC10013265
LOC10013265
LOC10013265
LOC10013267
LOC10013267
LOC10013270
LOC10013271
LOC10013271
LOC10013272
LOC10013272

LOC10013272
LOC10013274
LOC10013274
LOC10013276
LOC10013276
LOC10013277
LOC10013278
LOC10013279
LOC10013279
LOC10013279
LOC10013280
LOC10013280
LOC10013282
LOC10013286
LOC10013289
LOC10013290
LOC10013291
LOC10013292
LOC10013299
LOC10013300
LOC10013301
LOC10013301
LOC10013301
LOC10013304
LOC10013305
LOC10013307
LOC10013307
LOC10013309
LOC10013312
LOC10013314
LOC10013316
LOC10013317
LOC10013318
LOC10013320
LOC10013321
LOC10013322
LOC10013322
LOC10013323
LOC10013323
LOC10013323
LOC10013326
LOC10013327
LOC10013327
LOC10013329
LOC10013332
LOC10013332
LOC10013337
LOC10013339
LOC10013343
LOC10013343

LOC10013345
LOC10013346
LOC10013347
LOC10013347
LOC10013348
LOC10013348
LOC10013350
LOC10013351
LOC10013351
LOC10013356
LOC10013357
LOC10013358
LOC10013360
LOC10013360
LOC10013360
LOC10013364
LOC10013366
LOC10013366
LOC10013367
LOC10013368
LOC10013369
LOC10013369
LOC10013373
LOC10013374
LOC10013375
LOC10013377
LOC10013377
LOC10013379
LOC10013380
LOC10013380
LOC10013381
LOC10013382
LOC10013383
LOC10013385
LOC10013387
LOC10013388
LOC10013391
LOC10013392
LOC10013393
LOC10013393
LOC10013395
LOC10013398
LOC10013399
LOC10013400
LOC10013401
LOC10013401
LOC10013405
LOC10013407
LOC10013408
LOC10013409

LOC10013410
LOC10013410
LOC10013412
LOC10013413
LOC10013414
LOC10013415
LOC10013418
LOC10013418
LOC10013418
LOC10013420
LOC10013424
LOC10013426
LOC10013427
LOC10013429
LOC10013430
LOC10013430
LOC10013430
LOC10013436
LOC10013436
LOC10013439
LOC10013440
LOC10013442
LOC10013444
LOC10013446
LOC10013446
LOC10013447
LOC10013450
LOC10013452
LOC10013453
LOC10013453
LOC10013454
LOC10013456
LOC10013458
LOC10013463
LOC10013464
LOC10013468
LOC10013471
LOC10013473
LOC10013479
LOC10013481
LOC10013486
LOC10019093
LOC10019093
LOC10019098
LOC113386
LOC119358
LOC123688
LOC124512
LOC126235
LOC127295

LOC128192
LOC130773
LOC131691
LOC132241
LOC133993
LOC134505
LOC134997
LOC136143
LOC137107
LOC143543
LOC143666
LOC144438
LOC144481
LOC145853
LOC146053
LOC146177
LOC146439
LOC146517
LOC147645
LOC147727
LOC147804
LOC148413
LOC148430
LOC148709
LOC148915
LOC149134
LOC149224
LOC149448
LOC149501
LOC150051
LOC150568
LOC151162
LOC151300
LOC151457
LOC151579
LOC152195
LOC152217
LOC153561
LOC154860
LOC158160
LOC158301
LOC158345
LOC161527
LOC163233
LOC168474
LOC169834
LOC196752
LOC199800
LOC200030
LOC201229

LOC201725
LOC202051
LOC202134
LOC202227
LOC202781
LOC203547
LOC205251
LOC220433
LOC220686
LOC221442
LOC221710
LOC23117
LOC253039
LOC255167
LOC255783
LOC257396
LOC25845
LOC283267
LOC283412
LOC283481
LOC283755
LOC283788
LOC283932
LOC283953
LOC283999
LOC284023
LOC284167
LOC284230
LOC284371
LOC284393
LOC284620
LOC284821
LOC284988
LOC285053
LOC285074
LOC285176
LOC285359
LOC285500
LOC285550
LOC285741
LOC285900
LOC285908
LOC285943
LOC286016
LOC286157
LOC286208
LOC286367
LOC286444
LOC286467
LOC286512

LOC338758
LOC338799
LOC338870
LOC339290
LOC339352
LOC339483
LOC339535
LOC339778
LOC339804
LOC339843
LOC339970
LOC340260
LOC340357
LOC340598
LOC341230
LOC341315
LOC341457
LOC341784
LOC341965
LOC342994
LOC343184
LOC344595
LOC344741
LOC345041
LOC345645
LOC346085
LOC346887
LOC346950
LOC347292
LOC347376
LOC347544
LOC374395
LOC387683
LOC387703
LOC387753
LOC387763
LOC387791
LOC387820
LOC387825
LOC387841
LOC387867
LOC387882
LOC387930
LOC387934
LOC388076
LOC388122
LOC388275
LOC388312
LOC388339
LOC388344

LOC388397
LOC388401
LOC388474
LOC388494
LOC388524
LOC388532
LOC388556
LOC388564
LOC388588
LOC388621
LOC388654
LOC388692
LOC388707
LOC388720
LOC388789
LOC388796
LOC388820
LOC388907
LOC388955
LOC388969
LOC389049
LOC389101
LOC389137
LOC389141
LOC389156
LOC389168
LOC389203
LOC389223
LOC389286
LOC389293
LOC389322
LOC389342
LOC389386
LOC389404
LOC389435
LOC389465
LOC389517
LOC389599
LOC389662
LOC389672
LOC389765
LOC389787
LOC389816
LOC389873
LOC389901
LOC390183
LOC390298
LOC390345
LOC390354
LOC390414

LOC390466
LOC390530
LOC390557
LOC390578
LOC390660
LOC390671
LOC390705
LOC390735
LOC390876
LOC390956
LOC391019
LOC391044
LOC391045
LOC391075
LOC391126
LOC391132
LOC391169
LOC391334
LOC391370
LOC391532
LOC391578
LOC391655
LOC391656
LOC391670
LOC391692
LOC391769
LOC391777
LOC391811
LOC391825
LOC391833
LOC392008
LOC392285
LOC392301
LOC392437
LOC392501
LOC392522
LOC392635
LOC392787
LOC392871
LOC399491
LOC399744
LOC399748
LOC399804
LOC399829
LOC399881
LOC399900
LOC399942
LOC399965
LOC399988
LOC400013

LOC400027
LOC400061
LOC400214
LOC400304
LOC400389
LOC400446
LOC400455
LOC400464
LOC400506
LOC400578
LOC400652
LOC400657
LOC400713
LOC400721
LOC400750
LOC400836
LOC400879
LOC400890
LOC400948
LOC400963
LOC401002
LOC401010
LOC401019
LOC401074
LOC401076
LOC401098
LOC401115
LOC401127
LOC401152
LOC401206
LOC401218
LOC401237
LOC401238
LOC401321
LOC401357
LOC401397
LOC401433
LOC401533
LOC401537
LOC401561
LOC401620
LOC401640
LOC401648
LOC401650
LOC401676
LOC401677
LOC401717
LOC401720
LOC401817
LOC401847

LOC402057
LOC402112
LOC402175
LOC402221
LOC402251
LOC402342
LOC402509
LOC402562
LOC402644
LOC402677
LOC402694
LOC404266
LOC407835
LOC439949
LOC439950
LOC439953
LOC439992
LOC439994
LOC440027
LOC440043
LOC440055
LOC440063
LOC440093
LOC440132
LOC440145
LOC440157
LOC440160
LOC440258
LOC440280
LOC440311
LOC440341
LOC440345
LOC440348
LOC440349
LOC440353
LOC440354
LOC440359
LOC440366
LOC440389
LOC440396
LOC440459
LOC440487
LOC440498
LOC440509
LOC440563
LOC440575
LOC440585
LOC440589
LOC440595
LOC440704

LOC440731
LOC440733
LOC440737
LOC440748
LOC440776
LOC440864
LOC440895
LOC440905
LOC440910
LOC440926
LOC440927
LOC440928
LOC440957
LOC440991
LOC441009
LOC441013
LOC441019
LOC441034
LOC441046
LOC441050
LOC441061
LOC441066
LOC441073
LOC441087
LOC441089
LOC441114
LOC441131
LOC441150
LOC441154
LOC441155
LOC441191
LOC441237
LOC441241
LOC441246
LOC441253
LOC441268
LOC441294
LOC441377
LOC441408
LOC441426
LOC441442
LOC441453
LOC441454
LOC441455
LOC441461
LOC441481
LOC441484
LOC441506
LOC441528
LOC441550

LOC441642
LOC441743
LOC441763
LOC441775
LOC441876
LOC441896
LOC441907
LOC442057
LOC442075
LOC442153
LOC442162
LOC442180
LOC442181
LOC442232
LOC442270
LOC442442
LOC442454
LOC442582
LOC442597
LOC442727
LOC493869
LOC550112
LOC550643
LOC552889
LOC554203
LOC554206
LOC572558
LOC613037
LOC641298
LOC641367
LOC641710
LOC641727
LOC641746
LOC641750
LOC641765
LOC641768
LOC641798
LOC641802
LOC641814
LOC641820
LOC641844
LOC641848
LOC641849
LOC641972
LOC641978
LOC641992
LOC641996
LOC642017
LOC642031
LOC642033

LOC642076
LOC642082
LOC642156
LOC642197
LOC642210
LOC642236
LOC642250
LOC642255
LOC642299
LOC642333
LOC642357
LOC642361
LOC642367
LOC642393
LOC642412
LOC642443
LOC642446
LOC642449
LOC642458
LOC642468
LOC642469
LOC642489
LOC642502
LOC642513
LOC642559
LOC642567
LOC642570
LOC642585
LOC642590
LOC642636
LOC642661
LOC642678
LOC642691
LOC642726
LOC642738
LOC642741
LOC642755
LOC642771
LOC642780
LOC642784
LOC642815
LOC642817
LOC642828
LOC642852
LOC642859
LOC642892
LOC642897
LOC642909
LOC642921
LOC642934

LOC642946
LOC642947
LOC642956
LOC642975
LOC642980
LOC642981
LOC642989
LOC643007
LOC643011
LOC643015
LOC643031
LOC643035
LOC643123
LOC643159
LOC643167
LOC643176
LOC643206
LOC643220
LOC643224
LOC643233
LOC643272
LOC643284
LOC643287
LOC643293
LOC643300
LOC643308
LOC643310
LOC643313
LOC643319
LOC643336
LOC643342
LOC643357
LOC643358
LOC643384
LOC643387
LOC643401
LOC643423
LOC643431
LOC643433
LOC643438
LOC643446
LOC643451
LOC643452
LOC643507
LOC643509
LOC643531
LOC643534
LOC643624
LOC643664
LOC643665

LOC643668
LOC643778
LOC643779
LOC643790
LOC643802
LOC643856
LOC643863
LOC643870
LOC643872
LOC643873
LOC643882
LOC643894
LOC643905
LOC643918
LOC643949
LOC643960
LOC643995
LOC643997
LOC644029
LOC644033
LOC644037
LOC644039
LOC644063
LOC644075
LOC644094
LOC644101
LOC644124
LOC644128
LOC644131
LOC644132
LOC644144
LOC644150
LOC644162
LOC644172
LOC644191
LOC644214
LOC644237
LOC644250
LOC644254
LOC644256
LOC644265
LOC644276
LOC644310
LOC644315
LOC644330
LOC644338
LOC644353
LOC644360
LOC644363
LOC644380

LOC644404
LOC644422
LOC644423
LOC644464
LOC644482
LOC644511
LOC644517
LOC644563
LOC644590
LOC644591
LOC644604
LOC644615
LOC644617
LOC644619
LOC644629
LOC644634
LOC644642
LOC644670
LOC644684
LOC644739
LOC644743
LOC644745
LOC644760
LOC644761
LOC644762
LOC644774
LOC644790
LOC644799
LOC644809
LOC644816
LOC644852
LOC644860
LOC644863
LOC644869
LOC644877
LOC644879
LOC644889
LOC644907
LOC644914
LOC644919
LOC644928
LOC644931
LOC644934
LOC644935
LOC644936
LOC644937
LOC644949
LOC644950
LOC644979
LOC644988

LOC645001
LOC645015
LOC645018
LOC645058
LOC645086
LOC645094
LOC645100
LOC645138
LOC645157
LOC645166
LOC645173
LOC645174
LOC645175
LOC645176
LOC645231
LOC645233
LOC645236
LOC645251
LOC645289
LOC645296
LOC645304
LOC645317
LOC645321
LOC645332
LOC645351
LOC645362
LOC645378
LOC645381
LOC645385
LOC645387
LOC645430
LOC645431
LOC645436
LOC645452
LOC645466
LOC645489
LOC645515
LOC645550
LOC645558
LOC645566
LOC645586
LOC645605
LOC645609
LOC645630
LOC645683
LOC645688
LOC645691
LOC645693
LOC645715
LOC645726

LOC645737
LOC645762
LOC645863
LOC645895
LOC645899
LOC645904
LOC645937
LOC645968
LOC645978
LOC645979
LOC646034
LOC646043
LOC646044
LOC646067
LOC646093
LOC646100
LOC646103
LOC646123
LOC646135
LOC646144
LOC646195
LOC646197
LOC646200
LOC646214
LOC646278
LOC646294
LOC646301
LOC646316
LOC646332
LOC646347
LOC646348
LOC646350
LOC646446
LOC646463
LOC646476
LOC646483
LOC646508
LOC646527
LOC646531
LOC646547
LOC646548
LOC646567
LOC646609
LOC646630
LOC646672
LOC646674
LOC646688
LOC646723
LOC646750
LOC646753

LOC646766
LOC646769
LOC646783
LOC646784
LOC646785
LOC646786
LOC646791
LOC646808
LOC646817
LOC646819
LOC646821
LOC646836
LOC646841
LOC646849
LOC646897
LOC646900
LOC646909
LOC646916
LOC646936
LOC646942
LOC646949
LOC646956
LOC646966
LOC646993
LOC646996
LOC647000
LOC647009
LOC647012
LOC647030
LOC647037
LOC647074
LOC647081
LOC647086
LOC647099
LOC647104
LOC647150
LOC647243
LOC647276
LOC647285
LOC647288
LOC647302
LOC647307
LOC647322
LOC647340
LOC647346
LOC647349
LOC647361
LOC647363
LOC647389
LOC647436

LOC647450
LOC647456
LOC647474
LOC647597
LOC647673
LOC647691
LOC647718
LOC647741
LOC647784
LOC647805
LOC647834
LOC647856
LOC647859
LOC647886
LOC647949
LOC647954
LOC647971
LOC648000
LOC648024
LOC648057
LOC648059
LOC648099
LOC648103
LOC648153
LOC648176
LOC648210
LOC648237
LOC648249
LOC648283
LOC648294
LOC648342
LOC648343
LOC648370
LOC648390
LOC648399
LOC648434
LOC648526
LOC648581
LOC648605
LOC648622
LOC648638
LOC648659
LOC648665
LOC648695
LOC648705
LOC648729
LOC648732
LOC648740
LOC648742
LOC648758

LOC648771
LOC648822
LOC648852
LOC648907
LOC648921
LOC648927
LOC648931
LOC648980
LOC649009
LOC649044
LOC649049
LOC649076
LOC649143
LOC649150
LOC649169
LOC649209
LOC649214
LOC649299
LOC649330
LOC649346
LOC649365
LOC649422
LOC649445
LOC649447
LOC649548
LOC649553
LOC649555
LOC649639
LOC649679
LOC649754
LOC649821
LOC649839
LOC649864
LOC649873
LOC649946
LOC649970
LOC650029
LOC650034
LOC650116
LOC650128
LOC650152
LOC650157
LOC650215
LOC650263
LOC650276
LOC650298
LOC650321
LOC650369
LOC650406
LOC650515

LOC650518
LOC650526
LOC650646
LOC650698
LOC650717
LOC650737
LOC650739
LOC650803
LOC650826
LOC650832
LOC650840
LOC650898
LOC650909
LOC651064
LOC651137
LOC651143
LOC651149
LOC651166
LOC651198
LOC651202
LOC651296
LOC651302
LOC651380
LOC651436
LOC651453
LOC651556
LOC651575
LOC651576
LOC651697
LOC651745
LOC651816
LOC651894
LOC651919
LOC652002
LOC652071
LOC652097
LOC652281
LOC652322
LOC652324
LOC652330
LOC652377
LOC652388
LOC652458
LOC652470
LOC652481
LOC652489
LOC652534
LOC652541
LOC652545
LOC652565

LOC652577
LOC652595
LOC652607
LOC652608
LOC652615
LOC652624
LOC652672
LOC652675
LOC652685
LOC652694
LOC652726
LOC652741
LOC652755
LOC652826
LOC652838
LOC652864
LOC652900
LOC652903
LOC652968
LOC652993
LOC653071
LOC653079
LOC653080
LOC653086
LOC653103
LOC653108
LOC653111
LOC653147
LOC653156
LOC653158
LOC653162
LOC653171
LOC653210
LOC653226
LOC653232
LOC653242
LOC653308
LOC653314
LOC653324
LOC653344
LOC653354
LOC653375
LOC653377
LOC653381
LOC653382
LOC653419
LOC653421
LOC653438
LOC653450
LOC653468

LOC653471
LOC653472
LOC653479
LOC653487
LOC653489
LOC653496
LOC653505
LOC653506
LOC653557
LOC653566
LOC653583
LOC653590
LOC653596
LOC653604
LOC653631
LOC653635
LOC653658
LOC653663
LOC653702
LOC653737
LOC653752
LOC653773
LOC653778
LOC653820
LOC653829
LOC653874
LOC653877
LOC653878
LOC653881
LOC653884
LOC653888
LOC653972
LOC653994
LOC654000
LOC654069
LOC654074
LOC654085
LOC654103
LOC654109
LOC654121
LOC654123
LOC654126
LOC654155
LOC654161
LOC654174
LOC654189
LOC654191
LOC654194
LOC654244
LOC654260

LOC654350
LOC678655
LOC723972
LOC727726
LOC727732
LOC727735
LOC727758
LOC727761
LOC727762
LOC727773
LOC727803
LOC727808
LOC727818
LOC727820
LOC727821
LOC727825
LOC727826
LOC727848
LOC727865
LOC727868
LOC727877
LOC727884
LOC727899
LOC727914
LOC727935
LOC727947
LOC727948
LOC727962
LOC727963
LOC727967
LOC727970
LOC727980
LOC727984
LOC727987
LOC728002
LOC728006
LOC728014
LOC728026
LOC728031
LOC728034
LOC728037
LOC728059
LOC728060
LOC728069
LOC728086
LOC728098
LOC728105
LOC728115
LOC728116
LOC728126

LOC728127
LOC728128
LOC728138
LOC728139
LOC728142
LOC728153
LOC728170
LOC728179
LOC728181
LOC728188
LOC728190
LOC728207
LOC728208
LOC728226
LOC728229
LOC728244
LOC728247
LOC728263
LOC728275
LOC728310
LOC728312
LOC728324
LOC728368
LOC728408
LOC728411
LOC728416
LOC728417
LOC728428
LOC728431
LOC728440
LOC728448
LOC728452
LOC728453
LOC728457
LOC728465
LOC728467
LOC728470
LOC728473
LOC728476
LOC728481
LOC728484
LOC728485
LOC728492
LOC728499
LOC728509
LOC728517
LOC728518
LOC728530
LOC728532
LOC728533

LOC728537
LOC728553
LOC728554
LOC728556
LOC728564
LOC728565
LOC728572
LOC728576
LOC728590
LOC728591
LOC728602
LOC728620
LOC728635
LOC728640
LOC728643
LOC728650
LOC728653
LOC728658
LOC728661
LOC728666
LOC728672
LOC728678
LOC728686
LOC728689
LOC728693
LOC728698
LOC728715
LOC728728
LOC728732
LOC728734
LOC728739
LOC728741
LOC728743
LOC728755
LOC728758
LOC728772
LOC728774
LOC728779
LOC728780
LOC728782
LOC728787
LOC728791
LOC728802
LOC728809
LOC728820
LOC728823
LOC728825
LOC728830
LOC728832
LOC728843

LOC728844
LOC728855
LOC728873
LOC728877
LOC728888
LOC728889
LOC728901
LOC728903
LOC728908
LOC728919
LOC728931
LOC728937
LOC728944
LOC728953
LOC728965
LOC728969
LOC728971
LOC728973
LOC728975
LOC728979
LOC728992
LOC729004
LOC729009
LOC729021
LOC729046
LOC729057
LOC729081
LOC729082
LOC729086
LOC729090
LOC729101
LOC729102
LOC729120
LOC729123
LOC729137
LOC729142
LOC729143
LOC729148
LOC729157
LOC729200
LOC729208
LOC729217
LOC729222
LOC729234
LOC729236
LOC729255
LOC729259
LOC729272
LOC729279
LOC729298

LOC729301
LOC729313
LOC729317
LOC729324
LOC729332
LOC729340
LOC729342
LOC729348
LOC729351
LOC729362
LOC729366
LOC729372
LOC729375
LOC729389
LOC729397
LOC729402
LOC729404
LOC729406
LOC729409
LOC729417
LOC729421
LOC729423
LOC729438
LOC729439
LOC729446
LOC729466
LOC729484
LOC729495
LOC729500
LOC729505
LOC729510
LOC729513
LOC729519
LOC729535
LOC729559
LOC729570
LOC729580
LOC729587
LOC729595
LOC729603
LOC729608
LOC729617
LOC729646
LOC729660
LOC729666
LOC729669
LOC729677
LOC729679
LOC729680
LOC729683

LOC729684
LOC729686
LOC729687
LOC729692
LOC729708
LOC729742
LOC729760
LOC729764
LOC729768
LOC729769
LOC729774
LOC729776
LOC729779
LOC729780
LOC729789
LOC729798
LOC729806
LOC729810
LOC729816
LOC729841
LOC729843
LOC729858
LOC729859
LOC729885
LOC729887
LOC729898
LOC729903
LOC729920
LOC729926
LOC729941
LOC729950
LOC729952
LOC729954
LOC729960
LOC729964
LOC729970
LOC729978
LOC729985
LOC729992
LOC730004
LOC730005
LOC730012
LOC730020
LOC730024
LOC730029
LOC730050
LOC730051
LOC730052
LOC730060
LOC730074

LOC730081
LOC730098
LOC730101
LOC730107
LOC730134
LOC730167
LOC730173
LOC730176
LOC730183
LOC730187
LOC730202
LOC730234
LOC730235
LOC730246
LOC730254
LOC730255
LOC730256
LOC730273
LOC730278
LOC730284
LOC730286
LOC730288
LOC730313
LOC730316
LOC730323
LOC730324
LOC730382
LOC730413
LOC730415
LOC730417
LOC730427
LOC730432
LOC730455
LOC730525
LOC730534
LOC730535
LOC730704
LOC730740
LOC730744
LOC730746
LOC730754
LOC730820
LOC730841
LOC730990
LOC730993
LOC730994
LOC731007
LOC731049
LOC731096
LOC731139

LOC731231
LOC731308
LOC731314
LOC731365
LOC731542
LOC731640
LOC731642
LOC731724
LOC731751
LOC731777
LOC731779
LOC731789
LOC731835
LOC731878
LOC731895
LOC731915
LOC731932
LOC731950
LOC731954
LOC731985
LOC731999
LOC732007
LOC732075
LOC732146
LOC732165
LOC732172
LOC732360
LOC732425
LOC791120
LOC81691
LOC88523
LOC90120
LOC90342
LOC90586
LOC90624
LOC91316
LOC91431
LOC91461
LOC91561
LOC91661
LOC92249
LOC92497
LOC92659
LOC92755
LOC92973
LOC93622
LOH12CR1
LONP1
LONP2
LONRF1

LOXL1
LOXL3
LPAR1
LPAR2
LPAR3
LPCAT1
LPCAT2
LPCAT3
LPCAT4
LPGAT1
LPHN1
LPHN2
LPHN3
LPIN1
LPIN2
LPP
LPPR3
LPXN
LQK1
LRAP
LRBA
LRCH2
LRCH3
LRCH4
LRDD
LRFN3
LRFN4
LRFN5
LRG1
LRIG1
LRIG2
LRP1
LRP10
LRP11
LRP1B
LRP2
LRP3
LRP4
LRP5
LRP5L
LRP6
LRP8
LRPAP1
LRPPRC
LRRC1
LRRC14
LRRC16
LRRC16A
LRRC20
LRRC23

LRRC28
LRRC3
LRRC32
LRRC37B
LRRC37B2
LRRC40
LRRC41
LRRC42
LRRC45
LRRC47
LRRC49
LRRC57
LRRC58
LRRC59
LRRC6
LRRC61
LRRC69
LRRC8A
LRRC8D
LRRC8E
LRRCC1
LRRFIP1
LRRFIP2
LRRN1
LRRN2
LRRN4
LRSAM1
LRTOMT
LRWD1
LSAMP
LSG1
LSM1
LSM10
LSM12
LSM14A
LSM2
LSM3
LSM4
LSM5
LSM6
LSM7
LSM8
LSMD1
LSP1
LSR
LSS
LTA
LTA4H
LTB4R
LTBP2

LTBP3
LTBP4
LTBR
LTV1
LUC7L
LUC7L2
LUM
LUZP1
LXN
LY6E
LYAR
LYL1
LYN
LYPD1
LYPD3
LYPD6
LYPD6B
LYPLA1
LYPLA2
LYPLAL1
LYRM1
LYRM2
LYRM4
LYRM5
LYRM7
LYSMD1
LYSMD2
LYSMD3
LYSMD4
LZIC
LZTFL1
LZTR1
LZTS2
M6PR
M6PRBP1
MAB21L2
MACF1
MACROD1
MAD1L1
MAD2L1
MAD2L1BP
MAD2L2
MADD
MAEA
MAF
MAF1
MAFB
MAFF
MAFG
MAGEC2

MAGED1
MAGED2
MAGED4B
MAGEE1
MAGEF1
MAGEH1
MAGEL2
MAGI1
Magmas
MAGOH
MAGOHB
MAGT1
MAK10
MAK16
MAL2
MALT1
MAMDC2
MAML1
MAN1A1
MAN1A2
MAN1B1
MAN1C1
MAN2A1
MAN2B1
MAN2B2
MAN2C1
MANBA
MANBAL
MANEA
MANSC1
MAOA
MAP1B
MAP1LC3A
MAP1LC3B
MAP1S
MAP2K1
MAP2K1IP1
MAP2K2
MAP2K3
MAP2K4
MAP2K5
MAP2K7
MAP3K1
MAP3K10
MAP3K11
MAP3K12
MAP3K14
MAP3K2
MAP3K3
MAP3K4

MAP3K6
MAP3K7
MAP3K7IP1
MAP3K7IP2
MAP3K8
MAP3K9
MAP4
MAP4K1
MAP4K2
MAP4K3
MAP4K4
MAP4K5
MAP6D1
MAP7
MAP7D1
MAP7D3
MAPBPIP
MAPK1
MAPK10
MAPK13
MAPK3
MAPK4
MAPK6
MAPK7
MAPK8IP1
MAPK8IP3
MAPK9
MAPKAP1
MAPKAPK2
MAPKAPK3
MAPKAPK5
MAPKSP1
MAPRE1
MAPRE2
MAPRE3
11. Mrz
02. Mrz
03. Mrz
05. Mrz
06. Mrz
07. Mrz
MARCKS
MARCKSL1
MARK1
MARK2
MARK3
MARS
MARS2
MARVELD1
MARVELD2

MARVELD3
MASP1
MAST2
MAST3
MASTL
MAT2A
MAT2B
MATK
MATN2
MATN3
MATR3
MAX
MBD1
MBD2
MBD3
MBD4
MBD6
MBIP
MBL1P1
MBLAC2
MBNL1
MBNL2
MBNL3
MBOAT1
MBOAT2
MBOAT7
MBP
MBTD1
MBTPS1
MC1R
MCART1
MCAT
MCCC1
MCEE
MCF2L
MCFD2
MCHR2
MCL1
MCM10
MCM2
MCM3
MCM3AP
MCM3APAS
MCM4
MCM5
MCM6
MCM7
MCM8
MCOLN1
MCOLN2

MCOLN3
MCPH1
MCRS1
MCTS1
MDC1
MDH1
MDH2
MDK
MDN1
MDP1
MDS1
ME1
ME2
ME3
MEA1
MEAF6
MECR
MED1
MED10
MED11
MED12
MED13
MED13L
MED14
MED15
MED16
MED17
MED18
MED19
MED20
MED21
MED22
MED23
MED24
MED25
MED26
MED27
MED28
MED29
MED30
MED31
MED4
MED6
MED7
MED8
MED9
MEF2D
MEG8
MEGF10
MEGF6

MEGF8
MEIS1
MEIS2
MEIS3
MEIS3P1
MELK
MEMO1
MEN1
MEPCE
MERTK
MESDC1
MEST
MET
METAP1
METAP2
METRN
METRNL
METT10D
METT11D1
METT5D1
METTL1
METTL10
METTL11A
METTL13
METTL14
METTL2A
METTL3
METTL4
METTL5
METTL6
METTL7A
METTL7B
METTL9
MEX3A
MEX3B
MEX3C
MEX3D
MFAP1
MFAP2
MFAP3
MFAP3L
MFAP4
MFAP5
MFF
MFGE8
MFHAS1
MFN1
MFN2
MFSD1
MFSD10

MFSD11
MFSD3
MFSD5
MFSD6
MFSD6L
MFSD8
MGA
MGAT1
MGAT2
MGAT3
MGAT4A
MGAT4B
MGC102966
MGC10997
MGC11082
MGC12760
MGC12965
MGC13057
MGC15634
MGC15763
MGC16121
MGC16169
MGC16384
MGC16703
MGC18216
MGC20983
MGC23284
MGC24103
MGC26356
MGC27345
MGC27348
MGC2752
MGC29506
MGC3020
MGC3032
MGC3196
MGC33556
MGC35361
MGC3731
MGC39900
MGC40489
MGC42630
MGC4677
MGC52000
MGC57346
MGC57359
MGC61598
MGC70857
MGC71993
MGC72080

MGC72104
MGC87042
MGC87895
MGEA5
MGMT
MGRN1
MGST1
MGST2
MGST3
MIA3
MIB1
MIB2
MICAL1
MICALL1
MICB
MID1
MID1IP1
MID2
MIDN
MIER1
MIER2
MIF
MIF4GD
MIIP
MINA
MINK1
MINPP1
MIOS
MIPEP
MIPOL1
MIR1185-1
MIR1228
MIR125B2
MIR129-2
MIR130A
MIR1909
MIR1974
MIR1978
MIR205
MIR2116
MIR25
MIR300
MIR342
MIR345
MIR373
MIR488
MIR557
MIR586
MIR599
MIR607

MIR657
MIR877
MIR886
MIR98
MIS12
MITD1
MITF
MKI67
MKI67IP
MKKS
MKL1
MKL2
MKLN1
MKNK1
MKNK2
MKRN1
MKRN2
MKS1
MLC1
MLEC
MLF1
MLF1IP
MLF2
MLH1
MLH3
MLL
MLL4
MLL5
MLLT10
MLLT11
MLLT6
MLPH
MLST8
MLX
MLXIPL
MLYCD
MMAA
MMAB
MMACHC
MMADHC
MMD
MME
MMGT1
MMP11
MMP15
MMP2
MMP23A
MMP23B
MMP24
MMP28

MMS19
MMS19L
MN1
MNAT1
MND1
MNS1
MNT
MNX1
MOAP1
MOBK1B
MOBKL1A
MOBKL1B
MOBKL2A
MOBKL2B
MOBKL2C
MOBKL3
MOCOS
MOCS1
MOGS
MON1A
MON1B
MON2
MORC2
MORC4
MORF4L1
MORF4L2
MORG1
MORN2
MORN4
MOS
MOSC1
MOSC2
MOSPD1
MOSPD2
MOSPD3
MOV10
MPDU1
MPDZ
MPG
MPHOSPH10
MPHOSPH6
MPHOSPH8
MPHOSPH9
MPI
MPND
MPP1
MPP5
MPP6
MPPE1
MPPED2

MPRIP
MPST
MPV17
MPV17L2
MPZ
MPZL1
MPZL2
MRAP2
MRC2
MRE11A
MRFAP1
MRFAP1L1
MRI1
MRLC2
MRP63
MRPL1
MRPL10
MRPL11
MRPL12
MRPL13
MRPL14
MRPL15
MRPL16
MRPL17
MRPL18
MRPL19
MRPL2
MRPL20
MRPL21
MRPL22
MRPL23
MRPL24
MRPL27
MRPL28
MRPL3
MRPL30
MRPL32
MRPL33
MRPL34
MRPL35
MRPL36
MRPL37
MRPL38
MRPL39
MRPL4
MRPL40
MRPL41
MRPL42
MRPL43
MRPL44

MRPL45
MRPL46
MRPL47
MRPL48
MRPL49
MRPL50
MRPL51
MRPL52
MRPL53
MRPL54
MRPL55
MRPL9
MRPS10
MRPS11
MRPS12
MRPS14
MRPS15
MRPS16
MRPS17
MRPS18A
MRPS18B
MRPS18C
MRPS2
MRPS21
MRPS22
MRPS23
MRPS24
MRPS25
MRPS26
MRPS27
MRPS28
MRPS30
MRPS31
MRPS33
MRPS34
MRPS35
MRPS36
MRPS5
MRPS6
MRPS7
MRPS9
MRRF
MRS2
MRTO4
MSH2
MSH3
MSH5
MSH6
MSI1
MSI2

MSL1
MSL2
MSL3
MSL3L1
MSN
MSRB2
MSRB3
MST1
MST4
MSTO1
MSX1
MSX2
MT1A
MT1E
MT1F
MT1G
MT1H
MT1X
MT2A
MTA1
MTA2
MTA3
MTAP
MTBP
MTCH1
MTCH2
MTCP1
MTDH
MTE
MTERF
MTERFD1
MTF1
MTF2
MTFMT
MTFR1
MTG1
MTHFD1
MTHFD1L
MTHFD2
MTHFD2L
MTHFR
MTHFS
MTHFSD
MTIF2
MTIF3
MTL5
MTM1
MTMR1
MTMR10
MTMR11

MTMR12
MTMR14
MTMR15
MTMR2
MTMR3
MTMR4
MTMR6
MTMR7
MTMR9
MTO1
MTP18
MTPN
MTR
MTRF1
MTRF1L
MTRR
MTSS1
MTUS1
MTX1
MTX2
MTX3
MUC1
MUC16
MUDENG
MUL1
MUM1
MUM1L1
MURC
MUS81
MUSTN1
MUT
MUTED
MUTYH
MVD
MVK
MVP
MX1
MXD1
MXD3
MXD4
MXI1
MXRA5
MXRA7
MYADM
MYB
MYBBP1A
MYBL1
MYBL2
MYC
MYCBP

MYCBP2
MYCN
MYD88
MYH10
MYH9
MYL12A
MYL4
MYL5
MYL6
MYL6B
MYL7
MYL9
MYLIP
MYNN
MYO10
MYO18A
MYO19
MYO1B
MYO1C
MYO1D
MYO3A
MYO3B
MYO5A
MYO5B
MYO5C
MYO6
MYO9A
MYO9B
MYOF
MYOM2
MYOZ1
MYOZ3
MYPOP
MYST1
MYST2
MYST3
MZF1
N4BP1
N4BP2
N4BP2L1
N4BP2L2
N6AMT1
N6AMT2
NAAA
NAALAD2
NAB1
NAB2
NACA
NACA2
NACAP1

NACC1
NACC2
NADK
NADSYN1
NAE1
NAF1
NAG18
NAGA
NAGK
NAGLU
NAGPA
NAIF1
NAMPT
NANOS1
NANOS3
NANS
NAP1L1
NAP1L3
NAP1L4
NAP1L5
NAP1L6
NAPA
NAPB
NAPEPLD
NAPG
NAPRT1
NARF
NARFL
NARG1
NARG1L
NARG2
NARS
NARS2
NASP
NAT10
NAT11
NAT12
NAT13
NAT14
NAT15
NAT5
NAT6
NAT9
NAV1
NAV2
NBAS
NBEA
NBEAL2
NBL1
NBN

NBPF1
NBPF10
NBPF14
NBPF20
NBPF3
NBPF8
NBR2
NCALD
NCAM1
NCAN
NCAPD2
NCAPD3
NCAPG
NCAPG2
NCAPH2
NCBP1
NCBP2
NCCRP1
NCDN
NCK1
NCK2
NCKAP1
NCKIPSD
NCL
NCLN
NCOA1
NCOA3
NCOA4
NCOA5
NCOA6
NCOA6IP
NCOA7
NCOR1
NCOR2
NCRNA00081
NCRNA00085
NCRNA00087
NCRNA00092
NCRNA00094
NCRNA00095
NCRNA00152
NCRNA00153
NCRNA00200
NCRNA00219
NCSTN
NDC80
NDE1
NDEL1
NDFIP1
NDFIP2

NDN
NDNL2
NDRG1
NDRG2
NDRG3
NDRG4
NDST1
NDST2
NDUFA1
NDUFA10
NDUFA11
NDUFA12
NDUFA13
NDUFA2
NDUFA3
NDUFA4
NDUFA5
NDUFA6
NDUFA7
NDUFA8
NDUFA9
NDUFAB1
NDUFAF1
NDUFAF2
NDUFAF3
NDUFB1
NDUFB10
NDUFB11
NDUFB2
NDUFB3
NDUFB5
NDUFB6
NDUFB7
NDUFB8
NDUFB9
NDUFC1
NDUFS1
NDUFS2
NDUFS3
NDUFS4
NDUFS5
NDUFS7
NDUFS8
NDUFV1
NDUFV2
NDUFV3
NEBL
NECAB3
NECAP1
NECAP2

NEDD1
NEDD4
NEDD4L
NEDD8
NEDD9
NEFH
NEIL2
NEIL3
NEK1
NEK2
NEK3
NEK6
NEK8
NELF
NELL2
NENF
NEO1
NES
NET1
NETO2
NEU1
NEURL1B
NEURL2
NEURL4
NEUROG2
NEXN
NF1
NFAT5
NFATC1
NFATC2IP
NFATC3
NFE2
NFE2L1
NFE2L2
NFE2L3
NFIB
NFIL3
NFKB1
NFKB2
NFKBIA
NFKBIB
NFKBID
NFKBIE
NFKBIL2
NFKBIZ
NFRKB
NFU1
NFX1
NFXL1
NFYA

NFYB
NFYC
NGDN
NGEF
NGF
NGFRAP1
NGLY1
NGRN
NHLRC2
NHLRC3
NHP2
NHP2L1
NHS
NICN1
NID2
NIF3L1
NIN
NINJ1
NINL
NIP30
NIP7
NIPA1
NIPA2
NIPBL
NIPSNAP1
NIPSNAP3A
NISCH
NIT1
NIT2
NKAP
NKD2
NKIRAS1
NKIRAS2
NKRF
NKTR
NKX3-1
NLE1
NLGN1
NLGN2
NLGN4X
NLGN4Y
NLK
NLN
NLRP12
NLRP7
NLRP8
NLRX1
NMB
NMD3
NME1

NME1-NME2
NME2
NME3
NME4
NME5
NME6
NME7
NMI
NMNAT1
NMNAT2
NMRAL1
NMT1
NMT2
NMU
NNT
NOB1
NOC2L
NOC3L
NOC4L
NOD1
NODAL
NOL10
NOL11
NOL12
NOL3
NOL6
NOL7
NOL8
NOLA1
NOLC1
NOMO1
NOMO2
NONO
NOP10
NOP14
NOP16
NOP2
NOP56
NOP58
NOSIP
NOTCH1
NOTCH2
NOTCH2NL
NOTCH3
NOVA1
NOX4
NOXA1
NOXO1
NP
N-PAC

NPAL3
NPAS1
NPAT
NPB
NPC1
NPC2
NPDC1
NPEPL1
NPEPPS
NPHP1
NPHP3
NPHP4
NPIP
NPLOC4
NPM3
NPPB
NPR1
NPR2
NPTN
NPW
NPY
NPY5R
NQO1
NQO2
NR1D1
NR1D2
NR1H2
NR1H3
NR2C1
NR2C2
NR2C2AP
NR2F2
NR2F6
NR3C1
NR3C2
NR5A2
NRAS
NRBF2
NRBP1
NRBP2
NRCAM
NRD1
NRGN
NRIP1
NRIP3
NRK
NRM
NRP1
NRSN2
NSA2

NSBP1
NSD1
NSDHL
NSF
NSFL1C
NSL1
NSMAF
NSMCE1
NSMCE2
NSMCE4A
NSUN2
NSUN3
NSUN4
NSUN5
NSUN5B
NSUN5C
NSUN6
NSUN7
NT5C
NT5C2
NT5C3
NT5C3L
NT5DC1
NT5DC2
NT5DC3
NT5M
NTAN1
NTF3
NTHL1
NTS
NUAK1
NUAK2
NUB1
NUBP1
NUBP2
NUBPL
NUCB1
NUCB2
NUCKS1
NUDC
NUDCD1
NUDCD2
NUDCD3
NUDT1
NUDT11
NUDT14
NUDT15
NUDT16
NUDT16L1
NUDT18

NUDT2
NUDT21
NUDT22
NUDT3
NUDT5
NUDT6
NUDT7
NUDT9
NUF2
NUFIP1
NUFIP2
NUMA1
NUMB
NUMBL
NUP107
NUP133
NUP153
NUP155
NUP160
NUP188
NUP205
NUP210
NUP214
NUP35
NUP37
NUP43
NUP50
NUP54
NUP62
NUP62CL
NUP85
NUP88
NUP93
NUPL2
NUPR1
NUS1
NUSAP1
NUTF2
NVL
NXF1
NXN
NXNL1
NXT1
NXT2
NYNRIN
NY-REN-7
OAF
OAS1
OAT
OAZ1

OAZ2
OBFC1
OBFC2A
OBFC2B
OBSCN
OBSL1
OCEL1
OCIAD1
OCIAD2
OCRL
ODC1
ODF2
ODF2L
ODZ3
ODZ4
OFD1
OGDH
OGDHL
OGFOD1
OGFR
OGFRL1
OGG1
OGT
OIP5
OKL38
OLFML3
OMA1
OPA1
OPA3
OPLAH
OPN3
OPRL1
OPTN
OR11H1
OR1J1
OR2A20P
OR2A42
OR2A9P
OR2B2
OR56B1
OR7E156P
OR9A4
ORAI1
ORAI3
ORAOV1
ORC1L
ORC2L
ORC3L
ORC4L
ORC5L

ORC6L
ORMDL1
ORMDL2
ORMDL3
OS9
OSAP
OSBP
OSBPL10
OSBPL11
OSBPL1A
OSBPL2
OSBPL3
OSBPL5
OSBPL6
OSBPL7
OSBPL8
OSBPL9
OSCP1
OSGEP
OSGEPL1
OSGIN2
OSTalpha
OSTC
OSTCL
OSTF1
OSTM1
OTP
OTUB1
OTUB2
OTUD1
OTUD3
OTUD4
OTUD5
OTUD6B
OTX2
OVGP1
OVOL2
OXA1L
OXCT1
OXCT2
OXR1
OXSM
OXSR1
OXTR
P15RS
P2RX2
P2RX4
P2RY11
P2RY5
P2RY6

P4HA1
P4HA2
P4HB
P4HTM
P704P
P76
PA2G4
PAAF1
PABPC1
PABPC1L
PABPC3
PABPC4
PABPC4L
PABPC5
PABPN1
PACS1
PACS2
PACSIN1
PACSIN2
PACSIN3
PAF1
PAFAH1B1
PAFAH1B3
PAFAH2
PAG1
PAGE4
PAH
PAICS
PAIP1
PAIP2
PAK1
PAK1IP1
PAK2
PAK4
PAK6
PALB2
PALLD
PALM
PAM
PAMR1
PAN2
PAN3
PANK1
PANK2
PANK3
PANK4
PANX1
PANX2
PAOX
PAPD1

PAPD4
PAPD5
PAPLN
PAPOLA
PAPOLG
PAPPA
PAPSS1
PAPSS2
PAQR3
PAQR4
PAQR6
PAQR7
PAQR8
PAR5
PARD3
PARD6A
PARD6G
PARG
PARK7
PARL
PARM1
PARN
PARP1
PARP10
PARP11
PARP12
PARP14
PARP16
PARP2
PARP3
PARP4
PARP6
PARP8
PARP9
PARS2
PART1
PARVA
PARVB
PASK
PATE2
PATE3
PATL1
PAWR
PAXIP1
PBK
PBRM1
PBX1
PBX2
PBX3
PBXIP1

PC
PCBD1
PCBD2
PCBP1
PCBP2
PCBP4
PCCA
PCCB
PCDH10
PCDH11X
PCDH11Y
PCDH17
PCDH18
PCDH19
PCDH7
PCDHA1
PCDHA2
PCDHA3
PCDHA4
PCDHB16
PCDHB17
PCDHB18
PCDHB19P
PCDHB2
PCDHB3
PCDHB4
PCDHB5
PCDHB9
PCDHGB6
PCF11
PCGF1
PCGF2
PCGF6
PCID2
PCIF1
PCK2
PCM1
PCMT1
PCMTD1
PCMTD2
PCNA
PCNP
PCNT
PCNX
PCNXL2
PCNXL3
PCOLCE
PCOLCE2
PCSK1N
PCSK4

PCSK5
PCSK7
PCTK2
PCTK3
PCTP
PCYOX1
PCYOX1L
PCYT2
PDCD10
PDCD11
PDCD2
PDCD2L
PDCD4
PDCD5
PDCD6
PDCD6IP
PDCD7
PDCL
PDCL3
PDDC1
PDE4A
PDE4C
PDE4D
PDE5A
PDE6B
PDE6D
PDE7A
PDE9A
PDGFA
PDGFC
PDGFD
PDGFRA
PDGFRB
PDGFRL
PDHA1
PDHB
PDHX
PDIA3P
PDIA4
PDIA5
PDIA6
PDIK1L
PDK2
PDK3
PDK4
PDLIM1
PDLIM3
PDLIM7
PDP2
PDPK1

PDPN
PDPR
PDRG1
PDS5A
PDS5B
PDSS1
PDSS2
PDXDC1
PDXK
PDXP
PDZD2
PDZD8
PDZK1
PDZK1P1
PDZRN3
PEA15
PEBP1
PECI
PECR
PEF1
PEG10
PEG3
PELI1
PELI2
PELO
PELP1
PEMT
PEPD
PER1
PER2
PER3
PERP
PES1
PET112L
PEX1
PEX10
PEX11A
PEX11B
PEX11G
PEX13
PEX14
PEX16
PEX19
PEX5
PEX6
PEX7
PFAAP5
PFAS
PFDN1
PFDN2

PFDN4
PFDN5
PFDN6
PFKFB2
PFKFB3
PFKFB4
PFKL
PFKM
PFKP
PFN1
PFN2
PFTK1
PGAM1
PGAM4
PGAM5
PGAP1
PGAP3
PGBD1
PGBD2
PGBD3
PGCP
PGD
PGF
PGGT1B
PGK1
PGLS
PGM1
PGM2
PGM2L1
PGM3
PGM5
PGP
PGRMC1
PGRMC2
PGS1
PHACTR1
PHACTR2
PHACTR4
PHAX
PHB
PHB2
PHC1
PHC2
PHC3
PHCA
PHF1
PHF10
PHF11
PHF12
PHF13

PHF14
PHF15
PHF16
PHF17
PHF19
PHF2
PHF20
PHF20L1
PHF21A
PHF21B
PHF23
PHF3
PHF5A
PHF7
PHGDH
PHIP
PHKA1
PHKA2
PHKB
PHKG2
PHLDA1
PHLDA3
PHLDB1
PHLDB2
PHLDB3
PHLPP1
PHLPP2
PHOSPHO2
PHPT1
PHRF1
PHTF1
PHYH
PHYHD1
PI15
PI4K2A
PI4K2B
PI4KAP1
PI4KAP2
PI4KB
PIAS1
PIAS2
PIAS3
PIAS4
PIB5PA
PIBF1
PICALM
PICK1
PIF1
PIGA
PIGC

PIGF
PIGG
PIGH
PIGK
PIGL
PIGM
PIGN
PIGO
PIGP
PIGQ
PIGS
PIGT
PIGU
PIGV
PIGW
PIGX
PIGY
PIH1D1
PIK3C2A
PIK3C2B
PIK3C3
PIK3CA
PIK3CB
PIK3CD
PIK3IP1
PIK3R1
PIK3R2
PIK3R4
PIK4CA
PILRA
PILRB
PIM1
PIM2
PIM3
PIN1
PIN4
PINK1
PINX1
PIP3-E
PIP4K2A
PIP4K2B
PIP4K2C
PIP5K1C
PIP5K2A
PIP5K2B
PIPSL
PIR
PISD
PITPNA
PITPNB

PITPNC1
PITPNM1
PITRM1
PITX1
PITX2
PJA1
PJA2
PKD1
PKD2
PKDCC
PKIA
PKIB
PKIG
PKM2
PKMYT1
PKN1
PKN2
PKN3
PKNOX1
PKNOX2
PKP2
PKP4
PLA2G10
PLA2G12A
PLA2G15
PLA2G2D
PLA2G4B
PLA2G6
PLAA
PLAC1
PLAC9
PLAG1
PLAGL1
PLAGL2
PLAT
PLAU
PLCB1
PLCD1
PLCD3
PLCE1
PLCG1
PLCG2
PLCL2
PLCXD1
PLCXD3
PLD1
PLD2
PLD3
PLDN
PLEC1

PLEKHA1
PLEKHA2
PLEKHA3
PLEKHA4
PLEKHA5
PLEKHA6
PLEKHA7
PLEKHA9
PLEKHB2
PLEKHF1
PLEKHF2
PLEKHG2
PLEKHG3
PLEKHG4
PLEKHG6
PLEKHH1
PLEKHH3
PLEKHJ1
PLEKHM1
PLEKHM2
PLEKHN1
PLEKHO1
PLEKHO2
PLIN2
PLIN5
PLK1
PLK2
PLK4
PLLP
PLOD1
PLOD2
PLOD3
PLP1
PLRG1
PLS1
PLS3
PLSCR1
PLSCR3
PLSCR4
PLTP
PLXDC2
PLXNA1
PLXNA2
PLXNA3
PLXNA4
PLXNB1
PLXNB2
PLXND1
PMAIP1
PMEPA1

PMF1
PML
PMM1
PMM2
PMP22
PMPCA
PMPCB
PMS1
PMS2
PMS2CL
PMS2L1
PMS2L2
PMS2L3
PMS2L4
PMS2L5
PMVK
PNCK
PNKD
PNKP
PNMA1
PNMAL1
PNN
PNO1
PNPLA2
PNPLA6
PNPLA7
PNPLA8
PNPO
PNPT1
PNRC2
PODXL
POFUT1
POFUT2
POGK
POGZ
POL3S
POLA1
POLA2
POLB
POLD1
POLD2
POLD3
POLD4
POLDIP2
POLDIP3
POLE
POLE2
POLE3
POLE4
POLG

POLG2
POLI
POLL
POLM
POLN
POLQ
POLR1B
POLR1C
POLR1D
POLR1E
POLR2A
POLR2B
POLR2C
POLR2D
POLR2E
POLR2F
POLR2G
POLR2H
POLR2I
POLR2J
POLR2J2
POLR2J3
POLR2J4
POLR2K
POLR2L
POLR3A
POLR3B
POLR3C
POLR3D
POLR3E
POLR3F
POLR3G
POLR3GL
POLR3H
POLR3K
POLRMT
POLS
POM121C
POMC
POMGNT1
POMP
POMT1
POMT2
PON2
POP1
POP4
POP5
POP7
POR
PORCN

POT1
POTEF
POTEG
POU2F1
POU5F1P1
POU6F1
PP14571
PPA1
PPA2
PPAN
PPAP2A
PPAP2B
PPAP2C
PPAPDC1B
PPAPDC2
PPARBP
PPARD
PPARG
PPARGC1A
PPAT
PPBP
PPCS
PPDPF
PPFIA1
PPFIBP1
PPFIBP2
PPHLN1
PPIA
PPIAL4A
PPIB
PPIC
PPIE
PPIG
PPIH
PPIL1
PPIL2
PPIL3
PPIL5
PPL
PPM1A
PPM1B
PPM1D
PPM1E
PPM1F
PPM1G
PPM1H
PPM1K
PPM1M
PPME1
POX

PPP1CA
PPP1CB
PPP1CC
PPP1R10
PPP1R11
PPP1R12A
PPP1R12B
PPP1R12C
PPP1R13B
PPP1R13L
PPP1R14A
PPP1R14B
PPP1R15A
PPP1R15B
PPP1R16A
PPP1R1A
PPP1R2
PPP1R3B
PPP1R3C
PPP1R3D
PPP1R3F
PPP1R7
PPP1R8
PPP1R9A
PPP2CA
PPP2CB
PPP2R1A
PPP2R1B
PPP2R2A
PPP2R2B
PPP2R2D
PPP2R3A
PPP2R3B
PPP2R3C
PPP2R4
PPP2R5A
PPP2R5B
PPP2R5C
PPP2R5D
PPP2R5E
PPP3CA
PPP3CB
PPP3CC
PPP3R1
PPP4C
PPP4R1
PPP4R1L
PPP4R4
PPP6C
PPPDE1

PPPDE2
PPRC1
PPT1
PPT2
PPTC7
PPWD1
PQBP1
PQLC1
PQLC2
PQLC3
PRAF2
PRAGMIN
PRAMEF7
PRB3
PRC1
PRCC
PRCP
PRDM1
PRDM10
PRDM4
PRDX1
PRDX2
PRDX3
PRDX4
PRDX5
PRDX6
PREB
PREI3
PRELID1
PREP
PREPL
PREX1
PRIC285
PRICKLE1
PRICKLE2
PRICKLE3
PRICKLE4
PRIM1
PRIM2A
PRINS
PRKAA1
PRKAB1
PRKAB2
PRKACB
PRKAG1
PRKAG2
PRKAR1A
PRKAR2A
PRKCA
PRKCABP

PRKCD
PRKCDBP
PRKCH
PRKCI
PRKCQ
PRKCSH
PRKCZ
PRKD1
PRKD2
PRKD3
PRKDC
PRKRA
PRKRIP1
PRKRIR
PRKX
PRKY
PRLR
PRMT1
PRMT10
PRMT2
PRMT3
PRMT5
PRMT6
PRMT7
PRNP
PRNPIP
PRO1853
PROCA1
PROCR
PRODH
PROK1
PROM1
PROM2
PROS1
ProSAPiP1
PROSC
PRPF18
PRPF19
PRPF3
PRPF31
PRPF38A
PRPF38B
PRPF4
PRPF40A
PRPF4B
PRPF6
PRPF8
PRPH
PRPS1
PRPS2

PRPSAP1
PRPSAP2
PRR11
PRR13
PRR14
PRR15L
PRR19
PRR3
PRR4
PRR5
PRRC1
PRRG1
PRRG2
PRRG4
PRRT1
PRRT2
PRRT3
PRRX1
PRSS12
PRSS16
PRSS23
PRSS35
PRSS7
PRSS8
PRTFDC1
PRUNE
PSAP
PSAT1
PSCD1
PSCD2
PSCDBP
PSD
PSD3
PSEN1
PSEN2
PSENEN
PSIP1
PSKH1
PSMA1
PSMA2
PSMA3
PSMA4
PSMA5
PSMA6
PSMB1
PSMB10
PSMB2
PSMB3
PSMB4
PSMB5

PSMB6
PSMB7
PSMB8
PSMC1
PSMC2
PSMC3
PSMC3IP
PSMC4
PSMC5
PSMC6
PSMD1
PSMD10
PSMD11
PSMD12
PSMD13
PSMD14
PSMD2
PSMD3
PSMD4
PSMD5
PSMD6
PSMD7
PSMD8
PSMD9
PSME1
PSME2
PSME3
PSME4
PSMF1
PSMG1
PSMG2
PSMG3
PSMG4
PSORS1C1
PSPC1
PSPH
PSRC1
PSTK
PSTPIP2
PTAR1
PTBP1
PTBP2
PTCD1
PTCD2
PTCD3
PTCH1
PTDSS1
PTDSS2
PTEN
PTER

PTGES
PTGES2
PTGES3
PTGFRN
PTGIS
PTGR1
PTGR2
PTGS2
PTH1R
PTK2
PTK2B
PTK7
PTMA
PTMS
PTN
PTOV1
PTP4A1
PTP4A2
PTP4A3
PTPDC1
PTPLA
PTPLAD1
PTPLAD2
PTPLB
PTPMT1
PTPN1
PTPN11
PTPN12
PTPN13
PTPN14
PTPN2
PTPN21
PTPN23
PTPN3
PTPN4
PTPN6
PTPN9
PTPRA
PTPRD
PTPRE
PTPRF
PTPRG
PTPRK
PTPRM
PTPRR
PTPRU
PTRF
PTRH1
PTRH2
PTS

PTTG1
PTTG1IP
PTTG3P
PUF60
PUM1
PUM2
PURA
PURB
PURG
PUS1
PUS3
PUS7
PUS7L
PUSL1
PVR
PVRL2
PVRL3
PWP1
PWP2
PWWP2
PWWP2A
PWWP2B
PXDN
PXK
PXMP2
PXMP3
PXN
PYCARD
PYCR1
PYCR2
PYGB
PYGL
PYGO2
PYROXD1
QARS
QDPR
QKI
QPCT
QPRT
QRFPR
QRICH1
QSER1
QSOX1
QSOX2
QTRT1
QTRTD1
R3HCC1
R3HDM1
R3HDM2
RAB10

RAB11A
RAB11FIP1
RAB11FIP2
RAB11FIP3
RAB11FIP4
RAB11FIP5
RAB12
RAB13
RAB14
RAB15
RAB17
RAB18
RAB1A
RAB1B
RAB20
RAB21
RAB22A
RAB23
RAB24
RAB25
RAB27A
RAB28
RAB2A
RAB2B
RAB30
RAB31
RAB32
RAB33B
RAB34
RAB35
RAB37
RAB38
RAB3B
RAB3GAP1
RAB3GAP2
RAB3IL1
RAB3IP
RAB40B
RAB40C
RAB43
RAB4A
RAB4B
RAB5A
RAB5B
RAB5C
RAB6A
RAB6B
RAB7A
RAB8A
RAB8B

RAB9A
RABAC1
RABEP1
RABEPK
RABGAP1
RABGAP1L
RABGEF1
RABGGTA
RABGGTB
RABIF
RABL2A
RABL2B
RABL3
RABL4
RAC1
RAC2
RAC3
RACGAP1
RAD1
RAD17
RAD21
RAD23A
RAD23B
RAD50
RAD51
RAD51AP1
RAD51C
RAD51L1
RAD51L3
RAD52
RAD54B
RAD54L
RAD54L2
RAD9A
RADIL
RAE1
RAF1
RAG1AP1
RAGE
RAI1
RAI14
RALA
RALB
RALBP1
RALGAPA1
RALGAPB
RALGDS
RALGPS1
RALY
RALYL

RAN
RANBP1
RANBP10
RANBP2
RANBP2L1
RANBP3
RANBP6
RANBP9
RANGAP1
RANGRF
RAP1B
RAP1BL
RAP1GAP
RAP1GDS1
RAP2A
RAP2C
RAPGEF1
RAPGEF2
RAPGEF5
RAPGEF6
RAPGEFL1
RAPH1
RARA
RARRES2
RARRES3
RARS
RARS2
RASA1
RASA4P
RASAL2
RASAL3
RASD1
RASIP1
RASL10B
RASL11A
RASL11B
RASL12
RASSF1
RASSF2
RASSF4
RASSF5
RASSF6
RASSF7
RASSF9
RAVER1
RAVER2
RAX2
RAXL1
RB1CC1
RBBP4

RBBP5
RBBP6
RBBP7
RBBP8
RBBP9
RBCK1
RBED1
RBKS
RBL2
RBM10
RBM11
RBM12
RBM12B
RBM14
RBM15
RBM15B
RBM16
RBM17
RBM18
RBM20
RBM22
RBM23
RBM25
RBM26
RBM27
RBM28
RBM3
RBM33
RBM34
RBM35A
RBM38
RBM39
RBM4
RBM41
RBM42
RBM45
RBM47
RBM4B
RBM5
RBM6
RBM7
RBM9
RBMS1
RBMS2
RBMS2P
RBMX
RBMX2
RBMY2FP
RBP1
RBP5

RBP7
RBPJ
RBPMS
RBPMS2
RBX1
RC3H2
RCADH5
RCAN1
RCAN2
RCAN3
RCBTB1
RCBTB2
RCC2
RCCD1
RCE1
RCHY1
RCL1
RCN1
RCN2
RCN3
RCOR2
RCOR3
RDBP
RDH10
RDH11
RDH13
RDH14
RDH5
RDM1
RDX
REC8
RECK
RECQL
RECQL4
RECQL5
REEP2
REEP4
REEP5
REEP6
REG1A
RELA
RELB
RELL1
RELN
RENBP
REP15
REPIN1
REPS1
REPS2
RER1

RERE
RERG
RETSAT
REV1
REXO1
REXO2
REXO4
RFC1
RFC2
RFC3
RFC4
RFC5
RFESD
RFFL
RFK
RFNG
RFP
RFPL3S
RFPL4A
RFTN1
RFWD2
RFWD3
RFX1
RFX3
RFX4
RFX5
RFX7
RFXANK
RFXAP
RG9MTD1
RG9MTD2
RG9MTD3
RGL1
RGL2
RGL3
RGMA
RGMB
RGPD4
RGPD8
RGS10
RGS12
RGS16
RGS17
RGS19
RGS2
RGS4
RGS5
RGS7BP
RHBDD1
RHBDD2

RHBDD3
RHBDF1
RHBDF2
RHBDL1
RHBDL2
RHBDL3
RHEB
RHOA
RHOB
RHOBTB1
RHOBTB2
RHOBTB3
RHOC
RHOD
RHOF
RHOG
RHOQ
RHOT1
RHOT2
RHOU
RHPN2
RIC3
RIC8A
RIC8B
RICH2
RICS
RIF1
RILPL1
RILPL2
RIMBP3
RIMKLB
RIMS2
RIMS3
RIMS4
RIN1
RIN2
RING1
RINT1
RIOK1
RIOK2
RIOK3
RIPK1
RIPK2
RIPK4
RIPK5
RIT1
RLF
RLN2
RMI1
RMND1

RMND5A
RMND5B
RN5S9
RN7SK
RN7SL1
RNASE1
RNASE10
RNASE4
RNASEH1
RNASEH2A
RNASEH2B
RNASEH2C
RNASEK
RNASEL
RNASEN
RNASET2
RND2
RND3
RNF10
RNF103
RNF11
RNF111
RNF112
RNF113A
RNF114
RNF115
RNF121
RNF122
RNF123
RNF126
RNF128
RNF13
RNF130
RNF135
RNF138
RNF14
RNF141
RNF144
RNF144A
RNF144B
RNF145
RNF146
RNF149
RNF150
RNF152
RNF157
RNF160
RNF165
RNF166
RNF167

RNF169
RNF170
RNF181
RNF182
RNF185
RNF187
RNF19A
RNF19B
RNF20
RNF213
RNF214
RNF215
RNF216
RNF216L
RNF217
RNF219
RNF220
RNF24
RNF25
RNF26
RNF31
RNF34
RNF38
RNF39
RNF4
RNF40
RNF41
RNF43
RNF44
RNF5
RNF5P1
RNF7
RNF8
RNFT1
RNFT2
RNGTT
RNH1
RNMT
RNMTL1
RNPC3
RNPEP
RNPEPL1
RNPS1
RNU105A
RNU1-3
RNU1-5
RNU1A3
RNU1F1
RNU1G2
RNU2-1

RNU4-2
RNU4ATAC
RNU6-1
RNU6-15
RNY1
RNY3
RNY5
ROBLD3
ROBO1
ROBO3
ROCK1
ROCK2
ROD1
ROGDI
ROM1
ROMO1
ROR1
ROR2
RORA
RP11-529I10.4
RP5-1022P6.2
RP9
RPA1
RPA2
RPA3
RPA4
RPAIN
RPAP1
RPAP2
RPAP3
RPE
RPESP
RPF1
RPF2
RPGR
RPIA
RPL10A
RPL11
RPL12
RPL12P6
RPL13
RPL13A
RPL13L
RPL14
RPL14L
RPL15
RPL17
RPL18
RPL18A
RPL19

RPL21
RPL22
RPL23
RPL23A
RPL23AP13
RPL23AP53
RPL23AP7
RPL24
RPL26
RPL26L1
RPL27
RPL27A
RPL28
RPL29
RPL29P2
RPL3
RPL30
RPL31
RPL31P10
RPL32
RPL34
RPL35
RPL35A
RPL36
RPL36A
RPL36AL
RPL37
RPL37A
RPL38
RPL39
RPL39L
RPL4
RPL41
RPL5
RPL6
RPL7
RPL7A
RPL7L1
RPL8
RPL9
RPLP0
RPLP1
RPLP2
RPN1
RPN2
RPP14
RPP21
RPP25
RPP38
RPP40

RPPH1
RPRC1
RPRD1A
RPRD1B
RPRD2
RPRM
RPS10
RPS10P3
RPS11
RPS12
RPS13
RPS14
RPS15
RPS15A
RPS16
RPS17
RPS18
RPS19
RPS19BP1
RPS2
RPS20
RPS21
RPS23
RPS24
RPS25
RPS26
RPS26L
RPS26P10
RPS26P11
RPS27
RPS27A
RPS27L
RPS28
RPS29
RPS3
RPS3A
RPS4X
RPS4Y1
RPS4Y2
RPS5
RPS6
RPS6KA1
RPS6KA2
RPS6KA3
RPS6KA4
RPS6KA5
RPS6KB1
RPS6KB2
RPS6KC1
RPS6KL1

RPS6P1
RPS7
RPS8
RPS9
RPSA
RPTOR
RPUSD1
RPUSD2
RPUSD3
RPUSD4
RQCD1
RRAGA
RRAGB
RRAGC
RRAGD
RRAS
RRAS2
RRBP1
RREB1
RRM1
RRM2B
RRN3
RRP1
RRP12
RRP15
RRP1B
RRP7A
RRP7B
RRP8
RRP9
RRS1
RSAD1
RSBN1
RSBN1L
RSC1A1
RSF1
RSL1D1
RSL24D1
RSPH3
RSPH9
RSPO2
RSPO3
RSPRY1
RSRC1
RSRC2
RSU1
RTCD1
RTEL1
RTF1
RTKN

RTKN2
RTN2
RTN3
RTN4
RTN4IP1
RTN4R
RTTN
RUFY1
RUFY2
RUFY3
RUNDC1
RUNDC2C
RUNX1T1
RUNX2
RUNX3
RUSC1
RUSC2
RUVBL1
RUVBL2
RWDD1
RWDD2A
RWDD2B
RWDD3
RWDD4A
RXRA
RXRB
RXRG
RYBP
RYK
RYR2
RYR3
S100A10
S100A11
S100A13
S100A14
S100A16
S100A4
S100A6
S100A9
S100PBP
S1PR3
S1PR4
SAAL1
SAC3D1
SACM1L
SACS
SAE1
SAFB
SAFB2
SALL2

SALL3
SALL4
SAMD1
SAMD11
SAMD4B
SAMD6
SAMD8
SAMM50
SAP130
SAP18
SAP30
SAP30BP
SAP30L
SAPS1
SAPS2
SAPS3
SAR1A
SAR1B
SARS
SARS2
SART3
SASH1
SASS6
SAT1
SAT2
SATB1
SATB2
SAV1
SBDS
SBDSP
SBF1
SBF2
SBK1
SBNO2
SC4MOL
SC5DL
SC65
SCAMP1
SCAMP2
SCAMP3
SCAMP4
SCAMP5
SCAND1
SCAND2
SCAND3
SCAP
SCAPER
SCARA3
SCARB1
SCARB2

SCARF2
SCARNA10
SCARNA13
SCARNA16
SCCPDH
SCD
SCD5
SCFD1
SCG5
SCGB3A2
SCHIP1
SCLT1
SCLY
SCMH1
SCML1
SCML2
SCNM1
SCNN1A
SCNN1D
SCO1
SCO2
SCOC
SCPEP1
SCRIB
SCRN1
SCRN2
SCRN3
SCTR
SCYL1
SCYL1BP1
SCYL2
SCYL3
SDAD1
SDC1
SDC2
SDC4
SDCBP
SDCCAG1
SDCCAG10
SDCCAG3
SDF2
SDF2L1
SDF4
SDHA
SDHAF1
SDHAF2
SDHALP1
SDHAP2
SDHAP3
SDHB

SDHC
SDHD
SDK2
SDSL
SEC11A
SEC11C
SEC13
SEC14L1
SEC16A
SEC22A
SEC22B
SEC22C
SEC23A
SEC23B
SEC23IP
SEC24A
SEC24B
SEC24C
SEC24D
SEC31A
SEC31B
SEC61A1
SEC61A2
SEC61B
SEC61G
SEC62
SEC63
SECISBP2
SECISBP2L
SEH1L
SEL1L
SEL1L3
SELENBP1
SELI
SELK
SELM
SELO
SELS
SELT
SELV
SEMA3A
SEMA3C
SEMA3E
SEMA3F
SEMA4B
SEMA4C
SEMA4D
SEMA4F
SEMA4G
SEMA5A

SEMA5B
SEMA6A
SEMA6B
SEMA6C
SENP2
SENP3
SENP5
SENP6
SENP7
15. Sep
SEPHS1
SEPHS2
SEPN1
SEPP1
SEPSECS
10. Sep
11. Sep
02. Sep
03. Sep
04. Sep
05. Sep
06. Sep
07. Sep
09. Sep
SEPW1
SEPX1
SERAC1
SERBP1
SERF1A
SERF1B
SERF2
SERGEF
SERHL
SERINC1
SERINC2
SERINC3
SERINC4
SERINC5
SERP1
SERPINA1
SERPINA10
SERPINA5
SERPINB1
SERPINB6
SERPINB9
SERPINE1
SERPINE2
SERPINF1
SERPING1
SERPINH1

SERPINI1
SERTAD1
SERTAD2
SERTAD3
SERTAD4
SESN1
SESN2
SESN3
SESTD1
SET
SETBP1
SETD1A
SETD1B
SETD2
SETD3
SETD4
SETD5
SETD6
SETD8
SETDB1
SETDB2
SETMAR
SETX
SEZ6L2
SF1
SF3A1
SF3A2
SF3A3
SF3B1
SF3B14
SF3B2
SF3B3
SF3B4
SF3B5
SF4
SFMBT1
SFMBT2
SFPQ
SFRP1
SFRP2
SFRS1
SFRS10
SFRS11
SFRS12
SFRS13A
SFRS13B
SFRS14
SFRS15
SFRS16
SFRS17A

SFRS18
SFRS2
SFRS2B
SFRS2IP
SFRS3
SFRS4
SFRS5
SFRS6
SFRS7
SFRS8
SFRS9
SFT2D1
SFT2D2
SFT2D3
SFTPD
SFXN1
SFXN2
SFXN4
SFXN5
SGCB
SGCE
SGK
SGK1
SGK3
SGOL1
SGOL2
SGPL1
SGPP2
SGSH
SGSM1
SGSM2
SGSM3
SGTA
SH2B1
SH2B3
SH2D1A
SH2D3A
SH2D4A
SH2D5
SH3BGRL
SH3BGRL2
SH3BGRL3
SH3BP1
SH3BP4
SH3BP5L
SH3D19
SH3GL1
SH3GL2
SH3GL3
SH3GLB1

SH3GLB2
SH3KBP1
SH3PXD2A
SH3PXD2B
SH3RF1
SH3RF2
SH3YL1
SHANK3
SHARPIN
SHB
SHBG
SHC1
SHC2
SHCBP1
SHF
SHFM1
SHISA2
SHISA5
SHKBP1
SHMT1
SHMT2
SHOC2
SHPK
SHPRH
SHQ1
SHRM
SHROOM2
SHROOM3
SHROOM4
SIAE
SIAH1
SIAH2
SIDT2
SIGIRR
SIGLEC6
SIGMAR1
SIK2
SIK3
SIKE
SIL1
SILV
SIN3A
SIN3B
SIP1
SIPA1
SIPA1L1
SIPA1L2
SIPA1L3
SIRPA
SIRT1

SIRT2
SIRT4
SIRT5
SIRT7
SIVA
SIVA1
SIX4
SIX5
SKA1
SKA2
SKAP2
SKI
SKIL
SKIV2L
SKIV2L2
SKP1
SKP1A
SKP2
SLAIN1
SLAIN2
SLAMF7
SLBP
SLC10A3
SLC10A7
SLC11A2
SLC12A2
SLC12A4
SLC12A6
SLC12A8
SLC12A9
SLC13A4
SLC15A1
SLC15A3
SLC15A4
SLC16A1
SLC16A10
SLC16A12
SLC16A2
SLC16A3
SLC16A4
SLC16A5
SLC16A9
SLC19A1
SLC19A2
SLC19A3
SLC1A1
SLC1A3
SLC1A5
SLC20A1
SLC20A2

SLC22A15
SLC22A17
SLC22A18
SLC22A23
SLC22A5
SLC23A2
SLC24A1
SLC24A3
SLC24A6
SLC25A1
SLC25A10
SLC25A11
SLC25A12
SLC25A13
SLC25A14
SLC25A15
SLC25A16
SLC25A17
SLC25A18
SLC25A19
SLC25A20
SLC25A22
SLC25A23
SLC25A25
SLC25A26
SLC25A28
SLC25A29
SLC25A3
SLC25A36
SLC25A37
SLC25A38
SLC25A39
SLC25A4
SLC25A40
SLC25A42
SLC25A43
SLC25A44
SLC25A45
SLC25A46
SLC25A5
SLC25A6
SLC26A11
SLC26A2
SLC26A6
SLC27A1
SLC27A2
SLC27A3
SLC27A5
SLC27A6
SLC29A1

SLC29A2
SLC29A3
SLC29A4
SLC2A1
SLC2A10
SLC2A11
SLC2A12
SLC2A14
SLC2A3
SLC2A4RG
SLC2A5
SLC2A8
SLC30A1
SLC30A3
SLC30A5
SLC30A7
SLC30A9
SLC31A1
SLC31A2
SLC33A1
SLC35A1
SLC35A2
SLC35A3
SLC35A4
SLC35A5
SLC35B1
SLC35B2
SLC35B3
SLC35B4
SLC35C1
SLC35C2
SLC35D2
SLC35E1
SLC35E3
SLC35F1
SLC35F2
SLC35F3
SLC35F5
SLC36A1
SLC36A4
SLC37A1
SLC37A3
SLC37A4
SLC38A1
SLC38A10
SLC38A2
SLC38A5
SLC38A6
SLC38A7
SLC38A9

SLC39A1
SLC39A10
SLC39A11
SLC39A13
SLC39A14
SLC39A2
SLC39A3
SLC39A4
SLC39A6
SLC39A7
SLC39A8
SLC3A2
SLC40A1
SLC41A1
SLC41A3
SLC43A1
SLC43A2
SLC43A3
SLC44A1
SLC44A2
SLC44A3
SLC44A4
SLC45A3
SLC45A4
SLC46A3
SLC47A1
SLC48A1
SLC4A11
SLC4A1AP
SLC4A2
SLC4A5
SLC4A7
SLC4A8
SLC5A10
SLC5A12
SLC5A3
SLC5A6
SLC5A8
SLC5A9
SLC6A10P
SLC6A15
SLC6A6
SLC6A8
SLC6A9
SLC7A1
SLC7A11
SLC7A2
SLC7A3
SLC7A5
SLC7A6

SLC7A6OS
SLC7A7
SLC7A8
SLC9A1
SLC9A3R1
SLC9A4
SLC9A5
SLC9A6
SLC9A7
SLC9A8
SLCO1A2
SLCO1B1
SLCO2A1
SLCO2B1
SLCO3A1
SLCO4A1
SLCO4C1
SLFN5
SLIT2
SLIT3
SLK
SLMAP
SLMO1
SLTM
SLU7
SMA4
SMA5
SMAD2
SMAD3
SMAD4
SMAD5
SMAD6
SMAD7
SMAGP
SMAP1
SMAP2
SMARCA1
SMARCA2
SMARCA4
SMARCA5
SMARCAD1
SMARCAL1
SMARCB1
SMARCC1
SMARCC2
SMARCD1
SMARCD2
SMARCD3
SMARCE1
SMC2

SMC3
SMC4
SMC6
SMCR5
SMCR7L
SMEK2
SMG1
SMG5
SMG6
SMG7
SMN1
SMN2
SMNDC1
SMO
SMOX
SMPD1
SMPD2
SMPDL3A
SMPDL3B
SMS
SMTN
SMU1
SMUG1
SMURF1
SMYD2
SMYD3
SMYD4
SMYD5
SNAI2
SNAP23
SNAP29
SNAP47
SNAP91
SNAPC1
SNAPC2
SNAPC3
SNAPC4
SNAPC5
SNAPIN
SNCA
SNCAIP
SND1
SNF8
SNHG1
SNHG11
SNHG12
SNHG3-RCC1
SNHG5
SNHG6
SNHG7

SNHG8
SNHG9
SNIP1
SNN
SNORA10
SNORA11D
SNORA12
SNORA18
SNORA24
SNORA25
SNORA26
SNORA29
SNORA32
SNORA33
SNORA41
SNORA42
SNORA45
SNORA57
SNORA58
SNORA59A
SNORA59B
SNORA5C
SNORA6
SNORA61
SNORA62
SNORA64
SNORA67
SNORA68
SNORA70
SNORA70B
SNORA70C
SNORA73A
SNORA73B
SNORA76
SNORA7B
SNORA8
SNORA84
SNORD104
SNORD12C
SNORD13
SNORD14A
SNORD17
SNORD21
SNORD22
SNORD31
SNORD35A
SNORD35B
SNORD36A
SNORD36C
SNORD38A

SNORD3A
SNORD3C
SNORD3D
SNORD49A
SNORD4A
SNORD52
SNORD57
SNORD6
SNORD65
SNORD68
SNORD73A
SNORD80
SNORD83B
SNRK
SNRNP200
SNRNP25
SNRNP27
SNRNP35
SNRNP40
SNRNP48
SNRNP70
SNRPA
SNRPA1
SNRPB
SNRPB2
SNRPC
SNRPD1
SNRPD2
SNRPD3
SNRPE
SNRPF
SNRPG
SNRPN
SNTA1
SNTB1
SNTB2
SNUPN
SNURF
SNW1
SNX1
SNX10
SNX11
SNX12
SNX13
SNX14
SNX16
SNX17
SNX19
SNX2
SNX21

SNX22
SNX24
SNX25
SNX26
SNX27
SNX29
SNX3
SNX30
SNX33
SNX4
SNX5
SNX6
SNX7
SNX8
SOAT1
SOCS1
SOCS2
SOCS3
SOCS4
SOCS5
SOD1
SOD2
SOHLH2
SOLH
SON
SORBS1
SORBS2
SORBS3
SORCS1
SORD
SORL1
SORT1
SOX11
SOX13
SOX15
SOX17
SOX18
SOX2
SOX21
SOX3
SOX4
SOX7
SOX8
SOX9
SP1
SP110
SP140L
SP2
SP3
SP4

SP5
SP6
SP8
SPA17
SPAG1
SPAG16
SPAG5
SPAG7
SPAG9
SPARC
SPAST
SPATA17
SPATA18
SPATA2
SPATA20
SPATA2L
SPATA5L1
SPATA6
SPATA7
SPATC1
SPATS2
SPATS2L
SPC24
SPC25
SPCS1
SPCS2
SPCS3
SPEN
SPG11
SPG20
SPG21
SPG3A
SPG7
SPHAR
SPHK1
SPHK2
SPIN1
SPIN3
SPIN4
SPINK1
SPINK5
SPINT1
SPINT2
SPINT3
SPIRE1
SPIRE2
SPN
SPNS1
SPNS2
SPOCK1

SPOCK2
SPON1
SPOP
SPOPL
SPP1
SPPL2A
SPPL2B
SPPL3
SPR
SPRED1
SPRED2
SPRR1A
SPRY1
SPRY2
SPRY4
SPRYD3
SPRYD4
SPRYD5
SPSB1
SPSB2
SPSB3
SPSB4
SPTAN1
SPTBN1
SPTBN2
SPTLC1
SPTLC2
SPTY2D1
SQLE
SQSTM1
SR140
SRA1
SRBD1
SRC
SRCRB4D
SRD5A1
SREBF1
SREBF2
SRF
SRFBP1
SRGAP1
SRGAP2
SRGAP3
SRI
SRM
SRP14
SRP14P1
SRP19
SRP54
SRP68

SRP72
SRP9
SRPK1
SRPK2
SRPR
SRPRB
SRPX
SRRD
SRRM1
SRRM1L
SRRM2
SRXN1
SS18
SS18L1
SS18L2
SSB
SSBP1
SSBP2
SSBP3
SSBP4
SSH2
SSH3
SSNA1
SSPN
SSPO
SSR1
SSR2
SSR3
SSR4
SSRP1
SSSCA1
SSTR2
SSU72
SSX2IP
ST13
ST14
ST3GAL1
ST3GAL2
ST3GAL3
ST3GAL5
ST3GAL6
ST5
ST6GAL1
ST6GALNAC2
ST6GALNAC3
ST6GALNAC4
ST6GALNAC6
ST7
ST7L
ST7OT1

ST8SIA4
STAG1
STAG2
STAG3
STAG3L1
STAG3L2
STAG3L3
STAG3L4
STAM
STAM2
STAMBP
STAMBPL1
STAP2
STAR
STARD10
STARD13
STARD3
STARD3NL
STARD5
STARD7
STARD8
STAT1
STAT2
STAT3
STAT4
STAT5A
STAT5B
STAT6
STAU1
STAU2
STBD1
STC1
STC2
STEAP1
STEAP2
STEAP3
STIL
STIM1
STIM2
STIP1
STK10
STK11
STK11IP
STK16
STK17B
STK19
STK24
STK25
STK3
STK35

STK36
STK38
STK38L
STK39
STK4
STK40
STMN1
STMN2
STMN3
STOM
STOML1
STOML2
STON1
STOX1
STOX2
STRA13
STRA6
STRADA
STRADB
STRAP
STRBP
STRC
STRN
STRN3
STRN4
STS-1
STT3A
STT3B
STUB1
STX10
STX12
STX16
STX17
STX1A
STX2
STX3
STX4
STX5
STX6
STX7
STX8
STXBP1
STXBP2
STXBP3
STXBP5
STXBP6
STYK1
STYXL1
SUB1
SUCLA2

SUCLG1
SUCLG2
SUDS3
SUGT1
SULF1
SULF2
SULT1A1
SULT1A2
SULT1A3
SULT1A4
SUMF1
SUMF2
SUMO1
SUMO1P3
SUMO2
SUMO3
SUOX
SUPT16H
SUPT3H
SUPT4H1
SUPT5H
SUPT6H
SUPT7L
SUPV3L1
SURF1
SURF2
SURF4
SURF6
SUSD1
SUSD2
SUSD3
SUSD5
SUV39H1
SUV39H2
SUV420H1
SUV420H2
SUZ12
SUZ12P
SV2A
SVEP1
SVIL
SVOPL
SWAP70
SYAP1
SYCE1L
SYDE1
SYDE2
SYF2
SYMPK
SYN1

SYN2
SYNCRIP
SYNE2
SYNGR1
SYNJ1
SYNJ2
SYNJ2BP
SYNM
SYNPR
SYPL1
SYS1
SYT1
SYT11
SYT13
SYT15
SYT17
SYT3
SYT6
SYT7
SYTL1
SYTL2
SYVN1
TAC3
TACC1
TACC2
TACC3
TACO1
TACSTD1
TACSTD2
TADA1L
TADA2A
TADA2B
TADA3
TAF1
TAF10
TAF12
TAF15
TAF1A
TAF1B
TAF1C
TAF1D
TAF1L
TAF2
TAF4
TAF5
TAF5L
TAF6
TAF6L
TAF7
TAF9

TAGLN
TAGLN2
TAGLN3
TALDO1
TANC1
TANC2
TANK
TAOK1
TAOK2
TAOK3
TAP1
TAP2
TAPBP
TAPT1
TARBP1
TARBP2
TARDBP
TARS
TARS2
TARSL2
TASP1
TATDN1
TATDN2
TATDN3
TAX1BP1
TAX1BP3
TAZ
TBC1D1
TBC1D10A
TBC1D10B
TBC1D13
TBC1D14
TBC1D15
TBC1D16
TBC1D17
TBC1D19
TBC1D2
TBC1D20
TBC1D22A
TBC1D22B
TBC1D23
TBC1D24
TBC1D25
TBC1D2B
TBC1D3B
TBC1D3C
TBC1D3F
TBC1D3G
TBC1D3H
TBC1D3I

TBC1D4
TBC1D5
TBC1D7
TBC1D8
TBC1D9
TBC1D9B
TBCA
TBCB
TBCC
TBCCD1
TBCD
TBCE
TBK1
TBKBP1
TBL1X
TBL1XR1
TBL2
TBL3
TBP
TBPL1
TBRG4
TBX1
TBX19
TBX2
TBX21
TBX3
TC2N
TCAM1
TCEA1
TCEA2
TCEA3
TCEAL1
TCEAL2
TCEAL3
TCEAL4
TCEAL7
TCEAL8
TCEB1
TCEB2
TCEB3
TCERG1
TCERG1L
TCF12
TCF19
TCF2
TCF20
TCF25
TCF3
TCF4
TCF7L1

TCF7L2
TCFL5
TCHP
TCIRG1
TCL1B
TCL6
TCN2
TCP1
TCP11L1
TCTA
TCTEX1D2
TCTN1
TCTN3
TDG
TDO2
TDP1
TDRD1
TDRD3
TDRD7
TEAD2
TEAD3
TEAD4
TECPR1
TECR
TEF
TEK
TEKT1
TELO2
TERF1
TERF2
TERF2IP
TES
TESK1
TESK2
TESSP5
TET1
TEX10
TEX2
TEX261
TEX264
TEX9
TFAM
TFAMP1
TFAP2A
TFAP2C
TFAP4
TFB1M
TFB2M
TFCP2
TFDP1

TFDP2
TFE3
TFG
TFIP11
TFPI
TFPT
TFRC
TGDS
TGFB1I1
TGFB3
TGFBI
TGFBR2
TGFBR3
TGFBRAP1
TGIF1
TGIF2
TGM1
TGM2
TGOLN2
TH1L
THADA
THAP1
THAP10
THAP11
THAP6
THAP7
THAP9
THBS3
THBS4
THEM2
THNSL1
THNSL2
THOC1
THOC2
THOC3
THOC4
THOC5
THOC6
THOC7
THOP1
THRA
THRAP3
THRAP5
THSD1P
THSD4
THUMPD1
THUMPD2
THUMPD3
THY1
THYN1

TIA1
TIAF1
TIAL1
TIAM1
TIAM2
TICAM1
TICAM2
TIGA1
TIGD2
TIGD5
TIGD7
TIMELESS
TIMM10
TIMM17B
TIMM22
TIMM23
TIMM23B
TIMM44
TIMM8A
TIMM8B
TIMM9
TIMP1
TIMP2
TIMP4
TINF2
TINP1
TIPARP
TIPIN
TIPRL
TIRAP
TJAP1
TJP1
TJP2
TJP3
TK1
TK2
TKT
TLCD1
TLE1
TLE2
TLE3
TLE4
TLK1
TLK2
TLN1
TLN2
TM2D1
TM2D2
TM2D3
TM7SF2

TM7SF3
TM9SF1
TM9SF2
TM9SF3
TM9SF4
TMBIM1
TMBIM4
TMBIM6
TMC4
TMC6
TMC7
TMCC1
TMCO1
TMCO3
TMCO6
TMCO7
TMED1
TMED10
TMED10P
TMED2
TMED3
TMED4
TMED5
TMED7
TMED9
TMEFF1
TMEM1
TMEM100
TMEM101
TMEM104
TMEM106A
TMEM106B
TMEM106C
TMEM107
TMEM108
TMEM109
TMEM11
TMEM111
TMEM115
TMEM116
TMEM118
TMEM120A
TMEM123
TMEM125
TMEM126A
TMEM126B
TMEM127
TMEM128
TMEM129
TMEM131

TMEM132A
TMEM132B
TMEM133
TMEM134
TMEM135
TMEM136
TMEM137
TMEM138
TMEM141
TMEM143
TMEM144
TMEM147
TMEM149
TMEM14A
TMEM14B
TMEM14C
TMEM14D
TMEM150A
TMEM154
TMEM156
TMEM158
TMEM159
TMEM160
TMEM161A
TMEM164
TMEM165
TMEM166
TMEM167A
TMEM167B
TMEM168
TMEM16A
TMEM16B
TMEM17
TMEM170A
TMEM170B
TMEM175
TMEM177
TMEM178
TMEM179B
TMEM18
TMEM180
TMEM181
TMEM183A
TMEM183B
TMEM184B
TMEM184C
TMEM185A
TMEM185B
TMEM186
TMEM187

TMEM188
TMEM189
TMEM189-UB
TMEM19
TMEM191A
TMEM191B
TMEM192
TMEM194
TMEM194A
TMEM198
TMEM199
TMEM2
TMEM200A
TMEM203
TMEM205
TMEM206
TMEM207
TMEM208
TMEM209
TMEM214
TMEM216
TMEM218
TMEM219
TMEM22
TMEM222
TMEM231
TMEM25
TMEM27
TMEM30A
TMEM30B
TMEM33
TMEM38A
TMEM38B
TMEM39A
TMEM39B
TMEM4
TMEM41A
TMEM41B
TMEM42
TMEM43
TMEM44
TMEM45A
TMEM47
TMEM48
TMEM49
TMEM5
TMEM50A
TMEM50B
TMEM51
TMEM53

TMEM54
TMEM55A
TMEM55B
TMEM57
TMEM59
TMEM59L
TMEM60
TMEM62
TMEM63A
TMEM63B
TMEM64
TMEM66
TMEM67
TMEM69
TMEM70
TMEM77
TMEM79
TMEM8
TMEM80
TMEM85
TMEM86B
TMEM87A
TMEM87B
TMEM88
TMEM9
TMEM90B
TMEM91
TMEM92
TMEM93
TMEM97
TMEM98
TMEM99
TMEM9B
TMF1
TMLHE
TMOD1
TMOD3
TMPRSS12
TMPRSS2
TMSB10
TMSB15A
TMSB4X
TMSB4Y
TMSL3
TMTC1
TMTC3
TMTC4
TMUB1
TMUB2
TMX1

TMX3
TMX4
TNC
TNFAIP1
TNFAIP3
TNFAIP8
TNFAIP8L1
TNFRSF10B
TNFRSF10C
TNFRSF10D
TNFRSF12A
TNFRSF19
TNFRSF1A
TNFRSF21
TNFRSF25
TNFRSF8
TNFSF13B
TNFSF14
TNFSF15
TNFSF4
TNIP1
TNIP2
TNK2
TNKS
TNKS1BP1
TNNC1
TNNI3
TNNT1
TNNT2
TNPO1
TNPO2
TNPO3
TNRC15
TNRC6A
TNRC6B
TNS1
TNS3
TOB1
TOB2
TOE1
TOLLIP
TOM1
TOM1L1
TOM1L2
TOMM20
TOMM22
TOMM34
TOMM40
TOMM40L
TOMM5

TOMM6
TOMM7
TOMM70A
TOP1
TOP1MT
TOP1P2
TOP2A
TOP2B
TOP3A
TOP3B
TOPBP1
TOPORS
TOR1A
TOR1AIP1
TOR1AIP2
TOR1B
TOR3A
TOX
TOX2
TOX4
TP53
TP53AP1
TP53BP1
TP53BP2
TP53I13
TP53I3
TP53INP1
TP53INP2
TP53RK
TP53TG1
TP63
TPBG
TPCN2
TPD52
TPD52L1
TPD52L2
TPI1
TPK1
TPM1
TPM2
TPM3
TPM4
TPMT
TPP1
TPP2
TPR
TPRG1L
TPRKB
TPST1
TPST2

TPT1
TPX2
TRA1P2
TRA2A
TRABD
TRADD
TRAF2
TRAF3IP1
TRAF3IP2
TRAF4
TRAF5
TRAF6
TRAF7
TRAFD1
TRAIP
TRAK1
TRAK2
TRAM1
TRAM2
TRAP1
TRAPPC1
TRAPPC2
TRAPPC2L
TRAPPC2P1
TRAPPC3
TRAPPC4
TRAPPC5
TRAPPC6A
TRAPPC6B
TRAPPC9
TRERF1
TRIAP1
TRIB1
TRIB2
TRIB3
TRIL
TRIM11
TRIM13
TRIM2
TRIM21
TRIM22
TRIM23
TRIM24
TRIM25
TRIM26
TRIM27
TRIM28
TRIM3
TRIM32
TRIM33

TRIM35
TRIM36
TRIM37
TRIM38
TRIM39
TRIM4
TRIM41
TRIM44
TRIM45
TRIM5
TRIM52
TRIM55
TRIM56
TRIM6
TRIM65
TRIM66
TRIM68
TRIM71
TRIM78P
TRIM8
TRIM9
TRIML1
TRIML2
TRIO
TRIOBP
TRIP10
TRIP11
TRIP12
TRIP13
TRIP4
TRIP6
TRIT1
TRK1
TRMT1
TRMT11
TRMT112
TRMT12
TRMT2A
TRMT2B
TRMT5
TRMT6
TRMT61A
TRMU
TRNAU1AP
TRNP1
TRNT1
TRO
TROAP
TROVE2
TRPC1

TRPC4AP
TRPM4
TRPT1
TRPV1
TRRAP
TRUB2
TSC1
TSC2
TSC22D1
TSC22D2
TSC22D3
TSC22D4
TSEN15
TSEN2
TSEN34
TSEN54
TSFM
TSG101
TSGA14
TSHZ1
TSHZ3
TSKU
TSLP
TSN
TSNAX
TSPAN10
TSPAN12
TSPAN13
TSPAN14
TSPAN15
TSPAN17
TSPAN18
TSPAN3
TSPAN31
TSPAN33
TSPAN4
TSPAN5
TSPAN6
TSPAN7
TSPAN9
TSPO
TSPYL1
TSPYL2
TSPYL3
TSPYL5
TSPYL6
TSR1
TSR2
TSSC1
TSSC4

TST
TSTA3
TSTD1
TSTD2
TTC1
TTC12
TTC13
TTC14
TTC15
TTC17
TTC19
TTC23
TTC25
TTC26
TTC27
TTC3
TTC31
TTC32
TTC33
TTC35
TTC37
TTC38
TTC39B
TTC39C
TTC4
TTC5
TTC7A
TTC7B
TTC8
TTC9C
TTF1
TTF2
TTK
TTL
TTLL1
TTLL12
TTLL3
TTLL4
TTLL5
TTPAL
TTRAP
TTTY14
TTTY15
TTYH2
TTYH3
TUB
TUBA1A
TUBA1B
TUBA1C
TUBA3D

TUBB
TUBB2A
TUBB2B
TUBB2C
TUBB3
TUBB4
TUBB4Q
TUBB6
TUBB8
TUBD1
TUBE1
TUBG1
TUBG2
TUBGCP2
TUBGCP3
TUBGCP4
TUBGCP5
TUBGCP6
TUFM
TUFT1
TUG1
TULP3
TULP4
TUSC1
TUSC2
TUSC3
TUSC4
TUT1
TWF2
TWIST1
TWIST2
TWISTNB
TWSG1
TXLNA
TXN
TXN2
TXNDC11
TXNDC12
TXNDC14
TXNDC15
TXNDC16
TXNDC17
TXNDC3
TXNDC5
TXNDC9
TXNIP
TXNL1
TXNL2
TXNL4B
TXNRD1

TXNRD2
TYK2
TYMS
TYRO3
TYSND1
TYW1
TYW1B
TYW3
U1SNRNPBP
U2AF1
U2AF1L2
U2AF1L4
U2AF2
UAP1
UAP1L1
UBA1
UBA2
UBA3
UBA5
UBA52
UBA6
UBAC1
UBAC2
UBAP1
UBAP2
UBAP2L
UBB
UBC
UBE1
UBE1C
UBE1DC1
UBE2A
UBE2C
UBE2CBP
UBE2D2
UBE2D3
UBE2D4
UBE2E1
UBE2E2
UBE2E3
UBE2F
UBE2G1
UBE2G2
UBE2H
UBE2I
UBE2J1
UBE2J2
UBE2K
UBE2L3
UBE2L6

UBE2M
UBE2N
UBE2O
UBE2Q1
UBE2Q2
UBE2R2
UBE2T
UBE2V1
UBE2V2
UBE2W
UBE2Z
UBE3A
UBE3B
UBE3C
UBE4A
UBE4B
UBFD1
UBIAD1
UBL3
UBL4A
UBL5
UBL7
UBLCP1
UBN1
UBN2
UBOX5
UBP1
UBQLN1
UBQLN2
UBQLN4
UBR2
UBR3
UBR4
UBR5
UBR7
UBTD1
UBTD2
UBTF
UBXN1
UBXN11
UBXN2A
UBXN2B
UBXN4
UBXN6
UBXN8
UCA1
UCHL1
UCHL3
UCHL5
UCHL5IP

UCK1
UCK2
UCKL1
UCN
UCP2
UCRC
UEVLD
UFC1
UFM1
UFSP2
UGCG
UGCGL1
UGCGL2
UGDH
UGP2
UGT1A3
UGT2B11
UGT3A1
UGT3A2
UGT8
UHMK1
UHRF1
UHRF1BP1
UHRF1BP1L
UHRF2
UIMC1
ULBP1
ULK1
ULK2
ULK3
UNC119
UNC119B
UNC13B
UNC45A
UNC50
UNC5B
UNC84A
UNC84B
UNC93B1
UNG
UNKL
UPF1
UPF2
UPF3A
UPF3B
UPLP
UPP1
UPRT
UQCC
UQCR

UQCRB
UQCRC1
UQCRC2
UQCRFS1
UQCRH
UQCRHL
UQCRQ
URB1
URB2
URG4
URM1
UROD
UROS
USE1
USF1
USF2
USH1G
USMG5
USO1
USP1
USP10
USP11
USP12
USP13
USP14
USP15
USP16
USP18
USP21
USP22
USP24
USP25
USP28
USP3
USP30
USP32
USP33
USP34
USP35
USP36
USP37
USP38
USP39
USP4
USP41
USP42
USP46
USP47
USP48
USP49

USP5
USP54
USP6
USP6NL
USP7
USP8
USP9X
USP9Y
USPL1
UST
UTP11L
UTP14A
UTP14C
UTP15
UTP18
UTP23
UTP3
UTP6
UTRN
UTS2
UTX
UTY
UVRAG
UXS1
UXT
VAC14
VAMP1
VAMP2
VAMP3
VAMP4
VAMP5
VAMP7
VAMP8
VANGL2
VARS
VARS2
VASH1
VASH2
VASN
VASP
VAT1
VAT1L
VAV2
VAV3
VBP1
VCAM1
VCAN
VCL
VCP
VCPIP1

VCX
VCX3A
VCX-C
VDAC1
VDAC2
VDAC3
VEGFA
VEGFB
VEGFC
VENTXP1
VEZF1
VEZT
VGF
VGLL4
VHL
VIL2
VIM
VIPR1
VISA
VKORC1
VKORC1L1
VLDLR
VPRBP
VPS11
VPS13A
VPS13C
VPS13D
VPS16
VPS18
VPS24
VPS25
VPS26
VPS26A
VPS26B
VPS28
VPS29
VPS33A
VPS33B
VPS35
VPS36
VPS37A
VPS37B
VPS37C
VPS37D
VPS39
VPS41
VPS45
VPS4A
VPS4B
VPS52

VPS54
VPS72
VPS8
VRK1
VRK2
VRK3
VTA1
VTCN1
VTI1B
VWA1
VWA5A
VWCE
VWF
WAC
WARS
WAS
WASF2
WASF3
WASH1
WASH2P
WASH5P
WASL
WASPIP
WBP1
WBP11
WBP2
WBP4
WBP5
WBSCR16
WBSCR19
WBSCR22
WBSCR27
WDFY1
WDFY2
WDFY3
WDHD1
WDR1
WDR12
WDR13
WDR18
WDR19
WDR20
WDR21A
WDR22
WDR23
WDR24
WDR25
WDR26
WDR27
WDR33

WDR34
WDR35
WDR36
WDR37
WDR4
WDR40A
WDR41
WDR42A
WDR43
WDR44
WDR45
WDR45L
WDR46
WDR48
WDR5
WDR51A
WDR51B
WDR53
WDR54
WDR55
WDR57
WDR59
WDR5B
WDR6
WDR60
WDR61
WDR62
WDR67
WDR68
WDR7
WDR70
WDR72
WDR73
WDR74
WDR75
WDR77
WDR79
WDR8
WDR81
WDR82
WDR85
WDR86
WDR89
WDR90
WDR91
WDR92
WDSOF1
WDSUB1
WDYHV1
WEE1

WFDC2
WFS1
WHAMM
WHSC1L1
WHSC2
WIBG
WIPF1
WIPI1
WIPI2
WIZ
WNK1
WNK3
WNT3
WNT4
WNT5A
WNT5B
WRB
WRN
WRNIP1
WSB1
WSB2
WSCD1
WTAP
WTIP
WWC1
WWC2
WWC3
WWOX
WWP1
WWP2
XAB2
XAF1
XBP1
XDH
XIAP
XIST
XKR6
XKR8
XPA
XPC
XPNPEP1
XPNPEP2
XPNPEP3
XPO1
XPO4
XPO5
XPO6
XPO7
XPR1
XRCC1

XRCC2
XRCC3
XRCC5
XRCC6
XRCC6BP1
XRN1
XRN2
XYLB
XYLT2
YAF2
YAP1
YARS
YARS2
YBX1
YBX2
YDJC
YEATS2
YEATS4
YES1
YIF1A
YIF1B
YIPF1
YIPF2
YIPF3
YIPF4
YIPF5
YIPF6
YJEFN3
YKT6
YME1L1
YOD1
YPEL1
YPEL2
YPEL3
YPEL5
YRDC
YTHDC1
YTHDC2
YTHDF1
YTHDF2
YTHDF3
YWHAB
YWHAE
YWHAG
YWHAH
YWHAQ
YWHAZ
YY1
YY1AP1
ZADH2

ZAK
ZBBX
ZBED1
ZBED3
ZBED4
ZBED5
ZBTB11
ZBTB17
ZBTB2
ZBTB20
ZBTB22
ZBTB24
ZBTB25
ZBTB3
ZBTB33
ZBTB34
ZBTB39
ZBTB4
ZBTB40
ZBTB42
ZBTB43
ZBTB44
ZBTB45
ZBTB46
ZBTB48
ZBTB5
ZBTB6
ZBTB7A
ZBTB8A
ZBTB8OS
ZBTB9
ZC3H10
ZC3H11B
ZC3H12A
ZC3H12B
ZC3H12C
ZC3H14
ZC3H15
ZC3H18
ZC3H3
ZC3H4
ZC3H5
ZC3H6
ZC3H7A
ZC3H8
ZC3HAV1
ZC3HC1
ZC4H2
ZCCHC11
ZCCHC14

ZCCHC17
ZCCHC24
ZCCHC3
ZCCHC6
ZCCHC7
ZCCHC8
ZCCHC9
ZCRB1
ZCWPW1
ZDHHC1
ZDHHC11
ZDHHC12
ZDHHC13
ZDHHC16
ZDHHC17
ZDHHC18
ZDHHC2
ZDHHC23
ZDHHC24
ZDHHC3
ZDHHC4
ZDHHC5
ZDHHC6
ZDHHC7
ZDHHC8
ZDHHC8P
ZDHHC9
ZER1
ZFAND1
ZFAND2A
ZFAND2B
ZFAND3
ZFAND5
ZFAND6
ZFC3H1
ZFHX2
ZFHX3
ZFP1
ZFP106
ZFP112
ZFP14
ZFP161
ZFP2
ZFP30
ZFP36
ZFP36L1
ZFP36L2
ZFP37
ZFP42
ZFP64

ZFP82
ZFP90
ZFP91
ZFPL1
ZFPM1
ZFR
ZFX
ZFY
ZFYVE1
ZFYVE16
ZFYVE19
ZFYVE20
ZFYVE21
ZFYVE26
ZFYVE27
ZGPAT
ZHX1
ZHX2
ZHX3
ZIC2
ZIC3
ZIK1
ZKSCAN1
ZKSCAN2
ZKSCAN3
ZKSCAN4
ZKSCAN5
ZMAT2
ZMAT3
ZMAT5
ZMIZ1
ZMIZ2
ZMPSTE24
ZMYM1
ZMYM2
ZMYM3
ZMYM4
ZMYM5
ZMYM6
ZMYND11
ZMYND19
ZMYND8
ZNF10
ZNF101
ZNF114
ZNF12
ZNF124
ZNF131
ZNF133
ZNF134

ZNF135
ZNF136
ZNF137
ZNF138
ZNF14
ZNF140
ZNF142
ZNF143
ZNF146
ZNF148
ZNF154
ZNF155
ZNF16
ZNF160
ZNF165
ZNF167
ZNF17
ZNF174
ZNF175
ZNF177
ZNF18
ZNF181
ZNF182
ZNF185
ZNF187
ZNF189
ZNF193
ZNF195
ZNF197
ZNF2
ZNF20
ZNF200
ZNF202
ZNF204
ZNF205
ZNF207
ZNF211
ZNF212
ZNF213
ZNF215
ZNF217
ZNF219
ZNF22
ZNF222
ZNF223
ZNF224
ZNF225
ZNF226
ZNF227
ZNF23

ZNF232
ZNF234
ZNF235
ZNF239
ZNF24
ZNF248
ZNF25
ZNF250
ZNF251
ZNF252
ZNF254
ZNF256
ZNF259
ZNF26
ZNF260
ZNF263
ZNF264
ZNF266
ZNF268
ZNF271
ZNF273
ZNF274
ZNF275
ZNF277
ZNF28
ZNF280B
ZNF280C
ZNF280D
ZNF281
ZNF282
ZNF283
ZNF285A
ZNF286A
ZNF286C
ZNF295
ZNF296
ZNF3
ZNF30
ZNF300
ZNF302
ZNF304
ZNF317
ZNF318
ZNF319
ZNF32
ZNF320
ZNF322A
ZNF322B
ZNF323
ZNF324

ZNF324B
ZNF326
ZNF329
ZNF330
ZNF331
ZNF333
ZNF334
ZNF335
ZNF337
ZNF33A
ZNF33B
ZNF34
ZNF341
ZNF343
ZNF347
ZNF35
ZNF354A
ZNF358
ZNF362
ZNF364
ZNF383
ZNF384
ZNF385A
ZNF394
ZNF395
ZNF397
ZNF398
ZNF407
ZNF408
ZNF410
ZNF415
ZNF416
ZNF417
ZNF418
ZNF419
ZNF420
ZNF423
ZNF425
ZNF426
ZNF428
ZNF430
ZNF431
ZNF432
ZNF433
ZNF434
ZNF436
ZNF438
ZNF439
ZNF442
ZNF443

ZNF444
ZNF446
ZNF45
ZNF451
ZNF454
ZNF462
ZNF467
ZNF468
ZNF470
ZNF471
ZNF473
ZNF48
ZNF480
ZNF483
ZNF484
ZNF485
ZNF486
ZNF488
ZNF490
ZNF491
ZNF493
ZNF498
ZNF500
ZNF502
ZNF503
ZNF507
ZNF509
ZNF511
ZNF512
ZNF512B
ZNF513
ZNF514
ZNF517
ZNF518A
ZNF518B
ZNF519
ZNF521
ZNF524
ZNF525
ZNF526
ZNF529
ZNF532
ZNF543
ZNF544
ZNF548
ZNF549
ZNF550
ZNF551
ZNF555
ZNF557

ZNF558
ZNF559
ZNF561
ZNF562
ZNF564
ZNF565
ZNF567
ZNF570
ZNF571
ZNF573
ZNF574
ZNF576
ZNF577
ZNF579
ZNF580
ZNF581
ZNF583
ZNF585A
ZNF585B
ZNF586
ZNF589
ZNF592
ZNF593
ZNF594
ZNF598
ZNF599
ZNF600
ZNF605
ZNF606
ZNF607
ZNF608
ZNF609
ZNF610
ZNF613
ZNF614
ZNF615
ZNF616
ZNF618
ZNF621
ZNF622
ZNF624
ZNF625
ZNF626
ZNF627
ZNF629
ZNF630
ZNF638
ZNF641
ZNF644
ZNF646

ZNF649
ZNF650
ZNF652
ZNF653
ZNF654
ZNF655
ZNF658
ZNF658B
ZNF660
ZNF667
ZNF668
ZNF669
ZNF670
ZNF671
ZNF672
ZNF673
ZNF674
ZNF675
ZNF679
ZNF680
ZNF681
ZNF682
ZNF684
ZNF687
ZNF688
ZNF689
ZNF69
ZNF691
ZNF692
ZNF695
ZNF696
ZNF697
ZNF7
ZNF700
ZNF701
ZNF702P
ZNF706
ZNF707
ZNF708
ZNF711
ZNF714
ZNF716
ZNF720
ZNF721
ZNF738
ZNF74
ZNF746
ZNF75A
ZNF75D
ZNF76

ZNF761
ZNF763
ZNF764
ZNF765
ZNF766
ZNF767
ZNF768
ZNF77
ZNF770
ZNF772
ZNF773
ZNF775
ZNF776
ZNF777
ZNF783
ZNF784
ZNF785
ZNF786
ZNF787
ZNF788
ZNF789
ZNF79
ZNF791
ZNF792
ZNF800
ZNF805
ZNF813
ZNF815
ZNF816A
ZNF821
ZNF823
ZNF827
ZNF828
ZNF83
ZNF830
ZNF837
ZNF839
ZNF84
ZNF841
ZNF845
ZNF85
ZNF860
ZNF91
ZNF93
ZNFX1
ZNHIT1
ZNHIT2
ZNHIT3
ZNHIT6
ZNRD1

ZNRF3
ZP3
ZRANB1
ZRANB2
ZRANB3
ZRSR2
ZSCAN12
ZSCAN12L1
ZSCAN16
ZSCAN18
ZSCAN2
ZSCAN21
ZSCAN29
ZSCAN5A
ZSWIM1
ZSWIM3
ZSWIM4
ZSWIM5
ZSWIM6
ZSWIM7
ZUFSP
ZW10
ZWILCH
ZWINT
ZXDC
ZYG11B
ZYX
ZZEF1
ZZZ3

Supplementary Table S7: List of genes used for heatmaps in Supplementary Figure S3 and regulatory network analysis via oPOSSUM data base.

POU5F1	iPSC/ESC
SOX2	DE
NANOG	HE
HNF1A	HLC
HNF1B	
LGR5	
ONECUT1	
ONECUT2	
ALB	
AFP	
TTR	
SOX17	
SALL4	
GPBAR1	
DLK1	
DLK2	
KIT	
CD34	
THY1	
CYP3A7	
CYP1A1	
TRIM15	
WNT3A	
SOX9	
NOTCH1	
NOTCH2	
NOTCH3	
JAG1	
JAG2	
FOXD4	
FOXJ1	
PROX1	
CXCR4	
GATA4	

Supplementary Table S8: Regulatory network analysis via oPOSSUM data base.

TF	JASPAR ID	Class	Target gene hits	Target TFBS hits	Z-score	Fisher score	p_fisher
Myc	MA0147.1	Zipper-Type	20	47	12,538	10,045	4,3402E-05
HNF1A	MA0046.1	Helix-Turn-Helix	8	12	11,142	6,095	0,00225411
SP1	MA0079.2	Zinc-coordinating	26	166	10,879	8,689	0,00016843
MZF1_5-13	MA0057.1	Zinc-coordinating	28	144	10,634	11,781	7,6485E-06
HNF4A	MA0114.1	Zinc-coordinating	18	25	10,585	13,199	1,8525E-06
Klf4	MA0039.2	Zinc-coordinating	28	176	10,414	9,748	5,8411E-05
Mycn	MA0104.2	Zipper-Type	21	45	9,937	10,917	1,8147E-05
MZF1_1-4	MA0056.1	Zinc-coordinating	28	341	9,472	5,875	0,00280879
INSM1	MA0155.1	Zinc-coordinating	19	38	9,017	9,948	4,7823E-05
ESR2	MA0258.1	Zinc-coordinating	6	7	8,72	4,996	0,00676495
Tcfcp2l1	MA0145.1	Other	24	58	8,586	11,598	9,1844E-06
HIF1A::ARNT	MA0259.1	Zipper-Type	24	79	7,882	9,734	5,9235E-05
NR2F1	MA0017.1	Zinc-coordinating	9	11	7,151	5,998	0,00248371
EBF1	MA0154.1	Zipper-Type	21	67	6,987	6,61	0,00134683
Ddit3::Cebpa	MA0019.1	Zipper-Type	10	19	6,879	4,026	0,01784557
FOXF2	MA0030.1	Winged Helix-Turn-Helix	5	13	6,758	1,371	0,25385298
Myf	MA0055.1	Zipper-Type	15	46	6,493	2,883	0,05596661
CEBPA	MA0102.2	Zipper-Type	18	82	6,326	3,134	0,04354328
Zfx	MA0146.1	Zinc-coordinating	25	62	6,141	12,815	2,7197E-06
REL	MA0101.1	Ig-fold	18	51	6,11	4,805	0,0081887
ZNF354C	MA0130.1	Zinc-coordinating	32	305	6,079	11,03	1,6208E-05
Gata1	MA0035.2	Zinc-coordinating	27	104	5,945	10,923	1,8039E-05
Evi1	MA0029.1	Zinc-coordinating	5	5	5,373	4,562	0,01044116
Arnt::Ahr	MA0006.1	Zipper-Type	26	111	5,35	8,846	0,00014396
Nobox	MA0125.1	Helix-Turn-Helix	20	106	5,301	4,043	0,01754476
ZEB1	MA0103.1	Zinc-coordinating	28	253	5,154	5,227	0,00536961
RELA	MA0107.1	Ig-fold	14	25	5,017	5,551	0,00388357
Pou5f1	MA0142.1	Helix-Turn-Helix	7	9	4,877	4,269	0,01399577
PPARG::RXRA	MA0065.2	Zinc-coordinating	11	19	4,841	3,983	0,01862967

Myb	MA0100.1	Helix-Turn-Helix	27	93	4,688	10,595	2,5041E-05
REST	MA0138.2	Zinc-coordinating	1	1	4,664	1,653	0,19147462
Sox2	MA0143.1	Other Alpha-Helix	6	7	4,575	4,042	0,01756231
NFIL3	MA0025.1	Zipper-Type	12	24	4,522	4,82	0,00806679
NF-kappaB	MA0061.1	Ig-fold	15	33	4,513	4,554	0,01052502
Zfp423	MA0116.1	Zinc-coordinating	11	18	4,352	4,124	0,01617967
NFE2L2	MA0150.1	Zipper-Type	9	15	4,308	3,352	0,03501426
Gfi	MA0038.1	Zinc-coordinating	20	90	4,209	3,539	0,02904235
NFKB1	MA0105.1	Ig-fold	9	13	4,01	4,455	0,01162032
HLF	MA0043.1	Zipper-Type	10	16	3,855	4,192	0,01511602
Sox17	MA0078.1	Other Alpha-Helix	25	89	3,733	8,542	0,0001951
Arnt	MA0004.1	Zipper-Type	16	29	3,712	6,66	0,00128115
IRF2	MA0051.1	Winged Helix-Turn-Helix	2	2	3,403	2,04	0,13002871
SOX9	MA0077.1	Other Alpha-Helix	20	56	2,997	6,505	0,00149594
RORA_1	MA0071.1	Zinc-coordinating	12	26	2,962	2,836	0,05865984
FOXI1	MA0042.1	Winged Helix-Turn-Helix	20	63	2,961	6,583	0,00138369
TAL1::TCF3	MA0091.1	Zipper-Type	10	20	2,823	2,522	0,08029885
NFYA	MA0060.1	Other Alpha-Helix	9	18	2,692	1,966	0,1400158
HNF1B	MA0153.1	Helix-Turn-Helix	8	10	2,669	4,384	0,01247536
Tal1::Gata1	MA0140.1	Zipper-Type	8	15	2,596	2,057	0,12783691
E2F1	MA0024.1	Winged Helix-Turn-Helix	17	31	2,475	6,452	0,00157736
SRY	MA0084.1	Other Alpha-Helix	24	125	2,317	6,405	0,00165327
Spz1	MA0111.1	Other	15	19	2,287	7,699	0,00045328
SPIB	MA0081.1	Winged Helix-Turn-Helix	28	230	2,286	5,703	0,00333594
NHLH1	MA0048.1	Zipper-Type	11	16	2,276	4,195	0,01507074
FOXA1	MA0148.1	Winged Helix-Turn-Helix	19	78	1,7	3,562	0,028382
FOXD1	MA0031.1	Winged Helix-Turn-Helix	22	78	1,661	6,626	0,00132545
FOXO3	MA0157.1	Winged Helix-Turn-Helix	20	86	1,643	4,021	0,01793502
Nr2e3	MA0164.1	Zinc-coordinating	8	17	1,57	1,783	0,16813299
NR4A2	MA0160.1	Zinc-coordinating	24	77	1,512	6,893	0,00101486
Nkx2-5	MA0063.1	Helix-Turn-Helix	26	232	1,494	5,331	0,00483923
Egr1	MA0162.1	Zinc-coordinating	14	23	1,489	4,95	0,00708341
Nkx3-2	MA0122.1	Helix-Turn-Helix	28	117	1,447	9,562	7,0352E-05

TBP	MA0108.2	Beta-sheet	15	42	1,362	3,121	0,04411303
Foxa2	MA0047.2	Winged Helix-Turn-Helix	16	53	1,313	3,064	0,04670052
MIZF	MA0131.1	Zinc-coordinating	4	5	1,298	1,819	0,16218786
Prrx2	MA0075.1	Helix-Turn-Helix	25	146	1,176	7,031	0,00088405
Pax6	MA0069.1	Helix-Turn-Helix	2	2	1,093	1,531	0,21631924
ELK1	MA0028.1	Winged Helix-Turn-Helix	23	79	0,821	5,141	0,00585183
NR3C1	MA0113.1	Zinc-coordinating	4	4	0,717	2,18	0,11304153
SPI1	MA0080.2	Winged Helix-Turn-Helix	26	122	0,676	6,098	0,00224736
PLAG1	MA0163.1	Zinc-coordinating	4	4	0,666	2,147	0,11683413
CREB1	MA0018.2	Zipper-Type	15	27	0,6	3,876	0,02073359
TEAD1	MA0090.1	Helix-Turn-Helix	7	11	0,497	1,844	0,15818343
RXR::RAR_DR!	MA0159.1	Zinc-coordinating	2	2	0,393	1,314	0,26874293
NKX3-1	MA0124.1	Helix-Turn-Helix	19	78	0,176	4,268	0,01400977
RORA_2	MA0072.1	Zinc-coordinating	6	6	0,141	2,875	0,05641614
USF1	MA0093.1	Zipper-Type	18	30	0,059	6,359	0,0017311
MYC::MAX	MA0059.1	Zipper-Type	6	6	-0,237	2,546	0,07839462
Sox5	MA0087.1	Other Alpha-Helix	21	80	-0,403	5,7	0,00334597
Foxq1	MA0040.1	Winged Helix-Turn-Helix	12	24	-0,444	3,571	0,02812771
RUNX1	MA0002.2	Ig-fold	25	69	-0,677	8,801	0,00015058
Foxd3	MA0041.1	Winged Helix-Turn-Helix	18	54	-0,843	5,052	0,00639653
ELK4	MA0076.1	Winged Helix-Turn-Helix	9	13	-0,907	1,993	0,13628595
SRF	MA0083.1	Other Alpha-Helix	1	1	-0,928	0,683	0,50509942
znf143	MA0088.1	Zinc-coordinating	2	2	-1,005	1,013	0,36312796
T	MA0009.1	Beta-Hairpin-Ribbon	2	2	-1,01	0,985	0,37343923
HOXA5	MA0158.1	Helix-Turn-Helix	27	232	-1,09	5,2	0,00551656
Stat3	MA0144.1	Ig-fold	15	28	-1,18	3,413	0,03294223
Hand1::Tcfe2a	MA0092.1	Zipper-Type	25	62	-1,291	9,575	6,9443E-05
TLX1::NFIC	MA0119.1	Helix-Turn-Helix::Other	1	1	-1,453	0,587	0,55599276
TP53	MA0106.1	Zinc-coordinating	0	0	-1,512	0	1
Pdx1	MA0132.1	Helix-Turn-Helix	25	152	-1,555	6,374	0,00170532
PPARG	MA0066.1	Zinc-coordinating	0	0	-1,589	0	1
STAT1	MA0137.2	Ig-fold	7	12	-1,619	1,163	0,31254713
Esrrb	MA0141.1	Zinc-coordinating	17	29	-1,681	4,935	0,00719046

NR1H2::RXRA	MA0115.1	Zinc-coordinating	0	0	-1,797	0	1
PBX1	MA0070.1	Helix-Turn-Helix	4	6	-1,826	0,949	0,38712796
ARID3A	MA0151.1	Helix-Turn-Helix	24	175	-1,979	4,683	0,00925122
RREB1	MA0073.1	Zinc-coordinating	2	2	-2,111	0,867	0,42021029
NFATC2	MA0152.1	Ig-fold	25	107	-2,213	6,561	0,00141447
Pax4	MA0068.1	Helix-Turn-Helix	0	0	-2,345	0	1
EWSR1-FLI1	MA0149.1	Winged Helix-Turn-Helix	0	0	-2,592	0	1
GABPA	MA0062.2	Winged Helix-Turn-Helix	17	27	-2,682	4,453	0,01164358
FEV	MA0156.1	Winged Helix-Turn-Helix	22	90	-2,977	3,047	0,04750121
ESR1	MA0112.2	Zinc-coordinating	0	0	-3,111	0	1
RXRA::VDR	MA0074.1	Zinc-coordinating	0	0	-3,126	0	1
AP1	MA0099.2	Zipper-Type	24	119	-3,19	3,965	0,01896804
Pax5	MA0014.1	Helix-Turn-Helix	1	1	-3,44	0,367	0,69280965
IRF1	MA0050.1	Winged Helix-Turn-Helix	6	10	-3,795	0,821	0,43999144
Ar	MA0007.1	Zinc-coordinating	0	0	-4,058	0	1
ELF5	MA0136.1	Winged Helix-Turn-Helix	24	128	-4,107	3,331	0,03575733
CTCF	MA0139.1	Zinc-coordinating	3	3	-4,519	0,625	0,53526143
YY1	MA0095.1	Zinc-coordinating	28	167	-4,655	6,467	0,00155388
Lhx3	MA0135.1	Helix-Turn-Helix	8	9	-4,832	2,43	0,08803683
MAX	MA0058.1	Zipper-Type	11	14	-4,86	2,027	0,13173012
MEF2A	MA0052.1	Other Alpha-Helix	7	11	-5,293	0,937	0,39180148

Supplementary Table S9: Gene lists of a venn diagram.

hlc_vs_ipsc	fetal_liver_vs_ipsc	hlc_vs_ipsc AND fetal_liver_vs_ipsc	phh_vs_ipsc	hlc_vs_ipsc AND phh_vs_ipsc	fetal_liver_vs_ipsc AND phh_vs_ipsc	hlc_vs_ipsc AND fetal_liver_vs_ipsc AND phh_vs_ipsc
AAK1	ABCC1	AACSL	A1BG	A2LD1	A2M	A1CF
ABCA4	ABCC13	ABCE1	A4GALT	AAAS	A2ML1	AADAC
ABCG4	ABHD11	ABCG2	AARS2	AACS	AAA1	AADACL1
ACER3	ABI3BP	ABLIM1	AATK	AADAT	ABAT	AAMP
ACSBG1	ACO2	ACLY	ABCA10	AARSD1	ABCA1	AARS
ACTA2	ACPT	ACOT8	ABCA11	ABCA2	ABCA6	AASDH
ADAMTS18	ACSL6	ACP6	ABCB7	ABCC10	ABCA9	AASS
ADAMTS20	ACSS1	ACPP	ABCB9	ABCF3	ABCB1	ABCA3
ADAMTS6	ACVRL1	ACTR3B	ABCC9	ABHD12	ABCB10	ABCA5
ADAMTSL5	ADAM12	ADAMTS8	ABHD2	ABHD4	ABCB11	ABCA7
ADARB1	ADCY1	ADCK4	ABI1	ABHD8	ABCB4	ABCA8
ADH5	ADCY4	ADD1	ACAA2	ACAD8	ABCB6	ABCC3
ADPGK	ADCY7	ADIPOR1	ACACA	ACAP1	ABCC11	ABCC5
AFAP1L1	ADRA2C	ADIPOR2	ACBD4	ACAP2	ABCC2	ABCD1
AFF2	AFF3	ADNP2	ACD	ACBD3	ABCC4	ABCF2
AGFG1	AFF4	ADRM1	ACSM1	ACOT11	ABCC6	ABCG1
AHDC1	ALAS2	AGA	ACTR2	ACP1	ABCC6P1	ABHD1
AHI1	ALDH16A1	AGPAT1	ACTR8	ACPL2	ABCC6P2	ABHD12B
AHSA1	ALG11	AHCYL1	ADAM15	ACRBP	ABCD3	ABHD15
AIM2	ALKBH4	AIM1L	ADAMTS2	ACTG2	ABCG5	ABHD7
AK2P2	ALMS1	AKIRIN2	ADAMTS7	ACTN4	ABCG8	ABHD9
AKAP2	AMN	ALDH7A1	ADAT1	ACTR1B	ABHD14A	ABI2
ALDH3B2	ANAPC2	ALG1L	ADAT3	ACYP1	ABHD14B	ABI3
ALG10	ANKAR	ALOX12	ADCY9	ADAL	ABHD3	ABL1
ALK	ANKLE1	AMHR2	ADCYAP1R1	ADAMTSL4	ABHD5	ABTB2
ALKBH2	ANKRD41	ANKRD10	ADH1B	ADAT2	ABHD6	ACAA1
ALOX15	ANO8	ANKRD13D	ADORA2A	ADCK5	ABLIM3	ACACB
AMBRA1	ANXA5	ANKRD16	ADPRH	ADK	ABR	ACADL
AMH	AP3D1	ANKRD20B	ADPRHL2	ADORA1	ABT1	ACADM
ANAPC7	APEX2	ANKRD38	ADRA2A	ADRA1A	ABTB1	ACADS
ANGPT2	APOBEC2	ANKRD9	ADRA2B	AGAP8	ACAD11	ACADVL
ANKHD1-EIF4I	ARFRP1	AP1S1	AES	AGL	ACAD9	ACAT1
ANKLE2	ARHGAP15	AP3S1	AFAR3	AGPAT5	ACADSB	ACBD6
ANKMY2	ARHGAP30	APPBP2	AFF1	AGPAT6	ACAT2	ACBD7
ANKRD13A	ARHGEF2	AQP1	AGGF1	AGRN	ACCS	ACE2
ANKRD26	ARHGEF5L	ARFGAP1	AGPAT3	AHCY	ACER2	ACIN1
ANKRD36	ARIH1	ARID4B	AGPS	AHR	ACHE	ACN9
ANKRD54	ARL9	ARL15	AGXT2L2	AHSA2	ACMSD	ACOT2
ANKRD6	ARRDC3	ARL3	AIDA	AKAP7	ACO1	ACOT7
ANKZF1	ASCL2	ARMCX3	AIP	AKR1A1	ACOT1	ACOT9
ANO6	ASTE1	ASAP1IT1	AIRE	AKR1B10	ACOT12	ACOX1
ANP32A	ATF2	ASCL5	AK3	AKR1B15	ACOT4	ACOX2
API5	ATP10A	ATF4	AKAP1	AKT1	ACSL4	ACOX3

APLP2	ATP5H	ATF7IP2	AKAP10	AKT1S1	ACSM2A	ACOXL
APOBEC3B	ATP6V1G1	ATG2B	AKAP11	ALAS1	ACSM2B	ACP2
APOLD1	ATP7A	ATG4B	AKAP8	ALDH3A2	ACSM5	ACP5
APRT	ATP8B4	ATG4D	AKR7A2	ALG1	ACTC1	ACSF2
AQP10	AXIN1	ATG9A	AKR7L	ALG13	ACTN1	ACSL1
ARD1A	AZU1	ATL3	ALG2	ALG3	ACTR1A	ACSL3
ARF5	B3GALT2	ATP13A2	ALG5	AMD1	ACTR3	ACSL5
ARHGAP1	BAG4	ATP2A2	ALOX5	AMOT	ACTR6	ACSM3
ARHGAP18	BAZ1B	ATP5G1	ALOXE3	ANAPC11	ACTRT1	ACSS2
ARHGAP20	BBC3	ATP6V0A1	ALPI	ANGPTL2	ACVR1	ACTA1
ARHGAP21	BCAT2	ATP6V1F	ALPK1	ANKRD17	ACVR1B	ACTB
ARHGAP27	BCL2L14	ATP9B	ALPP	ANKRD23	ACY1	ACTG1
ARHGAP29	BCL6B	ATXN1	ALS2	ANKRD37	ACY3	ACTL6A
ARHGEF11	BICD2	ATXN2L	ALS2CL	ANKRD44	ACYP2	ACTR5
ARHGEF18	BIK	ATXN7L2	ALS2CR14	ANKRD57	ADAM17	ACVR2A
ARMC8	BPI	AVEN	AMAC1L3	ANXA11	ADAMTSL2	ACVR2B
ARPC3	BRMS1L	BAGE5	AMY2B	ANXA2P3	ADAMTSL3	ADA
ARSJ	BST1	BAP1	ANAPC13	ANXA8	ADAR	ADAM10
ASB6	BTBD1	BCAR3	ANAPC4	ANXA8L2	ADD2	ADAM19
ATAD2B	BZRPL1	BCAS2	ANAPC5	AP1G1	ADD3	ADAM22
ATP10B	C10orf114	BCL2	ANGEL2	AP1G2	ADH1C	ADAM23
ATP13A3	C10orf12	BCL7C	ANKRD19	AP1S2	ADH4	ADAM9
ATP5C1	C10orf128	BCLAF1	ANKRD27	AP3B1	ADH6	ADAMTS1
ATP5S	C10orf57	BEGAIN	ANKRD29	APH1A	ADI1	ADAMTS19
ATP6AP1	C11orf21	BGN	ANKRD30B	APITD1	ADM	ADAMTS9
ATP6AP1L	C11orf49	BLOC1S2	ANKRD32	APOBEC3D	ADNP	ADAMTSL1
ATP6V1B2	C11orf71	BNIP1	ANKRD39	APOBEC3G	ADORA3	ADAP2
ATP6V1C2	C12orf41	BPGM	ANKRD40	APOL2	AFAP1	ADCK1
ATP6V1D	C13orf7	BRE	ANKRD49	AR	AGFG2	ADCY3
ATXN1L	C14orf159	BRI3BP	ANLN	ARF1	AGK	ADCY6
ATXN7L3	C16orf30	BRUNOL6	ANP32E	ARF4	AGMAT	ADCY8
AUH	C16orf38	BTG2	ANXA7	ARHGAP17	AGTR1	ADH1A
AURKC	C16orf57	BTK	AOAH	ARHGAP19	AGXT	ADHFE1
B3GALT4	C16orf88	BYSL	AP2M1	ARHGAP25	AGXT2	ADM2
B3GAT3	C17orf44	C10orf2	AP3S2	ARHGAP4	AHCYL2	ADO
B3GNT1	C17orf87	C10orf76	AP4B1	ARHGEF6	AHNAK2	ADORA2B
B3GNT5	C18orf1	C12orf66	APBA2BP	ARIH2	AHSG	ADRA1B
BAHCC1	C18orf2	C14orf100	APBB3	ARL1	AHSP	ADRB2
BAI2	C18orf45	C14orf126	APEG1	ARL4C	AIF1	ADSL
BANF1	C18orf51	C14orf138	APOL1	ARL4D	AIFM1	ADSS
BAX	C19orf22	C14orf169	APOOL	ARL6IP5	AIFM2	ADSSL1
BBS1	C19orf30	C14orf45	AQP7	ARMCX6	AK1	AEBP1
BBS12	C19orf33	C15orf28	AQP9	ARNTL	AK2	AEBP2
BCAR4	C19orf42	C16orf5	ARFGAP3	ARPC5L	AKR1C4	AEN
BCL2L2	C19orf59	C16orf87	ARHGAP11B	ARRB1	AKR1E2	AFAP1L2
BCMO1	C19orf67	C17orf101	ARHGDIA	ARRDC1	AKR7A3	AFM
BET1	C1orf131	C17orf76	ARHGEF10L	ARSB	AKT3	AFMID
BLCAP	C1orf175	C18orf21	ARID1A	ASF1A	ALCAM	AFP
BMF	C1orf186	C1orf116	ARID5A	ASH2L	ALDH18A1	AFTPH
BMP5	C1orf25	C1orf152	ARL13B	ASTN1	ALDH2	AGAP3

BMP6	C1orf31	C1orf43	ARL14	ATG16L1	ALDH3B1	AGBL5
BNC1	C1orf69	C20orf7	ARL17P1	ATG3	ALDH8A1	AGPAT2
BOC	C1orf92	C21orf59	ARL8B	ATL1	ALDOB	AGPAT4
BRCA1	C1QTNF5	C21orf91	ARMC1	ATP1A1	ALG9	AGPAT9
BRD4	C20orf12	C22orf29	ARMC5	ATP1B1	ALKBH7	AGT
BRF1	C20orf132	C2orf32	ARMET	ATP2B1	ALOX5AP	AGTPBP1
BTAF1	C20orf175	C3orf52	ARP11	ATP6V0E1	ALX1	AGTRAP
BUB3	C21orf124	C3orf70	ART1	ATPAF1	AMBP	AGXT2L1
C10orf118	C22orf28	C5orf23	ARVCF	AURKB	AMDHD2	AHNAK
C10orf41	C2orf24	C5orf25	ASB13	B9D1	AMICA1	AIF1L
C10orf61	C2orf42	C5orf30	ASIP	BACH2	AMN1	AIG1
C10orf73	C2orf64	C5orf53	ASNA1	BAD	AMT	AIMP2
C11orf10	C2orf88	C6orf134	ATG10	BAG3	AMY1A	AJAP1
C11orf46	C3AR1	C6orf72	ATG7	BANP	AMY1B	AK3L1
C11orf57	C3orf32	C6orf85	ATM	BAT2	AMY1C	AKAP12
C11orf59	C3orf58	C7orf63	ATMIN	BAT3	ANGPTL3	AKAP13
C11orf68	C5AR1	C7orf68	ATOH8	BAZ2B	ANGPTL6	AKIRIN1
C11orf75	C5orf20	C8orf33	ATP13A1	BCL3	ANK1	AKNA
C12orf52	C5orf24	C8orf38	ATP2C2	BCL7B	ANKHD1	AKR1B1
C13orf25	C5orf27	C8orf44	ATP5A1	BCL9L	ANKRA2	AKR1C2
C13orf27	C5orf45	C9orf123	ATP6AP2	BET1L	ANKRD11	AKR1C3
C14orf124	C6orf1	C9orf127	ATP6V0A2	BEX2	ANKRD13C	AKR1D1
C14orf2	C6orf182	C9orf43	ATP6V1A	BEX4	ANKRD36B	AKTIP
C14orf72	C6orf184	C9orf6	ATP8B2	BFSP1	ANKRD46	ALAD
C15orf38	C6orf81	C9orf82	ATPBD3	BHLHB2	ANKS3	ALB
C15orf39	C7	C9orf91	ATRIP	BIRC3	ANP32B	ALDH1A1
C16orf55	C7orf36	CA8	ATXN2	BIRC5	ANXA10	ALDH1A2
C16orf61	C9orf16	CAB39L	AURKA	BLZF1	ANXA13	ALDH1A3
C16orf80	C9orf30	CABLES1	AVPR2	BMP2	ANXA4	ALDH1B1
C17orf28	C9orf66	CALCA	B4GALT3	BNIPL	ANXA9	ALDH1L1
C17orf39	C9orf9	CALD1	BAHD1	BOLA2	AOF2	ALDH1L2
C17orf62	CA1	CAMK1G	BATF3	BPNT1	AP1M2	ALDH4A1
C17orf85	CADPS	CAMK2D	BCKDHA	BRD8	AP2A2	ALDH5A1
C19orf39	CALCRL	CAP2	BCL7A	BRPF3	AP3B2	ALDH6A1
C19orf47	CAMP	CAV1	BCYRN1	BTBD3	AP3M1	ALDH9A1
C19orf60	CAMSAP1	CBS	BDH2	BTBD7	APBB2	ALDOA
C1orf102	CAP1	CBX3	BECN1	BTG3	APCDD1L	ALDOC
C1orf105	CARD9	CCDC128	BEND5	BUB1	APCS	ALG10B
C1orf128	CASC5	CCDC146	BEXL1	BZW1	APLNR	ALG14
C1orf172	CBFA2T3	CCDC3	BIN3	C10orf104	APLP1	ALG6
C1orf21	CBLN4	CCDC55	BIRC6	C10orf26	APOA5	ALG8
C1orf213	CCDC147	CCDC86	BLM	C11orf35	APOC2	ALKBH1
C1orf229	CCDC26	CCK	BMS1	C11orf51	APOC4	ALKBH3
C1orf56	CCDC52	CCL2	BMS1P5	C12orf10	APOF	ALKBH5
C1orf9	CCL21	CCNDBP1	BNIP2	C12orf48	APOH	ALKBH8
C1orf93	CCL23	CCNYL1	BPHL	C12orf76	APOL3	ALOX12B
C1QTNF1	CCL8	CCRN4L	BRD1	C14orf135	APTX	ALOX15B
C20orf151	CCNO	CD1D	BRI3	C14orf153	AQP12A	ALPK2
C20orf194	CCR1	CD247	BRI3P1	C14orf167	AQP7P1	ALPL
C20orf43	CCR6	CD7	BRMS1	C14orf79	AQP7P2	ALPPL2

C21orf129	CCRL2	CD97	BSG	C14orf82	AQR	ALS2CR4
C21orf30	CD163L1	CDC14B	BSPRY	C15orf44	ARC	AMACR
C21orf71	CD1A	CDC2L6	BTN2A2	C15orf63	ARF3	AMDHD1
C21orf81	CD209	CDH15	BTN3A2	C16orf67	ARFGAP2	AMFR
C22orf9	CD276	CDH5	C10orf108	C16orf72	ARFIP1	AMMECR1
C2orf30	CD300A	CDK8	C10orf119	C16orf79	ARG1	AMPH
C2orf47	CD300C	CEBPG	C10orf137	C16orf91	ARGLU1	AMY2A
C3orf15	CD33	CECR5	C10orf21	C17orf41	ARHGAP11A	AMZ2
C3orf50	CD37	CEP250	C10orf39	C17orf42	ARHGAP23	ANAPC1
C4orf16	CD38	CETN2	C10orf4	C17orf45	ARHGAP8	ANAPC10
C4orf31	CD4	CGA	C10orf72	C17orf49	ARHGEF10	ANG
C4orf48	CD48	CH25H	C11orf24	C17orf79	ARHGEF16	ANGPT1
C4orf49	CD52	CHMP2B	C11orf31	C17orf89	ARHGEF4	ANGPTL4
C5orf15	CD53	CHORDC1	C11orf48	C18orf10	ARID2	ANK2
C5orf51	CD5L	CHST9	C11orf67	C18orf22	ARID3B	ANK3
C6orf136	CD81	CHSY3	C11orf74	C18orf55	ARID4A	ANKDD1A
C6orf203	CD84	CKLF	C11orf91	C18orf56	ARL2BP	ANKFY1
C6orf208	CDADC1	CLEC14A	C12orf28	C19orf20	ARL5A	ANKIB1
C6orf221	CDC26	CLIC3	C12orf29	C19orf28	ARL5B	ANKMY1
C7orf11	CDC42SE2	CLK4	C12orf34	C19orf62	ARL6IP4	ANKRD1
C7orf25	CDKN2D	CNIH	C12orf39	C19orf71	ARMC9	ANKRD12
C8orf40	CEACAM8	CNN3	C12orf64	C1orf109	ARMCX1	ANKRD20A1
C8orf76	CECR4	CNNM4	C12orf73	C1orf198	ARNT	ANKRD24
C9orf164	CENTG2	CNPY3	C13orf1	C1orf54	ARPC1A	ANKRD33
C9orf41	CEP170	CNTLN	C13orf18	C1orf59	ARPC2	ANKRD35
CA3	CERK	COBL	C14orf106	C1orf77	ARPP19	ANKRD43
CA5B	CES3	COL16A1	C14orf147	C20orf117	ARRDC2	ANKRD50
CACNG4	CETP	COL2A1	C14orf174	C20orf160	ARRDC4	ANKS1A
CAMK1D	CFHR4	COX15	C14orf19	C20orf24	ARS2	ANKS4B
CANX	CFP	CPE	C14orf85	C20orf27	ARSK	ANO1
CAPN14	CHML	CRAMP1L	C14orf93	C20orf4	ARV1	ANO10
CAPN6	CHRNA10	CRB1	C15orf21	C20orf94	AS3MT	ANO2
CAPS	CHRNB1	CREB3L2	C15orf48	C21orf33	ASB1	ANO4
CASP9	CINP	CRIPAK	C16orf33	C22orf27	ASCC1	ANP32C
CASQ2	CISH	CRYBB2	C16orf45	C22orf30	ASCC2	ANPEP
CBFA2T2	CLC	CSF3R	C16orf53	C2CD4B	ASF1B	ANTXR1
CBFB	CLDN2	CSMD2	C16orf63	C2orf40	ASH1L	ANTXR2
CBWD5	CLDND2	CTNNA2	C17orf37	C2orf60	ASL	ANXA1
CCAR1	CLEC10A	CTSE	C17orf67	C2orf63	ASPA	ANXA2
CCBL1	CLEC12A	CUTC	C17orf75	C2orf69	ASPG	ANXA2P1
CCDC105	CLEC1B	CXorf26	C17orf88	C3orf34	ASPH	ANXA3
CCDC111	CLEC3B	CYP11A1	C17orf90	C4orf29	ASPM	ANXA6
CCDC132	CLEC4G	CYP19A1	C17orf97	C4orf34	ASPSCR1	AOX1
CCDC134	CLEC4M	CYP1B1	C17orf98	C5orf21	ASXL2	AP1B1
CCDC35	CLIC2	CYP4Z2P	C18orf18	C5orf35	ATAD2	AP1M1
CCDC6	CLOCK	CYR61	C18orf19	C5orf54	ATCAY	AP2A1
CCDC71	CLTB	CYTH2	C18orf32	C6orf106	ATF1	AP2B1
CCDC85B	CLTCL1	DAAM1	C18orf8	C6orf145	ATF6B	AP3M2
CCDC96	CMAH	DACH1	C19orf10	C6orf162	ATF7IP	AP4E1
CCDC97	CMPK2	DAG1	C19orf24	C6orf47	ATG12	APAF1

CCNG2	CMTM2	DAGLB	C19orf29	C9orf23	ATP2B2	APBB1IP
CCNH	CMTM5	DBR1	C19orf36	C9orf25	ATP4A	APEX1
CCNT2	CNIH3	DCAF10	C19orf38	C9orf45	ATP5D	APH1B
CCR7	CNOT2	DCDC2	C19orf61	C9orf5	ATP5G3	APIP
CCT6B	CNPY2	DDX23	C19orf63	C9orf86	ATP5J	APOA1
CD55	CNPY4	DENND5A	C1GALT1C1	CA9	ATP5SL	APOA2
CD63	CNR1	DENND5B	C1orf107	CABC1	ATP6V0A4	APOA4
CDC14A	CNTFR	DGKD	C1orf123	CACNA1C	ATP6V0E2	APOB
CDGAP	COG4	DHODH	C1orf124	CACYBP	ATPAF2	APOBEC3F
CDH24	COL14A1	DHX30	C1orf144	CADM4	ATPIF1	APOC1
CDH8	COLEC10	DIO3	C1orf149	CADPS2	ATXN3	APOC3
CDK3	COMMD1	DIO3OS	C1orf151	CALCR	AURKAPS1	APOE
CDK5R1	COMMD4	DIRC2	C1orf165	CALHM3	AVPI1	APOM
CDKN2AIP	COPS2	DIXDC1	C1orf166	CAND1	AZGP1	APOO
CEACAM6	COPS6	dJ341D10.1	C1orf190	CANT1	AZI1	APP
CEP350	CORO1A	DLC1	C1orf200	CAPN11	B2M	APPL2
CERCAM	CPA3	DLGAP4	C1orf212	CAPN2	B3GALNT1	AQP11
CFDP1	CPNE8	DLK2	C1orf216	CAPNS1	B3GALNT2	AQP3
CFTR	CPOX	DNAJC25-GNG	C1orf52	CASZ1	B3GALT5	ARAP3
CGB	CR1L	DNAJC4	C1orf81	CATSPER2	B3GNTL1	ARCN1
CGB7	CREBL2	DNAJC5	C1orf85	CBLB	B4GALT6	ARFGEF1
CGB8	CSAG1	DNASE1L1	C1orf89	CBLL1	B9D2	ARG2
CHCHD7	CSH1	DNHD1	C20orf11	CBWD3	BAAT	ARHGAP10
CHD2	CSH2	DNLZ	C20orf196	CBY1	BAG5	ARHGAP12
CHD6	CST7	DOCK5	C20orf45	CCDC125	BAIAP2L1	ARHGAP22
CHERP	CTGLF1	DOCK8	C20orf62	CCDC21	BAK1	ARHGAP24
CHKB	CTSG	DPM1	C21orf119	CCDC23	BAT2D1	ARHGAP28
CHST11	CUL4A	DPY19L1	C21orf126	CCDC24	BAZ1A	ARHGAP9
CHST14	CX3CR1	DPY19L2P2	C21orf24	CCDC49	BBOX1	ARHGDIB
CIAO1	CXADR	DSC2	C21orf55	CCDC66	BBS10	ARHGEF1
CISD1	CXCR4	DSC3	C21orf56	CCDC8	BBS2	ARHGEF12
CIZ1	CYBB	DST	C2orf18	CCDC88A	BBS4	ARHGEF17
CKAP4	CYGB	DUSP5	C2orf43	CCDC93	BCAP29	ARHGEF19
CLCN6	CYorf14	DUSP9	C2orf44	CCND1	BCHE	ARHGEF3
CLDN9	CYTH4	DVL3	C2orf89	CCNE2	BCL2L10	ARHGEF5
CLEC1A	DAAM2	DYRK2	C3orf19	CD320	BCL2L11	ARHGEF9
CLEC4D	DARC	E2F3	C3orf36	CD44	BCL9	ARID3A
CLN6	DCDC5	ECSCR	C3orf37	CD46	BDH1	ARID5B
CLPP	DCHS1	EFNB1	C3orf39	CD72	BDNF	ARL16
CLSTN2	DCUN1D1	EGFL7	C3orf41	CDA	BFAR	ARL17B
CMC1	DCUN1D2	EGR2	C4A	CDC14C	BHMT	ARL2
CNTN4	DDX5	EIF2C3	C4B	CDC2	BHMT2	ARL4A
CNTROB	DEFA1	EIF2S2	C4orf32	CDC20	BID	ARL6
COG1	DEFA1B	EIF3CL	C4orf38	CDC25A	BLOC1S1	ARL6IP6
COL11A2	DEFA3	EIF4A1	C5orf22	CDC25B	BLVRB	ARMC10
COL15A1	DEFA4	EIF4G2	C5orf39	CDC40	BMP2K	ARMC4
COL18A1	DEPDC4	EIF5A	C5orf43	CDC42BPB	BMPER	ARMC6
COL8A2	DERL2	ELF4	C5orf46	CDC42EP1	BMX	ARMC7
COLQ	DFFB	ELL3	C6orf122	CDC45L	BNC2	ARMCX2
COMMD5	DGCR8	ELMOD2	C6orf130	CDCA8	BNIP3L	ARMCX4

COPA	DHFR	EML2	C6orf138	CDH10	BOAT	ARNT2
COPB1	DHFRL1	EMP1	C6orf142	CDH11	BOLA3	ARPC1B
COPB2	DHRS13	EPB41	C6orf165	CDIPT	BPTF	ARPC4
COPG2IT1	DHX29	EPDR1	C6orf167	CDK2	BRAF	ARSA
COPS3	DKFZP564O05	EPS8L1	C6orf222	CDK4	BRD3	ARSD
COPS5	DLEU2	ERCC1	C6orf225	CDK9	BRD7	ARSE
CORO1C	DNAJA2	ERP29	C6orf26	CDKN1C	BRD7P2	ARSG
CORO7	DNAJB11	ESPL1	C6orf62	CDKN2B	BRD9	ART3
COX4NB	DNASE1L3	EVC	C6orf64	CDKN2C	BRP44L	ART4
CPA4	DOCK10	EVI1	C6orf70	CDKN3	BRSK1	ART5
CREBBP	DOCK6	EYA2	C7orf13	CDRT4	BRSK2	ARTN
CRH	DOK2	FAM101A	C7orf29	CDT1	BRWD1	ASAH1
CRKL	DPEP2	FAM120B	C7orf49	CECR1	BRWD2	ASAM
CRNKL1	DPT	FAM125B	C7orf58	CECR7	BRWD3	ASAP1
CRSP9	DPYSL5	FAM150B	C7orf59	CENPA	BSCL2	ASAP2
CRYBA1	DRAP1	FAM178A	C7orf65	CENPL	BSDC1	ASB3
CSGALNACT1	DSCR10	FAM179B	C8orf37	CENPM	BSN	ASB7
CSN1S1	DSCR3	FAM20B	C8orf45	CENPN	BTBD16	ASB8
CSPP1	DTNB	FAM80A	C8orf46	CENPO	BTBD2	ASB9
CST4	DUSP13	FAM90A2P	C9	CENPP	BTN3A3	ASCC3
CTAGE5	DUSP15	FAM92A1	C9orf10OS	CENPV	BVES	ASGR1
CTNNAL1	DUSP21	FANCF	C9orf114	CENTG3	C10orf11	ASGR2
CTPS	DYNLT1	FBXL13	C9orf119	CEP135	C10orf116	ASMTL
CUBN	DYRK4	FBXL18	C9orf130	CEPT1	C10orf65	ASNS
CUL1	E2F4	FBXO18	C9orf167	CGB1	C10orf68	ASPHD1
CUL7	E2F6	FBXO2	C9orf38	CGB5	C10orf82	ASPHD2
CUL9	E2F8	FBXO38	C9orf7	CGGBP1	C10orf88	ASRGL1
CUX1	ECSIT	FBXO44	C9orf75	CGI-96	C11orf17	ASS1
CXCR6	EDEM3	FBXW7	C9orf89	CGN	C11orf45	ASTN2
CXorf40B	EEF1D	FCHO2	CA13	CGNL1	C11orf60	ASXL1
CXorf56	EGLN3	FCHSD2	CABIN1	CHAC2	C11orf61	ATAD1
CXXC5	EHD3	FIP1L1	CADM3	CHD8	C11orf70	ATE1
CYB5D1	EIF1AY	FJX1	CALN1	CHMP1A	C11orf84	ATF3
CYC1	EIF2C2	FLJ10996	CAMK2B	CHMP4B	C12orf30	ATF5
CYHR1	EIF4EBP2	FLJ20489	CAMKK2	CHPF	C12orf35	ATF6
CYP46A1	ELANE	FLJ20628	CAMTA1	CICE	C12orf4	ATG2A
DAGLA	ELN	FMOD	CAMTA2	CIDEA	C12orf43	ATG4A
DCAKD	ELOVL7	FNTA	CAPN5	CKAP2	C12orf49	ATG4C
DCLK2	ELP2P	FOXA3	CASC4	CKS2	C12orf57	ATG5
DCTD	ELP3	FOXC1	CASD1	CLDN19	C12orf62	ATHL1
DCX	EMCN	FOXI3	CBR4	CLIP1	C14orf105	ATIC
DDA1	EMID1	FOXJ2	CBX5	CLN5	C14orf131	ATN1
DDAH1	EMILIN1	FYB	CC2D1B	CLUAP1	C14orf132	ATOX1
DDAH2	ENG	FZD4	CC2D2A	CMAS	C14orf133	ATP11B
DDX24	ENO1	GAA	CCDC103	CMIP	C14orf143	ATP11C
DEFA6	ENTPD1	GAB1	CCDC106	CMTM6	C14orf145	ATP12A
DERA	EPB42	GAB2	CCDC107	COG6	C14orf166	ATP1A2
DFNA5	ERCC6	GABPB1	CCDC121	COL13A1	C14orf179	ATP1B2
DFNB31	ERGIC2	GAPVD1	CCDC13	COL4A1	C14orf4	ATP1B3
DGCR6	ERI2	GAR1	CCDC130	COL6A1	C14orf68	ATP2B4

DIP2B	ERICH1	GAS6	CCDC14	COL6A2	C14orf73	ATP2C1
DIP2C	EXD3	GATA5	CCDC15	COL9A1	C14orf78	ATP5E
DIRAS2	EXOD1	GDPD3	CCDC150	COLEC12	C15orf41	ATP5EP2
DISP1	F13A1	GDPD5	CCDC17	COMTD1	C15orf42	ATP5F1
DKFZP434L18	F2RL1	GNB1L	CCDC25	COPG	C15orf52	ATP5G2
DKK1	FAAH2	GNL3L	CCDC47	COPS4	C16orf13	ATP5I
DLD	FABP3	GNS	CCDC53	COQ2	C16orf50	ATP5J2
DMPK	FAIM3	GOLGA1	CCDC72	COQ6	C16orf52	ATP5L
DMRT1	FAM109B	GOLGA2	CCDC76	CORO2B	C16orf62	ATP5O
DNAH2	FAM111A	GPBAR1	CCDC81	CPD	C16orf7	ATP6V0B
DNAJC13	FAM113A	GPC5	CCDC88B	CPNE1	C16orf70	ATP6V0C
DNAJC14	FAM117A	GPRC5A	CCDC88C	CPNE3	C16orf73	ATP6V0D1
DNAJC17	FAM129C	GREM1	CCDC94	CREB1	C16orf86	ATP6V1B1
DNAJC19	FAM171A1	GRRP1	CCL13	CRELD1	C16orf93	ATP6V1E2
DNAJC7	FAM178B	GTF3C6	CCL16	CRELD2	C17orf106	ATP6V1G2
DOC2A	FAM188A	GTPBP4	CCNB1	CRYM	C17orf48	ATP6V1H
DOCK9	FAM22A	GUCY1A3	CCNL2	CSNK1G1	C17orf71	ATP7B
DPP3	FAM22D	H3F3B	CCNY	CSPG4	C18orf54	ATP8B3
DSC1	FAM26F	HACL1	CCR2	CSTB	C19orf43	ATP9A
DUOX2	FAM53B	HAND2	CCT5	CTCF	C19orf50	ATPBD1B
DUOXA2	FAM55C	HCG18	CCT8	CTDP1	C1GALT1	ATPBD4
DUSP18	FAM73B	HCN3	CD300E	CTDSPL	C1orf112	ATRN
DUSP5P	FAM76A	HEBP1	CD40	CTGLF3	C1orf162	AUTS2
DVL2	FAM82A2	HECTD1	CD83	CTSF	C1orf168	AVL9
DYNC1LI2	FAM83F	HES2	CDC34	CUGBP2	C1orf26	AVP
DYNLRB2	FAM90A6P	HES4	CDC37L1	CUTL1	C1orf41	AXIN2
DYRK1A	FAM99A	HIST1H4K	CDC42EP2	CXCL14	C1orf53	AXL
EAPP	FAM9A	HIST3H3	CDCA2	CXCR7	C1orf55	AXUD1
ECE1	FBXL12	HLA-DMA	CDH2	CYP2U1	C1orf96	AYP1p1
EDC4	FBXL17	HLA-DMB	CDH20	CYP4X1	C1QA	AZIN1
EEA1	FBXO16	HNRNPAB	CDH6	CYTH3	C1QB	B3GALT6
EFCAB3	FBXO9	HNRNPC	CDK10	DACH2	C1QC	B3GAT1
EI24	FBXW11	HNRNPM	CDKL2	DACT3	C1QL2	B3GNT2
EIF3J	FCER1A	HNRPM	CDNF	DAD1	C2	B3GNT6
EIF4ENIF1	FCGBP	HOPX	CDV3	DAPK1	C20orf111	B4GALNT4
EIF4H	FCGR2B	HOXA6	CDYL	DCAF7	C20orf127	B4GALT1
ELAVL3	FCN1	HOXB5	CDYL2	DCHS2	C20orf177	B4GALT4
ELF2	FCN2	HP1BP3	CENPI	DCP1A	C20orf20	BACE1
ELMOD1	FCRL2	HPS1	CEP110	DCPS	C20orf54	BACE2
ELOVL5	FCRLA	HSD17B1	CEP120	DCTN1	C21orf2	BAIAP2
ENOX1	FDXR	HSD17B14	CEP164	DCTPP1	C21orf34	BAIAP2L2
ENPP1	FECH	HSGT1	CEP192	DDEF2	C21orf57	BAMBI
ENY2	FEM1B	HTR2A	CEP27	DDHD2	C21orf66	BAPX1
EPHB3	FGD3	HTRA2	CEP290	DDX12	C21orf7	BARD1
EPM2A	FKBPL	HYLS1	CES1	DDX17	C22orf36	BARX1
ERBB2	FLI1	IER2	CES4	DDX39	C2orf37	BASP1
ERGIC3	FLII	IFI16	CFHR3	DEF6	C2orf49	BAT1
ERVWE1	FLJ11827	IFRD1	CFHR5	DEF8	C2orf7	BAT2L
ESD	FLJ23152	IFT122	CFL2	DEFB1	C3	BAT5
ETV7	FLJ23834	IL12A	CHAD	DEK	C3orf18	BBS7

EWSR1	FLJ31945	ING5	CHADL	DENND2C	C3orf21	BBS9
EXOC5	FLJ32255	INHBE	CHCHD10	DENND4B	C3orf25	BBX
EXOC7	FLJ33996	IP6K2	CHCHD5	DEPDC1B	C3orf31	BCAM
EXOSC2	FLJ36070	IPO13	CHD1	DEPDC5	C3orf46	BCAN
EXPH5	FLJ39827	IRX3	CHKA	DEPDC7	C3orf54	BCAR1
EYA1	FLJ41170	ITGB4	CHMP1B	DERL1	C3orf59	BCAS4
FADD	FLJ41603	JAK2	CHRDL2	DGCR14	C3orf60	BCAT1
FAF2	FLJ42627	JAZF1	CHRNA2	DHDDS	C4BPA	BCCIP
FAM100B	FLJ44290	JMJD7	CHST1	DHH	C4BPB	BCKDHB
FAM108A2	FLJ44606	JPH4	CHST10	DHPS	C4orf14	BCL10
FAM10A4	FLYWCH1	KCMF1	CHTF8	DHRS11	C4orf33	BCL11A
FAM118A	FOLR2	KCNJ16	CIAPIN1	DHTKD1	C4orf41	BCL11B
FAM119B	FOXC2	KCNJ4	CIDEC	DHX32	C5orf13	BCL2L1
FAM156A	FOXD4	KCNN4	CLCN3	DHX37	C5orf32	BCL2L12
FAM156B	FOXO1	KCNT2	CLDN20	DHX40	C5orf33	BCL6
FAM158A	FOXS1	KCTD1	CLDN23	DICER1	C6	BCOR
FAM160B2	FPR1	KCTD3	CLEC4A	DKFZP586I142	C6orf108	BCORL1
FAM168B	FPR3	KDELR2	CLIP2	DLEU1	C6orf111	BCR
FAM173B	FREQ	KHNYN	CLIP4	DLST	C6orf115	BEND3
FAM175B	FTSJ3	KIAA0430	CLMN	DMD	C6orf124	BEND4
FAM183A	GAB3	KIAA0892	CLN8	DNAJA3	C6orf132	BEND6
FAM193A	GABARAPL2	KIAA0922	CLRN1	DNAJB14	C6orf141	BEND7
FAM19A3	GALNT2	KIAA1143	CLTC	DNAJC28	C6orf173	BEST1
FAM20A	GALNT5	KIAA1345	CLU	DNAJC3	C6orf192	BEST2
FAM23B	GAPT	KIAA1539	CNDP1	DNASE1	C6orf52	BEST4
FAM39DP	GAS2L1	KIAA1641	CNGB1	DNMT3L	C7orf23	BEX1
FAM47E	GATA1	KIFAP3	CNNM2	DOHH	C7orf31	BEX5
FAM49A	GDF1	KLHDC8B	CNOT10	DOLK	C7orf38	BHLHB9
FAM53A	GDF15	KLHL17	CNOT8	DOPEY1	C7orf55	BICD1
FAM57B	GDF5OS	KLRAQ1	COBRA1	DOPEY2	C8A	BIN1
FAM62B	GFI1B	KPNA6	COG3	DPEP1	C8B	BIRC2
FAM72B	GGT5	KRCC1	COMMD8	DPH2	C8G	BLMH
FARS2	GGT7	KRT7	COPZ2	DPP10	C8orf41	BLVRA
FASTKD2	GGTL3	KRTCAP2	COQ10B	DPP8	C8orf42	BMI1
FASTKD3	GIMAP1	KRTCAP3	CORO6	DPY19L4	C8orf47	BMP1
FBXL21	GIMAP4	LARP7	COX16	DRAM1	C8ORFK36	BMP4
FDX1L	GIMAP5	LASS4	COX19	DSP	C9orf100	BMP7
FHIT	GIMAP6	LATS2	COX4I1	DTL	C9orf102	BMPR1A
FIBCD1	GIMAP7	LCOR	COX5B	DUS3L	C9orf126	BMPR2
FKRP	GIMAP8	LEF1	COX6C	DUSP1	C9orf150	BNIP3
FLJ11235	GIYD1	LEMD1	COX7A2	DUSP19	C9orf156	BOK
FLJ16779	GJA4	LGALS2	COX7A2L	DUSP22	C9orf21	BOP1
FLJ20444	GJC2	LIN54	COX7C	DUSP23	C9orf37	BP75
FLJ20718	GJC3	LITAF	CPLX1	DUSP3	C9orf64	BRIX1
FLJ22222	GKN2	LLGL2	CPPED1	DUSP8	C9orf78	BRP44
FLJ22639	GLCCI1	LOC10000858	CPSF1	DUT	C9orf93	BST2
FLJ23584	GLRX5	LOC10000858	CRB3	DYNC1I2	CA5A	BTBD10
FLJ31306	GLUD2	LOC10012820	CRCP	DYNLL2	CABP1	BTBD11
FLJ31568	GLUL	LOC10012868	CRCT1	DZIP3	CACNA2D3	BTBD12
FLJ35429	GMIP	LOC10012878	CRHR1	EAF2	CACNA2D4	BTD

FLJ35776	GNA15	LOC10012889	CRKRS	EBF3	CACNG1	BTF3
FLJ41327	GNB1	LOC10012990	CROCCL2	EBF4	CACNG6	BTF3L4
FLJ44313	GOSR2	LOC10013018	CRYAA	EBPL	CALY	BTG1
FLJ45422	GP1BA	LOC10013036	CSAG3	ECE2	CAMLG	BTN3A1
FLJ45983	GP6	LOC10013116	CSAG3A	ECHDC1	CAPG	BTRC
FLJ46552	GP9	LOC10013138	CSAG3B	ECHDC3	CAPZB	BUB1B
FLJ90757	GPD1L	LOC10013164	CSF1	EDC3	CARM1	BUD31
FN1	GPD2	LOC10013172	CSTF2	EDNRA	CARS2	BZW2
FNBP1	GPR124	LOC10013208	CTBP1	EEF1A1	CASC3	C10orf10
FNBP4	GPR172A	LOC10013228	CTBS	EEF1G	CASP1	C10orf125
FNDC3B	GPR180	LOC10013228	CTDSP2	EFTUD2	CASP2	C10orf140
FOXF1	GPR34	LOC10013239	CTNNBL1	EGFR	CBX1	C10orf32
FOXR1	GPR4	LOC10013241	CTNND1	EGLN1	CBX6	C10orf33
FOXRED1	GPR44	LOC10013360	CTR9	EGLN2	CCDC101	C10orf35
FOXRED2	GPR65	LOC10013380	CTRB1	EHBP1	CCDC113	C10orf47
FTO	GPT2	LOC10013392	CTSK	EHD2	CCDC123	C10orf54
FTSJD2	GRAP2	LOC10013436	CUL2	EIF2AK3	CCDC151	C10orf58
FUBP3	GRASP	LOC10013440	CUL5	EIF2C4	CCDC45	C10orf59
FUCA2	GSK3B	LOC10017093	CWF19L1	EIF4EBP3	CCDC64B	C10orf6
FZD1	GSTM2	LOC143666	CXCL13	EIF4G1	CCDC68	C10orf75
G3BP1	GSTM5	LOC152195	CXCL2	EIF5	CCDC69	C10orf78
GABRB1	GTSF1	LOC220686	CXorf12	ELAVL1	CCDC84	C10orf96
GABRE	GUCY2E	LOC283683	CXorf40A	ELMO1	CCL14	C11orf52
GALIG	GYPA	LOC283932	CYB561D2	EML3	CCL15	C11orf54
GALNT1	GYPB	LOC285016	CYB5RL	ENC1	CCL20	C11orf58
GAN	GYPE	LOC285296	CYLD	ENOX2	CCL3	C11orf63
GAS2L3	GZMA	LOC285943	CYP1A2	ENPEP	CCL3L1	C11orf73
GATAD2B	GZMK	LOC286016	CYP2A6	ENTPD6	CCL3L3	C11orf80
GATS	H2AFV	LOC340598	CYP2A7	EOMES	CCL4L1	C11orf82
GDAP1	HABP4	LOC345645	CYP2B6	EPB41L1	CCL4L2	C11orf9
GDE1	HAPLN4	LOC346085	CYP2B7P1	EPB41L2	CCM2	C12orf11
GDF6	HARBI1	LOC388312	CYP2C18	EPB41L3	CCND2	C12orf23
GFM1	HBA1	LOC388481	CYP2C19	EPB41L4B	CCNF	C12orf24
GFM2	HBA2	LOC388556	CYP2C9	EPHA4	CCNG1	C12orf27
GGA1	HBB	LOC389599	CYP2D7P1	EPPB9	CCNI	C12orf31
GGTLC1	HBBP1	LOC389641	CYP3A4	EPR1	CCNJL	C12orf32
GINS1	HBD	LOC399491	CYP4B1	EPS8	CCNK	C12orf44
GIPC1	HBE1	LOC400558	CYP4F11	ERMP1	CCNL1	C12orf45
GJA5	HBG1	LOC400986	CYP4F22	ERN1	CCRK	C12orf47
GLB1	HBM	LOC401052	CYP7A1	ESRRG	CCT4	C12orf5
GLI3	HBZ	LOC401233	CYSLTR2	ETS1	CD302	C12orf51
GLS	HCFC1	LOC402377	CYTSA	ETS2	CD58	C12orf56
GLTSCR1	HCG27	LOC440122	CYYR1	ETV6	CD59	C12orf60
GM2A	HDC	LOC440157	DACT2	EXO1	CD74	C13orf15
GNAS	HDDC3	LOC440353	DAK	EXOC3	CD79A	C13orf23
GNG7	HEBP2	LOC440354	DAO	EXOSC3	CD82	C13orf3
GOLGA3	HEMGN	LOC441124	DAPK2	EXT1	CD86	C13orf33
GOLGA5	HEPACAM2	LOC441131	DAPP1	FABP7	CD93	C13orf34
GOLGA7	HES5	LOC493754	DARS2	FAHD2A	CD99L2	C13orf37
GOLGA8B	HEXA	LOC552889	DAXX	FAM101B	CDC23	C14orf102

GPR101	HEXB	LOC641823	DC36	FAM102A	CDC42EP5	C14orf104
GPR137	HEYL	LOC642127	DCAF15	FAM113B	CDCA3	C14orf109
GPR162	HHIP	LOC642361	DCAF4L1	FAM120AOS	CDH1	C14orf115
GPR61	HIF3A	LOC642393	DCBLD1	FAM122B	CDH13	C14orf118
GPR87	HIST1H2BH	LOC642615	DCLRE1A	FAM126B	CDH17	C14orf129
GRAMD1B	HIST1H3F	LOC642678	DCLRE1B	FAM127B	CDK7	C14orf139
GRAMD2	HIST1H3G	LOC642784	DCTN5	FAM127C	CDKN1A	C14orf149
GRAMD3	HIST1H3H	LOC642921	DCTN6	FAM129B	CDKN2AIPNL	C14orf173
GRB2	HIST1H4E	LOC643507	DCUN1D3	FAM133B	CDR2	C14orf176
GRINL1A	HIST1H4H	LOC643624	DDTL	FAM160B1	CDX1	C14orf37
GRTP1	HIVEP3	LOC643668	DDX1	FAM172A	CECR6	C14orf43
GRWD1	HK3	LOC643856	DDX20	FAM176B	CELSR2	C14orf80
GTF2A2	HLA-DQA1	LOC643949	DDX27	FAM189B	CELSR3	C15orf23
GTF2I	HLA-DRB6	LOC644012	DDX42	FAM190B	CENPE	C15orf27
GTF3A	HMOX2	LOC644330	DDX46	FAM72D	CENPF	C15orf33
GTF3C5	HOXA2	LOC644589	DDX52	FAM73A	CENPJ	C15orf57
HARS2	HOXA5	LOC644684	DDX55	FAM83H	CENTB2	C16orf11
HDAC11	HOXD11	LOC644774	DDX60	FAM86B1	CEP152	C16orf35
HELQ	HRAS	LOC644860	DEPDC1	FAM86C	CEP55	C16orf48
HELZ	HSD11B2	LOC644931	DERPC	FAM96A	CEP70	C16orf59
HERV-FRD	HSPB6	LOC644935	DET1	FBXL6	CES2	C16orf68
HES6	HSPC159	LOC645086	DEXI	FBXO32	CES8	C16orf74
HIST1H4J	HYOU1	LOC645159	DGCR11	FBXO33	CFH	C16orf75
HIST3H2A	ICK	LOC645233	DGUOK	FBXO5	CFHR1	C17orf100
HLA-A29.1	IFI44L	LOC645367	DHDH	FBXW8	CFHR2	C17orf53
HNRNPA3P1	IFIT1L	LOC645558	DHRSX	FCAR	CFI	C17orf58
HNRPK	IFT20	LOC645733	DHX34	FER1L4	CHAF1B	C17orf59
HOOK3	IFT74	LOC645969	DHX57	FGFR1OP2	CHD3	C17orf61
HOXB3	IGLL1	LOC647009	DHX58	FGFR3	CHD7	C17orf63
HOXB4	IGLL3	LOC647012	DIRAS3	FGFRL1	CHES1	C17orf68
HOXB7	IGSF6	LOC647081	DIS3	FHL1	CHFR	C17orf69
HOXC4	IGSF9B	LOC647340	DIS3L2	FICD	CHIC2	C17orf80
HOXC8	IKZF1	LOC647349	DKFZp434K19	FIGNL1	CHMP4C	C17orf91
HS2ST1	IL10RA	LOC648024	DKFZp434M1[illegible]	FILIP1L	CHP	C17orf93
HS3ST1	IL13RA2	LOC648434	DKFZp451M2[illegible]	FKBP5	CHRFAM7A	C17orf95
HSBP1	IL21R	LOC648476	DKFZp686I15[illegible]	FLJ10213	CHRM3	C17orf96
HSD3B1	IL2RB	LOC648608	DKFZp686K16	FLJ10374	CHST13	C18orf26
HSPB2	IL33	LOC648984	DKFZp686O24	FLJ10986	CHST15	C19orf12
HSPB7	IL7R	LOC649841	DKFZP779L18[illegible]	FLJ11783	CHSY1	C19orf2
HSPG2	IL8RB	LOC650020	DKFZp779M0[illegible]	FLJ14213	CHTF18	C19orf4
HTR1E	IL8RBP	LOC650128	DMAP1	FLJ20021	CHURC1	C19orf48
HTRA4	IMPA1	LOC650515	DMXL2	FLJ21839	CICK0721Q.1	C19orf54
HTT	ING1	LOC651143	DNAJB4	FLJ35220	CLCC1	C19orf56
HYAL2	INSC	LOC651309	DNAJC1	FLJ35801	CLCF1	C19orf6
IAPP	INTS10	LOC652595	DNAJC27	FLJ41484	CLCN5	C19orf66
IDH3B	IP6K3	LOC652676	DNCL1	FLJ44124	CLDN3	C19orf70
IGF2AS	ISG15	LOC652755	DNM3	FLJ45032	CLDN5	C1orf104
IGF2R	ITGA2B	LOC652826	DNMT1	FLJ46309	CLDN7	C1orf106
IHH	ITGA4	LOC652903	DNPEP	FNDC3A	CLDND1	C1orf115
IKBIP	ITGAD	LOC653108	DOCK7	FOXN4	CLEC11A	C1orf122

IL17C	ITGB3	LOC653171	DPF1	FOXP4	CLRN3	C1orf133
ILDR2	ITLN1	LOC653308	DSEL	FREM1	CMBL	C1orf135
INHA	ITPK1	LOC653496	DSG1	FRZB	CMTM7	C1orf156
INMT	KCNK1	LOC653764	DUS2L	FSIP1	CMTM8	C1orf163
INPP5E	KCNK4	LOC653930	DUXA	FSTL3	CNIH2	C1orf182
INPP5K	KCNMB1	LOC727773	DUXAP3	FTHL7	CNO	C1orf187
INSL4	KCNU1	LOC728098	DYM	FUT6	CNOT1	C1orf188
INSM1	KEL	LOC728188	DYNLL1	FXR2	CNOT3	C1orf19
INVS	KIAA0240	LOC728428	DYNLT3	FXYD6	CNOT6	C1orf203
IRAK1	KIAA0319L	LOC728484	DYSFIP1	FYTTD1	COIL	C1orf218
IRF3	KIAA0649	LOC728537	EAF1	FZD9	COL11A1	C1orf24
ISM2	KIAA0748	LOC728661	EDAR	GALK2	COL17A1	C1orf51
ITGA11	KIAA0831	LOC728844	EDF1	GALNS	COL27A1	C1orf57
ITGA8	KIAA0895L	LOC728855	EEF2K	GALNT11	COL7A1	C1orf61
ITGB6	KIAA1383	LOC729004	EFHD1	GALNT6	COL9A2	C1orf63
ITIH5	KIAA1409	LOC729231	EGFL6	GALNTL4	COL9A3	C1orf74
ITPA	KIAA1571	LOC729298	EGOT	GAS1	COLEC11	C1orf86
JMJD2A	KIAA1737	LOC729581	EHMT2	GATA2	COMMD2	C1orf88
KANK3	KIAA1908	LOC729602	EID3	GATA6	COMT	C1orf94
KATNB1	KIF6	LOC729680	EIF1AD	GBA2	COPG2	C1orf97
KAZALD1	KIFC3	LOC729841	EIF2AK2	GCC1	COPS7B	C1QBP
KBTBD10	KIR2DS5	LOC729858	EIF2AK4	GCM1	COPZ1	C1QL1
KBTBD2	KIR3DL1	LOC730012	EIF2B5	GCSH	COQ9	C1QL4
KCNJ13	KIR3DL2	LOC730052	EIF2C1	GINS2	CORO1B	C1QTNF6
KCNK13	KISS1R	LOC730153	EIF2S1	GINS3	CORO2A	C1R
KCNK17	KLF1	LOC730273	EIF3D	GJB6	COX10	C1RL
KCNK9	KLK1	LOC731878	EIF4B	GLDN	COX11P	C1S
KCNQ1OT1	KLK10	LOC732007	ELAC1	GLE1	COX17	C20orf100
KCNQ4	KLRB1	LOC732165	ELMOD3	GLO1	COX6A2	C20orf103
KCTD20	KPNA4	LOC732360	ELOVL6	GMNN	COX7A1	C20orf108
KCTD6	KPTN	LOC732425	EML4	GMPS	COX7B	C20orf199
KIAA0040	KRT1	LOC88523	ENDOG	GNB4	COX8A	C20orf201
KIAA0100	KRT13	LOC91316	ENTPD7	GNPAT	CP	C20orf29
KIAA0182	KRT6C	LOC96610	ENTPD8	GOLGA8A	CP110	C20orf3
KIAA0195	L3MBTL2	LPPR4	EP300	GOLGB1	CPB2	C20orf46
KIAA0460	LAT	LRFN3	EPC1	GON4L	CPEB2	C20orf52
KIAA0556	LCE1E	LRRC17	EPHA3	GORASP1	CPEB3	C20orf55
KIAA0753	LCN1L1	LRRC2	EPHA6	GOT1	CPEB4	C20orf56
KIAA1024	LDHAL6A	LRRC23	EPHB4	GPR125	CPSF3	C20orf72
KIAA1045	LGALS14	LRRC34	EPN2	GPR137B	CPSF3L	C20orf75
KIAA1107	LGALS9	LRRC38	EPS15L1	GPR89B	CPSF4	C21orf45
KIAA1199	LHX2	LTV1	ERLIN2	GPR89C	CPT2	C21orf51
KIAA1202	LHX4	LY6H	ERO1LB	GPRASP2	CPVL	C21orf58
KIAA1407	LILRA2	LYPLAL1	ERRFI1	GPRC5C	CREB3	C21orf63
KIAA1429	LILRA3	LYST	ESM1	GPSM3	CREBZF	C21orf70
KIAA1522	LILRA5	MAF1	ESPNL	GPX1	CRHBP	C22orf13
KIAA1549	LILRA6	MAGED1	ESR1	GRAMD4	CRIM1	C22orf25
KIAA1772	LILRB2	MAGEH1	ESRRA	GREM2	CRIP2	C2CD2
KIDINS220	LILRB4	MAK16	ESRRAP2	GRHL1	CRISPLD1	C2orf15
KIF26B	LILRB5	MANSC1	ESYT1	GRM2	CRLF3	C2orf25

KLHDC4	LOC10012792	MAP3K13	ETFA	GRM3	CRLS1	C2orf28
KLHL24	LOC10012795	MAP3K9	ETHE1	GRPEL1	CROCC	C2orf34
KLHL26	LOC10012818	MAP7D3	ETV2	GSC	CRP	C2orf56
KLK11	LOC10012848	MAPK6	EVI5	GSTA4	CRYGS	C2orf65
KLK3	LOC10012854	MAPK7	EVI5L	GTF2H5	CS	C2orf68
KLK7	LOC10012869	MAPK8IP1	EXOC8	GTF2IRD2B	CSDC2	C2orf82
KLK8	LOC10012874	MARS2	EXOSC10	GTF3C1	CSHL1	C3orf10
KRBA2	LOC10012890	MCAM	EXOSC6	GVIN1	CSNK1A1L	C3orf14
KRT17P3	LOC10012897	MCTS1	F2RL3	H1F0	CSNK1G3	C3orf23
KRT18P19	LOC10012900	MDH1	FAAH	H2AFZ	CSNK2A2	C3orf26
KRT18P26	LOC10012909	MECP2	FADS3	HARS	CSRNP2	C3orf38
KRT18P30	LOC10012911	MED10	FAM100A	HAT1	CSRP1	C3orf64
KRT18P34	LOC10012920	MED12	FAM102B	HDAC7	CSRP2BP	C3orf67
KRT20	LOC10012953	MEIS1	FAM103A1	HDAC7A	CTDSPL2	C3orf71
KRT23	LOC10012965	MEIS2	FAM114A1	HDHD2	CTNNB1	C3orf72
KRTAP10-5	LOC10012967	MERTK	FAM115A	HEATR2	CTNND2	C3orf75
KRTAP19-6	LOC10012975	MESDC1	FAM118B	HEATR3	CTSD	C4orf18
KRTAP21-1	LOC10013000	METTL11A	FAM128A	HEATR5B	CTSS	C4orf19
KRTAP21-2	LOC10013025	METTL11B	FAM139A	HECA	CTTNBP2NL	C4orf27
KSR1	LOC10013031	MEX3A	FAM153B	HEG1	CTU2	C4orf43
LAG3	LOC10013049	MFSD6	FAM164C	HERPUD1	CWF19L2	C4orf46
LARP4B	LOC10013055	MGC3032	FAM174A	HEY1	CX3CL1	C4orf51
LASP1	LOC10013059	MGC3731	FAM175A	HHAT	CXCL10	C5
LDHC	LOC10013074	MGC45491	FAM186B	HHLA1	CXCL16	C5orf28
LGALS13	LOC10013082	MGC5457	FAM18B	HIAT1	CXorf23	C5orf34
LGALS3	LOC10013085	MGP	FAM19A4	HIATL2	CXorf45	C5orf37
LHB	LOC10013085	MIB2	FAM21C	HIF1AN	CYB5A	C5orf4
LHFP	LOC10013116	MICALL1	FAM40B	HIGD1A	CYBA	C5orf41
LIG4	LOC10013124	MIR155HG	FAM43A	HIP1R	CYBASC3	C5orf42
LIMA1	LOC10013133	MIR9-1	FAM45B	HIST1H1D	CYLN2	C5orf44
LIMCH1	LOC10013139	MLF2	FAM58A	HLA-A	CYP21A2	C5orf62
LMAN2L	LOC10013171	MLL5	FAM59A	HLA-H	CYP26B1	C6orf117
LMO1	LOC10013172	MMP11	FAM59B	HLTF	CYP2D6	C6orf125
LMOD1	LOC10013173	MPDZ	FAM69B	HM13	CYP2E1	C6orf126
LNX1	LOC10013190	MPHOSPH6	FAM76B	HMGB1L1	CYP2R1	C6orf129
LOC10012805	LOC10013214	MRPL21	FAM91A1	HMGB2	CYP39A1	C6orf148
LOC10012823	LOC10013224	MRPL44	FAM96B	HMMR	CYP3A43	C6orf160
LOC10012847	LOC10013245	MRPL55	FAM98A	HNRNPD	CYP3A5	C6orf168
LOC10012856	LOC10013249	MRPS15	FANCA	HNRNPH1	CYP4A11	C6orf170
LOC10012859	LOC10013250	MRPS30	FANCG	HNRNPH2	CYP4A22	C6orf204
LOC10012864	LOC10013270	MRPS34	FANCI	HNRNPK	CYP4F12	C6orf211
LOC10012899	LOC10013274	MSTO1	FASTKD5	HNRNPUL1	CYP4F2	C6orf48
LOC10012919	LOC10013276	MSX1	FBLN2	HNRPH1	CYP4F3	C6orf54
LOC10012954	LOC10013281	MTF1	FBXL15	HNRPUL1	CYP4V2	C6orf57
LOC10012955	LOC10013296	MTFMT	FBXL19	HOXB2	CYP8B1	C6orf59
LOC10012956	LOC10013297	MTMR12	FBXL5	HOXC13	D4S234E	C6orf66
LOC10012962	LOC10013303	MUSTN1	FBXL7	HOXC6	DACT1	C6orf89
LOC10012967	LOC10013307	MYADM	FBXO10	HS1BP3	DAD1L	C7orf10
LOC10012984	LOC10013327	MYCL1	FBXO15	HSD17B6	DAPK3	C7orf20
LOC10012995	LOC10013343	MYCT1	FBXO25	HSD17B7	DAZL	C7orf28A

LOC10012997	LOC10013343	MYL1	FBXO42	HSDL2	DBH	C7orf28B
LOC10012998	LOC10013345	MYLK4	FBXO46	HSN2	DBN1	C7orf40
LOC10013030	LOC10013347	MYO9B	FCAMR	HSPA12A	DCAF16	C7orf41
LOC10013045	LOC10013350	N4BP2L2	FCF1	HSPC171	DCBLD2	C7orf44
LOC10013079	LOC10013354	NAB1	FER1L5	HSPCAL3	DCP1B	C7orf46
LOC10013083	LOC10013356	NARF	FGF6	HSPD1	DCP2	C7orf50
LOC10013090	LOC10013367	NARG1	FGF9	HSZFP36	DCTN2	C7orf54
LOC10013104	LOC10013377	NAT13	FHOD1	HUWE1	DCUN1D4	C7orf57
LOC10013116	LOC10013388	NCK1	FIGN	HVCN1	DCXR	C7orf70
LOC10013119	LOC10013400	NCOA1	FIS	HYDIN	DDO	C8orf13
LOC10013125	LOC10013410	NCOA6IP	FIZ1	ICMT	DDRGK1	C8orf4
LOC10013128	LOC10013427	NCRNA00152	FKTN	ID3	DDT	C8orf55
LOC10013129	LOC10013429	NECAB2	FLJ11292	IDH1	DDX26B	C8orf58
LOC10013133	LOC10013436	NECAP2	FLJ12949	IER5L	DDX50	C8orf59
LOC10013147	LOC10019237	NGDN	FLJ16793	IFFO1	DDX51	C8ORFK29
LOC10013167	LOC10019237	NICN1	FLJ20397	IFIT3	DDX54	C9orf103
LOC10013173	LOC113386	NIN	FLJ20581	IFRD2	DDX56	C9orf125
LOC10013181	LOC124216	NMB	FLJ21687	IFT140	DDX59	C9orf129
LOC10013219	LOC133491	NMD3	FLJ25363	IFT52	DDX6	C9orf135
LOC10013221	LOC138652	NMNAT3	FLJ32011	IGF1	DECR2	C9orf140
LOC10013229	LOC142937	NOB1	FLJ32810	IGFBP5	DEDD2	C9orf3
LOC10013267	LOC151457	NOSTRIN	FLJ34047	IGSF21	DENND2D	C9orf40
LOC10013300	LOC153684	NPC2	FLJ35390	IL17D	DENND4A	C9orf46
LOC10013301	LOC200493	NPEPL1	FLJ35785	IL17RC	DGAT2	C9orf58
LOC10013311	LOC201229	NQO2	FLJ38379	IL28RA	DGKA	C9orf61
LOC10013317	LOC201651	NSUN4	FLJ40330	IL4R	DGKQ	C9orf72
LOC10013319	LOC282997	NT5C3	FLJ40722	IL8	DHRS1	C9orf80
LOC10013339	LOC285033	NUDCD3	FLJ42289	IMMP2L	DHRS12	C9orf85
LOC10013355	LOC285741	NUDT5	FLJ42709	IMP4	DHRS4L1	C9orf95
LOC10013369	LOC345630	NUMA1	FLJ44054	INO80C	DHRS7	CA10
LOC10013371	LOC347364	OAZ2	FLJ45513	INPP5D	DHRS9	CA11
LOC10013391	LOC347376	OGFOD1	FLJ45966	INTS4	DHX33	CA12
LOC10013425	LOC389102	OLFML1	FN3KRP	INTS5	DHX35	CA14
LOC10013454	LOC390712	OLIG2	FNDC4	INTS6	DHX36	CA2
LOC10013458	LOC390876	ONECUT1	FNIP2	IPP	DIP2A	CA4
LOC10014460	LOC391692	OR4K13	FNTB	IRAK3	DKFZp667M2	CABLES2
LOC10019093	LOC400236	OSBPL9	FOSL2	IRF2BP1	DKKL1	CABYR
LOC128192	LOC400879	OXSR1	FOXD2	IRS1	DLAT	CACHD1
LOC132241	LOC401002	P704P	FOXH1	IRX6	DLG4	CACNA1H
LOC137107	LOC401010	PAFAH1B2	FOXJ1	ISCA1	DLL1	CACNA2D2
LOC146517	LOC401076	PALLD	FPGS	ISCA2	DMGDH	CACNB2
LOC154761	LOC401237	PAQR7	FRAG1	ISM1	DMRT2	CACNB3
LOC162073	LOC440509	PARD6A	FRRS1	ISY1	DMTF1	CAD
LOC202051	LOC440570	PCBP4	FRY	ITGAE	DNA2	CADM1
LOC221442	LOC440900	PCDH17	FTH1	ITGB4BP	DNAJB1	CALB2
LOC257396	LOC441007	PCDHGB6	FTHL3	ITPKC	DNAJB5	CALCB
LOC25845	LOC441018	PCK2	FTHL8	ITPRIPL2	DNAJC10	CALCOCO1
LOC283874	LOC441081	PCMTD1	FTSJ2	IVD	DNAJC8	CALCOCO2
LOC283953	LOC441191	PCNT	FUNDC1	JARID1D	DNAJC9	CALM1
LOC284371	LOC441212	PEG3	FUT5	JMJD1A	DNAL1	CALML4

LOC285047	LOC441253	PELI2	FXC1	JMJD8	DNAL4	CALR
LOC340529	LOC441455	PFKFB4	FZD5	JUND	DNASE2	CALU
LOC341457	LOC441528	PGAM1	GAGE5	KANK1	DNM1	CAMK1
LOC341784	LOC441714	PGGT1B	GAK	KBTBD11	DNM1L	CAMK2G
LOC347292	LOC441743	PGM5	GALNT13	KBTBD8	DNTTIP1	CAMK2N1
LOC387723	LOC441763	PHYHIPL	GALNT3	KCNE1L	DNTTIP2	CAMK2N2
LOC387856	LOC442229	PIK3R2	GARNL3	KCNJ2	DOCK11	CAMKV
LOC389000	LOC442366	PILRA	GBAP	KCNK6	DPYD	CAMSAP1L1
LOC389322	LOC541471	PLAC8	GBE1	KCNMB3	DPYSL2	CAND2
LOC389332	LOC606724	PLAGL1	GBP3	KCTD10	DPYSL3	CAPN12
LOC389342	LOC641705	PLCD3	GBP7	KCTD13	DR1	CAPN13
LOC389901	LOC641804	PLEKHB2	GCET2	KCTD2	DRD4	CAPN3
LOC390372	LOC641992	PLEKHG3	GCN1L1	KDELC2	DRG2	CAPRIN1
LOC391019	LOC642073	PLEKHH3	GDA	KDELR3	DSG2	CAPRIN2
LOC391811	LOC642580	PLSCR1	GDI2	KDM6B	DSN1	CAPZA2
LOC392382	LOC642621	PLXNA2	GDPD4	KDSR	DSTN	CARD10
LOC400455	LOC642732	PLXNB3	GGA3	KIAA0284	DSTYK	CARD11
LOC400831	LOC642933	PMS2L4	GHITM	KIAA0355	DTX1	CARD8
LOC400890	LOC642953	PMS2L5	GIYD2	KIAA0408	DTX3	CARS
LOC400968	LOC643008	PNO1	GJB3	KIAA0415	DUOX1	CART1
LOC401238	LOC643792	POLR2J	GJB7	KIAA0513	DUS4L	CASK
LOC401533	LOC643872	POSTN	GK5	KIAA0564	DUSP10	CASP3
LOC402644	LOC644399	PPA1	GLB1L	KIAA1128	DUSP16	CASP4
LOC440978	LOC644544	PPARGC1B	GLG1	KIAA1257	DUX4	CASP6
LOC441050	LOC644596	PPFIA1	GLOD4	KIAA1530	DYNC1I1	CASP7
LOC441073	LOC644624	PPIA	GLOD5	KIAA1600	DYNC2H1	CAST
LOC441155	LOC644632	PPME1	GLRB	KIAA1602	DYRK3	CAT
LOC441193	LOC644830	PPP1R12B	GLT1D1	KIAA1632	E2F2	CAV2
LOC441241	LOC644869	PPP1R13L	GLTSCR2	KIAA1731	EARS2	CBARA1
LOC442249	LOC644884	PPP1R2	GMCL1	KIAA1751	EBI2	CBL
LOC641727	LOC644937	PPP1R3E	GMEB1	KIAA2013	EBP	CBLC
LOC641746	LOC645038	PPP2R1B	GMEB2	KIF16B	ECHS1	CBR1
LOC641750	LOC645276	PPRC1	GNAL	KIF21A	ECM2	CBR3
LOC641844	LOC645284	PPT2	GNB5	KIF21B	ECT2	CBX2
LOC641922	LOC645314	PRAMEF1	GNG2	KIF23	EDA2R	CBX4
LOC642062	LOC645534	PRDX4	GNPNAT1	KIF27	EDARADD	CBX7
LOC642250	LOC645626	PRKD1	GOLGA9P	KIF2C	EDEM2	CCBE1
LOC642333	LOC645661	PRNP	GOLPH3L	KIF3B	EEF2	CCBL2
LOC642412	LOC645732	PRO0628	GOSR1	KLF11	EEPD1	CCBP2
LOC642443	LOC646278	PROK1	GPATCH3	KLF15	EFEMP2	CCDC102A
LOC642446	LOC646301	PRR13	GPATCH8	KLF6	EFHC1	CCDC109A
LOC642456	LOC646345	PRR7	GPD1	KLF9	EFNA4	CCDC109B
LOC642486	LOC646561	PRRG1	GPHN	KLHL28	EGF	CCDC112
LOC642639	LOC646562	PRSS35	GPLD1	KLHL3	EHBP1L1	CCDC120
LOC642989	LOC646609	PSG2	GPR1	KLHL9	EID2	CCDC126
LOC643007	LOC646734	PSG4	GPR137C	KRT18P42	EID2B	CCDC136
LOC643018	LOC647054	PSG6	GPR141	KRT8	EIF1	CCDC138
LOC643035	LOC647074	PSG7	GPR88	KRT80	EIF2A	CCDC152
LOC643109	LOC647195	PSMA1	GPS1	LACTB	EIF2B2	CCDC18
LOC643123	LOC647251	PSMA6	GPSM2	LACTB2	EIF3E	CCDC28B

LOC643224	LOC647506	PSMC1	GPX4	LAGE3	EIF3F	CCDC34
LOC643300	LOC647543	PSMD11	GPX7	LAMB1	EIF3H	CCDC4
LOC643310	LOC647579	PTGS1	GRAMD1C	LAMB2L	EIF3L	CCDC41
LOC643384	LOC647597	PTPLAD1	GRIPAP1	LAP3	EIF4A2	CCDC43
LOC643389	LOC647673	PTPN21	GRK4	LASS6	EIF4A3	CCDC5
LOC643401	LOC647742	PTTG1IP	GRK6	LEMD3	EIF4G3	CCDC50
LOC643550	LOC647747	PURA	GRN	LGALS1	ELK4	CCDC51
LOC643605	LOC647805	PUS3	GSDM1	LHFPL2	ELMO3	CCDC56
LOC643802	LOC647855	PYGB	GSTT2B	LHPP	ELOVL2	CCDC58
LOC643985	LOC648294	PYROXD1	GSTTP2	LINGO1	ELOVL4	CCDC59
LOC644019	LOC648470	RAB11FIP4	GTDC1	LMAN1	ELP2	CCDC74A
LOC644063	LOC648581	RABEPK	GTF2A1	LMNB1	EME1	CCDC77
LOC644092	LOC648705	RAD21	GTPBP10	LMOD3	EMID2	CCDC90B
LOC644101	LOC649396	RAE1	GTSE1	LNPEP	EMP3	CCDC91
LOC644113	LOC649978	RALB	GUCA2B	LOC10012789	EMX2OS	CCDC92
LOC644276	LOC650494	RAP1A	GUSBL1	LOC10012797	ENHO	CCDC99
LOC644284	LOC650509	RAP2C	GYLTL1B	LOC10012800	ENOSF1	CCHCR1
LOC644334	LOC650518	RAPGEF6	GZF1	LOC10012809	ENPP3	CCKBR
LOC644496	LOC650566	RAVER1	HACE1	LOC10012812	ENPP4	CCL26
LOC644563	LOC650717	RBBP5	HAS2AS	LOC10012828	ENPP5	CCL5
LOC644580	LOC650850	RBM23	HAUS5	LOC10012829	ENPP7	CCNA1
LOC644612	LOC651380	RBM33	HCCS	LOC10012854	EPB49	CCNA2
LOC644615	LOC651635	RBM4	HCG4	LOC10012858	EPCAM	CCNB1IP1
LOC644634	LOC651751	RBM45	HDAC1	LOC10012888	EPHA8	CCNB2
LOC644701	LOC651959	RBM7	HDAC3	LOC10012907	EPHX1	CCNC
LOC644988	LOC651979	RCHY1	HEATR5A	LOC10012908	EPHX2	CCND3
LOC645181	LOC651997	RELN	HECTD3	LOC10012910	EPRS	CCNE1
LOC645231	LOC652140	RET	HEPACAM	LOC10012912	EPS15	CCNJ
LOC645241	LOC652183	RFNG	HERC2	LOC10012921	ERAP2	CCPG1
LOC645332	LOC652346	RFX5	HERPUD2	LOC10012926	ERC2	CCS
LOC645378	LOC652437	RGL3	HGS	LOC10012926	ERCC2	CCT2
LOC645515	LOC652479	RHBDL1	HIBADH	LOC10012929	ERLIN1	CCT3
LOC645550	LOC652534	RHEB	HINFP	LOC10012936	ERMAP	CCT6A
LOC645600	LOC652630	RHOQ	HIST1H3A	LOC10012950	ESPN	CCT6P1
LOC645837	LOC652679	RNASE1	HIST1H3D	LOC10012953	ETF1	CCT7
LOC645963	LOC652839	RNASE6	HIST1H3E	LOC10012955	ETFDH	CD14
LOC646144	LOC653276	RNASEH1	HIST2H4A	LOC10012960	EVC2	CD151
LOC646567	LOC653468	RNASEH2C	HIT-40	LOC10012966	EVI2B	CD163
LOC646779	LOC653600	RNASEL	HIVEP2	LOC10012972	EVL	CD164
LOC646786	LOC653657	RNF130	HJURP	LOC10012975	EXD2	CD19
LOC647174	LOC653895	RNF138	HK1	LOC10013009	EXOC1	CD200
LOC647262	LOC654078	RNF167	HLA-C	LOC10013015	EXOC2	CD24
LOC647264	LOC654126	RNF4	HLA-G	LOC10013022	EXOC4	CD248
LOC647346	LOC654127	RNPEP	HLCS	LOC10013027	EXT2	CD2AP
LOC647474	LOC654201	RNU1G2	HMGB1	LOC10013051	EXTL2	CD34
LOC647949	LOC654253	RPF1	HMGB3L1	LOC10013055	F11	CD36
LOC648025	LOC727751	RPL23AP13	HMHA1	LOC10013056	F13B	CD47
LOC648293	LOC727877	RRAGB	HNF4A	LOC10013062	F2	CD68
LOC648342	LOC727913	RRN3	HNRNPA2B1	LOC10013070	F5	CD70
LOC648366	LOC727914	RRP12	HNRNPF	LOC10013080	F8	CD79B

LOC648377	LOC728190	RRS1	HNRNPR	LOC10013083	F8A1	CD8A
LOC648570	LOC728351	RSBN1	HNRPR	LOC10013088	F9	CD9
LOC648622	LOC728453	RTF1	HNRPUL2	LOC10013089	FA2H	CD99
LOC648814	LOC728556	RTN2	HOXA9	LOC10013109	FABP1	CDAN1
LOC648907	LOC728608	RUFY3	HPDL	LOC10013154	FADS1	CDC123
LOC649379	LOC728758	RXRB	HPS4	LOC10013178	FADS2	CDC16
LOC649946	LOC728772	S100Z	HRCT1	LOC10013185	FAHD1	CDC25C
LOC650116	LOC728888	SAC3D1	HSD11B1	LOC10013189	FALZ	CDC2L5
LOC650144	LOC728934	SALL1	HSD11B1L	LOC10013244	FAM104A	CDC42
LOC650251	LOC728942	SCMH1	HSD17B12	LOC10013249	FAM105B	CDC42BPA
LOC650263	LOC728944	SCYL1BP1	HSD17B13	LOC10013250	FAM108A3	CDC42EP4
LOC650562	LOC728946	SDCCAG3	HSD17B7P2	LOC10013265	FAM108C1	CDC42SE1
LOC650580	LOC728953	SEC16A	HSF2BP	LOC10013271	FAM114A2	CDC7
LOC650706	LOC728965	SEC24C	HSF4	LOC10013271	FAM116B	CDCA4
LOC650867	LOC729123	SEH1L	HSPA1L	LOC10013274	FAM120A	CDCA5
LOC650885	LOC729148	SEMA4F	HSPA7	LOC10013311	FAM125A	CDCA7
LOC651102	LOC729200	SEMA5A	HSPE1	LOC10013348	FAM134C	CDCA7L
LOC651450	LOC729234	SENP2	HTR2B	LOC10013351	FAM135A	CDCP1
LOC652045	LOC729486	SERPINB9	HYI	LOC10013358	FAM14B	CDH23
LOC652481	LOC729519	SESN2	IARS2	LOC10013366	FAM162A	CDH26
LOC652607	LOC729558	SESN3	IBTK	LOC10013381	FAM173A	CDH3
LOC652634	LOC729768	SETD4	ICAM1	LOC10013386	FAM184A	CDH4
LOC652674	LOC729792	SETMAR	ICOSLG	LOC10013399	FAM195B	CDK2AP1
LOC652685	LOC729799	SEZ6	IDO2	LOC10013424	FAM30A	CDK2AP2
LOC652688	LOC729870	SF3B1	IDUA	LOC10013442	FAM36A	CDK5RAP1
LOC652713	LOC730378	SFRS12IP1	IFFO2	LOC10013450	FAM3B	CDK5RAP2
LOC652798	LOC730382	SFRS13A	IFIT5	LOC10013479	FAM45A	CDK5RAP3
LOC652846	LOC730415	SFRS2IP	IFNGR1	LOC10013481	FAM48A	CDK6
LOC652864	LOC730427	SFRS3	IFNGR2	LOC120376	FAM50A	CDKAL1
LOC653079	LOC730525	SFTPD	IFP38	LOC147727	FAM50B	CDKL3
LOC653188	LOC730995	SGCA	IFT172	LOC148430	FAM53C	CDO1
LOC653197	LOC731444	SGPL1	IGF2BP1	LOC148915	FAM63B	CDR2L
LOC653242	LOC731486	SH3BGRL2	IGHMBP2	LOC149501	FAM65A	CDS1
LOC653269	LOC731999	SH3BP5L	IGSF8	LOC151162	FAM65C	CDX2
LOC653316	LOC732316	SHC3	IKZF2	LOC200030	FAM78A	CDX4
LOC653423	LOC791120	SHE	IKZF5	LOC253039	FAM80B	CEACAM1
LOC653471	LOC92017	SHRM	IL10	LOC255275	FAM83D	CEBPA
LOC653596	LONP1	SIAH2	IL15RA	LOC283267	FAM90A3	CEBPB
LOC653857	LPCAT1	SIX2	IL17RA	LOC283755	FANCB	CEBPD
LOC654069	LPXN	SKIV2L	IL18	LOC284023	FANCE	CEBPZ
LOC654085	LRMP	SLA	IL1RAPL1	LOC284293	FAR1	CECR2
LOC654128	LRP11	SLAIN1	IL1RL1	LOC284821	FASTKD1	CEL
LOC654189	LRRC19	SLBP	IL1RL2	LOC285550	FAT1	CELP
LOC727762	LRRC28	SLC16A3	IL23A	LOC339970	FBLIM1	CENPB
LOC727882	LRRC29	SLC22A23	IL6ST	LOC341965	FBXL14	CENPH
LOC727901	LRRC42	SLC25A17	IMMT	LOC344328	FBXL20	CENPK
LOC728060	LRRN3	SLC25A4	IMPACT	LOC344405	FBXO3	CENPQ
LOC728178	LST1	SLC2A12	IMPDH1	LOC347544	FBXO36	CENPT
LOC728263	LY6G5C	SLC36A1	INCENP	LOC388796	FBXO4	CENTA1
LOC728320	LY6G6D	SLC36A4	INHBB	LOC388814	FBXO45	CEP68

LOC728408	LY86	SLC43A1	INPP4A	LOC388969	FBXO8	CEP78
LOC728493	LY9	SLC9A1	INPP5F	LOC389386	FBXW2	CER1
LOC728518	LYL1	SLC9A4	INTS1	LOC390183	FBXW5	CETN3
LOC728653	LYVE1	SLIT3	INTS12	LOC390354	FCER1G	CFB
LOC728924	M160	SLN	INTS3	LOC390940	FCGR2A	CFD
LOC729051	MAGEA12	SMARCC2	INTS9	LOC391132	FCGR3B	CFLAR
LOC729222	MAGI1	SMARCD2	IPO11	LOC391764	FCHO1	CGRRF1
LOC729279	MAP2K1	SNAP25	IPO9	LOC399815	FCN3	CHAC1
LOC729374	MAP3K5	SNAPC2	IPPK	LOC400750	FCRLB	CHAF1A
LOC729375	MAP7	SNAPC5	IQCC	LOC401098	FER	CHCHD2
LOC729408	MAP7D1	SNORA64	IRAK4	LOC401720	FERMT3	CHCHD3
LOC729466	MARCH7	SNORA70B	IRS2	LOC439949	FES	CHCHD4
LOC729495	MARCO	SNORD10	IRX5	LOC440927	FETUB	CHCHD6
LOC729513	MARVELD1	SNORD100	ITGB3BP	LOC441046	FEZ2	CHCHD9
LOC729642	MARVELD2	SNORD12	ITGB7	LOC441066	FGD1	CHD4
LOC729645	MCCD1	SNORD12C	ITPKB	LOC441087	FGD6	CHD5
LOC729647	MCF2L	SNORD14B	ITPR1	LOC441481	FGF11	CHD9
LOC729659	MED24	SNORD58B	ITPRIP	LOC441896	FGFR1	CHDH
LOC729764	MEF2C	SNORD68	JAGN1	LOC442075	FGL1	CHEK1
LOC729789	MEG8	SNORD83B	JAK1	LOC442727	FGL2	CHEK2
LOC729798	MEMO1	SNRNP70	JHDM1D	LOC642197	FGR	CHGA
LOC729843	METTL2A	SNX14	JMJD2C	LOC642502	FH	CHGB
LOC730004	MEX3C	SNX29	JMJD6	LOC643033	FHL3	CHMP2A
LOC730036	MFSD7	SNX3	JTB	LOC643256	FIG4	CHMP4A
LOC730074	MGC12760	SNX7	KBTBD6	LOC643308	FIS1	CHMP5
LOC730107	MGC29506	SPAG9	KCND3	LOC643779	FIT1	CHMP6
LOC730130	MGC3196	SPG21	KCNIP4	LOC643863	FKBP10	CHN1
LOC730134	MIA2	SPG7	KCTD5	LOC643911	FKBP1A	CHN2
LOC730288	MICAL2	SPOP	KCTD7	LOC643997	FLCN	CHODL
LOC730316	MIDN	SPOPL	KGFLP1	LOC644315	FLJ10324	CHP2
LOC730323	MIMT1	SPSB4	KIAA0196	LOC644380	FLJ10357	CHPF2
LOC730324	MINPP1	SPTAN1	KIAA0406	LOC644412	FLJ10661	CHPT1
LOC730396	MIR144	SRFBP1	KIAA0492	LOC644422	FLJ10781	CHRD
LOC730952	MIR182	SRGAP3	KIAA0495	LOC644517	FLJ12355	CHRNA3
LOC731007	MIR1976	SSH1	KIAA0738	LOC644739	FLJ12684	CHRNA5
LOC731985	MIR300	ST14	KIAA1147	LOC644877	FLJ14166	CHRNA9
LOC732160	MIR886	ST6GALNAC4	KIAA1160	LOC644936	FLJ22531	CHST12
LOC732432	MMRN1	ST8SIA4	KIAA1211	LOC645100	FLJ25006	CHST2
LOC732450	MNDA	STAG1	KIAA1285	LOC645296	FLJ35934	CHST3
LOC91661	MOBKL1A	STAT5A	KIAA1324	LOC645387	FLJ36131	CHST4
LOXL1	MPO	STK38	KIAA1333	LOC645508	FLJ37786	CHST6
LPCAT2	MPP3	STMN2	KIAA1524	LOC645659	FLJ39660	CHST7
LRP1B	MPP5	STOX1	KIAA1618	LOC645737	FLJ40113	CHST8
LRP2	MPV17L2	STRC	KIAA1671	LOC645822	FLJ44342	CIB1
LRRC67	MRPL42P5	STYX	KIAA1958	LOC646038	FLJ46906	CIB2
LRRFIP1	MRPL46	SUMO1	KIAA2026	LOC646123	FLJ90036	CIC
LRRN4	MRPS24	SUSD1	KIF11	LOC646531	FLJ90086	CIDEB
LSM10	MS4A3	SYT14	KIF14	LOC646785	FLNA	CIP29
LSM11	MS4A4A	SYT9	KIF18A	LOC646909	FLVCR2	CIR1
LSM6	MS4A7	TACC2	KIF1C	LOC647030	FLYWCH2	CIRH1A

LY6G6C	MSLN	TACO1	KIF20B	LOC647037	FMNL2	CITED2
LYG1	MSN	TACSTD2	KIF22	LOC647691	FMO3	CITED4
LYN	MSR1	TAF15	KIF24	LOC647886	FMO5	CKAP2L
LYPD3	MT1B	TAF9	KIF4A	LOC647954	FNDC1	CKAP5
LYPD5	MTHFD2L	TAP1	KILLIN	LOC648000	FOLH1B	CKB
LYPLA2P1	MUC12	TAS2R10	KLHDC3	LOC648638	FOXD4L1	CKMT1A
LZTR1	MX2	TBC1D1	KLHDC8A	LOC648822	FOXM1	CKMT1B
MAB21L2	MXI1	TBC1D10B	KLHL15	LOC648931	FOXN2	CKS1B
MAEA	MYCBPAP	TBC1D24	KLHL2	LOC649169	FRAP1	CLASP1
MAGT1	MYH3	TBL1X	KLHL35	LOC649209	FRAT1	CLASP2
MAML1	MYO16	TBRG4	KPNA7	LOC649270	FRG1	CLCNKA
MAML3	MYO1F	TBX2	KRT6B	LOC649330	FRK	CLCNKB
MAN1B1	MYO1G	TCEB1	KTI12	LOC649604	FRMD4A	CLDN1
MAP1A	NACAP1	TCEB3CL	L3MBTL3	LOC649639	FRMD5	CLDN10
MAP2	NAPSA	TCF21	LAD1	LOC649917	FRS3	CLDN11
MAP2K1IP1	NAPSB	TCTEX1D1	LAIR1	LOC650826	FTHL11	CLDN14
MAPK4	NCF1C	TEAD3	LAMP1	LOC651202	FTHL16	CLDN15
MAPKBP1	NCF2	TERF2	LANCL2	LOC651436	FTHL2	CLDN18
MARK2	NCF4	TES	LASS5	LOC652094	FTL	CLDN6
MATN1	NCKAP1L	TEX14	LAT1-3TM	LOC652624	FUBP1	CLEC16A
MATN2	NCRNA00081	TFRC	LCLAT1	LOC652741	FUCA1	CLEC2D
MBD3L2	NDUFA8	TGFA	LDHA	LOC652773	FUT10	CLGN
MBTPS1	NFKBIL1	THAP8	LDLRAD3	LOC652837	FUT9	CLIC1
MCHR2	NHLRC4	THUMPD1	LDOC1L	LOC653066	FVT1	CLIC4
MDH2	NID1	TIE1	LEP	LOC653080	FXYD2	CLIC6
MED12L	NKAP	TIMM23B	LETM1	LOC653110	G6PC	CLIP3
MED15	NKD2	TIPRL	LFNG	LOC653210	G6PC3	CLK1
MED16	NKG7	TJP2	LGALS4	LOC653333	GABARAP	CLK2
MED18	NLRP3	TMEM14B	LGALS9B	LOC653383	GABBR1	CLK3
MED7	NLRP4	TMEM170A	LGI1	LOC653505	GABBR2	CLN3
MEI1	NME4	TMEM173	LGTN	LOC653566	GABRG2	CLNS1A
MFN2	NOL10	TMEM194B	LILRB1	LOC653583	GADD45B	CLPTM1
MFSD3	NOL12	TMEM47	LIN7A	LOC653604	GAL3ST1	CLSTN1
MFSD8	NOTCH4	TMEM54	LIN9	LOC653631	GALE	CLTA
MGC21881	NPAS3	TMEM63A	LINS1	LOC653702	GALK1	CLYBL
MGC23270	NPC1	TMEM80	LMBR1	LOC654002	GALNT7	CMPK1
MGC23284	NPHP1	TMEM97	LMNB2	LOC654121	GAPDH	CMTM3
MGC26718	NPTN	TMLHE	LMO7	LOC654191	GAS2	CMTM4
MGC26733	NR2F1	TMTC2	LMTK2	LOC723972	GAS7	CNBP
MGC45800	NRF1	TNFAIP6	LOC10000967	LOC727761	GAS8	CNDP2
MICA	NRG3	TNFRSF19	LOC10012791	LOC727826	GATAD1	CNFN
MID1IP1	NSMCE2	TNFRSF1A	LOC10012797	LOC727950	GATAD2A	CNGA1
MIER1	NSUN2	TNFSF4	LOC10012798	LOC727970	GATM	CNIH4
MINK1	NSUN3	TNNT2	LOC10012799	LOC728411	GBA3	CNKSR3
MIP	NTSR1	TNRC15	LOC10012800	LOC728452	GBAS	CNN1
MIR1228	NXF3	TNS3	LOC10012806	LOC728499	GBP1	CNN2
MIR1247	OBFC2B	TOM1L1	LOC10012808	LOC728532	GCA	CNNM3
MIR128-2	OGN	TOX3	LOC10012819	LOC728734	GCAT	CNOT4
MIR30C2	OIT3	TP53	LOC10012822	LOC728843	GCK	CNOT6L
MIR507	OLFM2	TP53I3	LOC10012826	LOC728877	GCKR	CNOT7

MIR586	OLFM4	TPM3	LOC10012829	LOC729217	GCLC	CNRIP1
MIR720	OPRK1	TPP2	LOC10012834	LOC729259	GDF11	CNTN1
MKKS	OR1J1	TPST2	LOC10012839	LOC729505	GEN1	CNTNAP1
MKL1	OR5K4	TREML2	LOC10012839	LOC729535	GFOD1	CNTNAP2
MKRN2	ORAOV1	TRIM22	LOC10012846	LOC729687	GFOD2	CNTNAP3
MLEC	OTUD5	TRIM36	LOC10012850	LOC729708	GGCX	CNTNAP3B
MLL	P2RX1	TRIM44	LOC10012851	LOC729732	GGH	COASY
MLLT1	P2RY13	TRIM52	LOC10012852	LOC729774	GGT1	COBLL1
MLLT6	P2RY8	TRIM61	LOC10012867	LOC729852	GGT2	COCH
MMP1	P4HTM	TRIM68	LOC10012873	LOC729905	GGT3P	COG5
MMP15	PACAP	TRIP6	LOC10012877	LOC729926	GGTLC2	COL12A1
MMP28	PACSIN3	TRPV1	LOC10012893	LOC730041	GHRLOS	COL1A1
MOBKL2A	PADI4	TRPV2	LOC10012902	LOC730060	GIMAP2	COL1A2
MON2	PAFAH1B1	TSPAN12	LOC10012902	LOC730167	GLIPR1	COL22A1
MORG1	PARVB	TSPAN14	LOC10012905	LOC730202	GLMN	COL23A1
MORN3	PARVG	TSPAN17	LOC10012914	LOC730313	GLRX2	COL3A1
MPHOSPH8	PCDH12	TSR1	LOC10012930	LOC730993	GLTP	COL4A2
MRFAP1L1	PCDH9	TTC18	LOC10012934	LOC730996	GLTPD2	COL4A3BP
MRGPRX1	PCSK4	TTC3	LOC10012936	LOC731049	GLUD1	COL4A5
MRGPRX3	PCYT1B	TTL	LOC10012938	LOC732075	GLYAT	COL4A6
MRGPRX4	PDE1A	TTLL12	LOC10012942	LOC90586	GMFB	COL5A1
MRPL13	PDE2A	TTTY14	LOC10012942	LOC92755	GMFG	COL5A2
MRPL16	PECAM1	TUFT1	LOC10012944	LPAR1	GMPPA	COL6A3
MRPL20	PET112L	TWIST2	LOC10012944	LPIN1	GMPPB	COL8A1
MRPL30	PEX19	TXNDC5	LOC10012952	LPL	GMPR2	COMMD10
MRPL32	PEX5L	TXNDC9	LOC10012954	LPP	GNA13	COMMD3
MRPL4	PF4V1	UBE1DC1	LOC10012960	LRBA	GNAT1	COMMD7
MRPL48	PGA3	UBE2D2	LOC10012963	LRP1	GNB3	COMMD9
MRPS10	PGA5	UBE2D3	LOC10012970	LRRC1	GNGT1	COPE
MRPS11	PGLYRP1	UBE2E1	LOC10012974	LRRC3	GNL2	COPS7A
MRPS17	PHC3	UBE3B	LOC10012978	LRRC45	GNMT	COQ10A
MRPS18B	PHF12	UBTD1	LOC10012993	LRRC61	GNPDA1	COQ3
MRPS2	PHOSPHO1	UEVLD	LOC10012995	LSR	GOLGA4	COQ5
MSL1	PI4K2A	UHMK1	LOC10012996	LTBP4	GOLGA6B	COTL1
MTP18	PI4KA	ULK4	LOC10012997	LUM	GOLM1	COX11
MTRR	PIN4	UMPS	LOC10013005	LYSMD1	GOLT1B	COX6B1
MTUS2	PIP4K2C	UNC45A	LOC10013007	LZTS2	GPAA1	CPEB1
MTX1	PIP5K1B	UPF1	LOC10013016	MAD2L1BP	GPATCH2	CPM
MUC15	PIP5KL1	URB2	LOC10013017	MAGED2	GPC6	CPN1
MUM1L1	PKD2L1	USMG5	LOC10013035	MAGOH	GPIHBP1	CPN2
MUS81	PLEKHA3	UTP11L	LOC10013038	MALL	GPKOW	CPNE4
MUTED	PLGLA	VCAM1	LOC10013038	MAN1C1	GPN1	CPO
MXRA8	PLIN	VEZT	LOC10013042	MAN2B1	GPR116	CPS1
MYBBP1A	PLVAP	VISA	LOC10013042	MANEAL	GPR128	CPSF2
MYBPHL	PMF1	VKORC1L1	LOC10013044	MAP3K1	GPR146	CPSF6
MYCBP2	PMS2L3	VSIG2	LOC10013046	MAP3K2	GPR175	CPT1A
MYL3	PNMT	VSTM2B	LOC10013051	MAP3K6	GPR27	CPT1B
MYNN	PODN	VWF	LOC10013052	MAP3K7IP1	GPS2	CPT1C
MYO3A	POGK	WDR20	LOC10013054	MAP3K8	GPSM1	CPXM1
NACAD	POLR2C	WDR21A	LOC10013055	MAP4K4	GPT	CPXM2

NANS	POLRMT	WDR26	LOC10013055	MAP7D2	GPX3	CPZ
NARFL	POP7	WDR40A	LOC10013057	MAPK10	GRAP	CR2
NAT1	POU2AF1	WDR59	LOC10013059	MAPK3	GRB7	CRABP1
NAT14	PPAPDC1B	WDR60	LOC10013063	MAPK8IP3	GRHL3	CRABP2
NCOA3	PPDPF	WDR82	LOC10013080	MAPKAPK3	GRHPR	CRADD
NCOR1	PPFIA3	WDR91	LOC10013093	MAT2A	GRIN3B	CRAT
NCRNA00095	PPIL2	WFIKKN1	LOC10013093	MATK	GRIP2	CREB3L3
NDUFA10	PPM1F	WHSC1	LOC10013107	MBD4	GSS	CREB3L4
NDUFA13	PPM1J	WNT5A	LOC10013109	MBTD1	GSTM1	CREB5
NDUFA6	PPP1R14D	WRNIP1	LOC10013109	MCFD2	GSTM4	CREG1
NDUFAB1	PPTC7	WWP2	LOC10013122	MCM10	GSTO1	CRIP1
NDUFAF2	PQLC3	YPEL3	LOC10013126	MCM3AP	GSTP1	CRISPLD2
NDUFB4	PRAM1	YTHDF2	LOC10013127	MCM4	GTF2E1	CRK
NDUFB9	PRB3	YWHAE	LOC10013134	MCM5	GTF2H4	CRLF1
NEDD4L	PRKAA2	YWHAG	LOC10013136	MCM6	GTF2IRD1	CRMP1
NEU1	PRKG2	ZBTB40	LOC10013145	MCM7	GTF3C2	CROP
NF1	PRMT8	ZBTB8OS	LOC10013146	MCMDC1	GTF3C3	CROT
NFAT5	PRND	ZC3H15	LOC10013151	MDP1	GTPBP2	CRSP2
NGLY1	PRO0132	ZC3H4	LOC10013169	MED28	GUCA1A	CRTAP
NHLRC3	PRPF3	ZCCHC14	LOC10013170	METTL13	GUCA2A	CRTC2
NIPAL4	PRR15L	ZDHHC4	LOC10013177	MEX3D	GUCY2C	CRTC3
NIPSNAP1	PRSS21	ZER1	LOC10013178	MFSD11	GUK1	CRY1
NKX2-3	PRSS8	ZFAND6	LOC10013189	MGA	GUSB	CRY2
NOG	PRSSL1	ZFP30	LOC10013197	MGAT4A	GYS2	CRYGD
NOL4	PRTN3	ZMYND19	LOC10013198	MGAT4B	H2AFJ	CRYL1
NOLA1	PSCD4	ZNF137	LOC10013201	MGC10997	H2AFX	CRYZ
NOLC1	PSCDBP	ZNF148	LOC10013202	MGC27345	H2AFY2	CRYZL1
NPHP3	PSD	ZNF16	LOC10013223	MGC3020	HAAO	CSAD
NPNT	PSMA3	ZNF259	LOC10013228	MGC5139	HADH2	CSDA
NRXN3	PSMB2	ZNF264	LOC10013229	MGC72080	HADHA	CSDE1
NSMCE1	PSMB7	ZNF3	LOC10013234	MGST1	HADHB	CSE1L
NTF4	PSMD1	ZNF318	LOC10013243	MINA	HAGH	CSF1R
NTF5	PTCRA	ZNF346	LOC10013252	MIR1974	HAL	CSF2RA
NTRK2	PTPN1	ZNF407	LOC10013255	MIR503	HAMP	CSK
NUMB	PTPN11	ZNF436	LOC10013255	MKL2	HAO1	CSNK1A1
NUP214	PTPRB	ZNF470	LOC10013258	MLF1IP	HAO2	CSNK1D
NXPH2	PTPRC	ZNF488	LOC10013265	MLH1	HAS1	CSNK1E
OBSCN	PTPRCAP	ZNF491	LOC10013272	MLST8	HAUS4	CSNK2A1
OCLN	PTPRN2	ZNF500	LOC10013277	MNT	HAUS8	CSNK2A1P
ODAM	PTPRO	ZNF526	LOC10013286	MOCS1	HBG2	CSRP2
OFD1	PTPRU	ZNF670	LOC10013286	MOCS2	HCCA2	CST1
OLR1	PUSL1	ZNF791	LOC10013296	MOGS	HCFC1R1	CST3
OPHN1	PVALB	ZNF92	LOC10013304	MPHOSPH9	HCG2P7	CST6
OR10AG1	R3HDM2	ZYG11B	LOC10013305	MRPL1	HCN4	CSTF1
OR7E91P	RAB33B	ZYX	LOC10013305	MRPL36	HCST	CSTF3
OSAP	RAB6A		LOC10013307	MRPL37	HDDC2	CTAGE6
OSBPL11	RAB6B		LOC10013312	MRPL43	HDGF2	CTBP2
OSGEP	RAD23A		LOC10013314	MRPL50	HDHD1A	CTCFL
OSTF1	RAD23B		LOC10013317	MRPS12	HERC4	CTDSP1
OTUD4	RAG1AP1		LOC10013318	MRPS25	HES1	CTGF

OVCA2	RAMP3	LOC10013323	MRPS6	HEXDC	CTGLF7
OVOL1	RAP2A	LOC10013326	MRPS9	HFM1	CTH
OXGR1	RAPGEF2	LOC10013331	MSRB3	HGD	CTHRC1
OXTR	RARS	LOC10013346	MTERFD1	HGFAC	CTNNA1
P2RX6	RASSF10	LOC10013346	MTHFSD	HHEX	CTPS2
P2RY6	RB1CC1	LOC10013347	MXRA5	HINT1	CTSA
PABPC4	RBM16	LOC10013348	MXRA7	HINT2	CTSB
PACS1	RBM28	LOC10013356	MYH10	HIPK2	CTSC
PAK4	RBM38	LOC10013359	MYLIP	HIST1H2AC	CTSH
PAQR6	RBM43	LOC10013360	MYO5B	HIST1H2AE	CTSL1
PCCB	RBP2	LOC10013372	MYO9A	HIST1H4C	CTSL2
PCDHB10	RCE1	LOC10013374	MYOM2	HIST2H2AB	CTSO
PCDHB16	RDH14	LOC10013377	MYPOP	HIVEP1	CTSZ
PCDHB4	REEP5	LOC10013377	N4BP2	HLA-DPA1	CTXN1
PCDHGA12	REPS1	LOC10013393	NAALAD2	HLA-DRB4	CUL4B
PCIF1	REST	LOC10013405	NADSYN1	HLF	CUX2
PCP4	RETN	LOC10013408	NASP	HLX	CUZD1
PCSK7	RFX2	LOC10013408	NAT10	HMBOX1	CXCL1
PDCD6IP	RFXDC2	LOC10013409	NAT12	HMGA2	CXCL12
PDGFB	RGS18	LOC10013415	NAT5	HMGCL	CXCL5
PDRG1	RGS6	LOC10013418	NAV3	HMGCLL1	CXCL6
PDXDC2	RHAG	LOC10013421	NBEAL2	HMOX1	CXorf15
PDZD3	RHBDD3	LOC10013422	NBPF11	HMX2	CXorf38
PDZRN3	RHBG	LOC10013425	NBPF14	HNF1A	CXorf57
PELI3	RHCE	LOC10013426	NBPF3	HNF4G	CXorf64
PER3	RHOH	LOC10013436	NCAPD2	HNRNPA0	CXXC1
PES1	RHOJ	LOC10013458	NCAPG2	HNRNPH3	CXXC6
PFTK1	RHOT2	LOC10013466	NCL	HNRNPL	CYB561
PGBD5	RHPN1	LOC10013470	NCRNA00085	HNRNPUL2	CYB561D1
PGM1	RIOK2	LOC10013473	NCRNA00092	HNRPA1L-2	CYB5D2
PHCA	RIPK3	LOC10013486	NDUFS2	HNRPA1P4	CYB5R1
PHF10	RNASE2	LOC10019093	NEFH	HNRPC	CYB5R2
PHF20L1	RNASE3	LOC113230	NEIL3	HNRPH3	CYB5R3
PHF3	RNF14	LOC121792	NET1	HOMER1	CYBRD1
PHLPP1	RNF212	LOC123876	NEURL4	HOMER3	CYCS
PHPT1	RNU4ATAC	LOC130773	NFKBIA	HOXB1	CYCSL1
PIBF1	ROBO4	LOC131691	NGFR	HP	CYCSP52
PIGC	ROGDI	LOC136143	NIP7	HPCAL1	CYFIP1
PIGG	ROPN1B	LOC139735	NIPBL	HPD	CYFIP2
PIH1D1	RP9	LOC143941	NISCH	HPR	CYorf15A
PILRB	RPRC1	LOC145783	NKAIN1	HRC	CYorf15B
PIP5K1C	RPS29	LOC145837	NKTR	HRG	CYP1A1
PIPSL	RPS6KA1	LOC146053	NKX3-1	HSD3B7	CYP20A1
PIWIL4	RRP1	LOC149224	NLF2	HSDL1	CYP26A1
PKN2	RRP1B	LOC149351	NMRAL1	HSF1	CYP27A1
PLAA	RSAD2	LOC151579	NNMT	HSFX1	CYP27B1
PLAC2	RUNDC3A	LOC152217	NODAL	HSP90AB1	CYP2C8
PLCD1	S100A12	LOC152586	NOP16	HSPC268	CYP2F1
PLCXD3	S100A8	LOC197350	NOXA1	HULC	CYP2J2
PLD5	S100A9	LOC201175	NPEPPS	HYAL1	CYP2S1

PLEKHG4B	SAMD1	LOC203547	NPLOC4	IAH1	CYP3A7
PLEKHJ1	SAMD8	LOC220433	NPY	ICA1L	CYP51A1
PMFBP1	SAMD9L	LOC255167	NR1D2	ICAM2	CYTH1
PMS2	SAMSN1	LOC283481	NR2F6	ICAM3	CYTL1
PNRC2	SASH3	LOC283711	NRBP1	ID1	CYTSB
POLH	SATB2	LOC285095	NRBP2	ID4	D2HGDH
POLR2J2	SCARNA13	LOC285176	NRCAM	IFI27L1	DAB2
POLR2J3	SCFD2	LOC285908	NRD1	IFI44	DALRD3
POLR3E	SCGB3A1	LOC286002	NRIP1	IFITM4P	DAP
POM121C	SCN7A	LOC286367	NSBP1	IGBP1	DAZ4
POMT2	SCRIB	LOC286512	NSL1	IGF1R	DAZAP1
POPDC2	SCRN3	LOC338756	NT5DC2	IGF2BP2	DAZAP2
POTEE	SDPR	LOC338870	NTAN1	IGFALS	DBC1
PPAN-P2RY11	SEC24A	LOC339804	NTF3	IGFBP1	DBI
PPEF1	SEC24B	LOC339843	NTHL1	IHPK3	DBNDD1
PPIE	SELL	LOC342979	NTN4	IKBKAP	DBNDD2
PPIL6	SELP	LOC342994	NUDC	IKBKE	DBNL
PPP1R3D	SERAC1	LOC347487	NUDT18	IKBKG	DCAF6
PPP2R3A	SERPINA13	LOC388076	NUDT7	IL17RB	DCK
PPP3R1	SETD8	LOC388237	NUF2	IL18BP	DCLK1
PRAMEF13	SFI1	LOC388327	NUP62CL	IL1B	DCLRE1C
PRDM7	SFRS2B	LOC388339	NUP88	IL1RAP	DCN
PRDX3	SFRS8	LOC388503	NUPL2	IL1RN	DCTN3
PRINS	SGIP1	LOC388514	NVL	IL22RA1	DCUN1D5
PRKAR2A	SHROOM3	LOC388564	OGG1	IL6	DDB1
PRKCE	SIGLEC10	LOC388681	OGT	IL6R	DDB2
PRKRIP1	SIGLEC11	LOC388955	OLFML2A	ILDR1	DDC
PRPF18	SIGLEC14	LOC389053	OPLAH	ILKAP	DDIT3
PRPF40A	SIGLEC16	LOC389072	OPRL1	IMP3	DDIT4
PRPSAP1	SIGLEC7	LOC389156	ORC1L	IMPA2	DDIT4L
PRR15	SLC11A1	LOC389465	OSBP	IMPDH2	DDOST
PRRT1	SLC12A4	LOC389523	OSBPL5	INA	DDR1
PRSS22	SLC12A8	LOC389634	OSBPL7	INO80D	DDR2
PRTG	SLC13A3	LOC389791	OSCAR	INPP1	DDX10
PSKH1	SLC17A8	LOC390298	OSTC	INPP5B	DDX11
PSMA5	SLC1A4	LOC390671	OTUD1	INTS2	DDX19B
PSMB3	SLC22A16	LOC390956	P2RY2	INTS8	DDX21
PSMC4	SLC22A4	LOC391126	P2RY5	INTU	DDX25
PSMD3	SLC25A38	LOC391359	P4HB	IPO8	DDX28
PSMD8	SLC26A1	LOC391370	PAF1	IPW	DDX31
PSME3	SLC26A10	LOC391656	PAFAH2	IRAK2	DDX47
PSMG1	SLC26A3	LOC391817	PALM	IREB2	DEAF1
PSMG3	SLC30A2	LOC392145	PAM	IRF5	DECR1
PTAFR	SLC35E4	LOC392197	PAMR1	IRF7	DEFB130
PTOV1	SLC38A5	LOC399900	PANK2	IRX1	DEGS1
PUF60	SLC38A6	LOC400027	PAPOLA	ISCU	DEM1
PUM2	SLC4A1	LOC400174	PAPPA	ISG20	DENND1A
PURB	SLC4A3	LOC400446	PAQR8	ISG20L2	DENND2A
PVRL3	SLC5A11	LOC400578	PARP16	ISOC1	DENND4C
PVRL4	SLC9A7	LOC400657	PARP3	ITFG3	DENR

PYGO2	SLC9A9		LOC400721	PBX2	ITGA3	DEPDC6
QSOX2	SLCO5A1		LOC400759	PCBD2	ITGA5	DFFA
RAB11A	SMPD2		LOC401007	PCDH20	ITGAL	DFNB59
RAB13	SMPD4		LOC401218	PCDH7	ITGAM	DGAT1
RAB8B	SNAP47		LOC401398	PCDHB9	ITGB2	DGCR2
RABGAP1L	SNF8		LOC401588	PCTK3	ITGB5	DGCR5
RABL2B	SNHG3-RCC1		LOC401640	PCYOX1	ITIH2	DHCR24
RALGPS2	SNHG9		LOC401676	PDCD4	ITIH3	DHCR7
RANGRF	SNN		LOC401847	PDE4D	ITIH4	DHRS2
RAP1BL	SNORA13		LOC402342	PDE4DIP	ITSN1	DHRS3
RAPGEF1	SNORA70		LOC402571	PDE6G	IWS1	DHRS4
RAPGEFL1	SNORA78		LOC402677	PDE8A	IYD	DHRS4L2
RASA2	SNORD114-1		LOC407835	PDF	JAG1	DHX15
RBM15B	SNORD114-3		LOC440055	PDGFC	JAM3	DHX38
RBM42	SNORD15B		LOC440280	PDGFRB	JDP2	DHX9
RBM4B	SNORD1A		LOC440503	PDHB	JMJD4	DIABLO
RELT	SNORD33		LOC440551	PDLIM7	JMY	DIAPH1
REPIN1	SNORD35A		LOC440589	PDP2	JUNB	DIAPH2
RETNLB	SNORD42B		LOC440776	PDXDC1	KANK2	DIAPH3
RFP	SNORD4A		LOC440925	PEF1	KARS	DIDO1
RFPL3S	SNORD69		LOC440926	PEPD	KAT2A	DIMT1L
RFX6	SNRNP25		LOC440991	PEX11G	KAT5	DIO1
RG9MTD1	SNRPB2		LOC441114	PEX6	KCNA6	DIO2
RGAG1	SNRPG		LOC441150	PFAS	KCNF1	DKC1
RGAG4	SNX12		LOC441208	PGAM2	KCNH2	DKFZP564J102
RGL2	SNX21		LOC441282	PGBD1	KCNH6	DKFZp761P04[illegible]
RGP1	SNX31		LOC441461	PGBD3	KCNJ10	DKK3
RGS19	SOBP		LOC441488	PGM2	KCNMB2	DLG2
RHOBTB1	SOX6		LOC441511	PGM3	KCNS1	DLG3
RHOBTB2	SP2		LOC441550	PGP	KCTD11	DLG5
RIC8A	SPESP1		LOC441775	PHF1	KDM4D	DLGAP5
RICS	SPI1		LOC442421	PHF13	KEAP1	DLK1
RICTOR	SPIB		LOC442519	PHF19	KENAE	DLL3
RIN3	SPO11		LOC554223	PHIP	KHDC1	DLX3
RIOK1	SPTA1		LOC641710	PHKG2	KHDRBS1	DLX5
RLN1	SPTB		LOC641741	PHLDB3	KHDRBS2	DMC1
RMND5B	SPTBN4		LOC641772	PI4KAP2	KHK	DMKN
RN7SK	SRCRB4D		LOC641798	PIAS1	KIAA0101	DMRTA1
RNASET2	SRL		LOC641819	PIGA	KIAA0146	DMWD
RNF112	SSH3		LOC641848	PIGN	KIAA0174	DNAH1
RNF24	STAB1		LOC641941	PIGS	KIAA0363	DNAH14
RNF31	STAB2		LOC641972	PIGW	KIAA0427	DNAJA1
RNF43	STAG3		LOC641983	PIM3	KIAA0586	DNAJA4
RNMTL1	STAG3L2		LOC641989	PIN1	KIAA0907	DNAJB12
RNU1-5	STAG3L3		LOC642109	PIPOX	KIAA0947	DNAJB2
RNU1F1	STAT5B		LOC642373	PITPNM1	KIAA1012	DNAJB6
RNY3	STX8		LOC642441	PITRM1	KIAA1026	DNAJB9
RPA4	SUCNR1		LOC642458	PJCG6	KIAA1279	DNAJC12
RPL23	SULT1C2		LOC642628	PKD1	KIAA1688	DNAJC15
RPL26L1	SUMF2		LOC642656	PKMYT1	KIAA1797	DNAJC18

RPL29	SYNC1	LOC642749	PKNOX1	KIAA1826	DNAJC2
RPL31P10	TAL1	LOC642755	PLAG1	KIAA1881	DNAJC21
RPL35	TAX1BP3	LOC642769	PLCE1	KIAA1920	DNAJC22
RPL36	TBC1D10C	LOC642812	PLCG1	KIAA2010	DNAJC24
RPRD2	TBC1D19	LOC642828	PLD3	KIF12	DNAJC25
RPS15A	TBRG1	LOC642889	PLEC1	KIF20A	DNAJC30
RPS27	TBXAS1	LOC642947	PLEKHA4	KIF3C	DNALI1
RPS28	TEX13A	LOC642968	PLEKHA9	KIF7	DNHL1
RPS6KB1	TFDP1	LOC642981	PLEKHF2	KIFC2	DNM2
RPTOR	TFDP2	LOC643011	PLEKHM1	KISS1	DNMT3A
RPUSD2	TFE3	LOC643187	PLK1	KIT	DNMT3B
RQCD1	TFF3	LOC643373	PLK4	KLC1	DNTT
RRP15	THRA	LOC643382	PLLP	KLF4	DOCK1
RSPO1	THRAP5	LOC643446	PLOD1	KLHL36	DOCK2
RSPO2	TIMD4	LOC643509	PLS1	KLHL5	DOCK3
RUNX3	TJP3	LOC643534	PLTP	KMO	DOK4
S100A13	TLR4	LOC643768	PLXNA3	KNG1	DOK5
S100A3	TLR5	LOC643772	PLXNB2	KNTC1	DOLPP1
SAFB	TMC8	LOC643882	PMP22	KPNB1	DONSON
SAMD4B	TMCC2	LOC643897	POLD1	KRAS	DPAGT1
SAP30BP	TMCO6	LOC643905	POLD2	KRIT1	DPEP3
SAR1A	TMED10	LOC644029	POLE3	KRT17	DPF2
SBSN	TMED9	LOC644126	POLG	KRT222	DPH3
SCARNA16	TMEM100	LOC644128	POLR3C	KRT24	DPH5
SCARNA3	TMEM117	LOC644132	POLR3K	KRT75	DPM2
SCD5	TMEM119	LOC644162	POLS	KRTAP10-2	DPM3
SCN2B	TMEM137	LOC644250	POM121L4P	KRTAP4-8	DPP4
SCRG1	TMEM191C	LOC644343	POMGNT1	L1CAM	DPP7
SCUBE3	TMEM204	LOC644353	POMT1	LAMA3	DPP9
SCYL2	TMEM26	LOC644500	PPAPDC2	LAMB3	DPPA2
SCYL3	TMEM33	LOC644511	PPARD	LAMC3	DPPA3
SDCCAG1	TMEM4	LOC644584	PPIF	LANCL1	DPPA4
SDF2	TMEM43	LOC644590	PPIL5	LAPTM5	DPY19L3
SDHD	TMEM8	LOC644591	PPM1A	LARP4	DPY30
SDK1	TMIE	LOC644619	PPP1CA	LASS1	DPYS
SEC23A	TMPRSS9	LOC644642	PPP1R10	LASS2	DPYSL4
SEC31A	TNF	LOC644686	PPP1R12C	LAT2	DRD1IP
SEMA3B	TNFAIP8L3	LOC644689	PPP1R14A	LAYN	DRG1
SEMA3C	TNFRSF11B	LOC644733	PPP1R3F	LBA1	DSCC1
SEMA4B	TNFRSF6B	LOC644738	PPP1R8	LBP	DSCR1L1
SEMA6C	TNPO2	LOC644949	PPP1R9A	LBR	DSCR6
SEMA6D	TOMM7	LOC644990	PPP2R2D	LBX2	DSE
SEMA7A	TOP1	LOC644992	PPP3CB	LCAT	DTD1
SEP15	TOP1P1	LOC645094	PPP3CC	LCMT2	DTNA
SEPT1	TOP1P2	LOC645098	PQBP1	LCN12	DTNBP1
SERHL	TOR1A	LOC645128	PQLC2	LDOC1	DTWD1
SERINC3	TPD52	LOC645289	PRIC285	LEAP-2	DTWD2
SERPINE3	TPK1	LOC645312	PRKCA	LECT2	DTX2
SETBP1	TRAF3IP1	LOC645330	PRKCB	LEMD2	DTX3L
SF1	TRAPPC6B	LOC645362	PRKCB1	LENG8	DULLARD

SF3A1	TRIM32	LOC645452	PRKCH	LEPR	DUSP11		
SF3B2	TRIM39	LOC645478	PRKCQ	LEPROTL1	DUSP12		
SFMBT2	TRIM58	LOC645522	PRMT6	LGALS3BP	DUSP14		
SFRS10	TRIM62	LOC645566	PRMT7	LGMN	DUSP26		
SFRS12	TRIM66	LOC645671	PRPF19	LIG1	DUSP28		
SGK	TRMT12	LOC645676	PRPS1	LILRB3	DUSP6		
SGK1	TRPA1	LOC645726	PRPS2	LIMK1	DYNC1H1		
SGSH	TSPAN32	LOC645762	PRRG4	LIMS1	DYNC2LI1		
SGSM2	TTC35	LOC645848	PRUNE	LIN52	DYRK1B		
SGSM3	TTC9	LOC645937	PSAP	LINCR	DYSF		
SH2B1	TTLL7	LOC646044	PSEN2	LIPA	DZIP1		
SH3BP5	TTTY11	LOC646109	PSG5	LIPC	E2F5		
SHROOM2	TTTY5	LOC646214	PSMB8	LIX1L	E2F7		
SIGLEC6	TUBAL3	LOC646272	PSMC6	LLGL1	EBI3		
SIRT4	TUBB1	LOC646276	PSMD4	LMAN2	ECD		
SIVA	TYW1B	LOC646330	PSMG4	LMO2	ECEL1		
SIVA1	UBE2H	LOC646346	PTCD1	LMO3	ECGF1		
SLC12A5	UBP1	LOC646482	PTGFR	LNX2	ECH1		
SLC15A2	UBTD2	LOC646483	PTGR2	LOC10012789	ECHDC2		
SLC1A6	UCP3	LOC646547	PTMS	LOC10012801	ECM1		
SLC22A11	UFC1	LOC646576	PTN	LOC10012833	ECOP		
SLC26A8	ULBP2	LOC646585	PTPN23	LOC10012842	EDEM1		
SLC2A5	UNC13D	LOC646665	PTPN3	LOC10012865	EDG1		
SLC2A8	UNC84A	LOC646672	PTRH1	LOC10012869	EDG4		
SLC35F5	UPK3A	LOC646675	PTS	LOC10012891	EDIL3		
SLC39A2	UPP2	LOC646743	PTTG1	LOC10012897	EDN1		
SLC6A4	UROS	LOC646836	PTTG3P	LOC10012912	EDNRB		
SLC7A4	USP45	LOC646908	PUS7	LOC10012921	EED		
SLCO6A1	VAV1	LOC646916	PZP	LOC10012946	EEF1A2		
SMAD3	VDAC1	LOC646936	QARS	LOC10012955	EEF1B2		
SMAD9	VN1R1	LOC646942	QDPR	LOC10012958	EEF1E1		
SMCR7L	VNN2	LOC646982	QPRT	LOC10012958	EFCAB4A		
SMG5	VPREB1	LOC646996	RAB11FIP3	LOC10012959	EFCBP1		
SMO	VPS26B	LOC647000	RAB11FIP5	LOC10012988	EFEMP1		
SMPX	VPS29	LOC647060	RAB18	LOC10012989	EFHD2		
SMTNL2	VPS36	LOC647104	RAB26	LOC10012998	EFNA1		
SMUG1	VPS37A	LOC647229	RAB7L1	LOC10013011	EFNB2		
SND1	VSIG4	LOC647276	RAB8A	LOC10013028	EFNB3		
SNHG11	VWC2	LOC647357	RABAC1	LOC10013029	EFS		
SNORA24	WBSCR22	LOC647363	RABGAP1	LOC10013050	EGFLAM		
SNORA25	WDR25	LOC647389	RABGEF1	LOC10013051	EGR1		
SNORA3	WDR44	LOC647509	RANGAP1	LOC10013092	EHD1		
SNORA32	WDR5	LOC647570	RASA1	LOC10013098	EHD4		
SNORA52	WDR87	LOC647588	RASGRP1	LOC10013113	EHHADH		
SNORA55	WDR89	LOC647704	RASGRP4	LOC10013144	EIF1AX		
SNORA6	WFDC1	LOC647718	RASSF6	LOC10013147	EIF1B		
SNORA62	XLKD1	LOC647834	RASSF7	LOC10013151	EIF2AK1		
SNORA66	YIPF1	LOC647854	RBBP6	LOC10013157	EIF2B3		
SNORA70C	YOD1	LOC647910	RBBP7	LOC10013160	EIF2S3		
SNORA7A	YPEL4	LOC647965	RBM12	LOC10013171	EIF3I		

SNORD104	YTHDF1	LOC648059	RBM39	LOC10013173	EIF3K
SNORD13	ZBED4	LOC648153	RBM5	LOC10013183	EIF3M
SNORD14A	ZBTB45	LOC648226	RBM6	LOC10013197	EIF4E
SNORD32A	ZBTB8A	LOC648517	RBP7	LOC10013206	EIF4E3
SNORD56	ZC3H7A	LOC648600	RC3H2	LOC10013211	EIF4EBP1
SNORD57	ZEB2	LOC648659	RCBTB2	LOC10013211	ELF1
SNORD6	ZFAND3	LOC648691	RCN3	LOC10013232	ELF3
SNORD65	ZFP3	LOC648732	RDBP	LOC10013236	ELF5
SNUPN	ZFP92	LOC648742	RELL2	LOC10013242	ELL
SNX13	ZFPL1	LOC648744	RENBP	LOC10013247	ELL2
SNX6	ZFPM1	LOC648749	RERG	LOC10013249	ELOVL1
SOD3	ZMAT2	LOC648852	RFC5	LOC10013272	ELP4
SOS1	ZNF131	LOC648874	RFXANK	LOC10013277	ELTD1
SOX17	ZNF140	LOC648921	RHBDL2	LOC10013279	EMD
SP6	ZNF250	LOC649044	RHOB	LOC10013283	EMG1
SPAG16	ZNF37A	LOC649071	RHOBTB3	LOC10013293	EMILIN2
SPATA20	ZNF438	LOC649076	RHOC	LOC10013294	EML1
SPATA22	ZNF460	LOC649160	RHOD	LOC10013305	ENAH
SPCS1	ZNF48	LOC649214	RHOU	LOC10013343	ENDOD1
SPDYA	ZNF496	LOC649238	RHPN2	LOC10013351	ENO2
SPIN2B	ZNF579	LOC649431	RIOK3	LOC10013355	ENO3
SPNS3	ZNF583	LOC649456	RMI1	LOC10013357	ENOPH1
SPPL2B	ZNF585B	LOC649495	RMST	LOC10013367	ENPP2
SPPL3	ZNF597	LOC649497	RNASEH2B	LOC10013374	ENSA
SPRR1B	ZNF696	LOC649503	RNF122	LOC10013382	ENTPD4
SPTBN2	ZNF7	LOC649540	RNF126	LOC10013384	ENTPD5
SRCAP	ZNF75A	LOC649711	RNF141	LOC10013397	EP400
SRD5A2L2	ZNF8	LOC649801	RNF144	LOC10013404	EPAS1
SRM	ZSCAN29	LOC649821	RNF169	LOC10013410	EPB41L5
SRP14P1	ZSWIM5	LOC649853	RNF181	LOC10013426	EPHA1
SRP68	ZSWIM7	LOC649859	RNF185	LOC10013426	EPHA2
SRRD	ZZZ3	LOC649873	RNF214	LOC10013453	EPHB1
SRRM2		LOC650034	RNF26	LOC10013463	EPHB2
SSNA1		LOC650132	RNF8	LOC10013468	EPHB6
SST		LOC650157	RNFT1	LOC134505	EPM2AIP1
ST6GALNAC2		LOC650254	RNPC3	LOC143543	EPN3
STAM2		LOC650280	RNPEPL1	LOC145853	EPO
STK17A		LOC650293	RNPS1	LOC146177	EPOR
STK32B		LOC650346	RNU105C	LOC146909	EPSTI1
STON1		LOC650491	RNU1-3	LOC147710	ERAL1
STS		LOC650628	RNU1A3	LOC148413	ERBB3
STX19		LOC650681	RNU6-1	LOC150223	ERC1
STX7		LOC650840	RNU6-15	LOC169834	ERCC5
STXBP2		LOC650889	RNY5	LOC199800	ERCC6L
SURF6		LOC650909	RPL14L	LOC202227	ERCC8
SYNM		LOC650930	RPL34	LOC205251	ERF
SYNPO2		LOC651029	RPL36A	LOC221710	ERGIC1
SYNPR		LOC651112	RPPH1	LOC255326	ERH
SYTL3		LOC651285	RPS2	LOC255783	ERI1
TAF1L		LOC651296	RPS21	LOC283788	ERI3

TANC1	LOC651302	RPS27A	LOC284422	ERO1L
TARDBP	LOC651986	RPS27L	LOC284620	ERP27
TAX1BP1	LOC651987	RPS4X	LOC285500	ERVK6
TBC1D13	LOC652002	RPS4Y2	LOC285548	ESAM
TBC1D3G	LOC652175	RPS6KA4	LOC285620	ESCO1
TBC1D3I	LOC652185	RPUSD3	LOC285900	ESRRB
TBCC	LOC652322	RRAD	LOC286157	ETFB
TBX19	LOC652456	RRAGA	LOC286467	ETNK1
TCL6	LOC652545	RRAGC	LOC338799	ETNK2
TCP1	LOC652565	RRAGD	LOC339047	ETV4
TECPR1	LOC652627	RRAS	LOC344741	ETV5
TFAP2B	LOC652694	RRM2	LOC346950	EXOC6
TGFBRAP1	LOC652704	RRP7A	LOC374443	EXOG
TGS1	LOC652712	RSPH3	LOC387647	EXOSC1
THAP11	LOC652722	RSPRY1	LOC387686	EXOSC5
THBS3	LOC652768	RTN1	LOC387763	EXOSC7
THEM2	LOC653082	RUNX2	LOC387841	EXOSC8
THG1L	LOC653086	RUSC2	LOC387882	EXOSC9
THSD3	LOC653147	S100A10	LOC388275	EXTL3
TIMM10	LOC653162	S100A14	LOC388692	EYA3
TIMM23	LOC653199	S1PR3	LOC388820	EZH2
TINAGL1	LOC653232	SAMD14	LOC388907	EZR
TLCD1	LOC653234	SAR1B	LOC389286	F10
TLN2	LOC653270	SBDS	LOC389293	F11R
TM9SF3	LOC653284	SBDSP	LOC389662	F12
TMCC3	LOC653324	SBNO2	LOC389765	F2R
TMCO7	LOC653342	SCAMP2	LOC390414	F3
TMEM104	LOC653354	SCAND3	LOC390530	F7
TMEM126A	LOC653419	SDCBP	LOC390705	FABP5
TMEM160	LOC653489	SDCBP2	LOC391044	FABP5L2
TMEM25	LOC653513	SDHAF1	LOC391075	FABP5L3
TMEM30A	LOC653520	SEC13	LOC391169	FAF1
TMEM30B	LOC653590	SEC14L5	LOC391352	FAH
TMEM35	LOC653650	SEC22B	LOC391532	FAIM
TMEM39A	LOC653658	SEC61A1	LOC391833	FAM105A
TMEM40	LOC653696	SENP5	LOC392221	FAM107B
TMEM41B	LOC653717	SEPT4	LOC392288	FAM108B1
TMEM79	LOC653720	SEPT7	LOC392787	FAM109A
TMEM88	LOC653752	SERPINB1	LOC399748	FAM110A
TMEM99	LOC653773	SETD1A	LOC399959	FAM110B
TMOD3	LOC653829	SETDB1	LOC400163	FAM117B
TMPRSS13	LOC653881	SETX	LOC400389	FAM119A
TMUB2	LOC654109	SFRP1	LOC400713	FAM122A
TncRNA	LOC654123	SFRS15	LOC400836	FAM123A
TNFSF12-TNFSF13	LOC654135	SFRS16	LOC401074	FAM124A
TNRC9	LOC654260	SFRS5	LOC401357	FAM124B
TOR1B	LOC654350	SGSM1	LOC401397	FAM126A
TOR3A	LOC654433	SH2B3	LOC401431	FAM127A
TP63	LOC727815	SH2D4A	LOC401677	FAM129A
TP73L	LOC727820	SH3BP1	LOC402509	FAM133A

TPCN1	LOC727825	SH3KBP1	LOC402560	FAM134A
TPPP3	LOC727899	SH3YL1	LOC402694	FAM134B
TPRKB	LOC727937	SHB	LOC440059	FAM136A
TRAPPC1	LOC727947	SHROOM4	LOC440080	FAM136B
TRAPPC3	LOC728105	SIK1	LOC440313	FAM13A
TREM1	LOC728128	SKA1	LOC440348	FAM149A
TRIL	LOC728138	SLC10A4	LOC440396	FAM149B1
TRIM17	LOC728139	SLC13A4	LOC440563	FAM150A
TRIM41	LOC728160	SLC15A3	LOC440595	FAM151A
TRIM78P	LOC728181	SLC22A18	LOC440928	FAM159B
TRIP11	LOC728208	SLC24A3	LOC441019	FAM160A2
TRMU	LOC728211	SLC25A22	LOC441179	FAM161A
TRNP1	LOC728285	SLC25A3	LOC441237	FAM162B
TSGA10	LOC728288	SLC25A5	LOC441268	FAM164A
TSPAN10	LOC728310	SLC2A1	LOC441294	FAM165B
TSPAN3	LOC728457	SLC2A10	LOC441377	FAM167A
TTC14	LOC728467	SLC30A1	LOC441505	FAM174B
TTC23	LOC728553	SLC30A7	LOC442041	FAM176A
TTC26	LOC728558	SLC31A1	LOC54103	FAM177A1
TTC28	LOC728564	SLC35B2	LOC553137	FAM179A
TTC8	LOC728565	SLC35C1	LOC553158	FAM181B
TTTY6	LOC728672	SLC35C2	LOC554235	FAM188B
TWSG1	LOC728711	SLC35D2	LOC55908	FAM195A
TXNL1	LOC728779	SLC35D3	LOC641765	FAM20C
TXNRD2	LOC728802	SLC38A7	LOC641825	FAM24B
TYW1	LOC728903	SLC44A3	LOC641942	FAM26A
U2AF1	LOC728908	SLC44A4	LOC642076	FAM35A
UBAP2	LOC728973	SLC7A8	LOC642113	FAM38A
UBASH3B	LOC728975	SLC9A6	LOC642160	FAM38B
UBE1	LOC728992	SLCO2A1	LOC642267	FAM3A
UBE2F	LOC729008	SLFN13	LOC642282	FAM3C
UBE2QP2	LOC729081	SLIT2	LOC642377	FAM44B
UBE2W	LOC729090	SMA4	LOC642399	FAM46A
UBE3C	LOC729101	SMAD5	LOC642469	FAM46B
UBQLNL	LOC729120	SMARCD3	LOC642489	FAM46C
UBR2	LOC729252	SMC6	LOC642530	FAM49B
UBR3	LOC729313	SMG7	LOC642567	FAM54A
UCHL5	LOC729340	SMURF1	LOC642590	FAM54B
ULK1	LOC729348	SNORA12	LOC642726	FAM57A
ULK2	LOC729350	SNORA16A	LOC642780	FAM60A
UNC93B1	LOC729417	SNORA41	LOC642852	FAM62C
UPK2	LOC729438	SNORA67	LOC643031	FAM63A
UPK3B	LOC729446	SNORD35B	LOC643047	FAM64A
UPRT	LOC729510	SNORD3D	LOC643145	FAM65B
UQCRFS1	LOC729562	SNORD52	LOC643313	FAM69A
URM1	LOC729580	SNORD89	LOC643396	FAM71E1
USF1	LOC729603	SNORD96B	LOC643438	FAM71F1
USF2	LOC729666	SNRNP200	LOC643452	FAM72A
USP22	LOC729692	SNRNP48	LOC643665	FAM75B
UTP14A	LOC729769	SNRPD1	LOC643831	FAM7A1

VAPB	LOC729793	SNRPD2	LOC643836	FAM83B
VEZF1	LOC729859	SNRPF	LOC643933	FAM84B
VGLL1	LOC729885	SNX33	LOC644033	FAM86A
VGLL4	LOC729933	SOAT1	LOC644086	FAM89A
VIPR2	LOC729970	SOCS6	LOC644150	FAM89B
VIT	LOC729985	SOD2	LOC644191	FAM8A1
VPS11	LOC730045	SON	LOC644297	FAM90A1
VPS13D	LOC730077	SORBS1	LOC644310	FANCD2
VPS16	LOC730176	SOSTDC1	LOC644322	FANCL
VPS33B	LOC730187	SOX12	LOC644464	FAR2
VPS35	LOC730234	SP1	LOC644482	FARP1
VPS37C	LOC730243	SP3	LOC644617	FAS
VPS4B	LOC730254	SP4	LOC644761	FASN
WAC	LOC730255	SPC25	LOC644914	FASTK
WASL	LOC730256	SPCS3	LOC644919	FAT3
WDR19	LOC730291	SPG11	LOC644934	FBL
WDR24	LOC730357	SPG20	LOC645001	FBLN1
WDR61	LOC730387	SPIN4	LOC645118	FBLN5
WDR73	LOC730517	SPINK5	LOC645195	FBLN7
WHSC2	LOC730534	SPRY1	LOC645217	FBN2
WIPI1	LOC730740	SRC	LOC645251	FBN3
WISP1	LOC730841	SRD5A3	LOC645313	FBP1
WIZ	LOC730908	SRF	LOC645351	FBRS
WNT10A	LOC730990	SRGAP2	LOC645430	FBS1
WNT11	LOC730994	SRI	LOC645609	FBXL10
WNT4	LOC731139	SRPR	LOC645897	FBXL11
WNT6	LOC731196	SRPX	LOC645979	FBXL16
XAB2	LOC731724	SRRM1	LOC646089	FBXL2
XKR4	LOC731777	SRXN1	LOC646100	FBXL3
XKR8	LOC731789	SRY	LOC646111	FBXO11
XPC	LOC732146	SSBP4	LOC646197	FBXO17
YIPF5	LOC732424	SSH2	LOC646282	FBXO21
YPEL1	LOC92659	SSRP1	LOC646332	FBXO22
YWHAH	LPCAT3	SSTR2	LOC646347	FBXO28
ZBED2	LPIN3	STAP2	LOC646403	FBXO31
ZBTB4	LPPR2	STAR	LOC646452	FBXO34
ZBTB5	LRAP	STARD7	LOC646508	FBXO6
ZBTB7C	LRDD	STAT3	LOC646527	FBXO7
ZC3H11B	LRP5L	STIL	LOC646630	FCGR3A
ZC3H12B	LRPPRC	STK10	LOC646674	FCGRT
ZDHHC2	LRRC14	STK19	LOC646688	FDFT1
ZDHHC20	LRRC37B2	STK3	LOC646783	FDPS
ZDHHC24	LRRC47	STK35	LOC646784	FDX1
ZDHHC6	LRRC50	STMN3	LOC646821	FEM1C
ZDHHC8	LRRC59	STRA6	LOC646845	FEN1
ZDHHC8P	LRRCC1	STRN3	LOC646981	FER1L3
ZFP161	LRRK2	STS-1	LOC646993	FERMT1
ZFP36L1	LRRTM4	STX12	LOC647042	FERMT2
ZFYVE1	LSM2	STX2	LOC647108	FEZ1
ZFYVE20	LSM5	STX5	LOC647307	FGA

ZMIZ2	LTBP2	SUMO3	LOC647322	FGB
ZNF12	LUC7L	SUPT5H	LOC647786	FGD2
ZNF252	LUC7L2	SV2B	LOC648213	FGD4
ZNF271	LUZP2	SYDE1	LOC648390	FGD5
ZNF282	LYPLA2	SYF2	LOC648526	FGF12
ZNF292	LYRM2	SYMPK	LOC648682	FGF13
ZNF304	LYRM7	SYNCRIP	LOC648740	FGF16
ZNF32	MACROD1	SYNJ2	LOC648771	FGF19
ZNF33A	MAFG	SYPL2	LOC648982	FGF2
ZNF33B	MAGED4B	SYS1	LOC649009	FGF8
ZNF341	MAGEL2	SYT17	LOC649143	FGFR4
ZNF358	MAK	SYT7	LOC649365	FGG
ZNF385A	MAMDC4	TADA1L	LOC649999	FGGY
ZNF410	MAN2A1	TADA2B	LOC650028	FHDC1
ZNF416	MAN2C1	TAF1C	LOC650546	FHL2
ZNF417	MANEA	TBC1D10A	LOC650803	FHOD3
ZNF432	MAP1D	TBC1D14	LOC650832	FIBP
ZNF471	MAP1LC3B	TBC1D2	LOC651198	FILIP1
ZNF511	MAP3K14	TBC1D3C	LOC651333	FKBP11
ZNF529	MAP3K3	TBC1D7	LOC651568	FKBP14
ZNF548	MAPBPIP	TBC1D9B	LOC651575	FKBP15
ZNF559	MAPKSP1	TBK1	LOC651576	FKBP1B
ZNF564	MAPT	TBL2	LOC651621	FKBP2
ZNF580	MARCH5	TBPL1	LOC651659	FKBP3
ZNF586	MARCKS	TCEA2	LOC651697	FKBP4
ZNF587	MARK3	TCF25	LOC651894	FKBP9L
ZNF610	MAX	TCF4	LOC652324	FKSG30
ZNF641	MAZ	TDH	LOC652458	FLAD1
ZNF646	MBIP	TDRD1	LOC652669	FLJ10081
ZNF650	MBLAC2	TDRKH	LOC652790	FLJ10088
ZNF703	MCM3APAS	TEX15	LOC652819	FLJ10916
ZNF749	MDC1	TFB2M	LOC652900	FLJ12078
ZNF76	MDM2	TGFB1I1	LOC653071	FLJ13305
ZNF776	MEA1	TGM1	LOC653103	FLJ14712
ZNF785	MED11	THAP7	LOC653158	FLJ20125
ZNF79	MED17	THEM4	LOC653375	FLJ20254
ZNF807	MED26	THNSL2	LOC653382	FLJ20273
ZNF813	MED9	THOC2	LOC653458	FLJ20674
ZNRF2	MEGF8	THOC4	LOC653498	FLJ20699
ZNRF3	MEPCE	THRSP	LOC653609	FLJ20920
ZSCAN5A	METAP2	TICAM2	LOC653610	FLJ21986
	METRN	TIGD2	LOC653635	FLJ22184
	METT10D	TIMM8A	LOC653874	FLJ22536
	METT5D1	TIMP1	LOC653879	FLJ22662
	MEX3B	TIPARP	LOC653968	FLJ22795
	MFI2	TM6SF2	LOC654042	FLJ25404
	MFSD2	TM9SF1	LOC654074	FLJ30092
	MGC12982	TM9SF4	LOC654096	FLJ30428
	MGC13005	TMC6	LOC654164	FLJ35024
	MGC27348	TMCO3	LOC654174	FLJ35767

MGC42367	TMED4	LOC654194	FLJ38717
MGC42630	TMED7	LOC727726	FLJ39653
MGC48637	TMEM127	LOC727768	FLJ40194
MGC57359	TMEM131	LOC727848	FLJ40504
MGRN1	TMEM135	LOC727908	FLJ41481
MIA3	TMEM14C	LOC727987	FLJ42957
MIER2	TMEM158	LOC728014	FLJ44379
MIF	TMEM163	LOC728034	FLJ45244
MIR1185-1	TMEM177	LOC728037	FLJ45337
MIR1253	TMEM181	LOC728226	FLJ90231
MIR1282	TMEM185A	LOC728247	FLNB
MIR129-2	TMEM188	LOC728290	FLNC
MIR1909	TMEM189	LOC728324	FLRT2
MIR193A	TMEM192	LOC728362	FLRT3
MIR219-2	TMEM2	LOC728431	FLVCR1
MIR29B1	TMEM208	LOC728441	FMO1
MIR330	TMEM222	LOC728476	FMO4
MIR345	TMEM51	LOC728533	FNBP1L
MIR373	TMEM52	LOC728678	FNDC5
MIR383	TMEM59	LOC728723	FNIP1
MIR635	TMEM70	LOC728728	FOLH1
MIR98	TMEM93	LOC728743	FOLR1
MIS12	TMEM98	LOC728755	FOS
MLLT4	TMF1	LOC728787	FOSB
MLX	TMPO	LOC728809	FOSL1
MMAA	TMSB10	LOC728835	FOXA1
MMP3	TMUB1	LOC729009	FOXA2
MMP7	TNFAIP1	LOC729020	FOXD1
MOAP1	TNFAIP3	LOC729021	FOXI2
MOCS3	TNFSF15	LOC729102	FOXK1
MOGAT1	TNK2	LOC729137	FOXL2
MORC2	TNKS1BP1	LOC729351	FOXO3
MORN2	TOB2	LOC729366	FOXO4
MOSPD1	TOMM34	LOC729409	FOXP1
MPG	TOMM40L	LOC729559	FOXQ1
MPI	TOMM5	LOC729679	FRAS1
MPND	TOP2A	LOC729742	FRAT2
MPPE1	TOP3B	LOC729760	FREM2
MPPED1	TOR1AIP2	LOC729776	FRMD6
MPV17L	TRAFD1	LOC729806	FRMD8
MRC2	TRAM1	LOC729816	FRYL
MRPL17	TRAM2	LOC729887	FSCN1
MRPL19	TRIM11	LOC729952	FSD1
MRPL34	TRIM26	LOC730024	FST
MRPL41	TRIM5	LOC730032	FSTL1
MRPS27	TRIM56	LOC730101	FTCD
MRPS33	TRIM6	LOC730102	FTHL12
MSH3	TRIM8	LOC730110	FTSJ1
MSH5	TRIOBP	LOC730183	FUK
MSH6	TROVE2	LOC730226	FURIN

MST1R	TSC22D1	LOC730246	FUT1
MT1JP	TSC22D2	LOC730284	FUT4
MT3	TSKU	LOC730286	FUT8
MTBP	TSNAX	LOC730535	FXR1
MTDH	TSPAN15	LOC730805	FXYD1
MTM1	TSPAN4	LOC731096	FXYD5
MTPN	TSPAN8	LOC731231	FXYD7
MTRF1	TSPYL1	LOC731542	FYCO1
MTRF1L	TSSC4	LOC731656	FYN
MUC2	TTC12	LOC731915	FZD2
MUC20	TTC17	LOC731950	FZD3
MUCL1	TTYH3	LOC731969	FZD6
MUDENG	TUBA1A	LOC81691	FZD7
MUL1	TUBA4A	LOC85389	FZR1
MYCBP	TUG1	LOC90925	G0S2
MYH1	TUSC1	LOC91431	G3BP2
MYL12A	TUSC2	LOC91664	G6PD
MYLPF	TWF2	LONRF3	GABARAPL1
MYO3B	TXNDC14	LPA	GABPB2
NADK	TXNIP	LPCAT4	GABRA5
NAGK	TXNRD1	LPGAT1	GABRB3
NANOS1	U1SNRNPBP	LPHN3	GABRP
NANP	U2AF1L4	LPPR1	GAD1
NAPA	UBA1	LRCH1	GADD45A
NARG1L	UBA52	LRCH4	GADD45G
NAT11	UBE2C	LRFN5	GAL
NAT2	UBE2L6	LRG1	GAL3ST3
NAT6	UBE2M	LRRC16	GAL3ST4
NBAS	UBE2Q2	LRRC16A	GALC
NBN	UBE2T	LRRC16B	GALM
NBPF1	UBE4B	LRRC31	GALNT10
NBPF15	UBL3	LRRC37A	GALNT12
NCAPD3	UBL7	LRRC3B	GALNT14
NCAPH2	UBN2	LRRC6	GALNT4
NCDN	UBQLN3	LRRC8D	GALNTL1
NCF1	UBXN11	LRRC8E	GALR1
NCRNA00094	UBXN8	LRRN2	GALR2
NCSTN	UCHL3	LSAMP	GALT
NDE1	UCN	LSM14A	GAMT
NDFIP1	UCP1	LSMD1	GAP43
NDUFA4L2	UGCG	LSP1	GAPDHL6
NDUFAF1	UGCGL2	LTA4H	GARNL4
NDUFB1	UNC119B	LTB	GARS
NDUFB2	UNKL	LUZP1	GART
NDUFB5	UQCRC2	LXN	GATA3
NDUFS8	UQCRH	LY96	GATA4
NDUFV3	URG4	LYRM1	GBA
NEB	USHBP1	LYRM5	GBF1
NEIL1	USP1	LYZ	GBGT1
NEIL2	USP14	LZTFL1	GBP2

NEK1	USP16	M6PRBP1	GBP4
NEK10	USP21	MACF1	GBX2
NFATC2IP	USP35	MAD1L1	GC
NFE2L2	USP42	MAF	GCDH
NFYC	USP49	MAFB	GCGR
NGB	USP5	MAGEE1	GCH1
NHLRC2	USP6	MAGEF1	GCHFR
NIT1	UTP14C	MAGI2	GCLM
NKIRAS2	UTY	MAK10	GCNT1
NKRF	VAMP3	MAL	GCNT2
NLK	VAMP4	MAMLD1	GCNT3
NLRP8	VAV2	MAN2A2	GDF3
NMI	VBP1	MAOB	GDI1
NOD2	VCPIP1	MAP1S	GDPD1
NOL7	VIPR1	MAP2K5	GDPD2
NOL8	VMO1	MAP2K7	GEM
NOP14	VPS13C	MAP3K10	GFPT1
NOS2	VPS18	MAP3K15	GFPT2
NOS2A	VPS39	MAP3K7IP3	GFRA1
NOTCH2	VSNL1	MAP9	GFRA2
NOTCH2NL	VWA1	MAPK14	GFRA3
NPAS2	WASF2	MAPKAPK2	GGCT
NPAT	WASPIP	MAPRE1	GGNBP2
NPDC1	WBP5	MARCH8	GGPS1
NPHP4	WBSCR27	MARCKSL1	GH1
NR2C2	WDFY3	MASP1	GH2
NR2C2AP	WDR18	MASP2	GHDC
NR3C2	WDR27	MAST3	GHR
NR4A2	WDR33	MAST4	GINS4
NR6A1	WDR37	MASTL	GIT1
NRTN	WDR51A	MAT1A	GIT2
NSFL1C	WDR51B	MBD3	GJA1
NT5C2	WDR57	MBL1P1	GJB1
NUB1	WDR5B	MBL2	GJB2
NUBP1	WDR6	MBNL1	GJC1
NUBP2	WDR67	MBP	GK
NUBPL	WDR70	MCAT	GLA
NUCKS1	WDR74	MCCC2	GLB1L3
NUDT16P	WIF1	MCM8	GLCE
NUDT2	WIPF1	MCOLN1	GLI1
NUDT4	XAGE2B	MCOLN2	GLI2
NUFIP2	XBP1	MCOLN3	GLIPR1L1
NUP133	XRCC1	ME1	GLIPR2
NUP188	XRCC2	ME3	GLIS3
NUP210	XRCC6	MEAF6	GLRX
NXPH4	XRCC6BP1	MECR	GLRX3
NXT1	YBX1	MED13	GLS2
OASL	YPEL5	MED14	GLT25D1
OGDHL	ZADH2	MED20	GLT25D2
OLA1	ZBED1	MED21	GLT8D2

OPA3	ZBTB34	MED25	GLTPD1
OR10J1	ZBTB42	MED29	GLYATL1
OR1B1	ZBTB43	MED6	GLYCTK
OR1S2	ZC3H12A	MED8	GMDS
OR4M1	ZC3H12C	MEG3	GMPR
OR51S1	ZCCHC3	MEIS3P1	GNA11
OR52N2	ZCCHC6	MESP1	GNA12
OR52N5	ZCCHC9	METAP1	GNA14
OR6M1	ZDHHC18	METT11D1	GNAQ
OR9A4	ZDHHC3	METTL4	GNE
P2RY11	ZFAND5	METTL7A	GNG10
PABPC5	ZFC3H1	METTL7B	GNG11
PACRGL	ZFP112	METTL9	GNG12
PALM2	ZFYVE16	MFAP3L	GNG4
PAN3	ZFYVE19	MFF	GNG5
PAPD5	ZFYVE26	MFGE8	GNL3
PAPOLG	ZKSCAN1	MFHAS1	GNPTAB
PAPSS2	ZMIZ1	MFN1	GNPTG
PARG	ZMYM3	MFSD10	GOLIM4
PARS2	ZNF121	MFSD6L	GOLPH4
PATE1	ZNF160	MGC102966	GOLSYN
PATL1	ZNF212	MGC12965	GOLT1A
PAX8	ZNF22	MGC13057	GPAM
PAX9	ZNF236	MGC15634	GPATCH4
PBK	ZNF248	MGC16121	GPBP1
PBXIP1	ZNF260	MGC18216	GPBP1L1
PCCA	ZNF266	MGC20983	GPC1
PCDH18	ZNF285B	MGC2752	GPC2
PCDHA11	ZNF319	MGC35361	GPC3
PCDHA4	ZNF337	MGC4677	GPC4
PCDHAC2	ZNF362	MGC70857	GPER
PCDHB19P	ZNF443	MGEA5	GPM6B
PCF11	ZNF512	MGMT	GPN3
PCGF1	ZNF513	MICALL2	GPNMB
PCK1	ZNF514	MID2	GPR114
PCM1	ZNF518B	MIOS	GPR126
PCMTD2	ZNF521	MIPOL1	GPR143
PCSK1N	ZNF552	MIR1208	GPR160
PDCD5	ZNF557	MIR130A	GPR161
PDE8B	ZNF577	MIR205	GPR177
PDGFD	ZNF582	MIR21	GPR19
PDIA4	ZNF594	MIR574	GPR3
PDK2	ZNF606	MIR599	GPR37
PDLIM5	ZNF629	MIR877	GPR56
PDS5A	ZNF654	MIR92A2	GPR63
PDS5B	ZNF662	MKNK2	GPR64
PDSS2	ZNF684	MKRN1	GPR83
PDZK1IP1	ZNF689	MKS1	GPR89A
PELP1	ZNF706	MLH3	GPR98
PER1	ZNF763	MLLT10	GPRC5B

PER2	ZNF787	MLLT11	GPX2
PEX13	ZNF816A	MLN	GPX8
PEX16	ZNF823	MLYCD	GRAMD1A
PFKFB3	ZNF841	MMGT1	GRB10
PGAP1	ZNRD1	MMP23A	GRB14
PGK1	ZWINT	MMS19L	GRHL2
PGM2L1		MN1	GRIA3
PGRMC1		MOBKL2B	GRIK5
PHB		MOBKL2C	GRINA
PHF11		MOGAT3	GRK5
PHLPP2		MORN4	GRM4
PHYHD1		MOSPD3	GRM8
PIGM		MPL	GRPEL2
PIGP		MPST	GRPR
PIGR		MPV17	GSDMB
PIGU		MR1	GSDMD
PIGX		MRAP2	GSG2
PIK3CD		MREG	GSN
PIP5K2B		MRP63	GSPT2
PITPNA		MRPL14	GSR
PITPNB		MRPL22	GSTA1
PKNOX2		MRPL23	GSTA2
PLA2G2D		MRPL24	GSTA3
PLDN		MRPL40	GSTA5
PLEKHA7		MRPL51	GSTK1
PLEKHG2		MRPL52	GSTM3
PLEKHN1		MRPL54	GSTO2
PLEKHO2		MRPS14	GSTT1
PLIN2		MRPS18C	GSTT2
PLSCR3		MRPS28	GSTZ1
PMPCA		MRPS31	GTF2B
PNPLA3		MRPS36	GTF2E2
PNPLA4		MS4A6A	GTF2F2
PNPT1		MSRA	GTF2H2
PNRC1		MSRB2	GTF2H2B
POL3S		MSTP9	GTF2H3
POLA2		MSX2P1	GTF2IP1
POLD3		MT2A	GTF2IRD2P
POLDIP3		MTA1	GTPBP3
POLL		MTAP	GTPBP8
POLM		MTCH1	GUCA1B
POLN		MTCH2	GUF1
POLQ		MTCP1	GULP1
POLR1C		MTE	GXYLT1
POLR2A		MTERF	GYG1
POLR2B		MTHFS	GYPC
POLR2G		MTM	H19
POLR2H		MTMR14	H2AFY
POMP		MTMR15	H6PD
POP4		MTSS1	HABP2

POTEF	MUC6	HADH
POTEG	MUM1	HAGHL
POU2F1	MUPCDH	HAND1
PP14571	MURC	HAPLN1
PPA2	MUT	HAS2
PPCS	MYB	HAS3
PPID	MYCNOS	HAUS6
PPIH	MYH14	HAVCR2
PPM1D	MYL6B	HAX1
PPP1CB	MYOM1	HBEGF
PPP1R11	MYOZ1	HBP1
PPP1R15B	MYST2	HBQ1
PPP1R1C	N4BP1	HBXIP
PPP1R3A	NAAA	HCFC2
PPP2R5A	NAGLU	HCK
PPP3CA	NAGS	HCLS1
PPP6C	NALCN	HCP5
PPPDE2	NAP1L4	HDAC2
PRAMEF17	NAPEPLD	HDAC6
PRAMEF7	NAPRT1	HDAC8
PRDM4	NARG2	HDAC9
PRDX5	NAT8	HDGF
PRKAB1	NBEA	HDGFRP3
PRKAG1	NBPF10	HDHD3
PRKAR1A	NCKAP5	HEATR1
PRKCABP	NCKIPSD	HEATR6
PRKCSH	NCOR2	HEATR7A
PRLR	NCRNA00173	HECW2
PROSC	NDEL1	HELLS
PRPF31	NDRG4	HEPH
PRPF39	NDUFA11	HERC1
PRPF4	NDUFA9	HERC5
PRPF8	NDUFC1	HERC6
PRR22	NDUFC2	HESX1
PRR8	NEBL	HEXIM1
PRRG2	NECAP1	HEY2
PRRT2	NEK2	HFE2
PRSS1	NEU4	HHLA2
PRSS2	NEURL1B	HIATL1
PRSS7	NEXN	HIBCH
PSCA	NFE2L1	HIC2
PSCD2	NFIA	HIF1A
PSMA2	NFIB	HINT3
PSMA7	NFIC	HIP1
PSMC2	NFIX	HISPPD2A
PSMD2	NFKB1	HIST1H1C
PSMD5	NFKBIE	HIST1H2BC
PSMD9	NFKBIL2	HIST1H2BD
PSME2	NFKBIZ	HIST1H2BE
PTER	NFS1	HIST1H2BG

PTGER4	NFXL1	HIST1H2BJ
PTK6	NFYB	HIST1H2BK
PTPLAD2	NGF	HIST2H2AA3
PTPRN	NGFRAP1	HIST2H2AA4
PTPRT	NHEDC2	HIST2H2AC
PUS10	NHS	HIST2H2BE
PWWP2A	NINJ2	HK2
PXDNL	NIPA1	HKDC1
PXMP3	NIPA2	HKR1
QRSL1	NIT2	HLA-B
R3HCC1	NKX2-5	HLA-DOA
RAB10	NLGN2	HLA-DPB2
RAB12	NLGN4Y	HLA-DRA
RAB14	NMT2	HLA-E
RAB1A	NNAT	HLA-F
RAB1B	NOD1	HMBS
RAB21	NOL6	HMG20B
RAB2A	NOMO2	HMGA1
RAB2B	NONO	HMGB3
RAB35	NOS3	HMGCR
RAB40C	NPC1L1	HMGCS1
RABGGTA	NPR2	HMGCS2
RABL3	NPR3	HMGN1
RABL4	NR0B2	HMGN2
RAD1	NR1H2	HMGN4
RAD18	NR1H3	HMGXB4
RAD51L3	NR1H4	HN1
RAD52	NR1I2	HNF1B
RAD54L	NR1I3	HNMT
RAD9A	NR2C1	HNRNPA1
RAI2	NR2E3	HNRNPA1L2
RALGAPA1	NR3C1	HNRNPA3
RALY	NRAS	HNRNPU
RANBP2	NRGN	HNRPDL
RANBP3L	NRK	HNRPLL
RANBP6	NRM	HOMER2
RAP1B	NSF	HOOK1
RAPGEF4	NSMAF	HOXB8
RAPSN	NSUN6	HPGD
RARB	NSUN7	HPN
RASA3	NT5DC3	HPRT1
RASD1	NT5M	HPS3
RASL11A	NTN1	HPS6
RASSF2	NTNG1	HPSE
RASSF4	NUAK2	HPX
RAVER2	NUCB1	HRASLS
RAXL1	NUDT16	HRASLS3
RBAK	NUMBL	HRASLS5
RBBP4	NUP107	HRK
RBBP9	NUP155	HRSP12

RBED1	NUP160	HS3ST3A1
RBL1	NUP205	HS6ST2
RBM17	NUP43	HSCB
RBM26	NUP85	HSD17B10
RBM41	NUP93	HSD17B11
RBMY2FP	NUTF2	HSD17B2
RCP9	NXN	HSD17B8
REC8	OAS1	HSP90AA1
REL	OBFC1	HSPA13
RELB	OBP2B	HSPA14
RELL1	OBSL1	HSPA1A
REV1	OCEL1	HSPA1B
REV3L	ODF2L	HSPA4
REXO2	ODF3B	HSPA4L
RFC1	OKL38	HSPA5
RFPL4A	OLFML3	HSPA6
RFX4	OMA1	HSPA8
RGPD8	OPA1	HSPA9
RGR	OR2W5	HSPB1
RGS14	OR3A2	HSPB8
RGS3	ORC3L	HSPBAP1
RHBDD1	ORM1	HSPBL2
RHOF	ORM2	HSPC111
RIG	ORMDL1	HSPC157
RIPK4	OS9	HTATIP2
RMND5A	OSBP2	HTR2C
RNF114	OSBPL3	HTR3A
RNF115	OSBPL8	HTRA1
RNF13	OSGIN1	HYAL3
RNF148	OSTbeta	IARS
RNF152	OTC	ICA1
RNF165	OVOL2	ICAM4
RNF182	OXER1	ICT1
RNF187	OXSM	ID2
RNF20	OXT	IDH2
RNF207	P2RX7	IDH3G
RNF219	P76	IDI1
RNF25	PA2G4	IDO1
RNGTT	PABPC1	IDS
RNPC2	PABPC1L	IER3
ROBLD3	PABPC3	IER5
ROBO1	PACRG	IFI27
ROM1	PACS2	IFI27L2
RORC	PAH	IFI30
RP11-529I10.4	PAIP1	IFI35
RPA1	PALB2	IFI6
RPA2	PALMD	IFIH1
RPA3	PAN2	IFIT1
RPL23A	PANK1	IFIT2
RPL23AP53	PANX1	IFITM1

RPL28	PAPSS1	IFITM2
RPL3	PAR5	IFITM3
RPL37A	PARD3	IFNAR1
RPL5	PARD6G	IFNAR2
RPL7A	PARM1	IFT88
RPS17	PARN	IGDCC3
RPS23	PARP10	IGDCC4
RPS3	PARP11	IGF2
RPS3A	PARP12	IGF2BP3
RPS4Y1	PARP2	IGFBP2
RPS5	PBLD	IGFBP3
RPS6	PBX4	IGFBP4
RPS6KC1	PC	IGFBP6
RPS8	PCAF	IGFBP7
RREB1	PCDH10	IGSF1
RRM1	PCDH21	IGSF11
RRP8	PCDH24	IGSF3
RSHL3	PCDHA2	IGSF5
RSRC2	PCDHA3	IGSF9
RTEL1	PCDHB18	IKZF4
RTP4	PCDHB3	IL10RB
RUFY1	PCDHGA3	IL11RA
RUNDC1	PCDHGB3	IL13RA1
RUNDC2A	PCGF5	IL15
RUNDC3B	PCLO	IL17RD
RUSC1	PCNP	IL18R1
RWDD2B	PCSK6	IL1A
RYBP	PCYOX1L	IL1R1
SAA1	PDCD7	IL1R2
SAA2	PDCL	IL20RB
SAMD4A	PDDC1	IL27RA
SAMM50	PDE3B	IL32
SASS6	PDE5A	IL34
SAT1	PDE9A	ILF2
SCAF1	PDGFRL	ILF3
SCAMP3	PDHX	IMPAD1
SCAMP4	PDLIM1	INADL
SCAMP5	PDLIM2	INDO
SCAP	PDPR	INF2
SCAPER	PDZD8	ING3
SCARNA14	PEA15	INO80B
SCARNA7	PEBP1	INSIG1
SCARNA9L	PECI	INSIG2
SCFD1	PELO	INS-IGF2
SCNM1	PEX1	IP6K1
SCXA	PFDN1	IPO5
SDC3	PFDN6	IPO7
SDCCAG8	PFKFB1	IQCB1
SDF2L1	PFN2	IQCK
SDHAF2	PGAP3	IQGAP1

SDHB	PGBD2	IQGAP2
SDHC	PGCP	IQSEC1
SDS	PGLYRP2	IRAK1BP1
SEC14L2	PGPEP1	IRF1
SEC24D	PGRMC2	IRF2BP2
SEC61G	PHACS	IRF6
SENP1	PHACTR1	IRF8
SEPT11	PHACTR2	IRF9
SEPT13	PHF14	IRX2
SEPT2	PHF15	IRX4
SERINC2	PHF20	ISCA1L
SERPINB6	PHF21A	ISG20L1
SERPINB8	PHF7	ISL1
SERTAD1	PHKA1	ISL2
SESTD1	PHLDB1	ISOC2
SETD6	PI4K2B	ISYNA1
SETDB2	PI4KAP1	ITCH
SF3A2	PIAS4	ITFG2
SF3B3	PIGB	ITGA1
SF3B4	PIGH	ITGA2
SFRS4	PIGL	ITGA6
SFRS6	PIGQ	ITGA9
SFT2D3	PIK3AP1	ITGAV
SGK2	PIK3C2A	ITGB1
SGMS2	PIK3C2G	ITGB1BP1
SGOL2	PIP4K2B	ITGB1BP3
SH3BP2	PJA1	ITIH1
SH3D19	PKD1L2	ITLN2
SHCBP1	PKIB	ITM2A
SHFM1	PKLR	ITM2B
SHOC2	PKN1	ITM2C
SHPRH	PKN3	ITPKA
SHROOM1	PKP3	ITPR2
SIAE	PLA1A	ITPR3
SIGLEC15	PLA2G10	IVNS1ABP
SIRT7	PLA2G12A	JAG2
SKA2	PLA2G16	JAKMIP2
SKP1	PLA2G1B	JAM2
SLAIN2	PLA2G4C	JARID1A
SLAMF6	PLA2G7	JARID2
SLC11A2	PLAC9	JCLN
SLC12A6	PLAGL2	JMJD1C
SLC12A9	PLCB1	JMJD2B
SLC15A4	PLCB4	JOSD1
SLC16A12	PLCG2	JOSD2
SLC16A13	PLCH1	JPH1
SLC16A2	PLCH2	JPH2
SLC19A1	PLCXD1	JPH3
SLC19A2	PLEK	JUB
SLC1A2	PLEK2	JUN

SLC20A2	PLEKHA5	JUP
SLC22A1	PLEKHG1	KAL1
SLC22A10	PLG	KANK4
SLC22A15	PLGLB1	KAT2B
SLC22A6	PLGLB2	KATNA1
SLC25A30	PLK2	KATNAL1
SLC25A33	PLP2	KATNAL2
SLC25A35	PLSCR4	KCNA5
SLC25A44	PLXDC2	KCNAB1
SLC28A1	PLXNA1	KCND1
SLC29A3	PMM1	KCND2
SLC30A6	PMM2	KCNG1
SLC30A9	PMPCB	KCNG3
SLC33A1	PMS1	KCNIP3
SLC35D1	PNMA6A	KCNJ6
SLC35E3	PNMAL1	KCNJ8
SLC35F3	POLB	KCNK12
SLC37A1	POLD4	KCNK5
SLC38A11	POLDIP2	KCNK7
SLC39A7	POLE	KCNMA1
SLC39A9	POLG2	KCNMB4
SLC41A3	POLR2E	KCNN2
SLC45A2	POLR2J4	KCNQ2
SLC4A5	POLR2L	KCNS3
SLC5A3	POLR3A	KCTD12
SLC5A8	POLR3H	KCTD14
SLC6A13	PON1	KCTD15
SLC9A2	POR	KCTD21
SLC9A3R2	PORCN	KCTD8
SLED1	POU3F1	KDELC1
SLPI	POU6F1	KDELR1
SLU7	PPARGC1A	KDM3B
SMAD4	PPBP	KDM5B
SMARCAL1	PPIB	KHDC1L
SMC3	PPM1K	KHDRBS3
SMCR8	PPM1M	KIAA0020
SMYD2	PPP1R12A	KIAA0090
SMYD3	PPP1R15A	KIAA0114
SNCAIP	PPP1R16A	KIAA0194
SNHG6	PPP2CA	KIAA0232
SNIP1	PPP2R2A	KIAA0247
SNORA11C	PPP2R5E	KIAA0251
SNORA11E	PPWD1	KIAA0367
SNORA26	PRAP1	KIAA0368
SNORA28	PRC1	KIAA0494
SNORA5C	PRCC	KIAA0514
SNORA68	PRDM5	KIAA0528
SNORA72	PREB	KIAA0562
SNORA75	PREPL	KIAA0664
SNORA7B	PREX1	KIAA0672

SNORD17	PRG2	KIAA0773
SNORD3A	PRG4	KIAA0895
SNORD55	PRKAB2	KIAA0913
SNRNP40	PRKACB	KIAA1009
SNTA1	PRKCDBP	KIAA1033
SNX26	PRKCI	KIAA1161
SNX8	PRKX	KIAA1217
SORCS2	PRKY	KIAA1244
SPA17	PRO1853	KIAA1267
SPAG4	PROC	KIAA1274
SPAG4L	PROCA1	KIAA1310
SPAG5	PRODH2	KIAA1324L
SPATA13	PROM1	KIAA1370
SPATA2	PROM2	KIAA1430
SPATA2L	ProSAPiP1	KIAA1467
SPATA5L1	PROX1	KIAA1543
SPCS2	PROZ	KIAA1545
SPPL2A	PRPF38A	KIAA1598
SPRN	PRPF40B	KIAA1644
SPRR1A	PRPF4B	KIAA1683
SPSB3	PRPH	KIAA1712
SPTBN5	PRPH2	KIAA1715
SPTLC2	PRR4	KIAA1804
SQSTM1	PRSS16	KIAA1875
SRA1	PRSS3	KIAA1967
SRD5A2	PSD3	KIAA1984
SRGAP1	PSG9	KIF13A
SRP19	PSMB10	KIF13B
SRP54	PSMB5	KIF15
SRPRB	PSMB9	KIF17
SSBP2	PSPC1	KIF1A
SSBP3	PSRC1	KIF1B
SSFA2	PTCH1	KIF26A
SSTR1	PTCHD1	KIF2A
SSX2IP	PTDSS2	KIF5C
SSX4	PTEN	KIFC1
ST3GAL4	PTHLH	KIRREL2
ST7OT1	PTP4A1	KITLG
ST8SIA1	PTPDC1	KLB
ST8SIA2	PTPN14	KLC3
STAG2	PTPN7	KLF10
STAT4	PTPRD	KLF12
STAU2	PTPRH	KLF13
STK36	PUS7L	KLF2
STK4	PVR	KLF5
STT3A	PWP2	KLF8
STT3B	PXMP4	KLHDC5
STUB1	PXN	KLHDC9
STX10	PYCRL	KLHL12
STX1A	PYGL	KLHL21

STXBP1	QRFPR	KLHL22
SULT1B1	QSOX1	KLHL23
SULT4A1	QTRT1	KLHL7
SULT6B1	R3HDM1	KLHL8
SUMO4	RAB11FIP2	KLK5
SUPT16H	RAB15	KLK6
SUPT4H1	RAB24	KLKB1
SUPV3L1	RAB30	KLRA1
SURF1	RAB34	KLRG1
SUSD4	RAB37	KLRG2
SYCE1L	RAB3GAP2	KPNA2
SYN1	RAB3IL1	KREMEN2
SYNJ2BP	RAB7B	KRT18
SYT3	RACGAP1	KRT18P13
TADA2A	RAD17	KRT18P17
TAF10	RADIL	KRT18P28
TAF13	RAF1	KRT19
TAF5	RAGE	KRT8P9
TAF5L	RAI14	KRTAP6-3
TAF8	RALA	KRTDAP
TAGLN2	RALGAPB	KTELC1
TALDO1	RALGPS1	KTN1
TAOK3	RALYL	KYNU
TAS2R43	RAMP1	L1TD1
TBC1D21	RAPGEF3	L2HGDH
TBC1D5	RAPGEF5	L3MBTL
TBC1D8B	RAPH1	LAMA1
TBCD	RARRES3	LAMA2
TBKBP1	RASAL2	LAMA4
TBL3	RASAL3	LAMA5
TBX10	RASGRP3	LAMB2
TBX15	RASSF5	LAMC1
TCEAL1	RBM15	LAMC2
TCF3	RBM20	LAMP2
TCP11L1	RBM24	LAMP3
TEC	RBM25	LAPTM4B
TEF	RBMS2	LARGE
TFAP2E	RBMS2P	LARP1
TGM3	RBMX	LARP1B
THOC1	RBMX2	LARP6
THOC5	RBP5	LARS
TIAM1	RCAN1	LAS1L
TICAM1	RCAN3	LBH
TIFA	RCBTB1	LCK
TIGD1	RCCD1	LCMT1
TIGD6	RCN1	LCN15
TIGIT	RDH5	LCN2
TIMM17A	RECQL	LCP1
TIMM22	RECQL4	LCTL
TJAP1	REEP4	LDB2

TK1	RELA	LDHB
TLK1	RER1	LDHD
TLR1	RFFL	LDLR
TLR10	RFTN2	LEAP2
TLR3	RFWD2	LECT1
TLR6	RFX1	LEFTY1
TMBIM4	RFX7	LEFTY2
TMCO1	RGL4	LEPREL1
TMED2	RGN	LEPREL2
TMEM1	RGS1	LEPROT
TMEM105	RGS11	LETMD1
TMEM106A	RGS16	LGALS8
TMEM11	RGS20	LGI2
TMEM110	RHBDD2	LHFPL4
TMEM123	RHBDF1	LHX6
TMEM129	RHOG	LIAS
TMEM134	RIC3	LIFR
TMEM138	RILP	LIG3
TMEM161B	RILPL1	LIME1
TMEM169	RILPL2	LIMK2
TMEM184A	RIPK5	LIMS2
TMEM187	RIT1	LIN28
TMEM191A	RLTPR	LIN28B
TMEM191B	RN5S9	LIN37
TMEM199	RND1	LINGO2
TMEM207	RNF10	LIPG
TMEM39B	RNF11	LIPT1
TMEM41A	RNF123	LMBRD1
TMEM45B	RNF126P1	LMCD1
TMEM55B	RNF160	LMF2
TMEM66	RNF19A	LMNA
TMEM83	RNF19B	LMO4
TMEM9	RNF213	LMTK3
TMEM91	RNF215	LOC10012791
TMOD2	RNF32	LOC10012792
TMX3	RNF5	LOC10012798
TNFAIP2	ROD1	LOC10012798
TNFRSF10D	ROR1	LOC10012806
TNFRSF25	RORA	LOC10012808
TNFRSF9	RP2	LOC10012816
TNFSF14	RPH3AL	LOC10012825
TNIP2	RPIA	LOC10012826
TOB1	RPL14	LOC10012826
TOMM22	RPL15	LOC10012827
TOP2B	RPL22L1	LOC10012829
TOP3A	RPL23AP7	LOC10012832
TOPBP1	RPL37	LOC10012835
TOR1AIP1	RPL4	LOC10012841
TP53AIP1	RPN2	LOC10012868
TP53RK	RPRD1B	LOC10012873

TP53TG3	RPS6KA3	LOC10012876
TPSG1	RSAD1	LOC10012877
TPST1	RSBN1L	LOC10012883
TPTE2	RSC1A1	LOC10012888
TPX2	RSF1	LOC10012889
TRA1P2	RSPH10B	LOC10012903
TRAF3	RSPH9	LOC10012906
TRAP1	RTKN	LOC10012911
TRAPPC4	RTKN2	LOC10012913
TREH	RTN4	LOC10012914
TRIM16L	RTP2	LOC10012919
TRIM2	RTP3	LOC10012923
TRIM25	RTTN	LOC10012926
TRIM28	RWDD2A	LOC10012929
TRIM3	RWDD3	LOC10012965
TRIM31	RXRG	LOC10012965
TRIM6-TRIM34	RYR3	LOC10012967
TRIM73	S1PR1	LOC10012968
TRIML1	S1PR4	LOC10012982
TRIO	SAA4	LOC10012990
TRIP12	SAMD11	LOC10013000
TRIP4	SAMD5	LOC10013007
TRK1	SAMD9	LOC10013009
TRMT5	SAP18	LOC10013013
TRMT6	SARDH	LOC10013017
TRMT61A	SARM1	LOC10013022
TRPC4AP	SAT2	LOC10013023
TRPM4	SAV1	LOC10013029
TRPM8	SBF2	LOC10013056
TRQ1	SC65	LOC10013062
TRR1	SCAND1	LOC10013076
TRUB2	SCAND2	LOC10013076
TSEN2	SCARF2	LOC10013077
TSEN54	SCARNA17	LOC10013082
TSG101	SCCPDH	LOC10013088
TSLP	SCGN	LOC10013090
TSPAN1	SCHIP1	LOC10013091
TSPAN6	SCLT1	LOC10013091
TSPAN9	SCLY	LOC10013113
TSPYL3	SCN4B	LOC10013118
TSPYL6	SCN9A	LOC10013132
TSSK2	SCNN1D	LOC10013157
TSTD1	SCO2	LOC10013160
TTC21A	SCRN1	LOC10013165
TTC36	SDAD1	LOC10013178
TTC5	SDC1	LOC10013180
TTF2	SDC2	LOC10013185
TTLL4	SDK2	LOC10013186
TTRAP	SDR42E1	LOC10013194
TTYH2	SDSL	LOC10013196

TUBA1B	SEC11A	LOC10013203
TUBA3E	SEC14L4	LOC10013209
TUBA4	SEC22A	LOC10013213
TUBD1	SEC23B	LOC10013232
TUFM	SEC61A2	LOC10013239
TXNDC16	SEC62	LOC10013249
TXNDC17	SEL1L	LOC10013251
TXNL4B	SELENBP1	LOC10013252
UBAP2L	SELM	LOC10013253
UBC	SEMA3E	LOC10013256
UBE2A	SEMA4D	LOC10013267
UBE2D4	SEMA4G	LOC10013272
UBE2V1	SEMA5B	LOC10013279
UBQLN1	SEMA6A	LOC10013280
UBQLN4	SENP6	LOC10013290
UBR1	SENP7	LOC10013291
UBR4	SEPT6	LOC10013293
UCKL1	SEPT9	LOC10013299
UFSP1	SEPX1	LOC10013300
UGCGL1	SERP2	LOC10013301
UGT1A1	SERPINA10	LOC10013321
UGT1A10	SERPINA11	LOC10013322
UGT1A3	SERPINA4	LOC10013327
UGT1A4	SERPINA6	LOC10013332
UGT1A6	SERPINA7	LOC10013337
UGT1A7	SERPINB5	LOC10013343
UGT1A9	SERPIND1	LOC10013348
UGT3A1	SERPINE2	LOC10013359
UHRF1BP1	SERPINF1	LOC10013374
UHRF1BP1L	SESN1	LOC10013376
UNC5CL	SETD1B	LOC10013381
UPLP	SEZ6L2	LOC10013383
UQCRHL	SF3B5	LOC10013401
UROC1	SF4	LOC10013407
USP12	SFMBT1	LOC10013410
USP2	SFRS13B	LOC10013413
USP30	SFRS14	LOC10013414
USP34	SFRS18	LOC10013418
USP37	SFRS9	LOC10013419
USP41	SFT2D2	LOC10013429
USP48	SFXN2	LOC10013430
USP6NL	SFXN5	LOC10013430
USP7	SGCB	LOC10013430
USPL1	SGCE	LOC10013436
UTP23	SGMS1	LOC10013439
VARS	SGTB	LOC10013452
VCP	SH2D3A	LOC10013453
VEGFA	SHISA5	LOC10013481
VEGFC	SHMT1	LOC10019098
VPRBP	SIAH1	LOC123688

VPS26A	SIKE	LOC124512
VPS37B	SIPA1L3	LOC126767
VPS37D	SIRPA	LOC133993
VPS45	SIX4	LOC144481
VPS72	SKAP1	LOC146439
VTRNA1-1	SKIL	LOC147645
VWA3A	SKIV2L2	LOC147646
WASF1	SLAMF9	LOC148709
WBP4	SLC14A1	LOC149134
WBSCR16	SLC15A1	LOC149448
WBSCR19	SLC16A1	LOC153561
WDR40B	SLC16A10	LOC157627
WDR48	SLC16A9	LOC158160
WDR62	SLC17A1	LOC158345
WDR65	SLC17A2	LOC168474
WDR7	SLC17A3	LOC196752
WDR76	SLC17A4	LOC201725
WDR77	SLC17A9	LOC202134
WHSC1L1	SLC22A18AS	LOC202781
WIBG	SLC22A25	LOC220115
WNT8A	SLC22A3	LOC221136
WRN	SLC22A9	LOC23117
WSB2	SLC24A1	LOC283116
WWOX	SLC24A6	LOC283174
XDH	SLC25A1	LOC283340
XIAP	SLC25A10	LOC283663
XIST	SLC25A12	LOC284428
XKRX	SLC25A14	LOC284988
XPO6	SLC25A18	LOC285053
YDJC	SLC25A23	LOC285074
YES1	SLC25A28	LOC285141
YIF1A	SLC25A34	LOC285216
YIPF6	SLC25A36	LOC285359
YTHDC2	SLC25A42	LOC285733
ZBED3	SLC25A43	LOC286208
ZBTB17	SLC25A45	LOC286444
ZBTB26	SLC27A2	LOC338758
ZBTB32	SLC27A3	LOC339290
ZBTB39	SLC29A1	LOC339535
ZBTB44	SLC29A2	LOC339799
ZBTB46	SLC29A4	LOC340274
ZBTB7A	SLC2A2	LOC340970
ZBTB7B	SLC2A3	LOC341230
ZC3H10	SLC2A4RG	LOC341315
ZC3H5	SLC2A9	LOC342934
ZCCHC17	SLC30A10	LOC344595
ZCWPW1	SLC30A5	LOC345041
ZDHHC1	SLC35B1	LOC346887
ZDHHC16	SLC35B4	LOC374395
ZDHHC17	SLC37A4	LOC374491

ZDHHC9	SLC38A2	LOC387683
ZFP1	SLC38A3	LOC387703
ZFP64	SLC38A4	LOC387791
ZHX2	SLC38A9	LOC387820
ZKSCAN3	SLC39A10	LOC387825
ZKSCAN4	SLC39A3	LOC387867
ZKSCAN5	SLC39A8	LOC387934
ZMAT5	SLC3A1	LOC388122
ZMYM6	SLC41A2	LOC388458
ZNF133	SLC44A2	LOC388494
ZNF143	SLC46A1	LOC388524
ZNF174	SLC48A1	LOC388588
ZNF182	SLC4A1AP	LOC388654
ZNF184	SLC4A4	LOC388707
ZNF187	SLC6A1	LOC388755
ZNF223	SLC6A12	LOC389049
ZNF225	SLC6A6	LOC389137
ZNF227	SLC6A8	LOC389141
ZNF233	SLC7A6OS	LOC389168
ZNF234	SLCO1B3	LOC389203
ZNF235	SLCO4C1	LOC389404
ZNF24	SLFN5	LOC389517
ZNF25	SLITRK4	LOC389672
ZNF251	SLMAP	LOC389873
ZNF302	SMARCA4	LOC389895
ZNF320	SMARCA5	LOC390251
ZNF364	SMARCAD1	LOC390466
ZNF385B	SMARCB1	LOC390557
ZNF404	SMARCD1	LOC390578
ZNF418	SMNDC1	LOC390834
ZNF439	SMOC1	LOC391045
ZNF45	SMS	LOC391670
ZNF468	SNAPC1	LOC391769
ZNF480	SNAPC4	LOC392264
ZNF486	SNHG10	LOC392301
ZNF490	SNORA59A	LOC392437
ZNF498	SNORA76	LOC392871
ZNF509	SNORA8	LOC399804
ZNF543	SNORD38A	LOC399942
ZNF549	SNORD73A	LOC399965
ZNF562	SNTB2	LOC399988
ZNF566	SNX16	LOC400214
ZNF576	SNX17	LOC400406
ZNF593	SNX22	LOC400464
ZNF600	SNX24	LOC400506
ZNF615	SNX27	LOC400652
ZNF618	SOCS1	LOC400948
ZNF622	SOCS5	LOC401115
ZNF628	SORBS2	LOC401127
ZNF655	SOX13	LOC401152

ZNF658B	SOX15	LOC401252
ZNF664	SOX2OT	LOC401317
ZNF668	SOX4	LOC401321
ZNF691	SP100	LOC401537
ZNF707	SP110	LOC401622
ZNF711	SP140	LOC401623
ZNF738	SP140L	LOC401717
ZNF772	SP5	LOC402112
ZNF774	SPAG1	LOC402175
ZNF777	SPARCL1	LOC402221
ZNF778	SPAST	LOC402251
ZNF784	SPATA17	LOC402562
ZNF786	SPATS2	LOC439992
ZNF792	SPHK2	LOC440040
ZNF828	SPIC	LOC440043
ZNF860	SPIN1	LOC440061
ZNF98	SPINT2	LOC440093
ZNHIT2	SPIRE1	LOC440132
ZP3	SPN	LOC440145
ZSCAN12L1	SPNS1	LOC440160
ZSWIM3	SPON2	LOC440341
ZSWIM6	SPP2	LOC440345
ZUFSP	SPRED2	LOC440349
	SPRY2	LOC440359
	SPTBN1	LOC440498
	SQRDL	LOC440585
	SRBD1	LOC440731
	SRD5A1	LOC440895
	SRGN	LOC440905
	SRP72	LOC440910
	SRPK2	LOC440993
	SS18	LOC441013
	SSPN	LOC441032
	SSR2	LOC441061
	SSSCA1	LOC441089
	SSX3	LOC441408
	ST3GAL3	LOC441442
	ST7	LOC441453
	STAC	LOC441484
	STAG3L4	LOC441506
	STARD5	LOC441907
	STAT6	LOC442180
	STAU1	LOC442181
	STBD1	LOC442597
	STC2	LOC442609
	STIP1	LOC493869
	STK16	LOC550112
	STK38L	LOC550643
	STOML1	LOC554203
	STRADA	LOC613037

STRADB	LOC641768
STRN	LOC641814
STX11	LOC641849
STX16	LOC642033
STX6	LOC642148
STYK1	LOC642219
STYXL1	LOC642236
SUCLG1	LOC642252
SUDS3	LOC642280
SULF1	LOC642299
SULT1E1	LOC642362
SUSD3	LOC642367
SUZ12	LOC642464
SUZ12P	LOC642559
SWAP70	LOC642570
SYAP1	LOC642661
SYCE2	LOC642817
SYK	LOC642869
SYNE2	LOC642897
SYTL4	LOC642909
TAAR1	LOC642946
TACC3	LOC642956
TACSTD1	LOC643167
TAF12	LOC643272
TAF1B	LOC643287
TAF6	LOC643293
TAF6L	LOC643296
TANK	LOC643319
TAPBPL	LOC643357
TARBP1	LOC643431
TAT	LOC643445
TATDN2	LOC643700
TAZ	LOC643719
TBC1D15	LOC643778
TBC1D22B	LOC643790
TBCB	LOC643873
TCAM1	LOC643896
TCEB3	LOC643918
TCERG1L	LOC643960
TCF1	LOC643995
TCF12	LOC644037
TCF19	LOC644075
TCF7	LOC644124
TCL1B	LOC644131
TCP11L2	LOC644172
TCTA	LOC644214
TCTN1	LOC644237
TDRD3	LOC644254
TEK	LOC644715
TENC1	LOC644743

TEPP	LOC644745
TEX11	LOC644762
TEX261	LOC644852
TEX9	LOC644879
TFAP2C	LOC644928
TFCP2L1	LOC645018
TFR2	LOC645058
TGIF2	LOC645166
TH1L	LOC645173
THADA	LOC645236
THAP10	LOC645262
THAP9	LOC645317
THOC6	LOC645321
THOP1	LOC645323
THPO	LOC645381
THSD4	LOC645385
TIGD5	LOC645431
TIGD7	LOC645436
TIMM8B	LOC645466
TIRAP	LOC645489
TJP1	LOC645638
TK2	LOC645669
TLE1	LOC645682
TLK2	LOC645688
TLR8	LOC645691
TM2D3	LOC645693
TM4SF18	LOC645715
TM4SF4	LOC645781
TM7SF2	LOC645968
TM7SF3	LOC646043
TMBIM1	LOC646294
TMC7	LOC646316
TMED3	LOC646572
TMED5	LOC646723
TMED6	LOC646750
TMEM101	LOC646753
TMEM111	LOC646754
TMEM136	LOC646769
TMEM139	LOC646791
TMEM147	LOC646808
TMEM149	LOC646817
TMEM14A	LOC646849
TMEM154	LOC646956
TMEM168	LOC647150
TMEM171	LOC647169
TMEM176B	LOC647436
TMEM178	LOC647450
TMEM184C	LOC647456
TMEM19	LOC647784
TMEM194	LOC647856

TMEM194A	LOC647859
TMEM20	LOC648057
TMEM205	LOC648210
TMEM216	LOC648249
TMEM220	LOC648399
TMEM233	LOC648605
TMEM37	LOC648695
TMEM45A	LOC648927
TMEM49	LOC649049
TMEM53	LOC649150
TMEM57	LOC649181
TMEM67	LOC649553
TMEM68	LOC649555
TMEM77	LOC649679
TMEM82	LOC649970
TMEM87A	LOC650215
TMEM90B	LOC650298
TMEM92	LOC650369
TMEM9B	LOC650646
TMOD1	LOC650737
TMX4	LOC651149
TNC	LOC651745
TNFAIP8	LOC651816
TNFAIP8L1	LOC652097
TNFRSF10B	LOC652330
TNFRSF12A	LOC652377
TNFRSF14	LOC652470
TNNC1	LOC652489
TNPO1	LOC652492
TNPO3	LOC652570
TNRC6A	LOC652577
TOX	LOC652615
TOX4	LOC652675
TP53AP1	LOC652904
TP53TG1	LOC652968
TPBG	LOC653057
TPCN2	LOC653111
TPM4	LOC653189
TPMT	LOC653192
TRAF1	LOC653226
TRAF3IP2	LOC653257
TRAF6	LOC653337
TRAF7	LOC653344
TREML1	LOC653355
TRIB1	LOC653377
TRIB2	LOC653381
TRIM10	LOC653438
TRIM15	LOC653479
TRIM35	LOC653506
TRIM47	LOC653557

TRIM71	LOC653656
TRIM9	LOC653778
TRIP10	LOC653820
TROAP	LOC653888
T-SP1	LOC653907
TSPAN13	LOC653994
TSPAN31	LOC654053
TSPAN5	LOC654101
TSPYL5	LOC654103
TSSK6	LOC654244
TSTA3	LOC654342
TTC1	LOC678655
TTC25	LOC727758
TTC32	LOC727803
TTC37	LOC727828
TTC4	LOC727865
TTC9C	LOC727866
TTF1	LOC727935
TTLL1	LOC727962
TTLL5	LOC727980
TTTY15	LOC727984
TUBA1C	LOC728026
TUBA3D	LOC728031
TUBGCP3	LOC728059
TXN	LOC728069
TXN2	LOC728115
TXNDC3	LOC728126
TXNL4A	LOC728153
TYK2	LOC728170
TYMP	LOC728275
TYMS	LOC728312
TYROBP	LOC728473
U2AF1L2	LOC728492
UAP1	LOC728554
UAP1L1	LOC728590
UBA6	LOC728591
UBAC1	LOC728620
UBAP1	LOC728635
UBB	LOC728640
UBD	LOC728643
UBE2CBP	LOC728650
UBE2J2	LOC728666
UBE2O	LOC728689
UBE2Z	LOC728698
UBL4A	LOC728715
UBN1	LOC728732
UBR5	LOC728739
UBXN2A	LOC728811
UBXN6	LOC728823
UCK1	LOC728825

UCP2	LOC728873
UFD1L	LOC728887
UGT2A3	LOC728889
UGT2B10	LOC728927
UGT2B15	LOC728931
UGT2B17	LOC728937
UGT2B4	LOC728945
UNC13B	LOC728961
UNC50	LOC729057
UNC5B	LOC729082
UPB1	LOC729086
UPK1A	LOC729130
UQCC	LOC729317
UQCRB	LOC729342
UROD	LOC729378
USE1	LOC729389
USH2A	LOC729406
USP10	LOC729423
USP11	LOC729458
USP13	LOC729500
USP18	LOC729570
USP25	LOC729608
USP39	LOC729660
USP46	LOC729669
USP54	LOC729686
USP9Y	LOC729731
UTP3	LOC729779
UTP6	LOC729960
VAMP5	LOC729964
VARS2	LOC729978
VCAN	LOC730020
VCX	LOC730029
VCX3A	LOC730051
VCX-C	LOC730159
VDAC2	LOC730173
VIP	LOC730235
VMD2L3	LOC730268
VNN1	LOC730278
VNN3	LOC730413
VPREB3	LOC730417
VPS33A	LOC730455
VPS8	LOC730704
VWCE	LOC730746
WAS	LOC730820
WASF3	LOC731314
WBP11	LOC731751
WDFY1	LOC731835
WDFY2	LOC731895
WDR23	LOC731932
WDR42A	LOC731954

WDR45L	LOC732445
WDR47	LOC90624
WDR54	LOC91461
WDR55	LOC91561
WDR68	LOC92249
WDR72	LONP2
WDR75	LONRF1
WDR90	LOX
WDR92	LOXL3
WNK4	LOXL4
WNT5B	LPAR2
WTIP	LPAR3
WWC1	LPAR4
XPA	LPAR5
XPO4	LPHN1
XYLT2	LPHN2
YIPF3	LPIN2
YLPM1	LPPR3
YY1	LQK1
ZBBX	LRAT
ZBTB25	LRCH2
ZBTB48	LRFN4
ZBTB9	LRIG1
ZC3H7B	LRIG2
ZCCHC11	LRP10
ZCCHC7	LRP3
ZCRB1	LRP4
ZDHHC11	LRP5
ZDHHC19	LRP8
ZFAND2B	LRPAP1
ZFAT	LRRC20
ZFHX2	LRRC32
ZFP106	LRRC33
ZFP2	LRRC37B
ZFP37	LRRC40
ZFP82	LRRC49
ZFP90	LRRC58
ZFPM2	LRRC69
ZFX	LRRC8A
ZHX3	LRRFIP2
ZIM2	LRRN1
ZMYM1	LRWD1
ZMYM2	LSM12
ZMYM4	LSM4
ZMYND15	LSM8
ZNF10	LSS
ZNF114	LTBP3
ZNF124	LTBR
ZNF135	LY6E
ZNF14	LYAR

ZNF154	LYPD1
ZNF177	LYPD6
ZNF185	LYPD6B
ZNF193	LYPLA1
ZNF205	LYRM4
ZNF211	LYSMD2
ZNF213	LYSMD3
ZNF219	LZTS1
ZNF254	M6PR
ZNF26	MAD2L1
ZNF263	MAD2L2
ZNF277	MAFF
ZNF28	MAGEC2
ZNF285A	Magmas
ZNF286C	MAGOHB
ZNF322A	MAL2
ZNF326	MALT1
ZNF333	MAMDC2
ZNF334	MAN1A1
ZNF34	MAN1A2
ZNF347	MAN2B2
ZNF397	MANBA
ZNF415	MANBAL
ZNF419	MAOA
ZNF420	MAP1B
ZNF425	MAP1LC3A
ZNF426	MAP1LC3B2
ZNF429	MAP2K3
ZNF43	MAP2K4
ZNF431	MAP2K6
ZNF433	MAP3K11
ZNF434	MAP3K4
ZNF454	MAP3K7
ZNF462	MAP4K1
ZNF484	MAP4K2
ZNF501	MAP4K3
ZNF516	MAP6D1
ZNF518A	MAPK1
ZNF532	MAPK12
ZNF533	MAPK13
ZNF541	MAPK9
ZNF561	MAPKAP1
ZNF570	MAPKAPK5
ZNF571	MAPRE3
ZNF573	MARCH11
ZNF581	MARCH2
ZNF584	MARCH3
ZNF598	MARCH4
ZNF605	MARCH6
ZNF607	MARK1

ZNF609	MARS
ZNF611	MARVELD3
ZNF613	MAST1
ZNF616	MAT2B
ZNF621	MATN3
ZNF630	MATR3
ZNF658	MBD1
ZNF667	MBD2
ZNF671	MBD3L5
ZNF680	MBD6
ZNF700	MBNL2
ZNF701	MBNL3
ZNF708	MBOAT1
ZNF714	MBOAT2
ZNF720	MBOAT7
ZNF721	MC1R
ZNF766	MCART1
ZNF773	MCCC1
ZNF775	MCEE
ZNF783	MCF2
ZNF805	MCL1
ZNF81	MCM2
ZNF821	MCM3
ZNF839	MCRS1
ZRANB2	MCTP1
ZRANB3	MDGA2
ZRSR2	MDK
ZSCAN16	MDN1
ZSCAN18	ME2
ZSWIM4	MED22
	MED23
	MED27
	MED30
	MED31
	MEF2D
	MEGF10
	MEGF6
	MEGF9
	MEIS3
	MELK
	MEP1A
	MEST
	MET
	METRNL
	METTL1
	METTL3
	METTL5
	METTL6
	MFAP2
	MFAP4

MFAP5
MFNG
MFSD1
MFSD5
MGAT1
MGAT2
MGAT3
MGAT4C
MGC11082
MGC15763
MGC24103
MGC26356
MGC33556
MGC39900
MGC40168
MGC40489
MGC57346
MGC61598
MGC71993
MGC72104
MGC87042
MGLL
MGST2
MGST3
MIAT
MICAL1
MICB
MID1
MIF4GD
MIIP
MIR1978
MIR221
MIR302C
MIR302D
MIRLET7D
MITD1
MKI67
MKI67IP
MKLN1
MKNK1
MKX
MLC1
MLF1
MLKL
MLL4
MLPH
MLXIPL
MMAB
MMACHC
MMADHC

MMD
MME
MMP10
MMP2
MMP23B
MMP24
MMP25
MMP9
MMS19
MNAT1
MND1
MNS1
MOBKL3
MOCOS
MON1B
MORC4
MORF4L1
MOSC2
MOSPD2
MOV10
MOXD1
MPDU1
MPP1
MPP2
MPP6
MPRIP
MPZ
MPZL1
MPZL2
MRE11A
MRGPRF
MRI1
MRLC2
MRPL10
MRPL11
MRPL12
MRPL15
MRPL3
MRPL33
MRPL35
MRPL39
MRPL42
MRPL45
MRPL47
MRPL53
MRPL9
MRPS21
MRPS23
MRPS35
MRPS5

MRPS7
MRRF
MRS2
MRTO4
MSH2
MSI2
MSL3
MSL3L1
MST1
MST4
MSX2
MT1A
MT1E
MT1F
MT1G
MT1H
MT1M
MT1X
MTA2
MTA3
MTF2
MTHFD1
MTHFD1L
MTHFD2
MTHFR
MTIF2
MTL5
MTMR10
MTMR11
MTMR2
MTMR3
MTMR4
MTMR7
MTMR9
MTO1
MTR
MTSS1L
MTTP
MTUS1
MTX2
MTX3
MUC1
MUC16
MUC4
MUTYH
MVD
MVK
MVP
MX1
MXD1

MXD3
MXD4
MYC
MYCN
MYD88
MYH11
MYH9
MYL4
MYL5
MYL6
MYL7
MYL9
MYLC2PL
MYLK
MYO10
MYO18A
MYO19
MYO1B
MYO1C
MYO1D
MYO5A
MYO5C
MYO6
MYO7A
MYOF
MYOZ3
MYRIP
MYST3
MYT1
MZF1
N4BP2L1
N4BP3
N6AMT1
N6AMT2
NAB2
NACC2
NAE1
NAGPA
NAMPT
NANOG
NAP1L1
NAP1L2
NAP1L3
NAP1L6
NARS
NARS2
NAT8B
NAT9
NAV1
NAV2

NBL1
NBPF20
NBPF8
NBR2
NCALD
NCAPG
NCBP2
NCCRP1
NCK2
NCKAP1
NCOA4
NCOA5
NCOA6
NCOA7
NCRNA00153
NCRNA00219
NDC80
NDN
NDRG1
NDRG2
NDRG3
NDST1
NDST2
NDUFA1
NDUFA2
NDUFA3
NDUFAF3
NDUFB11
NDUFB3
NDUFB6
NDUFB7
NDUFV2
NECAB1
NEDD1
NEDD4
NEDD9
NEFL
NEFM
NEK3
NEK6
NELF
NELL2
NENF
NEO1
NES
NETO2
NFASC
NFATC1
NFATC3
NFE2

NFE2L3
NFIL3
NFKB2
NFKBIB
NFU1
NFX1
NGEF
NGRN
NHLH2
NHP2
NHP2L1
NID2
NIF3L1
NINJ1
NINL
NIPAL1
NIPSNAP3A
NKAIN4
NKIRAS1
NKX6-2
NLE1
NLGN1
NLGN4X
NLN
NLRP12
NLRP7
NME1
NME1-NME2
NME2
NME3
NME6
NME7
NMNAT2
NMU
NNT
NOC2L
NOC3L
NOC4L
NOL11
NOL3
NOMO1
NOP2
NOP56
NOP58
NOTCH1
NOTCH3
NOVA1
NOX4
NP
N-PAC

NPAL3
NPAS1
NPFFR2
NPIP
NPL
NPM3
NPPB
NPTX2
NPW
NQO1
NR2F2
NR5A2
NRBF2
NRG1
NRIP3
NRN1L
NRP1
NRP2
NRSN2
NSD1
NSDHL
NSMCE4A
NSUN5
NSUN5B
NSUN5C
NT5C
NT5DC1
NT5E
NTM
NTN3
NTRK3
NTS
NUAK1
NUCB2
NUDCD1
NUDCD2
NUDT1
NUDT10
NUDT11
NUDT14
NUDT15
NUDT21
NUDT3
NUDT6
NUDT9
NUFIP1
NUP35
NUP37
NUP54
NUP62

NUP98
NUPR1
NUSAP1
NXF1
NYNRIN
OAF
OAS2
OAS3
OAT
OBFC2A
OCIAD1
OCIAD2
OCRL
ODC1
ODF2
ODZ3
ODZ4
OGDH
OGFR
OGFRL1
OIP5
OLFM1
ONECUT2
OPN3
OPTN
OR2A1
OR2A42
OR2A9P
OR7E156P
ORAI3
ORC2L
ORC5L
ORC6L
ORMDL3
OSBPL10
OSBPL1A
OSBPL2
OSBPL6
OSCP1
OSGEPL1
OSGIN2
OSR1
OSTalpha
OSTM1
OTUB1
OTUB2
OTUD6B
OTX2
OVGP1
OVOS2

OXCT1
OXCT2
OXR1
P15RS
P2RX2
P2RX4
P4HA1
P4HA2
P8
PAAF1
PABPC4L
PACSIN1
PACSIN2
PAFAH1B3
PAG1
PAGE4
PAGE5
PAICS
PAIP2
PAK1
PAK1IP1
PAK2
PAK6
PANX2
PAOX
PAQR3
PAQR4
PAQR9
PARL
PARP1
PARP4
PARP6
PARP8
PARP9
PARVA
PASK
PATE2
PATE3
PAWR
PBRM1
PBX1
PBX3
PCBD1
PCBP2
PCDH1
PCDH11X
PCDH11Y
PCDH19
PCDHA1
PCDHB15

PCDHB17
PCDHB2
PCDHB5
PCGF2
PCGF6
PCID2
PCNA
PCNX
PCNXL2
PCNXL3
PCOLCE
PCOLCE2
PCSK5
PCSK9
PCYT2
PDCD10
PDCD2
PDCD2L
PDCL3
PDE12
PDE4A
PDE4B
PDE4C
PDE6B
PDE6D
PDE7A
PDGFA
PDGFRA
PDIA3P
PDIA5
PDIA6
PDIK1L
PDK3
PDK4
PDLIM3
PDPK1
PDPN
PDSS1
PDXK
PDXP
PDZD2
PDZD4
PDZK1
PDZK1P1
PEAR1
PECR
PEG10
PELI1
PEMT
PERP

PEX11A
PEX11B
PEX7
PFDN4
PFDN5
PFKFB2
PFKL
PFKM
PFKP
PGAM4
PGAM5
PGF
PGS1
PHAX
PHB2
PHC1
PHC2
PHF16
PHF17
PHF2
PHF21B
PHF5A
PHGDH
PHKA2
PHKB
PHLDA1
PHLDA2
PHLDA3
PHLDB2
PHOSPHO2
PHRF1
PHYH
PI15
PI4KB
PIAS2
PIAS3
PIB5PA
PICALM
PID1
PIF1
PIGF
PIGK
PIGZ
PIK3CB
PIK3IP1
PIK3R1
PIK4CA
PIM1
PIM2
PINK1

PINX1
PIP4K2A
PIP5K2A
PITPNC1
PITX1
PITX2
PJA2
PKD2
PKDCC
PKIA
PKIG
PKM2
PKP2
PKP4
PLA2G12B
PLA2G15
PLA2G3
PLAC1
PLAT
PLAU
PLAUR
PLCB2
PLCL2
PLD1
PLD2
PLD6
PLEKHA2
PLEKHA6
PLEKHB1
PLEKHF1
PLEKHG4
PLEKHG6
PLEKHH1
PLOD2
PLP1
PLRG1
PLS3
PLXNB1
PMAIP1
PMEPA1
PMS2CL
PMVK
PNCK
PNKD
PNMA1
PNMA2
PNMA3
PNN
PNPLA2
PNPLA6

PNPLA7
PNPLA8
PNPO
PODXL
PODXL2
POFUT1
POFUT2
POGZ
POLA1
POLE2
POLR1D
POLR1E
POLR2D
POLR2F
POLR3D
POLR3F
POLR3G
POLR3GL
POMC
PON2
PON3
POPDC3
POT1
POU5F1
POU5F1P1
PPAN
PPAP2A
PPAP2B
PPAP2C
PPAPDC3
PPARA
PPARG
PPAT
PPFIBP1
PPFIBP2
PPHLN1
PPIC
PPIL1
PPIL3
PPL
PPM1B
PPM1E
PPM1G
PPM1H
PPM2C
PPOX
PPP1CC
PPP1R13B
PPP1R14B
PPP1R14C

PPP1R16B
PPP1R1A
PPP1R1B
PPP1R3B
PPP1R3C
PPP2CB
PPP2R1A
PPP2R2B
PPP2R2C
PPP2R3B
PPP2R3C
PPP2R4
PPP2R5B
PPP2R5C
PPP2R5D
PPP4R4
PPPDE1
PPT1
PQLC1
PRAC
PRAGMIN
PRAMEF15
PRAMEF4
PRAMEF5
PRAMEF9
PRDM1
PRDM14
PRDX1
PRDX2
PREI3
PREP
PRICKLE1
PRICKLE2
PRICKLE4
PRIM1
PRIM2A
PRKAA1
PRKAG2
PRKAR1B
PRKCD
PRKCZ
PRKD2
PRKDC
PRKRA
PRMT1
PRMT2
PRMT3
PRMT5
PRNPIP
PROCR

PRODH
PROK2
PROS1
PRPSAP2
PRR11
PRR14
PRR16
PRR3
PRR5
PRRT3
PRRX2
PRSS12
PRSS23
PRTFDC1
PSAT1
PSCD1
PSD4
PSEN1
PSG3
PSIP1
psiTPTE22
PSMC3IP
PSMC5
PSMD10
PSMD12
PSMD14
PSMD7
PSME1
PSMF1
PSPH
PSTK
PSTPIP2
PTAR1
PTBP2
PTCD2
PTGES
PTGES2
PTGES3
PTGFRN
PTGIS
PTGR1
PTGS2
PTH1R
PTH2R
PTK2
PTK2B
PTK7
PTMA
PTP4A2
PTP4A3

PTPLA
PTPLB
PTPN12
PTPN13
PTPN2
PTPN6
PTPRA
PTPRE
PTPRF
PTPRG
PTPRK
PTPRM
PTPRZ1
PTRF
PTRH2
PUM1
PUS1
PVRL1
PVRL2
PVT1
PWP1
PWWP2
PWWP2B
PXDN
PXMP2
PYCARD
PYCR1
PYCR2
PYDC1
QKI
QPCT
QTRTD1
RAB11FIP1
RAB17
RAB20
RAB22A
RAB23
RAB25
RAB27A
RAB28
RAB31
RAB32
RAB38
RAB39B
RAB3B
RAB3C
RAB3IP
RAB40B
RAB43
RAB5A

RAB5B
RAB5C
RAB9A
RABGGTB
RAC1
RAC2
RAC3
RAD50
RAD51
RAD51AP1
RAD51C
RAD51L1
RAD54B
RAI1
RALBP1
RALGDS
RAMP2
RAN
RANBP1
RANBP10
RANBP3
RAP1GAP
RAP1GDS1
RARA
RARRES1
RARRES2
RASD2
RASGRF2
RASGRP2
RASIP1
RASL10A
RASL10B
RASL11B
RASL12
RASSF1
RASSF9
RAX
RBBP8
RBCK1
RBKS
RBL2
RBM11
RBM12B
RBM18
RBM3
RBM34
RBM35A
RBM47
RBM9
RBMS1

RBP1
RBP4
RBPJ
RBPMS
RBPMS2
RBX1
RCAN2
RCC2
RCL1
RCN2
RCOR2
RCOR3
RDH10
RDH12
RDH13
RDH16
RDM1
RDX
RECK
RECQL5
REEP1
REEP2
REEP6
REG1A
REP15
REPS2
RERE
RETSAT
RFC2
RFC3
RFC4
RFESD
RFTN1
RFWD3
RFX3
RFXAP
RGL1
RGMA
RGMB
RGS10
RGS12
RGS17
RGS2
RGS4
RGS7
RGS7BP
RHBDF2
RHBDL3
RHEBL1
RHOT1

RICH2
RIF1
RIMKLB
RIMS2
RIMS3
RIMS4
RIN2
RING1
RINL
RIPK1
RIPK2
RIT2
RLN2
RNASE4
RNASEH2A
RNASEK
RNASEN
RND2
RND3
RNF103
RNF125
RNF128
RNF135
RNF144A
RNF144B
RNF145
RNF146
RNF149
RNF150
RNF170
RNF175
RNF217
RNF220
RNF34
RNF38
RNF39
RNF40
RNF41
RNF44
RNF5P1
RNF7
RNFT2
RNH1
RNU105A
RNU86
ROBO3
ROMO1
ROR2
RORB
RP5-1022P6.2

RP9P
RPAIN
RPAP1
RPAP3
RPE
RPESP
RPF2
RPL10A
RPL12
RPL12P6
RPL13
RPL13A
RPL13L
RPL17
RPL22
RPL32
RPL36AL
RPL39L
RPL41
RPL6
RPL7
RPL7L1
RPL8
RPL9
RPLP0
RPP25
RPP38
RPP40
RPRD1A
RPRM
RPRML
RPS15
RPS24
RPS26
RPS26L
RPS26P10
RPS26P11
RPS6KA2
RPS6KA5
RPS6KB2
RPS6P1
RPS7
RPSA
RRAS2
RRBP1
RRM2B
RSL1D1
RSL24D1
RSPO3
RTCD1

RTN3
RTN4IP1
RTN4R
RTP1
RUNX1
RUNX1T1
RUVBL1
RWDD1
RXRA
RYK
RYR1
RYR2
S100A11
S100A16
S100A4
S100A6
S100P
S100PBP
SAAL1
SACS
SAE1
SAFB2
SALL2
SALL3
SALL4
SAMD13
SAP30
SAPS1
SAPS2
SARS
SARS2
SBK1
SC4MOL
SC5DL
SCAMP1
SCARA3
SCARB1
SCARB2
SCARNA10
SCARNA9
SCD
SCG2
SCG3
SCG5
SCGB3A2
SCML1
SCML2
SCN4A
SCNN1A
SCNN1G

SCO1
SCP2
SCPEP1
SCYL1
SDC4
SDCCAG10
SDF4
SDHA
SDHALP1
SDHAP2
SDHAP3
SEC11C
SEC14L1
SEC22C
SEC61B
SEC63
SECISBP2
SECISBP2L
SEL1L3
SELK
SELO
SELS
SELV
SEMA3A
SEMA4A
SEMA6B
SEMG1
SENP8
SEPHS1
SEPHS2
SEPN1
SEPP1
SEPSECS
SEPT10
SEPT3
SEPT5
SEPW1
SERF1A
SERF1B
SERF2
SERINC1
SERPINA1
SERPINA3
SERPINA5
SERPINC1
SERPINE1
SERPINF2
SERPING1
SERPINH1
SERPINI1

SERTAD2
SERTAD3
SERTAD4
SET
SETD3
SF3A3
SF3B14
SFN
SFRP2
SFRS17A
SFT2D1
SFTA1P
SFXN4
SGCG
SGK3
SGOL1
SH2D3C
SH2D5
SH3BGRL
SH3BGRL3
SH3BP4
SH3GL2
SH3GL3
SH3GLB2
SH3PXD2A
SH3PXD2B
SH3RF1
SH3RF2
SH3TC1
SHANK2
SHANK3
SHBG
SHC1
SHC2
SHD
SHISA2
SHISA3
SHMT2
SHQ1
SIDT2
SIGIRR
SIGMAR1
SIK3
SIL1
SILV
SIN3B
SIP1
SIPA1
SIPA1L2
SIRT1

SIRT2
SIRT5
SKAP2
SKP2
SLC10A1
SLC10A3
SLC12A2
SLC13A5
SLC16A14
SLC16A4
SLC16A5
SLC19A3
SLC1A1
SLC1A3
SLC1A5
SLC20A1
SLC22A17
SLC22A5
SLC22A7
SLC23A1
SLC23A2
SLC23A3
SLC25A15
SLC25A16
SLC25A19
SLC25A20
SLC25A21
SLC25A24
SLC25A25
SLC25A26
SLC25A29
SLC25A37
SLC25A39
SLC25A40
SLC25A6
SLC26A11
SLC26A6
SLC27A5
SLC27A6
SLC2A14
SLC2A6
SLC30A3
SLC31A2
SLC35A2
SLC35A4
SLC35A5
SLC35B3
SLC35E1
SLC35F1
SLC35F2

SLC37A3
SLC38A1
SLC38A10
SLC39A1
SLC39A11
SLC39A14
SLC39A4
SLC39A5
SLC39A6
SLC3A2
SLC40A1
SLC43A2
SLC43A3
SLC44A1
SLC45A3
SLC45A4
SLC46A3
SLC47A1
SLC4A11
SLC4A2
SLC4A7
SLC4A8
SLC5A6
SLC5A9
SLC6A10P
SLC6A15
SLC6A16
SLC6A9
SLC7A1
SLC7A10
SLC7A11
SLC7A14
SLC7A2
SLC7A3
SLC7A5
SLC7A6
SLC7A7
SLC7A9
SLC8A2
SLC9A3R1
SLC9A5
SLC9A8
SLCO1A2
SLCO1B1
SLCO2B1
SLCO3A1
SLFN11
SLK
SLMO1
SMA5

SMAD6
SMAD7
SMAGP
SMAP1
SMAP2
SMARCA1
SMARCA2
SMARCC1
SMC2
SMC4
SMCR5
SMG1
SMN1
SMN2
SMOC2
SMOX
SMPD1
SMPDL3A
SMPDL3B
SMTN
SMYD4
SNAI2
SNAP23
SNAP29
SNAP91
SNAPIN
SNCA
SNHG1
SNHG12
SNHG4
SNHG5
SNHG7
SNHG8
SNORA10
SNORA11D
SNORA18
SNORA33
SNORA45
SNORA73A
SNORA73B
SNORA79
SNORA84
SNORD11
SNORD16
SNORD21
SNORD22
SNORD25
SNORD30
SNORD31
SNORD36A

SNORD36C
SNORD48
SNORD76
SNORD80
SNORD96A
SNRK
SNRNP35
SNRPA
SNRPB
SNRPC
SNRPD3
SNRPN
SNTB1
SNURF
SNX1
SNX10
SNX11
SNX25
SNX30
SNX4
SNX5
SOAT2
SOCS2
SOCS3
SOCS4
SOD1
SOHLH2
SOLH
SORCS1
SORD
SORL1
SORT1
SOX10
SOX11
SOX18
SOX2
SOX21
SOX3
SOX7
SOX8
SOX9
SP8
SPARC
SPATA18
SPATA6
SPATA7
SPATS2L
SPC24
SPEN
SPG3A

SPHK1
SPIN3
SPINK1
SPINK2
SPINT1
SPIRE2
SPNS2
SPOCD1
SPOCK1
SPOCK2
SPON1
SPP1
SPR
SPRED1
SPRY4
SPRYD3
SPRYD4
SPRYD5
SPSB1
SPSB2
SPTLC1
SPTLC3
SPTY2D1
SQLE
SREBF1
SRP14
SRP9
SSB
SSPO
SSR4
SSU72
ST13
ST3GAL1
ST3GAL5
ST3GAL6
ST5
ST6GAL1
ST6GALNAC3
ST6GALNAC5
ST6GALNAC6
ST7L
ST8SIA5
STAG3L1
STAMBP
STAMBPL1
STARD10
STARD13
STARD3NL
STARD8
STAT1

STAT2
STC1
STEAP1
STEAP2
STEAP3
STIM1
STK11
STK24
STK25
STK32A
STK33
STK39
STK40
STMN1
STOM
STOML2
STOX2
STRAP
STRBP
STRN4
STX3
STX4
STXBP3
STXBP6
SUCLA2
SUCLG2
SULF2
SULT1A1
SULT1A2
SULT1A3
SULT1A4
SULT2A1
SULT2B1
SUMF1
SUMO1P3
SUMO2
SUOX
SUPT3H
SURF4
SUSD2
SUV420H1
SV2A
SVEP1
SVIL
SVOPL
SYN2
SYN3
SYNGR1
SYNGR3
SYNJ1

SYPL1
SYT1
SYT11
SYT13
SYT15
SYT4
SYT6
SYTL1
SYTL2
SYVN1
T
TAC1
TAC3
TACC1
TAF1
TAF1A
TAF1D
TAF2
TAF4B
TAF7
TAF9L
TAGLN
TAGLN3
TANC2
TAOK1
TAOK2
TAP2
TAPBP
TAPT1
TARBP2
TARS
TATDN1
TATDN3
TBC1D16
TBC1D17
TBC1D20
TBC1D23
TBC1D2B
TBC1D3B
TBC1D8
TBC1D9
TBCE
TBL1XR1
TBP
TBX1
TBX3
TC2N
TCEA1
TCEA3
TCEAL2

TCEAL3
TCEAL4
TCEAL7
TCEAL8
TCEB2
TCERG1
TCF2
TCF7L1
TCIRG1
TCL1A
TCN2
TCP10L
TCTEX1D2
TDG
TDGF1
TDGF3
TDO2
TDP1
TDRD7
TEAD2
TEAD4
TEKT3
TERF1
TERF2IP
TESC
TESK1
TET1
TEX10
TEX2
TEX264
TF
TFAM
TFAP2A
TFB1M
TFCP2
TFG
TFIP11
TFPI
TFPI2
TGFB3
TGFBI
TGFBR2
TGFBR3
TGIF1
TGM2
TGOLN2
THAP1
THBS2
THBS4
THNSL1

THOC3
THUMPD2
THY1
THYN1
TIA1
TIAF1
TIAL1
TIAM2
TIGA1
TIMELESS
TIMM44
TIMM9
TIMP2
TIMP3
TIMP4
TIPIN
TKT
TLE3
TLE4
TLE6
TLN1
TM2D1
TM2D2
TM4SF1
TM4SF5
TM6SF1
TMBIM6
TMEFF1
TMEFF2
TMEM106C
TMEM108
TMEM109
TMEM115
TMEM116
TMEM118
TMEM120A
TMEM125
TMEM126B
TMEM128
TMEM132A
TMEM132B
TMEM132D
TMEM140
TMEM141
TMEM143
TMEM144
TMEM145
TMEM14D
TMEM150A
TMEM155

TMEM159
TMEM165
TMEM166
TMEM167A
TMEM16A
TMEM16B
TMEM16D
TMEM17
TMEM170B
TMEM175
TMEM176A
TMEM179B
TMEM183A
TMEM183B
TMEM184B
TMEM185B
TMEM189-UB
TMEM195
TMEM200A
TMEM206
TMEM209
TMEM214
TMEM217
TMEM218
TMEM22
TMEM27
TMEM31
TMEM38B
TMEM42
TMEM44
TMEM48
TMEM50B
TMEM55A
TMEM56
TMEM59L
TMEM62
TMEM63B
TMEM64
TMEM69
TMEM86B
TMPRSS11E2
TMPRSS2
TMPRSS6
TMSB15A
TMSB4Y
TMTC1
TMTC3
TMX1
TNFRSF10A
TNFRSF1B

TNFRSF21
TNFRSF8
TNFSF10
TNFSF12
TNFSF13B
TNIK
TNIP1
TNMD
TNNC2
TNNI3
TNNT1
TNRC6B
TNS1
TOLLIP
TOM1
TOM1L2
TOMM20
TOMM40
TOMM6
TOP1MT
TOR2A
TOX2
TP53BP1
TP53BP2
TP53INP1
TP53INP2
TPD52L1
TPI1
TPM1
TPM2
TPP1
TPR
TPRG1L
TRADD
TRAF4
TRAF5
TRAIP
TRAK1
TRAK2
TRAM1L1
TRAPPC9
TRDMT1
TRERF1
TRH
TRIAP1
TRIB3
TRIM13
TRIM21
TRIM24
TRIM33

TRIM37
TRIM38
TRIM4
TRIM45
TRIM46
TRIM48
TRIM49
TRIM53
TRIM55
TRIM64
TRIM65
TRIML2
TRIP13
TRMT1
TRMT11
TRO
TRPC1
TRPC6
TRPM6
TRPV6
TRRAP
TSC1
TSC2
TSC22D3
TSC22D4
TSEN15
TSEN34
TSGA14
TSHZ1
TSHZ2
TSHZ3
TSPAN18
TSPAN33
TSPAN7
TSPO
TSPYL2
TSSC1
TST
TSTD2
TTC13
TTC15
TTC19
TTC27
TTC29
TTC38
TTC39B
TTC39C
TTK
TTLL3
TTLL6

TTPAL
TTR
TTYH1
TUB
TUBB
TUBB2A
TUBB2B
TUBB2C
TUBB3
TUBB4
TUBB4Q
TUBB6
TUBB8
TUBE1
TUBG1
TUBGCP6
TULP3
TULP4
TUSC3
TWIST1
TWISTNB
TXNDC11
TXNDC12
TXNL2
TYRO3
TYSND1
U2AF2
UBA2
UBA3
UBA7
UBE1C
UBE2E2
UBE2E3
UBE2G1
UBE2I
UBE2J1
UBE2L3
UBE2V2
UBIAD1
UBL5
UBLCP1
UBR7
UBXN4
UCA1
UCHL1
UCHL5IP
UCK2
UCRC
UFM1
UFSP2

UGDH
UGP2
UGT2B11
UGT2B28
UGT2B7
UGT3A2
UGT8
UHRF1
ULBP1
UNC119
UNC5A
UNC5D
UNC84B
UNC93A
UNG
UNQ9433
UPF2
UPF3A
UPF3B
UPP1
UQCRQ
USO1
USP15
USP24
USP28
USP3
USP32
USP33
USP36
USP38
USP44
USP47
USP53
USP9X
UST
UTF1
UTP15
UTP18
UTS2
UTX
UVRAG
UXT
VAMP1
VAMP2
VAMP8
VANGL1
VANGL2
VAPA
VASH1
VASH2

VASN
VAT1
VAT1L
VAV3
VCL
VEGFB
VENTX
VGF
VHL
VIL1
VIL2
VIM
VKORC1
VLDLR
VOPP1
VPS13A
VPS24
VPS25
VPS28
VPS41
VRK1
VRK3
VTCN1
VTI1B
VTN
VWA5A
WARS
WDHD1
WDR1
WDR12
WDR13
WDR22
WDR34
WDR35
WDR4
WDR41
WDR43
WDR45
WDR46
WDR53
WDR79
WDR8
WDR81
WDR86
WDSOF1
WDSUB1
WDYHV1
WEE1
WFDC2
WFS1

WHAMM
WIPF3
WIPI2
WNK1
WNK2
WNT10B
WNT3
WRB
WSB1
WSCD1
WTAP
WWC3
XAF1
XK
XKR6
XPNPEP2
XPO1
XPO5
XPO7
XPOT
XPR1
XRCC3
XRCC5
XRN1
XRN2
XYLB
YAF2
YAP1
YARS
YARS2
YBX2
YEATS2
YEATS4
YIF1B
YJEFN3
YME1L1
YPEL2
YRDC
YTHDC1
YWHAB
YWHAQ
YWHAZ
ZAK
ZBED5
ZBTB16
ZBTB2
ZBTB20
ZBTB22
ZBTB24
ZBTB3

ZBTB33
ZBTB47
ZC3H14
ZC3H8
ZC3HAV1
ZC3HC1
ZC4H2
ZCCHC24
ZDHHC13
ZDHHC14
ZDHHC15
ZDHHC22
ZDHHC23
ZFAND1
ZFAND2A
ZFHX3
ZFP14
ZFP36
ZFP36L2
ZFP42
ZFP62
ZFP91
ZFR2
ZFY
ZFYVE27
ZGPAT
ZHX1
ZIC2
ZIC3
ZIK1
ZKSCAN2
ZMAT3
ZMAT4
ZMYM5
ZMYND12
ZMYND8
ZNF101
ZNF134
ZNF138
ZNF146
ZNF165
ZNF167
ZNF175
ZNF18
ZNF189
ZNF195
ZNF197
ZNF20
ZNF200
ZNF204

ZNF215
ZNF217
ZNF226
ZNF232
ZNF239
ZNF256
ZNF268
ZNF273
ZNF274
ZNF275
ZNF280B
ZNF280C
ZNF280D
ZNF281
ZNF286A
ZNF295
ZNF296
ZNF30
ZNF300
ZNF311
ZNF324
ZNF330
ZNF331
ZNF343
ZNF35
ZNF354A
ZNF383
ZNF384
ZNF385D
ZNF391
ZNF394
ZNF395
ZNF398
ZNF423
ZNF428
ZNF430
ZNF442
ZNF444
ZNF451
ZNF467
ZNF483
ZNF485
ZNF493
ZNF502
ZNF503
ZNF512B
ZNF519
ZNF524
ZNF525
ZNF544

ZNF551
ZNF558
ZNF567
ZNF57
ZNF585A
ZNF589
ZNF608
ZNF614
ZNF620
ZNF624
ZNF625
ZNF626
ZNF627
ZNF643
ZNF644
ZNF649
ZNF653
ZNF669
ZNF672
ZNF673
ZNF675
ZNF679
ZNF681
ZNF682
ZNF688
ZNF69
ZNF695
ZNF697
ZNF702P
ZNF74
ZNF746
ZNF750
ZNF761
ZNF77
ZNF770
ZNF788
ZNF789
ZNF800
ZNF827
ZNF83
ZNF84
ZNF844
ZNF845
ZNF85
ZNF91
ZNF93
ZNFX1
ZNHIT1
ZNHIT3
ZNHIT6

ZSCAN10
ZSCAN12
ZSCAN2
ZSCAN21
ZSCAN4
ZSWIM1
ZWILCH
ZXDB
ZXDC
ZZEF1

Primary antibodies				
**Human antigene**	**Species raised in**	**Company**	**Catalog Nr.**	**Dilution**
OCT4	mouse	Santa Cruz Biotechnology	sc-5279	1:200
SOX17	goat	R&D Systems	AF1924	1:100
alpha fetoprotein (AFP)	mouse	Sigma-Aldrich	WH0000174M1	1:300
E-Cadherin (E-CAD)	mouse	Santa Cruz	sc-21791	1:200
Albumin (ALB)	mouse	Sigma-Aldrich	A-6684	1:200
HNF4a	mouse	Santa Cruz	sc-8987	1:200
A1AT	mouse	R&D Systems	MAB1268	1:200
BSEP	goat	Santa Cruz	sc-17292	1:200

Secondary antibodies				
**Antigene**	**Species raised in**	**Company**	**Catalog Nr.**	**Dilution**
anti-goat IgG, Alexa fluor 488	donkey	Invitrogen	A11055	1:300
anti-goat IgG, Alexa fluor 594	chicken	Invitrogen	A21468	1:300
anti-mouse IgG, Alexa fluor 488	goat	Invitrogen	A11001	1:300
anti-mouse IgG, Alexa fluor 594	goat	Invitrogen	A11005	1:300

Supplementary Table S11: Quantitative real-time PCR (QPCR) primer sequences.

**Gene**	**Size (bp)**	**Primer**	**Sequence 5`-3`**
OCT4	119	forward	GTGGAGGAAGCTGACAACAA
		reverse	ATTCTCCAGGTTGCCTCTCA
GAPDH	81	forward	CTGGTAAAGTGGATATTGTTGCCAT
		reverse	TGGAATCATATTGGAACATGTAAACC
SOX2	78	forward	GTATCAGGAGTTGTCAAGGCAGAG
		reverse	TCCTAGTCTTAAAGAGGCAGCAAAC
NANOG	78	forward	CCTGTGATTTGTGGGCCTG
		reverse	GACAGTCTCCGTGTGAGGCAT
LEFTY1	76	forward	AATGTGTCATTGTTTACTTGTCCTGTC
		reverse	CAGGTCTTAGGTCCAGAGTGGTG
GDF3	96	forward	TTGGCACAAGTGGATCATTGC
		reverse	TTGGCACAAGTGGATCATTGC
FGF4	109	forward	CCCTTCTTCACCGATGAGTGC
		reverse	CATTCTTGCTCAGGGCGATG
DPPA4	91	forward	TGGTGTCAGGTGGTGTGTGG
		reverse	CCAGGCTTGACCAGCATGAA
DNMT3B	93	forward	GCTCACAGGGCCCGATACTT
		reverse	GCAGTCCTGCAGCTCGAGTTTA
SOX17	86	forward	ACGTGTACTACGGCGCGATG
		reverse	CTGGTGCTGGTGCTGGTGTT
FOXA2	97	forward	TTCAGGCCCGGCTAACTCTG
		reverse	CCTTGCGTCTCTGCAACACC
HNF4α	102	forward	GTGCGGAAGAACCACATGTACTC
		reverse	GAAGCATTTCTTGAGCCTGCAGTA
AFP	88	forward	AGCAGCTTGGTGGTGGATGA
		reverse	CCTGAGCTTGGCACAGATCCT
ALB	77	forward	GCGCAGATGACAGGGCGGAA
		reverse	GTGCCGTAGCATGCGGGAGG
A1AT	76	forward	GGTCACAGAGGAGGCACCC
		reverse	AGTCCCTTTCTCGTCGATGGT
TBX3	88	forward	AGTCCTCCAGTGAACAAGCAG
		reverse	TCTTTGAGGTTCGATGTCCC
FAH	79	forward	CGGGCCGGAGCCAGAAAAC
		reverse	ACCATTCCCCAGGTCTATG
TDO2	77	forward	GGTGAAAGACGGCTGTCATACAG
		reverse	TGGAACCTAGGCTCTTCCCTG
PROM1/CD133	69	forward	GACTTGCGAACTCTCTTGAATGA
		reverse	GGTAGTGTTGTACTGGGCCAAT
LGR5	118	forward	TTTGGACAAGGGAGACCTGGAGAA
		reverse	AGAGGAGAAGGACAAGAAAGCCACA
CYP1A1	195	forward	AAACAGGGCCACATAGATGC
		reverse	AGGGTCCTGGTTTGGCTAGT
CYP3A4	79	forward	GTGACTTTGCCCATTGTTTAGAAAG
		reverse	CAGGCGTGAGCCACTGTG
CYP3A7	77	forward	GATTCTGTACGTGCATTGTGCTC

		reverse	ATTTGGTCATCTCCTCTATATTACCAAGT
CYP11A1	83	forward	CTGCATCTTCAGTCGTCTGTCC
		reverse	GGTGACCACTGAGAACCCATTC
CYP19A1	100	forward	TGGCTGTGCAGGAAAGTACATC
		reverse	AACACACTGTCCTTGCAATGTCTTC
